# Prospective Identification of Malaria Parasite Genes under Balancing Selection

**DOI:** 10.1371/journal.pone.0005568

**Published:** 2009-05-15

**Authors:** Kevin K. A. Tetteh, Lindsay B. Stewart, Lynette Isabella Ochola, Alfred Amambua-Ngwa, Alan W. Thomas, Kevin Marsh, Gareth D. Weedall, David J. Conway

**Affiliations:** 1 Department of Infectious and Tropical Diseases, London School of Hygiene and Tropical Medicine, London, United Kingdom; 2 MRC Laboratories, Fajara, Banjul, The Gambia; 3 KEMRI Centre for Geographic Medicine Research, Coast, Kilifi, Kenya; 4 Biomedical Primate Research Centre, Rijswijk, The Netherlands; 5 School of Biological Sciences, University of Liverpool, Liverpool, United Kingdom; Queensland Institute of Medical Research, Australia

## Abstract

**Background:**

Endemic human pathogens are subject to strong immune selection, and interrogation of pathogen genome variation for signatures of balancing selection can identify important target antigens. Several major antigen genes in the malaria parasite *Plasmodium falciparum* have shown such signatures in polymorphism-versus-divergence indices (comparing with the chimpanzee parasite *P. reichenowi*), and in allele frequency based indices.

**Methodology/Principal Findings:**

To compare methods for prospective identification of genes under balancing selection, 26 additional genes known or predicted to encode surface-exposed proteins of the invasive blood stage merozoite were first sequenced from a panel of 14 independent *P. falciparum* cultured lines and *P. reichenowi*. Six genes at the positive extremes of one or both of the Hudson-Kreitman-Aguade (HKA) and McDonald-Kreitman (MK) indices were identified. Allele frequency based analysis was then performed on a Gambian *P. falciparum* population sample for these six genes and three others as controls. Tajima's D (TjD) index was most highly positive for the *msp3/6*-like *PF10_0348* (TjD = 1.96) as well as the positive control *ama1* antigen gene (TjD = 1.22). Across the genes there was a strong correlation between population TjD values and the relative HKA indices (whether derived from the population or the panel of cultured laboratory isolates), but no correlation with the MK indices.

**Conclusions/Significance:**

Although few individual parasite genes show significant evidence of balancing selection, analysis of population genomic and comparative sequence data with the HKA and TjD indices should discriminate those that do, and thereby identify likely targets of immunity.

## Introduction

Dynamic interactions between hosts and pathogens result in positive selection on molecules responsible for pathogen invasion, host resistance, and pathogen evasion of host resistance [1]–[3]. Many surface protein genes reveal signatures of positive selection, with several clear examples in malaria parasites [4]–[11]. These include signatures of directional selection that increases fixation rates and divergence among populations and species [7]–[9] and balancing selection that maintains diversity within local populations [4]–[6]. Although heterozygote advantage might operate during the brief gamete fertilization and diploid stages inside the mosquito host, balancing selection on proteins in the haploid asexual blood stage is probably due to negative frequency-dependent immune selection [12]–[18]. Predictions that blood-stage proteins under balancing selection are important targets of acquired immunity have been supported by antibody inhibition assays in culture [19]–[22], and by studies of naturally acquired antibodies and incidence of clinical malaria in endemic populations [17], [23]–[25].

The ∼23 Mb *P. falciparum* genome that encodes ∼5300 proteins presents a challenge for identifying targets of immunity, but scans of currently available genome sequence data from different isolates can already identify loci with unusually high levels of polymorphism [5]–[7]. With available data, such scans do not discriminate loci under transient directional selection (such as drug resistance genes) [5], from those under balancing selection [6]. In parallel with the increasing availability of data on genome sequence diversity, there have been many developments of tests for evidence of positive directional selection [26], [27], but less focus on identifying genes under balancing selection [28]. The data requirements of different tests vary, so choices among these should determine the strategic sampling of parasite isolates for whole genome sequencing. Allele frequency based tests require sequences of many isolates from at least one defined population for Tajima's D (TjD) index [29], [30], or multiple populations for Wright's fixation (*F*_ST_) indices [31], while polymorphism-versus-divergence tests such as the Hudson-Kreitman-Aguade (HKA) [32] or McDonald-Kreitman (MK) [33] indices can be performed on fewer isolate sequences but require comparator sequence from a closely-related species.

To evaluate indices for large scale identification of genes under balancing selection, analysis was performed on a prospective sample of genes encoding surface-accessible proteins in a single parasite stage. Gene transcription and proteomic data on the merozoite of *P. falciparum* [34] and ongoing identification of proteins specifically located on the surface or in the apical organelles [35]–[37], allows components of this important erythrocyte invasive stage to be investigated. Studies comparing different members of small gene families expressed at this stage, including five *eba* [15], [16], three *Rh* [38] and five *RhopH1/Clag* [39] genes had previously shown how variable and locus-specific the signatures of selection are. The present study investigates a prospective panel of twenty six additional merozoite protein-coding genes, by sequencing from diverse laboratory cultured *P. falciparum* isolates and *P. reichenowi* to enable polymorphism-versus-divergence tests. A subset of the genes, together with positive and negative controls, was then sequenced from an endemic population sample in The Gambia to give an allele frequency based analysis with independent data. The HKA and TjD indices with the respective types of data sets are promising for large-scale analyses to detect the important minority of all parasite genes that are under balancing selection.

## Results

### Polymorphism and divergence analyses

**Figure 1:**
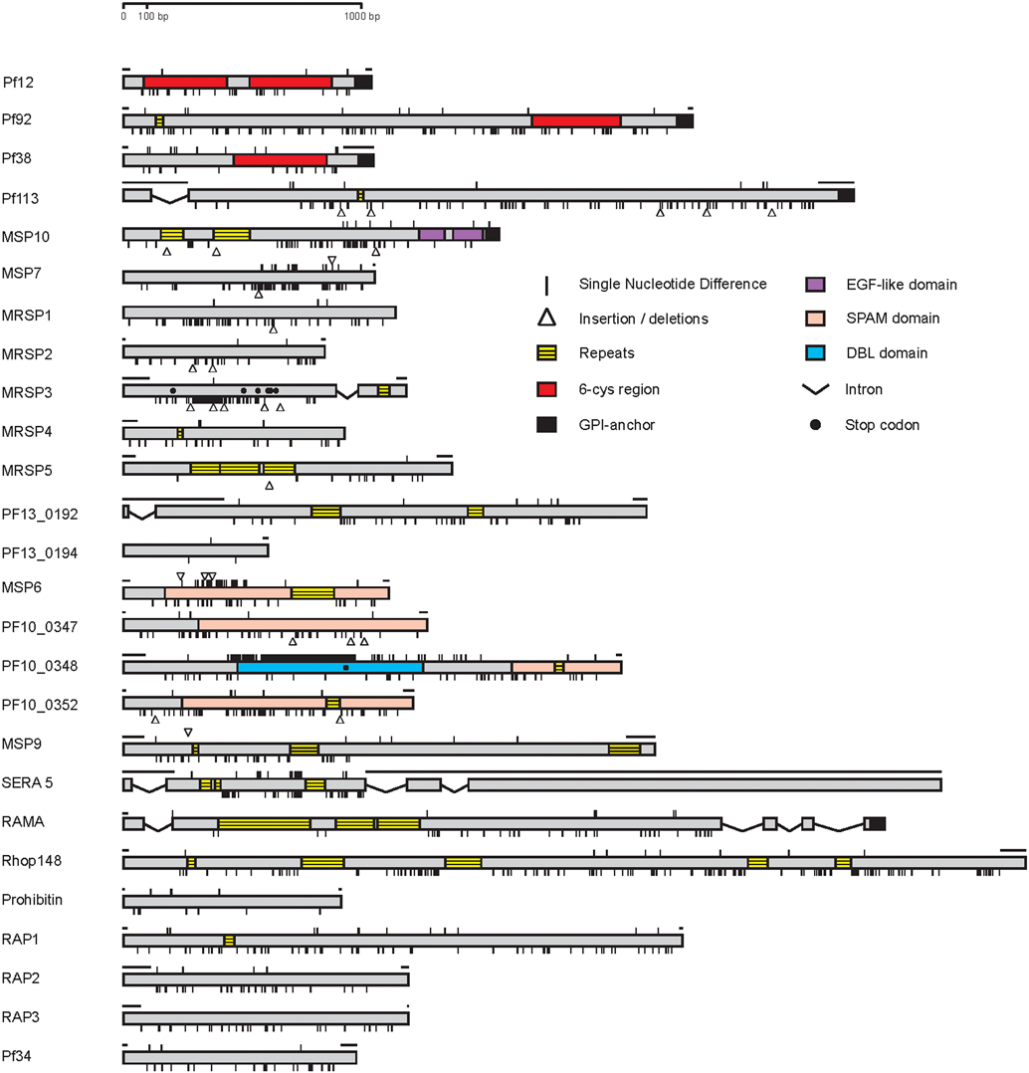
Scheme of the 26 genes studied showing positions of individual nucleotide polymorphisms among 14 *P. falciparum* isolates as vertical lines above each gene. Fixed differences between *P. falciparum* and *P. reichenowi* are shown as vertical lines below each gene. Positions of insertions, deletions, and repetitive sequence are also indicated on each gene, as well as verified and predicted protein domains. Stop codons within genes are shown for MRSP3 (in *P reichenowi* only) and for PF10_0348 (in *P. falciparum* R033 isolate). Horizontal bars above some genes indicate portions not analysed.

A screen for signatures of non-neutrality was first applied to a set of 26 genes known or predicted to encode surface-exposed proteins of the merozoite stage of the parasite. Alleles of each of the genes were sequenced from 14 cultured lines of *P. falciparum*, representing species-wide polymorphism, along with the *P. reichenowi* orthologue of each gene (Accession numbers are listed in Supplementary Table S1). Figure 1 shows the positions of insertions, deletions, and nucleotide polymorphisms and fixed differences between the species, as well as repetitive sequences (omitted from alignment-based analyses). Full alignments of the sequences are shown in Supplementary Figures S1, S2, S3, and the repetitive sequences in 15 of the genes are shown in Supplementary Figure S4. For one gene (*MRSP3*) there was a stop codon in the *P. reichenowi* orthologue, and for another (*PF10_0348*) there was a stop codon in the allele of *P. falciparum* clone RO33; for analysis, these stop codons were removed and the remainder of each sequence was included in frame. For *PF10_0348*, three of the *P. falciparum* isolates unexpectedly contained two distinct gene sequences, one of which was identical across the three isolates but different from all others (alignment shown in Supplementary Figure S5); this extra sequence was termed ‘copy B’ and omitted from analyses.

Table 1 shows the overall nucleotide diversity (π) and inter-specific divergence (K) indices for each gene, and the HKAr polymorphism-versus-divergence index (π/K ratio). The π values per gene ranged from 0 (no polymorphism in *rap3* or the *msp7-like* gene *PF13_0194*) up to 0.0568 (the highly polymorphic *msp3/6-like* gene *PF10_0348*). If the repeat sequences had not been identified and appropriately removed prior to analysis, the apparent diversity (π) values for several genes would have been elevated (Supplementary Figure S6). Also shown are the numbers of synonymous and nonsynonymous polymorphisms among the aligned *P. falciparum* alleles, and fixed differences from the *P. reichenowi* orthologues, together with the results of the MK test on these proportions.

**Table 1:** HKAr indices summarizing polymorphism (π, among 14 *P. falciparum* laboratory isolates) and divergence (K, from *P. reichenowi*) of 26 merozoite stage genes together with MK tests on synonymous (syn) and nonsynonymous (non-syn) polymorphic and fixed differences.

Gene	Locus	Nt	*π* (10^−3^)	K (10^−3^)	HKAr (*π*/K)	Syn		Non-syn		MK test
						Fixed	Poly	Fixed	Poly	P value
*Pf12/6-cys*	PFF0615c	990	1.0	35.9	0.028	12	0	23	3	0.54
*Pf92/6-cys*	Pf13_0338	2319◊	1.2	41.6	0.029	39	0	53	9	***0.01***
*Pf38/6-cys*	PFE0395c	900	3.4	21.3	0.160	6	0	11	11	0.06
*Pf113*	PF14_0201	2619◊	0.5	30.6	0.016	31	1	48	6	0.25
*MSP10*	PFF0995c	1329◊	1.7	45.6	0.037	15	0	44	8	0.18
*MSP6*	PF10_0346	867◊	17.5	59.3	0.295	5	3	27	35	0.46
*MSP3/6-like*	PF10_0347	1230	1.1	37.2	0.030	14	1	30	3	1.00
*MSP3/6-like*	PF10_0348	1935◊•^b^	56.8	82.8	0.686	16	72	58	167	0.18
*MSP3/6-like*	PF10_0352	1107◊	0.9	54.5	0.017	14	2	44	4	0.64
*MSP7*	PF13_0197	1044	5.6	59.8	0.094	13	1	44	17	0.17
*MRSP1*	PF13_0196	1143^b^	0.4	43.8	0.009	8	0	41	3	1.00
*MRSP2*	MAL13P1.174	819^b^	0.5	36.9	0.014	10	1	19	1	1.00
*MRSP3*	PF13_0193	897◊•	0.2	53.9	0.028	6	0	32	0	-
*MRSP4*	MAL13P1.173	897◊	1.2	26.9	0.045	11	0	12	4	0.12
*MRSP5*	Pf13_0191	870◊	0.3	19.7	0.015	7	0	10	1	1.00
*MRSP-like*	PF13_0192	1446◊	1.1	29.6	0.037	8	0	33	5	0.57
*MRSP-like*	PF13_0194	567	0.0	5.3	0.000	1	0	2	0	-
*MSP9/ABRA*	PFL1385c	1761◊^b^	1.4	48.2	0.029	29	0	54	6	0.17
*SERA 5*	PFB0340c	609◊^b^	16.0	98.2	0.163	7	4	32	23	1.0
*RAMA*	MAL7P1.208	1326◊	1.0	26.8	0.036	10	1	23	5	0.66
*Rhop148*	PF13_0348	3132◊	0.7	45.1	0.016	64	1	76	9	***0.04***
*Prohibitin*	PF10_0144	894	0.8	9.0	0.089	5	2	2	2	0.58
*RAP1*	PF14_0102	2271◊^a^	2.0	29.1	0.069	18	1	45	11	0.17
*RAP2*	PFE0080c	1053^a^	1.5	29.5	0.051	8	0	22	3	0.56
*RAP3*	PFE0075c	1119	0.0	26.8	0.000	17	0	13	0	-
*Pf34*	PFD0955w	894^b^	0.5	28.5	0.018	13	0	12	3	0.23

Nt, number of aligned nucleotides.

◊, repeats removed from gene sequences.

•, internal stop codon in *P. reichenowi mrsp3* gene, and in an allele of *P. falciparum* PF10_0348 (codons removed from analysis).

a complex codons in *rap1* and *rap2* not analysed.

b less sequence aligned when *P.reichenowi* added (Pf10_0347 N = 1200, Pf10_0348 N = 1866, Pf10_0352 N = 1104, MRSP1 N = 1140, MRSP2 N = 816, MRSP3 N = 705, MSP9 N = 1758, SERA5 N = 561, Pf34 N = 882); Sequences submitted to Genbank (Accession numbers are listed in Supplementary Table S1).

**Figure 2:**
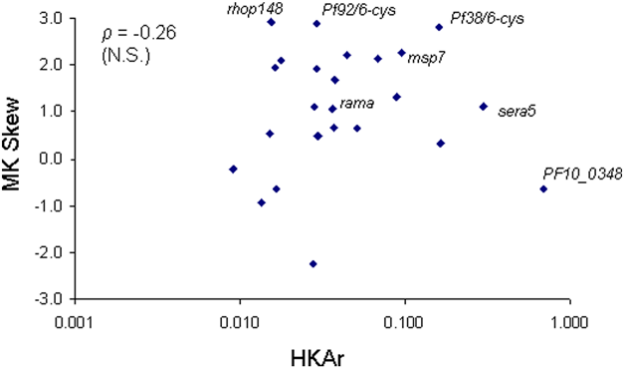
Scatterplot of McDonald-Kreitman (MK) skew (log_2_ transformation of the neutrality index with 0 representing no skew) and Hudson-Kreitman-Aguade ratio (HKAr) of polymorphism (in *P. falciparum*) and divergence (between *P. falciparum* and *P. reichenowi*) for each of the genes studied in Table 1. Two of the 26 genes had no polymorphism and are not plotted. There was no significant correlation between the two indices (Spearman's *ρ* = −0.26). Six genes at the outer fringe of the distributions, and one in the middle, are labelled and were selected for population based analysis.

Both HKAr and MK indices express polymorphism relative to divergence, although HKAr does this directly while MK skew expresses the imbalance in the ratios of nonsynonymous and synonymous polymorphisms versus fixed differences. Over all the genes there was no correlation between the HKAr and MK indices (Spearman's *ρ* = −0.26, P = 0.23) (Figure 2). The three genes showing the highest HKAr indices were the *msp3/6-like* gene *PF10_0348*, *msp7*, and *sera5*. Three other genes showed the most positively skewed MK indices (with higher ratio of nonsynonymous to synonymous polymorphisms than fixed differences), two of which were significant (*Pf92/6-cys* and *rhop148*, P<0.05), and one nearly significant (*Pf38/6-cys*; P = 0.06) (Table 1).

### Allele frequency based tests in an endemic *P. falciparum* population sample

The six genes with extreme positive values of either the HKAr or MK indices noted above were chosen for sequencing from a Gambian *P. falciparum* population, to allow application of allele frequency-based analyses. A gene that was previously shown to be under balancing selection in other populations (*ama1*, *PF11_0344*) was incorporated as a positive control, and a merozoite stage-specific gene encoding an internally expressed protein (*etramp10.2*, *PF10_0323*) was included as a negative control as well as one of the genes that had neither an extreme MK skew nor HKA index in the analysis above *(rama)*.

**Figure 3:**
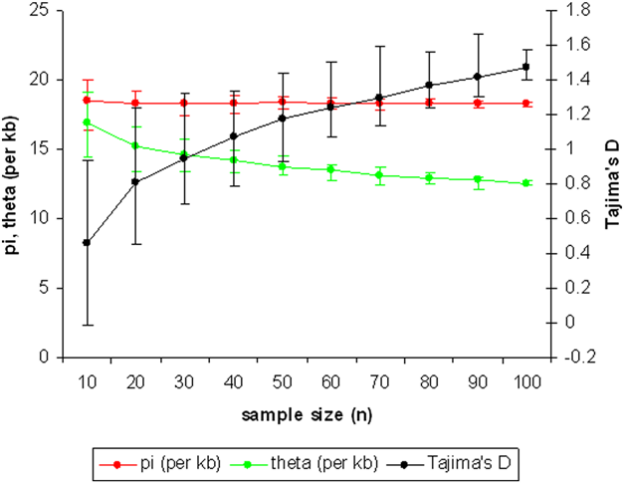
Sample size optimisation for Tajima's D test assessed using a large sample of *ama1* gene sequences from a Gambian *P. falciparum* population. 100 random samples of each size (n) were taken from a set of 114 *ama1* sequences covering the region encoding the ectodomain of the protein (nucleotides 442–1743). The median values and 95% confidence limits (3^rd^ and 97^th^ centile values of 100 estimates) of *π*, *θ*, and Tajima's *D* indices were calculated for each sample size.

First, for the positive control gene *ama1*, a sample of 114 allele sequences was derived from the Gambian population, and random subsets of the data were sampled to examine the relationship between sample size and Tajima's D (TjD) value. This showed that there is an increase in the point estimate of TjD with increasing sample size, but that above a sample size of ∼50 alleles the rate of increase becomes much slower (Figure 3). Therefore, to obtain a minimum sample size of 50 allele sequences per gene, amplification and direct sequencing was performed from a random panel of 89 of the Gambian isolates together with positive and negative control samples in a 96-well array. After the expected dropout of some isolates with mixed allele sequences that produced superimposed electropherogram traces (particularly a problem for reading sequences of genes with variable repeat lengths), the numbers of allele sequences obtained ranged from 56 (for *sera5*) to 88 (for *Pf38/6-cys*). (Accession numbers are listed in Supplementary Table S2).

**Figure 4:**
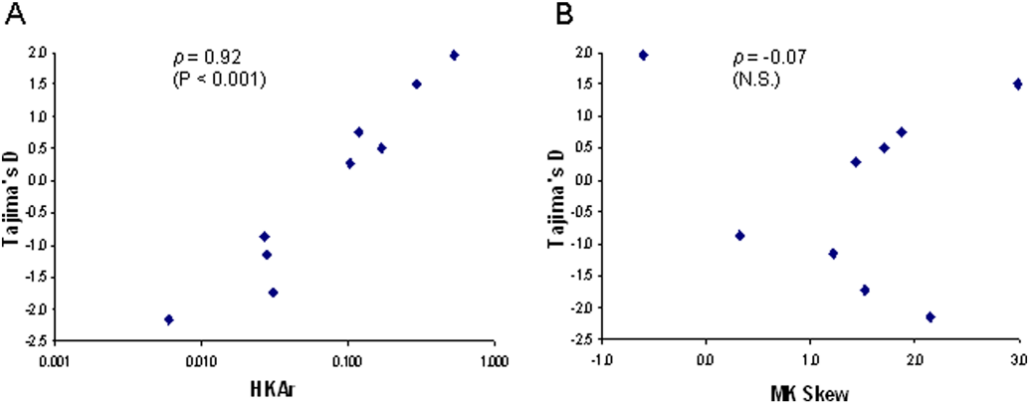
Correlations between the allele frequency-based Tajima's D index and two different polymorphism-versus-divergence indices for 9 merozoite stage genes (N>55 alleles of each gene sampled from a Gambian *P. falciparum* population, and interspecies comparison with *P. reichenowi*). A. Scatterplot showing strong positive correlation (Spearman's *ρ* = 0.92, P<0.001) between the HKA ratio (HKAr) index and the allele frequency-based Tajima's D index. B. Scatterplot indicating no correlation (Spearman's *ρ* = −0.07, N.S.) between the McDonald Kreitman (MK) skew and Tajima's D index.

Table 2 shows that the TjD value was positive for the *ama1* control, and negative for the *etramp10.2* and *rama* controls, as expected (see Discussion). The value for one of the test genes was highly positive (the *msp3/6-like* gene *PF10_0348*). Values for other genes were either modestly positive (for *sera5*, *Pf38* and *msp7*), or negative. Across the 9 genes tested in the population, there was a strong positive correlation between the TjD index and the HKAr index (Spearman's *ρ* = 0.92, P<0.001; Figure 4A), but not with the MK index (*ρ* = −0.07, P = 0.87; Figure 4B). Indeed, apart from *ama1*, the two genes that had the most positive MK skew (*Pf92/6-cys*, and *rhop148*) had the most negative TjD indices, illustrating that positively skewed MK indices are not commonly due to balancing selection. Fu and Li's F index correlated strongly with TjD (*ρ* = 0.97, p<0.001), and had similar correlations as TjD with the other indices (significant for HKAr, *ρ* = 0.88, P = 0.002; not significant for MK, *ρ* = 0.00, P = 1.0). For the 7 genes included in both data sets (Table 1 and Table 2), TjD indices from the Gambian population correlated with the HKAr indices calculated with the lab isolates (*ρ* = 0.79, P = 0.036), but did not correlate with the MK indices (*ρ* = −0.57, P = 0.18).

**Table 2:** Analysis of polymorphism in 9 merozoite stage expressed *P. falciparum* genes in a Gambian population sample.

Gene	Locus	No. of isolates	Nt	π (×10^3^)	K (×10^3^)	HKAr (*π*/K)	McDonald-Kreitman					Tajima's D	Fu & Li F
							SYN		NONSYN		MK		
							Fixed	Poly	Fixed	Poly	p-value		
*MSP3/6-like*	PF10_0348	66	1896◊•^c^	38.7	72.3	0.535	17	68	57	150	0.18	*1.96* *	*2.24* **
*msp7*	PF13_0197	85	1050^c^	6.4	62.0	0.103	13	3	45	28	0.16	0.28	0.19
*rhop148*	PF13_0348	81	1200◊^a^	0.3	47.7	0.006	30	2	27	8	0.09	*−2.16* *	*−4.02* **
*sera5*	PFB0340c	56	624◊^c^	11.2	93.6	0.119	11	2	36	24	0.12	0.76	0.75
*Pf38/6-cys*	PFE0395c	88	903	3.6	21.3	0.169	6	2	11	12	0.24	0.50	0.55
*Pf92/6-cys*	PF13_0338	87	2319◊	1.3	41.9	0.031	37	6	56	26	0.06	−1.73	*−4.15* **
*Rama*	MAL7P1.208	68	1323◊	0.7	26.4	0.027	11	2	22	5	1.00	−0.87	−1.98
*Etramp10.2* (−ve control)	PF10_0323	78	990	1.4	50.0	0.028	14	2	33	11	0.48	−1.37	−1.15
*ama1* (+ve control)	PF11_0344	114	1302^b^	16.3	40.0	0.420	11	3	21	61	0.0002 ***	*1.50*	*1.70*

Nt, number of aligned nucleotide positions analysed.

•, stop codon in 7 alleles of PF10_0348 (codon removed from analysis).

◊, repeats removed from gene sequences for analysis.

a only one region of *msp7* gene (of most dense polymorphism) was studied in the population.

b 5 complex codons in *ama1* not analysed.

c less sequence aligned when *P. reichenowi* added (Pf10_0348 N = 1824, *msp7* N = 1041, *sera5* N = 600).

* p<0.05.

** p<0.01.

*** p<0.001.

Values significantly different to 0 are shown in italics; for *ama1* Tajima's D is significant for Domain II (p<0.05) and Domain III (p<0.001), and Fu and Li's F is significant for Domain III (p<0.02); Sequences submitted to Genbank (Accession numbers for population datasets of allele sequences are listed in Supplementary Table S2).

## Discussion

This analysis of a large panel of merozoite stage-specific genes for signatures of balancing selection enables recommendations for scaling up to whole genome analyses. The advantage of using more than one test method is illustrated, particularly where it is possible to perform polymorphism-versus-divergence analysis as well as allele frequency-based analysis, ideally with independent datasets.

Although the MK test was originally developed to detect positive directional selection that has led to differences between species (thus displaying a negative skew in the ratios) [33], it has previously shown a very positive skew for some antigen genes that are under strong balancing selection (e.g. *ama1*, *eba175*, *trap*) [13], [15], [18]. However, it did not perform well here for prospectively identifying additional genes under such selection. Genes under weak negative selection (suppressing fixation of nonsynonymous changes between species lineages but allowing nonsynonymous polymorphism at low frequency) would have a superficially similar skew in the MK test to that caused by balancing selection [40], but would be associated with low rather than high TjD values [41], [42], as seen here for the genes *Pf92/6-cys*, and *rhop148*. Another limitation of the MK test is that its power is very low for analysis of most *P. falciparum* genes, as the number of polymorphic sites is low in most genes [7], so splitting these further into nonsynonymous and synonymous classes can exceed the limitations of the data.

The HKAr index utilizes the overall polymorphism-versus-divergence data in a manner that is more efficient for the present purpose. The HKAr indices here did not correlate with the MK indices for the same set of data, but did correlate strongly with the allele frequency-based TjD index in independent data. This is encouraging for the application of both HKAr (to data from disparate laboratory cultured lines or a population sample) and TjD (requiring a population sample) which are informative but have some limitations when used alone [27]. The TjD test is influenced not only by selection but also by the population history that can alter neutral allele frequency expectations [43], as in the case of previous population expansion that causes the neutral index for *P. falciparum* to be negative rather than zero [15], [44]. This makes the test for balancing selection conservative, and it is possible that the modest positive TjD values here for *sera5*, *Pf38/6-cys*, and *msp7* also reflect balancing selection. The sampling distribution of neutral values could be re-estimated by modeling past population growth parameters, which would lead to negative neutral values of TjD [44] and thus increase the sensitivity of the test for genes under balancing selection. Although we have shown elsewhere how such modifications can potentially benefit the application of the TjD test to *P. falciparum* genes [15], here we retain a conservative approach by testing TjD values under a constant population size model, as our primary aim is to compare among different types of standard tests with minimal modifications. The between-species divergence (used as denominator for the HKAr index) is less sensitive to the effects of demographic history than the number of polymorphic nucleotides in a population sample (used towards both the numerator and denominator of the TjD index). Therefore, concordant high values of HKAr and TjD indices for these genes are very supportive of balancing selection, encouraging a two-dimensional approach to identifying genes under such selection [45].

Of the panel of malaria parasite merozoite stage genes prospectively investigated here, the one with the strongest signature of balancing selection, as indicated by the HKAr as well as the TjD index, was *PF10_0348* (a member of the *msp3/6*-like family). This gene had two unusual features, however. Firstly, one of the laboratory isolates and a minority of field isolates had an internal stop codon, so these may be null with regard to protein expression. Secondly, a minority of isolates contained a second, more divergent, *PF10_0348*-like sequence that does not match with other known loci, and although this was not included in the analysis it suggests there are paralogous genes in some parasites. Further studies of *PF10_*0348 are needed, including characterization of its transcription and protein expression. Generally, if a protein is known to be encoded by multiple gene copies that undergo gene conversion or ectopic recombination, non-classical approaches to analyzing sequence polymorphism may need to be developed [46], [47], and it is possible that deeper sequencing of *P. falciparum* genomes will reveal this to be the case for more merozoite stage genes than has yet been appreciated [38], [39], [48].

It should be noted that any test, or combination of tests, may be conservative if a protein is under positive directional selection as well as balancing selection. Previous data indicate this to be the case for the erythrocyte binding antigen EBA175, a merozoite ligand adapted for binding to species-specific receptor structures on host glycophorin A (leading to an excess of sequence substitutions between species) [49] and also under selection from acquired immune responses (leading to an excess of polymorphisms) [15], [16]. Different types of selection that lead towards fixed differences or maintenance of allelic diversity should be discriminated where possible by information on protein function or antigenicity, as their co-occurrence limits the power of general tests.

Means of scaling up to a comprehensive investigation of balancing selection in a pathogen can be determined by available funding, accessibility of appropriate genome samples, as well as the epidemiological and population genetic structure of the pathogen. Capillary sequencing is rate-limiting for large population analyses of many genes, so it can be efficient to focus such analysis on genes with more than a minimal amount of polymorphism, and for *P. falciparum* initial screening can now be performed using emerging genome sequence diversity data, accessible through PlasmoDB (www.plasmodb.org) [5]–[7]. However, solid phase ‘next generation’ sequencing methods should soon allow whole genome sequencing to be performed on population samples [50]. As such methods can now be successfully applied to *P. falciparum* genome sequencing [51], it is possible that >50 isolates from an endemic population could be realistically sequenced for a complete genome screen to detect signatures of balancing and directional selection. Subsequent comparison of allele frequency distributions in multiple population genomic samples could further test these signatures, and such data could also be used to refine less intensive approaches to identifying selection in pathogens with large genomes.

## Materials and Methods

### Ethics Statement

Written informed consent was given by the parents of each child, and verbal assent by each child, for collection of a <5 ml venous blood sample for analyses including investigation of malaria parasite DNA. The study and protocol was approved by the Scientific Co-ordinating Committee and the Ethics Committee of the MRC Gambia Unit and the Gambian Government.

### Gene sequencing from *P. falciparum* cultured isolates and *P. reichenowi*

Twenty six merozoite stage-expressed genes were chosen for analysis as they encode surface or apical organelle proteins in *P. falciparum* merozoites, or are predicted to do so by close homology with proteins that are so localized. These encode five surface proteins that are GPI-anchored (*Pf12*, *Pf38*, *Pf92*, *Pf113*, and *msp10*), 14 known or predicted to be surface associated but not membrane anchored (4 members of the *msp3/6*-like family: *msp6*, *PF10_0347*, *PF10_0348*, *PF10_0352*; 6 members of the *msp7*-like family: *msp7*, *mrsp1*, *mrsp2*, *mrsp3*, *mrsp4*, *mrsp5*; and 4 others: the *msp7*-linked *PF13_0192* and *PF13_0194*, and the unlinked *sera5* and *msp9*), and 7 expressed in the apical rhoptry organelle (*rama*, *rhop148*, *rap1*, *rap2*, *rap3*, *prohibitin*, *Pf34*). Each gene was amplified from genomic DNA of 14 genotypically distinct cultured *P. falciparum* lines from diverse original sources (3D7, cloned from an airport malaria case in The Netherlands; D6, RO33 and Palo Alto, from Africa; K1, T9/96, T9/102, Dd2, FCC2 and D10, from Southeast Asia; FCR3 and Wellcome, nominally from Africa but suspected to have been previously cross-contaminated by parasites of unknown source during culture; HB3 from Honduras; 7G8 from Brazil) and the only existing known isolate of *P. reichenowi* (CDC-1 strain isolated over 50 years ago from a chimpanzee from the Belgian Congo), using primers and amplification conditions listed in Supplementary Tables S3 and S4. PCR products were purified with the QIAquick PCR purification kit (QIAGEN, UK), and sequenced using the outer amplification primers and several internal sequencing primers, using BIG DYE terminator v3.1 chemistry (Applied Biosystems, UK) and an ABI 3130xl capillary sequencer (Applied Biosystems, UK). Sequences were assembled, edited and aligned using SeqMan and MegAlign (DNASTAR, Madison, WI). The data covered the complete or near complete coding sequence of each of the 26 genes, except *sera5* for which analysis was focused on the most polymorphic exon 2.

### Gene sequencing from a Gambian *P. falciparum* population

Parasites were studied from children aged <13 years living in the coastal urban/peri-urban area within 40 km south of Banjul who presented with *P. falciparum* malaria to the Medical Research Council (MRC) outpatients clinic at Fajara, or to the Royal Victoria Teaching Hospital in Banjul during a single malaria season (between September 2005 and January 2006). Heparinised venous blood samples were collected and centrifuged to remove plasma and leukocytes for other studies, and erythrocytes were then washed three times in RPMI medium. The DNA was extracted from packed erythrocytes using the QIAamp DNA Blood Mini Kit (QIAGEN, UK). The *ama1* gene was chosen as a positive control gene as results from other populations indicate that it is consistently under balancing selection [12]–[14]. A sample of 114 *ama1* alleles from the Gambian population (one allele from each of 114 independent clinical isolates) was sequenced to enable estimation of smaller sample sizes that would be efficient for detecting positive Tajima's D values (see below). Six of the genes analysed in Table 1 (and Figure 2) that had positive HKAr or MK signatures, one that did not (*rama*), and another negative control (*PF10_0323*) encoding an internal protein, were then amplified from genomic DNA from a panel of 89 isolates, and products were directly sequenced to obtain a final sample size of >55 allele sequences of each gene (more than half of the isolates yielded a clear single allele sequence and those showing mixed sequences were discarded). All nucleotide alleles that had not been previously seen were confirmed by independent re-amplification and re-sequencing of the gene from each relevant sample.

### Tests of neutrality

Tests were performed using DnaSP4 [52]. Tajima's D (TjD) test detects departures from neutrality in allele frequency distributions by considering the number of polymorphic sites and the pairwise nucleotide diversity [29], while Fu and Li's F test is based on the number of polymorphic sites with singleton alleles [53]. The McDonald-Kreitman (MK) test [33], compares numbers of nonsynonymous (_NS_) and synonymous (_S_) nucleotide changes polymorphic (P) within species, and apparently fixed (F) between closely-related species, with a Fisher's exact test on the 2×2 contingency table. A measure of MK skew was calculated: log_2_ [(P_NS_/F_NS_)/(P_S_/F_S_)], where 0 represents no skew, positive values represent an excess of nonsynonymous polymorphism (or deficiency of nonsynonymous differences between species) and negative values represent selection against nonsynonymous polymorphism (or elevated fixation of nonsynonymous differences). The Hudson-Kreitman-Aguade ratio (HKAr) index for a gene is the pairwise nucleotide polymorphism (*π*) divided by the divergence (*K*) from a closely-related species, and is used here to identify genes with exceptionally high ratios [45].

## Supporting Information

Figure S1 (0.17 MB PDF)

Figure S2 (0.36 MB PDF)

Figure S3 (0.27 MB PDF)

Figure S4 (0.05 MB PDF)

Figure S5 (1.42 MB PDF)

Figure S6 (0.03 MB DOC)

Table S1 (0.05 MB DOC)

Table S2 (0.03 MB DOC)

Table S3 (0.06 MB DOC)

Table S4 (0.06 MB DOC)
