# Supplementary material for: Prospective Identification of Malaria Parasite Genes under Balancing Selection
Source: PLoS One. 2009 May 15;4(5):e5568. doi: 10.1371/journal.pone.0005568 (PMC2679211; doi:10.1371/journal.pone.0005568)
Supplement: Figure S1 — (0.17 MB PDF) [file pone.0005568.s001.pdf]

Supplementary Figure 1

PFF0615c

```
                                10        20        30        40        50        60        70        80
                        ....|....| ....|....| ....|....| ....|....| ....|....| ....|....| ....|....| ....|....|
PFF0615c                ATGATAAAAT TAAGTAAGAA GTATTGTTTA GGGATATCCT TTGTATTATA TATTTTGTTG TCTGTTTGTG AAGGGCATAA
3D7                                                      .......... .......... .......... .......... ..........
RO33                                                     .......... .......... .......... .......... ..........
Palo Alto                                                .......... .......... .......... .......... ..........
FCR3                                                     .......... .......... .......... .......... ..........
Wellcome                                                 .......... .......... .......... .......... ..........
D6                                                       .......... .......... .......... .......... ..........
T996                                                     .......... .......... .......... .......... ..........
T9102                                                    .......... .......... .......... .......... ..........
K1                                                       .......... .......... .......... .......... ..........
Dd2                                                      .......... .......... .......... .......... ..........
D10                                                      .......... .......... .......... .......... ..........
FCC2                                                     .......... .......... .......... .......... ..........
HB3                                                      .......... .......... .......... .......... ..........
7G8                                                      .......... .......... .......... .......... ..........
P.reichenowi            .......... ..G....... .......... .......... .......... .......... .......... .....TT...

                                90       100       110       120       130       140       150       160
                        ....|....| ....|....| ....|....| ....|....| ....|....| ....|....| ....|....| ....|....|
PFF0615c                AAATTTAACA TGTGACTTTA ACGATGTATA CAAATTAGAA TTTCATCCTA ATCAACAAAC AAGTGTTACT AAATTATGTA
3D7                     .......... .......... .......... .......... .......... .......... .......... ..........
RO33                    .......... .......... .......... .......... .......... .......... .......... ..........
Palo Alto               .......... .......... .......... .......... .......... .......... .......... ..........
FCR3                    .......... .......... .......... .......... .......... .......... .......... ..........
Wellcome                .......... .......... .......... .......... .......... .......... .......... ..........
D6                      .......... .......... .......... .......... .......... .......... .......... ..........
T996                    .......... .......... .......... .......... .......... .......... .......... ..........
T9102                   .......... .......... .......... .......... .......... .......... .......... ..........
K1                      .......... .......... .......... .......... .......... .......... .......... ..........
Dd2                     .......... .......... .......... .......... .......... .......... .......... ..........
D10                     .......... .......... .......... .......... .......... .......... .......... ..........
FCC2                    .......... .......... .......... .......... .......... .......... .......... ..........
HB3                     .......... .......... .......... .......... .......... .......... .......... ..........
7G8                     .......... .......... .......... .......... .......... .......... .......... ..........
P.reichenowi            ..G....... .......... .......... T......... .....C.... .......... ..A....... ..........

                               170       180       190       200       210       220       230       240
                        ....|....| ....|....| ....|....| ....|....| ....|....| ....|....| ....|....| ....|....|
PFF0615c                ATTTAACTCC TAATGTATTA GAAAAGGTAA CTATAAAATG TGGTTCAGAT AAATTAAATT ATAATTTATA TCCTCCAACT
3D7                     .......... .......... .......... .......... .......... .......... .......... ..........
RO33                    .......... .......... .......... .......... .......... .......... .......... ..........
Palo Alto               .......... .......... .......... .......... .......... .......... .......... ..........
FCR3                    ..G....... .......... .......... .......... .......... .......... .......... ..........
Wellcome                ..G....... .......... .......... .......... .......... .......... .......... ..........
D6                      .......... .......... .......... .......... .......... .......... .......... ..........
T996                    .......... .......... .......... .......... .......... .......... .......... ..........
T9102                   ..G....... .......... .......... .......... .......... .......... .......... ..........
K1                      .......... .......... .......... .......... .......... .......... .......... ..........
Dd2                     .......... .......... .......... .......... .......... .......... .......... ..........
D10                     .......... .......... .......... .......... .......... .......... .......... ..........
FCC2                    .......... .......... .......... .......... .......... .......... .......... ..........
HB3                     .......... .......... .......... .......... .......... .......... .......... ..........
7G8                     .......... .......... .......... .......... .......... .......... .......... ..........
P.reichenowi            .......... .......... .....A.... .A........ .......... .......... .......... ..........

                               250       260       270       280       290       300       310       320
                        ....|....| ....|....| ....|....| ....|....| ....|....| ....|....| ....|....| ....|....|
PFF0615c                TGTTTTGAAG AGGTATATGC ATCTAGGAAT ATGATGCATT TAAAAAAAAT AAAAGAGTTT GTAATCGGAT CATCAATGTT
3D7                     .......... .......... .......... .......... .......... .......... .......... ..........
RO33                    .......... .......... .......... .......... .......... .......... .......... ..........
Palo Alto               .......... .......... .......... .......... .......... .......... .......... ..........
FCR3                    .......... .......... .......... .......... .......... .......... .......... ..........
Wellcome                .......... .......... .......... .......... .......... .......... .......... ..........
D6                      .......... .......... .......... .......... .......... .......... .......... ..........
T996                    .......... .......... .......... .......... .......... .......... .......... ..........
T9102                   .......... .......... .......... .......... .......... .......... .......... ..........
K1                      .......... .......... .......... .......... .......... .......... .......... ..........
Dd2                     .......... .......... .......... .......... .......... .......... .......... ..........
D10                     .......... .......... .......... .......... .......... .......... .......... ..........
FCC2                    .......... .......... .......... .......... .......... .......... .......... ..........
HB3                     .......... .......... .......... .......... .......... .......... .......... ..........
7G8                     .......... .......... .......... .......... .......... .......... .......... ..........
P.reichenowi            .......... ........A. TCT....... .......... .......... ...T...... .......... ........C.
```

```
                              330        340        350        360        370        380        390        400
                     ....|....| ....|....| ....|....| ....|....| ....|....| ....|....| ....|....| ....|....|
PFF0615c             TATGAGACGT AGTTTAACAC CAAATAAAAT TAACGAAGTT TCTTTCAGAA TTCCACCTAA TATGATGCCT GAAAAACCTA
3D7                  .......... .......... .......... .......... .......... .......... .......... ..........
RO33                 .......... .......... .......... .......... .......... .......... .......... ..........
Palo_Alto            .......... .......... .......... .......... .......... .......... .......... ..........
FCR3                 .......... .......... .......... .......... .......... .......... .......... ..........
Wellcome             .......... .......... .......... .......... .......... .......... .......... ..........
D6                   .......... .......... .......... .......... .......... .......... .......... ..........
T996                 .......... .......... .......... .......... .......... .......... .......... ..........
T9102                .......... .......... .......... .......... .......... .......... .......... ..........
K1                   .......... .......... .......... .......... .......... .......... .......... ..........
Dd2                  .......... .......... .......... .......... .......... .......... .......... ..........
D10                  .......... .......... .......... .......... .......... .......... .......... ..........
FCC2                 .......... .......... .......... .......... .......... .......... .......... ..........
HB3                  .......... .......... .......... .......... .......... .......... .......... ..........
7G8                  .......... .......... .......... .......... .......... .......... .......... ..........
P.reichenowi         .......... .......... .......... .......... .......... .......... ...T...... ..........

                              410        420        430        440        450        460        470        480
                     ....|....| ....|....| ....|....| ....|....| ....|....| ....|....| ....|....| ....|....|
PFF0615c             TATATTGTTT TTGTGAAAAT AAAAAAACAA TAACTATTAA TGGTTCCAAT GGAAATCCTT CAAGTAAAAA AGATATAATA
3D7                  .......... .......... .......... .......... .......... .......... .......... ..........
RO33                 .......... .......... .......... .......... .......... .......... .......... ..........
Palo_Alto            .......... .......... .......... .......... .......... .......... .......... ..........
FCR3                 .......... .......... .......... .......... .......... .......... .......... ..........
Wellcome             .......... .......... .......... .......... .......... .......... .......... ..........
D6                   .......... .......... .......... .......... .......... .......... .......... ..........
T996                 .......... .......... .......... .......... .......... .......... .......... ..........
T9102                .......... .......... .......... .......... .......... .......... .......... ..........
K1                   .......... .......... .......... .......... .......... .......... .......... ..........
Dd2                  .......... .......... .......... .......... .......... .......... .......... ..........
D10                  .......... .......... .......... .......... .......... .......... .......... ..........
FCC2                 .......... .......... .......... .......... .......... .......... .......... ..........
HB3                  .......... .......... .......... .......... .......... .......... .......... ..........
7G8                  .......... .......... .......... .......... .......... .......... .......... ..........
P.reichenowi         .......... .......... .......... .......... ......A... ......T... ......CC.. G..A......

                              490        500        510        520        530        540        550        560
                     ....|....| ....|....| ....|....| ....|....| ....|....| ....|....| ....|....| ....|....|
PFF0615c             AATAGAGGAA TAGTTGAAAT TATTATACCT TCATTAAATG AAAAAGTTAA AGGATGTGAT TTTACAACAA GCGAATCTAC
3D7                  .......... .......... .......... .......... .......... .......... .......... ..........
RO33                 .......... .......... .......... .......... .......... .......... .......... ..........
Palo_Alto            .......... .......... .......... .......... .......... .......... .......... ..........
FCR3                 .......... .......... .......... .......... .......... .......... .......... ..........
Wellcome             .......... .......... .......... .......... .......... .......... .......... ..........
D6                   .......... .......... .......... .......... .......... .......... .......... ..........
T996                 .......... .......... .......... .......... .......... .......... .......... ..........
T9102                .......... .......... .......... .......... .......... .......... .......... ..........
K1                   .......... .......... .......... .......... .......... .......... .......... ..........
Dd2                  .......... .......... .......... .......... .......... .......... .......... ..........
D10                  .......... .......... .......... .......... .......... .......... .......... ..........
FCC2                 .......... .......... .......... .......... .......... .......... .......... ..........
HB3                  .......... .......... .......... .......... .......... .......... .......... ..........
7G8                  .......... .......... .......... .......... .......... .......... .......... ..........
P.reichenowi         .......... .......... .......... .......... .......... .......... .......... ..........

                              570        580        590        600        610        620        630        640
                     ....|....| ....|....| ....|....| ....|....| ....|....| ....|....| ....|....| ....|....|
PFF0615c             AATTTTCTCA AAAGGATATA GTATTAATGA AATATCTAAT AAATCATCAA ATAACCAACA AGATATTGTA TGTACAGTTA
3D7                  .......... .......... .......... .......... .......... .......... .......... ..........
RO33                 .......... .......... .......... .......... .......... .......... .......... ..........
Palo_Alto            .......... .......... .......... .......... .......... .......... .......... ..........
FCR3                 .......... .......... .......... .......... .......... .......... .......... ..........
Wellcome             .......... .......... .......... .......... .......... .......... .......... ..........
D6                   .......... .......... .......... .......... .......... .......... .......... ..........
T996                 .......... .......... .......... .......... .......... .......... .......... ..........
T9102                .......... .......... .......... .......... .......... .......... .......... ..........
K1                   .......... .......... .......... .......... .......... .......... .......... ..........
Dd2                  .......... .......... .......... .......... .......... .......... .......... ..........
D10                  .......... .......... .......... .......... .......... .......... .......... ..........
FCC2                 .......... .......... .......... .......... .......... .......... .......... ..........
HB3                  .......... .......... .......... .......... .......... .......... .......... ..........
7G8                  .......... .......... .......... .......... .......... .......... .......... ..........
P.reichenowi         C......... ..C....... A......... T......... .......... .......... .......... .....G....
```

```
                                 650        660        670        680        690        700        710        720
                           ....|....| ....|....| ....|....| ....|....| ....|....| ....|....| ....|....| ....|....|
PFF0615c                   AGGCACATGC TAATGATTTA ATCGGATTTA AATGTCCAAG CAATTATTCT GTTGAACCAC ATGATTGTTT TGTTAGTGCA
3D7                        .......... .......... .......... .......... .......... .......... .......... ..........
RO33                       .......... .......... .......... .......... .......... .......... .......... ..........
Palo_Alto                  .......... .......... .......... .......... .......... .......... .......... ..........
FCR3                       .......... .......... .......... .......... .......... .......... .......... ..........
Wellcome                   .......... .......... .......... .......... .......... .......... .......... ..........
D6                         .......... .......... .......... .......... .......... .......... .......... ..........
T996                       .......... .......... .......... .......... .......... .......... .......... ..........
T9102                      .......... .......... .......... .......... .......... .......... .......... ..........
K1                         .......... .......... .......... .......... .......... .......... .......... ..........
Dd2                        .......... .......... .......... .......... .......... .......... .......... ..........
D10                        .......... .......... .......... .......... .......... .......... .......... ..........
FCC2                       .......... .......... .......... .......... .......... .......... .......... ..........
HB3                        .......... .......... .......... .......... .......... .......... .......... ..........
7G8                        .......... .......... .......... .......... .......... .......... .......... ..........
P.reichenowi               ......G... .......... .......... ....C..... .......... .......... .......... ..........

                                 730        740        750        760        770        780        790        800
                           ....|....| ....|....| ....|....| ....|....| ....|....| ....|....| ....|....| ....|....|
PFF0615c                   TTCAATTTAA GTGGGAAAAA TGAAAACCTA GAAAATAAAC TTAAATTAAC AAATATAATT ATGGATCATT ATAATAATAC
3D7                        .......... .......... .......... .......... .......... .......... .......... ..........
RO33                       .......... .......... .......... .......... .......... .......... .......... ..........
Palo_Alto                  .......... .......... .......... .......... .......... .......... .......... ..........
FCR3                       .......... .......... .......... .......... .......... .......... .......... ..........
Wellcome                   .......... .......... .......... .......... .......... .......... .......... ..........
D6                         .......... .......... .......... .......... .......... .......... .......... ..........
T996                       .......... .......... .......... .......... .......... .......... .......... ..........
T9102                      .......... .......... .......... .......... .......... .......... .......... ..........
K1                         .......... .......... .......... .......... ......C... .......... .......... ..........
Dd2                        .......... .......... .......... .......... ......C... .......... .......... ..........
D10                        .......... .......... .......... .......... .......... .......... .......... ..........
FCC2                       .......... .......... .......... .......... .......... .......... .......... ..........
HB3                        .......... .......... .......... .......... .......... .......... .......... ..........
7G8                        .......... .......... .......... .......... .......... .......... .......... ..........
P.reichenowi               .......... .......... .......... .......... .......... .......... .......... ..........

                                 810        820        830        840        850        860        870        880
                           ....|....| ....|....| ....|....| ....|....| ....|....| ....|....| ....|....| ....|....|
PFF0615c                   TTTCTATTCA AGATTACCAA GTTTAATTTC TGATAATTGG AAATTCTTTT GTGTATGTTC AAAAGATAAT GAAAAAAAAT
3D7                        .......... .......... .......... .......... .......... .......... .......... ..........
RO33                       .......... .......... .......... .......... .......... .......... .......... ..........
Palo_Alto                  .......... .......... .......... .......... .......... .......... .......... ..........
FCR3                       .......... .......... .......... .......... .......... .......... .......... ..........
Wellcome                   .......... .......... .......... .......... .......... .......... .......... ..........
D6                         .......... .......... .......... .......... .......... .......... .......... ..........
T996                       .......... .......... .......... .......... .......... .......... .......... ..........
T9102                      .......... .......... .......... .......... .......... .......... .......... ..........
K1                         .......... .......... .......... .......... .......... .......... .......... ..........
Dd2                        .......... .......... .......... .......... .......... .......... .......... ..........
D10                        .......... .......... .......... .......... .......... .......... .......... ..........
FCC2                       .......... .......... .......... .......... .......... .......... .......... ..........
HB3                        .......... .......... .......... .......... .......... .......... .......... ..........
7G8                        .......... .......... .......... .......... .......... .......... .......... ..........
P.reichenowi               .......... .......... .......... ........C. .......... .......... .......... ..........

                                 890        900        910        920        930        940        950        960
                           ....|....| ....|....| ....|....| ....|....| ....|....| ....|....| ....|....| ....|....|
PFF0615c                   TAGTCTTTAC CGTAGAAGCA AGCATTTCAT CAAGTAATAC TAAACTTGCT TCAAGAGATA ATACATACCA AGATTATATA
3D7                        .......... .......... .......... .......... .......... .......... .......... ..........
RO33                       .......... .......... .......... .......... .......... .......... .......... ..........
Palo_Alto                  .......... .......... .......... .......... .......... .......... .......... ..........
FCR3                       .......... .......... .......... .......... .......... ......T... .......... ..........
Wellcome                   .......... .......... .......... .......... .......... ......T... .......... ..........
D6                         .......... .......... .......... .......... .......... .......... .......... ..........
T996                       .......... .......... .......... .......... .......... .......... .......... ..........
T9102                      .......... .......... .......... .......... .......... .......... .......... ..........
K1                         .......... .......... .......... .......... .......... .......... .......... ..........
Dd2                        .......... .......... .......... .......... .......... .......... .......... ..........
D10                        .......... .......... .......... .......... .......... .......... .......... ..........
FCC2                       .......... .......... .......... .......... .......... ......T... .......... ..........
HB3                        .......... .......... .......... .......... .......... .......... .......... ..........
7G8                        .......... .......... .......... .......... .......... .......... .......... ..........
P.reichenowi               .......... .......A.. .......... .......... .......... ........C. ........A. ..........
```

```
                            970        980        990       1000       1010       1020       1030       1040
                     ....|....| ....|....| ....|....| ....|....| ....|....| ....|....| ....|....| ....|....| ....
PFF0615c             TCCAACTCTT CTTTCTTAAC CCTTTCATCA TATTGTGCCT TCATCACTTT TATTATCACA TCATTCTTAT CATTCATCTT ATAA
3D7                  .......... .......... .......... .......... .......... ..........
RO33                 .......... .......... .......... .......... .......... ..........
Palo_Alto            .......... .......... .......... .......... .......... ..........
FCR3                 .......... .......... .......... .......... .......... ..........
Wellcome             .......... .......... .......... .......... .......... ..........
D6                   .......... .......... .......... .......... .......... ..........
T996                 .......... .......... .......... .......... .......... ..........
T9102                .......... .......... .......... .......... .......... ..........
K1                   .......... .......... .......... .......... .......... ..........
Dd2                  .......... .......... .......... .......... .......... ..........
D10                  .......... .......... .......... .......... .......... ..........
FCC2                 .......... .......... .......... .......... .......... ..........
HB3                  .......... .......... .......... .......... .......... ..........
7G8                  .......... .......... .......... .......... .......... ..........
P.reichenowi         .......... .......... .T........ ....A....C .......... .......... .......... .......... ....
```

PF13_0338

```
                             10         20         30         40         50         60         70         80
                     ....|....| ....|....| ....|....| ....|....| ....|....| ....|....| ....|....| ....|....|
PF13_0338            ATGTTTGCTG TAAACTTAAA AATTTGTATA TTTTTGTCAC TTGTTTCATT TTTGTTACAA TGTAAGAATA CACTTGCTAA
3D7                                   ..... .......... .......... .......... .......... .......... ..........
RO33                                  ..... .......... .......... .......... .......... .......... ..........
Palo Alto                             ..... .......... .......... .......... .......... .......... ..........
FCR3                                  ..... .......... .......... .......... .......... .......... ..........
Wellcome                              ..... .......... .......... .......... .......... .......... ..........
D6                                    ..... .......... .......... .......... .......... .......... ..........
T996                                  ..... .......... .......... .......... .......... .......... ..........
T9102                                 ..... .......... .......... .......... .......... .......... ..........
K1                                    ..... .......... .......... .......... .......... .......... ..........
Dd2                                   ..... .......... .......... .......... .......... .......... ..........
FCC2                                  ..... .......... .......... .......... .......... .......... ..........
HB3                                   ..... .......... .......... .......... .......... .......... ..........
7G8                                   ..... .......... .......... .......... .......... .......... ..........
P.reichenowi         .......... .......... .......... ........G. .......... .......... .......... .C....G...

                             90        100        110        120        130        140        150        160
                     ....|....| ....|....| ....|....| ....|....| ....|....| ....|....| ....|....| ....|....|
PF13_0338            TGTAACTTTT GAACAAAAGG TACAGACAAA TTTAAGTCAT GATAAAAATG GAGATATAGT ATATGGACAC AGAGAATTTA
3D7                  .......... .......... .......... .......... .......... .......... .......... ..........
RO33                 .......... .......... .......... .......... .......... .......... .......... ..........
Palo Alto            .......... .......... .......... .......... .......... .......... .......... ..........
FCR3                 .......... .......... .......... .......... .......... .......... .......... ..........
Wellcome             .......... .......... .......... .......... .......... .......... .......... ..........
D6                   .......... A......... .......... .......... .......... .......... .......... ..........
T996                 .......... .......... .......... .......... .......... .......... .......... ..........
T9102                .......... .......... .......... .......... .......... .......... .......... ..........
K1                   .......... .......... .......... .......... .......... .......... .......... ..........
Dd2                  .......... .......... .......... .......... .......... .......... .......... ..........
D10                  .......... .......... .......... .......... .......... .......... .......... ..........
FCC2                 .......... .......... .......... .......... .......... .......... .......... ..........
HB3                  .......... A......... .......... .......... .......... .......... .......... ..........
7G8                  .......... .......... .......... .......... .......... .......... .......... ..........
P.reichenowi         .......... ........A. ..G....... A......... A....TG... .......... T......... ..........

                            170        180        190        200        210        220        230        240
                     ....|....| ....|....| ....|....| ....|....| ....|....| ....|....| ....|....| ....|....|
PF13_0338            AA-------- ---------- ---------- ---------- ---------- ---------- --------GG AGGTATTTAT
3D7                  ..-------- ---------- ---------- ---------- ---------- ---------- --------.. ..........
RO33                 ..GGAGGTAT AGTATATGGA CACAGAGAAT TTAAA----- ---------- ---------- --------.. ..........
Palo Alto            ..GGAGGTAT AGTATATGGA CACAGAGAAT TTAAA----- ---------- ---------- --------.. ..........
FCR3                 ..GGAGGTAT AGTATATGGA CACAGAGAAT TTAAAGGAGG TATAGTATAT GGACACAGAG AATTTAAA.. ..........
Wellcome             ..GGAGGTAT AGTATATGGA CACAGAGAAT TTAAAGGAGG TATAGTATAT GGACACAGAG AATTTAAA.. ..........
D6                   ..-------- ---------- ---------- ---------- ---------- ---------- --------.. ..........
T996                 ..GGAGGTAT AGTATATGGA CACAGAGAAT TTAAA----- ---------- ---------- --------.. ..........
T9102                ..-------- ---------- ---------- ---------- ---------- ---------- --------.. ..........
K1                   ..GGAGGTAT AGTATATGGA CACAGAGAAT TTAAA----- ---------- ---------- --------.. ..........
Dd2                  ..-------- ---------- ---------- ---------- ---------- ---------- --------.. ..........
D10                  ..GGAGGTAT AGTATATGGA CACAGAGAAT TTAAA----- ---------- ---------- --------.. ..........
FCC2                 ..-------- ---------- ---------- ---------- ---------- ---------- --------.. ..........
HB3                  ..-------- ---------- ---------- ---------- ---------- ---------- --------.. ..........
7G8                  ..-------- ---------- ---------- ---------- ---------- ---------- --------.. ..........
P.reichenowi         ..-------- ---------- ---------- ---------- ---------- ---------- --------.. ....G.....
```

```
                           250        260        270        280        290        300        310        320
                   ....|....| ....|....| ....|....| ....|....| ....|....| ....|....| ....|....| ....|....|
PF13_0338          GCATTTTTAG GATATGACAA CTGTACAATA GAAGTTAATA AAACAGTGAA TGGAATAGAT TGGAATGAAA AAAAGGACGT
3D7                .......... .......... .......... .......... .......... .......... .......... ..........
RO33               .......... .......... .......... .......... .......... .......... .......... ..........
Palo Alto          .......... .......... .......... .......... .......... .......... .......... ..........
FCR3               .......... .......... .......... .......... .......... .......... .......... ..........
Wellcome           .......... .......... .......... .......... .......... .......... .......... ..........
D6                 .......... .......... .......... .......... .......... .......... .......... ..........
T996               .......... .......... A......... .......... .......... .......... .......... ..........
T9102              .......... .......... .......... A......... .......... .......... .......... ..........
K1                 .......... .......... .......... .......... .......... .......... .......... ..........
Dd2                .......... .......... .......... A......... .......... .......... .......... ..........
D10                .......... .......... .......... .......... .......... .......... .......... ..........
FCC2               .......... .......... .......... A......... .......... .......... .......... ..........
HB3                .......... .......... .......... .......... .......... .......... .......... ..........
7G8                .......... .......... .......... A......... .......... .......... .......... ..........
P.reichenowi       .......... ....C..... G....G.... ......T... .......... .......... .......... .......G..

                           330        340        350        360        370        380        390        400
                   ....|....| ....|....| ....|....| ....|....| ....|....| ....|....| ....|....| ....|....|
PF13_0338          AAAAGTAAGT GGTAATAATA ATATTGCTGT AGTATATTCC ATTTTTACAA GTGAAGAGAA GATGATATTA ATTTTTAAAT
3D7                .......... .......... .......... .......... .......... .......... .......... ..........
RO33               .......... .......... .......... .......... .......... .......... .......... ..........
Palo Alto          .......... .......... .......... .......... .......... .......... .......... ..........
FCR3               .......... .......... .......... .......... .......... .......... .......... ..........
Wellcome           .......... .......... .......... .......... .......... .......... .......... ..........
D6                 .......... .......... .......... .......... .......... .......... .......... ..........
T996               .......... .......... .......... .......... .......... .......... .......... ..........
T9102              .......... .......... .......... .......... .......... .......... .......... ..........
K1                 .......... .......... .......... .......... .......... .......... .......... ..........
Dd2                .......... .......... .......... .......... .......... .......... .......... ..........
D10                .......... .......... .......... .......... .......... .......... .......... ..........
FCC2               .......... .......... .......... .......... .......... .......... .......... ..........
HB3                .......... .......... .......... .......... .......... .......... .......... ..........
7G8                .......... .......... .......... .......... .......... .......... .......... ..........
P.reichenowi       .......... .......... ........A. .......... .......... .......... .......... ..........

                           410        420        430        440        450        460        470        480
                   ....|....| ....|....| ....|....| ....|....| ....|....| ....|....| ....|....| ....|....|
PF13_0338          GTGATAATAA ATTTTATATT ACTAAATATG GAAAAGAATT TAAATGGTCA GATCCTAAAG TTATAGATGT ATCTAATGTT
3D7                .......... .......... .......... .......... .......... .......... .......... ..........
RO33               .......... .......... .......... .......... .......... .......... .......... ..........
Palo Alto          .......... .......... .......... .......... .......... .......... .......... ..........
FCR3               .......... .......... .......... .......... .......... .......... .......... ..........
Wellcome           .......... .......... .......... .......... .......... .......... .......... ..........
D6                 .......... .......... .......... .......... .......... .......... .......... ..........
T996               .......... .......... .......... .......... .......... .......... .......... ..........
T9102              .......... .......... .......... .......... .......... .......... .......... ..........
K1                 .......... .......... .......... .......... .......... .......... .......... ..........
Dd2                .......... .......... .......... .......... .......... .......... .......... ..........
D10                .......... .......... .......... .......... .......... .......... .......... ..........
FCC2               .......... .......... .......... .......... .......... .......... .......... ..........
HB3                .......... .......... .......... .......... .......... .......... .......... ..........
7G8                .......... .......... .......... .......... .......... .......... .......... ..........
P.reichenowi       .......... .......... .......... .......... .G........ .....A...C .......... ..........

                           490        500        510        520        530        540        550        560
                   ....|....| ....|....| ....|....| ....|....| ....|....| ....|....| ....|....| ....|....|
PF13_0338          ATTGGTACAA ATACTACCCC AGCTGTTTAC TCTGGTTCTT TACTATCTAT GAATAATGAT TTTGAAAAAT ATATTTTGGT
3D7                .......... .......... .......... .......... .......... .......... .......... ..........
RO33               .......... .......... .......... .......... .......... .......... .......... ..........
Palo Alto          .......... .......... .......... .......... .......... .......... .......... ..........
FCR3               .......... .......... .......... .......... .......... .......... .......... ..........
Wellcome           .......... .......... .......... .......... .......... .......... .......... ..........
D6                 .......... .......... .......... .......... .......... .......... .......... ..........
T996               .......... .......... .......... .......... .......... .......... .......... ..........
T9102              .......... .......... .......... .......... .......... .......... .......... ..........
K1                 .......... .......... .......... .......... .......... .......... .......... ..........
Dd2                .......... .......... .......... .......... .......... .......... .......... ..........
D10                .......... .......... .......... .......... .......... .......... .......... ..........
FCC2               .......... .......... .......... .......... .......... .......... .......... ..........
HB3                .......... .......... .......... .......... .......... .......... .......... ..........
7G8                .......... .......... .......... .......... .......... .......... .......... ..........
P.reichenowi       .......... ....A.T... ....A..... .......... .......... .......... .......... .C........
```

```
                        570        580        590        600        610        620        630        640
               ....|....| ....|....| ....|....| ....|....| ....|....| ....|....| ....|....| ....|....|
PF13_0338      TTGTGAAAAT CATAGTCAGA ATTATATAAA TGTAGTAGAC CAGGAATATA TGAGAGAAAT ACGTTTATTA GGTAAATGTA
3D7            .......... .......... .......... .......... .......... .......... .......... ..........
RO33           .......... .......... .......... .......... .......... .......... .......... ..........
Palo Alto      .......... .......... .......... .......... .......... .......... .......... ..........
FCR3           .......... .......... .......... .......... .......... .......... .......... ..........
Wellcome       .......... .......... .......... .......... .......... .......... .......... ..........
D6             .......... .......... .......... .......... .......... .......... .......... ..........
T996           .......... .......... .......... .......... .......... .......... .......... ..........
T9102          .......... .......... .......... .......... .......... .......... .......... ..........
K1             .......... .......... .......... .......... .......... .......... .......... ..........
Dd2            .......... .......... .......... .......... .......... .......... .......... ..........
D10            .......... .......... .......... .......... .......... .......... .......... ..........
FCC2           .......... .......... .......... .......... .......... .......... .......... ..........
HB3            .......... .......... .......... .......... .......... .......... .......... ..........
7G8            .......... .......... .......... .......... .......... .......... .......... ..........
P.reichenowi   .......... .......... .......... .....G...G ..A....... .......... .......... ..........

                        650        660        670        680        690        700        710        720
               ....|....| ....|....| ....|....| ....|....| ....|....| ....|....| ....|....| ....|....|
PF13_0338      TGTTATCTTT TGATGAAGGA AATAACTGGA AGAATGAAGT AATGAATTTA TATAGTGATG AAGGTTATAC AAAAATTAAT
3D7            .......... .......... .......... .......... .......... .......... .......... ..........
RO33           .......... .......... .......... .......... .......... .......... .......... ..........
Palo Alto      .......... .......... .......... .......... .......... .......... .......... ..........
FCR3           .......... .......... .......... .......... .......... .......... .......... ..........
Wellcome       .......... .......... .......... .......... .......... .......... .......... ..........
D6             .......... .......... .......... .......... .......... .......... .......... ..........
T996           .......... .......... .......... .......... .......... .......... .......... ..........
T9102          .......... .......... .......... .......... .......... .......... .......... ..........
K1             .......... .......... .......... .......... .......... .......... .......... ..........
Dd2            .......... .......... .......... .......... .......... .......... .......... ..........
D10            .......... .......... .......... .......... .......... .......... .......... ..........
FCC2           .......... .......... .......... .......... .......... .......... .......... ..........
HB3            .......... .......... .......... .......... .......... .......... .......... ..........
7G8            .......... .......... .......... .......... .......... .......... .......... ..........
P.reichenowi   .......... C......... ..A....... .......... G...G..... .......... .......... ..........

                        730        740        750        760        770        780        790        800
               ....|....| ....|....| ....|....| ....|....| ....|....| ....|....| ....|....| ....|....|
PF13_0338      ACTTTAAGAT TATCAGATTA CGGAGGAAAA ATACTTGTGA AAGGAACTAA TGCACAAAAT CTTAACCAAA CGATACGATC
3D7            .......... .......... .......... .......... .......... .......... .......... ..........
RO33           .......... .......... .......... .......... .......... .......... .......... ..........
Palo Alto      .......... .......... .......... .......... .......... .......... .......... ..........
FCR3           .......... .......... .......... .......... .......... .......... .......... ..........
Wellcome       .......... .......... .......... .......... .......... .......... .......... ..........
D6             .......... .......... .......... .......... .......... .......... .......... ..........
T996           .......... .......... .......... .......... .......... .......... .......... ..........
T9102          .......... .......... .......... .......... .......... .......... .......... ..........
K1             .......... .......... .......... .......... .......... .......... .......... ..........
Dd2            .......... .......... .......... .......... .......... .......... .......... ..........
D10            .......... .......... .......... .......... .......... .......... .......... ..........
FCC2           .......... .......... .......... .......... .......... .......... .......... ..........
HB3            .......... .......... .......... .......... .......... .......... .......... ..........
7G8            .......... .......... .......... .......... .......... .......... .......... ..........
P.reichenowi   .......... .......... T......... .......... .......... .......... .......... ..........

                    810        820        830        840        850        860        870        880
               ....|....| ....|....| ....|....| ....|....| ....|....| ....|....| ....|....| ....|....|
PF13_0338      CATAATTCTT TTATGTAGTA ATTTGCATGA TTGGAAATTA TTTTGTGGAC TTCCTACCAT AAGATTCAGG AAGGATATTT
3D7            .......... .......... .......... .......... .......... .......... .......... ..........
RO33           .......... .......... .......... .......... .......... .......... .......... ..........
Palo Alto      .......... .......... .......... .......... .......... .......... .......... ..........
FCR3           .......... .......... .......... .......... .......... .......... .......... ..........
Wellcome       .......... .......... .......... .......... .......... .......... .......... ..........
D6             .......... .......... .......... .......... .......... .......... .......... ..........
T996           .......... .......... .......... .......... .......... .......... .......... ..........
T9102          .......... .......... .......... .......... .......... .......... .......... ..........
K1             .......... .......... .......... .......... .......... .......... .......... ..........
Dd2            .......... .......... .......... .......... .......... .......... .......... ..........
D10            .......... .......... .......... .......... .......... .......... .......... ..........
FCC2           .......... .......... .......... .......... .......... .......... .......... ..........
HB3            .......... .......... .......... .......... .......... .......... .......... ..........
7G8            .......... .......... .......... .......... .......... .......... .......... ..........
P.reichenowi   A.....A... .......A.. .......... .......... .......C.. .......... .......... ..........
```

```
                              890        900        910        920        930        940        950        960
                        ....|....| ....|....| ....|....| ....|....| ....|....| ....|....| ....|....| ....|....|
PF13_0338               CAGTAGAAAA TCTAACATAT TTAAATACTT ATCATCTAGC CATTGTAAAA AATGAAGATA AATTACAATT AGCTTTTACA
3D7                     .......... .......... .......... .......... .......... .......... .......... ..........
RO33                    .......... .......... .......... .......... .......... .......... .......... ..........
Palo Alto               .......... .......... .......... .......... .......... .......... .......... ..........
FCR3                    .......... .......... .......... .......... .......... .......... .......... ..........
Wellcome                .......... .......... .......... .......... .......... .......... .......... ..........
D6                      .......... .......... .......... .......... .......... .......... .......... ..........
T996                    .......... .......... .......... .......... ....A..... .......... .......... ..........
T9102                   .......... .......... .......... .......... .......... .......... .......... ..........
K1                      .......... .......... .......... .......... .......... .......... .......... ..........
Dd2                     .......... .......... .......... .......... .......... .......... .......... ..........
D10                     .......... .......... .......... .......... .......... .......... .......... ..........
FCC2                    .......... .......... .......... .......... .......... .......... .......... ..........
HB3                     .......... .......... .......... .......... .......... .......... .......... ..........
7G8                     .......... .......... .......... .......... .......... .......... .......... ..........
P.reichenowi            .......... .......... ..T....... .......... .......... .......... .......... ..........

                              970        980        990       1000       1010       1020       1030       1040
                        ....|....| ....|....| ....|....| ....|....| ....|....| ....|....| ....|....| ....|....|
PF13_0338               TATGATTTAT TTGAAACATT TGATCCGCAA TATTTGAACA CTGAATTTAA TGGTGTTTCT CATTATTTTG TTTTAGCTCC
3D7                     .......... .......... .......... .......... .......... .......... .......... ..........
RO33                    .......... .......... .......... .......... .......... .......... .......... ..........
Palo Alto               .......... .......... .......... .......... .......... .......... .......... ..........
FCR3                    .......... .......... .......... .......... .......... .......... .......... ..........
Wellcome                .......... .......... .......... .......... .......... .......... .......... ..........
D6                      .......... .......... .......... .......... .......... .......... .......... ..........
T996                    .......... .......... .......... .......... .......... .......... .......... ..........
T9102                   .......... .......... .......... .......... .......... .......... .......... ..........
K1                      .......... .......... .......... .......... .......... .......... .......... ..........
Dd2                     .......... .......... .......... .......... .......... .......... .......... ..........
D10                     .......... .......... .......... .......... .......... .......... .......... ..........
FCC2                    .......... .......... .......... .......... .......... .......... .......... ..........
HB3                     .......... .......... .......... .......... .......... .......... .......... ..........
7G8                     .......... .......... .......... .......... .......... .......... .......... ..........
P.reichenowi            .......... .......... ......A... .......... T........T GT........ ........C. .........A

                             1050       1060       1070       1080       1090       1100       1110       1120
                        ....|....| ....|....| ....|....| ....|....| ....|....| ....|....| ....|....| ....|....|
PF13_0338               TGATGAAATG GTATATTTAT TTTATCATGG AAATGAGAAA AAAAATTATG TCATAAAAAT AAAAACAGTA CCAAGAAAAA
3D7                     .......... .......... .......... .......... .......... .......... .......... ..........
RO33                    .......... .......... .......... .......... .......... .......... .......... ..........
Palo Alto               .......... .......... .......... .......... .......... .......... .......... ..........
FCR3                    .......... .......... .......... .......... .......... .......... .......... ..........
Wellcome                .......... .......... .......... .......... .......... .......... .......... ..........
D6                      .......... .......... .......... .......... .......... .......... .......... ..........
T996                    .......... .......... .......... .......... .......... .......... .......... ..........
T9102                   .......... .......... .......... .......... .......... .......... .......... ..........
K1                      .......... .......... .......... .......... .......... .......... .......... ..........
Dd2                     .......... .......... .......... .......... .......... .......... .......... ..........
D10                     .......... .......... .......... .......... .......... .......... .......... ..........
FCC2                    .......... .......... .......... .......... .......... .......... .......... ..........
HB3                     .......... .......... .......... .......... .......... .......... .......... ..........
7G8                     .......... .......... .......... .......... .......... .......... .......... ..........
P.reichenowi            .......... .......... ...G.....A ........G. .......... .......... .......A.. ..........

                             1130       1140       1150       1160       1170       1180       1190       1200
                        ....|....| ....|....| ....|....| ....|....| ....|....| ....|....| ....|....| ....|....|
PF13_0338               TAGGTTGTGA ATTAAATACA AATGATACTG TAAATAAAAT TTATACATAC ACGTATAAAT ATATATATAA TAATAAGCTA
3D7                     .......... .......... .......... .......... .......... .......... .......... ..........
RO33                    .......... .......... .......... ......C... .......... .......... .......... ..........
Palo Alto               .......... .......... .......... .......... .......... .......... .......... ..........
FCR3                    .......... .......... .......... ......C... .......... .......... .......... ..........
Wellcome                .......... .......... .......... ......C... .......... .......... .......... ..........
D6                      .......... .......... .......... ......C... .......... .......... .......... ..........
T996                    .......... .......... .......... ......C... .......... .......... .......... ..........
T9102                   .......... .......... .......... ......C... .......... .......... .......... ..........
K1                      .......... .......... .......... ......C... .......... .......... .......... ..........
Dd2                     .......... .......... .......... ......C... .......... .......... .......... ..........
D10                     .......... .......... .......... ......C... .......... .......... .......... ..........
FCC2                    .......... .......... .......... ......C... .......... .......... .......... ..........
HB3                     .......... .......... .......... ......C... .......... .......... .......... ..........
7G8                     .......... .......... .......... ......C... .......... .......... .......... ..........
P.reichenowi            ....A..... .......... .......... .......... .......... ..T....... .C........ ......CT..
```

```
                         1210       1220       1230       1240       1250       1260       1270       1280
                ....|....| ....|....| ....|....| ....|....| ....|....| ....|....| ....|....| ....|....|
PF13_0338       AGTGCGAAAA CATGTAAAGT TCCATCATCA CATTTAAAAT ATTCGAGTGA TGGTCTATAT AAATTATTTG AGGTAAGATT
3D7             .......... .......... .......... .......... .......... .......... .......... ..........
RO33            .......... .......... .......... .......... .......... .......... .......... ..........
Palo Alto       .......... .......... .......... .......... .......... .......... .......... ..........
FCR3            ...C...... .......... .......... .......... .......... .......... .......... ..........
Wellcome        ...C...... .......... .......... .......... .......... .......... .......... ..........
D6              .......... .......... .......... .......... .......... .......... .......... ..........
T996            .......... .......... .......... .......... .......... .......... .......... ..........
T9102           ...C...... .......... .......... .......... .......... .......... .......... ..........
K1              ...C...... .......... .......... .......... .......... .......... .......... ..........
Dd2             ...C...... .......... .......... .......... .......... .......... .......... ..........
D10             ...C...... .......... .......... .......... .......... .......... .......... ..........
FCC2            .......... .......... .......... .......... .......... .......... .......... ..........
HB3             .......... .......... .......... .......... .......... .......... .......... ..........
7G8             .......... .......... .......... .......... .......... .......... .......... ..........
P.reichenowi    ...C...... .......... .......... .......... .......... .......... .......... ..........

                         1290       1300       1310       1320       1330       1340       1350       1360
                ....|....| ....|....| ....|....| ....|....| ....|....| ....|....| ....|....| ....|....|
PF13_0338       ACCAAAAGAT ATAAAAGTAA CTGAAAATTG TTTTAGATAT TCTTTCTTGA GTGATTTAAA TAATAAATAT CATACAACTA
3D7             .......... .......... .......... .......... .......... .......... .......... ..........
RO33            .......... .......... .......... .......... .......... .......... .......... ..........
Palo Alto       .......... .......... .......... .......... .......... .......... .......... ..........
FCR3            .......... .......... .......... .......... .......... .......... .......... ..........
Wellcome        .......... .......... .......... .......... .......... .......... .......... ..........
D6              .......... .......... .......... .......... .......... .......... .......... ..........
T996            .......... .......... .......... .......... .......... .......... ...A...... ..........
T9102           .......... .......... .......... .......... .......... .......... .......... ..........
K1              .......... .......... .......... .......... .......... .......... .......... ..........
Dd2             .......... .......... .......... .......... .......... .......... .......... ..........
D10             .......... .......... .......... .......... .......... .......... .......... ..........
FCC2            .......... .......... .......... .......... .......... .......... .......... ..........
HB3             .......... .......... .......... .......... .......... .......... ...A...... ..........
7G8             .......... .......... .......... .......... .......... .......... .......... ..........
P.reichenowi    .......... .......... .......... .......... .....T.... ....A..... .......... .....T....

                         1370       1380       1390       1400       1410       1420       1430       1440
                ....|....| ....|....| ....|....| ....|....| ....|....| ....|....| ....|....| ....|....|
PF13_0338       TCATAAAAAC ACGAGTTATT AACAAATTAG AAGATTATGT AGAAGTACAA TTTCACTTTC CGATTTATTA TACTAAATTT
3D7             .......... .......... .......... .......... .......... .......... .......... ..........
RO33            .......... .......... .......... .......... .......... .......... .......... ..........
Palo Alto       .......... .......... .......... .......... .......... .......... .......... ..........
FCR3            .......... .......... .......... .......... .......... .......... .......... ..........
Wellcome        .......... .......... .......... .......... .......... .......... .......... ..........
D6              .......... .......... .......... .......... .......... .......... .......... ..........
T996            .......... .......... .......... .......... .......... .......... .......... ..........
T9102           .......... .......... .......... .......... .......... .......... .......... ..........
K1              .......... .......... .......... .......... .......... .......... .......... ..........
Dd2             .......... .......... .......... .......... .......... .......... .......... ..........
D10             .......... .......... .......... .......... .......... .......... .......... ..........
FCC2            .......... .......... .......... .......... .......... .......... .......... ..........
HB3             .......... .......... .......... .......... .......... .......... .......... ..........
7G8             .......... .......... .......... .......... .......... .......... .......... ..........
P.reichenowi    .A........ .A........ .......... .......... .......... .......... .......... ..........

                         1450       1460       1470       1480       1490       1500       1510       1520
                ....|....| ....|....| ....|....| ....|....| ....|....| ....|....| ....|....| ....|....|
PF13_0338       TTATACAATT ATAAAAGTAC GTATTGTGTC TTGAGTAATA ACTATAGAAT AGTTGTTGAA TTTGATTATA TACGTAATCA
3D7             .......... .......... .......... .......... .......... .......... .......... ..........
RO33            .......... .......... .......... .......... .......... .......... .......... ..........
Palo Alto       .......... .......... .......... .......... .......... .......... .......... ..........
FCR3            .......... .......... .......... .......... .......... .......... .......... ..........
Wellcome        .......... .......... .......... .......... .......... .......... .......... ..........
D6              .......... .......... .......... .......... .......... .......... .......... ..........
T996            .......... .......... .......... .......... .......... .......... .......... ..........
T9102           .......... .......... .......... .......... .......... .......... .......... ..........
K1              .......... .......... .......... .......... .......... .......... .......... ..........
Dd2             .......... .......... .......... .......... .......... .......... .......... ..........
D10             .......... .......... .......... .......... .......... .......... .......... ..........
FCC2            .......... .......... .......... .......... .......... .......... .......... ..........
HB3             .......... .......... .......... .......... .......... .......... .......... ..........
7G8             .......... .......... .......... .......... .......... .......... .......... ..........
P.reichenowi    .......... .......... A......... .......... .......... .......... .......... ..........
```

```
                                 1530       1540       1550       1560       1570       1580       1590       1600
                         ....|....| ....|....| ....|....| ....|....| ....|....| ....|....| ....|....| ....|....|
PF13_0338                TATTGATCTA GATTTTCCGT TTGATACAGA TACTGTAAAG TTATATAGTA ATGAAAGTGT TACACATGCA TTTAGAAATA
3D7                      .......... .......... .......... .......... .......... .......... .......... ..........
RO33                     .......... .......... .......... .......... .......... .......... .......... ..........
Palo Alto                .......... .......... .......... .......... .......... .......... .......... ..........
FCR3                     .......... .......... .......... .......... .......... .......... .......... ..........
Wellcome                 .......... .......... .......... .......... .......... .......... .......... ..........
D6                       .......... .......... .......... .......... .......... .......... .......... ..........
T996                     .......... .......... .......... .......... .......... .......... .......... ..........
T9102                    .......... .......... .......... .......... .......... .......... .......... ..........
K1                       .......... .......... .......... .......... .......... .......... .......... ..........
Dd2                      .......... .......... .......... .......... .......... .......... .......... ..........
D10                      .......... .......... .......... .......... .......... .......... .......... ..........
FCC2                     .......... .......... .......... .......... .......... .......... .......... ..........
HB3                      .......... .......... .......... .......... .......... .......... .......... ..........
7G8                      .......... .......... .......... .......... .......... .......... .......... ..........
P.reichenowi             .......... ........C. .......... .......... .......... ......T... .......... ..........

                                 1610       1620       1630       1640       1650       1660       1670       1680
                         ....|....| ....|....| ....|....| ....|....| ....|....| ....|....| ....|....| ....|....|
PF13_0338                ATACAGAAAA AACACATGTG CATAAATTTC CTAAAGGTAC ATATATGACA TCTTATTTTA GTTATGAAAA AGAATATGTT
3D7                      .......... .......... .......... .......... .......... .......... .......... ..........
RO33                     .......... .......... .......... .......... .......... .......... .......... ..........
Palo Alto                .......... .......... .......... .......... .......... .......... .......... ..........
FCR3                     .......... .......... .......... .......... .......... .......... .......... ..........
Wellcome                 .......... .......... .......... .......... .......... .......... .......... ..........
D6                       .......... .......... .......... .......... .......... .......... .......... ..........
T996                     .......... .......... .......... .......... .......... .......... .......... ..........
T9102                    .......... .......... .......... .......... .......... .......... .......... ..........
K1                       .......... .......... .......... .......... .......... .......... .......... ..........
Dd2                      .......... .......... .......... .......... .......... .......... .......... ..........
D10                      .......... .......... .......... .......... .......... .......... .......... ..........
FCC2                     .......... .......... .......... .......... .......... .......... .......... ..........
HB3                      .......... .......... .......... .......... .......... .......... .......... ..........
7G8                      .......... .......... .......... .......... .......... .......... .......... ..........
P.reichenowi             ........G. ......CAA. ...G....G. .C........ T......... .......... .......... ..........

                                 1690       1700       1710       1720       1730       1740       1750       1760
                         ....|....| ....|....| ....|....| ....|....| ....|....| ....|....| ....|....| ....|....|
PF13_0338                ATATCTAATT ATATAGAAGA ACCATTTTCT ACTACCTTTA CTATATTAAC CCAAACACAA ATGAATGTTC ATTTTATGGC
3D7                      .......... .......... .......... .......... .......... .......... .......... ..........
RO33                     .......... ........A. .......... .......... .......... .......... .......... ..........
Palo Alto                .......... .......... .......... .......... .......... .......... .......... ..........
FCR3                     .......... .......... .......... .......... .......... .......... .......... ..........
Wellcome                 .......... .......... .......... .......... .......... .......... .......... ..........
D6                       .......... .......... .......... .......... .......... .......... .......... ..........
T996                     .......... .......... .......... .......... .......... .......... .......... ..........
T9102                    .......... .......... .......... .......... .......... .......... .......... ..........
K1                       .......... .......... .......... .......... .......... .......... .......... ..........
Dd2                      .......... .......... .......... .......... .......... .......... .......... ..........
D10                      .......... .......... .......... .......... .......... .......... .......... ..........
FCC2                     .......... .......... .......... .......... .......... .......... .......... ..........
HB3                      .......... ........A. .......... .......... .......... .......... .......... ..........
7G8                      .......... ........A. .......... .......... .......... .......... .......... ..........
P.reichenowi             .......... .......... .A........ .....A.... .......... A.....A... .........A ..........

                                 1770       1780       1790       1800       1810       1820       1830       1840
                         ....|....| ....|....| ....|....| ....|....| ....|....| ....|....| ....|....| ....|....|
PF13_0338                TGGTGGACAA AAATATAAAT ATGAAGGTAT CGATTTAACA GATTCTTCAC CAAACTATGA ATTATCACTT AATTCTTTGT
3D7                      .......... .......... .......... .......... .......... .......... .......... ..........
RO33                     .......... .......... .......... .......... .......... .......... .......... ..........
Palo Alto                .......... .......... .......... .......... .......... .......... .......... ..........
FCR3                     .......... .......... .......... .......... .......... .......... .......... ..........
Wellcome                 .......... .......... .......... .......... .......... .......... .......... ..........
D6                       .......... .......... .......... .......... .......... .......... .......... ..........
T996                     .......... .......... .......... .......... .......... .......... .......... ..........
T9102                    .......... .......... .......... .......... .......... .......... .......... ..........
K1                       .......... .......... .......... .......... .......... .......... .......... ..........
Dd2                      .......... .......... .......... .......... .......... .......... .......... ..........
D10                      .......... .......... .......... .......... .......... .......... .......... ..........
FCC2                     .......... .......... .......... .......... .......... .......... .......... ..........
HB3                      .......... .......... .......... .......... .......... .......... .......... ..........
7G8                      .......... .......... .......... .......... .......... .......... .......... ..........
P.reichenowi             .......... .......... .......... .......... .......... .......... ...G...... .....C...G
```

```
                      1850       1860       1870       1880       1890       1900       1910       1920
                ....|....| ....|....| ....|....| ....|....| ....|....| ....|....| ....|....| ....|....|
PF13_0338       CAGATAGCCA AAATGTTGAT ATTTTTGTAT CCAAATTTGA TAATAATAAA ACGATAGGTT TTGTTTGTCC TGTTAAAAGT
3D7             .......... .......... .......... .......... .......... .......... .......... ..........
RO33            .......... .......... .......... .......... .......... .......... .......... ..........
Palo Alto       .......... .......... .......... .......... .......... .......... .......... ..........
FCR3            .......... .......... .......... .......... .......... .......... .......... ..........
Wellcome        .......... .......... .......... .......... .......... .......... .......... ..........
D6              .......... .......... .......... .......... .......... .......... .......... ..........
T996            .......... .......... .......... .......... .......... .......... .......... ..........
T9102           .......... .......... .......... .......... .......... .......... .......... ..........
K1              .......... .......... .......... .......... .......... .......... .......... ..........
Dd2             .......... .......... .......... .......... .......... .......... .......... ..........
D10             .......... .......... .......... .......... .......... .......... .......... ..........
FCC2            .......... .......... .......... .......... .......... .......... .......... ..........
HB3             .......... .......... .......... .......... .......... .......... .......... ..........
7G8             .......... .......... .......... .......... .......... .......... .......... ..........
P.reichenowi    .......... ....A..... .......... .......... .......... ..CC...... .......... ..........

                      1930       1940       1950       1960       1970       1980       1990       2000
                ....|....| ....|....| ....|....| ....|....| ....|....| ....|....| ....|....| ....|....|
PF13_0338       AGTTATGACG GATTAAATTG TTTTGACAAC GTTTATATAA AAAATAAGAC ACTTGTCAAA ATCGAATATT TATTTGGAGA
3D7             .......... .......... .......... .......... .......... .......... .......... ..........
RO33            .......... .......... .......... .......... .......... .......... .......... ..........
Palo Alto       .......... .......... .......... .......... .......... .......... .......... ..........
FCR3            .......... .......... .......... .......... .......... .......... .......... ..........
Wellcome        .......... .......... .......... .......... .......... .......... .......... ..........
D6              .......... .......... .......... .......... .......... .......... .......... ..........
T996            .......... .......... .......... .......... .......... .......... .......... ..........
T9102           .......... .......... .......... .......... .......... .......... .......... ..........
K1              .......... .......... .......... .......... .......... .......... .......... ..........
Dd2             .......... .......... .......... .......... .......... .......... .......... ..........
D10             .......... .......... .......... .......... .......... .......... .......... ..........
FCC2            .......... .......... .......... .......... .......... .......... .......... ..........
HB3             .......... .......... .......... .......... .......... .......... .......... ..........
7G8             .......... .......... .......... .......... .......... .......... .......... ..........
P.reichenowi    .......... .......... .........T .......... .......... .......... .......... ..........

                      2010       2020       2030       2040       2050       2060       2070       2080
                ....|....| ....|....| ....|....| ....|....| ....|....| ....|....| ....|....| ....|....|
PF13_0338       AAATGATATC TTTGTTGTTC CTCAAAGGAG AATATATAAA ACTGAAGGTA CAGCTATGGA AAGTCTTTTA TATTTAAATA
3D7             .......... .......... .......... .......... .......... .......... .......... ..........
RO33            .......... .......... .......... .......... .......... .......... .......... ..........
Palo Alto       .......... .......... .......... .......... .......... .......... .......... ..........
FCR3            .......... .......... .......... .......... .......... .......... .......... ..........
Wellcome        .......... .......... .......... .......... .......... .......... .......... ..........
D6              .......... .......... .......... .......... .......... .......... .......... ..........
T996            .......... .......... .......... .......... .......... .......... .......... ..........
T9102           .......... .......... .......... .......... .......... .......... .......... ..........
K1              .......... .......... .......... .......... .......... .......... .......... ..........
Dd2             .......... .......... .......... .......... .......... .......... .......... ..........
D10             .......... .......... .......... .......... .......... .......... .......... ..........
FCC2            .......... .......... .......... .......... .......... .......... .......... ..........
HB3             .......... .......... .......... .......... .......... .......... .......... ..........
7G8             .......... .......... .......... .......... .......... .......... .......... ..........
P.reichenowi    .......... .......... .......... .......... ...A...... .......... .......... .........G

                      2090       2100       2110       2120       2130       2140       2150       2160
                ....|....| ....|....| ....|....| ....|....| ....|....| ....|....| ....|....| ....|....|
PF13_0338       ATAACAATGT AAAAAAATTA ATAGATGATA AAAGTATAAT ACATTTCTAT TGTGAATGTA ATGTTAATAA TAATGTAATC
3D7             .......... .......... .......... .......... .......... .......... .......... ..........
RO33            .......... .......... .......... .......... .......... .......... .......... ..........
Palo Alto       .......... .......... .......... .......... .......... .......... .......... ..........
FCR3            .......... .......... .......... .......... .......... .......... .......... ..........
Wellcome        .......... .......... .......... .......... .......... .......... .......... ..........
D6              .......... .......... .......... .......... .......... .......... .......... ..........
T996            .......... .......... .......... .......... .......... .......... .......... ..........
T9102           .......... .......... .......... .......... .......... .......... .......... ..........
K1              .......... .......... .......... .......... .......... .......... .......... ..........
Dd2             .......... .......... .......... .......... .......... .......... .......... ..........
D10             .......... .......... .......... .......... .......... .......... .......... ..........
FCC2            .......... .......... .......... .......... .......... .......... .......... ..........
HB3             .......... .......... .......... .......... .......... .......... .......... ..........
7G8             .......... .......... .......... .......... .......... .......... .......... ..........
P.reichenowi    .C........ ...G...... .A........ ...C...... .......... .......... .......... ..........
```

```
                         2170       2180       2190       2200       2210       2220       2230       2240
                     ....|....| ....|....| ....|....| ....|....| ....|....| ....|....| ....|....| ....|....|
PF13_0338            AAAGTTAATT ATTATATATC TCCATTTTAT GATGAAAATA GTATAAAACA AGAAATCAAC AAAAAAGACC AAGAAATAAC
3D7                  .......... .......... .......... .......... .......... .......... .......... ..........
RO33                 .......... .......... .......... .......... .......... .......... .......... ........C.
Palo Alto            .......... .......... .......... .......... .......... .......... .......... ..........
FCR3                 .......... .......... .......... .......... .......... .......... .......... ........C.
Wellcome             .......... .......... .......... .......... .......... .......... .......... ........C.
D6                   .......... .......... .......... .......... .......... .......... .......... ..........
T996                 .......... .......... .......... .......... .......... .......... .......... ........C.
T9102                .......... .......... .......... .......... .......... .......... .......... ........C.
K1                   .......... .......... .......... .......... .......... .......... .......... ........C.
Dd2                  .......... .......... .......... .......... .......... .......... .......... ........C.
D10                  .......... .......... .......... .......... .......... .......... .......... ........C.
FCC2                 .......... .......... .......... .......... .......... .......... .......... ........C.
HB3                  .......... .......... .......... .......... .......... .......... .......... ........C.
7G8                  .......... .......... .......... .......... .......... .......... .......... ........C.
P.reichenowi         .......... .......... .......... .......... ........A. ......A.G. .......... ........C.

                         2250       2260       2270       2280       2290       2300       2310       2320
                     ....|....| ....|....| ....|....| ....|....| ....|....| ....|....| ....|....| ....|....|
PF13_0338            TATGATTAAT AAAACTATAC CACAAGATGA AAAAGATATT CTATTCAATA ATGAAAAAGT TGTTCCTTTA TCAAACGAAC
3D7                  .......... .......... .......... .......... .......... .......... .......... ..........
RO33                 .......... .......... .......... .......... .......... .......... .......... ..........
Palo Alto            .......... .......... .......... .......... .......... .......... .......... ..........
FCR3                 .......... .......... .......... .......... .......... .......... .......... ..........
Wellcome             .......... .......... .......... .......... .......... .......... .......... ..........
D6                   .......... .......... .......... .......... .......... .......... .......... ..........
T996                 .......... .......... .......... .......... .......... .......... .......... ..........
T9102                .......... .......... .......... .......... .......... .......... .......... ..........
K1                   .......... .......... .......... .......... .......... .......... .......... ..........
Dd2                  .......... .......... .......... .......... .......... .......... .......... ..........
D10                  .......... .......... .......... .......... .......... .......... .......... ..........
FCC2                 .......... .......... .......... .......... .......... .......... .......... ..........
HB3                  .......... .......... .......... .......... .......... .......... .......... ..........
7G8                  .......... .......... .......... .......... .......... .......... .......... ..........
P.reichenowi         ...T...... .......... .....A.... .......... .......... .......... .......... ..........

                         2330       2340       2350       2360       2370       2380       2390       2400
                     ....|....| ....|....| ....|....| ....|....| ....|....| ....|....| ....|....| ....|....|
PF13_0338            CTCAAGAAAT TATTCAACCA CCTATTCAGG AAAAATTAAA TACTACTGAT CCTTCAAAAG CATATATATA TGGAGCTAAT
3D7                  .......... .......... .......... .......... .......... .......... .......... ..........
RO33                 .......... .......... .......... .......... .......... .......... .......... ..........
Palo Alto            .......... .......... .......... .......... .......... .......... .......... ..........
FCR3                 .......... .......... .......... .......... .......... .......... .......... ..........
Wellcome             .......... .......... .......... .......... .......... .......... .......... ..........
D6                   .......... .......... .......... .......... .......... .......... .......... ..........
T996                 .......... .......... .......... .......... .......... .......... .......... ..........
T9102                .......... .......... .......... .......... .......... .......... .......... ..........
K1                   .......... .......... .......... .......... .......... .......... .......... ..........
Dd2                  .......... .......... .......... .......... .......... .......... .......... ..........
D10                  .......... .......... .......... .......... .......... .......... .......... ..........
FCC2                 .......... .......... .......... .......... .......... .......... .......... ..........
HB3                  .......... .......... .......... .......... .......... .......... .......... ..........
7G8                  .......... .......... .......... .......... .......... .......... .......... ..........
P.reichenowi         .......... .......... ........A. .......... .......... .......... .......... ..........

                         2410       2420       2430       2440       2450
                     ....|....| ....|....| ....|....| ....|....| ....|....| ....|..
PF13_0338            ATTATATTTA TTGCAATTAT ATCTATAATA TCCTTGTCTA TTTCTTCTTT CATATAA
3D7                  .......... .......... .......... ........
RO33                 .......... .......... .......... ........
Palo Alto            .......... .......... .......... ........
FCR3                 .......... .......... .......... ........
Wellcome             .......... .......... .......... ........
D6                   .......... .......... .......... ........
T996                 .......... .......... .......... ........
T9102                .......... .......... .......... ........
K1                   .......... .......... .......... ........
Dd2                  .......... .......... .......... ........
D10                  .......... .......... .......... ........
FCC2                 .......... .......... .......... ........
HB3                  .......... .......... .......... ........
7G8                  .......... .......... .......... ........
P.reichenowi         .......... .......... ..G....... .G........ .......... .
```

PFE0395c

```
                      10         20         30         40         50         60         70         80
              ....|....| ....|....| ....|....| ....|....| ....|....| ....|....| ....|....| ....|....|
PFE0395c      ATGAAACGCT GGTCAATCAT TACAGGAATC GTTATAATAT TCTGCATATT AACATGTAAA GGTCAAGTAG AAAACAAAAA
3D7                                     ...... .......... .......... .......... .......... ..........
RO33                                    ...... .......... .......... .......... .......... ..........
Palo Alto                               ...... .......... .......... .......... .......... ..........
FCR3                                    ...... .......... .......... .......... .......... ..........
Wellcome                                ...... .......... .......... .......... .......... ..........
D6                                      ...... .......... .......... .......... .......... ..........
T996                                    ...... .......... .......... .......... .......... ..........
T9102                                   ...... .......... .......... .......... .......... ..........
K1                                      ...... .......... .......... .......... .......... ..........
Dd2                                     ...... .......... .......... .......... .......... ..........
D10                                     ...... .......... .......... .......... .......... ..........
FCC2                                    ...... .......... .......... .......... .......... ..........
HB3                                     ...... .......... .......... .......... .......... ..........
7G8                                     ...... .......... .......... .......... .......... ..........
P.reichenowi                            ...... .......... .......... .......... .......... ..........

                      90        100        110        120        130        140        150        160
              ....|....| ....|....| ....|....| ....|....| ....|....| ....|....| ....|....| ....|....|
PFE0395c      GGTTGATTTT CGAACAGAAA AAGGAAAGTT CGTGCCTTTA AATCTAGTAC CAGGAGATGT TGTAGAATAT TCGTGTCCGT
3D7           .......... .......... .......... .......... .......... .......... .......... ..........
RO33          .......... .......... ..A......C .......... .......... .......... .......... ..........
Palo Alto     .......... .......... ..A....... .......... .......... .......... .......... ..........
FCR3          .......... .......... .......... .......... .......... .......... .......... ..........
Wellcome      .......... .......... ..A....... .......... .......... .......... .......... ..........
D6            .......... .......... ..A....... .......... .......... .......... .......... ..........
T996          .......... .......... ..A....... .......... .......... .......... .......... ..........
T9102         .......... .......... ..A......C .......... .......... .......... .......... ..........
K1            .......... .......... .......... .......... .......... .......... .......... ..........
Dd2           .......... .......... .......... .......... .......... .......... .......... ..........
D10           .......... .......... ..A....... .......... .......... .......... .......... ..........
FCC2          .......... .......... ..A....... .......... .......... .......... .......... ..........
HB3           .......... .......... ..A......C .......... .......... .......... .......... ..........
7G8           .......... .......... .......... .......... .......... .......... .......... ..........
P.reichenowi  ...G...... .......... ..A....... T......... .......... .......... .......... ..A.....A.

                     170        180        190        200        210        220        230        240
              ....|....| ....|....| ....|....| ....|....| ....|....| ....|....| ....|....| ....|....|
PFE0395c      ATAGTTTAAA TAATGATATC CGAAATATGA ATGGGGTTGA ACGAGAACAT TTCGACAATA AAAAATTTTG TTTTGATTAT
3D7           .......... .......... .......... .......... .......... .......... .......... ..........
RO33          .......... .......... .......... .......... .......... .......... .......... ..........
Palo Alto     .......... .......... .......... .......... .......... ....T..... ..C....... ..........
FCR3          .......... .......... .......... .......... .......... .......... ..C....... ..........
Wellcome      .......... .......... .......... .......... .......... .......... .......... ..........
D6            .......... .......... .......... ........A. .......... .......... ..C....... ..........
T996          .......... .......... .......... .......... .......... .......... ..C....... ..........
T9102         .......... .......... .......... .......... .......... .......... .......... ..........
K1            .......... .......... .......... .......... .......... .......... .......... ..........
Dd2           .......... .......... .......... .......... .......... .......... ..C....... ..........
D10           .......... .......... .......... .......... .......... .......... ..C....... ..........
FCC2          .......... .......... .......... .......... .......... .......... .......... ..........
HB3           .......... .......... .......... .......... .......... .......... .......... ..........
7G8           .......... .......... .......... .......... .......... .......... .......... ..........
P.reichenowi  .......... .......... .......... .......... .......... ........G. ..C....... ..........

                     250        260        270        280        290        300        310        320
              ....|....| ....|....| ....|....| ....|....| ....|....| ....|....| ....|....| ....|....|
PFE0395c      ATATTTGTTG GAAGTAAGTT AACATTTTTA AAAGAATATG TTCGTGGTTC TTATAATGTG GTACATAAAG AAGAAGGGAA
3D7           .......... .......... .......... .......... .......... .......... .......... ..........
RO33          .......... .......... .......... .......... .......... .......... .......... ..........
Palo Alto     .......... .......... .......... .......... .......... .......... .......... ..........
FCR3          .......... .......... .......... .......... .......... .......... .........A ..........
Wellcome      .......... .......... .......... .......... .......... .......... .......... ..........
D6            .......... .......... .......... .......... .......... .......... .......... ..........
T996          .......... .......... .......... .......... .......... .......... .......... ..........
T9102         .......... .......... .......... .......... .......... .......... .......... ..........
K1            .......... .......... .......... .......... .......... .......... .......... ..........
Dd2           .......... .......... .......... .......... .......... .......... .........A ..........
D10           .......... .......... .......... .......... .......... .......... .......... ..........
FCC2          .......... .......... .......... .......... .......... .......... .......... ..........
HB3           .......... .......... .......... .......... .......... .......... .......... ..........
7G8           .......... .......... .......... .......... .......... .......... .........A ..........
P.reichenowi  .......... .......... .......... .......... .......... .......... .......... ..........
```

```
                     330        340        350        360        370        380        390        400
              ....|....| ....|....| ....|....| ....|....| ....|....| ....|....| ....|....| ....|....|
PFE0395c      TTTATATACA TCACAATTTA GTGTTCCTCC TGTTGTTTTA ACACATCGAA ATTTTGATTG TTTTTGTTAT ATGGAAGAAA
3D7           .......... .......... .......... .......... .......... .......... .......... ..........
RO33          .......... .......... .......... .......... .......... .......... .......... ..........
Palo Alto     .......... .......... .......... .......... .......... .......... .......... ..........
FCR3          .......... .......... .......... .......... .......... .......... .......... ..........
Wellcome      .......... .......... .......... .......... .......... .......... .......... ..........
D6            .......... .......... .......... .......... .......... .......... .......... ..........
T996          .......... .......... .......... .......... .......... .......... .......... ..........
T9102         .......... .......... .......... .......... .......... .......... .......... ..........
K1            .......... .......... .......... .......... .......... .......... .......... ..........
Dd2           .......... .......... .......... .......... .......... .......... .......... ..........
D10           .......... .......... .......... .......... .......... .......... .......... ..........
FCC2          .......... .......... .......... .......... .......... .......... .......... ..........
HB3           .......... .......... .......... .......... .......... .......... .......... ..........
7G8           .......... .......... .......... .......... .......... .......... .......... ..........
P.reichenowi  .......... .......... .......... .......... .......... .......... .......... ..........

                     410        420        430        440        450        460        470        480
              ....|....| ....|....| ....|....| ....|....| ....|....| ....|....| ....|....| ....|....|
PFE0395c      ACAATGTTGT GGTTAAGAAG GTTTTAAGAA TACATATATC AAATGGTGTT CTTAGGAAAA TACCGGGATG TGATTTTAAT
3D7           .......... .......... .......... .......... .......... .......... .......... ..........
RO33          .......... .......... .......... .......... .......... .......... .......... ..........
Palo Alto     .......... .......... .......... .......... .......... .......... .......... ..........
FCR3          .......... .......... .......... .......... .......... .......... .......... ..........
Wellcome      .......... .......... .......... .......... .......... .......... .......... ..........
D6            .......... .......... ........C. .......... .......... .......... .......... ..........
T996          .......... .......... .......... .......... .......... .......... .......... ..........
T9102         .......... .......... .......... .......... .......... .......... .......... ..........
K1            .......... .......... .......... .......... .......... .......... .......... ..........
Dd2           .......... .......... .......... .......... .......... .......... .......... ..........
D10           .......... .......... .......... .......... .......... .......... .......... ..........
FCC2          .......... .......... .......... .......... .......... .......... .......... ..........
HB3           .......... .......... .......... .......... .......... .......... .......... ..........
7G8           .......... .......... .......... .......... .......... .......... .......... ..........
P.reichenowi  .T........ .......... .......... .......... .......... .......... .......... ..........

                     490        500        510        520        530        540        550        560
              ....|....| ....|....| ....|....| ....|....| ....|....| ....|....| ....|....| ....|....|
PFE0395c      GCTGATTATA AAGAATCGAC AGCTATAACT ACTTTTAGTA ATATGAGTCC TCGTCGTGTA AAAGTATGTG ACGTATATCC
3D7           .......... .......... .......... .......... .......... .......... .......... ..........
RO33          .......... .......... .......... .......... .......... .......... .......... ..........
Palo Alto     .......... .......... .......... .......... .......... .......... .......... ..........
Wellcome      .......... .......... .......... .......... .......... .......... .......... ..........
D6            .......... .......... .......... .......... .......... .......... .......... ..........
T996          .......... .......... .......... .......... .......... .......... .......... ..A.......
T9102         .......... .......... .......... .......... .......... .......... .......... ..........
K1            .......... .......... .......... .......... .......... .......... .......... ..........
Dd2           .......... .......... .......... .......... .......... .......... .......... ..........
D10           .......... .......... .......... .......... .......... .......... .......... ..........
FCC2          .......... .......... .......... .......... .......... .......... .......... ..........
HB3           .......... .......... .......... .......... .......... .......... .......... ..........
7G8           .......... .......... .......... .......... .......... .......... .......... ..........
P.reichenowi  .......... .......... .......... .......... .......... .......... .......... ..........

                     570        580        590        600        610        620        630        640
              ....|....| ....|....| ....|....| ....|....| ....|....| ....|....| ....|....| ....|....|
PFE0395c      GAAGAGTGGA GATTTCATAA GTTTAATGTG TCCATCAGAT TATTCAATTA AACCTGATGG TTGTTTTTCA AATGTATATG
3D7           .......... .......... .......... .......... .......... .......... .......... ..........
RO33          .......... .......... .......... .......... .......... .......... .......... ..........
Palo Alto     .......... .......... .......... .......... .......... .......... .......... ..........
FCR3          .......... .......... .......... .......... .......... .......... .......... ..........
Wellcome      .......... .......... .......... .......... .......... .......... .......... ..........
D6            .......... .......... .......... .......... .......... .......... .......... ..........
T996          .......... .......... .......... ........G. .......... .......... .......... ..........
T9102         .......... .......... .......... .......... .......... .......... .......... ..........
K1            .......... .......... .......... .......... .......... .......... .......... ..........
Dd2           .......... .......... .......... .......... .......... .......... .......... ..........
D10           .......... .......... .......... .......... .......... .......... .......... ..........
FCC2          .......... .......... .......... .......... .......... .......... .......... ..........
HB3           .......... .......... .......... .......... .......... .......... .......... ..........
7G8           .......... .......... .......... .......... .......... .......... .......... ..........
P.reichenowi  .......... .......... .......... .......... .......... .......... .......... ..........
```

```
                                    650        660        670        680        690        700        710        720
                           ....|....| ....|....| ....|....| ....|....| ....|....| ....|....| ....|....| ....|....|
PFE0395c                   TAAAAAGATA TCCTAATGAA GAAGTGAAAG AAGAAGATCG TTTTAATTTG AATAGAAAAT GGGATGCAAG TAAATATAAT
3D7                        .......... .......... .......... .......... .......... .......... .......... ..........
RO33                       .......... .......... .......... .......... .......... .......... .......... ..........
Palo Alto                  .......... .......... .......... .......... .......... .......... .......... ..........
FCR3                       .......... .......... .......... .......... .......... .......... .......... ..........
Wellcome                   .......... .......... .......... .......... .......... .......... .......... ..........
D6                         .......... .......... .......... .......... .......... .......... .......... ..........
T996                       .......... .......... .......... .......... .......... .......... .......... ..........
T9102                      .......... .......... .......... .......... .......... .......... .......... ..........
K1                         .......... .......... .......... .......... .......... .......... .......... ..........
Dd2                        .......... .......... .......... .......... .......... .......... .......... ..........
D10                        .......... .......... .......... .......... .......... .......... .......... ..........
FCC2                       .......... .......... .......... .......... .......... .......... .......... ..........
HB3                        .......... .......... .......... .......... .......... .......... .......... ..........
7G8                        .......... .......... .......... .......... .......... .......... .......... ..........
P.reichenowi               ......A... .......... .......... .......... .......A.. .......... .......... ..........

                                    730        740        750        760        770        780        790        800
                           ....|....| ....|....| ....|....| ....|....| ....|....| ....|....| ....|....| ....|....|
PFE0395c                   GTTGTGTCTA TAGAAACAGT TCTAAAAATG AATATGATAA CACAAGGTGA TAAATATTCT ATATTTTCAA AATTACCAGA
3D7                        .......... .......... .......... .......... .......... .......... .......... ..........
RO33                       .......... .......... .......... .......... .......... .......... .......... ..........
Palo Alto                  .......... .......... .......... .......... .......... .......... .......... ..........
FCR3                       .......... .......... .......... .......... .......... .......... .......... ..........
Wellcome                   .......... .......... .......... .......... .......... .......... .......... ..........
D6                         .......... .......... .......... .......... .......... .......... .......... ..........
T996                       .......... .......... .......... .......... .......... .......... .......... ..........
T9102                      .......... .......... .......... .......... .......... .......... .......... ..........
K1                         .......... .......... .......... .......... .......... .......... .......... ..........
Dd2                        .......... .......... .......... .......... .......... .......... .......... ..........
D10                        .......... .......... .......... .......... .......... .......... .......... ..........
FCC2                       .......... .......... .......... .......... .......... .......... .......... ..........
HB3                        .......... .......... .......... .......... .......... .......... .......... ..........
7G8                        .......... .......... .......... .......... .......... .......... .......... ..........
P.reichenowi               ......C... .......... .........T .......... .......... .......... .......... ..........

                                    810        820        830        840        850        860        870        880
                           ....|....| ....|....| ....|....| ....|....| ....|....| ....|....| ....|....| ....|....|
PFE0395c                   TGTAAAAGAT CAAGTTGATT TTACATGTAT TTGTCAATCA AATGATGAAC AAGATAATCT AATGATGAAT GTTTATATAA
3D7                        .......... .......... .......... .......... .......... .......... .......... ..........
RO33                       .......... .......... .......... .......... .......... .......... .......... ..........
Palo Alto                  .......... .......... .......... .......... .......... .......... .......... ..........
FCR3                       .......... .......... .......... .......... .......... .......... .......... ..........
Wellcome                   .......... .......... .......... .......... .......... .......... .......... ..........
D6                         .......... .......... .......... .......... .......... .......... .......... ..........
T996                       .......... .......... .......... .......... .......... .......... .......... ..........
T9102                      .......... .......... .......... .......... .......... .......... .......... ..........
K1                         .......... .......... .......... .......... .......... .......... .......... ..........
Dd2                        .......... .......... .......... .......... .......... .......... .......... ..........
D10                        .......... .......... .......... .......... .......... .......... .......... ..........
FCC2                       .......... .......... .......... .......... .......... .......... .......... ..........
HB3                        .......... .......... .......... .......... .......... .......... .......... ..........
7G8                        .......... .......... .......... .......... .......... .......... .......... ..........
P.reichenowi               .......A.. .......... .......... .......... ...A...... .......... .......... ........G.

                                    890        900        910        920        930        940        950        960
                           ....|....| ....|....| ....|....| ....|....| ....|....| ....|....| ....|....| ....|....|
PFE0395c                   ATAACACATC TTACCTTACA AATAATACAA GAAGTATTGG AGTTAACAAG CATTCATTTT CTAATTCTGA AATTTTTGAA
3D7                        .......... .......... .......... .......... ....
RO33                       .......... .......... .......... .......... ....
Palo Alto                  .......... .C......T. .......... .......... ....
FCR3                       .......... .C........ .......... .......... ....
Wellcome                   .......... .C........ .......... .......... ....
D6                         .......... .......... .......... .......... ....
T996                       .......... .......... .......... .......... ....
T9102                      .......... .C........ .......... .......... ....
K1                         .......... .......... .......... .......... ....
Dd2                        .......... .C........ .......... .......... ....
D10                        .......... .C........ .......... .......... ....
FCC2                       .......... .C........ .......... .......... ....
HB3                        .......... .C........ .......... .......... ....
7G8                        .......... .C........ .......... .......... ....
P.reichenowi               ......A... .CTGA..... .......... .......... ....
```

```
                              970        980        990        1000       1010       1020       1030       1040
                      ....|....| ....|....| ....|....| ....|....| ....|....| ....|....| ....|....| ....|....|
PFE0395c              AGAATAGAAA GAGAAGAAAT TTCATTTGCG TTTTCTTCCT ATTTATCAAT AACTTTAATT CTTTTATATC TTTTCTTTTT
3D7
RO33
Palo Alto
FCR3
Wellcome
D6
T996
T9102
K1
Dd2
D10
FCC2
HB3
7G8
P.reichenowi
```

```
                              1050
                      ....|....|
PFE0395c              AAACTTTTAA
3D7
RO33
Palo Alto
FCR3
Wellcome
D6
T996
T9102
K1
Dd2
D10
FCC2
HB3
7G8
P.reichenowi
```

PF14_0201(Pf113)

```
                               10         20         30         40         50         60         70         80
                      ....|....| ....|....| ....|....| ....|....| ....|....| ....|....| ....|....| ....|....|
PF14_0201             ATGAAAATAC CGTTTTTTAT TTTACATATT TTATTATTAC AATTTTTATT ATGTTTAATA CGTTGTTATG TGCACAATGA
3D7
RO33
Palo Alto
FCR3
Wellcome
D6
T996
T9102
K1
Dd2
D10
FCC2
HB3
7G8
P.reichenowi
```

```
                               90        100        110        120        130        140        150        160
                      ....|....| ....|....| ....|....| ....|....| ....|....| ....|....| ....|....| ....|....|
PF14_0201             TGTAATAAAA TTTGGAGAAG AAAATTCGTT AAAATGTTCA CAAGGAAACC TGTATGTGTT ACATTGTGAA GTTCAATGTT
3D7                                                        ...... .......... .......... .......... ..........
RO33                                                       ...... .......... .......... .......... ..........
Palo Alto                                                  ...... .......... .......... .......... ..........
FCR3                                                       ...... .......... .......... .......... ..........
Wellcome                                                   ...... .......... .......... .......... ..........
D6                                                         ...... .......... .......... .......... ..........
T996                                                       ...... .......... .......... .......... ..........
T9102                                                      ...... .......... .......... .......... ..........
K1                                                         ...... .......... .......... .......... ..........
Dd2                                                        ...... .......... .......... .......... ..........
D10                                                        ...... .......... .......... .......... ..........
FCC2                                                       ...... .......... .......... .......... ..........
HB3                                                        ...... .......... .......... .......... ..........
7G8                                                        ...... .......... .......... .......... ..........
P.reichenowi                                               ...... .......... .......... ...C...... ..........
```

```
                               170        180        190        200        210        220        230        240
                      ....|....| ....|....| ....|....| ....|....| ....|....| ....|....| ....|....| ....|....|
PF14_0201             TGAATGGGAA TAACGAGATA ATACATAAAA GATGTAATGA CGACATAGAA AAGAAATGTA ATGGTAATAA TAAATGCATA
3D7                   .......... .......... .......... .......... .......... .......... .......... ..........
RO33                  .......... .......... .......... .......... .......... .......... .......... ..........
Palo Alto             .......... .......... .......... .......... .......... .......... .......... ..........
FCR3                  .......... .......... .......... .......... .......... .......... .......... ..........
Wellcome              .......... .......... .......... .......... .......... .......... .......... ..........
D6                    .......... .......... .......... .......... .......... .......... .......... ..........
T996                  .......... .......... .......... .......... .......... .......... .......... ..........
T9102                 .......... .......... .......... .......... .......... .......... .......... ..........
K1                    .......... .......... .......... .......... .......... .......... .......... ..........
Dd2                   .......... .......... .......... .......... .......... .......... .......... ..........
D10                   .......... .......... .......... .......... .......... .......... .......... ..........
FCC2                  .......... .......... .......... .......... .......... .......... .......... ..........
HB3                   .......... .......... .......... .......... .......... .......... .......... ..........
7G8                   .......... .......... .......... .......... .......... .......... .......... ..........
P.reichenowi          .......... .......... .......... .......... .......... .......... .......... C.........

                               250        260        270        280        290        300        310        320
                      ....|....| ....|....| ....|....| ....|....| ....|....| ....|....| ....|....| ....|....|
PF14_0201             TATTTTTTTG AATATGAACT TCGAAAAAAA ACACAATCCT TTCGAAATAA GAATAGTATA GAAATAAGTG AATGTGTAGA
3D7                   .......... .......... .......... .......... .......... .......... .......... ..........
RO33                  .......... .......... .......... .......... .......... .......... .......... ..........
Palo Alto             .......... .......... .......... .......... .......... .......... .......... ..........
FCR3                  .......... .......... .......... .......... .......... .......... .......... ..........
Wellcome              .......... .......... .......... .......... .......... .......... .......... ..........
D6                    .......... .......... .......... .......... .......... .......... .......... ..........
T996                  .......... .......... .......... .......... .......... .......... .......... ..........
T9102                 .......... .......... .......... .......... .......... .......... .......... ..........
K1                    .......... .......... .......... .......... .......... .......... .......... ..........
Dd2                   .......... .......... .......... .......... .......... .......... .......... ..........
D10                   .......... .......... .......... .......... .......... .......... .......... ..........
FCC2                  .......... .......... .......... .......... .......... .......... .......... ..........
HB3                   .......... .......... .......... .......... .......... .......... .......... ..........
7G8                   .......... .......... .......... .......... .......... .......... .......... ..........
P.reichenowi          .......... .......... .......... ........T. .......... .......... .......... ..........

                               330        340        350        360        370        380        390        400
                      ....|....| ....|....| ....|....| ....|....| ....|....| ....|....| ....|....| ....|....|
PF14_0201             ATCGGAACAG AACGAAGTAA AAACATCTAC GACTTGTTTA TTAAGTAATA GTTTTATTTT AGATGAAGCA TTTATTCAAT
3D7                   .......... .......... .......... .......... .......... .......... .......... ..........
RO33                  .......... .......... .......... .......... .......... .......... .......... ..........
Palo Alto             .......... .......... .......... .......... .......... .......... .......... ..........
FCR3                  .......... .......... .......... .......... .......... .......... .......... ..........
Wellcome              .......... .......... .......... .......... .......... .......... .......... ..........
D6                    .......... .......... .......... .......... .......... .......... .......... ..........
T996                  .......... .......... .......... .......... .......... .......... .......... ..........
T9102                 .......... .......... .......... .......... .......... .......... .......... ..........
K1                    .......... .......... .......... .......... .......... .......... .......... ..........
Dd2                   .......... .......... .......... .......... .......... .......... .......... ..........
D10                   .......... .......... .......... .......... .......... .......... .......... ..........
FCC2                  .......... .......... .......... .......... .......... .......... .......... ..........
HB3                   .......... .......... .......... .......... .......... .......... .......... ..........
7G8                   .......... .......... .......... .......... .......... .......... .......... ..........
P.reichenowi          .......... .......... .......... .......... .......... .......... .......... ..........

                               410        420        430        440        450        460        470        480
                      ....|....| ....|....| ....|....| ....|....| ....|....| ....|....| ....|....| ....|....|
PF14_0201             ATTTCTTTTT TATTAAGAAT AAGAATGAGG AACCTGTTAT TTGTAAAGAT GGTAATATAA ATATTAAAAG TGCACTTTTA
3D7                   .......... .......... .......... .......... .......... .......... .......... ..........
RO33                  .......... .......... .......... .......... .......... .......... .......... ..........
Palo Alto             .......... .......... .......... .......... .......... .......... .......... ..........
FCR3                  .......... .......... .......... .......... .......... .......... .......... ..........
Wellcome              .......... .......... .......... .......... .......... .......... .......... ..........
D6                    .......... .......... .......... .......... .......... .......... .......... ..........
T996                  .......... .......... .......... .......... .......... .......... .......... ..........
T9102                 .......... .......... .......... .......... .......... .......... .......... ..........
K1                    .......... .......... .......... .......... .......... .......... .......... ..........
Dd2                   .......... .......... .......... .......... .......... .......... .......... ..........
D10                   .......... .......... .......... .......... .......... .......... .......... ..........
FCC2                  .......... .......... .......... .......... .......... .......... .......... ..........
HB3                   .......... .......... .......... .......... .......... .......... .......... ..........
7G8                   .......... .......... .......... .......... .......... .......... .......... ..........
P.reichenowi          .......... .......... .......... .......... .......... .......... .......... ..........
```

```
                         490        500        510        520        530        540        550        560
                  ....|....| ....|....| ....|....| ....|....| ....|....| ....|....| ....|....| ....|....|
PF14_0201         CATTCTCCCT TTTGTGAAAT AAAATTAAAA GATATATCAG AATATATTAG AAAAAAATGT GATAATAATA AAGAATGTTT
3D7               .......... .......... .......... .......... .......... .......... .......... ..........
RO33              .......... .......... .......... .......... .......... .......... .......... ..........
Palo Alto         .......... .......... .......... .......... .......... .......... .......... ..........
FCR3              .......... .......... .......... .......... .......... .......... .......... ..........
Wellcome          .......... .......... .......... .......... .......... .......... .......... ..........
D6                .......... .......... .......... .......... .......... .......... .......... ..........
T996              .......... .......... .......... .......... .......... .......... .......... ..........
T9102             .......... .......... .......... .......... .......... .......... .......... ..........
K1                .......... .......... .......... .......... .......... .......... .......... ..........
Dd2               .......... .......... .......... .......... .......... .......... .......... ..........
D10               .......... .......... .......... .......... .......... .......... .......... ..........
FCC2              .......... .......... .......... .......... .......... .......... .......... ..........
HB3               .......... .......... .......... .......... .......... .......... .......... ..........
7G8               .......... .......... .......... .......... .......... .......... .......... ..........
P.reichenowi      .......... .......... .......... .......... .......... .......... .......... ..........

                         570        580        590        600        610        620        630        640
                  ....|....| ....|....| ....|....| ....|....| ....|....| ....|....| ....|....| ....|....|
PF14_0201         AATAGATCCA TTAGATGTAC AGAAAAATTT ATTAAATGAA GAAGATCCAT GTTATATTAA TAATTCGTAT GTGTCTGTTA
3D7               .......... .......... .......... .......... .......... .......... .......... ..........
RO33              .......... .......... .......... .......... .......... .......... .......... ..........
Palo Alto         .......... .......... .......... .......... .......... .......... .......... ..........
FCR3              .......... .......... .......... .......... .......... .......... .......... ..........
Wellcome          .......... .......... .......... .......... .......... .......... .......... ..........
D6                .......... .......... .......... .......... .......... .......... .......... ..........
T996              .......... .......... .......... .......... .......... .......... .......... ..........
T9102             .......... .......... .......... .......... .......... .......... .......... ..........
K1                .......... .......... .......... .......... .......... .......... .......... ..........
Dd2               .......... .......... .......... .......... .......... .......... .......... ..........
D10               .......... .......... .......... .......... .......... .......... .......... ..........
FCC2              .......... .......... .......... .......... .......... .......... .......... ..........
HB3               .......... .......... .......... .......... .......... .......... .......... ..........
7G8               .......... .......... .......... .......... .......... .......... .......... ..........
P.reichenowi      .......... .......... ......C... .......... .......... .......... ......T... .....A....

                         650        660        670        680        690        700        710        720
                  ....|....| ....|....| ....|....| ....|....| ....|....| ....|....| ....|....| ....|....|
PF14_0201         ATGTTGTATG TAATAAAGAA GAAGAAATTG GAGATGAAAG TACAGATTCT AGTTCAATGG AAATTCAGGA TTCTACATCT
3D7               .......... .......... .......... .......... .......... .......... .......... ..........
RO33              .......... .......... .......... .......... .......... .......... .......... ..........
Palo Alto         .......... .......... .......... .......... .......... .......... .......... ..T.......
FCR3              .......... .......... .......... .......... .......... .........A .......... ..........
Wellcome          .......... .......... .......... .......... .......... .........A .......... ..........
D6                .......... .......... .......... .......... .......... .......... .......... ..........
T996              .......... .......... .......... .......... .......... .......... .......... ..........
T9102             .......... .......... .......... .......... .......... .......... .......... ..........
K1                .......... .......... .......... .......... .......... .........A .......... ..........
Dd2               .......... .......... .......... .......... .......... .......... .......... ..........
D10               .......... .......... .......... .......... .......... .........A .......... ..........
FCC2              .......... .......... .......... .......... .......... .......... .......... ..........
HB3               .......... .......... .......... .......... .......... .........A .......... ..........
7G8               .......... .......... .......... .......... .......... .........A .......... ..........
P.reichenowi      .......... .......... .......... .......... ...C..G... .........A .......A.. ....GT..T.

                         730        740        750        760        770        780        790        800
                  ....|....| ....|....| ....|....| ....|....| ....|....| ....|....| ....|....| ....|....|
PF14_0201         AATGAACAAG ATGAAAATGT TAAAGGAATG AGT---TCAT CACAAGAAAT GAATTCTAAT AACGACGAAA ATAAAAATCA
3D7               .......... .......... .......... ...---.... .......... .......... .......... ..........
RO33              .......... .......... .......... ...---.... .......... .......... .......... ..........
Palo Alto         .......... .......... .......... ...---.... .......... .......... .......... ..........
FCR3              .......... .......... .......... ...---.... .......... .......... .......... ..........
Wellcome          .......... .......... .......... ...---.... .......... .......... .......... ..........
D6                .......... .......... .......... ...---.... .......... .......... .......... ..........
T996              .......... .......... .......... ...---.... .......... .......... .......... ..........
T9102             .......... .......... .......... ...---.... .......... .......... .......... ..........
K1                .......... .......... .......... ...---.... .......... .......... .......... ..........
Dd2               .......... .......... .......... ...---.... .......... .......... .......... ..........
D10               .......... .......... .......... ...---.... .......... .......... .......... ..........
FCC2              .......... .......... .......... ...---.... .......... .......... .......... ..........
HB3               .......... .......... .......... ...---.... .......... .......... .......... ..........
7G8               .......... .......... .......... ...---.... .......... .......... .......... ..........
P.reichenowi      .......... .......... .......... G..GGT.... .......... .......... ..G...C... ..........
```

```
                      810        820        830        840        850        860        870        880
               ....|....| ....|....| ....|....| ....|....| ....|....| ....|....| ....|....| ....|....|
PF14_0201      AGATAATGAA AGTGATGATG ATGTTAATAA TAATAATAAT AATAATAATG ATGATCAAGA TGAACAAGGT AATGATGGTG
3D7            .......... .......... .......... .......... .......... .......... .......... ..........
RO33           .......... .......... .......... .......... ------.... .......... .......... ..........
Palo Alto      .......... .......... .......... .......... .......... .......... .......... ..........
FCR3           .......... .......... .......... .......... .......... .......... .......... ..........
Wellcome       .......... .......... .......... .......... .......... .......... .......... ..........
D6             .......... .......... .......... .......... ------.... .......... .......... ..........
T996           .......... .......... .......... .......... ------.... .......... .......... ..........
T9102          .......... .......... .......... .......... .......... .......... .......... ..........
K1             .......... .......... .......... .......... .......... .......... .......... ..........
Dd2            .......... .......... .......... .......... .......... .......... .......... ..........
D10            .......... .......... .......... .......... .......... .......... .......... ..........
FCC2           .......... .......... .......... .......... .......... .......... .......... ..........
HB3            .......... .......... .......... .......... .......... .......... .......... ..........
7G8            .......... .......... .......... .......... .......... .......... .......... ..........
P.reichenowi   .......... .......... .......... .......... ---------. .......... .......C.. ..........

                      890        900        910        920        930        940        950        960
               ....|....| ....|....| ....|....| ....|....| ....|....| ....|....| ....|....| ....|....|
PF14_0201      AT---GTTAC CTCTTCTATG AATAAAAATG AAGATAACAA AGATTTAGAA CATGGCTCTT CAAATGATGT TAATAATAAT
3D7            ..---..... .......... .......... .......... .......... .......... .......... ..........
RO33           ..---..... .......... .......... .......... .......... .......... .......... ..........
Palo Alto      ..---..... .......... .......... .......... .......... ....T..... .......... ..........
FCR3           ..---..... .......... .......... .......... .......... .......... .......... ..........
Wellcome       ..---..... .......... .......... .......... .......... .......... .......... ..........
D6             ..---..... .......... .......... .......... .......... .......... .......... ..........
T996           ..---..... .......... .......... .......... .......... .......... .......... ..........
T9102          ..---..... .......... .......... .......... .......... .......... .......... ..........
K1             ..---..... .......... .......... .......... .......... .......... .......... ..........
Dd2            ..---..... .......... .......... .......... .......... .......... .......... ..........
D10            ..---..... .......... .......... .......... .......... .......... .......... ..........
FCC2           ..---..... .......... .......... .......... .......... .......... .......... ..........
HB3            ..---..... .......... .......... .......... .......... .......... .......... ..........
7G8            ..---..... .......... .......... .......... .......... .......... .......... ..........
P.reichenowi   ..GTTA.... .......... .......... .......... .......... .....T.... .......... ..........

                      970        980        990       1000       1010       1020       1030       1040
               ....|....| ....|....| ....|....| ....|....| ....|....| ....|....| ....|....| ....|....|
PF14_0201      ACAGACACTC TTGTTAATAA TAAAGAAAAT AAAGAATTTG TATTAAAAGA AAAATCAAGT TTAACATCTA AAATTAATAA
3D7            .......... .......... .......... .......... .......... .......... .......... ..........
RO33           .......... .......... .......... .......... .......... .......... .......... ..........
Palo Alto      .......... .......... .......... .......... .......... .......... .......... ..........
FCR3           .......... .......... .......... .......... .......... .......... .......... ..........
Wellcome       .......... .......... .......... .......... .......... .......... .......... ..........
D6             .......... .......... .......... .......... .......... .......... .......... ..........
T996           .......... .......... .......... .......... .......... .......... .......... ..........
T9102          .......... .......... .......... .......... .......... .......... .......... ..........
K1             .......... .......... .......... .......... .......... .......... .......... ..........
Dd2            .......... .......... .......... .......... .......... .......... .......... ..........
D10            .......... .......... .......... .......... .......... .......... .......... ..........
FCC2           .......... .......... .......... .......... .......... .......... .......... ..........
HB3            .......... .......... .......... .......... .......... .......... .......... ..........
7G8            .......... .......... .......... .......... .......... .......... .......... ..........
P.reichenowi   .......... .......... .......... .......... .......... .......... .......... ..........

                     1050       1060       1070       1080       1090       1100       1110       1120
               ....|....| ....|....| ....|....| ....|....| ....|....| ....|....| ....|....| ....|....|
PF14_0201      AGAATTAGCA CATAGAACCG CTCTTTTTAA TAAATTAGCT GATAATATAT CCTTATTATT GAATAAAAAA TATGATTCCT
3D7            .......... .......... .......... .......... .......... .......... .......... ..........
RO33           .......... .......... .......... .......... .......... .......... .......... ..........
Palo Alto      .......... .......... .......... .......... .......... .......... .......... ..........
FCR3           .......... .......... .......... .......... .......... .......... .......... ..........
Wellcome       .......... .......... .......... .......... .......... .......... .......... ..........
D6             .......... .......... .......... .......... .......... .......... .......... ..........
T996           .......... .......... .......... .......... .......... .......... .......... ..........
T9102          .......... .......... .......... .......... .......... .......... .......... ..........
K1             .......... .......... .......... .......... .......... .......... .......... ..........
Dd2            .......... .......... .......... .......... .......... .......... .......... ..........
D10            .......... .......... .......... .......... .......... .......... .......... ..........
FCC2           .......... .......... .......... .......... .......... .......... .......... ..........
HB3            .......... .......... .......... .......... .......... .......... .......... ..........
7G8            .......... .......... .......... .......... .......... .......... .......... ..........
P.reichenowi   .......... .......... .......... .......... .......... .A........ .......... ........G.
```

```
                        1130       1140       1150       1160       1170       1180       1190       1200
                ....|....| ....|....| ....|....| ....|....| ....|....| ....|....| ....|....| ....|....|
PF14_0201       TCGAAATTAA AGATGTATTA GAAGATAGAT ATAATGAAAT GAAACGTGAT GCAAATCCAG ATGTATATTA CATTTATTTA
3D7             .......... .......... .......... .......... .......... .......... .......... ..........
RO33            .......... .......... .......... .......... .......... .......... .......... ..........
Palo Alto       .......... .......... .......... .......... .......... .......... .......... ..........
FCR3            .......... .......... .......... .......... .......... .......... .......... ..........
Wellcome        .......... .......... .......... .......... .......... .......... .......... ..........
D6              .......... .......... .......... .......... .......... .......... .......... ..........
T996            .......... .......... .......... .......... .......... .......... .......... ..........
T9102           .......... .......... .......... .......... .......... .......... .......... ..........
K1              .......... .......... .......... .......... .......... .......... .......... ..........
Dd2             .......... .......... .......... .......... .......... .......... .......... ..........
D10             .......... .......... .......... .......... .......... .......... .......... ..........
FCC2            .......... .......... .......... .......... .......... .......... .......... ..........
HB3             .......... .......... .......... .......... .......... .......... .......... ..........
7G8             .......... .......... .......... .......... .......... .......... .......... ..........
P.reichenowi    .......... .......... .......... .......... .......... A......... .......... ..........

                        1210       1220       1230       1240       1250       1260       1270       1280
                ....|....| ....|....| ....|....| ....|....| ....|....| ....|....| ....|....| ....|....|
PF14_0201       ATGGATACAT TAGATATAGA AAAAATAGAA GATATAAATT TAGAAGAAGT TAAAATGTCA TTATTAGCTT CCTTAAAAGA
3D7             .......... .......... .......... .......... .......... .......... .......... ..........
RO33            .......... .......... .......... .......... .......... .......... .......... ..........
Palo Alto       .......... .......... .......... .......... .......... .......... .......... ..........
FCR3            .......... .......... .......... .......... .......... .......... .......... ..........
Wellcome        .......... .......... .......... .......... .......... .......... .......... ..........
D6              .......... .......... .......... .......... .......... .......... .......... ..........
T996            .......... .......... .......... .......... .......... .......... .......... ..........
T9102           .......... .......... .......... .......... .......... .......... .......... ..........
K1              .......... .......... .......... .......... .......... .......... .......... ..........
Dd2             .......... .......... .......... .......... .......... .......... .......... ..........
D10             .......... .......... .......... .......... .......... .......... .......... ..........
FCC2            .......... .......... .......... .......... .......... .......... .......... ..........
HB3             .......... .......... .......... .......... .......... .......... .......... ..........
7G8             .......... .......... .......... .......... .......... .......... .......... ..........
P.reichenowi    .....C.... .......T.. .......... .......... .......... .......... .......... T.........

                        1290       1300       1310       1320       1330       1340       1350       1360
                ....|....| ....|....| ....|....| ....|....| ....|....| ....|....| ....|....| ....|....|
PF14_0201       AACAATGAAT AAAATAGATA CTATAGAAAA GAAAATTGAA GAATTCAAAA ATAAATATAT TTCATTATAT AATAAAGTAA
3D7             .......... .......... .......... .......... .......... .......... .......... ..........
RO33            .......... .......... .......... .......... .......... .......... .......... ..........
Palo Alto       .......... .......... .......... .......... .......... .......... .......... ..........
FCR3            .......... .......... .......... .......... .......... .......... .......... ..........
Wellcome        .......... .......... .......... .......... .......... .......... .......... ..........
D6              .......... .......... .......... .......... .......... .......... .......... ..........
T996            .......... .......... .......... .......... .......... .......... .......... ..........
T9102           .......... .......... .......... .......... .......... .......... .......... ..........
K1              .......... .......... .......... .......... .......... .......... .......... ..........
Dd2             .......... .......... .......... .......... .......... .......... .......... ..........
D10             .......... .......... .......... .......... .......... .......... .......... ..........
FCC2            .......... .......... .......... .......... .......... .......... .......... ..........
HB3             .......... .......... .......... .......... .......... .......... .......... ..........
7G8             .......... .......... .......... .......... .......... .......... .......... ..........
P.reichenowi    .......... .......... .......... .......... .......... .......... .......... ..........

                        1370       1380       1390       1400       1410       1420       1430       1440
                ....|....| ....|....| ....|....| ....|....| ....|....| ....|....| ....|....| ....|....|
PF14_0201       AAACTACCAT GCCAGAACTT TTCGATTTAA ATGAAGATTT AGTATTATTA TATAATGATT TCCCTTTCGA TAACGGTATG
3D7             .......... .......... .......... .......... .......... .......... .......... ..........
RO33            .......... .......... .......... .......... .......... .......... .......... ..........
Palo Alto       .......... .......... .......... .......... .......... .......... .......... ..........
FCR3            .......... .......... .......... .......... .......... .......... .......... ..........
Wellcome        .......... .......... .......... .......... .......... .......... .......... ..........
D6              .......... .......... .......... .......... .......... .......... .......... ..........
T996            .......... .......... .......... .......... .......... .......... .......... ..........
T9102           .......... .......... .......... .......... .......... .......... .......... ..........
K1              .......... .......... .......... .......... .......... .......... .......... ..........
Dd2             .......... .......... .......... .......... .......... .......... .......... ..........
D10             .......... .......... .......... .......... .......... .......... .......... ..........
FCC2            .......... .......... .......... .......... .......... .......... .......... ..........
HB3             .......... .......... .......... .......... .......... .......... .......... ..........
7G8             .......... .......... .......... .......... .......... .......... .......... ..........
P.reichenowi    ....A..A.. .......... .......... .......... .......... .......... .......... ...T..C...
```

```
                         1450       1460       1470       1480       1490       1500       1510       1520
                    ....|....| ....|....| ....|....| ....|....| ....|....| ....|....| ....|....| ....|....|
PF14_0201           ATATCATCTG ATATATTCTT TAAATATAAT CCAAGTGAAA ATATTATGGA TCATCAGGAA ATGGTAAAAA AGGGTTCTAT
3D7                 .......... .......... .......... .......... .......... .......... .......... ..........
RO33                .......... .......... .......... .......... .......... .......... .......... ..........
Palo Alto           .......... .......... .......... .......... .......... .......... .......... ..........
FCR3                .......... .......... .......... .......... .......... .......... .......... ..........
Wellcome            .......... .......... .......... .......... .......... .......... .......... ..........
D6                  .......... .......... .......... .......... .......... .......... .......... ..........
T996                .......... .......... .......... .......... .......... .......... .......... ..........
T9102               .......... .......... .......... .......... .......... .......... .......... ..........
K1                  .......... .......... .......... .......... .......... .......... .......... ..........
Dd2                 .......... .......... .......... .......... ........A. .......... .......... ..........
D10                 .......... .......... .......... .......... .......... .......... .......... ..........
FCC2                .......... .......... .......... .......... .......... .......... .......... ..........
HB3                 .......... .......... .......... .......... .......... .......... .......... ..........
7G8                 .......... .......... .......... .......... .......... .......... .......... ..........
P.reichenowi        ........C. ....T..... .......T.. .........G .A........ ....G..... .......... ......T...

                         1530       1540       1550       1560       1570       1580       1590       1600
                    ....|....| ....|....| ....|....| ....|....| ....|....| ....|....| ....|....| ....|....|
PF14_0201           AACGGAAGAC GAATTAAGAA TTGTTAATGA CCTTGAACCA TTAGATAATT ATAGAAGAAG AAAAAGAATT ACAGAATTAA
3D7                 .......... .......... .......... .......... .......... .......... .......... ..........
RO33                .......... .......... .......... .......... .......... .......... .......... ..........
Palo Alto           .......... .......... .......... .......... .......... .......... .......... ..........
FCR3                .......... .......... .......... .......... .......... .......... .......... ..........
Wellcome            .......... .......... .......... .......... .......... .......... .......... ..........
D6                  .......... .......... .......... .......... .......... .......... .......... ..........
T996                .......... .......... .......... .......... .......... .......... .......... ..........
T9102               .......... .......... .......... .......... .......... .......... .......... ..........
K1                  .......... .......... .......... .......... .......... .......... .......... ..........
Dd2                 .......... .......... .......... .......... .......... .......... .......... ..........
D10                 .......... .......... .......... .......... .......... .......... .......... ..........
FCC2                .......... .......... .......... .......... .......... .......... .......... ..........
HB3                 .......... .......... .......... .......... .......... .......... .......... ..........
7G8                 .......... .......... .......... .......... .......... .......... .......... ..........
P.reichenowi        .........T .......... .......... .......... .......... .......... .......... ..........

                         1610       1620       1630       1640       1650       1660       1670       1680
                    ....|....| ....|....| ....|....| ....|....| ....|....| ....|....| ....|....| ....|....|
PF14_0201           GAAAAATTCT TGTTGAAAAA TTAAGAATCT TATATTTAGA AAAGAATAAC TTATTTAATA CACAAGCTAG TTGTATCAAA
3D7                 .......... .......... .......... .......... .......... .......... .......... ..........
RO33                .......... .......... .......... .......... .......... .......... .......... ..........
Palo Alto           .......... .......... .......... .......... .......... .......... .......... ..........
FCR3                .......... .......... .......... .......... .......... .......... .......... ..........
Wellcome            .......... .......... .......... .......... .......... .......... .......... ..........
D6                  .......... .......... .......... .......... .......... .......... .......... ..........
T996                .......... .......... .......... .......... .......... .......... .......... ..........
T9102               .......... .......... .......... .......... .......... .......... .......... ..........
K1                  .......... .......... .......... .......... .......... .......... .......... ..........
Dd2                 .......... .......... .......... .......... .......... .......... .......... ..........
D10                 .......... .......... .......... .......... .......... .......... .......... ..........
FCC2                .......... .......... .......... .......... .......... .......... .......... ..........
HB3                 .......... .......... .......... .......... .......... .......... .......... ..........
7G8                 .......... .......... .......... .......... .......... .......... .......... ..........
P.reichenowi        .......... ......G... ........T. .......... .......... .......... .......... ..........

                         1690       1700       1710       1720       1730       1740       1750       1760
                    ....|....| ....|....| ....|....| ....|....| ....|....| ....|....| ....|....| ....|....|
PF14_0201           TCGTATTGTT ACAAGAACCC ATTGAATCTA AAAACACTAG AAGTATTATT AAAGAAAAAT TATTATAGAT TAAAAGAAAA
3D7                 .......... .......... .......... .......... .......... .......... .......... ..........
RO33                .......... .......... .......... .......... .......... .......... .......... ..........
Palo Alto           .......... .......... .......... .......... .......... .......... .......... ..........
FCR3                .......... .......... .......... .......... .......... .......... .......... ..........
Wellcome            .......... .......... .......... .......... .......... .......... .......... ..........
D6                  .......... .......... .......... .......... .......... .......... .......... ..........
T996                .......... .......... .......... .......... .......... .......... .......... ..........
T9102               .......... .......... .......... .......... .......... .......... .......... ..........
K1                  .......... .......... .......... .......... .......... .......... .......... ..........
Dd2                 .......... .......... .......... .......... .......... .......... .......... ..........
D10                 .......... .......... .......... .......... .......... .......... .......... ..........
FCC2                .......... .......... .......... .......... .......... .......... .......... ..........
HB3                 .......... .......... .......... .......... .......... .......... .......... ..........
7G8                 .......... .......... .......... .......... .......... .......... .......... ..........
P.reichenowi        .......... ....A..... .......... .G........ .......... .......... .......... ..........
```

```
                     1770      1780      1790      1800      1810      1820      1830      1840
                ....|....| ....|....| ....|....| ....|....| ....|....| ....|....| ....|....| ....|....|
PF14_0201       TAAAGATTAT GATGTTGTAT CAAGTATTAT ACAACATCTT GATAATGTCG ATGCAAACAA GAAAAAAAAA TGGCTTACAC
3D7             .......... .......... .......... .......... .......... .......... .......... ..........
RO33            .......... .......... .......... .......... .......... .......... .......... ..........
Palo Alto       .......... .......... .......... .......... .......... .......... .......... ..........
FCR3            .......... .......... .......... .......... .......... .......... .......... ..........
Wellcome        .......... .......... .......... .......... .......... .......... .......... ..........
D6              .......... .......... .......... .......... .......... .......... .......... ..........
T996            .......... .......... .......... .......... .......... .......... .......... ..........
T9102           .......... .......... .......... .......... .......... .......... .......... ..........
K1              .......... .......... .......... .......... .......... .......... .......... ..........
Dd2             .......... .......... .......... .......... .......... .......... .......... ..........
D10             .......... .......... .......... .......... .......... .......... .......... ..........
FCC2            .......... .......... .......... .......... .......... .......... .......... ..........
HB3             .......... .......... .......... .......... .......... .......... .......... ..........
7G8             .......... .......... .......... .......... .......... .......... .......... ..........
P.reichenowi    .......... .......... .......... .......... ........T. .......... ......T... ..........

                     1850      1860      1870      1880      1890      1900      1910      1920
                ....|....| ....|....| ....|....| ....|....| ....|....| ....|....| ....|....| ....|....|
PF14_0201       ATGAAAGAAT ACTTAAAAAA TTACAAGTAC TTATAGCTGA AGGATATAAA AGAATAAACG AAAAAGAAAA AGATATCGAT
3D7             .......... .......... .......... .......... .......... .......... .......... ..........
RO33            .......... .......... .......... .......... .......... .......... .......... ..........
Palo Alto       .......... .......... .......... .......... .......... .......... .......... ..........
FCR3            .......... .......... .......... .......... .......... .......... .......... ..........
Wellcome        .......... .......... .......... .......... .......... .......... .......... ..........
D6              .......... .......... .......... .......... .......... .......... .......... ..........
T996            .......... .......... .......... .......... .......... .......... .......... ..........
T9102           .......... .......... .......... .......... .......... .......... .......... ..........
K1              .......... .......... .......... .......... .......... .......... .......... ..........
Dd2             .......... .......... .......... .......... .......... .......... .......... ..........
D10             .......... .......... .......... .......... .......... .......... .......... ..........
FCC2            .......... .......... .......... .......... .......... .......... .......... ..........
HB3             .......... .......... .......... .......... .......... .......... .......... ..........
7G8             .......... .......... .......... .......... .......... .......... .......... ..........
P.reichenowi    ..A....... .......... .......... .......... .......... .......... .......... ......T...

                     1930      1940      1950      1960      1970      1980      1990      2000
                ....|....| ....|....| ....|....| ....|....| ....|....| ....|....| ....|....| ....|....|
PF14_0201       AGAAGAATGG CTGTATATAA TGCCCTATAT GAAAAAGCAC AATCTTATAA TTTACAAAAA TTATTTAATG ATAGTAATGA
3D7             .......... .......... .......... .......... .......... .......... .......... ..........
RO33            .......... .......... .......... .......... .......... .......... .......... ..........
Palo Alto       .......... .......... .......... .......... .......... .......... .......... ..........
FCR3            .......... .......... .......... .......... .......... .......... .......... ..........
Wellcome        .......... .......... .......... .......... .......... .......... .......... ..........
D6              .......... .......... .......... .......... .......... .......... .......... ..........
T996            .......... .......... .......... .......... .......... .......... .......... ..........
T9102           .......... .......... .......... .......... .......... .......... .......... ..........
K1              .......... .......... .......... .......... .......... .......... .......... ..........
Dd2             .......... .......... .......... .......... .......... .......... .......... ..........
D10             .......... .......... .......... .......... .......... .......... .......... ..........
FCC2            .......... .......... .......... .......... .......... .......... .......... ..........
HB3             .......... .......... .......... .......... .......... .......... .......... ..........
7G8             .......... .......... .......... .......... .......... .......... .......... ..........
P.reichenowi    .......... .C........ ...T...... .......... .......... .......... .......... ..........

                     2010      2020      2030      2040      2050      2060      2070      2080
                ....|....| ....|....| ....|....| ....|....| ....|....| ....|....| ....|....| ....|....|
PF14_0201       TTTCTTAAAG AAATATGCAA TAATGGGTAA TTCTTTTGAT GATGGAGATG AAGTATTCGG AAGCCAATCA TCCAACTTTA
3D7             .......... .......... .......... .......... .......... .......... .......... ..........
RO33            .......... .......... .......... .......... .......... .......... .......... ..........
Palo Alto       .......... .......... .......... .......... .......... .......... .......... ..........
FCR3            .......... .......... .......... .......... .......... .......... .......... ..........
Wellcome        .......... .......... .......... .......... .......... .......... .......... ..........
D6              .......... .......... .......... .......... .......... .......... .......... ..........
T996            .......... .......... .......... .......... .......... .......... .......... ..........
T9102           .......... .......... .......... .......... .......... .......... .......... ..........
K1              .......... .......... .......... .......... .......... .......... .......... ..........
Dd2             .......... .......... .......... .......... .......... .......... .......... ..........
D10             .......... .......... .......... .......... .......... .......... .......... ..........
FCC2            .......... .......... .......... .......... .......... .......... .......... ..........
HB3             .......... .......... .......... .......... .......... .......... .......... ..........
7G8             .......... .......... .......... .......... .......... .......... .......... ..........
P.reichenowi    .......... ........T. .......... .......... .......... .......... .......... ..........
```

```
                          2090       2100       2110       2120       2130       2140       2150       2160
                    ....|....| ....|....| ....|....| ....|....| ....|....| ....|....| ....|....| ....|....|
PF14_0201           ATATTTTTGA TAGTAATAAC ---ACTGATC AAAATAATGA ACAAGAACAA CCAAAACAAG ACGATCAATT ACTAAATAAT
3D7                 .......... .......... ---....... .......... .......... .......... .......... ..........
RO33                .......... .......... ---....... .......... .......... .......... .......... ..........
Palo Alto           .......... .......... ---....... .......... .......... .......... .......... ..........
FCR3                .......... .......... ---....... .......... .......... .......... .......... ..........
Wellcome            .......... .......... ---....... .......... .......... .......... .......... ..........
D6                  .......... .......... ---....... .......... .......... .......... .......... ..........
T996                .......... .......... ---....... .......... .......... .......... .......... ..........
T9102               .......... .......... ---....... .......... .......... .......... .......... ..........
K1                  .......... .......... ---....... .......... .......... .......... .......... ..........
Dd2                 .......... .......... ---....... .......... .......... .......... .......... ..........
D10                 .......... .......... ---....... .......... .......... .......... .......... ..........
FCC2                .......... .......... ---....... .......... .......... .......... .......... ..........
HB3                 .......... .......... ---....... .......... .......... .......... .......... ..........
7G8                 .......... .......... ---....... .......... .......... .......... .......... ..........
P.reichenowi        .......... .........T AAC....... .T........ .......... ....C..... .....A..C. .G........

                          2170       2180       2190       2200       2210       2220       2230       2240
                    ....|....| ....|....| ....|....| ....|....| ....|....| ....|....| ....|....| ....|....|
PF14_0201           AATAATGATG ATGTCCTATC AGAATCCAAT AACGAAAATA AAGAGAAAAC AAGCGACGAT GCAACTCACA AAGAGACACA
3D7                 .......... .......... .......... .......... .......... .......... .......... ..........
RO33                .......... .......... .......... .......... .......... .......... .......... ..........
Palo Alto           .......... .......... .......... .......... .......... .......... .......... ..........
FCR3                .......... .......... .......... .......... .......... .......... .......... ..........
Wellcome            .......... .......... .......... .......... .......... .......... .......... ..........
D6                  .......... .......... .......... .......... .......... .......... .......... ..........
T996                .......... .......... .......... .......... .......... .......... .......... ..........
T9102               .......... .......... .......... .......... .......... .......... .......... ..........
K1                  .......... .......... .......... .......... .......... .......... .......... ..........
Dd2                 .......... .......... .......... .......... .......... .......... .......... ..........
D10                 .......... .......... .......... .......... .......... .......... .......... ..........
FCC2                .......... .......... .......... .......... .......... .......... .......... ..........
HB3                 .......... .......... .......... .......... .......... .......... .......... ..........
7G8                 .......... .......... .......... .......... .......... .......... .......... ..........
P.reichenowi        .......... .....G.... .......... .......... ....T..... .......... .......... ........G.

                          2250       2260       2270       2280       2290       2300       2310       2320
                    ....|....| ....|....| ....|....| ....|....| ....|....| ....|....| ....|....| ....|....|
PF14_0201           AGAAAAATCA GATCAAGAAC CTTCACAAAA TATTCAAGAA GATAATAGTG ATGAAAAA-- -CATGCAGAA AATGAGGAAA
3D7                 .......... .......... .......... .......... .......... ........-- -......... ..........
RO33                .......... .......... .......... .......... .......... ........-- -......... ..........
Palo Alto           .......... .......... .......... .......... .......... ........-- -......... ..........
FCR3                .......... .......... .......... .......... .......... ........-- -......... ..........
Wellcome            .......... .......... .......... .......... .......... ........-- -......... ..........
D6                  .......... .......... .......... .......... .......... ........-- -......... ..........
T996                .......... .......... .......... .......... .......... ........-- -......... ..........
T9102               .......... .......... .......... .......... .......... ........-- -......... ..........
K1                  .......... .......... .......... .......... .......... ........-- -......... ..........
Dd2                 .......... .......... .......... .......... .......... ........-- -......... ..........
D10                 .......... .......... .......... .......... .......... ........-- -......... ..........
FCC2                .......... .......... .......... .......... .......... ........-- -......... ..........
HB3                 .......... .......... .......... .......... .......... ........-- -......... ..........
7G8                 .......... .......... .......... .......... .......... ........-- -......... ..........
P.reichenowi        .......... .......... .....G.... .......... .......... ........AA A...CA.... .....C....

                          2330       2340       2350       2360       2370       2380       2390       2400
                    ....|....| ....|....| ....|....| ....|....| ....|....| ....|....| ....|....| ....|....|
PF14_0201           ATGTAGAACA AATTGAAACA GACAGTAACG TTTCTGAAGA AGCTAATGAC GAAAATAAAG ACAATATGCA AACAACAACT
3D7                 .......... .......... .......... .......... .......... .......... .......... ..........
RO33                .......... .......... .......... .......... .......... .......... .......... ..........
Palo Alto           .......... .......... .......... .......... .......... .......... .......... ..........
FCR3                .......... .......... .......... .......... .......... .......... .......... ..........
Wellcome            .......... .......... .......... .......... .......... .......... .......... ..........
D6                  .......... .......... .......... .......... .......... .......... .......... ..........
T996                .......... .......... .......... .......... .......... .......... .......... ..........
T9102               .......... .......... .......... .......... .......... .......... .......... ..........
K1                  .......... .......... .......... .......... .......... .......... .......... ..........
Dd2                 .......... .......... .......... .......... .......... .......... .......... ..........
D10                 .......... .......... .......... .......... .......... .......... .......... ..........
FCC2                .......... .......... .......... .......... .......... .......... .......... ..........
HB3                 .......... .......... .......... .......... .......... .......... .......... ..........
7G8                 .......... .......... .......... .......... .......... .......... .......... ..........
P.reichenowi        .........C .......... .C........ .......... .......... .......... .......... ..........
```

```
                     2410       2420       2430       2440       2450       2460       2470       2480
                ....|....| ....|....| ....|....| ....|....| ....|....| ....|....| ....|....| ....|....|
PF14_0201       GATGAAGGAA CTGAAGAATT ACAACAAAAT GATGAAGATG CTGAATCTCT AACAAAGGAA AATTCAAAAT CGGAAGAACA
3D7             .......... .......... .......... .......... .......... .......... .......... ..........
RO33            .......... .......... .......... .......... .......... .......... .......... ..........
Palo Alto       .......... .......... .......... .......... .......... .......... .......... ..........
FCR3            .......... .......... .......... .......... .......... .......... .......... ..........
Wellcome        .......... .......... .......... .......... .......... .......... .......... ..........
D6              .......... .......... .......... .......... .......... .......... .......... ..........
T996            .......... .......... .......... .......... .......... .......... .......... ..........
T9102           .......... .......... .......... .......... .......... .......... .......... ..........
K1              .......... .......... .......... .......... .......... .......... .......... ..........
Dd2             .......... .......... .......... .......... .......... .......... .......... ..........
D10             .......... .......... .......... .......... .......... .......... .......... ..........
FCC2            .......... .......... .......... .......... .......... .......... .......... ..........
HB3             .......... .......... .......... .......... .......... .......... .......... ..........
7G8             .......... .......... .......... .......... .......... .......... .......... ..........
P.reichenowi    .......... G......... .......... .......... .......G.A .......... ....T..... ..........

                     2490       2500       2510       2520       2530       2540       2550       2560
                ....|....| ....|....| ....|....| ....|....| ....|....| ....|....| ....|....| ....|....|
PF14_0201       AGAAAATGAA GATTCAACAG ATGCTGAAGC TATTGACAAA GAAGAAGTAG AAACAGAAGA AAAAGGAAAA GATGAACAAA
3D7             .......... .......... .......... .......... .......... .......... .......... ..........
RO33            .......... .......... .......... .......... .......... .......... .......... ..........
Palo Alto       .......... .......... .......... .......... .......... .......... .......... ..........
FCR3            .......... .......... .......... .......... .......... .......... .......... ..........
Wellcome        .......... .......... .......... .......... .......... .......... .......... ..........
D6              .......... .......... .......... .......... .......... .......... .......... ..........
T996            .......... .......... .......... .......... .......... .......... .......... ..........
T9102           .......... .......... .......... .......... .......... .......... .......... ..........
K1              .......... .......... .......... .......... .......... .......... .......... ..........
Dd2             .......... .......... .......... .......... .......... .......... .......... ..........
D10             .......... .......... .......... .......... .......... .......... .......... ..........
FCC2            .......... .......... .......... .......... .......... .......... .......... ..........
HB3             .......... .......... .......... .......... .......... .......... .......... ..........
7G8             .......... .......... .......... .......... .......... .......... .......... ..........
P.reichenowi    .......... .......... .......... ......G... .......... .......... .....A.... ..A.......

                     2570       2580       2590       2600       2610       2620       2630       2640
                ....|....| ....|....| ....|....| ....|....| ....|....| ....|....| ....|....| ....|....|
PF14_0201       AA-------- -AAAGACGAA CAAAAAGAAC AAGATGAAGA AGAAGATGGC GAAAAAGAAA ATAAACATAA ATCAAGTGAA
3D7             ..-------- -......... .......... .......... .......... .......... .......... ..........
RO33            ..-------- -......... .......... .......... .......... .......... .......... ..........
Palo Alto       ..-------- -......... .......... .......... .......... .......... .......... ..........
FCR3            ..-------- -......... .......... .......... .......... .......... .......... ..........
Wellcome        ..-------- -......... .......... .......... .......... .......... .......... ..........
D6              ..-------- -......... .......... .......... .......... ....C..... .......... ..........
T996            ..-------- -......... .......... .......... .......... .......... .......... ..........
T9102           ..-------- -......... .......... .......... .......... .......... .......... ..........
K1              ..-------- -......... .......... .......... .......... .......... .......... ..........
Dd2             ..-------- -......... .......... .......... .......... .......... .......... ..........
D10             ..-------- -......... .......... .......... .......... .......... .......... ..........
FCC2            ..-------- -......... .......... .......... .......... .......... .......... ..........
HB3             ..-------- -......... .......... .......... .......... .......... .......... ..........
7G8             ..-------- -......... .......... .......... .......... .......... .......... ..........
P.reichenowi    ..GACGAACA A......... .......... .......... ........A. .......... .....G.... ..........

                     2650       2660       2670       2680       2690       2700       2710       2720
                ....|....| ....|....| ....|....| ....|....| ....|....| ....|....| ....|....| ....|....|
PF14_0201       ACAACCAACG AAACAGTAAC TGACATTGAA GAAAATAAAA ATGAAGTAAA AGGTGAAGAA CACCTACAAG GATCAGAACA
3D7             .......... .......... .......... .......... .......... .......... .......... ..........
RO33            .......... .......... .......... .......... .......... .......... .......... ..........
Palo Alto       .......... .......... .......... .......... .......... .......... ........G. ..........
FCR3            .......... .......... .......... .......... .......... .......... .......... ..........
Wellcome        .......... .......... .......... .......... .......... .......... .......... ..........
D6              .......... .......... .......... .......... .......... .......... .......... ..........
T996            .......... .......... .......... .......... .......... .......... .......... ..........
T9102           .......... .......... .......... .......... .......... .......... .......... ..........
K1              .......... .......... .......... .......... .......... .......... .......... ..........
Dd2             .......... .......... .......... .......... .......... .......... .......... ..........
D10             .......... .......... .......... .......... .......... .......... .......... ..........
FCC2            .......... .......... .......... .......... .......... .......... .......... ..........
HB3             .......... .......... .......... .......... .......... .......... .......... ..........
7G8             .......... .......... .......... .......... .......... .......... .......... ..........
P.reichenowi    .......... .......... .A..C..... .......... .......... .......... .......... C.........
```

```
                          2730       2740       2750       2760       2770       2780       2790       2800
                    ....|....| ....|....| ....|....| ....|....| ....|....| ....|....| ....|....| ....|....|
PF14_0201           ATCAATAGAA GCATCTGAAT CATCTCAAAA AGATGAAACT AAAGAAACAG AAGATAAAGA AGAATATGTA AATGCAAATG
3D7                 .......... .......... .......... .......... .......... .......... .......... ..........
RO33                .......... .......... .......... .......... .......... .......... .......... ..........
Palo Alto           .......... .......... .......... .......... .......... .......... .......... ..........
FCR3                .......... .......... .......... .......... .......... .......... .......... ..........
Wellcome            .......... .......... .......... .......... .......... .......... .......... ..........
D6                  .......... .......... .......... .......... .......... .......... .......... ..........
T996                .......... .......... .......... .......... .......... .......... .......... ..........
T9102               .......... .......... .......... .......... .......... .......... .......... ..........
K1                  .......... .......... .......... .......... .......... .......... .......... ..........
Dd2                 .......... .......... .......... .......... .......... .......... .......... ..........
D10                 .......... .......... .......... .......... .......... .......... .......... ..........
FCC2                .......... .......... .......... .......... .......... .......... .......... ..........
HB3                 .......... .......... .......... .......... .......... .......... .......... ..........
7G8                 ....G..... .......... .......... .......... .......... .......... .......... ..........
P.reichenowi        .G.....C.. ........T. ....C..... ......T.A. .......... .......... .......... ..........

                          2810       2820       2830       2840       2850       2860       2870       2880
                    ....|....| ....|....| ....|....| ....|....| ....|....| ....|....| ....|....| ....|....|
PF14_0201           ATGATGAATC TAGTGAAGAA GATACGACTC CAAATGAAAC AAACAAAACT GATAATGGCA GTTCATTTTT CTTTGCTATG
3D7                 .......... .......... .......... .......... .......... .......... .......... ..........
RO33                .......... .......... .......... .......... .......... .......... .......... ..........
Palo Alto           .......... .......... .......... .......... .......... .......... .......... ..........
FCR3                .......... .......... .......... .......... .......... .......... .......... ..........
Wellcome            .......... .......... .......... .......... .......... .......... .......... ..........
D6                  .......... .......... .......... .......... .......... .......... .......... ..........
T996                .......... .......... .......... .......... .......... .......... .......... ..........
T9102               .......... .......... .......... .......... .......... .......... .......... ..........
K1                  .......... .......... .......... .......... .......... .......... .......... ..........
Dd2                 .......... .......... .......... .......... .......... .......... .......... ..........
D10                 .......... .......... .......... .......... .......... .......... .......... ..........
FCC2                .......... .......... .......... .......... .......... .......... .......... ..........
HB3                 .......... .......... .......... .......... .......... .......... .......... ..........
7G8                 .......... .......... .......... .......... .......... .......... .......... ..........
P.reichenowi        .......... .......... .........A .......... .......... .T........ .......... ..........

                          2890       2900       2910       2920       2930
                    ....|....| ....|....| ....|....| ....|....| ....|....| .
PF14_0201           AGTAATGCAC TCTTAGTAAT TTTACTTTTA TTATTTATAG AATTCCTATA A
3D7
RO33
Palo Alto
FCR3
Wellcome
D6
T996
T9102
K1
Dd2
D10
FCC2
HB3
7G8
P.reichenowi
```

PFF0995c

```
                         10         20         30         40         50         60         70         80
                 ....|....| ....|....| ....|....| ....|....| ....|....| ....|....| ....|....| ....|....|
PFF0995c         ATGATGTTTT TTAAGTGTAA TCAGGTTTTT ACCTTGGTTT TTTTGTTGCT CTTATATTTT AACAATATTG TCTACACCCA
3D7              .......... .......... .......... .......... .......... .......... .......... ..........
RO33             .......... .......... .......... .......... .......... .......... .......... ..........
Palo Alto        .......... .......... .......... .......... .......... .......... .......... ..........
FCR3             .......... .......... .......... .......... .......... .......... .......... ..........
Wellcome         .......... .......... .......... .......... .......... .......... .......... ..........
D6               .......... .......... .......... .......... .......... .......... .......... ..........
T996             .......... .......... .......... .......... .......... .......... .......... ..........
T9102            .......... .......... .......... .......... .......... .......... .......... ..........
K1               .......... .......... .......... .......... .......... .......... .......... ..........
Dd2              .......... .......... .......... .......... .......... .......... .......... ..........
D10              .......... .......... .......... .......... .......... .......... .......... ..........
FCC2             .......... .......... .......... .......... .......... .......... .......... ..........
HB3              .......... .......... .......... .......... .......... .......... .......... ..........
7G8              .......... .......... .......... .......... .......... .......... .......... ..........
P.reichenowi     .......... .......... .A.T...... .......... .......... .......... .......... ..........

                         90        100        110        120        130        140        150        160
                 ....|....| ....|....| ....|....| ....|....| ....|....| ....|....| ....|....| ....|....|
PFF0995c         TGTAGATGAT ATAAAAAATA CATCCCAGAA GAAAATTACA TATGATAAAT ATAATAAAAA TAAAGAAAAT ATGAATAATG
3D7              .......... .......... .......... .......... .......... .......... .......... ..........
RO33             .......... .......... .......... .......... .......... .......... .......... ..........
Palo Alto        .......... .......... .......... .......... .......... .......... .......... ..........
FCR3             .......... .......... .......... .......... .......... .......... .......... ..........
Wellcome         .......... .......... .......... .......... .......... .......... .......... ..........
D6               .......... .......... .......... .......... .......... .......... .......... ..........
T996             .......... .......... .......... .......... .......... .......... .......... ..........
T9102            .......... .......... .......... .......... .......... .......... .......... ..........
K1               .......... .......... .......... .......... .......... .......... .......... ..........
Dd2              .......... .......... .......... .......... .......... .......... .......... ..........
D10              .......... .......... .......... .......... .......... .......... .......... ..........
FCC2             .......... .......... .......... .......... .......... .......... .......... ..........
HB3              .......... .......... .......... .......... .......... .......... .......... ..........
7G8              .......... .......... .......... .......... .......... .......... .......... ..........
P.reichenowi     .......... ......G... .......... .......... .......... ......C... .......... ..........

                        170        180        190        200        210        220        230        240
                 ....|....| ....|....| ....|....| ....|....| ....|....| ....|....| ....|....| ....|....|
PFF0995c         AAAAAAATGA TAATAAAGAT AATAAAGATA ATATTTATAA TGATAATATT AATAATGATA ATATTAATAA TGATAATATT
3D7              .......... .......... .......... .......... .......... .......... .......... ..........
RO33             .......... .......... .......... .......... .......... .......... .......... .---------
Palo Alto        .......... .......... ...------- --........ .......... .......... .......... .---------
FCR3             .......... .......... .......... .......... .......... ......---- ---------- ----------
Wellcome         .......... .......... .......... .......... .......... ......---- ---------- ----------
D6               .......... .......... .......... .......... .......... .......... .......... .---------
T996             .......... .......... ...------- --........ .......... .......... .......... .---------
T9102            .......... .......... .......... .......... .......... .......... .......... .---------
K1               .......... .......... .......... .......... .......... .......... .......... .---------
Dd2              .......... .......... .......... .......... .......... ......---- ---------- ----------
D10              .......... .......... ...------- --........ .......... .......... .......... .---------
FCC2             .......... .......... .......... .......... .......... ......---- ---------- ----------
HB3              .......... .......... .......... .......... .......... .......... .......... .---------
7G8              .......... .......... .......... .......... .......... ......---- ---------- ----------
P.reichenowi     .......... .......--- ------.... G......... .A......A. ------.... .......... G---------

                        250        260        270        280        290        300        310        320
                 ....|....| ....|....| ....|....| ....|....| ....|....| ....|....| ....|....| ....|....|
PFF0995c         AATAATGAAG ATGAGTATAA ATTTTTATCT ATGAAACATT ATAAAGATAG TTTATCAAAT AAATTAAATA ATGAAAATGA
3D7              .......... .......... .......... .......... .......... .......... .......... ..........
RO33             ------.... .......... .......... .......... .......... .......... .......... ..........
Palo Alto        ------.... .......... .......... .......... .......... .......... .......... ..........
FCR3             ------.... .......... .......... .......... .......... .......... .......... ..........
Wellcome         ------.... .......... .......... .......... .......... .......... .......... ..........
D6               ------.... .......... .......... .......... .......... .......... .......... ..........
T996             ------.... .......... .......... .......... .......... .......... .......... ..........
T9102            ------.... .......... .......... .......... .......... .......... .......... ..........
K1               ------.... .......... .......... .......... .......... .......... .......... ..........
Dd2              ------.... .......... .......... .......... .......... .......... .......... ..........
D10              ------.... .......... .......... .......... .......... .......... .......... ..........
FCC2             ------.... .......... .......... .......... .......... .......... .......... ..........
HB3              ------.... .......... .......... .......... .......... .......... .......... ..........
7G8              ------.... .......... .......... .......... .......... .......... .......... ..........
P.reichenowi     ------.... .......... .......... ..A...A... ...GG....A .......... .......... ..........
```

```
                               330        340        350        360        370        380        390        400
                        ....|....| ....|....| ....|....| ....|....| ....|....| ....|....| ....|....| ....|....|
PFF0995c                TCATATGAAC TATTTAATAA GAAAAAGAAA AGATAATACT CAAGGAAGTC AACATTTCAA TGAAAATATT GAAAATAATG
3D7                     .......... .......... .......... .......... .......... .......... .......... ..........
RO33                    .......... .......... .......... .......... .......... .......... .......... ..........
Palo Alto               .......... .......... .......... .......... .......... .......... .......... ..........
FCR3                    .......... .......... .......... .......... .......... .......... .......... ..........
Wellcome                .......... .......... .......... .......... .......... .......... .......... ..........
D6                      .......... .......... .......... .......... .......... .......... .......... ..........
T996                    .......... .......... .......... .......... .......... .......... .......... ..........
T9102                   .......... .......... .......... .......... .......... .......... .......... ..........
K1                      .......... .......... .......... .......... .......... .......... .......... ..........
Dd2                     .......... .......... .......... .......... .......... .......... .......... ..........
D10                     .......... .......... .......... .......... .......... .......... .......... ..........
FCC2                    .......... .......... .......... .......... .......... .......... .......... ..........
HB3                     .......... .......... .......... .......... .......... .......... .......... ..........
7G8                     .......... .......... .......... .......... .......... .......... .......... ..........
P.reichenowi            .......... .......... .......... .......G.. .......... ........-- ---------- ----------

                               410        420        430        440        450        460        470        480
                        ....|....| ....|....| ....|....| ....|....| ....|....| ....|....| ....|....| ....|....|
PFF0995c                AAAATGTTGA AAATAATGAA AATATTGAAA ATAATGAAAA TAATGAAAAT ATTGAAAATA TTGAAAATAA TGAAAATAAT
3D7                     .......... .......... .......... .......... .......... .......... .......... ..........
RO33                    ......C... .......... .......... .......... .......... .......... .......... ..........
Palo Alto               .......... .......... .......... .......... .......... ...------- --........ ..........
FCR3                    .......... .......... .......... .......... .......... .......... .......... ..........
Wellcome                .......... .......... .......... .......... .......... .......... .......... ..........
D6                      .......... .......... .......... .......... .......... .......... .......... ..........
T996                    .......... .......... .......... .......... .......... .......... .......... ..........
T9102                   ......C... .......... .......... .......... .......... .......... .......... ..........
K1                      .......... .......... .......... .......... .......... .......... .......... ..........
Dd2                     .......... .......... .......... .......... .......... .......... .......... ..........
D10                     .......... .......... .......... .......... .......... .......... .......... ..........
FCC2                    .......... .......... .......... .......... .......... .......... .......... ..........
HB3                     ......C... .......... .......... .......... .......... .......... .......... ..........
7G8                     .......... .......... .......... .......... .......... .......... .......... ..........
P.reichenowi            ---------- ---------- ---------- ---------- ---------- ---A.TG.A. A.A.TG.A.. .A.TG.A...

                               490        500        510        520        530        540        550        560
                        ....|....| ....|....| ....|....| ....|....| ....|....| ....|....| ....|....| ....|....|
PFF0995c                GAAAATAATG AAAATATTGA AAATAATGAA AATAATGAAA ATAATG---- -----AAAAT TCATCTATCA TGAATTCAGA
3D7                     .......... .......... .......... .......... ......---- -----..... .......... ..........
RO33                    .......... .......... .......... .......... ......---- -----..... .......... ..........
Palo Alto               .......... .......... .......... .......... ......---- -----..... .......... ..........
FCR3                    .......... .......... .......... .......... ......---- -----..... .......... ..........
Wellcome                .......... .......... .......... .......... ......---- -----..... .......... ..........
D6                      .......... ......A... .....T.... .......... ......AAAA TAATG..... .......... ..........
T996                    .......... .......... .......... .......... ......AAAA TAATG..... .......... ..........
T9102                   .......... ......A... .......... .......... ......---- -----..... .......... ..........
K1                      .......... .......... .......... .......... ......---- -----..... .......... ..........
Dd2                     .......... .......... .......... .......... ......---- -----..... .......... ..........
D10                     .......... .......... .......... .......... ......---- -----..... .......... ..........
FCC2                    .......... .......... .......... .......... ......---- -----..... .......... ..........
HB3                     .......... .......... .......... .......... ......---- -----..... .......... ..........
7G8                     .......... .......... .......... .......... ......---- -----..... .......... ..........
P.reichenowi            A.TG.A...A .TG.A.A.A. TG.A...A.T G.A...A.TG .A...----- ---------- .......... ..........

                               570        580        590        600        610        620        630        640
                        ....|....| ....|....| ....|....| ....|....| ....|....| ....|....| ....|....| ....|....|
PFF0995c                ATCATATAAT AATATTATCA ATTCTAATGA ACATAATGAA GAACAAATAA AAAAAAAAGA AGAAGATTTA ATTGAAGCAT
3D7                     .......... .......... .......... .......... .......... .......... .......... ..........
RO33                    .......... .......... .......... .......... .......... .......... .......... ..........
Palo Alto               .......... .......... .......... .......... .......... .......... .......... ..........
FCR3                    .......... .......... .......... .......... .......... .......... .......... ..........
Wellcome                .......... .......... .......... .......... .......... .......... .......... ..........
D6                      .......... .......... .......... .......... .......... .......... .......... ..........
T996                    .......... .......... .......... .......... .......... .......... .......... ..........
T9102                   .......... .......... .......... .......... .......... .......... .......... ..........
K1                      .......... .......... .......... .......... .......... .......... .......... ..........
Dd2                     .......... .......... .......... .......... .......... .......... .......... ..........
D10                     .......... .......... .......... .......... .......... .......... .......... ..........
FCC2                    .......... .......... .......... .......... .......... .......... .......... ..........
HB3                     .......... .......... .......... .......... .......... .......... .......... ..........
7G8                     .......... .......... .......... .......... .......... .......... .......... ..........
P.reichenowi            .......... .......... .......... .......... .......... .......... .......... ..........
```

```
                               650        660        670        680        690        700        710        720
                      ....|....| ....|....| ....|....| ....|....| ....|....| ....|....| ....|....| ....|....|
PFF0995c              TTTTCCCATT TATATTAAAA AAATTAGATA ACGAATCATT ATCTCTTGAT AATAAATATG ATGATTATTA TAATTTACCA
3D7                   .......... .......... .......... .......... .......... .......... .......... ..........
RO33                  .......... .......... .......... .......... .......... .......... .......... ..........
Palo Alto             .......... .......... .......... .......... .......... .......... .......... ..........
FCR3                  .......... .......... .......... .......... .......... .......... .......... ..........
Wellcome              .......... .......... .......... .......... .......... .......... .......... ..........
D6                    .......... .......... .......... .......... .......... .......... .......... ..........
T996                  .......... .......... .......... .......... .......... .......... .......... ..........
T9102                 .......... .......... .......... .......... .......... .......... .......... ..........
K1                    .......... .......... .......... .......... .......... .......... .......... ..........
Dd2                   .......... .......... .......... .......... .......... .......... .......... ..........
D10                   .......... .......... .......... .......... .......... .......... .......... ..........
FCC2                  .......... .......... .......... .......... .......... .......... .......... ..........
HB3                   .......... .......... .......... .......... .......... .......... .......... ..........
7G8                   .......... .......... .......... .......... .......... .......... .......... ..........
P.reichenowi          .......T.. .......... ......A... .......... ....G..... C.......C. ........G. ..........

                               730        740        750        760        770        780        790        800
                      ....|....| ....|....| ....|....| ....|....| ....|....| ....|....| ....|....| ....|....|
PFF0995c              AATGATCATA ATGACACACA TAAAGAAAAT TCAAGTGATC ATAATTTATT AGGATATAAA TTGGGTAACA ATCTAAAATC
3D7                   .......... .......... .......... .......... .......... .......... .......... ..........
RO33                  .......... .......... .......... .......... .......... .......... .......... ..........
Palo Alto             .......... .......... .......... .......... .......... .......... .......... ..........
FCR3                  .......... .......... .......... .......... .......... .......... .......... ..........
Wellcome              .......... .......... .......... .......... .......... .......... .......... ..........
D6                    .......... .......... .......... .......... .......... .......... .......... ..........
T996                  .......... .......... .......... .......... .......... .......... .......... ..........
T9102                 .......... .......... .......... .......... .......... .......... .......... ..........
K1                    .......... .......... .......... .......... .......... .......... .......... ..........
Dd2                   .......... .......... .......... .......... .......... .......... .......... ..........
D10                   .......... .......... .......... .......... .......... .......... .......... ..........
FCC2                  .......... .......... .......... .......... .......... .......... .......... ..........
HB3                   .......... .......... .......... .......... .......... .......... .......... ..........
7G8                   .......... .......... .......... .......... .......... .......... .......... ..........
P.reichenowi          .G........ .......... .......... .......... .......... ..A....... ........T. .C........

                               810        820        830        840        850        860        870        880
                      ....|....| ....|....| ....|....| ....|....| ....|....| ....|....| ....|....| ....|....|
PFF0995c              ATATTTGATA GAAGAAAATG ATGTTTCTCA AAAAAAAACT GATGACATAA ATGAAAGTGC AAGTAGTGAC TCTGAAAACA
3D7                   .......... .......... .......... .......... .......... .......... .......... ..........
RO33                  .......... .......... .......... .......... .......... .......... .......... ..........
Palo Alto             .......... .......... .......... .......... .......... .......... .......... ..........
FCR3                  .......... .......... .......... .......... .......... .......... .......... ..........
Wellcome              .......... .......... .......... .......... .......... .......... .......... ..........
D6                    .......... .......... .......... .......... .......... .......... .......... ..........
T996                  .......... .......... .......... .......... .......... .......... .......... ..........
T9102                 .......... .......... .......... .......... .......... .......... .......... ..........
K1                    .......... .......... .......... .......... .......... .......... .......... ..........
Dd2                   .......... .......... .......... .......... .......... .......... .......... ..........
D10                   .......... .......... .......... .......... .......... .......... .......... ..........
FCC2                  .......... .......... .......... .......... .......... .......... .......... ..........
HB3                   .......... .......... .......... .......... .......... .......... .......... ..........
7G8                   .......... .......... .......... .......... .......... .......... .......... ..........
P.reichenowi          .......G.. .......... .....C.... ..C..C.... .......... .......C.A .......... ......G...

                               890        900        910        920        930        940        950        960
                      ....|....| ....|....| ....|....| ....|....| ....|....| ....|....| ....|....| ....|....|
PFF0995c              TTCAAGAAAT TCTCAGTACG GATTCAAATA CATCTCATTT GAAGGAACGA AAAAATCAAA AAGCTCCACC CGGTGAACAT
3D7                   .......... .......... .......... .......... .......... .......... .......... ..........
RO33                  .......... .......... .......... .......... .......... .C........ .......... ..........
Palo Alto             .......... .......... .......... .......... .......... .......... .......... ..........
FCR3                  .......... .......... .......... .......... .......... .......... .......... ..........
Wellcome              .......... .......... .......... .......... .......... .......... .......... ..........
D6                    .......... .......... .......... .......... .......... .......... .......... ..........
T996                  .......... .......... .......... .......... .......... .......... .......... ..........
T9102                 .......... .......... .......... .......... .......... .......... ........T. ..........
K1                    .......... .......... .......... .......... .......... .......... .......... ..........
Dd2                   .......... .......... .......... .......... .......... .......... .......... ..........
D10                   .......... .......... .......... .......... .......... .......... .......... ..........
FCC2                  .......... .......... .......... .......... .......... .......... .......... ..........
HB3                   .......... .......... .......... .......... .......... .......... .......... ..........
7G8                   .......... .......... .......... .......... .......... .......... .......... ..........
P.reichenowi          .......... .......... .......... .......... ........A. ..G....... C.AA...... AA..C.....
```

```
                                   970       980       990      1000      1010      1020      1030      1040
                          ....|....| ....|....| ....|....| ....|....| ....|....| ....|....| ....|....| ....|....|
PFF0995c                  AAACCAGAAG TAAAAAATGC ATTGTTAAAT TCACAAGTAG CTTCACCTAA AGGAGAAGAT GAAAAAAAAT CACAACCTCA
3D7                       .......... .......... .......... .......... .......... .......... .......... ..........
RO33                      .......... .......... .....C.... .......... .......... .......... .......... ..........
Palo Alto                 .......... .......... .......... .......... .......... .......... .......... ..........
FCR3                      .......... .......... .....C.... .......... .......... .......... .......... ..........
Wellcome                  .......... .......... .....C.... .......... .......... .......... .......... ..........
D6                        .......... .......... .......... .......... .......... .......... .......... ..........
T996                      .......... .......... .......... .......... .......... .......... .......... ..........
T9102                     .......... .......... .....C.... .......... .......... .......... .......... ..........
K1                        .......... .......... .....C.... .......... .......... .......... .......... ..........
Dd2                       .......... .......... .....C.... .......... .......... .......... .......... ..........
D10                       .......... .......... .....C.... .......... .......... .......... .......... ..........
FCC2                      .......... .......... .....C.... .......... .......... .......... .......... ..........
HB3                       .......... .......... .....C.... .......... .......... .......... .......... ..........
7G8                       .......... .......... .......... .......... .......... .......... .......... ..........
P.reichenowi              ...T..C... G..C...G.G TG.A.C.... .........A T.......G. ....A...G. ...C...... ..........

                                  1050      1060      1070      1080      1090      1100      1110      1120
                          ....|....| ....|....| ....|....| ....|....| ....|....| ....|....| ....|....| ....|....|
PFF0995c                  ACACCCTTTA GTTAATAGTG GGGATCAAT- --TACAACAT CCAAAAGAAA TTGATGAGAA TGCGGAAAAA ATAAGAAGAA
3D7                       .......... .......... .........- --........ .......... .......... .......... ..........
RO33                      .......... .......... .........- --........ .......... .......... .......... ..........
Palo Alto                 .......... .......... .........- --........ .......... .......... .......... ..........
FCR3                      .......... .......... .........- --........ .......... .......... .......... ..........
Wellcome                  .......... .......... .........- --........ .......... .......... .......... ..........
D6                        .......... .......... .........- --........ .......... .......... .......... ..........
T996                      .......... .......... .........- --........ .......... .......... .......... ..........
T9102                     .......... .......... .........- --........ .......... .......... .......... ..........
K1                        .......... .......... .........- --........ .......... .......... .......... ..........
Dd2                       .......... .......... .........- --........ .......... .......... .......... ..........
D10                       .......... .......... .........- --........ .......... .......... .......... ..........
FCC2                      .......... .......... .........- --........ .......... .......... .......... ..........
HB3                       .....T.... .......... .........- --........ .......... .......... .......... ..........
7G8                       .......... .......... .........- --........ .......... .......... .......... ..........
P.reichenowi              .......... .....G.... ........AA AT..G....C .......... .......T.. .......... ..........

                                  1130      1140      1150      1160      1170      1180      1190      1200
                          ....|....| ....|....| ....|....| ....|....| ....|....| ....|....| ....|....| ....|....|
PFF0995c                  CGTTATTAAA AGAAAGCAGG GATATTAAAA ATACAACAGC CATAATAGAC GAAACAGTAT ATAAATTTGA ACAACTCATT
3D7                       .......... .......... .......... .......... .......... .......... .......... ..........
RO33                      .......... .......... .......... .......... .......... .......... .......... ..........
Palo Alto                 .......... .......... .......... .......... .......... .......... .......... ..........
FCR3                      .......... .......... .......... .......... .......... .......... .......... ..........
Wellcome                  .......... .......... .......... .......... .......... .......... .......... ..........
D6                        .......... .......... .......... .......... .......... .......... .......... ..........
T996                      .......... .......... .......... .......... .......... .......... .......... ..........
T9102                     .......... .....T.... .......... .......... .......... .......... .......... ..........
K1                        .......... .......... .......... .......... .......... .......... .......... ..........
Dd2                       .......... .......... .......... .......... .......... .......... .......... ..........
D10                       .......... .......... .......... .......... .......... .......... .......... ..........
FCC2                      .......... .......... .......... .......... .......... .......... .......... ..........
HB3                       .......... .......... .......... .......... .......... .......... .......... ..........
7G8                       .......... .......... .......... .......... .......... .......... .......... ..........
P.reichenowi              .......... .........A .......... .......... .......... .......... .......... ......G...

                                  1210      1220      1230      1240      1250      1260      1270      1280
                          ....|....| ....|....| ....|....| ....|....| ....|....| ....|....| ....|....| ....|....|
PFF0995c                  ATGAAAGGAA GATATTATGC CACTGCAGTG AGAAATTTTG TTATATTTAA AGTAAATTAT ATTTGTGAAT ATTCCAAATG
3D7                       .......... .......... .......... .......... .......... .......... .......... ..........
RO33                      .......... .......... .......... .......... .......... .......... .......... ..........
Palo Alto                 .......... .......... .......... .......... .......... .......... .......... ..........
FCR3                      .......... .......... .......... .A........ .......... .......... .......... ..........
Wellcome                  .......... .......... .......... .A........ .......... .......... .......... ..........
D6                        .......... .......... .......... .......... .......... .......... .......... ..........
T996                      .......... .......... .......... .......... .......... .......... .......... ..........
T9102                     .......... .......... .......... .A........ .......... .......... .......... ..........
K1                        .......... .......... .......... .A........ .......... .......... .......... ..........
Dd2                       .......... .......... .......... .A........ .......... .......... .......... ..........
D10                       .......... .......... .......... .A........ .......... .......... .......... ..........
FCC2                      .......... .......... .......... .A........ .......... .......... .......... ..........
HB3                       .......... .......... .......... .......... .......... .......... .......... ..........
7G8                       .......... .......... .......... .......... .......... .......... .......... ..........
P.reichenowi              .......... .......... .......... .A........ .......... .......... .......... ..........
```

```
                             1290       1300       1310       1320       1330       1340       1350       1360
                     ....|....| ....|....| ....|....| ....|....| ....|....| ....|....| ....|....| ....|....|
PFF0995c             TGGACCCAAT TCACGTTGTT ATATTGTCGA AAAAGATAAA GAACAATGTA GATGCCGACC TAATTATATA GTTGATATGA
3D7                  .......... .......... .......... .......... .......... .......... .......... ..........
RO33                 .......... .......... .......... .......... .......... .......... .......... ..........
Palo Alto            .......... .......... .......... .......... .......... .......... .......... ..........
FCR3                 .......... .......... .......... .......... .......... .......... .......... ..........
Wellcome             .......... .......... .......... .......... .......... .......... .......... ..........
D6                   .......... .......... .......... .......... .......... .......... .......... ..........
T996                 .......... .......... .......... .......... .......... .......... .......... ..........
T9102                .......... .......... .......... .......... .......... .......... .......... ..........
K1                   .......... .......... .......... .......... .......... .......... .......... ..........
Dd2                  .......... .......... .......... .......... .......... .......... .......... ..........
D10                  .......... .......... .......... .......... .......... .......... .......... ..........
FCC2                 .......... .......... .......... .......... .......... .......... .......... ..........
HB3                  .......... .......... .......... .......... .......... .......... .......... ..........
7G8                  .......... .......... .......... .......... .......... .......... .......... ..........
P.reichenowi         .......... .......... .......G.. .......... .......... .......... .......... ..........

                             1370       1380       1390       1400       1410       1420       1430       1440
                     ....|....| ....|....| ....|....| ....|....| ....|....| ....|....| ....|....| ....|....|
PFF0995c             GTGTAAATTA TTTTAAATGT ATACCTATGA AAGATATGAA CTGTTCAAAA AATAATGGAG GTTGTGATGT AAATGCAGAA
3D7                  .......... .......... .......... .......... .......... .......... .......... ..........
RO33                 .......... .......... .......... .......... .......... .......... .......... ..........
Palo Alto            .......... .......... .......... .......... .......... .......... .......... ..........
FCR3                 .......... .......... .......... .......... .......... .......... .......... ..........
Wellcome             .......... .......... .......... .......... .......... .......... .......... ..........
D6                   .......... .......... .......... .......... .......... .......... .......... ..........
T996                 .......... .......... .......... .......... .......... .......... .......... ..........
T9102                .......... .......... .......... .......... .......... .......... .......... ..........
K1                   .......... .......... .......... .......... .......... .......... .......... ..........
Dd2                  .......... .......... .......... .......... .......... .......... .......... ..........
D10                  .......... .......... .......... .......... .......... .......... .......... ..........
FCC2                 .......... .......... .......... .......... .......... .......... .......... ..........
HB3                  .......... .......... .......... .......... .......... .......... .......... ..........
7G8                  .......... .......... .......... .......... .......... .......... .......... ..........
P.reichenowi         .......... .......... .......... .......... .......... .......... .......... .........G

                             1450       1460       1470       1480       1490       1500       1510       1520
                     ....|....| ....|....| ....|....| ....|....| ....|....| ....|....| ....|....| ....|....|
PFF0995c             TGTACTATTG TTGAAGGTGC AGTAAAATGT CAGTGTTCAC ATCTTTATTT TGGTGATGGT GTATTTTGTG TAAAGAATTC
3D7                  .......... .......... .......... .......... .......... .......... .......... ..........
RO33                 .......... .......... .......... .......... .......... .......... .......... ..........
Palo Alto            .......... .......... .......... .......... .......... .......... .......... ..........
FCR3                 .......... .......... .......... .......... .A........ .......... .......... ..........
Wellcome             .......... .......... .......... .......... .A........ .......... .......... ..........
D6                   .......... .......... .......... .......... .......... .......... .......... ..........
T996                 .......... .......... .......... .......... .......... .......... .......... ..........
T9102                .......... .......... .......... .......... .A........ .......... .......... ..........
K1                   .......... .......... .......... .......... .A........ .......... .......... ..........
Dd2                  .......... .......... .......... .......... .A........ .......... .......... ..........
D10                  .......... .......... .......... .......... .......... .......... .......... ..........
FCC2                 .......... .......... .......... .......... .A........ .......... .......... ..........
HB3                  .......... .......... .......... .......... .......... .......... .......... ..........
7G8                  .......... .......... .......... .......... .......... .......... .......... ..........
P.reichenowi         .......... .......... .A........ .......... .......... .......... .......... ..........

                             1530       1540       1550       1560       1570       1580       1590
                     ....|....| ....|....| ....|....| ....|....| ....|....| ....|....| ....|....|
PFF0995c             TCAAACAAAA CAAACTTTAT ATATTTTATT TATAGTAATA TTGTTAGTTT TTCAAAATTT TTTCATTTAA
3D7                  .......... .......... .......... .......... .......... .......... ..........
RO33                 .......... .......... ........C. .......... .......... .......... ..........
Palo Alto            .......... .......... ........C. .......... .......... .......... ..........
FCR3                 .......... .......... ........C. .......... .......... .......... ..........
Wellcome             .......... .......... ........C. .......... .......... .......... ..........
D6                   .......... .......... ........C. .......... .......... .......... ..........
T996                 .......... .......... ........C. .......... .......... .......... ..........
T9102                .......... .......... ........C. .......... .......... .......... ..........
K1                   .......... .......... ........C. .......... .......... .......... ..........
Dd2                  .......... .......... ........C. .......... .......... .......... ..........
D10                  .......... .......... ........C. .......... .......... .......... ..........
FCC2                 .......... .......... ........C. .......... .......... .......... ..........
HB3                  .......... .......... ........C. .......... .......... .......... ..........
7G8                  .......... .......... ........C. .......... .......... .......... ..........
P.reichenowi         .......... .......... ........C. .......... .......... .......... ..........
```
