# Supplementary material for: Prospective Identification of Malaria Parasite Genes under Balancing Selection
Source: PLoS One. 2009 May 15;4(5):e5568. doi: 10.1371/journal.pone.0005568 (PMC2679211; doi:10.1371/journal.pone.0005568)
Supplement: Figure S2 — (0.36 MB PDF) [file pone.0005568.s002.pdf]

Supplementary Figure 2
MSP6

```
                               10         20         30         40         50         60         70         80
                       ....|....| ....|....| ....|....| ....|....| ....|....| ....|....| ....|....| ....|....|
PF10_0346_3D7          ATGAATAAGA TTTATAATAT TACTTTTCTT TTCATTCTTT TAAACTTATA TATAAATGAA AATAACTTTA TCAGAAATGA
PF10_0346_K1_AY518890  .......... .......... .......... .......... .......... .......... .......... ..........
3D7                                                     .......... .......... .......... .......... ..........
RO33                                                    .......... .......... .......... .......... ..........
Palo Alto                                               .......... .......... .......... .......... ..........
FCR3                                                    .......... .......... .......... .......... ..........
Wellcome                                                .......... .......... .......... .......... ..........
D6                                                      .......... .......... .......... .......... ..........
T996                                                    .......... .......... .......... .......... ..........
T9102                                                   .......... .......... .......... .......... ..........
K1                                                      .......... .......... .......... .......... ..........
Dd2                                                     .......... .......... .......... .......... ..........
D10                                                     .......... .......... .......... .......... ..........
FCC2                                                    .......... .......... .......... .......... ..........
7G8                                                     .......... .......... .......... .......... ..........
HB3                                                     .......... .......... .......... .......... ..........
P.reichenowi                                            .......... .......... .......... .......... ..........

                               90        100        110        120        130        140        150        160
                       ....|....| ....|....| ....|....| ....|....| ....|....| ....|....| ....|....| ....|....|
PF10_0346_3D7          ACTTATAAAC GAAAAAAACC ATAATTTAAG AAATGGTTCA ATGTATAATA ACGATAAAAT ATTAAGTAAA AATGAAGTAG
PF10_0346_K1_AY518890  .......... .......... .......... .......... .......... .......... .......... ..........
3D7                    .......... .......... .......... .......... .......... .......... .......... ..........
RO33                   .......... .......... .......... .......... .......... .......... .......... ..........
Palo Alto              .......... .......... .......... .......... .......... .......... .......... ..........
FCR3                   .......... .......... .......... .......... .......... .......... .......... ..........
Wellcome               .......... .......... .......... .......... .......... .......... .......... ..........
D6                     .......... .......... .......... .......... .......... .......... .......... ..........
T996                   .......... .......... .......... .......... .......... .......... .......... ..........
T9102                  .......... .......... .......... .......... .......... .......... .......... ..........
K1                     .......... .......... .......... .......... .......... .......... .......... ..........
Dd2                    .......... .......... .......... .......... .......... .......... .......... ..........
D10                    .......... .......... .......... .......... .......... .......... .......... ..........
FCC2                   .......... .......... .......... .......... .......... .......... .......... ..........
7G8                    .......... .......... .......... .......... .......... .......... .......... ..........
HB3                    .......... .......... .......... .......... .......... .......... .......... ..........
P.reichenowi           .......... .......... .......... .......... ..A....... .......... .......... ..........

                              170        180        190        200        210        220        230        240
                       ....|....| ....|....| ....|....| ....|....| ....|....| ....|....| ....|....| ....|....|
PF10_0346_3D7          ATACTAATAT AGAAAGTAAC GAAAATAGTA TTCACGAATC TGGACATAAG ATTGATGGGG AAGAAGTTTT AAAAGCTAAT
PF10_0346_K1_AY518890  .......... .......... .......... .......... .......... .......... .......... ..........
3D7                    .......... .......... .......... .......... .......... .......... .......... ..........
RO33                   .......... .......... .......... .......... .......... .......... .......... ..........
Palo Alto              .......... .......... .......... .......... .......... .......... .......... ..........
FCR3                   .......... .......... .......... .......... .......... .......... .......... ..........
Wellcome               .......... .......... .......... .......... .......... .......... .......... ..........
D6                     .......... .......... .......... .......... .......... .......... .......... ..........
T996                   .......... .......... .......... .......... .......... .......... .......... ..........
T9102                  .......... .......... .......... .......... .......... .......... .......... ..........
K1                     .......... .......... .......... .......... .......... .......... .......... ..........
Dd2                    .......... .......... .......... .......... .......... .......... .......... ..........
D10                    .......... .......... .......... .......... .......... .......... .......... ..........
FCC2                   .......... .......... .......... .......... .......... .......... .......... ..........
7G8                    .......... .......... .......... .......... .......... .......... .......... ..........
HB3                    .......... .......... .......... .......... .......... .......... .......... ..........
P.reichenowi           .......... .........T .......... .......... .......... .....G.... .........C ..........

                              250        260        270        280        290        300        310        320
                       ....|....| ....|....| ....|....| ....|....| ....|....| ....|....| ....|....| ....|....|
PF10_0346_3D7          ---------- -----GTAGA TGATATAACA TACAAAAAAA AAAATGTTGA TGATTCAGAA ATTCCTTTTT CTGGTTATGA
PF10_0346_K1_AY518890  CAAGAACAAG CTAAT..... A......... .......... .......... .......... .......... ......C...
3D7                    ---------- -----..... .......... .......... .......... .......... .......... ..........
RO33                   ---------- -----..... .......... .......... .......... .......... .......... ..........
Palo Alto              CAAGAACAAG CTAAT..... A......... .......... .......... .......... .......... ......C...
FCR3                   ---------- -----..... .......... .......... .......... .......... .......... ..........
Wellcome               ---------- -----..... .......... .......... .......... .......... .......... ..........
D6                     CAAGAACAAG CTAAT..... A......... .......... .......... .......... .......... ......C...
T996                   ---------- -----..... .......... .......... .......... .......... .......... ..........
T9102                  ---------- -----..... .......... .......... .......... .......... .......... ..........
K1                     CAAGAACAAG CTAAT..... A......... .......... .......... .......... .......... ......C...
Dd2                    CAAGAACAAG CTAAT..... A......... .......... .......... .......... .......... ......C...
D10                    ---------- -----..... .......... .......... .......... .......... .......... ..........
FCC2                   ---------- -----..... .......... .......... .......... .......... .......... ..........
7G8                    ---------- -----..... .......... .......... .......... .......... .......... ..........
HB3                    ---------- -----..... .......... .......... .......... .......... .......... ..........
P.reichenowi           CAAGAACAAC CTAAT..... A......... .......... ..G......C .......... .......... ......C...
```

```
                              330        340        350        360        370        380        390        400
                      ....|....| ....|....| ....|....| ....|....| ....|....| ....|....| ....|....| ....|....|
PF10_0346_3D7         TATACAAGCA ACATATCAAT TTCCTTCTAC ATCA------ ---------- --------GG AGGAAATAAT GTAATTCCAC
PF10_0346_K1_AY518890 ......G... .......... .....C.... .C..GGAAGA ATTATAAATC CTCGTACT.. ........C. ..........
3D7                   .......... .......... .......... ....------ ---------- --------.. .......... ..........
RO33                  .......... .......... .......... ....------ ---------- --------.. .......... ..........
Palo Alto             ......G... .......... .....C.... .C..GGAAGA ATTATAAATC CTCGTACT.. ........C. ..........
FCR3                  .......... .......... .......... ....------ ---------- --------.. .......... ..........
Wellcome              .......... .......... .......... ....------ ---------- --------.. .......... ..........
D6                    ......G... .......... .....C.... .C..GGAAGA ATTATAAATC CTCGTACT.. ........C. ......A...
T996                  .......... .......... .......... ....------ ---------- --------.. .......... ..........
T9102                 .......... .......... .......... ....------ ---------- --------.. .......... ..........
K1                    ......G... .......... .....C.... .C..GGAAGA ATTATAAATC CTCGTACT.. ........C. ..........
Dd2                   ......G... .......... .....C.... .C..GGAAGA ATTATAAATC CTCGTACT.. ........C. ..........
D10                   .......... .......... .......... ....------ ---------- --------.. .......... ..........
FCC2                  .......... .......... .......... ....------ ---------- --------.. .......... ..........
7G8                   .......... .......... .......... ....------ ---------- --------.. .......... ..........
HB3                   .......... .......... .......... ....------ ---------- --------.. .......... ..........
P.reichenowi          ......G.G. .......... .....CG..A .C..GGAAGA ATTACAAATC CTCGTACT.. ........C. A.........

                              410        420        430        440        450        460        470        480
                      ....|....| ....|....| ....|....| ....|....| ....|....| ....|....| ....|....| ....|....|
PF10_0346_3D7         TTCCTATAAA ACAAAGT--- ---------- ---------- ---------- ---------- ---------- ----------
PF10_0346_K1_AY518890 C.....G..G ..T....GGA CTAGAAGGTG TTTCTTATCC ACATGTATTT ACATCTTCTA ATCAATCACA TCCTCAAAGA
3D7                   .......... .......--- ---------- ---------- ---------- ---------- ---------- ----------
RO33                  .......... .......--- ---------- ---------- ---------- ---------- ---------- ----------
Palo Alto             C.....G..G ..T....GGA CTAGAAGGTG TTTCTTATCC ACATGTATTT ACATCTTCTA ATCAATCACA TCCTCAAAGA
FCR3                  .......... .......--- ---------- ---------- ---------- ---------- ---------- ----------
Wellcome              .......... .......--- ---------- ---------- ---------- ---------- ---------- ----------
D6                    C.....G..G ..T....GGA CTAGAAGGTG TTTCTTATCC ACATGTATTT ACATCTTCTA ATCAATCACA TCCTCAAAGA
T996                  .......... .......--- ---------- ---------- ---------- ---------- ---------- ----------
T9102                 .......... .......--- ---------- ---------- ---------- ---------- ---------- ----------
K1                    C.....G..G ..T....GGA CTAGAAGGTG TTTCTTATCC ACATGTATTT ACATCTTCTA ATCAATCACA TCCTCAAAGA
Dd2                   C.....G..G ..T....GGA CTAGAAGGTG TTTCTTATCC ACATGTATTT ACATCTTCTA ATCAATCACA TCCTCAAAGA
D10                   .......... .......--- ---------- ---------- ---------- ---------- ---------- ----------
FCC2                  .......... .......--- ---------- ---------- ---------- ---------- ---------- ----------
7G8                   .C........ .......--- ---------- ---------- ---------- ---------- ---------- ----------
HB3                   .......... .......--- ---------- ---------- ---------- ---------- ---------- ----------
P.reichenowi          C.....GT.G ..T....GGA CTAGAAGGTG TTTCTTATCC ATTTGTATAT ACATCTTCTA ATCAACCACG TCCTCAAAGA

                              490        500        510        520        530        540        550        560
                      ....|....| ....|....| ....|....| ....|....| ....|....| ....|....| ....|....| ....|....|
PF10_0346_3D7         ---------- ---------- ---------- ---------- ---------- ---------- ------GGAG AAAATCAATA
PF10_0346_K1_AY518890 GCCAACATCG GAGGAATATC CCATTTATCA GGGACACGTG GAATTCATAC TTATTCAGGT GAAAGT.... .......CC.
3D7                   ---------- ---------- ---------- ---------- ---------- ---------- ------.... ..........
RO33                  ---------- ---------- ---------- ---------- ---------- ---------- ------.... ..........
Palo Alto             GCCAACATCG GAGGAATATC CCATTTATCA GGGACACGTG GAATTCATAC TTATTCAGGT GAAAGT.... .......CC.
FCR3                  ---------- ---------- ---------- ---------- ---------- ---------- ------.... ..........
Wellcome              ---------- ---------- ---------- ---------- ---------- ---------- ------.... ..........
D6                    GCCAACATCG GAGGAATATC CCATTTATCA GGGACACGTG GAATTCATAC TTATTCAGGT GAAAGT.... .......CC.
T996                  ---------- ---------- ---------- ---------- ---------- ---------- ------.... ..........
T9102                 ---------- ---------- ---------- ---------- ---------- ---------- ------.... ..........
K1                    GCCAACATCG GAGGAATATC CCATTTATCA GGGACACGTG GAATTCATAC TTATTCAGGT GAAAGT.... .......CC.
Dd2                   GCCAACATCG GAGGAATATC CCATTTATCA GGGACACGTG GAATTCATAC TTATTCAGGT GAAAGT.... .......CC.
D10                   ---------- ---------- ---------- ---------- ---------- ---------- ------.... ..........
FCC2                  ---------- ---------- ---------- ---------- ---------- ---------- ------.... ..........
7G8                   ---------- ---------- ---------- ---------- ---------- ---------- ------.... ..........
HB3                   ---------- ---------- ---------- ---------- ---------- ---------- ------.... ..........
P.reichenowi          GCCAACATCG GAGGCATATC CCATTTATCA GGGATACGTG GAATTCATAC TTATTCAGGT GAAAGT.... ........C.

                              570        580        590        600        610        620        630        640
                      ....|....| ....|....| ....|....| ....|....| ....|....| ....|....| ....|....| ....|....|
PF10_0346_3D7         TACTGTTACA TCTATATCAG GTATTCAAAA GGGAGCAAAT GGTTTAACTG GTGCAACAGA AAATATTACA CAAGTTGTAC
PF10_0346_K1_AY518890 ..T....... A.C.G.C..A A..C...... CC.......A .......... .......... ...A...... ..C.G..C.G
3D7                   .......... .......... .......... .......... .......... .......... .......... ..........
RO33                  .......... .......... .......... .......... .......... .......... .......... ..........
Palo Alto             ..T....... A.C.G.C..A A..C...... CC.......A .......... .......... ...A...... ..C.G..C.G
FCR3                  .......... .......... .......... .......... .......... .......... .......... ..........
Wellcome              .......... .......... .......... .......... .......... .......... .......... ..........
D6                    ..T....... A.C.G.C..A A..C...... CC.......A .......... .......... ...A...... ..C.G..C.G
T996                  .......... .......... .......... .......... .......... .......... .......... ..........
T9102                 .......... .......... .......... .......... .......... .......... .......... ..........
K1                    ..T....... A.C.G.C..A A..C...... CC.......A .......... .......... ...A...... ..C.G..C.G
Dd2                   ..T....... A.C.G.C..A A..C...... CC.......A .......... .......... ...A...... ..C.G..C.G
D10                   .....G.... .......... .......... .......... .......... .......... .......... ..........
FCC2                  .......... .......... .......... .......... .......... .......... .......... ..........
7G8                   .......... .......... .......... .......... .......... .......... .......... ..........
HB3                   .......... .......... .......... .......... .......... .......... .......... ..........
P.reichenowi          ..T....... ..C.G....A A..C...... C......... .A........ ..A.....C. .G.A...... ....G..A..
```

```
                                    650       660       670       680       690       700       710       720
                            ....|....| ....|....| ....|....| ....|....| ....|....| ....|....| ....|....| ....|....|
PF10_0346_3D7               AAGCAAACTC TGAAACAAAT AAAAATCCTA CTTCTCATAG TAATAGTACT ACAACTTCTC TGAATAATAA TATACTTGGA
PF10_0346_K1_AY518890       ...A...... .......... .......... ..C..GG... ...A...... .......... .......... ..........
3D7                         .......... .......... .......... .......... .......... .......... .......... ..........
RO33                        .......... .......... .......... .......... .......... .......... .......... ..........
Palo Alto                   ...A...... .......... .......... ..C..GG... ...A...... .......... .......... ..........
FCR3                        .......... .......... .......... .......... .......... .......... .......... ..........
Wellcome                    .......... .......... .......... .......... .......... .......... .......... ..........
D6                          ...A...... .......... .......... ..C..GG... ...A...... .......... .......... ..........
T996                        .......... .......... .......... .......... .......... .......... .......... ..........
T9102                       .......... .......... .......... .......... .......... .......... .......... ..........
K1                          ...A...... .......... .......... ..C..GG... ...A...... .......... .......... ..........
Dd2                         ...A...... .......... .......... ..C..GG... ...A...... .......... .......... ..........
D10                         .......... .......... .......... .......... .......... .......... .......... ..........
FCC2                        .......... .......... .......... .......... .......... .......... .......... ..........
7G8                         .......... .......... .......... .......... .......... .......... .......... ..........
HB3                         .......... .......... .......... .......... .......... .......... .......... ..........
P.reichenowi                ......G... .......... .G........ .....GG... .......... .......... .......... .........C

                                    730       740       750       760       770       780       790       800
                            ....|....| ....|....| ....|....| ....|....| ....|....| ....|....| ....|....| ....|....|
PF10_0346_3D7               TGGGAATTTG GAGGAGGTGC TCCTCAAAAT GGAGCTGCAG AAGATAAAAA GACAGAATAT TTACTAGAAC AAATAAAAAT
PF10_0346_K1_AY518890       .......... .......... .......... .......... .......... .......... .......... ..........
3D7                         .......... .......... .......... .......... .......... .......... .......... ..........
RO33                        .......... .......... .......... .......... .......... .......... .......... ..........
Palo Alto                   .......... .......... .......... .......... .......... .......... .......... ..........
FCR3                        .......... .......... .......... .......... .......... .......... .......... ..........
Wellcome                    .......... .......... .......... .......... .......... .......... .......... ..........
D6                          .......... .......... .......... .......... .......... .......... .......... ..........
T996                        .......... .......... .......... .......... .......... .......... .......... ..........
T9102                       .......... .......... .......... .......... .......... .......... .......... ..........
K1                          .......... .......... .......... .......... .......... .......... .......... ..........
Dd2                         .......... .......... .......... .......... .......... .......... .......... ..........
D10                         .......... .......... .......... .......... .......... .......... .......... ..........
FCC2                        .......... .......... .......... .......... .......... .......... .......... ..........
7G8                         .......... .......... .......... .......... .......... .......... .......... ..........
HB3                         .......... .......... .......... .......... .......... .......... .......... ..........
P.reichenowi                .......... .......... .......... .......... .......... ....A.TC.. .......... ..........

                                    810       820       830       840       850       860       870       880
                            ....|....| ....|....| ....|....| ....|....| ....|....| ....|....| ....|....| ....|....|
PF10_0346_3D7               TCCATCATGG GATAGAAATA ACATCCCCGA TGAGAATGAA CAAGTAATAG AGGACCCTCA AGAAGATAAT AAAGATGAAG
PF10_0346_K1_AY518890       .......... .......... .......... .......... .......A.. .......... .......... ..........
3D7                         .......... .......... .......... .......... .......... .......... .......... ..........
RO33                        .......... .......... .......... .......... .......... .......... .......... ..........
Palo Alto                   .......... .......... .......... .......... .......A.. .......... .......... ..........
FCR3                        .......... .......... .......... .......... .......... .......... .......... ..........
Wellcome                    .......... .......... .......... .......... .......... .......... .......... ..........
D6                          .......... .......... .......... .......... .......A.. .......... .......... ..........
T996                        .......... .......... .......... .......... .......... .......... .......... ..........
T9102                       .......... .......... .......... .......... .......A.. .......... .......... ..........
K1                          .......... .......... .......... .......... .......A.. .......... .......... ..........
Dd2                         .......... .......... .......... .......... .......A.. .......... .......... ..........
D10                         .......... .......... .......... .......... .......... .......... .......... ..........
FCC2                        .......... .......... .......... .......... .......A.. .......... .......... ..........
7G8                         .......... .......... .......... .......... .......A.. .......... .......... ..........
HB3                         .......... .......... .......... .......... .......A.. .......... .......... ..........
P.reichenowi                .......... ......G... .......... .......... .......... .......... .......... ...A....C.

                                    890       900       910       920       930       940       950       960
                            ....|....| ....|....| ....|....| ....|....| ....|....| ....|....| ....|....| ....|....|
PF10_0346_3D7               ATGAAGATGA A------GAA ACAGAAACAG AAAATTTGGA AACAGAAGAT GATAATAATG AAGAGATAGA AGAAAATGAA
PF10_0346_K1_AY518890       .......... .------... .......... .......... .......... .......... .......... ..........
3D7                         .......... .------... .......... .......... .......... .......... .......... ..........
RO33                        .......... .------... .......... .......... .......... .......... .......... ..........
Palo Alto                   .......... .GATGAA... .......... .......... .......... .......... .......... ..........
FCR3                        .......... .------... .......... .......... .......... .......... .......... ..........
Wellcome                    .......... .------... .......... .......... .......... .......... .......... ..........
D6                          .......... .------... .......... .......... .......... .......... .......... ..........
T996                        .......... .------... .......... .......... .......... .......... .......... ..........
T9102                       .......... .------... .......... .......... .......... .......... .......... ..........
K1                          .......... .------... .......... .......... .......... .......... .......... ..........
Dd2                         .......... .------... .......... .......... .......... .......... .......... ..........
D10                         .......... .------... .......... .......... .......... .......... .......... ..........
FCC2                        .......... .------... .......... .......... .......... .......... .......... ..........
7G8                         .......... .------... .......... .......... .......... .......... .......... ..........
HB3                         .......... .------... .......... .......... .......... .......... .......... ..........
P.reichenowi                .G.....AAC .------... .......... .......... ...T.....G .......... .......... ..........
```

```
                                 970        980        990       1000       1010       1020       1030       1040
                          ....|....| ....|....| ....|....| ....|....| ....|....| ....|....| ....|....| ....|....|
PF10_0346_3D7             GAAGATGACA TAGATGAAGA AAGTGTAGAA GAAAAGGAAG AAGAGGAAGA AAAAAAGGAA GAAGAAGAAA AAAAGGAAGA
PF10_0346_K1_AY518890     .......... .......... .......... .......... .......... .......... .......... ..........
3D7                       .......... .......... .......... .......... .......... .......... .......... ..........
RO33                      .......... .......... .......... .......... .......... .......... .......... ....A.....
Palo Alto                 .......... .......... .......... .......... .......... .......... .......... ..........
FCR3                      .......... .......... .......... .......... .......... .......... .......... ..........
Wellcome                  .......... .......... .......... .......... .......... .......... .......... ..........
D6                        .......... .......... .......... .......... .......... .......... .......... ..........
T996                      .......... .......... .......... .......... .......... .......... .......... ..........
T9102                     .......... .......... .......... .......... .......... .......... .......... ..........
K1                        .......... .......... .......... .......... .......... .......... .......... ..........
Dd2                       .......... .......... .......... .......... .......... .......... .......... ..........
D10                       .......... .......... .......... .......... .......... .......... .......... ..........
FCC2                      .......... .......... .......... .......... .......... .......... .......... ..........
7G8                       .......... .......... .......... .......... .......... .......... .......... ..........
HB3                       .......... .......... .......... .......... .......... .......... .......... ..........
P.reichenowi              ........T. .......... ..T....... .....C.... ....T..CAT ...TG.T... AGT.T....G ..........

                                1050       1060       1070       1080       1090       1100       1110       1120
                          ....|....| ....|....| ....|....| ....|....| ....|....| ....|....| ....|....| ....|....|
PF10_0346_3D7             AAAAAAAGAA GAAAAAAAAC CAGACAATGA AATTACAAAT GAAGTTAAAG AGGAACAAAA ATATAGTTCA CCAAGTGATA
PF10_0346_K1_AY518890     .......... .......... .......... .......... .......... .......... .......... ..........
3D7                       .......... .......... .......... .......... .......... .......... .......... ..........
RO33                      .......... .......... .......... .......... .......... .......... .......... ..........
Palo Alto                 .......... .......... .......... .......... .......... .......... .......... ..........
FCR3                      .......... .......... .......... .......... .......... .......... .......... ..........
Wellcome                  .......... .......... .......... .......... .......... .......... .......... ..........
D6                        .......... .......... .......... .......... .......... .......... .......... ..........
T996                      .......... .......... .......... .......... .......... .......... .......... ..........
T9102                     .......... .......... .......... .......... .......... .......... .......... ..........
K1                        .......... .......... .......... .......... .......... .......... .......... ..........
Dd2                       .......... .......... .......... .......... .......... .......... .......... ..........
D10                       .......... .......... .......... .......... .......... .......... .......... ..........
FCC2                      .......... .......... .......... .......... .......... .......... .......... ..........
7G8                       .......... .......... .......... .......... .......... .......... .......... ..........
HB3                       .......... .......... .......... .......... .......... .......... .......... ..........
P.reichenowi              .....C.... .......C.- --..A...A. .......... ....G..... .......... GA...A.... ..........

                                1130       1140       1150       1160       1170       1180       1190       1200
                          ....|....| ....|....| ....|....| ....|....| ....|....| ....|....| ....|....| ....|....|
PF10_0346_3D7             TAAATGCCCA AAATTTAATT TCTAATAAGA ATAAAAAGAA TGATGAAACA AAAAAGACTG CTGAAAATAT AGTTAAAACA
PF10_0346_K1_AY518890     .......... .......... .......... .......... .......... .......... .......... ..........
3D7                       .......... .......... .......... .......... .......... .......... .......... ..........
RO33                      .......... .......... .......... .......T.. .......... .......... .......... ..........
Palo Alto                 .......... .......... .......... .......... .......... .......... .......... ..........
FCR3                      .......... .......... .......... .......... .......... .......... .......... ..........
Wellcome                  .......... .......... .......... .......... .......... .......... .......... ..........
D6                        .......... .......... .......... .......... .......... .......... .......... ..........
T996                      .......... .......... .......... .......... .......... .......... .......... ..........
T9102                     .......... .......... .......... .......... .......... .......... .......... ..........
K1                        .......... .......... .......... .......... .......... .......... .......... ..........
Dd2                       .......... .......... .......... .......... .......... .......... .......... ..........
D10                       .......... .......... .......... .......... .......... .......... .......... ..........
FCC2                      .......... .......... .......... .......... .......... .......... .......... ..........
7G8                       .......... .......... .......... .......... .......... .......... .......... ..........
HB3                       .......... .......... .......... .......... .......... .......... .......... ..........
P.reichenowi              .......... .......... .........C .......... .......... .......... .......... ..........

                                1210       1220       1230       1240       1250       1260       1270       1280
                          ....|....| ....|....| ....|....| ....|....| ....|....| ....|....| ....|....| ....|....|
PF10_0346_3D7             TTGGTTGGAT TATTTAATGA AAAAAATGAG ATAGATTCTA CTATAAATAA TTTAGTACAA GAAATGATCC ATCTATTTAG
PF10_0346_K1_AY518890     .......... .......... .......... .......... .......... .......... .......... ..........
3D7                       .......... .......... .......... .......... .......... ........
RO33                      .......... .......... .......... .......... .......... ........
Palo Alto                 .......... .......... .......... .......... .......... ........
FCR3                      .......... .......... .......... .......... .......... ........
Wellcome                  .......... .......... .......... .......... .......... ........
D6                        .......... .......... .......... .......... .......... ........
T996                      .......... .......... .......... .......... .......... ........
T9102                     .......... .......... .......... .......... .......... ........
K1                        .......... .......... .......... .......... .......... ........
Dd2                       .......... .......... .......... .......... .......... ........
D10                       .......... .......... .......... .......... .......... ........
FCC2                      .......... .......... .......... .......... .......... ........
7G8                       .......... .......... .......... .......... .......... ........
HB3                       .......... .......... .......... .......... .......... ........
P.reichenowi              .......... ....C.G... .......C.. .......... .......... ........
```

```
                                1290
                        ....|....|
PF10_0346_3D7           TAATAATTAA
PF10_0346_K1_AY518890   .......
3D7
RO33
Palo Alto
FCR3
Wellcome
D6
T996
T9102
K1
Dd2
D10
FCC2
7G8
HB3
P.reichenowi
```

PF10_0347

```
                                 10         20         30         40         50         60         70         80
                        ....|....| ....|....| ....|....| ....|....| ....|....| ....|....| ....|....| ....|....|
PF10_0347               ATGAAAAAAA TCGTGAATAT AATATTTTAT ATCTTATATT TATATATATA TAAAAGAAAC TTAGTACAAA ACGAAAATGT
3D7                                  ........ .......... .......... .......... .......... .......... ..........
RO33                                 ........ .......... .......... .......... .......... .......... ..........
Palo Alto                            ........ .......... .......... .......... .......... .......... ..........
FCR3                                 ........ .......... .......... .......... .......... .......... ..........
Wellcome                             ........ .......... .......... .......... .......... .......... ..........
D6                                   ........ .......... .......... .......... .......... .......... ..........
T996                                 ........ .......... .......... .......... .......... .......... ..........
T9102                                ........ .......... .......... .......... .......... .......... ..........
K1                                   ........ .......... .......... .......... .......... .......... ..........
Dd2                                  ........ .......... .......... .......... .......... .......... ..........
D10                                  ........ .......... .......... .......... .......... .......... ..........
FCC2                                 ........ .......... .......... .......... .......... .......... ..........
HB3                                  ........ .......... .......... .......... .......... .......... ..........
7G8                                  ........ .......... .......... .......... .......... .......... ..........
P.reichenowi                         ........ .......... .......... .......... .......... .......... ..A...C...

                                 90        100        110        120        130        140        150        160
                        ....|....| ....|....| ....|....| ....|....| ....|....| ....|....| ....|....| ....|....|
PF10_0347               AAATAAATCT AATCTAAGAA AAGGATTATC TACTAATAAT TCAGAAAATG GAATAAAAAG TCTAAAGGAT GAAGATGAAC
3D7                     .......... .......... .......... .......... .......... .......... .......... ..........
RO33                    .......... .......... .......... .......... .......... .......... .......... ..........
Palo Alto               .......... .......... .......... .......... .......... .......... .......... ..........
FCR3                    .......... .......... .......... .......... .......... .......... .......... ..........
Wellcome                .......... .......... .......... .......... .......... .......... .......... ..........
D6                      .......... .......... .......... .......... .......... .......... .......... ..........
T996                    .......... .......... .......... .......... .......... .......... .......... ..........
T9102                   .......... .......... .......... .......... .......... .......... .......... ..........
K1                      .......... .......... .......... .......... .......... .......... .......... ..........
Dd2                     .......... .......... .......... .......... .......... .......... .......... ..........
D10                     .......... .......... .......... .......... .......... .......... .......... ..........
FCC2                    .......... .......... .......... .......... .......... .......... .......... ..........
HB3                     .......... .......... .......... .......... .......... .......... .......... ..........
7G8                     .......... .......... .......... .......... .......... .......... .......... ..........
P.reichenowi            .......... .......... .G........ .......... ....G..... .......... .......... ..........

                                170        180        190        200        210        220        230        240
                        ....|....| ....|....| ....|....| ....|....| ....|....| ....|....| ....|....| ....|....|
PF10_0347               ATATTAATAT TATAGGAGAT GATTTTTCAG CATTTTCTTA TGGTGGTTAT CCTATTTATG AAACTACAGG AAGTCTAGGA
3D7                     .......... .......... .......... .......... .......... .......... .......... ..........
RO33                    .......... .......... .......... .......... .......... .......... .......... ..........
Palo Alto               .......... .......... .......... .......... .......... .......... .......... ..........
FCR3                    .......... .......... .......... .......... .......... .......... .......... ..........
Wellcome                .......... .......... .......... .......... .......... .......... .......... ..........
D6                      .......... .......... .......... .......... .......... .......... .......... ..........
T996                    .......... .......... .......... .......... .......... .......... .......... ..........
T9102                   .......... .......... .......... .......... .......... .......... .......... ..........
K1                      .......... .......... .......... .......... .......... .......... .......... ....T.....
Dd2                     .......... .......... .......... .......... .......... .......... .......... ....T.....
D10                     .......... .......... .......... .......... .......... .......... .......... ..........
FCC2                    .......... .......... .......... .......... .......... .......... .......... ..........
HB3                     .......... .......... .......... .......... .......... .......... .......... ..........
7G8                     .......... .......... .......... .......... .......... .......... .......... ..........
P.reichenowi            .......... .......... .......... .......... .......... .......... .......... ....G.....
```

```
                     250        260        270        280        290        300        310        320
              ....|....| ....|....| ....|....| ....|....| ....|....| ....|....| ....|....| ....|....|
PF10_0347     ACTGGGGTTG AAAGTGTAAA AGCTATTGAT GGGGAAAGTG GTACTTCAAT GGATTCTAAA CCTAAAGAGA ATAAAATTAG
3D7           .......... .......... .......... .......... .......... .......... .......... ..........
RO33          .......... .......... .......... .......... .......... .......... .......... ..........
Palo Alto     .......... .......... .......... .......... .......... .......... .......... ..........
FCR3          .......... .......... .......... .......... .......... .......... .......... ..........
Wellcome      .......... .......... .......... .......... .......... .......... .......... ..........
D6            .......... .......... .......... .......... .......... .......... .......... ..........
T996          .......... .......... .......... .......... .......... .......... .......... ..........
T9102         .......... .......... .......... .......... .......... .......... .......... ..........
K1            .......... .......... .......... .......... .......... .......... .......... ..........
Dd2           .......... .......... .......... .......... .......... .......... .......... ..........
D10           .......... .......... .......... .......... .......... .......... .......... ..........
FCC2          .......... .......... .......... .......... .......... .......... .......... ..........
HB3           .......... .......... .......... .......... C......... .......... .......... ..........
7G8           .......... .......... .......... .......... C......... .......... .......... ..........
P.reichenowi  ......C... ...C...... .......... .......A.. .......... .......... .....G.... .A........

                     330        340        350        360        370        380        390        400
              ....|....| ....|....| ....|....| ....|....| ....|....| ....|....| ....|....| ....|....|
PF10_0347     TACTGAACCA GGAGCAGACC AGGTGTCTAT TGGATTGGTC AACGAATCTG ATAGTAGTTT AGAAAATGAT AAAAAAAAAA
3D7           .......... .......... .......... .......... .......... .......... .......... ..........
RO33          .......... .......... .......... .......... .......... .......... .......... ..........
Palo Alto     .......... .......... .......... .......... .......... .......... .......... ..........
FCR3          .......... .......... .......... .......... .......... .......... .......... ..........
Wellcome      .......... .......... .......... .......... .......... .......... .......... ..........
D6            .......... .......... .......... .......... .......... .......... .......... ..........
T996          .......... .......... .......... .......... .......... .......... .......... ..........
T9102         .......... .......... .......... .......... .......... .......... .......... ..........
K1            .......... .......... .......... .......... .......... .......... .......... ..........
Dd2           .......... .......... .......... .......... .......... .......... .......... ..........
D10           .......... .......... .......... .......... .......... .......... .......... ..........
FCC2          .......... .......... .......... .......... .......... .......... .......... ..........
HB3           .......... .......... .......... .......... .......... .......... .......... ..........
7G8           .......... .......... .......... .......... .......... .......... .......... ..........
P.reichenowi  .......... ....T..G.. .....A.... .........A .......... .......... .......... GG........

                     410        420        430        440        450        460        470        480
              ....|....| ....|....| ....|....| ....|....| ....|....| ....|....| ....|....| ....|....|
PF10_0347     AAGAAAACGT AAAAAAAGAA ATGCTTGGTA CTGAAAAGGA AGGTTCTCCA GATAGTCATG ATAGTTCTAA GGAAAAATTA
3D7           .......... .......... .......... .......... .......... .......... .......... ..........
RO33          .......... .......... .......... .......... .......... .......... .......... ..........
Palo Alto     .......... .......... .......... .......... .......... .......... .......... ..........
FCR3          .......... .......... .......... .......... .......... .......... .......... ..........
Wellcome      .......... .......... .......... .......... .......... .......... .......... ..........
D6            .......... .......... .......... .......... .......... .......... .......... ..........
T996          .......... .......... .......... .......... .......... .......... .......... ..........
T9102         .......... .......... .......... .......... .......... .......... .......... ..........
K1            .......... .......... .......... .......... .......... .......... .......... ..........
Dd2           .......... .......... .......... .......... .......... .......... .......... ..........
D10           .......... .......... .......... .......... .......... .......... .......... ..........
FCC2          .......... .......... .......... .......... .......... .......... .......... ..........
HB3           .......... .......... .......... .......... .......... .......... .......... ..........
7G8           .......... .......... .......... .......... .......... .......... .......... ..........
P.reichenowi  .......... .......... .......... .......T.. .......... .......... .......... ..........

                     490        500        510        520        530        540        550        560
              ....|....| ....|....| ....|....| ....|....| ....|....| ....|....| ....|....| ....|....|
PF10_0347     AATCTTAACG ACAATTCCAA ATGGTCTGAT TTTCTTAAAA ATATCGTAAC GTTTGGTGGT TTTGGTCCTA CTGTGGTTCA
3D7           .......... .......... .......... .......... .......... .......... .......... ..........
RO33          .......... .......... .......... .......... .......... .......... .......... ..........
Palo Alto     .......... .......... .......... .......... .......... .......... .......... ..........
FCR3          .......... .......... .......... .......... ....G..... .......... .......... ..........
Wellcome      .......... .......... .......... .......... ....G..... .......... .......... ..........
D6            .......... .......... .......... .......... .......... .......... .......... ..........
T996          .......... .......... .......... .......... .......... .......... .......... ..........
T9102         .......... .......... .......... .......... .......... .......... .......... ..........
K1            .......... .......... .......... .......... .......... .......... .......... ..........
Dd2           .......... .......... .......... .......... .......... .......... .......... ..........
D10           .......... .......... .......... .......... ....G..... .......... .......... ..........
FCC2          .......... .......... .......... .......... .......... .......... .......... ..........
HB3           .......... .......... .......... .......... .......... .......... .......... ..........
7G8           .......... .......... .......... .......... .......... .......... .......... ..........
P.reichenowi  .......... .......T.. .......... .......... .........T .......... .......... T.........
```

```
                        570        580        590        600        610        620        630        640
                ....|....| ....|....| ....|....| ....|....| ....|....| ....|....| ....|....| ....|....|
PF10_0347       TGATGTTAGT GATACCCTTT CAGATATATC TAAAGATGAA GTTACTCAAA AAACAACTAA AGATATTGGT AGTACTTTAC
3D7             .......... .......... .......... .......... .......... .......... .......... ..........
RO33            .......... .......... .......... .......... .......... .......... .......... ..........
Palo Alto       .......... .......... .......... .......... .......... .......... .......... ..........
FCR3            .......... .......... .......... .......... .......... .......... .......... ..........
Wellcome        .......... .......... .......... .......... .......... .......... .......... ..........
D6              .......... .......... .......... .......... .......... .......... .......... ..........
T996            .......... .......... .......... .......... .......... .......... .......... ..........
T9102           .......... .......... .......... .......... .......... .......... .......... ..........
K1              .......... .......... .......... .......... .......... .......... .......... ..........
Dd2             .......... .......... .......... .......... .......... .......... .......... ..........
D10             .......... .......... .......... .......... .......... .......... .......... ..........
FCC2            .......... .......... .......... .......... .......... .......... .......... ..........
HB3             .......... .......... .......... .......... .......... .......... .......... ..........
7G8             .......... .......... .......... .......... .......... .......... .......... ..........
P.reichenowi    ......C... .....A.... .......... .......... .......... .G........ .......... ..........

                        650        660        670        680        690        700        710        720
                ....|....| ....|....| ....|....| ....|....| ....|....| ....|....| ....|....| ....|....|
PF10_0347       TTGACTTTTT TTTACCATTA CCGACTAAAA ACACTAATAC ATATGAGAAG AAAAATGAAA ATAAAAATGT ATCAAATGTA
3D7             .......... .......... .......... .......... .......... .......... .......... ..........
RO33            .......... .......... .......... .......... .......... .......... .......... ..........
Palo Alto       .......... .......... .......... .......... .......... .......... .......... ..........
FCR3            .......... .......... .......... .......... .......... .......... .......... ..........
Wellcome        .......... .......... .......... .......... .......... .......... .......... ..........
D6              .......... .......... .......... .......... .......... .......... .......... ..........
T996            .......... .......... .......... .......... .......... .......... .......... ..........
T9102           .......... .......... .......... .......... .......... .......... .......... ..........
K1              .......... .......... .......... .......... .......... .......... .......... ..........
Dd2             .......... .......... .......... .......... .......... .......... .......... ..........
D10             .......... .......... .......... .......... .......... .......... .......... ..........
FCC2            .......... .......... .......... .......... .......... .......... .......... ..........
HB3             .......... .......... .......... .......... .......... .......... .......... ..........
7G8             .......... .......... .......... .......... .......... .......... .......... ..........
P.reichenowi    .......... .......... ..A...C... .......... .......... .......... .........A .---...---

                        730        740        750        760        770        780        790        800
                ....|....| ....|....| ....|....| ....|....| ....|....| ....|....| ....|....| ....|....|
PF10_0347       GATTCAAAAA CAAAATCAAA TGAAAAAGGA AGACCTCCTA CATATTCTCC TATTCTGGAT GATGGGATAG AATTTAGTGG
3D7             .......... .......... .......... .......... .......... .......... .......... ..........
RO33            .......... .......... .......... .......... .......... .......... .......... ..........
Palo Alto       .......... .......... .......... .......... .......... .......... .......... ..........
FCR3            .......... .......... .......... .......... .......... .......... .......... ..........
Wellcome        .......... .......... .......... .......... .......... .......... .......... ..........
D6              .......... .......... .......... .......... .......... .......... .......... ..........
T996            .......... .......... .......... .......... .......... .......... .......... ..........
T9102           .......... .......... .......... .......... .......... .......... .......... ..........
K1              .......... .......... .......... .......... .......... .......... .......... ..........
Dd2             .......... .......... .......... .......... .......... .......... .......... ..........
D10             .......... .......... .......... .......... .......... .......... .......... ..........
FCC2            .......... .......... .......... .......... .......... .......... .......... ..........
HB3             .......... .......... .......... .......... .......... .......... .......... ..........
7G8             .......... .......... .......... .......... .......... .......... .......... ..........
P.reichenowi    ------.... ATGT...... .......... .CG....... .......... .........C .....C.... ..........

                        810        820        830        840        850        860        870        880
                ....|....| ....|....| ....|....| ....|....| ....|....| ....|....| ....|....| ....|....|
PF10_0347       TGGTTTATAT TTTAATGAGA AAAAGTCGAC TGAAGAAAAT AAACAAAAAA ATGTTTTAGA ATCAGTAAAT TTAACATCGT
3D7             .......... .......... .......... .......... .......... .......... .......... ..........
RO33            .......... .......... .......... .......... .......... .......... .......... ..........
Palo Alto       .......... .......... .......... .......... .......... .......... .......... ..........
FCR3            .......... .......... .......... .......... .......... .......... .......... ..........
Wellcome        .......... .......... .......... .......... .......... .......... .......... ..........
D6              .......... .......... .......... .......... .......... .......... .......... ..........
T996            .......... .......... .......... .......... .......... .......... .......... ..........
T9102           .......... .......... .......... .......... .......... .......... .......... ..........
K1              .......... .......... .......... .......... .......... .......... .......... ..........
Dd2             .......... .......... .......... .......... .......... .......... .......... ..........
D10             .......... .......... .......... .......... .......... .......... .......... ..........
FCC2            .......... .......... .......... .......... .......... .......... .......... ..........
HB3             .......... .......... .......... .......... .......... .......... .......... ..........
7G8             .......... .......... .......... .......... .......... .......... .......... ..........
P.reichenowi    .......... ....C..... .......... .......... .......... .......... .......... ..........
```

```
                           890        900        910        920        930        940        950        960
                    ....|....| ....|....| ....|....| ....|....| ....|....| ....|....| ....|....| ....|....|
PF10_0347           GGGATAAAGA GGATATTGTT AAGGAAAATG AGGATGTTAA AGATGAAAAG GATGAAGATG ATGAAGAAGA AGAAGAAAAA
3D7                 .......... .......... .......... .......... .......... .......... .......... ..........
RO33                .......... .......... .......... .......... .......... .......... .......... ..........
Palo Alto           .......... .......... .......... .......... .......... .......... .......... ..........
FCR3                .......... .......... .......... .......... .......... .......... .......... ..........
Wellcome            .......... .......... .......... .......... .......... .......... .......... ..........
D6                  .......... .......... .......... .......... .......... .......... .......... ..........
T996                .......... .......... .......... .......... .......... .......... .......... ..........
T9102               .......... .......... .......... .......... .......... .......... .......... ..........
K1                  .......... .......... .......... .......... .......... .......... .......... ..........
Dd2                 .......... .......... .......... .......... .......... .......... .......... ..........
D10                 .......... .......... .......... .......... .......... .......... .......... ..........
FCC2                .......... .......... .......... .......... .......... .......... .......... ..........
HB3                 .......... .......... .......... .......... .......... .......... .......... ..........
7G8                 .......... .......... .......... .......... .......... .......... .......... ..........
P.reichenowi        .......... C......... .......... .......A.. .......... ...A...... .......... ....---...

                           970        980        990       1000       1010       1020       1030       1040
                    ....|....| ....|....| ....|....| ....|....| ....|....| ....|....| ....|....| ....|....|
PF10_0347           TACGAAAACG AAATTATAAA GCAACCAGAA GACATATTGG ATGAGGAAGA AGTATTAGAA GAAGAAATAT TAGAAGAAAA
3D7                 .......... .......... .......... .......... .......... .......... .......... ..........
RO33                .......... .......... .......... .......... .......... .......... .......... ..........
Palo Alto           .......... .......... .......... .......... .......... .......... .......... ..........
FCR3                .......... .......... .......... .......... .......... .......... .......... ..........
Wellcome            .......... .......... .......... .......... .......... .......... .......... ..........
D6                  .......... .......... .......... .......... .......... .......... .......... ..........
T996                .......... .......... .......... .......... .......... .......... .......... ..........
T9102               .......... .......... .......... .......... .......... .......... .......... ..........
K1                  .......... .......... .......... .......... .......... .......... .......... ..........
Dd2                 .......... .......... .......... .......... .......... .......... .......... ..........
D10                 .......... .......... .......... .......... .......... .......... .......... ..........
FCC2                .......... .......... .......... .......... .......... .......... .......... ..........
HB3                 .......... .......... .......... .......... .......... .......... .......... ..........
7G8                 .......... .......... .......... .......... .......... .......... .......... ..........
P.reichenowi        .......G.. .......... .......... .......CA. ........-- ---------- ---....... ..........

                          1050       1060       1070       1080       1090       1100       1110       1120
                    ....|....| ....|....| ....|....| ....|....| ....|....| ....|....| ....|....| ....|....|
PF10_0347           TAAAAATGAT ACAGTAGATA CAAGTGATTT AGAAAAGAAA AATATACCAG ATTTATCAAA CGATAATAAT TATTATAGTT
3D7                 .......... .......... .......... .......... .......... .......... .......... ..........
RO33                ......A... .......... .......... .......... .......... .......... .......... ..........
Palo Alto           .......... .......... .......... .......... .......... .......... .......... ..........
FCR3                ......A... .......... .......... .......... .......... .......... .......... ..........
Wellcome            ......A... .......... .......... .......... .......... .......... .......... ..........
D6                  .......... .......... .......... .......... .......... .......... .......... ..........
T996                .......... .......... .......... .......... .......... .......... .......... ..........
T9102               .......... .......... .......... .......... .......... .......... .......... ..........
K1                  .......... .......... .......... .......... .......... .......... .......... ..........
Dd2                 .......... .......... .......... .......... .......... .......... .......... ..........
D10                 ......A... .......... .......... .......... .......... .......... .......... ..........
FCC2                .......... .......... .......... .......... .......... .......... .......... ..........
HB3                 .......... .......... .......... .......... .......... .......... .......... ..........
7G8                 .......... .......... .......... .......... .......... .......... .......... ..........
P.reichenowi        .......... .......... .......... .......G.. .......... .......... .......... ..........

                          1130       1140       1150       1160       1170       1180       1190       1200
                    ....|....| ....|....| ....|....| ....|....| ....|....| ....|....| ....|....| ....|....|
PF10_0347           TAATTTATAA GAACTATAAG GATAATGATA AATCAGAAAA AACTGCACAA ACATTAATCA CAGCTCTGAT AAGTTTATTA
3D7                 .......... .......... .......... .......... .......... .......... .......... ..........
RO33                .......... .......... .......... .......... .......... .......... .......... ..........
Palo Alto           .......... .......... .......... .......... .......... .......... .......... ..........
FCR3                .......... .......... .......... .......... .......... .......... .......... ..........
Wellcome            .......... .......... .......... .......... .......... .......... .......... ..........
D6                  .......... .......... .......... .......... .......... .......... .......... ..........
T996                .......... .......... .......... .......... .......... .......... .......... ..........
T9102               .......... .......... .......... .......... .......... .......... .......... ..........
K1                  .......... .......... .......... .......... .......... .......... .......... ..........
Dd2                 .......... .......... .......... .......... .......... .......... .......... ..........
D10                 .......... .......... .......... .......... .......... .......... .......... ..........
FCC2                .......... .......... .......... .......... .......... .......... .......... ..........
HB3                 .......... .......... .......... .......... .......... .......... .......... ..........
7G8                 .......... .......... .......... .......... .......... .......... .......... ..........
P.reichenowi        .......... .......... .......... .......... .......... .......... .......... ..........
```

```
                      1210       1220       1230       1240       1250       1260       1270
              ....|....| ....|....| ....|....| ....|....| ....|....| ....|....| ....|....| ....|
PF10_0347     AATGGAAAAA ATGAATTAGA TGCTACCATA AGAAGATTAA AACATAGGTT TATGGAATTT TTTACATATA ATTAA
3D7           .......... .......... .......... .......... ..
RO33          .......... .......... .......... .......... ..
Palo Alto     .......... .......... .......... .......... ..
FCR3          .......... .......... .......... .......... ..
Wellcome      .......... .......... .......... .......... ..
D6            .......... .......... .......... .......... ..
T996          .......... .......... .......... .......... ..
T9102         .......... .......... .......... .......... ..
K1            .......... .......... .......... .......... ..
Dd2           .......... .......... .......... .......... ..
D10           .......... .......... .......... .......... ..
FCC2          .......... .......... .......... .......... ..
HB3           .......... .......... .......... .......... ..
7G8           .......... .......... .......... .......... ..
P.reichenowi  .......... .......... .......... ...GA..... ..
```

PF10_0348

```
                        10         20         30         40         50         60         70         80
              ....|....| ....|....| ....|....| ....|....| ....|....| ....|....| ....|....| ....|....|
PF10_0348     ATGAAGAAAA TATATAGTAT TTTCTTTTCT TTATTTATTT TGAATCTTCA TATATATATA AAAAATATCA AATGCAATGA
3D7
RO33
Palo Alto
FCR3
Wellcome
D6
T996
T9102
K1
Dd2
D10
FCC2
HB3
7G8
P.reichenowi
```

```
                        90        100        110        120        130        140        150        160
              ....|....| ....|....| ....|....| ....|....| ....|....| ....|....| ....|....| ....|....|
PF10_0348     CCTAATAAAT TATAATGATT CGAATCTAAG AAACGGATTA CTAAATAATA GTTTAGATTT AACAAATGGA TTAAATAACA
3D7                            .... .......... .......... .......... .......... .......... ..........
RO33                           .... .......... .......... .......... .......... .......... ..........
Palo Alto                      .... .......... .......... .......... .......... .......... ..........
FCR3                           .... .......... .......... .......... .......... .......... ..........
Wellcome                       .... .......... .......... .......... .......... .......... ..........
D6                             .... .......... .......... .......... .......... .......... ..........
T996                           .... .......... .......... .......... .......... .......... ..........
T9102                          .... .......... .......... .......... .......... .......... ..........
K1                             .... .......... .......... .......... .......... .......... ..........
Dd2                            .... .......... .......... .......... .......... .......... ..........
D10                            .... .......... .......... .......... .......... .......... ..........
FCC2                           .... .......... .......... .......... .......... .......... ..........
HB3                            .... .......... .......... .......... .......... .......... ..........
7G8                            .... .......... .......... .......... .......... .......... ..........
P.reichenowi                   .... .......... .......... ....C..... ........GC .......... C.........
```

```
                       170        180        190        200        210        220        230        240
              ....|....| ....|....| ....|....| ....|....| ....|....| ....|....| ....|....| ....|....|
PF10_0348     AAGATAACAG TTTTATTGAT TCTAAAATTG AAGAACATGA AAATAAATCT TACCAAAATA AAGATAATAA TATCTCTATC
3D7           .......... .......... .......... .......... .......... .......... .......... ..........
RO33          .......... .......... .......... .......... .......... .......... .......... ..........
Palo Alto     .......... .......... .......... .......... .......... .......... .......... ..........
FCR3          .......... .......... .......... .......... .......... .......... .......... ..........
Wellcome      .......... .......... .......... .......... .......... .......... .......... ..........
D6            .......... .......... .......... .......... .......... .......... .......... ..........
T996          .......... .......... .......... .......... .......... .......... .......... ..........
T9102         .......... .......... .......... .......... .......... .......... .......... ..........
K1            .......... .......... .......... .......... .......... .......... .......... ..........
Dd2           .......... .......... .......... .......... .......... .......... .......... ..........
D10           .......... .......... .......... .......... .......... .......... .......... ..........
FCC2          .......... .......... .......... .......... .......... .......... .......... ..........
HB3           .......... .......... .......... .......... .......... .......... .......... ..........
7G8           .......... .......... .......... .......... .......... .......... .......... ..........
P.reichenowi  .......... .....A.... .......... .......... ......C... .......... .......... ..........
```

```
                          250        260        270        280        290        300        310        320
                    ....|....| ....|....| ....|....| ....|....| ....|....| ....|....| ....|....| ....|....|
PF10_0348           GTTGGACAAG ATGTGCCTAT TACATCGGTA TATTCTTCTA AAATTATAAA TGCTAATGAT TTAGAAGGAA ATAGTATTGA
3D7                 .......... .......... .......... .......... .......... .......... .......... ..........
RO33                .......... .......... .......... G......... .......... .......... .......... ..........
Palo Alto           .......... .......... .......... G......... .......... .......... .......... ..........
FCR3                .......... .......... .......... G......... .......... .......... .......... ..........
Wellcome            .......... .......... .......... G......... .......... .......... .......... ..........
D6                  .......... .......... .......... G......... .......... .......... .......... ..........
T996                .......... .......... .......... G......... .......... .......... .......... ..........
T9102               .......... .......... .......... G......... .......... .......... .......... ..........
K1                  .......... .......... .......... G......... .......... .......... .......... ..........
Dd2                 .......... .......... .......... G......... .......... .......... .......... ..........
D10                 .......... .......... .......... G......... .......... .......... .......... ..........
FCC2                .......... .......... .......... G......... .......... .......... .......... ..........
HB3                 .......... .......... .......... G......... .......... .......... .......... ..........
7G8                 .......... .......... .......... G......... .......... .......... .......... ..........
P.reichenowi        .......... .........C .......... GC........ .......... ......C... .......... ..........

                          330        340        350        360        370        380        390        400
                    ....|....| ....|....| ....|....| ....|....| ....|....| ....|....| ....|....| ....|....|
PF10_0348           CGATACTAAA GGTCTTAGTG TTACTAATAG TGGATTTGAT GATGGTAGTG CTTTTGGTGG TGGACTCCCT TTTTCTGGTT
3D7                 .......... .......... .......... .......... .......... .......... .......... ..........
RO33                .......... .......... .......... .......... .......... .......... .......... ..........
Palo Alto           .......... .......... .......... .......... .......... .......... .......... ..........
FCR3                .......... .......... .......... .......... .......... .......... .......... ..........
Wellcome            .......... .......... .......... .......... .......... .......... .......... ..........
D6                  .......... .......... .......... .......... .......... .......... .......... ..........
T996                .......... .......... .......... .......... .......... .......... .......... ..........
T9102               .......... .......... .......... .......... .......... .......... .......... ..........
K1                  .......... .......... .......... .......... .......... .......... .......... ..........
Dd2                 .......... .......... .......... .......... .......... .......... .......... ..........
D10                 .......... .......... .......... .......... .......... .......... .......... ..........
FCC2                .......... .......... .......... .......... .......... .......... .......... ..........
HB3                 .......... .......... .......... .......... .......... .......... .......... ..........
7G8                 .......... .......... .......... .......... .......... .......... .......... ..........
P.reichenowi        TA........ .......... .......... .......... .......... .......... .......... ..G.......

                          410        420        430        440        450        460        470        480
                    ....|....| ....|....| ....|....| ....|....| ....|....| ....|....| ....|....| ....|....|
PF10_0348           ATTCTCCTCT ACAAGGAAAT CATAATAAAT GTCCTGATGA AAATTTTTGT AAGGGTATTA AAAATGTCTT ATCCTGTCCT
3D7                 .......... .......... .......... .......... .......... .......... .......... ..........
RO33                .......... .......... .......... .......... .......... .......... ......C... ..........
Palo Alto           .......... .......... .......... ........A. .......... ..T......C .......GCC TAAT......
FCR3                .......... .......... .......... .......... .......... .......... .......... ..........
Wellcome            .......... .......... .......... .......... .......... .......... .......... ..........
D6                  .......... .......... .......... ........A. .......... ..T......C .......GCC TAAT......
T996                .......... .......... .......... .......... .......... .......... .......... ..........
T9102               .......... .......... .......... .......... .......... .......... ......C... ..........
K1                  .......... .......... .......... .......... .......... .......... ......C... ..........
Dd2                 .......... .......... .......... .......... .......... .......... ......C... ..........
D10                 .......... .......... .......... .......... .......... .......... ......C... ..........
FCC2                .......... .......... .......... .......... .......... .......... .......... ..........
HB3                 .......... .......... .......... ........A. .......... ..T......C .......GCC TAAT......
7G8                 .......... .......... .......... ........A. .......... ..T......C .......GCC TAAT......
P.reichenowi        .......... .......... .......... .....AG... .......... G......... ......C... ...T......

                          490        500        510        520        530        540        550        560
                    ....|....| ....|....| ....|....| ....|....| ....|....| ....|....| ....|....| ....|....|
PF10_0348           CCAAAAAATT CTACTGGTAG AAATGGGGAT TGGATTAGTG TGGCTGTTAA AGAAAGTTCA ACTACAAATA AAGGTGTTCT
3D7                 .......... .......... .......... .......... .......... .......... .......... ..........
RO33                .......... .......... .......... .......... ..A....... .......... .......... ..........
Palo Alto           .T........ TC.......C ...A..T... ...GC...CT CAAA...C.G .A.TTTC.T. ..AGTT.... .......C..
FCR3                .......... .......... ....C..... .......... .......... .......... .......... ..........
Wellcome            .......... .......... ....C..... .......... .......... .......... .......... ..........
D6                  .T........ TC.......C ...A..T... ...GC...CT CAAA...C.G .A.TTTC.T. ..AGTT.... .......C..
T996                .......... .......... .......... .......... .......... .......... .......... ..........
T9102               .......... .......... .......... .......... .......... .......... .......... ..........
K1                  .......... .......... .......... .......... .......... .......... .......... ..........
Dd2                 .......... .......... .......... .......... .......... .......... .......... ..........
D10                 .......... .......... .......... .......... .......... .......... .......... ..........
FCC2                .......... .......... .......... ...GC...CT CAAA...C.G .A.TTTC.T. ..AGTT.... .......C..
HB3                 .T....G... TC.......C ...A..T... ...GC...CT CAAA...C.G .A.TTTC.T. ..AGTT.... .......C..
7G8                 .T....G... TC.......C ...A..T... ...GC...CT CAAA...C.G .A.TTTC.T. ..AGTT.... .......C..
P.reichenowi        GA....C... T......... .......... .......... .A........ .......... .......... ..........
```

```
                           570        580        590        600        610        620        630        640
                    ....|....| ....|....| ....|....| ....|....| ....|....| ....|....| ....|....| ....|....|
PF10_0348           TGTTCCCCCC AGAAGAACAA AATTATGTCT AAGA---AAT ATTAACAAGG TTTGGCATCG AATCAAAGAC GAGAAAAATT
3D7                 .......... .......... .......... ....---... .......... .......... .......... ..........
RO33                .......... .......... .......... ....---... .......... .......... .......... ..........
Palo Alto           .........A .......A.C ..A.G...T. T...ATT... ..A.....TT ..CCTG.ATT ..AA...ACT ..AGGT..A.
FCR3                .......... .......... .......... ....---... .......... .......... .......... ..........
Wellcome            .......... .......... .......... ....---... .......... .......... .......... ..........
D6                  .........A .......A.C ..A.G...T. T...ATT... ..A.....TT ..CCTG.ATT ..AA...ACT ..AGGT..A.
T996                .......... .......A.. .......... ....---... ......C... .......... .......... ..........
T9102               .......... .......A.. .......... ....---... ......C... .......... .......... ..........
K1                  .......... .......... .......... ....---... .......... .......... .......... ..........
Dd2                 .......... .......... .......... ....---... .......... .......... .......... ..........
D10                 .......... .......... .......... ....---... .......... .......... .......... ..........
FCC2                .........A .......A.C ..A.G...T. T...ATT... ..A.....TT ..CCTG.ATT ..AA...ACT ..AGGT..A.
HB3                 .........A .......A.C ..A.G...T. T...ATT... ..A.....TT ..CCTA.ATT ..AA...ACT ..AGGT..A.
7G8                 .........A .......A.C ..A.G...T. T...ATT... ..A.....TT ..CCTA.ATT ..AA...ACT ..AGGT..A.
P.reichenowi        .........A .......G.. .......... ....---... ....C.C.TA .....T.... ...T..C... ..........

                           650        660        670        680        690        700        710        720
                    ....|....| ....|....| ....|....| ....|....| ....|....| ....|....| ....|....| ....|....|
PF10_0348           TTAAAGAAGA ATTTGTTAAA GTTGCTTTAG GAGAATCAAA TGCTTTAATG AAACATTATA AAGAAAAAAA TCTGAATGCC
3D7                 .......... .......... .......... .......... .......... .......... .......... ..........
RO33                .......... .......... .......... .......... .......... .......... .......... ..........
Palo Alto           ..G..A.TTT CA..TACTC. TC...AGGTT CT...G.T.. ACAA.....A ...TTA...G GTA.T..C.C AGAA..A..A
FCR3                .......... .......... .......... .......... .......... .......... .......... ..........
Wellcome            .......... .......... .......... .......... .......... .......... .......... ..........
D6                  ..G..A.TTT CA..TACTC. TC...AGGTT CT...G.T.. ACAA.....A ...TTA...G GTA.T..C.C AGAA..A..A
T996                .......... .......... .......... .......... .......... .......... .......... ..........
T9102               .......... .......... .......... .......... .......... .......... .......... ..........
K1                  .......... .......... .......... .......... .......... .......... .......... ..........
Dd2                 .......... .......... .......... .......... .......... .......... .......... ..........
D10                 .......... .......... .......... .......... .......... .......... .......... ..........
FCC2                ..G..A.TTT CA..TACTC. TC...AGGTT CT...G.T.. ACAA.....A ...TTA...G GTA.T..C.C AGAA..A..A
HB3                 ..G..A.TTT CA..TACTC. TC...AGGTT CT...G.T.. ACAA.....A ...TTA...G GTA.T..C.C AGAA..A..A
7G8                 ..G..A.TTT CA..TACTC. TC...AGGTT CT...G.T.. ACAA.....A ...TTA...G GTA.T..C.C AGAA..A..A
P.reichenowi        ........A. ....T..... .A........ .......... .........A ...T...... .......... ...A...C.A

                           730        740        750        760        770        780        790        800
                    ....|....| ....|....| ....|....| ....|....| ....|....| ....|....| ....|....| ....|....|
PF10_0348           CTTACAGCTA TAAAATATGG ATTTTCAGAT ATGGGAGATA TAATAAAGGG AACAGACCTA ATTGACTATC AAATTACTAA
3D7                 .......... .......... .......... .......... .......... .......... .......... ..........
RO33                .......... .......... .......... .......... .......... .......... .......... ..........
Palo Alto           ...CA...A. .G........ T...G.C... ..T...A... .TG.CC.A.. ..AT...A.G ..A..TACA. C..C.T....
FCR3                .......... .......... .......... .......... .......... .......... .......... ..........
Wellcome            .......... .......... .......... .......... .......... .......... .......... ..........
D6                  ...CA...A. .G........ T...G.C... ..T...A... .TG.CC.A.. ..AT...A.G ..A..TACA. C..C.T....
T996                .......... .......... .......... .......... .......... .......... .......... ..........
T9102               .......... .......... .......... .......... .......... .......... .......... ..........
K1                  .......... .......... .......... .......... .......... .......... .......... ..........
Dd2                 .......... .......... .......... .......... .......... .......... .......... ..........
D10                 .......... .......... .......... .......... .......... .......... .......... ..........
FCC2                ...CA...A. .G........ T...G.C... ..T...A... .TG.CC.A.. ..AT...A.G ..A..TACA. C..C.T....
HB3                 ...CA...A. .G........ T...G.C... ..T...A... .TG.CC.A.. ..AT...A.G ..A..TACA. C..C.T....
7G8                 ...CA...A. .G........ T...G.C... ..T...A... .TG.CC.A.. ..AT...A.G ..A..TACA. C..C.T....
P.reichenowi        .......... .......... .......... .......... .......... .......T.. .......... ..........

                           810        820        830        840        850        860        870        880
                    ....|....| ....|....| ....|....| ....|....| ....|....| ....|....| ....|....| ....|....|
PF10_0348           AAATATAAAT AGGGCATTAG ATAAAATATT ACGTAATGAA ACAAGTAAT- --GACAAAAT TAAAAAACGT GTAGACTGGT
3D7                 .......... .......... .......... .......... .........- --........ .......... ..........
RO33                .......... .......... .......... .G........ .........- --........ .......... ..........
Palo Alto           T..A.C...A .CATAT..G. .AG..G..C. CG.A..AC.. TAT.AA...G TTA.TG.TCC A...G.TGC. AA.AC.....
FCR3                .......... .......... .......... .G........ .........- --........ .......... ..........
Wellcome            .......... .......... .......... .G........ .........- --........ .......... ..........
D6                  T..A.C...A .CATAT..G. .AG..G..C. CG.A..AC.. TAT.AA...G TTA.TG.TCC A...G.TGC. AA.AC.....
T996                .......... .......... .......... .G........ .........- --........ .......... ..........
T9102               .......... .......... .......... .G........ .........- --........ .......... ..........
K1                  .......... .......... .......... .G........ .........- --........ .......... ..........
Dd2                 .......... .......... .......... .G........ .........- --........ .......... ..........
D10                 .......... .......... .......... .G........ .........- --........ .......... ..........
FCC2                T..A.C...A .CATAT..G. .AG..G..C. CG.A..AC.. TAT.AA...G TTA.TG.TCC A...G.TGC. AA.AC.....
HB3                 T..A.C...A .CATAT..G. .AG..G..C. CG.A..AC.. TAT.AA...G TTA.TG.TCC A...G.TGC. AA.AC.....
7G8                 T..A.C...A .CATAT..G. .AG..G..C. CG.A..AC.. TAT.AA...G TTA.TG.TCC A...G.TGC. AA.AC.....
P.reichenowi        .......... .......... .......... .G........ .GG......- --.C.G.... A....T.... ..........
```

```
                          890        900        910        920        930        940        950        960
                   ....|....| ....|....| ....|....| ....|....| ....|....| ....|....| ....|....| ....|....|
PF10_0348          GGGAAGCTAA TAAAAGTGCA TTCTGGGATG CATTCATGTG TGGATATAAA GTTCATATCG GAAATAAACC ATGTCCAGAA
3D7                .......... .......... .......... .......... .......... .......... .......... ..........
RO33               .......... .......... .......... .......... .......... .........T .......... ..........
Palo Alto          ..ATTCAA.. C...CA.CGT G.T....... ..A.G..... .......... .......... .......... ..........
FCR3               .......... .......... .......... .......... .......... .......... .......... ..........
Wellcome           .......... .......... .......... .......... .......... .......... .......... ..........
D6                 ..ATTCAA.. C...CA.CGT G.T....... ..A.G..... .......... .......... .......... ..........
T996               .......... .......... .......... .......... .......... .......... .......... ..........
T9102              .......... .......... .......... .......... .......... .......... .......... ..........
K1                 .......... .......... .......... .......... .......... .......... .......... ..........
Dd2                .......... .......... .......... .......... .......... .......... .......... ..........
D10                .......... .......... .......... .......... .......... .......... .......... ..........
FCC2               ..ATTCAA.. C...CA.CGT G.T....... ..A.G..... .......... .......... .......... ..........
HB3                ..ATTCAA.. C...CA.CGT G.T....... ..A.G..... C......C.. TA.G.A.AAA A.G....TAA ....A...G.
7G8                ..ATTCAA.. C...CA.CGT G.T....... ..A.G..... C......C.. TA.G.A.AAA A.G....TAA ....A...G.
P.reichenowi       .......... ........A. ..A....... ....A..... .......... A.......A. ........G. ..........

                          970        980        990       1000       1010       1020       1030       1040
                   ....|....| ....|....| ....|....| ....|....| ....|....| ....|....| ....|....| ....|....|
PF10_0348          CATGATAATA TGGACAGAAT ACCACAATAT CTTAGATGGT TTAGAGAATG GGGAACATAT GTTTGCAGCG AATATAAAAA
3D7                .......... .......... .......... .......... .......... .......... .......... ..........
RO33               .......... .......... .......... .......... .......... .......... .......... ..........
Palo Alto          .......... .......... .......... .......... .......... .......... .......... ..........
FCR3               .......... .......... .......... .......... .......... .......... .......... ..........
Wellcome           .......... .......... .......... .......... .......... .......... .......... ..........
D6                 .......... .......... .......... .......... .......... .......... .......... ..........
T996               .......... .......... .......... .......... .......... .......... .......... ..........
T9102              .......... .......... .......... .......... .......... .......... .......... ..........
K1                 .......... .......... .......... .......... .......... .......... .......... ..........
Dd2                .......... .......... .......... .......... .......... .......... .......... ..........
D10                .......... .......... .......... .......... .......... .......... .......... ..........
FCC2               .......... .......... .......... .......... .......... .......... .......... ..........
HB3                T...GC.... .AT.TGAT.. .......... .......... .......... .......... .......... ..........
7G8                T...GC.... .AT.TGAT.. .......... .......... .......... .......... .......... ..........
P.reichenowi       .......... .......... .......... .......... .......... .......... .....T.... ..........

                         1050       1060       1070       1080       1090       1100       1110       1120
                   ....|....| ....|....| ....|....| ....|....| ....|....| ....|....| ....|....| ....|....|
PF10_0348          TAAGTTTGAG GATGTAATAA AATTATGTAA TATCCAACAA TTTACAAACC AGGATGATTC ACAACTATTA GAAATATCAA
3D7                .......... .......... .......... .......... .......... .......... .......... ..........
RO33               .......... A........G .......... .G.AAG.... A......... ..A....... .......... ..........
Palo Alto          .......... A........G .......... .G.AAG.... A......... ..A....... .......... ..........
FCR3               .......... A........G .......... .G.AAG.... A......... ..A....... .......... ..........
Wellcome           .......... A........G .......... .G.AAG.... A......... ..A....... .......... ..........
D6                 .......... A........G .......... .G.AAG.... A......... ..A....... .......... ..........
T996               .......... .......... .......... .......... .......... .......... .......... ..........
T9102              .......... .......... .......... .......... .......... ..A....... .......... ..........
K1                 .......... A........G .......... .G.AAG.... A......... ..A....... .......... ..........
Dd2                .......... A........G .......... .G.AAG.... A......... ..A....... .......... ..........
D10                .......... .......... .......... .......... .......... .......... .......... ..........
FCC2               .......... A........G .......... .G.AAG.... A......... ..A....... .......... ..........
HB3                .......... .......... .......... .......... .......... .......... .......... ..........
7G8                .......... .......... .......... .......... .......... .......... .......... ..........
P.reichenowi       .......... A......... .......... .......... A......... .......... .......... .G.T......

                         1130       1140       1150       1160       1170       1180       1190       1200
                   ....|....| ....|....| ....|....| ....|....| ....|....| ....|....| ....|....| ....|....|
PF10_0348          AAAAGGATAA ATGTAAAGAA GCATTAAAGC ATTATGAAGA ATGGGTTAAT AGAAGGAGAC CTGAATGGAA AGGCCAATGT
3D7                .......... .......... .......... .......... .......... .......... .......... ..........
RO33               .......... ........GT .......... .......... .......... .......... .......... ...T......
Palo Alto          .......... ........GT .......... .......... .......... .......... .......... ...T......
FCR3               .......... ........GT .......... .......... .......... .......... .......... ...T......
Wellcome           .......... ........GT .......... .......... .......... .......... .......... ...T......
D6                 .......... ........GT .......... .......... .......... .......... .......... ...T......
T996               .......... .......... .......... .......... .......... .......... .......... ..........
T9102              .......... ........GT .......... .......... .......... .......... .......... ...T......
K1                 .......... ........GT .......... .......... .......... .......... .......... ...T......
Dd2                .......... ........GT .......... .......... .......... .......... .......... ...T......
D10                .......... .......... .......... .......... .......... .......... .......... ..........
FCC2               .......... ........GT .......... .......... .......... .......... .......... ...T......
HB3                .......... .......... .......... .......... .......... .......... .......... ..........
7G8                .......... .......... .......... .......... .......... .......... .......... ..........
P.reichenowi       .TG.T..... .........T .......... .......... ....A..... .......... .......... ...T......
```

```
                      1210       1220       1230       1240       1250       1260       1270       1280
                  ....|....| ....|....| ....|....| ....|....| ....|....| ....|....| ....|....| ....|....|
PF10_0348         GATAAATTTG AAAAAGAAAA AAGTAAATAT GAAGATACTA AAAGTATAAC TGCTGAAAAA TATTTAAAAG AAATATGTTC
3D7               .......... .......... .......... .......... .......... .......... .......... ..........
RO33              .......... .......... .......... .......... ......G... ........T. .......... ..........
Palo Alto         .......... .......... .......... .......... ......G... ........T. .......... ..........
FCR3              .......... .......... .......... .......... ......G... ........T. .......... ..........
Wellcome          .......... .......... .......... .......... ......G... ........T. .......... ..........
D6                .......... .......... .......... .......... ......G... ........T. .......... ..........
T996              .......... .......... .......... .......... .......... .......... .......... ..........
T9102             .......... .......... .......... .......... ......G... ........T. .......... ..........
K1                .......... .......... .......... .......... ......G... ........T. .......... ..........
Dd2               .......... .......... .......... .......... ......G... ........T. .......... ..........
D10               .......... .......... .......... .......... .......... .......... .......... ..........
FCC2              .......... .......... .......... .......... ......G... ........T. .......... ..........
HB3               .......... .......... .......... .......... .......... .......... .......... ..........
7G8               .......... .......... .......... .......... .......... .......... .......... ..........
P.reichenowi      .......... .......... ..A....... .......... .......... .......... .......... ..........

                      1290       1300       1310       1320       1330       1340       1350       1360
                  ....|....| ....|....| ....|....| ....|....| ....|....| ....|....| ....|....| ....|....|
PF10_0348         TGAATGTGAT TGTAAATATA AAGATTTGGA TAATACATTT AAAGAATTTA AAGATAACGT TACACTTCTT AAAGCAGTAA
3D7               .......... .......... .......... .......... .......... .......... .......... ..........
RO33              .......... .......... .......... .......... .......... .......... ...G...... ..........
Palo Alto         .......... .......... .......... .......... .......... .T........ ...G...... ..........
FCR3              .......... .......... .......... .......... .......... .......... ...G...... ..........
Wellcome          .......... .......... .......... .......... .......... .......... ...G...... ..........
D6                .......... .......... .......... .......... .......... .T........ ...G...... ..........
T996              .......... .......... .......... .......... .......... .......... .......... ..........
T9102             .......... .......... .......... .......... .......... .T........ ...G...... ..........
K1                .......... .......... .......... .......... .......... .......... ...G...... ..........
Dd2               .......... .......... .......... .......... .......... .......... ...G...... ..........
D10               .......... .......... .......... .......... .......... .......... .......... ..........
FCC2              .......... .......... .......... .......... .......... .T........ ...G...... ..........
HB3               .......... .......... .......... .......... .......... .......... .......... ..........
7G8               .......... .......... .......... .......... .......... .......... .......... ..........
P.reichenowi      .......... .......... .......... .......... .......... .......A.. ...G...... ..........

                      1370       1380       1390       1400       1410       1420       1430       1440
                  ....|....| ....|....| ....|....| ....|....| ....|....| ....|....| ....|....| ....|....|
PF10_0348         TTGATAACAA AAAAAATCAA GATTCTCTAA CAACCACTTC TTTATCAACG TCTATTAATA GTGTTAGGGA TTCTAGTAAT
3D7               .......... .......... .......... .......... .......... .......... .......... ..........
RO33              .......... .......... .......... .......... .......... .......... .......... ..........
Palo Alto         .......... .......... .......... .......... .......... .......... .......... ..........
FCR3              .......... .......... .......... .......... .......... .......... .......... ..........
Wellcome          .......... .......... .......... .......... .......... .......... .......... ..........
D6                .......... .......... .......... .......... .......... .......... .......... ..........
T996              .......... .......... .......... .......... .......... .......... .......... ..........
T9102             .......... .......... .......... .......... .......... .......... .......... ..........
K1                .......... .......... .......... .......... .......... .......... .......... ..........
Dd2               .......... .......... .......... .......... .......... .......... .......... ..........
D10               .......... .......... .......... ....A..... ....A..... .......... ...G...... ..........
FCC2              .......... .......... .......... .......... .......... .......... .......... ..........
HB3               .......... .......... .......... ....A..... ....A..... .......... ...G...... ..........
7G8               .......... .......... .......... ....A..... ....A..... .......... ...G...... ..........
P.reichenowi      ....G..... .......... .......... ....A..A-- ---------- ---------- ---------- ----------

                      1450       1460       1470       1480       1490       1500       1510       1520
                  ....|....| ....|....| ....|....| ....|....| ....|....| ....|....| ....|....| ....|....|
PF10_0348         CTAGATCAAC GAGGGAATAT AACAACATCT CAAGGAAATT CACACCGTGC AACTGTTGTG CAACAAGTTG ATCAAACCAA
3D7               .......... .......... .......... .......... .......... .......... .......... ..........
RO33              .......... .......... .......... .......... .......... .......... .......C.. ..........
Palo Alto         .......... .......... .......... .......... .......... .......... .......C.. ..........
FCR3              .......... .......... .......... .......... .......... .......... .......C.. ..........
Wellcome          .......... .......... .......... .......... .......... .......... .......C.. ..........
D6                .......... .......... .......... .......... .......... .......... .......C.. ..........
T996              .......... .......... .......... .......... .......... .......... .......C.. ..........
T9102             .......... .......... .......... .......... .......... .......... .......C.. ..........
K1                .......... .......... .......... .......... .......... .......... .......C.. ..........
Dd2               .......... .......... .......... .......... .......... .......... .......C.. ..........
D10               ...A...... .......... .......... .......... .......... .......... .......C.. ..........
FCC2              .......... .......... .......... .......... .......... .......... .......C.. ..........
HB3               ...A...... .......... .......... .......... .......... .......... .......C.. ..........
7G8               ...A...... .......... .......... .......... .......... .......... .......C.. ..........
P.reichenowi      ---------- ---------- -------... .......... .........A .......... ......CCC. ...G......
```

```
                    1530       1540       1550       1560       1570       1580       1590       1600
               ....|....| ....|....| ....|....| ....|....| ....|....| ....|....| ....|....| ....|....|
PF10_0348      CAGATTAGAT AATGTAAACT CTGTAACGCA AAGAGGAAAT AATAACTACA ACAATAATTT AGAGCGTGGA TTGGGTTCTG
3D7            .......... .......... .......... .......... .......... .......... .......... ..........
RO33           .......... .......... .......... .......... .......... .......... .......... ..........
Palo Alto      .......... .......... .......... .......... .......... .......... .......... ..........
FCR3           .......... .......... .......... .......... .......... .......... .......... ..........
Wellcome       .......... .......... .......... .......... .......... .......... .......... ..........
D6             .......... .......... .......... .......... .......... .......... .......... ..........
T996           .......... .......... .......... .......... .......... .......... .......... ..........
T9102          .......... .......... .......... .......... .......... .......... .......... ..........
K1             .......... .......... .......... .......... .......... .......... .......... ..........
Dd2            .......... .......... .......... .......... .......... .......... .......... ..........
D10            .......... .......... .......... .......... .......... .......... .......... ..........
FCC2           .......... .......... .......... .......... .......... .......... .......... ..........
HB3            .......... .......... .......... .......... .......... .......... .......... ..........
7G8            .......... .......... .......... .......... .......... .......... .......... ..........
P.reichenowi   T.T....... .........G .A........ .......... ......C... .......... .......... ......CA..

                    1610       1620       1630       1640       1650       1660       1670       1680
               ....|....| ....|....| ....|....| ....|....| ....|....| ....|....| ....|....| ....|....|
PF10_0348      GTGCTCTTCC TGGTACAAAT ATTATTACTG AAGAAAAATA TTCTCTAGAA TTAATAAAAT TAACATCAAA GGATGAAGAA
3D7            .......... .......... .......... .......... .......... .......... .......... ..........
RO33           .......... .......... .......... .......... .......... .......... .......... ..........
Palo Alto      .......... .......... .......... .......... .......... .......... .......... ..........
FCR3           .......... .......... .......... .......... .......... .......... .......... ..........
Wellcome       .......... .......... .......... .......... .......... .......... .......... ..........
D6             .......... .......... .......... .......... .......... .......... .......... ..........
T996           .......... .......... .......... .......... .......... .......... .......... ..........
T9102          .......... .......... .......... .......... .......... .......... .......... ..........
K1             .......... .......... .......... .......... .......... .......... .......... ..........
Dd2            .......... .......... .......... .......... .......... .......... .......... ..........
D10            .......... .......... .......... .......... .......... .......... .......... ..........
FCC2           .......... .......... .......... .......... .......... .......... .......... ..........
HB3            .......... .......... .......... .......... .......... .......... .......... ..........
7G8            .......... .......... .......... .......... .......... .......... .......... ..........
P.reichenowi   .......... .......... .......... ........A. ACT....... ..T.....T. .......... ..........

                    1690       1700       1710       1720       1730       1740       1750       1760
               ....|....| ....|....| ....|....| ....|....| ....|....| ....|....| ....|....| ....|....|
PF10_0348      GATATTATAA AGCATAATGA GGATGTGAGA GAAGAAATAG AAGAACAACA AGAAGACATC GAGGAAGATG AAGAAGAATT
3D7            .......... .......... .......... .......... .......... .......... .......... ..........
RO33           .......... .......... .......... .......... .......... .......... .......... ..........
Palo Alto      .......... .......... .......... .......... .......... .......... .......... ..........
FCR3           .......... .......... .......... .......... .......... .......... .......... ..........
Wellcome       .......... .......... .......... .......... .......... .......... .......... ..........
D6             .......... .......... .......... .......... .......... .......... .......... ..........
T996           .......... .......... .......... .......... .......... .......... .......... ..........
T9102          .......... .......... .......... .......... .......... .......... .......... ..........
K1             .......... .......... .......... .......... .......... .......... .......... ..........
Dd2            .......... .......... .......... .......... .......... .......... .......... ..........
D10            .......... .......... .......... .......... .......... .......... .......... ..........
FCC2           .......... .......... .......... .......... .......... .......... .......... ..........
HB3            .......... .......... .......... .......... .......... .......... .......... ..........
7G8            .......... .......... .......... .......... .......... .......... .......... ..........
P.reichenowi   .......C.. .A........ .......... .......... .......... ......A..A .......... ..........

                    1770       1780       1790       1800       1810       1820       1830       1840
               ....|....| ....|....| ....|....| ....|....| ....|....| ....|....| ....|....| ....|....|
PF10_0348      GGAAAATGAA GGAGAAGAA- ---------- ---------- ---------- ---------- ---------- ---ACAAAAG
3D7            .......... .........- ---------- ---------- ---------- ---------- ---------- ---.......
RO33           .......... .........- ---------- ---------- ---------- ---------- ---------- ---.......
Palo Alto      .......... .........- ---------- ---------- ---------- ---------- ---------- ---.......
FCR3           .......... .........- ---------- ---------- ---------- ---------- ---------- ---.......
Wellcome       .......... .........- ---------- ---------- ---------- ---------- ---------- ---.......
D6             .......... .........- ---------- ---------- ---------- ---------- ---------- ---.......
T996           .......... .........- ---------- ---------- ---------- ---------- ---------- ---.......
T9102          .......... .........- ---------- ---------- ---------- ---------- ---------- ---.......
K1             .......... .........- ---------- ---------- ---------- ---------- ---------- ---.......
Dd2            .......... .........- ---------- ---------- ---------- ---------- ---------- ---.......
D10            .......... .........- ---------- ---------- ---------- ---------- ---------- ---.......
FCC2           .......... .........- ---------- ---------- ---------- ---------- ---------- ---.......
HB3            .......... .........- ---------- ---------- ---------- ---------- ---------- ---.......
7G8            .......... .........- ---------- ---------- ---------- ---------- ---------- ---.......
P.reichenowi   A......... .........C AACAAGAAGA AATAGAGGAA GATGAAGAAG AATTGGAAAA TGAAGGAGAA GAA.......
```

```
                  1850       1860       1870       1880       1890       1900       1910       1920
              ....|....| ....|....| ....|....| ....|....| ....|....| ....|....| ....|....| ....|....|
PF10_0348     AAGAAGATGA TGAAGAAAAG AATGAAACAA ATGATACGGA AGAT------ ---ACGGACG ATACTGAAGA TACGGAAGAT
3D7           .......... .......... .......... .......... ....------ ---....... .......... ..........
RO33          .......... .......... .......... .......... ....------ ---....... .......... ..........
Palo Alto     .......... .......... .......... .......... ....------ ---....... .......... ..........
FCR3          .......... .......... .......... .......... ....------ ---....... .......... ..........
Wellcome      .......... .......... .......... .......... ....------ ---....... .......... ..........
D6            .......... .......... .......... .......... ....------ ---....... .......... ..........
T996          .......... .......... .......... .......... ....ACGGAA GAT....... .......... ..........
T9102         .......... .......... .......... .......... ....------ ---....... .......... ..........
K1            .......... .......... .......... .......... ....------ ---....... .......... ..........
Dd2           .......... .......... .......... .......... ....------ ---....... .......... ..........
D10           .......... .......... .......... .......... ....------ ---....... .......... ..........
FCC2          .......... .......... .......... .......... ....------ ---....... .......... ..........
HB3           .......... .......... .......... .......... ....------ ---....... .......... ..........
7G8           .......... .......... .......... .......... ....------ ---....... .......... ..........
P.reichenowi  .......... .......... .......... .......... ....------ ---------- ---------- ----------

                  1930       1940       1950       1960       1970       1980       1990       2000
              ....|....| ....|....| ....|....| ....|....| ....|....| ....|....| ....|....| ....|....|
PF10_0348     ATAGAAGAGG AAAATAAGGA AAAAGAACTC AGTAATCAAC AACAAAGTGA AAAAAAAAGT ATTTCAAAAG TTGACGAAGA
3D7           .......... .......... .......... .......... .......... .......... .......... ..........
RO33          .......... .......... .......... .......... .......... .......... .......... ..........
Palo Alto     .......... .......... .......... .......... .......... .......... .......... ..........
FCR3          .......... .......... .......... .......... .......... .......... .......... ..........
Wellcome      .......... .......... .......... .......... .......... .......... .......... ..........
D6            .......... .......... .......... .......... .......... .......... .......... ..........
T996          .......... .......... .......... .......... .......... .......... .......... ..........
T9102         .......... .......... .......... .......... .......... .......... .......... ..........
K1            .......... .....G.... .......... .......... .......... .......... .......... ..........
Dd2           .......... .....G.... .......... .......... .......... .......... .......... ..........
D10           .......... .....G.... .......... .......... .......... .......... .......... ..........
FCC2          .......... .......... .......... .......... .......... .......... .......... ..........
HB3           .......... .......... .......... .......... .......... .......... .......... ..........
7G8           .......... .......... .......... .......... .......... .......... .......... ..........
P.reichenowi  .......... .......... .....G.... .........A .......... .G........ .......... ....T.....

                  2010       2020       2030       2040       2050       2060       2070       2080
              ....|....| ....|....| ....|....| ....|....| ....|....| ....|....| ....|....| ....|....|
PF10_0348     TTCATATCGA ATACTATCAG TAAGTTATAA GGACAATAAT GAAGTAAAAA ATGTTGCTGA ATCTATAGTG AAAAAACTAT
3D7           .......... .......... .......... .......... .......... .......... .......... ..........
RO33          .......... .......... .......... .......... .......... .......... .......... ..........
Palo Alto     .......... .......... .......... .......... .......... .......... .......... ..........
FCR3          .......... .......... .......... .......... .......... .......... .......... ..........
Wellcome      .......... .......... .......... .......... .......... .......... .......... ..........
D6            .......... .......... .......... .......... .......... .......... .......... ..........
T996          .......... .......... .......... .......... .......... .......... .......... ..........
T9102         .......... .......... .......... .......... .......... .......... .......... ..........
K1            .......... .......... .......... .......... .......... .C........ .......... ..........
Dd2           .......... .......... .......... .......... .......... .C........ .......... ..........
D10           .......... .......... .......... .......... .......... .C........ .......... ..........
FCC2          .......... .......... .......... .......... .......... .......... .......... ..........
HB3           .......... .......... .......... .......... .......... .......... .......... ..........
7G8           .......... .......... .......... .......... .......... .......... .......... ..........
P.reichenowi  .......... .......... .......... .......... .......... .C...C.... .......... ..........

                  2090       2100       2110       2120       2130       2140       2150       2160
              ....|....| ....|....| ....|....| ....|....| ....|....| ....|....| ....|....| ....|....| ...
PF10_0348     TTAGTTTATT TAATGATAAT AATAATTTGG AAACTATTTT TAAGGGTTTG ACAGAAGATA TGACAGATTT ATTTCAAAAA TAA
3D7           .......... .......... .......... .......... .......... .........
RO33          .......... .......... .......... .......... .......... .........
Palo Alto     .......... .......... .......... .......... .......... .........
FCR3          .......... .......... .......... .......... .......... .........
Wellcome      .......... .......... .......... .......... .......... .........
D6            .......... .......... .......... .......... .......... .........
T996          .......... .......... .......... .......... .......... .........
T9102         .......... .......... .......... .......... .......... .........
K1            .......... .......... .......... .......... .......... .........
Dd2           .......... .......... .......... .......... .......... .........
D10           .......... .......... .......... .......... .......... .........
FCC2          .......... .......... .......... .......... .......... .........
HB3           .......... .......... .......... .......... .......... .........
7G8           .......... .......... .......... .......... .......... .........
P.reichenowi  .......... .......... .......... .......... .......... .........
```

PF10_0352

```
                       10         20         30         40         50         60         70         80
               ....|....| ....|....| ....|....| ....|....| ....|....| ....|....| ....|....| ....|....|
PF10_0352      ATGAATAAGT TTTTGAATAT TATATTTTAC ATTTTTCTAA TATTAAATTT CTCTTTCTTC CAAAGCAATG CCACAAGTAA
3D7                            ..... .......... .......... .......... .......... .......... ..........
RO33                           ..... .......... .......... .......... .......... .......... ..........
Palo Alto                      ..... .......... .......... .......... .......... .......... ..........
FCR3                           ..... .......... .......... .......... .......... .......... ..........
Wellcome                       ..... .......... .......... .......... .......... .......... ..........
D6                             ..... .......... .......... .......... .......... .......... ..........
T996                           ..... .......... .......... .......... .......... .......... ..........
T9102                          ..... .......... .......... .......... .......... .......... ..........
K1                             ..... .......... .......... .......... .......... .......... ..........
Dd2                            ..... .......... .......... .......... .......... .......... ..........
D10                            ..... .......... .......... .......... .......... .......... ..........
FCC2                           ..... .......... .......... .......... .......... .......... ..........
HB3                            ..... .......... .......... .......... .......... .......... ..........
7G8                            ..... .......... .......... .......... .......... .......... ..........
P.reichenowi                   ..... .......... .......... .......... .......... .......... .T........

                       90        100        110        120        130        140        150        160
               ....|....| ....|....| ....|....| ....|....| ....|....| ....|....| ....|....| ....|....|
PF10_0352      GGAAATTCAA AAAGATGAAC AAAAGAATTT AAGAAATGGT TCTTCAATAA ATAATAACAA AAATATAGAA AATAAAAATG
3D7            .......... .......... .......... .......... .......... .......... .......... ..........
RO33           .......... .......... .......... .......... .......... .......... .......... ..........
Palo Alto      .......... .......... .......... .......... .......... .......... .......... ..........
FCR3           .......... .......... .......... .......... .......... .......... .......... ..........
Wellcome       .......... .......... .......... .......... .......... .......... .......... ..........
D6             .......... .......... .......... .......... .......... .......... .......... ..........
T996           .......... .......... .......... .......... .......... .......... .......... ..........
T9102          .......... .......... .......... .......... .......... .......... .......... ..........
K1             .......... .......... .......... .......... .......... .......... .......... ..........
Dd2            .......... .......... .......... .......... .......... .......... .......... ..........
D10            .......... .......... .......... .......... .......... .......... .......... ..........
FCC2           .......... .......... .......... .......... .......... .......... .......... ..........
HB3            .......... .......... .......... .......... .......... .......... .......... ..........
7G8            .......... .......... .......... .......... .......... .......... .......... ..........
P.reichenowi   .....A...G .......... .......... ........T. .......... ...---.... .......... ..........

                      170        180        190        200        210        220        230        240
               ....|....| ....|....| ....|....| ....|....| ....|....| ....|....| ....|....| ....|....|
PF10_0352      ATAATATTGA AACTCAATAT GAAGCTTCAG AATATATAGA AAAACAAAAT GACATTTTAA ATATGTATAA TGATGAAAAA
3D7            .......... .......... .......... .......... .......... .......... .......... ..........
RO33           .......... .......... .......... .......... ......G... .......... .......... ..........
Palo Alto      .......... .......... .......... .......... ......G... .......... .......... ..........
FCR3           .......... .......... .......... .......... ......G... .......... .......... ..........
Wellcome       .......... .......... .......... .......... ......G... .......... .......... ..........
D6             .......... .......... .......... .......... ......G... .......... .......... ..........
T996           .......... .......... .......... .......... ......G... .......... .......... ..........
T9102          .......... .......... .......... .......... ......G... .......... .......... ..........
K1             .......... .......... .......... .......... ......G... .......... .......... ..........
Dd2            .......... .......... .......... .......... ......G... .......... .......... ..........
D10            .......... .......... .......... .......... ......G... .......... .......... ..........
FCC2           .......... .......... .......... .......... ......G... .......... .......... ..........
HB3            .......... .......... .......... .......... ......G... .......... .......... ..........
7G8            .......... .......... .......... .......... ......G... .......... .......... ..........
P.reichenowi   .......... .G........ .......... ..C....... ......G... ..T....... .......... .........G

                      250        260        270        280        290        300        310        320
               ....|....| ....|....| ....|....| ....|....| ....|....| ....|....| ....|....| ....|....|
PF10_0352      GAGAAAAATA ATAATAATTC ATTAGATACA AATGTAACAA AAAATACTGT AATTGATAAT TCAAATAAAT TTCAATCAAT
3D7            .......... .......... .......... .......... .......... .......... .......... ..........
RO33           .......... .......... .......... .......... .......... .......... .......... ..........
Palo Alto      .......... .......... .......... .......... .......... .......... .......... ..........
FCR3           .......... .......... .......... .......... .......... .......... .......... ..........
Wellcome       .......... .......... .......... .......... .......... .......... .......... ..........
D6             .......... .......... .......... .......... .......... .......... .......... ..........
T996           .......... .......... .......... .......... .......... .......... .......... ..........
T9102          .......... .......... .......... .......... .......... .......... .......... ..........
K1             .......... .......... .......... .......... .......... .......... .......... ..........
Dd2            .......... .......... .......... .......... .......... .......... .......... ..........
D10            .......... .......... .......... .......... .......... .......... .......... ..........
FCC2           .......... .......... .......... .......... .......... .......... .......... ..........
HB3            .......... .......... .......... .......... .......... .......... .......... ..........
7G8            .......... .......... .......... .......... .......... .......... .......... ..........
P.reichenowi   ........G. .......... .......... ...A...... .......... ......C... ......G... .....A...G
```

```
                        330        340        350        360        370        380        390        400
                ....|....| ....|....| ....|....| ....|....| ....|....| ....|....| ....|....| ....|....|
PF10_0352       TGAAGACAAT AATGTATACA ATAAAGGTAT ATTTGTAGGT ACTGGGATAA AATTAAATGA TTCACAAACT ACATCTGATA
3D7             .......... .......... .......... .......... .......... .......... .......... ..........
RO33            .......... .......... .......... .......... .......... .......... .......... ..........
Palo Alto       .......... .......... .......... .......... .......... .......... .......... ..........
FCR3            .......... .......... .......... .......... .......... .......... .......... ..........
Wellcome        .......... .......... .......... .......... .......... .......... .......... ..........
D6              .......... .......... .......... .......... .......... .......... .......... ..........
T996            .......... .......... .......... .......... .......... .......... .......... ..........
T9102           .......... .......... .......... .......... .......... .......... .......... ..........
K1              .......... .......... .......... .......... .......... .......... .......... ..........
Dd2             .......... .......... .......... .......... .......... .......... .......... ..........
D10             .......... .......... .......... .......... .......... .......... .......... ..........
FCC2            .......... .......... .......... .......... .......... .......... .......... ..........
HB3             .......... .......... .......... .......... .......... .......... .......... ..........
7G8             .......... .......... .......... .......... .......... .......... .......... ..........
P.reichenowi    ......T.G. .......... .......... .......... .......... .......... .......... ..........

                        410        420        430        440        450        460        470        480
                ....|....| ....|....| ....|....| ....|....| ....|....| ....|....| ....|....| ....|....|
PF10_0352       ATTACAAAAA TGAACGATAT CAAATAGACG ATGAAAAATT GAAGTATGGA GGGTCGTTTG ACACAATTTT TTCAGGTTTT
3D7             .......... .......... .......... .......... .......... .......... .......... ..........
RO33            .......... .......... .......... .......... .......... .......... .......... ..........
Palo Alto       .......... .......... .......... .......... .......... .......... .......... ..........
FCR3            .......... .......... .......... .......... .......... .......... .......... ..........
Wellcome        .......... .......... .......... .......... .......... .......... .......... ..........
D6              .......... .......... .......... .......... .......... .......... .......... ..........
T996            .......... .......... .......... .......... .......... .......... .......... ..........
T9102           .......... .......... .......... .......... .......... .......... ........A. ..........
K1              .......... .......... .......... .......... .......... .......... .......... ..........
Dd2             .......... .......... .......... .......... .......... .......... .......... ..........
D10             .......... .......... .......... .......... .......... .......... .......... ..........
FCC2            .......... .......... .......... .......... .......... .......... .......... ..........
HB3             .......... .......... .......... .......... .......... .......... .......... ..........
7G8             .......... .......... .......... .......... .......... A...G..... .......... ..........
P.reichenowi    .......... .......... .......... ..A.....G. .......... ....G..... ..T....... ....A.....

                        490        500        510        520        530        540        550        560
                ....|....| ....|....| ....|....| ....|....| ....|....| ....|....| ....|....| ....|....|
PF10_0352       GTTAATTTAT TAACACCATC AAGTCCTACT CAAAACGATG GATCTACAGG AAGAAATGTA CCACCTCCTA GTGAACCTAA
3D7             .......... .......... .......... .......... .......... .......... .......... ..........
RO33            .......... .......... .......... .......... .......... .......... .......... ..........
Palo Alto       .......... .......... .......... .......... .......... .......... .......... ..........
FCR3            .......... .......... .......... .......... .......... .......... .......... ..........
Wellcome        .......... .......... .......... .......... .......... .......... .......... ..........
D6              .......... .......... .......... .......... .......... .......... .......... ..........
T996            .......... .......... .......... .......... .......... .......... .......... ..........
T9102           .......... .......... .......... .......... .......... .......... .......... ..........
K1              .......... .......... .......... .......... .......... .......... .......... ..........
Dd2             .......... .......... .......... .......... .......... .......... .......... ..........
D10             .......... .......... .......... .......... .......... .......... .......... ..........
FCC2            .......... .......... .......... .......... .......... .......... .......... ..........
HB3             .......... .......... .......... .......... .......... .......... .......... ..........
7G8             .......... .......... .......... .......... .......... .......... .......... ..........
P.reichenowi    .......... ........A. C.A.....T. ........C. .......... .G........ .......A.. ...C......

                        570        580        590        600        610        620        630        640
                ....|....| ....|....| ....|....| ....|....| ....|....| ....|....| ....|....| ....|....|
PF10_0352       TGTTGATACA CCAGATCCTC CAACAGCACC CGCACCTGTA AAGGTACCTG AAGATGCAAA ATTATCAAGT TCTCCTAGAC
3D7             .......... .......... .......... .......... .......... .......... .......... ..........
RO33            .......... .......... .......... .......... .......... .......... .......... ..........
Palo Alto       .......... .......... .......... .......... .......... .......... .......... ..........
FCR3            .......... .......... .......... .......... .......... .......... .......... ..........
Wellcome        .......... .......... .......... .......... .......... .......... .......... ..........
D6              .......... .......... .......... .......... .......... .......... .......... ..........
T996            .......... .......... .......... .......... .......... .......... .......... ..........
T9102           .......... .......... .......... .......... .......... .......... .......... ..........
K1              .......... .......... .......... .......... .......... .......... .......... ..........
Dd2             .......... .......... .......... .......... .......... .......... .......... ..........
D10             .......... .......... .......... .......... .......... .......... .......... ..........
FCC2            .......... .......... .......... .......... .......... .......... .......... ..........
HB3             .......... .......... .......... .......... .......... .......... .......... ..........
7G8             .......... .......... ........T. .......... .......... .......... .......... ..........
P.reichenowi    ........G. T....AA..T .....A..T. ....T..... .......... ......A... .......... C.........
```

```
                         650        660        670        680        690        700        710        720
                  ....|....| ....|....| ....|....| ....|....| ....|....| ....|....| ....|....| ....|....|
PF10_0352         CTGAAGGACC AAGAGCAAAC AATAGAAATG AAAATAATCA AAATACAGAT CCATATAACC ACTATTTTGC ATGGGAAATT
3D7               .......... .......... .......... .......... .......... .......... .......... ..........
RO33              .......... .......... .......... .......... .......... .......... .......... ..........
Palo Alto         .......... .......... .......... .......... .......... .......... .......... ..........
FCR3              .......... .......... .......... .......... .......... .......... .......... ..........
Wellcome          .......... .......... .......... .......... .......... .......... .......... ..........
D6                .......... .......... .......... .......... .......... .......... .......... ..........
T996              .......... .......... .......... .......... .......... .......... .......... ..........
T9102             .......... .......... .......... .......... .......... .......... .......... ..........
K1                .......... .......... .......... .......... .......... .......... .......... ..........
Dd2               .......... .......... .......... .......... .......... .......... .......... ..........
D10               .......... .......... .......... .......... .......... .......... .......... ..........
FCC2              .......... .......... .......... .......... .......... .......... .......... ..........
HB3               .......... .......... .......... .......... .......... .......... .......... ..........
7G8               .......... .......... .......... .......... .......... .......... .......... ..........
P.reichenowi      .......... .......... .......... .......... ....G..... ....T..... .......... ..........

                         730        740        750        760        770        780        790        800
                  ....|....| ....|....| ....|....| ....|....| ....|....| ....|....| ....|....| ....|....|
PF10_0352         GGAGGTGGTG CTCCAACGTA TAAACCCGAG AACAATAAGA ACGATAATAT TTTGCTAGAA CACGTAAAAA TTACCTCGTG
3D7               .......... .......... .......... .......... .......... .......... .......... ..........
RO33              .......... .......... .......... .......... .......... .......... .......... ..........
Palo Alto         .......... .......... .......... .......... .......... .......... .......... ..........
FCR3              .......... .......... .......... .......... .......... .......... .......... ..........
Wellcome          .......... .......... .......... .......... .......... .......... .......... ..........
D6                .......... .......... .......... .......... .......... .......... .......... ..........
T996              .......... .......... .......... .......... .......... .......... .......... ..........
T9102             .......... .......... .......... .......... .......... .......... .......... ..........
K1                .......... .......... .......... .......... .......... .......... .......... ..........
Dd2               .......... .......... .......... .......... .......... .......... .......... ..........
D10               .......... .......... .......... .......... .......... .......... .......... ..........
FCC2              .......... .......... .......... .......... .......... .......... .......... ..........
HB3               .......... .......... .......... .......... .......... .......... .......... ..........
7G8               .......... .......... .......... .......... .......... .......... .......... ..........
P.reichenowi      .....C.... .......... .........A .......... .......... .......... ..AA...... .......A..

                         810        820        830        840        850        860        870        880
                  ....|....| ....|....| ....|....| ....|....| ....|....| ....|....| ....|....| ....|....|
PF10_0352         GGATAAAGAA GATATAATTA AAGAAAATGA AGACACAAAA CGCGAAGTTC AAGAAACTGA AGACACTGAC GAAACTGAAG
3D7               .......... .......... .......... .......... .......... .......... .......... ..........
RO33              .......... .......... .......... .......... .......... .......... .......... ..........
Palo Alto         .......... .......... .......... .......... .......... .......... .......... ..........
FCR3              .......... .......... .......... ...T...... .......... .......... .......... ..........
Wellcome          .......... .......... .......... ...T...... .......... .......... .......... ..........
D6                .......... .......... .......... .......... .......... .......... .......... ..........
T996              .......... .......... .......... .......... .......... .......... .......... ..........
T9102             .......... .......... .......... .......... .......... .......... .......... ..........
K1                .......... .......... .......... .......... .......... .......... .......... ..........
Dd2               .......... .......... .......... .......... .......... .......... .......... ..........
D10               .......... .......... .......... .......... .......... .......... .......... ..........
FCC2              .......... .......... .......... .......... .......... .......... .......... ..........
HB3               .......... .......... .......... .......... .......... .......... .......... ..........
7G8               .......... .......... .......... .......... .......... .......... .......... ..........
P.reichenowi      .......... .......... .......... .........G .T....A... .......... C.TA.....T ........C.

                         890        900        910        920        930        940        950        960
                  ....|....| ....|....| ....|....| ....|....| ....|....| ....|....| ....|....| ....|....|
PF10_0352         ATACTGACGA AACTGAAGAA ACAGAAGATA ---------T GGAAGATGAA AACGAAATTG TGGAAGATCA ATTACAAGAA
3D7               .......... .......... .......... ---------. .......... .......... .......... ..........
RO33              .......... .......... .......... ---------. .......... .......... .......... ..........
Palo Alto         .......... .......... .......... ---------. .......... .......... .......... ..........
FCR3              .......... .......... .......... ---------. .......... .......... .......... ..........
Wellcome          .......... .......... .......... ---------. .......... .......... .......... ..........
D6                .......... .......... .......... ---------. .......... .......... .......... ..........
T996              .......... .......... .......... ---------. .......... .......... .......... ..........
T9102             .......... .......... .......... ---------. .......... .......... .......... ..........
K1                .......... .......... .......... ---------. .......... .......... .......... ..........
Dd2               .......... .......... .......... ---------. .......... .......... .......... ..........
D10               .......... .......... .......... ---------. .......... .......... .......... ..........
FCC2              .......... .......... .......... ---------. .......... .......... .......... ..........
HB3               .......... .......... .......... ---------. .......... .......... .......... ..........
7G8               .......... .......... .......... ---------. .......... .......... .......... ..........
P.reichenowi      .A.....A.. .......... .......... ATTTAGATA. ....A..... .......... ........G. ..........
```

```
                       970       980       990       1000      1010      1020      1030      1040
                ....|....| ....|....| ....|....| ....|....| ....|....| ....|....| ....|....| ....|....|
PF10_0352       AATGAAGATG ATGAGGATAA TGTAAATTTA GAAGATATTA ATAAAAATAC TAGAAATGAT ATATTTGAAG AACAAATAAA
3D7             .......... .......... .......... .......... .......... .......... .......... ..........
RO33            .......... .......... .......... .......... .......... .......... .......... ..........
Palo Alto       .......... .......... .......... .......... .......... .......... .......... ..........
FCR3            .......... .......... .......... .......... .......... .......... .......... ..........
Wellcome        .......... .......... .......... .......... .......... .......... .......... ..........
D6              .......... .......... .......... .......... .......... .......... .......... ..........
T996            .......... .......... .......... .......... .......... .......... .......... ..........
T9102           .......... .......... .......... .......... .......... .......... .......... ..........
K1              .......... .......... .......... .......... .......... .......... .......... ..........
Dd2             .......... .......... .......... .......... .......... .......... .......... ..........
D10             .......... .......... .......... .......... .......... .......... .......... ..........
FCC2            .......... .......... .......... .......... .......... .......... .......... ..........
HB3             .......... .......... .......... .......... .......... .......... .......... ..........
7G8             .......... .......... .......... .......... .......... .......... .......... ..........
P.reichenowi    .........A .......... .......... .......... .........T ..A....A.. .......... ..........

                       1050      1060      1070      1080      1090      1100      1110      1120
                ....|....| ....|....| ....|....| ....|....| ....|....| ....|....| ....|....| ....|....|
PF10_0352       ATTAGATTCT ACGCAAGATG ACAAAGCTCA AAAATTAATT TCTAATGAAT ATAAAAAAAC TGAAGAAAAA AAATCATTAG
3D7             .......... .......... .......... .......... .......... .......... .......... ..........
RO33            .......... .......... .......... .......... .......... .......... .......... ..........
Palo Alto       .......... .......... .......... .......... .......... .......... .......... ..........
FCR3            .......... .......... .......... .......... .......... .......... .......... ..........
Wellcome        .......... .......... .......... .......... .......... .......... .......... ..........
D6              .......... .......... .......... .......... .......... .......... .......... ..........
T996            .......... .......... .......... .......... .......... .......... .......... ..........
T9102           .......... .......... .......... .......... .......... .......... .......... ..........
K1              .......... .......... .......... .......... .......... .......... .......... ..........
Dd2             .......... .......... .......... .......... .......... .......... .......... ..........
D10             .......... .......... .......... .......... .......... .......... .......... ..........
FCC2            .......... .......... .......... .......... .......... .......... .......... ..........
HB3             .......... .......... .......... .......... .......... .......... .......... ..........
7G8             .......... .......... .......... .......... .......... .......... .......... ..........
P.reichenowi    .......... .......... .......... .......... .....C.... .......... ....A..... ..T.......

                       1130      1140      1150      1160      1170      1180      1190      1200
                ....|....| ....|....| ....|....| ....|....| ....|....| ....|....| ....|....| ....|....|
PF10_0352       AAGATCATGT AAATCTATTA TTTAATTTTT TACAAACAAA TAACCAACTA GATCCTTCAC TAAAAGATTT AGAAAATGAG
3D7             .......... .......... .......... .......... .......... .......... .....
RO33            .......... .......... .......... .......... .......... .......... .....
Palo Alto       .......... .......... .......... .......... .......... .......... .....
FCR3            .......... .......... .......... .......... .......... .......... .....
Wellcome        .......... .......... .......... .......... .......... .......... .....
D6              .......... .......... .......... .......... .......... .......... .....
T996            .......... .......... .......... .......... .......... .......... .....
T9102           .......... .......... .......... .......... .......... .......... .....
K1              .......... .......... .......... .......... .......... .......... .....
Dd2             .......... .......... .......... .......... .......... .......... .....
D10             .......... .......... .......... .......... .......... .......... .....
FCC2            .......... .......... .......... .......... .......... .......... .....
HB3             .......... .......... .......... .......... .......... .......... .....
7G8             .......... .......... .......... .......... .......... .......... .....
P.reichenowi    .......... .......... .......... .......... G......... .......... .....

                      1210      1220
                ....|....| ....|....| ....|..
PF10_0352       TTAACTTTTT TTTTAAATAA CTATTGA
3D7
RO33
Palo Alto
FCR3
Wellcome
D6
T996
T9102
K1
Dd2
D10
FCC2
HB3
7G8
P.reichenowi
```

MSP7

```
                       10         20         30         40         50         60         70         80
               ....|....| ....|....| ....|....| ....|....| ....|....| ....|....| ....|....| ....|....|
PF13_0197      ATGAAGAGTA ATATCATATT TTATTTTTCT TTTTTTTTTG TGTACTTATA CTATGTTTCG TGTAATCAAT CAACTCATAG
3D7            .......... .......... .......... .......... .......... .......... .......... ..........
RO33           .......... .......... .......... .......... .......... .......... .......... ..........
Palo Alto      .......... .......... .......... .......... .......... .......... .......... ..........
FCR3           .......... .......... .......... .......... .......... .......... .......... ..........
Wellcome       .......... .......... .......... .......... .......... .......... .......... ..........
D6             .......... .......... .......... .......... .......... .......... .......... ..........
T996           .......... .......... .......... .......... .......... .......... .......... ..........
T9102          .......... .......... .......... .......... .......... .......... .......... ..........
K1             .......... .......... .......... .......... .......... .......... .......... ..........
Dd2            .......... .......... .......... .......... .......... .......... .......... ..........
D10            .......... .......... .......... .......... .......... .......... .......... ..........
FCC2           .......... .......... .......... .......... .......... .......... .......... ..........
HB3            .......... .......... .......... .......... .......... .......... .......... ..........
7G8            .......... .......... .......... .......... .......... .......... .......... ..........
P.reichenowi   .......... .......... .......... .......... .......... .......... .......... .....A....

                       90        100        110        120        130        140        150        160
               ....|....| ....|....| ....|....| ....|....| ....|....| ....|....| ....|....| ....|....|
PF13_0197      TACACCAGTA AATAATGAAG AAGATCAAGA AGAATTATAT ATTAAAAATA AAAAATTGGA AAAACTAAAA AATATAGTAT
3D7            .......... .......... .......... .......... .......... .......... .......... ..........
RO33           .......... .......... .......... .......... .......... .......... .......... ..........
Palo Alto      .......... .......... .......... .......... .......... .......... .......... ..........
FCR3           .......... .......... .......... .......... .......... .......... .......... ..........
Wellcome       .......... .......... .......... .......... .......... .......... .......... ..........
D6             .......... .......... .......... .......... .......... .......... .......... ..........
T996           .......... .......... .......... .......... .......... .......... .......... ..........
T9102          .......... .......... .......... .......... .......... .......... .......... ..........
K1             .......... .......... .......... .......... .......... .......... .......... ..........
Dd2            .......... .......... .......... .......... .......... .......... .......... ..........
D10            .......... .......... .......... .......... .......... .......... .......... ..........
FCC2           .......... .......... .......... .......... .......... .......... .......... ..........
HB3            .......... .......... .......... .......... .......... .......... .......... ..........
7G8            .......... .......... .......... .......... .......... .......... .......... ..........
P.reichenowi   .......... ........T. .......... .......... .......... .......... .......... ..........

                      170        180        190        200        210        220        230        240
               ....|....| ....|....| ....|....| ....|....| ....|....| ....|....| ....|....| ....|....|
PF13_0197      CAGGAGATTT TGTTGGAAAT TATAAAAATA ATGAAGAATT ATTAAACAAA AAAATTGAAG AATTACAAAA CAGTAAAGAA
3D7            .......... .......... .......... .......... .......... .......... .......... ..........
RO33           .......... .......... .......... .......... .......... .......... .......... ..........
Palo Alto      .......... .......... .......... .......... .......... .......... .......... ..........
FCR3           .......... .......... .......... .......... .......... .......... .......... ..........
Wellcome       .......... .......... .......... .......... .......... .......... .......... ..........
D6             .......... .......... .......... .......... .......... .......... .......... ..........
T996           .......... .......... .......... .......... .......... .......... .......... ..........
T9102          .......... .......... .......... .......... .......... .......... .......... ..........
K1             .......... .......... .......... .......... .......... .......... .......... ..........
Dd2            .......... .......... .......... .......... .......... .......... .......... ..........
D10            .......... .......... .......... .......... .......... .......... .......... ..........
FCC2           .......... .......... .......... .......... .......... .......... .......... ..........
HB3            .......... .......... .......... .......... .......... .......... .......... ..........
7G8            .......... .......... .......... .......... .......... .......... .......... ..........
P.reichenowi   .......... .......... .......... .......... ......T... .....C.... ..G....... ..........

                      250        260        270        280        290        300        310        320
               ....|....| ....|....| ....|....| ....|....| ....|....| ....|....| ....|....| ....|....|
PF13_0197      AAAAATGTAC ATGTATTAAT TAATGGAAAT TCAATTATTG ATGAAATAGA AAAAAATGAA GAAAATGATG ATAACGAAGA
3D7            .......... .......... .......... .......... .......... .......... .......... ..........
RO33           .......... .......... .......... .......... .......... .......... .......... ..........
Palo Alto      .......... .......... .......... .......... .......... .......... .......... ..........
FCR3           .......... .......... .......... .......... .......... .......... .......... ..........
Wellcome       .......... .......... .......... .......... .......... .......... .......... ..........
D6             .......... .......... .......... .......... .......... .......... .......... ..........
T996           .......... .......... .......... .......... .......... .......... .......... ..........
T9102          .......... .......... .......... .......... .......... .......... .......... ..........
K1             .......... .......... .......... .......... .......... .......... .......... ..........
Dd2            .......... .......... .......... .......... .......... .......... .......... ..........
D10            .......... .......... .......... .......... .......... .......... .......... ..........
FCC2           .......... .......... .......... .......... .......... .......... .......... ..........
HB3            .......... .......... .......... .......... .......... .......... .......... ..........
7G8            .......... .......... .......... .......... .......... .......... .......... ..........
P.reichenowi   .......... .......... .......... .......... .......... .......... .......... ..........
```

```
                       330       340       350       360       370       380       390       400
               ....|....| ....|....| ....|....| ....|....| ....|....| ....|....| ....|....| ....|....|
PF13_0197      AAATAATGAT GATGACAATA CATATGAATT AGATATGAAT GATGACACAT TCTTAGGACA AAATAACGAT TCACATTTTG
3D7            .......... .......... .......... .......... .......... .......... .......... ..........
RO33           .......... .......... .......... .......... .......... .......... .......... ..........
Palo Alto      .......... .......... .......... .......... .......... .......... .......... ..........
FCR3           .......... .......... .......... .......... .......... .......... .......... ..........
Wellcome       .......... .......... .......... .......... .......... .......... .......... ..........
D6             .......... .......... .......... .......... .......... .......... .......... ..........
T996           .......... .......... .......... .......... .......... .......... .......... ..........
T9102          .......... .......... .......... .......... .......... .......... .......... ..........
K1             .......... .......... .......... .......... .......... .......... .......... ..........
Dd2            .......... .......... .......... .......... .......... .......... .......... ..........
D10            .......... .......... .......... .......... .......... .......... .......... ..........
FCC2           .......... .......... .......... .......... .......... .......... .......... ..........
HB3            .......... .......... .......... .......... .......... .......... .......... ..........
7G8            .......... .......... .......... .......... .......... .......... .......... ..........
P.reichenowi   .......... .......... .......... .......... .......... .......... .......... ..........

                       410       420       430       440       450       460       470       480
               ....|....| ....|....| ....|....| ....|....| ....|....| ....|....| ....|....| ....|....|
PF13_0197      AAAATGTTGA TGATGACGCA GTAGAAAATG AACAAGAAGA TGAAAACAAG GAAAAATCAG AATCATTTCC ATTATTCCAA
3D7            .......... .......... .......... .......... .......... .......... .......... ..........
RO33           .......... .......... .......... .......... .......... .......... .......... ..........
Palo Alto      .......... .......... .......... .......... .......... .......... .......... ..........
FCR3           .......... .......... .......... .......... .......... .......... .......... ..........
Wellcome       .......... .......... .......... .......... .......... .......... .......... ..........
D6             .......... .......... .......... .......... .......... .......... .......... ..........
T996           .......... .......... .......... .......... .......... .......... .......... ..........
T9102          .......... .......... .......... .......... .......... .......... .......... ..........
K1             .......... .......... .......... .......... .......... .......... .......... ..........
Dd2            .......... .......... .......... .......... .......... .......... .......... ..........
D10            .......... .......... .......... .......... .......... .......... .......... ..........
FCC2           .......... .......... .......... .......... .......... .......... .......... ..........
HB3            .......... .......... .......... .......... .......... .......... .......... ..........
7G8            .......... .......... .......... .......... .......... .......... .......... ..........
P.reichenowi   .......... .......T.. .A........ .......... ....G.T... .......... .......... ..........

                       490       500       510       520       530       540       550       560
               ....|....| ....|....| ....|....| ....|....| ....|....| ....|....| ....|....| ....|....|
PF13_0197      AATTTAGGAT TATTCGGTAA AAACGTATTA TCAAAGGTAA AGGCACAAAG TGAAACAGAT ACTCAATCTA AAAATGAACA
3D7            .......... .......... .......... .......... .......... .......... .......... ..........
RO33           .......... .......... .......... .......... .......... .......... .......... ..........
Palo Alto      .......... .......... .......... .......... .......... .......... .......... ..........
FCR3           .......... .......... .......... .......... .......... .......... .......... ..........
Wellcome       .......... .......... .......... .......... .......... .......... .......... ..........
D6             .......... .......... .......... .......... .......... .......... .......... ..........
T996           .......... .......... .......... .......... .......... .......... .......... ..........
T9102          .......... .......... .......... .......... .......... .......... .......... ..........
K1             .......... .......... .......... .......... .......... .......... .......... ..........
Dd2            .......... .......... .......... .......... .......... .......... .......... ..........
D10            .......... .......... .......... .......... .......... .......... .......... ..........
FCC2           .......... .......... .......... .......... .......... .......... .......... ..........
HB3            .......... .......... .......... .......... .......... .......... .......... ..........
7G8            .......... .......... .......... .......... .......... .......... .......... ..........
P.reichenowi   .......... .......... ......G... .......... .......... ........T. .G........ .T....G...

                       570       580       590       600       610       620       630       640
               ....|....| ....|....| ....|....| ....|....| ....|....| ....|....| ....|....| ....|....|
PF13_0197      AGAGATATCA ACACAA---- ---------- ---------- ---------G GACAAGAAGT ACAAAAACCA GCACAAGGAG
3D7            .......... ......---- ---------- ---------- ---------. .......... .......... ..........
RO33           .......... ......---- ---------- ---------- ---------. ..A....... .......... ..........
Palo Alto      .......... ......---- ---------- ---------- ---------. .......... .......... ..........
FCR3           .......... ......---- ---------- ---------- ---------. ..A....... .......... ..........
Wellcome       .......... ......---- ---------- ---------- ---------. ..A....... .......... ..........
D6             .......... ......---- ---------- ---------- ---------. .......... .......... ..........
T996           .......... ......---- ---------- ---------- ---------. .......... .......... ..........
T9102          .......... ......---- ---------- ---------- ---------. ..A....... .......... ..........
K1             .......... ......---- ---------- ---------- ---------. .......... .......... ..........
Dd2            .......... ......---- ---------- ---------- ---------. .....A.... .......... ..........
D10            .......... ......---- ---------- ---------- ---------. .......... .......... ..........
FCC2           .......... ......---- ---------- ---------- ---------. .......... .......... ..........
HB3            .......... ......---- ---------- ---------- ---------. .......... .......... ..........
7G8            .......... ......---- ---------- ---------- ---------. .......... .......... ..........
P.reichenowi   ...AG..CA. ....C.ACAC AAAGACAAAA TGTACAAACA CCAACACCA. .....A.T.. .....C.... A......T..
```

```
                             650        660        670        680        690        700        710        720
                      ....|....| ....|....| ....|....| ....|....| ....|....| ....|....| ....|....| ....|....|
PF13_0197             GAGAATCGAC ATTTCAAAAA GACCTAGATA AGAAATTATA TAATTTAGGA GATGTTTTTA ATCATGTAGT TGATATTTCA
3D7                   .......... .......... .......... .......... .......... .......... .......... ..........
RO33                  .......... .....G.... .......T.. .A........ .......... .......... .......... ..........
Palo Alto             .......... .......... .......... .......... .......... .......... .......... ..........
FCR3                  .......... .....G.... .......... .......... .......... .......... .......... ..........
Wellcome              .......... .....G.... .......... .......... .......... .......... .......... ..........
D6                    .......... .......... .......... .......... .......... .......... .......... ..........
T996                  .......... .......... .......... .......... .......... .......... .......... ..........
T9102                 .......... .....G.... .......... .......... .......... .......... .......... ..........
K1                    .......... .....G.... .......... .......... .......... .......... .......... ..........
Dd2                   .......... .......... .......T.. .A........ .......... .......... .......... ..........
D10                   .......... .......... .......... .......... .......... .......... .......... ..........
FCC2                  .......... .......... .......... .......... .......... .......... .......... ..........
HB3                   .......... .....G.... .......T.. .A........ .......... .......... .......... ..........
7G8                   .......... .......... .......... .......... .......... .......... .......... ..........
P.reichenowi          C.A....C.. ....G..... ..T.C.AC.. .......... .......... ........G. .....A.... .C........

                             730        740        750        760        770        780        790        800
                      ....|....| ....|....| ....|....| ....|....| ....|....| ....|....| ....|....| ....|....|
PF13_0197             AACAAAAAGA ACAAAATAAA TCTCGATGAA TATGGTAAAA AATATACAGA TTTCAAAAAA GAATATGAAG ACTTCGTTTT
3D7                   .......... .......... .......... .......... .......... .......... .......... ..........
RO33                  ...G..G... .......... .......A.. .........G .......... .......... .......... ..........
Palo Alto             .......... .......... .A.....A.. .........G .......... .......... .......... ..........
FCR3                  ......G... .......... .......... ....A..... .......... .......... .......... ..........
Wellcome              ......G... .......... .......... ....A..... .......... .......... .......... ..........
D6                    .......... .......... .......... C...A..... .......... .......... .......... ..........
T996                  .......... .......... .......... C...A..... .......... .......... .......... ..........
T9102                 ......G... .......... .......... ....A..... .......... .......... .......... ..........
K1                    ...G..G... .......... .......A.. .......... .......... .......... .......... ..........
Dd2                   ...G..G... .......... .......A.. .........G .......... .......... .......... ..........
D10                   ......G... .......... .......... .......... .......... .......... .......... ..........
FCC2                  ...G..G... .......... .......A.. .........G .......... .......... .......... ..........
HB3                   ...G..G... .......... .......A.. .........G .......... .......... .......... ..........
7G8                   .......... .......... .......... .......... .......... .......... .......... ..........
P.reichenowi          ......G.A. .......... .A..AT.A.T ......G... .....C.... .......... .......... ..........

                             810        820        830        840        850        860        870        880
                      ....|....| ....|....| ....|....| ....|....| ....|....| ....|....| ....|....| ....|....|
PF13_0197             AAATTCTAAA GAATATGATA TAATCAAAAA TCTAATAATT ATGTTTGGTC AAGAAGATAA TAAGAGTAAA AATGGCAAAA
3D7                   .......... .......... .......... .......... .......... .......... .......... ..........
RO33                  .......... .......... .......... .......... .......... .......... .......... ..........
Palo Alto             .......... .......... .......... .......... .......... .......... .......... ..........
FCR3                  .......... .......... .......... .......... .......... .......... .......... ..........
Wellcome              .......... .......... .......... .......... .......... .......... .......... ..........
D6                    .......... .......... .......... .......... .......... .......... .......... ..........
T996                  .......... .......... .......... .......... .......... .......... .......... ..........
T9102                 .......... .......... .......... .......... .......... .......... .......... ..........
K1                    .......... .......... .......... .......... .......... .......... .......... ..........
Dd2                   .......... .......... .......... .......... .......... .......... .......... ..........
D10                   .......... .......... .......... .......... .......... .......... .......... ..........
FCC2                  .......... .......... .......... .......... .......... .......... .......... .C........
HB3                   .......... .......... .......... .......... .......... .......... .......... ..........
7G8                   .......... .......... .......... .......... .......... .......... .......... ..........
P.reichenowi          .......... .......... .......... .......... .......... .......... .........T .....AC...

                             890        900        910        920        930        940        950        960
                      ....|....| ....|....| ....|....| ....|....| ....|....| ....|....| ....|....| ....|....|
PF13_0197             CGGATATTGT AAGTGAAGCT AAACATATGA CTGAAATTTT CATAAAACTA TTTAAAGATA AGGAATACCA TGAACAATTT
3D7                   .......... .......... .......... .......... .......... .......... .......... ..........
RO33                  ....G..... .......... .......... T......... .......... .......... .......... ..........
Palo Alto             .......... .......... .......... .......... .......... .......... .......... ..........
FCR3                  .......... .......... .......... .......... .......... .......... .......... ..........
Wellcome              .......... .......... .......... .......... .......... .......... .......... ..........
D6                    .......... .......... .......... .......... .......... .......... .......... ..........
T996                  .......... .......... .......... .......... .......... .......... .......... ..........
T9102                 .......... .......... .......... .......... .......... .......... .......... ..........
K1                    .......... .......... .......... T......... .......... .......... .......... ..........
Dd2                   ..T....... .......... .......... T......... .......... .......... .......... ..........
D10                   ..T....... .......... .........G T......... .......... .......... .......... ..........
FCC2                  .......... .......... ......---. T......... .......... .......... .......... ..........
HB3                   ....G..... .......... .......... T......... .......... .....T.... .......... ..........
7G8                   .......... .......... .......... .......... .......... .......... .......... ..........
P.reichenowi          ...G....T. .G.......A .......... .......... .......T.. ..AG.C...G ..A....... .C........
```

```
                                970        980        990        1000       1010       1020       1030       1040
                        ....|....| ....|....| ....|....| ....|....| ....|....| ....|....| ....|....| ....|....|
PF13_0197               AAAAATTATA TTTATGGTGT TTATAGTTAT GCAAAACAAA ATAGTCACTT AAGTGAGAAA AAAATAAAAC CAGAAGAGGA
3D7                     .......... .......... .......... .......... .......... .......... .......... ..........
RO33                    .......... .......... .......... .......... .......... .......... .......... ..........
Palo Alto               .......... .......... .......... .......... .......... .......... .......... ..........
FCR3                    .......... .......... .......... .......... .......... .......... .......... ..........
Wellcome                .......... .......... .......... .......... .......... .......... .......... ..........
D6                      .......... .......... .......... .......... .......... .......... .......... ..........
T996                    .......... .......... .......... .......... .......... .......... .......... ..........
T9102                   .......... .......... .......... .......... .......... .......... .......... ..........
K1                      .......... .......... .......... .......... .......... .......... .......... ..........
Dd2                     .......... .......... .......... .......... .......... .......... .......... ..........
D10                     .......... .......... .......... .......... .......... .......... .......... ..........
FCC2                    .......... .......... .......... .......... .......... .......... .......... A.........
HB3                     .......... .......... .......... .......... .......... .......... .......... ..........
7G8                     .......... .......... .......... .......... .......... .......... .......... ..........
P.reichenowi            .......T.. .......... .......... .......... .......... .......... .......... .T...A.T..

                                1050       1060       1070       1080
                        ....|....| ....|....| ....|....| ....|....| ....|....
PF13_0197               ATATAAAAAA TTCTTAGAAT ATTCATTTAA TTTACTAAAC ACAATGTAA
3D7                     .......... .......... .......... ..........
RO33                    .......... .......... .......... ..........
Palo Alto               .......... .......... .......... ..........
FCR3                    .......... .......... .......... ..........
Wellcome                .......... .......... .......... ..........
D6                      .......... .......... .......... ..........
T996                    .......... .......... .......... ..........
T9102                   .......... .......... .......... ..........
K1                      .......... .......... .......... ..........
Dd2                     .......... .......... .......... ..........
D10                     .......... .......... .......... ..........
FCC2                    .......... .......... .......... ..........
HB3                     .......... .......... .......... ..........
7G8                     .......... .......... .......... ..........
P.reichenowi            .......... .......... .......... ..........
```

MRSP1

```
                                 10         20         30         40         50         60         70         80
                        ....|....| ....|....| ....|....| ....|....| ....|....| ....|....| ....|....| ....|....|
PF13_0196               ATGAAAAGAC AAATTATATT GTTTACTTCT TTATTTATAT TTTCTCTTAA TTTAACGTTG TCATATGAAA AATCAAATAA
3D7                     .......... .......... .......... .......... .......... .......... .......... ..........
RO33                    .......... .......... .......... .......... .......... .......... .......... ..........
Palo Alto               .......... .......... .......... .......... .......... .......... .......... ..........
FCR3                    .......... .......... .......... .......... .......... .......... .......... ..........
Wellcome                .......... .......... .......... .......... .......... .......... .......... ..........
D6                      .......... .......... .......... .......... .......... .......... .......... ..........
T996                    .......... .......... .......... .......... .......... .......... .......... ..........
T9102                   .......... .......... .......... .......... .......... .......... .......... ..........
K1                      .......... .......... .......... .......... .......... .......... .......... ..........
Dd2                     .......... .......... .......... .......... .......... .......... .......... ..........
D10                     .......... .......... .......... .......... .......... .......... .......... ..........
FCC2                    .......... .......... .......... .......... .......... .......... .......... ..........
HB3                     .......... .......... .......... .......... .......... .......... .......... ..........
7G8                     .......... .......... .......... .......... .......... .......... .......... ..........
P.reichenowi            .......... .......... ....G..... .......... .......... .......... .......... ..........

                                90        100        110        120        130        140        150        160
                        ....|....| ....|....| ....|....| ....|....| ....|....| ....|....| ....|....| ....|....|
PF13_0196               TAGATTAGGA TATTATAATG AAGAAAATGC AGACAATAAG GAGAAGAATA AAATTACTTT CATGTTTAAC GATATTAAGG
3D7                     .......... .......... .......... .......... .......... .......... .......... ..........
RO33                    .......... .......... .......... .......... .......... .......... .......... ..........
Palo Alto               .......... .......... .......... .......... .......... .......... .......... ..........
FCR3                    .......... .......... .......... .......... .......... .......... .......... ..........
Wellcome                .......... .......... .......... .......... .......... .......... .......... ..........
D6                      .......... .......... .......... .......... .......... .......... .......... ..........
T996                    .......... .......... .......... .......... .......... .......... .......... ..........
T9102                   .......... .......... .......... .......... .......... .......... .......... ..........
K1                      .......... .......... .......... .......... .......... .......... .......... ..........
Dd2                     .......... .......... .......... .......... .......... .......... .......... ..........
D10                     .......... .......... .......... .......... .......... .......... .......... ..........
FCC2                    .......... .......... .......... .......... .......... .......... .......... ..........
HB3                     .......... .......... .......... .......... .......... .......... .......... ..........
7G8                     .......... .......... .......... .......... .......... .......... .......... ..........
P.reichenowi            .......... .......... .......... .......... .......... .........C .......... ..........
```

```
                           170        180        190        200        210        220        230        240
                    ....|....| ....|....| ....|....| ....|....| ....|....| ....|....| ....|....| ....|....|
PF13_0196           TAGGTATGCA AAATGATGTG GAAGAAAATT TAGATAATGG AAAAAATGTG TTAGAAGATG AACATTTGGT TATAGGGCAA
3D7                 .......... .......... .......... .......... .......... .......... .......... ..........
RO33                .......... .......... .......... .......... .......... .......... .......... ..........
Palo Alto           .......... .......... .......... .......... .......... .......... .......... ..........
FCR3                .......... .......... .......... .......... .......... .......... .......... ..........
Wellcome            .......... .......... .......... .......... .......... .......... .......... ..........
D6                  .......... .......... .......... .......... .......... .......... .......... ..........
T996                .......... .......... .......... .......... .......... .......... .......... ..........
T9102               .......... .......... .......... .......... .......... .......... .......... ..........
K1                  .......... .......... .......... .......... .......... .......... .......... ..........
Dd2                 .......... .......... .......... .......... .......... .......... .......... ..........
D10                 .......... .......... .......... .......... .......... .......... .......... ..........
FCC2                .......... .......... .......... .......... .......... .......... .......... ..........
HB3                 .......... .......... .......... .......... .......... .......... .......... ..........
7G8                 .......... .......... .......... .......... .......... .......... .......... ..........
P.reichenowi        ...A...A.. ...C...... .......... .......... .......... .......... .......... ..........

                           250        260        270        280        290        300        310        320
                    ....|....| ....|....| ....|....| ....|....| ....|....| ....|....| ....|....| ....|....|
PF13_0196           GAAGGACAAA CCGACAATGA GGGTCCTCAT ACAGAAACTA AACCTGAAAG ATCTGTTGAA TCAGGAGGTA CAGGCGATAG
3D7                 .......... .......... .......... .......... .......... .......... .......... ..........
RO33                .......... .......... .......... .........G .......... .......... .......... ..........
Palo Alto           .......... .......... .......... .........G .......... .......... .......... ..........
FCR3                .......... .......... .......... .........G .......... .......... .......... ..........
Wellcome            .......... .......... .......... .........G .......... .......... .......... ..........
D6                  .......... .......... .......... .........G .......... .......... .......... ..........
T996                .......... .......... .......... .........G .......... .......... .......... ..........
T9102               .......... .......... .......... .........G .......... .......... .......... ..........
K1                  .......... .......... .......... .........G .......... .......... .......... ..........
Dd2                 .......... .......... .......... .........G .......... .......... .......... ..........
D10                 .......... .......... .......... .........G .......... .......... .......... ..........
FCC2                .......... .......... .......... .........G .......... .......... .......... ..........
HB3                 .......... .......... .......... .........G .......... .......... .......... ..........
7G8                 .......... .......... .......... .........G .......... .......... .......... ..........
P.reichenowi        .......... .GA....... ....T..... .......A.G ..T..C.... ........G. ......AC.. ....T.C...

                           330        340        350        360        370        380        390        400
                    ....|....| ....|....| ....|....| ....|....| ....|....| ....|....| ....|....| ....|....|
PF13_0196           CGTTTCAAAT CATGAATCGG AATTACCAAG AGATACAGAA ACTCGTGAAG TTGTAACAAC AGTACAACCA GGAACACAAT
3D7                 .......... .......... .......... .......... .......... .......... .......... ..........
RO33                .......... .......... .......... .......... .......... .......... .......... ..........
Palo Alto           .......... .......... .......... .......... .......... .......... .......... ..........
FCR3                .......... .......... .......... .......... .......... .......... .......... ..........
Wellcome            .......... .......... .......... .......... .......... .......... .......... ..........
D6                  .......... .......... .......... .......... .......... .......... .......... ..........
T996                .......... .......... .......... .......... .......... .......... .......... ..........
T9102               .......... .......... .......... .......... .......... .......... .......... ..........
K1                  .......... .......... .......... .......... .......... .......... .......... ..........
Dd2                 .......... .......... .......... .......... .......... .......... .......... ..........
D10                 .......... .......... .......... .......... .......... .......... .......... ..........
FCC2                .......... .......... .......... .......... .......... .......... .......... ..........
HB3                 .......... .......... .......... .......... .......... .......... .......... ..........
7G8                 .......... .......... .......... .......... .......... .......... .......... ..........
P.reichenowi        ....G..... .G........ .......... ..C....... C......... .......... .......... .T........

                           410        420        430        440        450        460        470        480
                    ....|....| ....|....| ....|....| ....|....| ....|....| ....|....| ....|....| ....|....|
PF13_0196           TAGGAGAATC AGGAGCAAAA GAAACATTAG AGTCACAAAC ATCACCAGCA CCACAGGGAA CATCAGGATT ACAAGGAGAA
3D7                 .......... .......... .......... .......... .......... .......... .......... ..........
RO33                .......... .......... .......... .......... .......... .......... .......... ..........
Palo Alto           .......... .......... .......... .......... .......... .......... .......... ..........
FCR3                .......... .......... .......... .......... .......... .......... .......... ..........
Wellcome            .......... .......... .......... .......... .......... .......... .......... ..........
D6                  .......... .......... .......... .......... .......... .......... .......... ..........
T996                .......... .......... .......... .......... .......... .......... .......... ..........
T9102               .......... .......... .......... .......... .......... .......... .......... ..........
K1                  .......... .......... .......... .......... .......... .......... .......... ..........
Dd2                 .......... .......... .......... .......... .......... .......... .......... ..........
D10                 .......... .......... .......... .......... .......... .......... .......... ..........
FCC2                .......... .......... .......... .......... .......... .......... .......... ..........
HB3                 .......... .......... .......... .......... .......... .......... .......... ..........
7G8                 .......... .......... .......... .......... .......... .......... .......... ..........
P.reichenowi        C......... ....A...T. .....T...C ........GA .....G..G. .......... .......... ..........
```

```
                         490        500        510        520        530        540        550        560
                  ....|....| ....|....| ....|....| ....|....| ....|....| ....|....| ....|....| ....|....|
PF13_0196         AAGGTACCAG GTGCCTTATC AAGTGTATCA CAAACGTCTA GTCCCGATGT ATCATCAAGA TCTGAACAAC CACAAACGAT
3D7               .......... .......... .......... .......... .......... .......... .......... ..........
RO33              .......... .......... .......... .......... .......... .......... .......... ..........
Palo Alto         .......... .......... .......... .......... .......... .......... .......... ..........
FCR3              .......... .......... .......... .......... .......... .......... .......... ..........
Wellcome          .......... .......... .......... .......... .......... .......... .......... ..........
D6                .......... .......... .......... .......... .......... .......... .......... ..........
T996              .......... .......... .......... .......... .......... .......... .......... ..........
T9102             .......... .......... .......... .......... .......... .......... .......... ..........
K1                .......... .......... .......... .......... .......... .......... .......... ..........
Dd2               .......... .......... .......... .......... .......... .......... .......... ..........
D10               .......... .......... .......... .......... .......... .......... .......... ..........
FCC2              .......... .......... .......... .......... .......... .......... .......... ..........
HB3               .......... .......... .......... .......... .......... .......... .......... ..........
7G8               .......... .......... .......... .......... .......... .......... .......... ..........
P.reichenowi      .G........ .......... .......... .......... A......... .......... .......... ........G.

                         570        580        590        600        610        620        630        640
                  ....|....| ....|....| ....|....| ....|....| ....|....| ....|....| ....|....| ....|....|
PF13_0196         GCCAGAATCA CCAAGACCAG AAGGAACATC AACCGAATCA CAACCAAGAG GATCGACAGA ATCAGATGCA AGT------A
3D7               .......... .......... .......... .......... .......... .......... .......... ...------.
RO33              .......... .......... .......... .......... .......... .......... .......... ...------.
Palo Alto         .......... .......... .......... .......... .......... .......... .......... ...------.
FCR3              .......... .......... .......... .......... .......... .......... .......... ...------.
Wellcome          .......... .......... .......... .......... .......... .......... .......... ...------.
D6                .......... .......... .......... .......... .......... .......... .......... ...------.
T996              .......... .......... .......... .......... .......... .......... .......... ...------.
T9102             .......... .......... .......... .......... .......... .......... .......... ...------.
K1                .......... .......... .......... .......... .......... .......... .......... ...------.
Dd2               .......... .......... .......... .......... .......... .......... .......... ...------.
D10               .......... .......... .......... .......... .......... .......... .......... ...------.
FCC2              .......... .......... .......... .......... .......... .......... .......... ...------.
HB3               .......... .......... .......... .......... .......... .......... .......... ...------.
7G8               .......... .......... .......... .......... .......... .......... .......... ...------.
P.reichenowi      .......... .......... .......... ...AC..... .........A .......... ...C...... TC.GCAGAT.

                         650        660        670        680        690        700        710        720
                  ....|....| ....|....| ....|....| ....|....| ....|....| ....|....| ....|....| ....|....|
PF13_0196         ATAAAAATGG TTCCCAAACT AATGTTAGGA CAATTTCAAA TTCTAGTAAT AGTATCACAT CTCCTCAAAC GACACAACCT
3D7               .......... .......... .......... .......... .......... .......... .......... ..........
RO33              .......... .......... .......... .......... .......... .......... .......... ..........
Palo Alto         .......... .......... .......... .......... .......... .......... .......... ..........
FCR3              .......... .......... .......... .......... .......... .......... .......... ..........
Wellcome          .......... .......... .......... .......... .......... .......... .......... ..........
D6                .......... .......... .......... .......... .......... .......... .......... ..........
T996              .......... .......... .......... .......... .......... .......... .......... ..........
T9102             .......... .......... .......... .......... .......... .......... .......... ..........
K1                .......... .......... .......... .......... .......... .......... .......... ..........
Dd2               .......... .......... .......... .......... .......... .......... .......... ..........
D10               .......... .......... .......... .......... .......... .......... .......... ..........
FCC2              .......... .......... .......... .......... .......... .......... .......... ..........
HB3               .......... .......... .......... .......... .......... .......... .......... ..........
7G8               .......... .......... .......... .......... .......... .......... .......... ..........
P.reichenowi      .......... .......... .......... .......... .......... .......... .......... .G........

                         730        740        750        760        770        780        790        800
                  ....|....| ....|....| ....|....| ....|....| ....|....| ....|....| ....|....| ....|....|
PF13_0196         AGTAATAATC AAAATAATAC AGTAAGTACA ACCTCAGAAG TGAAATATTT AGATCTCCTT TATGATGAAC TTCTTACAAA
3D7               .......... .......... .......... .......... .......... .......... .......... ..........
RO33              .......... .......... .......... .......... .......... .......... .......... ..........
Palo Alto         .......... .......... .......... .......... .......... .......... .......... ..........
FCR3              .......... .......... .......... .......... .......... .......... .......... ..........
Wellcome          .......... .......... .......... .......... .......... .......... .......... ..........
D6                .......... .......... .......... .......... .......... .......... .......... ..........
T996              .......... .......... .......... .......... .......... .......... .......... ..........
T9102             .......... .......... .......... .......... .......... .......... .......... ..........
K1                .......... .......... .......... .......... .......... .......... .......... ..........
Dd2               .......... .......... .......... .......... .......... .......... .......... ..........
D10               .......... .......... .......... .......... .......... .......... .......... ..........
FCC2              .......... .......... .......... .......... .......... .......... .......... ..........
HB3               .......... .......... .......... .......... .......... .......... .......... ..........
7G8               .......... .......... .......... .......... .......... .......... .......... ..........
P.reichenowi      .........G .......... .......... .......... .......... .......... ........T. ..........
```

```
                      810        820        830        840        850        860        870        880
              ....|....| ....|....| ....|....| ....|....| ....|....| ....|....| ....|....| ....|....|
PF13_0196     TAGCGAAGGA AAACATCAAG TTCATTTTGG AGAAAATCAC AAAAAATATA ATATTTTTAG AAAGCATTAT GATAATTTTG
3D7           .......... .......... .......... .......... .......... .......... .......... ..........
RO33          .......... .......... .......... .......... .......... .......... .......... ..........
Palo Alto     .......... .......... .......... .......... .......... .......... .......... ..........
FCR3          .......... .......... .......... .......... .......... .......... .......... ..........
Wellcome      .......... .......... .......... .......... .......... .......... .......... ..........
D6            .......... .......... .......... .......... .......... .......... .......... ..........
T996          .......... .......... .......... .......... .......... .......... .......... ..........
T9102         .......... .......... .......... .......... .......... .......... .......... ..........
K1            .......... .......... .......... .......... .......... .......... .......... ..........
Dd2           .......... .......... .......... .......... .......... .......... .......... ..........
D10           .......... .......... .......... .......... .......... .......... .......... ..........
FCC2          .......... .......... ..G....... .......... .......... .......... T......... ..........
HB3           .......... .......... .......... .......... .......... .......... .......... ..........
7G8           .......... .......... .......... .......... .......... .......... .......... ..........
P.reichenowi  .......... .......... ..G....... .......... .......... .......... .......... ...G......

                      890        900        910        920        930        940        950        960
              ....|....| ....|....| ....|....| ....|....| ....|....| ....|....| ....|....| ....|....|
PF13_0196     TCATTAATCA AAAAGAGTAT GATATAATTA AGAAACTTCT TAATTCTATT TTTAGAGATA ATAATGAAAA TGAAAAAATG
3D7           .......... .......... .......... .......... .......... .......... .......... ..........
RO33          .......... .......... .......... .......... .......... .......... .......... ..........
Palo Alto     .......... .......... .......... .......... .......... .......... .......... ..........
FCR3          .......... .......... .......... .......... .......... .......... .......... ..........
Wellcome      .......... .......... .......... .......... .......... .......... .......... ..........
D6            .......... .......... .......... .......... .......... .......... .......... ..........
T996          .......... .......... .......... .......... .......... .......... .......... ..........
T9102         .......... .......... .......... .......... .......... .......... .......... ..........
K1            .......... .......... .......... .......... .......... .......... .......... ..........
Dd2           .......... .......... .......... .......... .......... .......... .......... ..........
D10           .......... .......... .......... .......... .......... .......... .......... ..........
FCC2          .......... .......... .......... .......... .......... .......... .......... ..........
HB3           .......... .......... .......... .......... .......... .......... .......... ..........
7G8           .......... .......... .......... .......... .......... .......... .......... ..........
P.reichenowi  .......... .......... .......... .......... .......... .......... .......... ..........

                      970        980        990       1000       1010       1020       1030       1040
              ....|....| ....|....| ....|....| ....|....| ....|....| ....|....| ....|....| ....|....|
PF13_0196     AAAAATTTAG TCAGCATTTT TCAAAAAGCA TTAAATGACA AGAAATTTCA TGATCAATTT AAAAACTTTA TTTATGGTAT
3D7           .......... .......... .......... .......... .......... .......... .......... ..........
RO33          .......... .......... .......... .......... .......... .......... .......... ..........
Palo Alto     .......... .......... .......... .......... .......... .......... .......... ..........
FCR3          .......... .......... .......... .......... .......... .......... .......... ..........
Wellcome      .......... .......... .......... .......... .......... .......... .......... ..........
D6            .......... .......... .......... .......... .......... .......... .......... ..........
T996          .......... .......... .......... .......... .......... .......... .......... ..........
T9102         .......... .......... .......... .......... .......... .......... .......... ..........
K1            .......... .......... .......... .......... .......... .......... .......... ..........
Dd2           .......... .......... .......... .......... .......... .......... .......... ..........
D10           .......... .......... .......... .......... .......... .......... .......... ..........
FCC2          .......... .......... .......... .......... .......... .......... .......... ..........
HB3           .......... .......... .......... .......... .......... .......... .......... ..........
7G8           .......... .......... .......... .......... .......... .......... .......... ..........
P.reichenowi  .....G.... .....G.... .......... ....C..... .......... .......... .......... .CC.......

                     1050       1060       1070       1080       1090       1100       1110       1120
              ....|....| ....|....| ....|....| ....|....| ....|....| ....|....| ....|....| ....|....|
PF13_0196     TTATGGATTT GCAAAACGTC ATAGTTATTT AAGAAATGAA AAAATACAAA ACGATAGTGA ATATAAAGAA TTCTTTGAAA
3D7           .......... .......... .......... .......... .......... .......... .......... ..........
RO33          .......... .......... .......... .......... .......... .......... .......... ..........
Palo Alto     .......... .......... .......... .......... .......... .......... .......... ..........
FCR3          .......... .......... .......... .......... .......... .......... .......... ..........
Wellcome      .......... .......... .......... .......... .......... .......... .......... ..........
D6            .......... .......... .......... .......... .......... .......... .......... ..........
T996          .......... .......... .......... .......... .......... .......... .......... ..........
T9102         .......... .......... .......... .......... .......... .......... .......... ..........
K1            .......... .......... .......... .......... .......... .......... .......... ..........
Dd2           .......... .......... .......... .......... .......... .......... .......... ..........
D10           .......... .......... .......... .......... .......... .......... .......... ..........
FCC2          .......... .......... .......... .......... .......... .......... .......... ..........
HB3           .......... .......... .......... .......... .......... .......... .......... ..........
7G8           .......... .......... .......... .......... .......... .......... .......... ..........
P.reichenowi  .......... .......... .......... .......... .......... ....C..... .......... ..........
```

```
                          1130       1140
                     ....|....| ....|....| ....|....
PF13_0196            ATGCAGTGGA TTTATTAAAT ACACTTTGA
3D7                  .......... .......... .........
RO33                 .......... .......... .........
Palo Alto            .......... .......... .........
FCR3                 .......... .......... .........
Wellcome             .......... .......... .........
D6                   .......... .......... .........
T996                 .......... .......... .........
T9102                .......... .......... .........
K1                   .......... .......... .........
Dd2                  .......... .......... .........
D10                  .......... .......... .........
FCC2                 .......... .......... .........
HB3                  .......... .......... .........
7G8                  .......... .......... .........
P.reichenowi         .......... .......... .........
```

MRSP2

```
                             10         20         30         40         50         60         70         80
                     ....|....| ....|....| ....|....| ....|....| ....|....| ....|....| ....|....| ....|....|
MAL13P1.174          ATGAAGGGAC AAGCAATTTA TTTCGTTTTT GTTTTCTTAT ATTTTCTCAA TTGTGTGGGG TGCAATTCAA AGAATAATAA
3D7                           . .......... .......... .......... .......... .......... .......... ..........
RO33                          . .......... .......... .......... .......... .......... .......... ..........
PALO ALTO                     . .......... .......... .......... .......... .......... .......... ..........
FCR3                          . .......... .......... .......... .......... .......... .......... ..........
WELLCOME                      . .......... .......... .......... .......... .......... .......... ..........
D6                            . .......... .......... .......... .......... .......... .......... ..........
T996                          . .......... .......... .......... .......... .......... .......... ..........
T9102                         . .......... .......... .......... .......... .......... .......... ..........
K1                            . .......... .......... .......... .......... .......... .......... ..........
Dd2                           . .......... .......... .......... .......... .......... .......... ..........
D10                           . .......... .......... .......... .......... .......... .......... ..........
FCC2                          . .......... .......... .......... .......... .......... .......... ..........
HB3                           . .......... .......... .......... .......... .......... .......... ..........
7G8                           . .......... .......... .......... .......... .......... .......... ..........
P.reichenowi         .......... .......... .......... .......... .......... A......T.. .......... ..........

                             90        100        110        120        130        140        150        160
                     ....|....| ....|....| ....|....| ....|....| ....|....| ....|....| ....|....| ....|....|
MAL13P1.174          TAAGCGAACA AGTTATAATA AGAAAAAAGA AAACTTAAAA AATTTCGATA ATATTGTTCT TGATGATTTT TATTCAGCGT
3D7                  .......... .......... .......... .......... .......... .......... .......... ..........
RO33                 .......... .......... .......... .......... .......... .......... .......... ..........
PALO ALTO            .......... .......... .......... .......... .......... .......... .......... ..........
FCR3                 .......... .......... .......... .......... .......... .......... .......... ..........
WELLCOME             .......... .......... .......... .......... .......... .......... .......... ..........
D6                   .......... .......... .......... .......... .......... .......... .......... ..........
T996                 .......... .......... .......... .......... .......... .......... .......... ..........
T9102                .......... .......... .......... .......... .......... .......... .......... ..........
K1                   .......... .......... .......... .......... .......... .......... .......... ..........
Dd2                  .......... .......... .......... .......... .......... .......... .......... ..........
D10                  .......... .......... .......... .......... .......... .......... .......... ..........
FCC2                 .......... .......... .......... .......... .......... .......... .......... ..........
HB3                  .......... .......... .......... .......... .......... .......... .......... ..........
7G8                  .......... .......... .......... .......... .......... .......... .......... ..........
P.reichenowi         .......... .......... .......... .G..C..... .......... .......... .......... ........A.

                            170        180        190        200        210        220        230        240
                     ....|....| ....|....| ....|....| ....|....| ....|....| ....|....| ....|....| ....|....|
MAL13P1.174          TTGATTCCCA AGATAATTAT GAAAGTAAAT TAAAAAAAAA TGAGGATGAT ATTATCGGAC AAGGTATCTT TTCTCTTATA
3D7                  .......... .......... .......... .......... .......... .......... .......... ..........
RO33                 .......... .......... .......... .......... .......... .......... .......... ..........
PALO ALTO            .......... .......... .......... .......... .......... .......... .......... ..........
FCR3                 .......... .......... .......... .......... .......... .......... .......... ..........
WELLCOME             .......... .......... .......... .......... .......... .......... .......... ..........
D6                   .......... .......... .......... .......... .......... .......... .......... ..........
T996                 .......... .......... .......... .......... .......... .......... .......... ..........
T9102                .......... .......... .......... .......... .......... .......... .......... ..........
K1                   .......... .......... .......... .......... .......... .......... .......... ..........
Dd2                  .......... .......... .......... .......... .......... .......... .......... ..........
D10                  .......... .......... .......... .......... .......... .......... .......... ..........
FCC2                 .......... .......... .......... .......... .......... .......... .......... ..........
HB3                  .......... .......... .......... .......... .......... .......... .......... ..........
7G8                  .......... .......... .......... .......... .......... .......... .......... ..........
P.reichenowi         .......... .......... .......... .......... .......... .....T.... .......... ..........
```

```
                         250        260        270        280        290        300        310        320
                ....|....| ....|....| ....|....| ....|....| ....|....| ....|....| ....|....| ....|....|
MAL13P1.174     AGTAAAAAGA ATCAAGAAAA GGAAAAGTCA TTAAAAGGGG AATCAGAAGA TAATACAAAA CTTCAAGTAA CCAAAGTCCA
3D7             .......... .......... .......... .......... .......... .......... .......... ..........
RO33            .......... .......... .......... .......... .......... .......... .......... ..........
PALO ALTO       .......... .......... .......... .......... .......... .......... .......... ..........
FCR3            .......... .......... .......... .......... .......... .......... .......... ..........
WELLCOME        .......... .......... .......... .......... .......... .......... .......... ..........
D6              .......... .......... .......... .......... .......... .......... .......... ..........
T996            .......... .......... .......... .......... .......... .......... .......... ..........
T9102           .......... .......... .......... .......... .......... .......... .......... ..........
K1              .......... .......... .......... .......... .......... .......... .......... ..........
Dd2             .......... .......... .......... .......... .......... .......... .......... ..........
D10             .......... .......... .......... .......... .......... .......... .......... ..........
FCC2            .......... .......... .......... .......... .......... .......... .......... ..........
HB3             .......... .......... .......... .......... .......... .......... .......... ..........
7G8             .......... .......... .......... .......... .......... .......... .......... ..........
P.reichenowi    .......... .......... .......... .......... .......... .---.....T ...A...... ..G.......

                         330        340        350        360        370        380        390        400
                ....|....| ....|....| ....|....| ....|....| ....|....| ....|....| ....|....| ....|....|
MAL13P1.174     AGGTGCTCAA GTTGATCAAG CGGTTGAACC ATTGGAAAAA TCACCAGAAG AT------GA AAATAAAGAA ATACCAACTT
3D7             .......... .......... .......... .......... .......... ..------.. .......... ..........
RO33            .......... .......... .......... .......... .......... ..------.. .......... ..........
PALO ALTO       .......... .......... .......... .......... .......... ..------.. .......... ..........
FCR3            .......... .......... .......... .......... .......... ..------.. .......... ..........
WELLCOME        .......... .......... .......... .......... .......... ..------.. .......... ..........
D6              .......... .......... .......... .......... .......... ..------.. .......... ..........
T996            .......... .......... .......... .......... .......... ..------.. .......... ..........
T9102           .......... .......... .......... .......... .......... ..------.. .......... ..........
K1              .......... .......... .......... .......... .......... ..------.. .......... ..........
Dd2             .......... .......... .......... .......... .......... ..------.. .......... ..........
D10             .......... .......... .......... .......... .......... ..------.. .......... ..........
FCC2            .......... .......... .......... .......... .......... ..------.. .......... ..........
HB3             .......... .......... .......... .......... .......... ..------.. .......... ..........
7G8             .......... .......... .......... .......... .......... ..------.. .......... ..........
P.reichenowi    .......... .......... ...C....T. .......... .......... ..AAACATA. .G........ ..........

                         410        420        430        440        450        460        470        480
                ....|....| ....|....| ....|....| ....|....| ....|....| ....|....| ....|....| ....|....|
MAL13P1.174     TGGATTCACC ACAAAATGGA AATCCTACTT CTTATACATC AAATTTAAGT ACACCACCTC TTAAACGCAT GGATGAAGTT
3D7             .......... .......... .......... .......... .......... .......... .......... ..........
RO33            .......... .......... .......... .......... .......... .......... .......... ..........
PALO ALTO       .......... .......... .......... .......... .......... .......... .......... ..........
FCR3            .......... .......... .......... .......... .......... .......... .......... ..........
WELLCOME        .......... .......... .......... .......... .......... .......... .......... ..........
D6              .......... .......... .......... .......... .......... .......... .......... ..........
T996            .......... .......... .......... .......... .......... .......... .......... ..........
T9102           .......... .......... .......... .......... .......... .......... .......... ..........
K1              .......... .......... .......... .......... .......... .......... .......... ..........
Dd2             .......... .......... .......... .......... .......... .......... .......... ..........
D10             .......... .......... .......... .......... .......... .......... .......... ..........
FCC2            .......... .......... .......... .......... .......... .......... .......... ..........
HB3             .......... .......... .......... .......... .......... .......... .......... ..........
7G8             .......... .......... .......... .......... .......... .......... .......... ..........
P.reichenowi    .A.C...... .......A.. .........A .......... .......... .......... .......... ..........

                       490        500        510        520        530        540        550        560
                ....|....| ....|....| ....|....| ....|....| ....|....| ....|....| ....|....| ....|....|
MAL13P1.174     TTTGACGATG TTCTTAAACA TTTAAATAAA GAAGATAAAG TTGTCACTGA TGAGAATAAA AATAAATATA ATGAATTTAA
3D7             .......... .......... .......... .......... .......... .......... .......... ..........
RO33            .......... .......... .......... .......... .......... .......... .......... ..........
PALO ALTO       .......... .......... .......... .......... .......... .......... .......... ..........
FCR3            .......... .......... .......... .......... .......... .......... .......... ..........
WELLCOME        .......... .......... .......... .......... .......... .......... .......... ..........
D6              .......... .......... .......... .......... .......... .......... .......... ..........
T996            .....T.... .......... .......... .......... .......... .......... .......... ..........
T9102           .......... .......... .......... .......... .......... .......... .......... ..........
K1              .......... .......... .......... .......... .......... .......... .......... ..........
Dd2             .......... .......... .......... .......... .......... .......... .......... ..........
D10             .......... .......... .......... .......... .......... .......... .......... ..........
FCC2            .......... .......... .......... .......... .......... .......... .......... ..........
HB3             .......... .......... .......... .......... .......... .......... .......... ..........
7G8             .......... .......... .......... .......... .......... .......... .......... ..........
P.reichenowi    .....T.... .......... .......... .......... .......... ..G....... .......... ..........
```

```
                      570        580        590        600        610        620        630        640
                ....|....| ....|....| ....|....| ....|....| ....|....| ....|....| ....|....| ....|....|
MAL13P1.174     AAAAGAGTTT GATATCTTTA CTATGAATGT AAGTGAATAT GAAATAATGA AAAACCTTCT TATTACATTC TCAAAGAAAA
3D7             .......... .......... .......... .......... .......... .......... .......... ..........
RO33            .......... .......... .......... .......... .......... .......... .......... ..........
PALO ALTO       .......... .......... .......... .......... .......... .......... .......... ..........
FCR3            .......... .......... .......... .......... .......... .......... .......... ..........
WELLCOME        .......... .......... .......... .......... .......... .......... .......... ..........
D6              .......... .......... .......... .......... .......... .......... .......... ..........
T996            .......... .......... .......... .......... .......... .......... .......... ..........
T9102           .......... .......... .......... .......... .......... .......... .......... ..........
K1              .......... .......... .......... .......... .......... .......... .......... ..........
Dd2             .......... .......... .......... .......... .......... .......... .......... ..........
D10             .......... .......... .......... .......... .......... .......... .......... ..........
FCC2            .......... .......... .......... .......... .......... .......... .......... ..........
HB3             .......... .......... .......... .......... .......... .......... .......... ..........
7G8             .......... .......... .......... .......... .......... .......... .......... ..........
P.reichenowi    .......... .......... .......... .......... .......... .......... .......... ..........

                      650        660        670        680        690        700        710        720
                ....|....| ....|....| ....|....| ....|....| ....|....| ....|....| ....|....| ....|....|
MAL13P1.174     TTGATGAAAA TAATCAAATT CAAACAAAAA TAGAAAATAT ATTTAATAAA GCTTTAAAGG ATAATAAATA TAAAGAACAA
3D7             .......... .......... .......... .......... .......... .......... .......... ..........
RO33            .......... .......... .......... .......... .......... .......... .......... ..........
PALO ALTO       .......... .......... .......... .......... .......... .......... .......... ..........
FCR3            .......... .......... .......... .......... .......... .T........ .......... ..........
WELLCOME        .......... .......... .......... .......... .......... .T........ .......... ..........
D6              .......... .......... .......... .......... .......... .......... .......... ..........
T996            .......... .......... .......... .......... .......... .......... .......... ..........
T9102           .......... .......... .......... .......... .......... .......... .......... ..........
K1              .......... .......... .......... .......... .......... .......... .......... ..........
Dd2             .......... .......... .......... .......... .......... .......... .......... ..........
D10             .......... .......... .......... .......... .......... .......... .......... ..........
FCC2            .......... .......... .......... .......... .......... .......... .......... ..........
HB3             .......... .......... .......... .......... .......... .......... .......... ..........
7G8             .......... .......... .......... .......... .......... .......... .......... ..........
P.reichenowi    .......... .......... .......... .......... .......... .......C.. .......... ..........

                      730        740        750        760        770        780        790        800
                ....|....| ....|....| ....|....| ....|....| ....|....| ....|....| ....|....| ....|....|
MAL13P1.174     TTTAAAAACT TTATCTATGG ATTGTATAGC TTTGCAAAAA GACATAATTA TTTAATAGTT AATAAAACTA ATGACACAAC
3D7             .......... .......... .......... .......... .......... .......... .......... ..........
RO33            .......... .......... .......... .......... .......... .......... .......... ..........
PALO ALTO       .......... .......... .......... .......... .......... .......... .......... ..........
FCR3            .......... .......... .......... .......... .......... .......... .......... ..........
WELLCOME        .......... .......... .......... .......... .......... .......... .......... ..........
D6              .......... .......... .......... .......... .......... .......... .......... ..........
T996            .......... .......... .......... .......... .......... .......... .......... ..........
T9102           .......... .......... .......... .......... .......... .......... .......... ..........
K1              .......... .......... .......... .......... .......... .......... .......... ..........
Dd2             .......... .......... .......... .......... .......... .......... .......... ..........
D10             .......... .......... .......... .......... .......... .......... .......... ..........
FCC2            .......... .......... .......... .......... .......... .......... .......... ..........
HB3             .......... .......... .......... .......... .......... .......... .......... ..........
7G8             .......... .......... .......... .......... .......... .......... .......... ..........
P.reichenowi    .......... .......C.. ...A...... ...A...... .......... .C...GG... .........T ..........

                      810        820        830        840        850
                ....|....| ....|....| ....|....| ....|....| ....|....| ..
MAL13P1.174     ACTACATAAA GATTTATTTG AAAATGCTCT TAATTTAATA AATACTATTT AG
3D7             .......... .......... .......... ....
RO33            .......... .......... .......... ....
PALO ALTO       .......... .......... .......... ....
FCR3            .......... .......... .......... ....
WELLCOME        .......... .......... .......... ....
D6              .......... .......... .......... ....
T996            .......... .......... .......... ....
T9102           .......... .......... .......... ....
K1              .......... .......... .......... ....
Dd2             .......... .......... .......... ....
D10             .......... .......... .......... ....
FCC2            .......... .......... .......... ....
HB3             .......... .......... .......... ....
7G8             .......... .......... .......... ....
P.reichenowi    ...T.....G ...A...... .......... .......... .......... ..
```

MRSP3

```
                                 10         20         30         40         50         60         70         80
                        ....|....| ....|....| ....|....| ....|....| ....|....| ....|....| ....|....| ....|....|
PF13_0193               ATGATAAAAG GACTTATTTT TTATTTGTTT ATATGCTTTT TTGTATTTTT CGTACACGCT GTTTCATCAC AGGAACAGCC
3D7
RO33
Palo Alto
FCR3
Wellcome
D6
T996
T9102
K1
Dd2
D10
FCC2
HB3
7G8
P.reichenowi            .......... ...CC..... .......... ....C..... .......... .......A.. ...C...... ..........

                                 90        100        110        120        130        140        150        160
                        ....|....| ....|....| ....|....| ....|....| ....|....| ....|....| ....|....| ....|....|
PF13_0193               AAACCATACA GACATATATA CCAATGAAGA TTATAAACTA TTAGAAGAAC TAGAAAAATA TCTTGATAAT TTAAAAAATA
3D7                                                       ......... .......... .......... .......... ..........
RO33                                                      ......... .......... .......... .......... ..........
Palo Alto                                                 ......... .......... .......... .......... ..........
FCR3                                                      ......... .......... .......... .......... ..........
Wellcome                                                  ......... .......... .......... .......... ..........
D6                                                        ......... .......... .......... .......... ..........
T996                                                      ......... .......... .......... .......... ..........
T9102                                                     ......... .......... .......... .......... ..........
K1                                                        ......... .......... .......... .......... ..........
Dd2                                                       ......... .......... .......... .......... ..........
D10                                                       ......... .......... .......... .......... ..........
FCC2                                                      ......... .......... .......... .......... ..........
HB3                                                       ......... .......... .......... .......... ..........
7G8                                                       ......... .......... .......... .......... ..........
P.reichenowi            .......... ..T....... A.T..A.... .......... .......... .....G.... .......... ...G......

                                170        180        190        200        210        220        230        240
                        ....|....| ....|....| ....|....| ....|....| ....|....| ....|....| ....|....| ....|....|
PF13_0193               CATTTACACA CAAGTTTCTT GAGAACTTTA AAGGTGATAT CGAATTATTA AAAAAAAACA TTCAAAACTT TGAAAAAAAT
3D7                     .......... .......... .......... .......... .......... .......... .......... ..........
RO33                    .......... .......... .......... .......... .......... .......... .......... ..........
Palo Alto               .......... .......... .......... .......... .......... .......... .......... ..........
FCR3                    .......... .......... .......... .......... .......... .......... .......... ..........
Wellcome                .......... .......... .......... .......... .......... .......... .......... ..........
D6                      .......... .......... .......... .......... .......... .......... .......... ..........
T996                    .......... .......... .......... .......... .......... .......... .......... ..........
T9102                   .......... .......... .......... .......... .......... .......... .......... ..........
K1                      .......... .......... .......... .......... .......... .......... .......... ..........
Dd2                     .......... .......... .......... .......... .......... .......... .......... ..........
D10                     .......... .......... .......... .......... .......... .......... .......... ..........
FCC2                    .......... .......... .......... .......... .......... .......... .......... ..........
HB3                     .......... .......... .......... .......... .......... .......... .......... ..........
7G8                     .......... .......... .......... .......... .......... .......... .......... ..........
P.reichenowi            .......... .......... ..A....... .......... .....A.... ........T. .......... ..........

                                250        260        270        280        290        300        310        320
                        ....|....| ....|....| ....|....| ....|....| ....|....| ....|....| ....|....| ....|....|
PF13_0193               AATTATAATA ATGTAACAGG TCAAAATGTG GACGATTATG AT-------- ---------- ------AAAA ATGGTGATAA
3D7                     .......... .......... .......... .......... ..-------- ---------- ------.... ..........
RO33                    .......... .......... .......... .......... ..-------- ---------- ------.... ..........
Palo Alto               .......... .......... .......... .......... ..-------- ---------- ------.... ..........
FCR3                    .......... .......... .......... .......... ..-------- ---------- ------.... ..........
Wellcome                .......... .......... .......... .......... ..-------- ---------- ------.... ..........
D6                      .......... .......... .......... .......... ..-------- ---------- ------.... ..........
T996                    .......... .......... .......... .......... ..-------- ---------- ------.... ..........
T9102                   .......... .......... .......... .......... ..-------- ---------- ------.... ..........
K1                      .......... .......... .......... .......... ..-------- ---------- ------.... ..........
Dd2                     .......... .......... .......... .......... ..-------- ---------- ------.... ..........
D10                     .......... .......... .......... .......... ..-------- ---------- ------.... ..........
FCC2                    .......... .......... .......... .......... ..-------- ---------- ------.... ..........
HB3                     .......... .......... .......... .......... ..-------- ---------- ------.... ..........
7G8                     .......... .......... .......... .......... ..-------- ---------- ------.... ..........
P.reichenowi            .......... .......... ....G..... ...A...... ..GATGAACA TCAAAACAGT TATGAT.... .....C....
```

```
                           330        340        350        360        370        380        390        400
                    ....|....| ....|....| ....|....| ....|....| ....|....| ....|....| ....|....| ....|....|
PF13_0193           TAATAATCAT AATGAT---- ---------- ---------- ---------- ---------- ---------- ----------
3D7                 .......... ......---- ---------- ---------- ---------- ---------- ---------- ----------
RO33                .......... ......---- ---------- ---------- ---------- ---------- ---------- ----------
Palo Alto           .......... ......---- ---------- ---------- ---------- ---------- ---------- ----------
FCR3                .......... ......---- ---------- ---------- ---------- ---------- ---------- ----------
Wellcome            .......... ......---- ---------- ---------- ---------- ---------- ---------- ----------
D6                  .......... ......---- ---------- ---------- ---------- ---------- ---------- ----------
T996                .......... ......---- ---------- ---------- ---------- ---------- ---------- ----------
T9102               .......... ......---- ---------- ---------- ---------- ---------- ---------- ----------
K1                  .......... ......---- ---------- ---------- ---------- ---------- ---------- ----------
Dd2                 .......... ......---- ---------- ---------- ---------- ---------- ---------- ----------
D10                 .......... ......---- ---------- ---------- ---------- ---------- ---------- ----------
FCC2                .......... ......---- ---------- ---------- ---------- ---------- ---------- ----------
HB3                 .......... ......---- ---------- ---------- ---------- ---------- ---------- ----------
7G8                 .......... ......---- ---------- ---------- ---------- ---------- ---------- ----------
P.reichenowi        A...G..G.. ..AA..GATG ATAAAAATGG TCATAAAAAT GATGATAAAA ATGGTCATAA AAATGATGAT AAAAATGATG

                           410        420        430        440        450        460        470        480
                    ....|....| ....|....| ....|....| ....|....| ....|....| ....|....| ....|....| ....|....|
PF13_0193           ---------- ---------- ---------- ---------- ---------- ---------- ---------- ----------
3D7                 ---------- ---------- ---------- ---------- ---------- ---------- ---------- ----------
RO33                ---------- ---------- ---------- ---------- ---------- ---------- ---------- ----------
Palo Alto           ---------- ---------- ---------- ---------- ---------- ---------- ---------- ----------
FCR3                ---------- ---------- ---------- ---------- ---------- ---------- ---------- ----------
Wellcome            ---------- ---------- ---------- ---------- ---------- ---------- ---------- ----------
D6                  ---------- ---------- ---------- ---------- ---------- ---------- ---------- ----------
T996                ---------- ---------- ---------- ---------- ---------- ---------- ---------- ----------
T9102               ---------- ---------- ---------- ---------- ---------- ---------- ---------- ----------
K1                  ---------- ---------- ---------- ---------- ---------- ---------- ---------- ----------
Dd2                 ---------- ---------- ---------- ---------- ---------- ---------- ---------- ----------
D10                 ---------- ---------- ---------- ---------- ---------- ---------- ---------- ----------
FCC2                ---------- ---------- ---------- ---------- ---------- ---------- ---------- ----------
HB3                 ---------- ---------- ---------- ---------- ---------- ---------- ---------- ----------
7G8                 ---------- ---------- ---------- ---------- ---------- ---------- ---------- ----------
P.reichenowi        ATAAAAATGA TGATAAAAAT GGTGATAAAA ATGATGATAA AAATGATGAT AAAAATGGTG ATAAAAATGA TGATAAAAAT

                           490        500        510        520        530        540        550        560
                    ....|....| ....|....| ....|....| ....|....| ....|....| ....|....| ....|....| ....|....|
PF13_0193           ---------- --CTTGAACA AAATCAATTA TTACAAAGTA ATATTAAATC ATTTTTTGGA CAAAGTAGAA CAACCAATAC
3D7                 ---------- --........ .......... .......... .......... .......... .......... ..........
RO33                ---------- --........ .......... .......... .......... .......... .......... ........G.
Palo Alto           ---------- --........ .......... .......... .......... .......... .......... ..........
FCR3                ---------- --........ .......... .......... .......... .......... .......... ..........
Wellcome            ---------- --........ .......... .......... .......... .......... .......... ..........
D6                  ---------- --........ .......... .......... .......... .......... .......... ..........
T996                ---------- --........ .......... .......... .......... .......... .......... ..........
T9102               ---------- --........ .......... .......... .......... .......... .......... ..........
K1                  ---------- --........ .......... .......... .......... .......... .......... ..........
Dd2                 ---------- --........ .......... .......... .......... .......... .......... ..........
D10                 ---------- --........ .......... .......... .......... .......... .......... ..........
FCC2                ---------- --........ .......... .......... .......... .......... .......... ..........
HB3                 ---------- --........ .......... .......... .......... .......... .......... ..........
7G8                 ---------- --........ .......... .......... .......... .......... .......... ..........
P.reichenowi        GATGATAAAA AT-------- ---------- ---------- ---------- ---------- ---------- ----------

                           570        580        590        600        610        620        630        640
                    ....|....| ....|....| ....|....| ....|....| ....|....| ....|....| ....|....| ....|....|
PF13_0193           AGTTGATGTT TCAGCAAGTA AAGTTACTCA AAAAAGTGAT GAAACCAAAG CTGATGAAAA AGCTGAACAG AATGCTGAAG
3D7                 .......... .......... .......... .......... .......... .......... .......... ..........
RO33                .......... .......... .......... .......... .......... .......... .......... ..........
Palo Alto           .......... .......... .......... .......... .......... .......... .......... ..........
FCR3                .......... .......... .......... .......... .......... .......... .......... ..........
Wellcome            .......... .......... .......... .......... .......... .......... .......... ..........
D6                  .......... .......... .......... .......... .......... .......... .......... ..........
T996                .......... .......... .......... .......... .......... .......... .......... ..........
T9102               .......... .......... .......... .......... .......... .......... .......... ..........
K1                  .......... .......... .......... .......... .......... .......... .......... ..........
Dd2                 .......... .......... .......... .......... .......... .......... .......... ..........
D10                 .......... .......... .......... .......... .......... .......... .......... ..........
FCC2                .......... .......... .......... .......... .......... .......... .......... ..........
HB3                 .......... .......... .......... .......... .......... .......... .......... ..........
7G8                 .......... .......... .......... .......... .......... .......... .......... ..........
P.reichenowi        ---------- ---------- ---------- ---------- ---------- --........ ..A..C...A .........C
```

```
                        650        660        670        680        690        700        710        720
                ....|....| ....|....| ....|....| ....|....| ....|....| ....|....| ....|....| ....|....|
PF13_0193       CTAACGCATC TTCATCCGCT TCATCAGAAG AACAAAGTTC ATCATTACAA AATACAAATG ACCAATCATT TGGTAACACA
3D7             .......... .......... .......... .......... .......... .......... .......... ..........
RO33            .......... .......... .......... .......... .......... .......... .......... ..........
Palo Alto       .......... .......... .......... .......... .......... .......... .......... ..........
FCR3            .......... .......... .......... .......... .......... .......... .......... ..........
Wellcome        .......... .......... .......... .......... .......... .......... .......... ..........
D6              .......... .......... .......... .......... .......... .......... .......... ..........
T996            .......... .......... .......... .......... .......... .......... .......... ..........
T9102           .......... .......... .......... .......... .......... .......... .......... ..........
K1              .......... .......... .......... .......... .......... .......... .......... ..........
Dd2             .......... .......... .......... .......... .......... .......... .......... ..........
D10             .......... .......... .......... .......... .......... .......... .......... ..........
FCC2            .......... .......... .......... .......... .......... .......... .......... ..........
HB3             .......... .......... .......... .......... .......... .......... .......... ..........
7G8             .......... .......... .......... .......... .......... .......... .......... ..........
P.reichenowi    .......... ......TC.. ...A....C. .......... ..G.G..... .......G.. .T........ ..A.......

                        730        740        750        760        770        780        790        800
                ....|....| ....|....| ....|....| ....|....| ....|....| ....|....| ....|....| ....|....|
PF13_0193       AATAATCCAA AAATGGATAG AAAAAATGCC GATGAACAAA AAAGAGATGC AGGTTTATCT AATTTAAAAA ATATAGATAA
3D7             .......... .......... .......... .......... .......... .......... .......... ..........
RO33            .......... .......... .......... .......... .......... .......... .......... ..........
Palo Alto       .......... .......... .......... .......... .......... .......... .......... ..........
FCR3            .......... .......... .......... .......... .......... .......... .......... ..........
Wellcome        .......... .......... .......... .......... .......... .......... .......... ..........
D6              .......... .......... .......... .......... .......... .......... .......... ..........
T996            .......... .......... .......... .......... .......... .......... .......... ..........
T9102           .......... .......... .......... .......... .......... .......... .......... ..........
K1              .......... .......... .......... .......... .......... .......... .......... ..........
Dd2             .......... .......... .......... .......... .......... .......... .......... ..........
D10             .......... .......... .......... .......... .......... .......... .......... ..........
FCC2            .......... .......... .......... .......... .......... .......... .......... ..........
HB3             .......... .......... .......... .......... .......... .......... .......... ..........
7G8             .......... .......... .......... .......... .......... .......... .......... ..........
P.reichenowi    .......... ....A....C .T...TAA.. .......... .......... ..TA..T.A. CTAA.TT... .A...T.G.T

                        810        820        830        840        850        860        870        880
                ....|....| ....|....| ....|....| ....|....| ....|....| ....|....| ....|....| ....|....|
PF13_0193       ACTTTGCGAT GAACTCCTAA TCTATTTATA TAAAAAAAAT GAAGTAAATA CCAATAATTA TCTTAGAAAA TATGATGAAT
3D7             .......... .......... .......... .......... .......... .......... .......... ..........
RO33            .......... .......... .......... .......... .......... .......... .......... ..........
Palo Alto       .......... .......... .......... .......... .......... .......... .......... ..........
FCR3            .......... .......... .......... .......... .......... .......... .......... ..........
Wellcome        .......... .......... .......... .......... .......... .......... .......... ..........
D6              .......... .......... .......... .......... .......... .......... .......... ..........
T996            .......... .......... .......... .......... .......... .......... .......... ..........
T9102           .......... .......... .......... .......... .......... .......... .......... ..........
K1              .......... .......... .......... .......... .......... .......... .......... ..........
Dd2             .......... .......... .......... .......... .......... .......... .......... ..........
D10             .......... .......... .......... .......... .......... .......... .......... ..........
FCC2            .......... .......... .......... .......... .......... .......... .......... ..........
HB3             .......... .......... .......... .......... .......... .......... .......... ..........
7G8             .......... .......... .......... .......... .......... .......... .......... ..........
P.reichenowi    .AAC.TT.CG ATGAA.TCCT AA.C.A.T.. ..T......A A.T.A.GTA. AT.CC...A. .TA.CTT.G. A.AT....TG

                        890        900        910        920        930        940        950        960
                ....|....| ....|....| ....|....| ....|....| ....|....| ....|....| ....|....| ....|....|
PF13_0193       TTAAAAATAA ATTTGATCAA TTTTTATTGA ATGTTAATAA ATATGAATTA CTTAAGAAAC TCATTCTTAA TTTTTTTAAA
3D7             .......... .......... .......... .......... .......... .......... .......... ..........
RO33            .......... .......... .......... .......... .......... .......... .......... ..........
Palo Alto       .......... .......... .......... .......... .......... .......... .......... ..........
FCR3            .......... .......... .......... .......... .......... .......... .......... ..........
Wellcome        .......... .......... .......... .......... .......... .......... .......... ..........
D6              .......... .......... .......... .......... .......... .......... .......... ..........
T996            .......... .......... .......... .......... .......... .......... .......... ..........
T9102           .......... .......... .......... .......... .......... .......... .......... ..........
K1              .......... .......... .......... .......... .......... .......... .......... ..........
Dd2             .......... .......... .......... .......... .......... .......... .......... ..........
D10             .......... .......... .......... .......... .......... .......... .......... ..........
FCC2            .......... .......... .......... .......... .......... .......... .......... ..........
HB3             .......... .......... .......... .......... .......... .......... .......... ..........
7G8             .......... .......... .......... .......... .......... .......... .......... ..........
P.reichenowi    AATTT..A.. TAAATT.G.T CAA..T..AT TGAA.GT... TA.AT.TGA. T.ACTT..GA AAC.CA..CT .AA.G..TTT
```

```
                       970        980        990       1000       1010       1020       1030       1040
                ....|....| ....|....| ....|....| ....|....| ....|....| ....|....| ....|....| ....|....|
PF13_0193       GATAATCTAA AAGATCATAG AGCACAATAC AAATTCTACA TTGCATTAAT GAAGGCTTTA GAAAATGAAA AATTTTCTCA
3D7             .......... .......... .......... .......... .......... .......... .......... ..........
RO33            .......... .......... .......... .......... .......... .......... .......... ..........
Palo Alto       .......... .......... .......... .......... .......... .......... .......... ..........
FCR3            .......... .......... .......... .......... .......... .......... .......... ..........
Wellcome        .......... .......... .......... .......... .......... .......... .......... ..........
D6              .......... .......... .......... .......... .......... .......... .......... ..........
T996            .......... .......... .......... .......... .......... .......... .......... ..........
T9102           .......... .......... .......... .......... .......... .......... .......... ..........
K1              .......... .......... .......... .......... .......... .......... .......... ..........
Dd2             .......... .......... .......... .......... .......... .......... .......... ..........
D10             .......... .......... .......... .......... .......... .......... .......... ..........
FCC2            .......... .......... .......... .......... .......... .......... .......... ..........
HB3             .......... .......... .......... .......... .......... .......... .......... ..........
7G8             .......... .......... .......... .......... .......... .......... .......... ..........
P.reichenowi    A.AG.GAATC T.A.AG..CA TA..G..C.A T.CAAA.T.T ACATTGC.T. A.T.AAGGAT TT.G.AA.TG ..AAA.T..C

                      1050       1060       1070       1080       1090       1100       1110       1120
                ....|....| ....|....| ....|....| ....|....| ....|....| ....|....| ....|....| ....|....|
PF13_0193       AGAATTTAAA AAGGTAATGT ATGGTTTATA TAACACTTTG ATGATAAAAC ATTATGAAAG TATAACAAAT GAACAAGTAC
3D7             .......... .......... .......... .......... .......... .......... .......... ..........
RO33            .......... .......... .......... .......... .......... .......... .......... ..........
Palo Alto       .......... .......... .......... .......... .......... .......... .......... ..........
FCR3            .......... .......... .......... .......... .......... .......... .......... ..........
Wellcome        .......... .......... .......... .......... .......... .......... .......... ..........
D6              .......... .......... .......... .......... .......... .......... .......... ..........
T996            .......... .......... .......... .......... .......... .......... .......... ..........
T9102           .......... .......... .......... .......... .......... .......... .......... ..........
K1              .......... .......... .......... .......... .......... .......... .......... ..........
Dd2             .......... .......... .......... .......... .......... .......... .......... ..........
D10             .......... .......... .......... .......... .......... .......... .......... ..........
FCC2            .......... .......... .......... .......... .......... .......... .......... ..........
HB3             .......... .......... .......... .......... .......... .......... .......... ..........
7G8             .......... .......... .......... .......... .......... .......... .......... ..........
P.reichenowi    TC..GAATTT ..AAAT..AA TGTA.GGT.T AT.T.ACACT T....G.T.A .AC..T.TGA A.GT.T..CA A.TG..CA.G

                      1130       1140       1150       1160       1170       1180       1190       1200
                ....|....| ....|....| ....|....| ....|....| ....|....| ....|....| ....|....| ....|....|
PF13_0193       CACAAGAACC AGGATCAACA TCATCAGAAA CAAATGAAGA TAAACAAGAA GATAAACAAG AAGATAAA-- ----------
3D7             .......... .......... .......... .......... .......... .......... ........-- ----------
RO33            .......... .......... .......... .......... .......... ......---- ---------- ----------
Palo Alto       .......... .......... .......... .......... .......... .......... ........-- ----------
FCR3            .......... .......... .......... .......... .......... .......... ........-- ----------
Wellcome        .......... .......... .......... .......... .......... .......... ........-- ----------
D6              .......... .......... .......... .......... .......... .......... ........CG AGAAGATAAA
T996            .......... .......... .......... .......... .......... .......... ........-- ----------
T9102           .......... .......... .......... .......... .......... .......... ........-- ----------
K1              .......... .......... .......... .......... .......... .......... ........-- ----------
Dd2             .......... .......... .......... .......... .......... .......... ........-- ----------
D10             .......... .......... .......... .......... .......... .......... ........-- ----------
FCC2            .......... .......... .......... .......... .......... .......... ........-- ----------
HB3             .......... .......... .......... .......... .......... .......... ........-- ----------
7G8             .......... .......... .......... .......... .......... .......... ........-- ----------
P.reichenowi

                      1210       1220       1230       1240       1250       1260       1270       1280
                ....|....| ....|....| ....|....| ....|....| ....|....| ....|....| ....|....| ....|....|
PF13_0193       ---------- ---------- ----CGAGAA GATAAACAAG AAGAAGGAGA AACAGCATCG GAACAAGAAA CCATACCATC
3D7             ---------- ---------- ----...... .......... .......... .......... .........
RO33            ---------- ---------- ----...... .......... .......... .......... .........
Palo Alto       ---------- ---------- ----...... .......... .......... .......... .........
FCR3            ---------- ---------- ----...... .......... .......... .......... .........
Wellcome        ---------- ---------- ----...... .......... .......... .......... .........
D6              CAAGAAGATA AACAAGAAGA TAAA...... .......... .......... .......... .........
T996            ---------- ---------- ----...... .......... .......... .......... .........
T9102           ---------- ---------- ----...... .......... .......... .......... .........
K1              ---------- ---------- ----...... .......... .......... .......... .........
Dd2             ---------- ---------- ----...... .......... .......... .......... .........
D10             ---------- ---------- ----...... .......... .......... .......... .........
FCC2            ---------- ---------- ----...... .......... .......... .......... .........
HB3             ---------- ---------- ----...... .......... .......... .......... .........
7G8             ---------- ---------- ----...... .......... .......... .......... .........
P.reichenowi
```

```
                       1290       1300       1310
                   ....|....| ....|....| ....|....| .
PF13_0193          ACCAGCCACA GCGACACAAC AAAAAAAATA A
3D7
RO33
Palo Alto
FCR3
Wellcome
D6
T996
T9102
K1
Dd2
D10
FCC2
HB3
7G8
P.reichenowi
```

MRSP4

```
                           10         20         30         40         50         60         70         80
                   ....|....| ....|....| ....|....| ....|....| ....|....| ....|....| ....|....| ....|....|
MAL13_P1.173       ATGAAAGGTA GAATTATATC TTTTTCATTT TTCCTTTTCT GTATGGTTCA TTTTGTATTT TGTGATAAAA ATACATTCCC
3D7                      .... .......... .......... .......... .......... .......... .......... ..........
RO33                     .... .......... .......... .......... .......... .......... .......... ..........
Palo Alto                .... .......... .......... .......... .......... .......... .......... ..........
FCR3                     .... .......... .......... .......... .......... .......... .......... ..........
Wellcome                 .... .......... .......... .......... .......... .......... .......... ..........
D6                       .... .......... .......... .......... .......... .......... .......... ..........
T996                     .... .......... .......... .......... .......... .......... .......... ..........
T9102                    .... .......... .......... .......... .......... .......... .......... ..........
K1                       .... .......... .......... .......... .......... .......... .......... ..........
Dd2                      .... .......... .......... .......... .......... .......... .......... ..........
FCC2                     .... .......... .......... .......... .......... .......... .......... ..........
D10                      .... .......... .......... .......... .......... .......... .......... ..........
HB3                      .... .......... .......... .......... .......... .......... .......... ..........
7G8                      .... .......... .......... .......... .......... .......... .......... ..........
P.reichenowi             .... .......... ......C... .......... .......... .......... ......G... ..........

                           90        100        110        120        130        140        150        160
                   ....|....| ....|....| ....|....| ....|....| ....|....| ....|....| ....|....| ....|....|
MAL13_P1.173       TAAAGAAATA ATTGAATATG AAAAAGACTT GGAATTAGCA AAAATAAAAG AAAAATTACA AAACTTAAAT GACATAATAG
3D7                .......... .......... .......... .......... .......... .......... .......... ..........
RO33               .......... .......... .......... .......... .......... .......... .......... ..........
Palo Alto          .......... .......... .......... .......... .......... .......... .......... ..........
FCR3               .......... .......... .......... .......... .......... .......... .......... ..........
Wellcome           .......... .......... .......... .......... .......... .......... .......... ..........
D6                 .......... .......... .......... .......... .......... .......... .......... ..........
T996               .......... .......... .......... .......... .......... .......... .......... ..........
T9102              .......... .......... .......... .......... .......... .......... .......... ..........
K1                 .......... .......... .......... .......... .......... .......... .......... ..........
Dd2                .......... .......... .......... .......... .......... .......... .......... ..........
FCC2               .......... .......... .......... .......... .......... .......... .......... ..........
D10                .......... .......... .......... .......... .......... .......... .......... ..........
HB3                .......... .......... .......... .......... .......... .......... .......... ..........
7G8                .......... .......... .......... .......... .......... .......... .......... ..........
P.reichenowi       .......... .A........ .......... .......... .......... .......... .......... ..........

                          170        180        190        200        210        220        230        240
                   ....|....| ....|....| ....|....| ....|....| ....|....| ....|....| ....|....| ....|....|
MAL13_P1.173       TAGACAAATT GATAGAATCC TTTAAAGATA ATGTTGATGT ATTAAAAGTA ATAATTCAAG AATTAGAAAA AGAAAAAGAA
3D7                .......... .......... .......... .......... .......... .......... .......... ..........
RO33               .......... .......... .......... .......... .......... .......... .......... ..........
Palo Alto          .......... .......... .......... .......... .......... .......... .......... ..........
FCR3               .......... .......... .......... .......... .......... .......... .......... ..........
Wellcome           .......... .......... .......... .......... .......... .......... .......... ..........
D6                 .......... .......... .......... .......... .......... .......... .......... ..........
T996               .......... .......... .......... .......... .......... .......... .......... ..........
T9102              .......... .......... .......... .......... .......... .......... .......... ..........
K1                 .......... .......... .......... .......... .......... .......... .......... ..........
Dd2                .......... .......... .......... .......... .......... .......... .......... ..........
FCC2               .......... .......... .......... .......... .......... .......... .......... ..........
D10                .......... .......... .......... .......... .......... .......... .......... ..........
HB3                .......... .......... .......... .......... .......... .......... .......... ..........
7G8                .......... .......... .......... .......... .......... .......... .......... ..........
P.reichenowi       .......... .........G .......... .......... ......T... .......... .......... ..........
```

```
                     250        260        270        280        290        300        310        320
              ....|....| ....|....| ....|....| ....|....| ....|....| ....|....| ....|....| ....|....|
MAL13_P1.173  AAAGAAAATA AATTGGCAAA GAAAAAAAGG GAAATAAGCA ATAATGATTT TTTAAACAAT AAGAATAGTA ATAAAATTAA
3D7           .......... .......... .......... .......... .......... .......... .......... ..........
RO33          .......... .......... .......... .......... .......... .......... .......... .....T.G..
Palo Alto     .......... .......... .......... .......... .......... .......... .......... .....T....
FCR3          ------.... .......... .......... .......... .......... .......... .......... .....T....
Wellcome      ------.... .......... .......... .......... .......... .......... .......... .....T....
D6            .......... .......... .......... .......... .......... .......... .......... .....T....
T996          .......... .......... .......... .......... .......... .......... .......... .....T....
T9102         .......... .......... .......... .......... .......... .......... .......... .....T....
K1            .......... .......... .......... .......... .......... .......... .......... .....T....
Dd2           .......... .......... .......... .......... .......... .......... .......... ..........
FCC2          .......... .......... .......... .......... .......... .......... .......... .....T....
D10           .......... .......... .......... .......... .......... .......... .......... .....T....
HB3           .......... .......... .......... .......... .......... .......... .......... .....T....
7G8           .......... .......... .......... .......... .......... .......... .......... .....T....
P.reichenowi  ------.... .......... ....G..... .......... .......... .......... ..A....T.. .....T....

                     330        340        350        360        370        380        390        400
              ....|....| ....|....| ....|....| ....|....| ....|....| ....|....| ....|....| ....|....|
MAL13_P1.173  AGGACAAGGA TTTTTTACTT CTACATGGAA ACAAATAAAA GGAAATTCTG AAGAAAATTC GGAGAATCAA CAAAAATCAT
3D7           .......... .......... .......... .......... .......... .......... .......... ..........
RO33          ....A..... .......... .......... .......... .......... .......... .......... ..........
Palo Alto     ....A..... .......... .......... .......... .......... .......... .......... ..........
FCR3          ....A..... .......... .......... .......... .......... .......... .......... ..........
Wellcome      ....A..... .......... .......... .......... .......... .......... .......... ..........
D6            ....A..... .......... .......... .......... .......... .......... .......... ..........
T996          ....A..... .......... .......... .......... .......... .......... .......... ..........
T9102         ....A..... .......... .......... .......... .......... .......... .......... ..........
K1            ....A..... .......... .......... .......... .......... .......... .......... ..........
Dd2           .......... .......... .......... .......... .......... .......... .......... ..........
FCC2          ....A..... .......... .......... .......... .......... .......... .......... ..........
D10           .......... .......... .......... .......... .......... .......... .......... ..........
HB3           ....A..... .......... .......... .......... .......... .......... .......... ..........
7G8           ....A..... .......... .......... .......... .......... .......... .......... ..........
P.reichenowi  .........T .......... .......... ......T... .......... ....G..... .......... ..........

                     410        420        430        440        450        460        470        480
              ....|....| ....|....| ....|....| ....|....| ....|....| ....|....| ....|....| ....|....|
MAL13_P1.173  CAAAAAGCCA ATCGTTACAA TCAAATGAAC CAAATAAATT AAATCAAGAA AATATATCCC CAGTACAAAC CACCATAACA
3D7           .......... .......... .......... .......... .......... .......... .......... ..........
RO33          .......... .......... .......... .......... .......... .......... .......... ..........
Palo Alto     .......... .......... .......... .......... .......... .......... .......... ..........
FCR3          .......... .......... .......... .......... .......... .......... .......... ..........
Wellcome      .......... .......... .......... .......... .......... .......... .......... ..........
D6            .......... .......... .......... .......... .......... .......... .......... ..........
T996          .......... .......... .......... .......... .......... .......... .......... ..........
T9102         .......... .......... .......... .......... .......... .......... .......... ..........
K1            .......... .......... .......... .......... .......... .......... .......... ..........
Dd2           .......... .......... .......... .......... .......... .......... .......... ..........
FCC2          .......... .......... .......... .......... .......... .......... .......... ..........
D10           .......... .......... .......... .......... .......... .......... .......... ..........
HB3           .......... .......... .......... .......... .......... .......... .......... ..........
7G8           .......... .......... .......... .......... .......... .......... .......... ..........
P.reichenowi  .......T.. .......... .......... .......... .......... .......... .......... ..........

                     490        500        510        520        530        540        550        560
              ....|....| ....|....| ....|....| ....|....| ....|....| ....|....| ....|....| ....|....|
MAL13_P1.173  AATACAAATG TTGATAATAC AATATCTGTA ATTAAATATT TAGATAATGC TTATGATGAA GTTCTTAAGG AAATGAATTT
3D7           .......... .......... .......... .......... .......... .......... .......... ..........
RO33          .......... .......... .......... .......... .......... .......... .......... ..........
Palo Alto     .......... .......... .......... .......... .......... .......... .......... ..........
FCR3          .......... .......... .......... .......... .......... .......... .......... ..........
Wellcome      .......... .......... .......... .......... .......... .......... .......... ..........
D6            .......... .......... .......... .......... .......... .......... .......... ..........
T996          .......... .......... .......... .......... .......... .......... .......... ..........
T9102         .......... .......... .......... .......... .......... .......... .......... ..........
K1            .......... .......... .......... .......... .......... .......... .......... ..........
Dd2           .......... .......... .......... .......... .......... .......... .......... ..........
FCC2          .......... .......... .......... .......... .......... .......... .......... ..........
D10           .......... .......... .......... .......... .......... .......... .......... ..........
HB3           .......... .......... .......... .......... .......... .......... .......... ..........
7G8           .......... .......... .......... .......... .......... .......... .......... ..........
P.reichenowi  .......... .......... .......... .......... .......... .......... ........A. ..........
```

```
                      570        580        590        600        610        620        630        640
                 ....|....| ....|....| ....|....| ....|....| ....|....| ....|....| ....|....| ....|....|
MAL13_P1.173     ATCCAAAAAT TCAGATAATG AAAAATATCG TAGCAGGTTT GACAATTTCA AACAAGGATT TGAAAATTTA ATCCTTAATC
3D7              .......... .......... .......... .......... .......... .......... .......... ..........
RO33             .......... .......... ........G. .......... .......... .......... .......... ..........
Palo Alto        .......... .......... .......... .......... .......... .......... .......... ..........
FCR3             .......... .......... .......... .......... .......... .......... .......... ..........
Wellcome         .......... .......... .......... .......... .......... .......... .......... ..........
D6               .......... .......... .......... .......... .......... .......... .......... ..........
T996             .......... .......... .......... .......... .......... .......... .......... ..........
T9102            .......... .......... .......... .......... .......... .......... .......... ..........
K1               .......... .......... .......... .......... .......... .......... .......... ..........
Dd2              .......... .......... .......... .......... .......... .......... .......... ..........
FCC2             .......... .......... .......... .......... .......... .......... .......... ..........
D10              .......... .......... .......... .......... .......... .......... .......... ..........
HB3              .......... .......... .......... .......... .......... .......... .......... ..........
7G8              .......... .......... ........G. .......... .......... .......... .......... ..........
P.reichenowi     ...G...... .......... .......... .......... ....GC.... .......... .......... ..........

                      650        660        670        680        690        700        710        720
                 ....|....| ....|....| ....|....| ....|....| ....|....| ....|....| ....|....| ....|....|
MAL13_P1.173     AAAATGAATA TGAACTTATT AAGAGACTTA TTCTTGCTTT TTCTAATCAA GAAGAATCAG GTACTAATAA AAAAAATCAT
3D7              .......... .......... .......... .......... .......... .......... .......... ..........
RO33             .......... .......... .......... .......... .......... .......... .......... ..........
Palo Alto        .......... .......... .......... .......... .......... .......... .......... ..........
FCR3             .......... .......... .......... .......... .......... .......... .......... ..........
Wellcome         .......... .......... .......... .......... .......... .......... .......... ..........
D6               .......... .......... .......... .......... .......... .......... .......... ..........
T996             .......... .......... .......... .......... .......... .......... .......... ..........
T9102            .......... .......... .......... .......... .......... .......... .......... ..........
K1               .......... .......... .......... .......... .......... .......... .......... ..........
Dd2              .......... .......... .......... .......... .......... .......... .......... ..........
FCC2             .......... .......... .......... .......... .......... .......... .......... ..........
D10              .......... .......... .......... .......... .......... .......... .......... ..........
HB3              .......... .......... .......... .......... .......... .......... .......... ..........
7G8              .......... .......... .......... .......... .......... .......... .......... ..........
P.reichenowi     .......... .......... .......... .......... .......... .........A .....G.... ..........

                      730        740        750        760        770        780        790        800
                 ....|....| ....|....| ....|....| ....|....| ....|....| ....|....| ....|....| ....|....|
MAL13_P1.173     ATAGTAAATA TGCTCAAAAA AGCTTTAGAA GAAGAGAAAT TTAGTGATGA ATTTAAAAAT TTTATTTATG GTATTTATGC
3D7              .......... .......... .......... .......... .......... .......... .......... ..........
RO33             .......... .......... .......... .......... .......... .......... .......... ..........
Palo Alto        .......... .......... .......... .......... .......... .......... .......... ..........
FCR3             .......... .......... .......... .......... .......... .......... .......... ..........
Wellcome         .......... .......... .......... .......... .......... .......... .......... ..........
D6               .......... .......... .......... .......... .......... .......... .......... ..........
T996             .......... .......... .......... .......... .......... .......... .......... ..........
T9102            .......... .......... .......... .......... .......... .......... .......... ..........
K1               .......... .......... .......... .......... .......... .......... .......... ..........
Dd2              .......... .......... .......... .......... .......... .......... .......... ..........
FCC2             .......... .......... .......... .......... .......... .......... .......... ..........
D10              .......... .......... .......... .......... .......... .......... .......... ..........
HB3              .......... .......... .......... .......... .......... .......... .......... ..........
7G8              .......... .......... .......... .......... .......... .......... .......... ..........
P.reichenowi     .......... .......... .......... .......... .......... .......... .......... ..........

                      810        820        830        840        850        860        870        880
                 ....|....| ....|....| ....|....| ....|....| ....|....| ....|....| ....|....| ....|....|
MAL13_P1.173     ATATGCCAAG AAACATAATT ATTTAAGACT TATAGATTCT AATAGGGACA TATATAAAAA CGTTTTTGAA AATGCAACCA
3D7              .......... .......... .......... .......... .......... .......... .......... ..........
RO33             .......... .......... .......... .......... .......... .......... .......... ..........
Palo Alto        .......... .......... .......... .......... .......... .......... .......... ..........
FCR3             .......... .......... .......... .......... .......... .......... .......... ..........
Wellcome         .......... .......... .......... .......... .......... .......... .......... ..........
D6               .......... .......... .......... .......... .......... .......... .......... ..........
T996             .......... .......... .......... .......... .......... .......... .......... ..........
T9102            .......... .......... .......... .......... .......... .......... .......... ..........
K1               .......... .......... .......... .......... .......... .......... .......... ..........
Dd2              .......... .......... .......... .......... .......... .......... .......... ..........
FCC2             .......... .......... .......... .......... .......... .......... .......... ..........
D10              .......... .......... .......... .......... .......... .......... .......... ..........
HB3              .......... .......... .......... .......... .......... .......... .......... ..........
7G8              .......... .......... .......... .......... .......... .......... .......... ..........
P.reichenowi     .......... .......... .......... .C........ .......... C......... G......... ..........
```

```
                           890        900        910        920        930
                    ....|....| ....|....| ....|....| ....|....| ....|....|
MAL13_P1.173        ACTTATTAGA TACTTTACAG ATGAAACTTA AGAGAGTTCC ATCACAATAA
3D7                 .......... .......... .......... .......... ..........
RO33                .......... .......... .......... .......... ..........
Palo Alto           .......... .......... .......... .......... ..........
FCR3                .......... .......... .......... .......... ..........
Wellcome            .......... .......... .......... .......... ..........
D6                  .......... .......... .......... .......... ..........
T996                .......... .......... .......... .......... ..........
T9102               .......... .......... .......... .......... ..........
K1                  .......... .......... .......... .......... ..........
Dd2                 .......... .......... .......... .......... ..........
FCC2                .......... .......... .......... .......... ..........
D10                 .......... .......... .......... .......... ..........
HB3                 .......... .......... .......... .......... ..........
7G8                 .......... .......... .......... .......... ..........
P.reichenowi        .......... .........A .....T.... .......... ..........
```

MRSP5

```
                            10         20         30         40         50         60         70         80
                    ....|....| ....|....| ....|....| ....|....| ....|....| ....|....| ....|....| ....|....|
PF13_0191           ATGCAAAGTG AATTCTTCAT TTGTGTTACC TTTTTTTTTG TGCTTTTACA TTATATTTCT TGTAATAAAC CAACACGTAA
3D7                                                                             ...... .......... ..........
RO33                                                                            ...... .......... ..........
Palo Alto                                                                       ...... .......... ..........
FCR3                                                                            ...... .......... ..........
Wellcome                                                                        ...... .......... ..........
D6                                                                              ...... .......... ..........
T996                                                                            ...... .......... ..........
T9102                                                                           ...... .......... ..........
K1                                                                              ...... .......... ..........
Dd2                                                                             ...... .......... ..........
D10                                                                             ...... .......... ..........
FCC2                                                                            ...... .......... ..........
HB3                                                                             ...... .......... ..........
7G8                                                                             ...... .......... ..........
P.reichenowi                                                                    ...... .......... ..........

                            90        100        110        120        130        140        150        160
                    ....|....| ....|....| ....|....| ....|....| ....|....| ....|....| ....|....| ....|....|
PF13_0191           TATATCTGTG AAATCTAATA AAGATAAAGA TGAATTGAAC AATATAAAAG AAAAGTTAGA TTTGATAAAT AATTCTATAA
3D7                 .......... .......... .......... .......... .......... .......... .......... ..........
RO33                .......... .......... .......... .......... .......... .......... .......... ..........
Palo Alto           .......... .......... .......... .......... .......... .......... .......... ..........
FCR3                .......... .......... .......... .......... .......... .......... .......... ..........
Wellcome            .......... .......... .......... .......... .......... .......... .......... ..........
D6                  .......... .......... .......... .......... .......... .......... .......... ..........
T996                .......... .......... .......... .......... .......... .......... .......... ..........
T9102               .......... .......... .......... .......... .......... .......... .......... ..........
K1                  .......... .......... .......... .......... .......... .......... .......... ..........
Dd2                 .......... .......... .......... .......... .......... .......... .......... ..........
D10                 .......... .......... .......... .......... .......... .......... .......... ..........
FCC2                .......... .......... .......... .......... .......... .......... .......... ..........
HB3                 .......... .......... .......... .......... .......... .......... .......... ..........
7G8                 .......... .......... .......... .......... .......... .......... .......... ..........
P.reichenowi        .......... .......... .......... .......... .......... .......... .......... ..........

                           170        180        190        200        210        220        230        240
                    ....|....| ....|....| ....|....| ....|....| ....|....| ....|....| ....|....| ....|....|
PF13_0191           AAGATAAAGT TATTGAAAAT TTTAAAGAAG ATATTGAATT ATTAAAAAAA AAGGTAGACG ATTTGGAAAA AAGGAAAAGT
3D7                 .......... .......... .......... .......... .......... .......... .......... ..........
RO33                .......... .......... .......... .......... .......... .......... .......... ..........
Palo Alto           .......... .......... .......... .......... .......... .......... .......... ..........
FCR3                .......... .......... .......... .......... .......... .......... .......... ..........
Wellcome            .......... .......... .......... .......... .......... .......... .......... ..........
D6                  .......... .......... .......... .......... .......... .......... .......... ..........
T996                .......... .......... .......... .......... .......... .......... .......... ..........
T9102               .......... .......... .......... .......... .......... .......... .......... ..........
K1                  .......... .......... .......... .......... .......... .......... .......... ..........
Dd2                 .......... .......... .......... .......... .......... .......... .......... ..........
D10                 .......... .......... .......... .......... .......... .......... .......... ..........
FCC2                .......... .......... .......... .......... .......... .......... .......... ..........
HB3                 .......... .......... .......... .......... .......... .......... .......... ..........
7G8                 .......... .......... .......... .......... .......... .......... .......... ..........
P.reichenowi        .......... .......... .......... .......... .......... .......... .......T.. ..........
```

```
                         250        260        270        280        290        300        310        320
                  ....|....| ....|....| ....|....| ....|....| ....|....| ....|....| ....|....| ....|....|
PF13_0191         GATAACACAC TCGGGAAAAG GCAAAAAAAA GAAGATGATG ATGATGAAGA AGAGACGGAT GAAGATGATG ACGAAGATTC
3D7               .......... .......... .......... .......... .......... .......... .......... ..........
RO33              .......... .......... .......... .......... .......... .......... .......... ..........
Palo Alto         .......... .......... .......... .......... .......... .......... .......... ..........
FCR3              .......... .......... .......... .......... .......... .......... .......... ..........
Wellcome          .......... .......... .......... .......... .......... .......... .......... ..........
D6                .......... .......... .......... .......... .......... .......... .......... ..........
T996              .......... .......... .......... .......... .......... .......... .......... ..........
T9102             .......... .......... .......... .......... .......... .......... .......... ..........
K1                .......... .......... .......... .......... .......... .......... .......... ..........
Dd2               .......... .......... .......... .......... .......... .......... .......... ..........
D10               .......... .......... .......... .......... .......... .......... .......... ..........
FCC2              .......... .......... .......... .......... .......... .......... .......... ..........
HB3               .......... .......... .......... .......... .......... .......... .......... ..........
7G8               .......... .......... .......... .......... .......... .......... .......... ..........
P.reichenowi      .......... .......... .......... .......... .......... ......A... .......... ..........

                         330        340        350        360        370        380        390        400
                  ....|....| ....|....| ....|....| ....|....| ....|....| ....|....| ....|....| ....|....|
PF13_0191         CGATGAAGAT GACGAAGAAC AAGAAGAATT AAATGTGGAA CCAAAAGAAA GAGAAGATGA ACAGGAAGAA ACAGACGATG
3D7               .......... .......... .......... .......... .......... .......... .......... ..........
RO33              .......... .......... .......... .......... .......... .......... .......... ..........
Palo Alto         .......... .......... .......... .......... .......... .......... .......... ..........
FCR3              .......... .......... .......... .......... .......... .......... .......... ..........
Wellcome          .......... .......... .......... .......... .......... .......... .......... ..........
D6                .......... .......... .......... .......... .......... .......... .......... ..........
T996              .......... .......... .......... .......... .......... .......... .......... ..........
T9102             .......... .......... .......... .......... .......... .......... .......... ..........
K1                .......... .......... .......... .......... .......... .......... .......... ..........
Dd2               .......... .......... .......... .......... .......... .......... .......... ..........
D10               .......... .......... .......... .......... .......... .......... .......... ..........
FCC2              .......... .......... .......... .......... .......... .......... .......... ..........
HB3               .......... .......... .......... .......... .......... .......... .......... ..........
7G8               .......... .......... .......... .......... .......... .......... .......... ..........
P.reichenowi      A......... .......... .......... .......... .......... .......... ...A...... ..........

                         410        420        430        440        450        460        470        480
                  ....|....| ....|....| ....|....| ....|....| ....|....| ....|....| ....|....| ....|....|
PF13_0191         AACAA----- ---------- ------AAAG AAACAGAAGA TGAACAAAAA GAAACAGAAG ATGAACAAAA AGAAACAGAA
3D7               .....----- ---------- ------.... .......... .......... .......... .......... ..........
RO33              ....GGAAGA AACAGACGAT GAACAA.... .......... .......... .......... .......... ..........
Palo Alto         ....GGAAGA AACAGACGAT GAACAA.... .......... .......... .......... .......... ..........
FCR3              .....----- ---------- ------.... .......... .......... .......... .......... ..........
Wellcome          .....----- ---------- ------.... .......... .......... .......... .......... ..........
D6                .....----- ---------- ------.... .......... .......... .......... .......... ..........
T996              .....----- ---------- ------.... .......... .......... .......... .......... ..........
T9102             .....----- ---------- ------.... .......... .......... .......... .......... ..........
K1                .....----- ---------- ------.... .......... .......... .......... .......... ..........
Dd2               .....----- ---------- ------.... .......... .......... .......... .......... ..........
D10               .....----- ---------- ------.... .......... .......... .......... .......... ..........
FCC2              .....----- ---------- ------.... .......... .......... .......... .......... ..........
HB3               .....----- ---------- ------.... .......... .......... .......... ........-- ----------
7G8               .....----- ---------- ------.... .......... .......... .......... .......... ..........
P.reichenowi      .....----- ---------- ------.... .......C.. .......... .....C..C. .......... ......C..C

                         490        500        510        520        530        540        550        560
                  ....|....| ....|....| ....|....| ....|....| ....|....| ....|....| ....|....| ....|....|
PF13_0191         GATGAACAAA AAGAAACAGA AGATGAACAA AAAGAAACAG AAGATGAACA AAAAGAATCG GATGATGAAC AA--------
3D7               .......... .......... .......... .......... .......... .......... .......... ..--------
RO33              .......... .......... .......... .......... .......... .......... .......... ..--------
Palo Alto         .........- ---------- ---------- ---------- ---------- -......... .......... ..--------
FCR3              .........- ---------- ---------- ---------- ---------- -......... .......... ..--------
Wellcome          .........- ---------- ---------- ---------- ---------- -......... .......... ..--------
D6                .........- ---------- ---------- ---------- ---------- -......... .......... ..AAAGAATC
T996              .......... .......... .......... ---------- ---------- -......... .......... ..--------
T9102             .......... .......... .......... ---------- ---------- -......... .......... ..--------
K1                .......... .......... .......... ---------- ---------- -......... .......... ..--------
Dd2               .........- ---------- ---------- ---------- ---------- -......... .......... ..--------
D10               .........- ---------- ---------- ---------- ---------- -......... .......... ..--------
FCC2              .......... .......... .......... .......... .......... .......... .......... ..--------
HB3               ---------- ---------- ---------- ---------- ---------- -......... .......... ..--------
7G8               .......... .......... .......... ---------- ---------- -......... .......... ..--------
P.reichenowi      .......... .......C.. C......... ........C. .C........ .......A.C ..C..C..T. ..AAAGAAAC
```

```
                       570       580       590       600       610       620       630       640
                ....|....| ....|....| ....|....| ....|....| ....|....| ....|....| ....|....| ....|....|
PF13_0191       ---------- ---AAAGAAA CAGAAGATGA ACAA------ ---------- ---------- ---------- ------AAAG
3D7             ---------- ---....... .......... ....------ ---------- ---------- ---------- ------....
RO33            ---------- ---....... .......... ....------ ---------- ---------- ---------- ------....
Palo Alto       ---------- ---....... .......... ....AAAGAA ACAGAAGATG AACAA----- ---------- ------....
FCR3            ---------- ---....... .......... ....AAAGAA ACAGAAGATG AACAAAAAGA AACAGAAGAT GAACAA....
Wellcome        ---------- ---....... .......... ....AAAGAA ACAGAAGATG AACAAAAAGA AACAGAAGAT GAACAA....
D6              GGATGATGAA CAA....... .......... ....------ ---------- ---------- ---------- ------....
T996            ---------- ---....... .......... ....------ ---------- ---------- ---------- ------....
T9102           ---------- ---....... .......... ....AAAGAA ACAGAAGATG AACAA----- ---------- ------....
K1              ---------- ---....... .......... ....AAAGAA ACAGAAGATG AACAA----- ---------- ------....
Dd2             ---------- ---....... .......... ....AAAGAA ACAGAAGATG AACAA----- ---------- ------....
D10             ---------- ---....... .......... ....AAAGAA ACAGAAGATG AACAA----- ---------- ------....
FCC2            ---------- ---....... .......... ....------ ---------- ---------- ---------- ------....
HB3             ---------- ---....... .......... ....------ ---------- ---------- ---------- ------....
7G8             ---------- ---....... .......... ....AAAGAA ACAGAAGATG AACAA----- ---------- ------....
P.reichenowi    CGACGACGAA CAA....... ....T..... ....------ ---------- ---------- ---------- ------....

                       650       660       670       680       690       700       710       720
                ....|....| ....|....| ....|....| ....|....| ....|....| ....|....| ....|....| ....|....|
PF13_0191       AAACAGAAGA TGAAGCGTCT GAAGAATATT CTGATAATGA AGAAGACGAT GAAGAAGATG AAGAAGATGA TGAAGAAGAA
3D7             .......... .......... .......... .......... .......... .......... .......... ..........
RO33            .......... .......... .......... .......... .......... ......---- -----..... ..........
Palo Alto       .......... .......... .......... .......... .......... ......---- -----..... ..........
FCR3            .......... .......... .......... .......... .......... ......---- -----..... ..........
Wellcome        .......... .......... .......... .......... .......... ......---- -----..... ..........
D6              .......... .......... .......... .......... .......... .......... .......... ..........
T996            .......... .......... .......... .......... .......... .......... .......... ..........
T9102           .......... .......... .......... .......... .......... .......... .......... ..........
K1              .......... .......... .......... .......... .......... .......... .......... ..........
Dd2             .......... .......... .......... .......... .......... .......... .......... ..........
D10             .......... .......... .......... .......... .......... .......... .......... ..........
FCC2            .......... .......... .......... .......... .......... .......... .......... ..........
HB3             .......... .......... .......... .......... .......... .......... .......... ..........
7G8             .......... .......... .......... .......... .......... .......... .......... ..........
P.reichenowi    .CGTC..... A......... .......... .......... ......A... ......---- --------.. ATC.......

                       730       740       750       760       770       780       790       800
                ....|....| ....|....| ....|....| ....|....| ....|....| ....|....| ....|....| ....|....|
PF13_0191       AACGAAGAAA ATAACGATAA TGAAACAAAT GAAGAAAATG AAGATAATGA TGAAAATGAA GATAATGATG AAAATGAAGA
3D7             .......... .......... .......... .......... .......... .......... .......... ..........
RO33            .......... .......... .......... .......... .......... .......--- ---------- -----.....
Palo Alto       .......... .......... .......... .......... .......... .......--- ---------- -----.....
FCR3            .......... .......... .......... .......... .......... .......--- ---------- -----.....
Wellcome        .......... .......... .......... .......... .......... .......--- ---------- -----.....
D6              .......... .......... .......... .......... .......... .......... .......... ..........
T996            .......... .......... .......... .......... .......... .......... .......... ..........
T9102           .......... .......... .......... .......... .......... .......--- ---------- -----.....
K1              .......... .......... .......... .......... .......... .......--- ---------- -----.....
Dd2             .......... .......... .......... .......... .......... .......--- ---------- -----.....
D10             .......... .......... .......... .......... .......... .......--- ---------- -----.....
FCC2            .......... .......... .......... .......... .......... .......--- ---------- -----.....
HB3             .......... .......... .......... .......... .......... .......--- ---------- -----.....
7G8             .......... .......... .......... .......... .......... .......--- ---------- -----.....
P.reichenowi    .......... .......... ...C...... .......... .......... .......--- ---------- -----.....

                       810       820       830       840       850       860       870       880
                ....|....| ....|....| ....|....| ....|....| ....|....| ....|....| ....|....| ....|....|
PF13_0191       AGAAATAGAA GTTACTGATG TTGAATTTGT AGGACAAAGT ACAAATAAAA ATGTAAGAAA TAATATGATC AGAAATTCTA
3D7             .......... .......... .......... .......... .......... .......... .......... ..........
RO33            .......... .......... .......... .......... .......... .......... .......... ..........
Palo Alto       .......... .......... .......... .......... .......... .......... .......... ..........
FCR3            .......... .......... .......... .......... .......... .......... .......... ..........
Wellcome        .......... .......... .......... .......... .......... .......... .......... ..........
D6              .......... .......... .......... .......... .......... .......... .......... ..........
T996            .......... .......... .......... .......... .......... .......... .......... ..........
T9102           .......... .......... .......... .......... .......... .......... .......... ..........
K1              .......... .......... .......... .......... .......... .......... .......... ..........
Dd2             .......... .......... .......... .......... .......... .......... .......... ..........
D10             .......... .......... .......... .......... .......... .......... .......... ..........
FCC2            .......... .......... .......... .......... .......... .......... .......... ..........
HB3             .......... .......... .......... .......... .......... .......... .......... ..........
7G8             .......... .......... .......... .......... .......... .......... .......... ..........
P.reichenowi    .........G ....A..... .......... .......... .......... .......... .......... ..........
```

```
                      890       900       910       920       930       940       950       960
              ....|....| ....|....| ....|....| ....|....| ....|....| ....|....| ....|....| ....|....|
PF13_0191     ATAAGGATAT AAAATCTTCT TCTCAAAATT CATCAATCAA AGCTCAAAAC AGTTCAACCA AAATTGGAAA TACACCAACG
3D7           .......... .......... .......... .......... .......... .......... .......... ..........
RO33          .......... .......... .......... .......... .......... .......... .......... ..........
Palo Alto     .......... .......... .......... .......... .......... .......... .......... ..........
FCR3          .......... .......... .......... .......... .......... .......... .......... ..........
Wellcome      .......... .......... .......... .......... .......... .......... .......... ..........
D6            .......... .......... .......... .......... .......... .......... .......... ..........
T996          .......... .......... .......... .......... .......... .......... .......... ..........
T9102         .......... .......... .......... .......... .......... .......... .......... ..........
K1            .......... .......... .......... .......... .......... .......... .......... ..........
Dd2           .......... .......... .......... .......... .......... .......... .......... ..........
D10           .......... .......... .......... .......... .......... .......... .......... ..........
FCC2          .......... .......... .......... .......... .......... .......... .......... ..........
HB3           .......... .......... .......... .......... .......... .......... .......... ..........
7G8           .......... .......... .......... .......... .......... .......... .......... ..........
P.reichenowi  .......... .......... .......... .......... .......... .......... .......... .........T

                      970       980       990      1000      1010      1020      1030      1040
              ....|....| ....|....| ....|....| ....|....| ....|....| ....|....| ....|....| ....|....|
PF13_0191     AAATTATCTA CACAAAATAC AAAATCAAAT TCAACATCTA ACCAATTAAT AACTCAATTA CAAAGCGAAA AATCATCCTC
3D7           .......... .......... .......... .......... .......... .......... .......... ..........
RO33          .......... .......... .......... .......... .......... .......... .......... ..........
Palo Alto     .......... .......... .......... .......... .......... .......... .......... ..........
FCR3          .......... .......... .......... .......... .......... .......... .......... ..........
Wellcome      .......... .......... .......... .......... .......... .......... .......... ..........
D6            .......... .......... .......... .......... .......... .......... .......... ..........
T996          .......... .......... .......... .......... .......... .......... .......... ..........
T9102         .......... .......... .......... .......... .......... .......... .......... ..........
K1            .......... .......... .......... .......... .......... .......... .......... ..........
Dd2           .......... .......... .......... .......... .......... .......... .......... ..........
D10           .......... .......... .......... .......... .......... .......... .......... ..........
FCC2          .......... .......... .......... .......... .......... .......... .......... ..........
HB3           .......... .......... .......... .......... .......... .......... .......... ..........
7G8           .......... .......... .......... .......... .......... .......... .......... ..........
P.reichenowi  .......... .......... .......... .......... .......... .......... .......... .....G.T..

                     1050      1060      1070      1080      1090      1100      1110      1120
              ....|....| ....|....| ....|....| ....|....| ....|....| ....|....| ....|....| ....|....|
PF13_0191     AAAAGTAGAT AACAATAAAA ATAATACAAA TGAAATAAAA TATATGGATA AACTTTGTGA TGACGTTCTT ACTGAATTAA
3D7           .......... .......... .......... .......... .......... .......... .......... ..........
RO33          .......... .......... .......... .......... .......... .......... .......... ..........
Palo Alto     .......... .......... .......... .......... .......... .......... .......... ..........
FCR3          .......... .......... .......... .......... .......... .......... .......... ..........
Wellcome      .......... .......... .......... .......... .......... .......... .......... ..........
D6            .......... .......... .......... .......... .......... .......... .......... ..........
T996          .......... .......... .......... .......... .......... .......... .......... ..........
T9102         .......... .......... .......... .......... .......... .......... .......... ..........
K1            .......... .......... .......... .......... .......... .......... .......... ..........
Dd2           .......... .......... .......... .......... .......... .......... .......... ..........
D10           .......... .......... .......... .......... .......... .......... .......... ..........
FCC2          .......... .......... .......... .......... .......... .......... .......... ..........
HB3           .......... .......... .......... .......... .......... .......... .......... ..........
7G8           .......... .......... .......... .......... .......... .......... .......... ..........
P.reichenowi  .......... .......... .......... ....G..... .......... .......C.. ....C..... ..........

                     1130      1140      1150      1160      1170      1180      1190      1200
              ....|....| ....|....| ....|....| ....|....| ....|....| ....|....| ....|....| ....|....|
PF13_0191     AGGAAAAGGA TAATGTAGAT AATAATATGA ATCATAGTAA ATATAATAAT CTCAAAAAGG AATTTTCTAC TTTTACTATG
3D7           .......... .......... .......... .......... .......... .......... .......... ..........
RO33          .......... .......... .......... .......... .......... .......... .......... ..........
Palo Alto     .......... .......... .......... .......... .......... .......... .......... ..........
FCR3          .......... .......... .......... .......... .......... .......... .......... ..........
Wellcome      .......... .......... .......... .......... .......... .......... .......... ..........
D6            .......... .......... .......... .......... .......... .......... .......... ..........
T996          .......... .......... .......... .......... .......... .......... .......... ..........
T9102         .......... .......... .......... .......... .......... .......... .......... ..........
K1            .......... .......... .......... .......... .......... .......... .......... ..........
Dd2           .......... .......... .......... .......... .......... .......... .......... ..........
D10           .......... .......... .......... .......... .......... .......... .......... ..........
FCC2          .......... .......... .......... .......... .......... .......... .......... ..........
HB3           .......... .......... .......... .......... .......... .......... .......... ..........
7G8           .......... .......... .......... .......... .......... .......... .......... ..........
P.reichenowi  .......... .....C.... .......... .......... .......... .......... .......... ..........
```

```
                                 1210       1220       1230       1240       1250       1260       1270       1280
                             ....|....| ....|....| ....|....| ....|....| ....|....| ....|....| ....|....| ....|....|
PF13_0191                    AATCAAAATG AATGTGATTT AATAAAAAAG TTGATTATTA CCTTTTCTCA AGAAAATGTA GAAATGAAAA GAGATTCCAT
3D7                          .......... .......... .......... .......... .......... .......... .......... ..........
RO33                         .......... .......... .......... .......... .......... .......... .......... ..........
Palo Alto                    .......... .......... .......... .......... .......... .......... .......... ..........
FCR3                         .......... .......... .......... .......... .......... .......... .......... ....G.....
Wellcome                     .......... .......... .......... .......... .......... .......... .......... ....G.....
D6                           .......... .......... .......... .......... .......... .......... .......... ..........
T996                         .......... .......... .......... .......... .......... .......... .......... ..........
T9102                        .......... .......... .......... .......... .......... .......... .......... ..........
K1                           .......... .......... .......... .......... .......... .......... .......... ..........
Dd2                          .......... .......... .......... .......... .......... .......... .......... ..........
D10                          .......... .......... .......... .......... .......... .......... .......... ..........
FCC2                         .......... .......... .......... .......... .......... .......... .......... ..........
HB3                          .......... .......... .......... .......... .......... .......... .......... ..........
7G8                          .......... .......... .......... .......... .......... .......... .......... ..........
P.reichenowi                 .......... ...A...C.. .......... .......... .......... .......... .......... ..........

                                    1290       1300       1310       1320       1330       1340       1350       1360
                             ....|....| ....|....| ....|....| ....|....| ....|....| ....|....| ....|....| ....|....|
PF13_0191                    AAAAGAAATT TTTTTAAAAG CTTTGGATGA TAAAAAATAC CGTGAAGTAT TTAAAAACTT CATGTATGGT GTATATAGTT
3D7                          .......... .......... .......... .......... .......... .......... .......... ..........
RO33                         .......... .......... .......... .......... .......... .......... .......... ..........
Palo Alto                    .......... .......... .......... .......... .......... .......... .......... ..........
FCR3                         .......... .......... .......... .......... .......... .......... .......... ..........
Wellcome                     .......... .......... .......... .......... .......... .......... .......... ..........
D6                           .......... .......... .......... .......... .......... .......... .......... ..........
T996                         .......... .......... .......... .......... .......... .......... .......... ..........
T9102                        .......... .......... .......... .......... .......... .......... .......... ..........
K1                           .......... .......... .......... .......... .......... .......... .......... ..........
Dd2                          .......... .......... .......... .......... .......... .......... .......... ..........
D10                          .......... .......... .......... .......... .......... .......... .......... ..........
FCC2                         .......... .......... .......... .......... .......... .......... .......... ..........
HB3                          .......... .......... .......... .......... .......... .......... .......... ..........
7G8                          .......... .......... .......... .......... .......... .......... .......... ..........
P.reichenowi                 .......... .......G.. .......... .........T A......... .......... T......... ..........

                                    1370       1380       1390       1400       1410       1420       1430       1440
                             ....|....| ....|....| ....|....| ....|....| ....|....| ....|....| ....|....| ....|....|
PF13_0191                    ATGCAAAACG TCATAATTAT TTGGATATTG AAAAAATGGA AAAAAATGAA AGGGCCTATA AAAAATTATT CGAAAACACA
3D7                          .......... .......... .......... .........
RO33                         .......... .......... .......... .........
Palo Alto                    .......... .......... .......... .........
FCR3                         .......... .......... .......... .........
Wellcome                     .......... .......... .......... .........
D6                           .......... .......... .......... .........
T996                         .......... .......... .......... .........
T9102                        .......... .......... .......... .........
K1                           .......... .......... .......... .........
Dd2                          .......... .......... .......... .........
D10                          .......... .......... .......... .........
FCC2                         .......... .......... .......... .........
HB3                          .......... .......... .......... .........
7G8                          .......... .......... .......... .........
P.reichenowi                 .......... .......... .......... .......... ....

                                    1450       1460
                             ....|....| ....|....| ....
PF13_0191                    CTTAACTTAT TAGATACCAT ATGA
3D7
RO33
Palo Alto
FCR3
Wellcome
D6
T996
T9102
K1
Dd2
D10
FCC2
HB3
7G8
P.reichenowi
```

PF13_0192

```
                       10         20         30         40         50         60         70         80
               ....|....| ....|....| ....|....| ....|....| ....|....| ....|....| ....|....| ....|....|
PF13_0192      ATGACGGTAC AGTATAATAG AATTATTTTT CTACCAATCA GTTCTAATTT TTTTTTATAT AGTATTTTAT TTTTATTAGA
3D7
RO33
Palo Alto
FCR3
Wellcome
D6
T996
T9102
K1
Dd2
D10
FCC2
HB3
7G8
P.reichenowi

                       90        100        110        120        130        140        150        160
               ....|....| ....|....| ....|....| ....|....| ....|....| ....|....| ....|....| ....|....|
PF13_0192      ATATTTAAAG GATTCCCAAA AAAAAAAAAA AAAAAAAAAA ATGAAAAGCA AAAAAATAAT ATGTTCATCT TGCTTATTTT
3D7                                                                 . .......... .......... ..........
RO33                                                                . .......... .......... ..........
Palo Alto                                                           . .......... .......... ..........
FCR3                                                                . .......... .......... ..........
Wellcome                                                            . .......... .......... ..........
D6                                                                  . .......... .......... ..........
T996                                                                . .......... .......... ..........
T9102                                                               . .......... .......... ..........
K1                                                                  . .......... .......... ..........
Dd2                                                                 . .......... .......... ..........
D10                                                                 . .......... .......... ..........
FCC2                                                                . .......... .......... ..........
HB3                                                                 . .......... .......... ..........
7G8                                                                 . .......... .......... ..........
P.reichenowi                                                        . .......... .......... ..........

                      170        180        190        200        210        220        230        240
               ....|....| ....|....| ....|....| ....|....| ....|....| ....|....| ....|....| ....|....|
PF13_0192      TAATATTTTT AAGTGTAATA TTTTGTAGTG AACCAGATAC AAATTCATTT GATGAAAATG TAAAGAAGAA TGAAGTTTTT
3D7            .......... .......... .......... .......... .......... .......... .......... ..........
RO33           .......... .......... .......... .......... .......... .......... .......... ..........
Palo Alto      .......... .......... ........A. .......... .......... .......... .......... ..........
FCR3           .......... .......... .......... .......... .......... .......... .......... ..........
Wellcome       .......... .......... .......... .......... .......... .......... .......... ..........
D6             .......... .......... .......... .......... .......... .......... .......... ..........
T996           .......... .......... .......... .......... .......... .......... .......... ..........
T9102          .......... .......... .......... .......... .......... .......... .......... ..........
K1             .......... .......... .......... .......... .......... .......... .......... ..........
Dd2            .......... .......... .......... .......... .......... .......... .......... ..........
D10            .......... .......... .......... .......... .......... .......... .......... ..........
FCC2           .......... .......... .......... .......... .......... .......... .......... ..........
HB3            .......... .......... .......... .......... .......... .......... .......... ..........
7G8            .......... .......... .......... .......... .......... .......... .......... ..........
P.reichenowi   .......A.. .......... .......... .......... .......... .......... .......... ..........

                      250        260        270        280        290        300        310        320
               ....|....| ....|....| ....|....| ....|....| ....|....| ....|....| ....|....| ....|....|
PF13_0192      AATGCCTTAA ATGAACATTT AGAAAGTATA AGTAATATCG TAAAAGTAAA TATTATGGAT GCCCTTTCAA ATAACCCTTC
3D7            .......... .......... .......... .......... .......... .......... .......... ..........
RO33           .......... .......... .......... .......... .......... .......... .......... ..........
Palo Alto      .......... .......... .......... .......... .......... .......... .......... ..........
FCR3           .......... .......... .......... .......... .......... .......... .......... ..........
Wellcome       .......... .......... .......... .......... .......... .......... .......... ..........
D6             .......... .......... .......... .......... .......... .......... .......... ..........
T996           .......... .......... .......... .......... .......... .......... .......... ..........
T9102          .......... .......... .......... .......... .......... .......... .......... ..........
K1             .......... .......... .......... .......... .......... .......... .......... ..........
Dd2            .......... .......... .......... .......... .......... .......... .......... ..........
D10            .......... .......... .......... .......... .......... .......... .......... ..........
FCC2           .......... .......... .......... .......... .......... .......... .......... ..........
HB3            .......... .......... .......... .......... .......... .......... .......... ..........
7G8            .......... .......... .......... .......... .......... .......... .......... ..........
P.reichenowi   .....T.... .......... .......... .......... ......C... .......... .......... ..........
```

```
                          330        340        350        360        370        380        390        400
                 ....|....| ....|....| ....|....| ....|....| ....|....| ....|....| ....|....| ....|....|
PF13_0192        GTTAATAAAA AAGACATACG AAGCTGTTGA AATAAATGAT GATGATTATG TTCTGGAATA TGTTGGGGAT TCGACAGGAA
3D7              .......... .......... .......... .......... .......... .......... .......... ..........
RO33             .......... .......... .......... .......... .......... .......... .......... ..........
Palo Alto        .......... .......... .......... .......... .......... .......... .......... ..........
FCR3             .......... .......... .......... .......... .......... .......... .......... ..........
Wellcome         .......... .......... .......... .......... .......... .......... .......... ..........
D6               .......... .......... .......... .......... .......... .......... .......... ..........
T996             .......... .......... .......... .......... .......... .......... .......... ..........
T9102            .......... .......... .......... .......... .......... .......... .......... ..........
K1               .......... .......... .......... .......... .......... .......... .......... ..........
Dd2              .......... .......... .......... .......... .......... .......... .......... ..........
D10              .......... .......... .......... .......... .......... .......... .......... ..........
FCC2             .......... .......... .......... .......... .......... .......... .......... ..........
HB3              .......... .......... .......... .......... .......... .......... .......... ..........
7G8              .......... .......... .......... .......... .......... .......... .......... ..........
P.reichenowi     T...T..... .......... .......... .......... .......... .......... .......... ..........

                          410        420        430        440        450        460        470        480
                 ....|....| ....|....| ....|....| ....|....| ....|....| ....|....| ....|....| ....|....|
PF13_0192        AATATGGAGA GGGCTCTATA TTCTATGATG AAAGTAAGAA ATATAATTAT AGAAAGTATT TAGATATAGA AAATGAATTA
3D7              .......... .......... .......... .......... .......... .......... .......... ..........
RO33             .......... .......... .......... .......... .......... .......... .......... ..........
Palo Alto        .......... .......... .......... .......... .......... .......... .......... ..........
FCR3             .......... .......... .......... .......... .......... .......... .......... ..........
Wellcome         .......... .......... .......... .......... .......... .......... .......... ..........
D6               .......... .......... .......... .......... .......... .......... .......... ..........
T996             .......... .......... .......... .......... .......... .......... .......... ..........
T9102            .......... .......... .......... .......... .......... .......... .......... ..........
K1               .......... .......... .......... .......... .......... .......... .......... ..........
Dd2              .......... .......... .......... .......... .......... .......... .......... ..........
D10              .......... .......... .......... .......... .......... .......... .......... ..........
FCC2             .......... .......... .......... .......... .......... .......... .......... ..........
HB3              .......... .......... .......... .......... .......... .......... .......... ..........
7G8              .......... .......... .......... .......... .......... .......... .......... ..........
P.reichenowi     .......... .......... ..T....... .......... .......... .......... .......... ..........

                          490        500        510        520        530        540        550        560
                 ....|....| ....|....| ....|....| ....|....| ....|....| ....|....| ....|....| ....|....|
PF13_0192        CGAAATATGA AAGGTGAAGA TGATGATGAT TTTGATGAAG ATGATGATGA TTTT------ ---------- ----------
3D7              .......... .......... .......... .......... .......... ....------ ---------- ----------
RO33             .......... .......... .......... .......... .......... ....------ ---------- ----------
Palo Alto        .......... .......... .......... .......... .......... ....------ ---------- ----------
FCR3             .......... .......... .......... .......... .......... ....GATGAA GATGATGATG ATTTT-----
Wellcome         .......... .......... .......... .......... .......... ....GATGAA GATGATGATG ATTTT-----
D6               .......... .......... .......... .......... .......... ....GATGAA GATGATGATG ATTTT-----
T996             .......... .......... .......... .......... .......... ....------ ---------- ----------
T9102            .......... .......... .......... .......... .......... ....GATGAA GATGATGATG ATTTT-----
K1               .......... .......... .......... .......... .......... ....GATGAA GATGATGATG ATTTT-----
Dd2              .......... .......... .......... .......... .......... ....GATGAA GATGATGATG ATTTTGATGA
D10              .......... .......... .......... .......... .......... ....GATGAA GATGATGATG ATTTT-----
FCC2             .......... .......... .......... .......... .......... ....------ ---------- ----------
HB3              .......... .......... .......... .......... .......... ....GATGAA GATGATGATG ATTTT-----
7G8              .......... .......... .......... .......... .......... ....------ ---------- ----------
P.reichenowi     .......... .......... .......--- ---------- ---------- ---------- ---------- ----------

                          570        580        590        600        610        620        630        640
                 ....|....| ....|....| ....|....| ....|....| ....|....| ....|....| ....|....| ....|....|
PF13_0192        ---------- ---------- ---------- -------GAT GAAGATGATG AAGATGATGA AGATGATGAA GATGATGAA-
3D7              ---------- ---------- ---------- ---------- ------.... .......... .......... .........G
RO33             ---------- ---------- ---------- ---------- ------.... .......... .......... .........G
Palo Alto        ---------- ---------- ---------- ---------- ---------- ---------- ---------- ---......G
FCR3             ---------- ---------- ---------- -------... .......... .......... .......... .........-
Wellcome         ---------- ---------- ---------- ---------- ------.... .......... .......... .........G
D6               ---------- ---------- ---------- ---------- ---------- ---------- ---------- ---......G
T996             ---------- ---------- ---------- ---------- ------.... .......... .......... .........G
T9102            ---------- ---------- ---------- ---------- ---------- ---------- -.....A..T .........G
K1               ---------- ---------- ---------- ---------- ---------- ---------- -.....A..T .........G
Dd2              AGATGATGAT GATTTTGATG AAGATGATGA TGATTTT... .......... ..-------- ---------- ----------
D10              ---------- ---------- ---------- -------... .......... .......... .......... .........-
FCC2             ---------- ---------- ---------- -------... .......... .......... .......... .........-
HB3              ---------- ---------- ---------- -------... .......... .......... .--------- ----------
7G8              ---------- ---------- ---------- -------... .......... .......... .......... .........G
P.reichenowi     ---------- ---------- ---------- ---------- ---------- ---------- ---------- ---......G
```

```
                        650        660        670        680        690        700        710        720
                ....|....| ....|....| ....|....| ....|....| ....|....| ....|....| ....|....| ....|....|
PF13_0192       --------GA TGATGTAGAT GATGAAGATG AT-------- ---------- CTAGATGTTG AAGATAATGT AGATGATGAA
3D7             ATGATGAA.. .......... .......... ..-------- ---------- .......... .......... ..........
RO33            ATGATGAA.. .......... .......... ..-------- ---------- G......... .......... ..........
Palo Alto       ATGATGAA.. .......... .......... ..GTAGATGA TGAAGATGAT .......... .......... ..........
FCR3            --------.. .......... .......... ..-------- ---------- .......... .......... ..........
Wellcome        ATGATGAA.. .......... .......... ..-------- ---------- .......... .......... ..........
D6              ATGATGAA.. .......... .......... ..GTAGATGA TGAAGATGAT G......A.. .......... ..........
T996            ATGATGAA.. .......... .......... ..-------- ---------- .......... .......... ..........
T9102           ATGATGAA.. .......... .......... ..-------- ---------- .......... .......... ..........
K1              ATGATGAA.. .......... .......... ..-------- ---------- .......... .......... ..........
Dd2             --------.. .......... .......... ..-------- ---------- .......... .......... ..........
D10             --------.. .......... .......... ..-------- ---------- .......... .......... ..........
FCC2            --------.. .......... .......... ..-------- ---------- .......... .......... ..........
HB3             --------.. .......... .......... ..-------- ---------- .......... .......... ..........
7G8             ATGATGAA.. .......... .......... ..-------- ---------- .......... .......... ..........
P.reichenowi    ATGATGAA.. .......... .......... ..-------- ---------- G......A.. .....G..T. ..........

                        730        740        750        760        770        780        790        800
                ....|....| ....|....| ....|....| ....|....| ....|....| ....|....| ....|....| ....|....|
PF13_0192       TATGATGATG AGCATAATCA TAATTATAAT ------GATG ACAAATTGAG TGAAAATCCT GAGAAGTATT CAAATTATAA
3D7             .......... .......... .......... ------.... .......... .......... .......... ..........
RO33            .......... .......... .......... ------.... .......... .......... .......... ..........
Palo Alto       .......... .......... .......... ------.... .......... .......... .......... ..........
FCR3            .......... .......... .......... ------.... .......... .......... .......... ..........
Wellcome        .......... .......... .......... ------.... .......... .......... .......... ..........
D6              .......... .......... .......... ------.... .......... .......... .......... ..........
T996            .......... .......... .......... ------.... .......... .......... .......... ..........
T9102           .......... .......... .......... ------.... .......... .......... .......... ..........
K1              .......... .......... .......... ------.... .......... .......... .......... ..........
Dd2             .......... .......... .......... ------.... .......... .......... .......... ..........
D10             .......... .......... .......... ------.... .......... .......... .......... ..........
FCC2            .......... .......... .......... ------.... .......... .......... .......... ..........
HB3             .......... .......... .......... ------.... .......... .......... .......... ..........
7G8             .......... .......... .......... ------.... .......... .......... .......... ..........
P.reichenowi    G......... ..T....... ....C..... AATAAT.... ......G.T. .......... .......... ..........

                        810        820        830        840        850        860        870        880
                ....|....| ....|....| ....|....| ....|....| ....|....| ....|....| ....|....| ....|....|
PF13_0192       TAAAAATATA CACGAAGATA AGAAAAAAGA TAATTTGAAT GAACCACATT TTAAACAAAC TCATTATATA TATTCATCAA
3D7             .......... .......... .......... .......... .......... .......... .......... ..........
RO33            .......... .......... .......... .......... .......... .......... .......... ..........
Palo Alto       .......... .......... .......... .......... .......... .......... .......... ..........
FCR3            .......... .......... .......... .......... .......... .......... .......... ..........
Wellcome        .......... .......... .......... .......... .......... .......... .......... ..........
D6              .......... .......... .......... .......... .......... .......... .......... ..........
T996            .......... .......... .......... .......... .......... .......... .......... ..........
T9102           .......... .......... .......... .......... .......... .......... .......... ..........
K1              .......... .......... .......... .......... .......... .......... .......... ..........
Dd2             .......... .......... .......... .......... .......... .......... .......... ..........
D10             .......... .......... .......... .......... .......... .......... .......... ..........
FCC2            .......... .......... .......... .......... .......... .......... .......... ..........
HB3             .......... .......... .......... .......... .......... .......... .......... ..........
7G8             .......... .......... .......... .......... .......... .......... .......... ..........
P.reichenowi    .......... .......... .......... .......... .......... .......... A......... ..........

                        890        900        910        920        930        940        950        960
                ....|....| ....|....| ....|....| ....|....| ....|....| ....|....| ....|....| ....|....|
PF13_0192       ATCACGATAA TAATGAAACA TCTAGATTTC CAAAAAAAAA TGTACCTAAA TATGATGAAA AATTAAATAA TGAATTTAAA
3D7             .......... .......... .......... .......... .......... .......... .......... ..........
RO33            .......... .......... .......... .......... .......... .......... .......... ..........
Palo Alto       .......... .......... .......... .......... .......... .......... .......... ..........
FCR3            .......... .......... .......... .......... .......... .......... .......... ..........
Wellcome        .......... .......... .......... .......... .......... .......... .......... ..........
D6              .......... .......... .......... .......... .......... .......... .......... ..........
T996            .......... .......... .......... .......... .......... .......... .......... ..........
T9102           .......... .......... .......... .......... .......... .......... .......... ..........
K1              .......... .......... .......... .......... .......... .......... .......... ..........
Dd2             .......... .......... .......... .......... .......... .......... .......... ..........
D10             .......... .......... .......... .......... .......... .......... .......... ..........
FCC2            .......... .......... .......... .......... .......... .......... .......... ..........
HB3             .......... .......... .......... .......... .......... .......... .......... ..........
7G8             .......... .......... .......... .......... .......... .......... .......... ..........
P.reichenowi    ....A..... .......... .......... .......... .....A.... .......... .......... ..........
```

```
                             970       980       990      1000      1010      1020      1030      1040
                    ....|....| ....|....| ....|....| ....|....| ....|....| ....|....| ....|....| ....|....|
PF13_0192           ACATATTTAA GAAGACCTGA AAAGAAAGAA GAAACAAAAG AATATCCTAA CAATGGATGT TCTGTAGTTC AAATATCTAT
3D7                 .......... .......... .......... .......... .......... .......... .......... ..........
RO33                .......... .......... .......... .......... .......... .......... .......... ..........
Palo Alto           .......... ......A... .......... .......... .......... .......... .......... ..........
FCR3                .......... .......... .......... .......... .......... .......... .......... ..........
Wellcome            .......... .......... .......... .......... .......... .......... .......... ..........
D6                  .......... ......A... .......... .......... .......... .......... .......... ..........
T996                .......... ......A... .......... .......... .......... .......... .......... ..........
T9102               .......... .......... .......... .......... .......... .......... .......... ..........
K1                  .......... .......... .......... .......... .......... .......... .......... ..........
Dd2                 .......... .......... .......... .......... .......... .......... .......... ..........
D10                 .......... ......A... .......... .......... .......... .......... .......... ..........
FCC2                .......... .......... .......... .......... .......... .......... .......... ..........
HB3                 .......... .......... .......... .......... .......... .......... .......... ..........
7G8                 .......... ......A... .......... .......... .......... .......... .......... ..........
P.reichenowi        .......... ......A... ...T...... .......... .......... .......... .....G.... ..........

                            1050      1060      1070      1080      1090      1100      1110      1120
                    ....|....| ....|....| ....|....| ....|....| ....|....| ....|....| ....|....| ....|....|
PF13_0192           AGTAACTAAT GAAGATTTTT TAAAAAAAGT TAGAGAAAGA AATAAAAAGA GAAATAATAA AAAAAGAACA AATATTTATG
3D7                 .......... .......... .......... .......... .......... .......... .......... ..........
RO33                .......... .......... .......... .......... .......... .......... .......... ..........
Palo Alto           .......... .......... .......... .......... .......... .......... .......... ..........
FCR3                .......... .......... .......... .......... .......... .......... .......... ..........
Wellcome            .......... .......... .......... .......... .......... .......... .......... ..........
D6                  .......... .......... .......... .......... .......... .......... .......... ..........
T996                .......... .......... .......... .......... .......... .......... .......... ..........
T9102               .......... .......... .......... .......... .......... .......... .......... ..........
K1                  .......... .......... .......... .......... .......... .......... .......... ..........
Dd2                 .......... .......... .......... .......... .......... .......... .......... ..........
D10                 .......... .......... .......... .......... .......... .......... .......... ..........
FCC2                .......... .......... .......... .......... .......... .......... .......... ..........
HB3                 .......... .......... .......... .......... .......... .......... .......... ..........
7G8                 .......... .......... .......... .......... .......... .......... .......... ..........
P.reichenowi        .......... .......... .......... .......... .......... .......... .......... ...TA.....

                            1130      1140      1150      1160      1170      1180      1190      1200
                    ....|....| ....|....| ....|....| ....|....| ....|....| ....|....| ....|....| ....|....|
PF13_0192           ATAGTGATGA AGAATCAGAA AGTTCAGAAG AAACAAGTAA AGATCCCTAT TCATCGGGTC CATATACAGT TGATCATAAA
3D7                 .......... .......... .......... .......... .......... .......... .......... ..........
RO33                .......... .......... .......... .......... .......... .......... .......... ..........
Palo Alto           .......... .......... .......... .......... .......... .......... .......... ..........
FCR3                .......... .......... .......... .......... .......... .......... .......... ..........
Wellcome            .......... .......... .......... .......... .......... .......... .......... ..........
D6                  .......... .......... .......... .......... .......... .......... .......... ..........
T996                .......... .......... .......... .......... .......... .......... .......... ..........
T9102               .......... .......... .......... .......... .......... .......... .......... ..........
K1                  .......... .......... .......... .......... .......... .......... .......... ..........
Dd2                 .......... .......... .......... .......... .......... .......... .......... ..........
D10                 .......... .......... .......... .......... .......... .......... .......... ..........
FCC2                .......... .......... .......... .......... .......... .......... .......... ..........
HB3                 .......... .......... .......... .......... .......... .......... .......... ..........
7G8                 .......... .......... .......... .......... .......... .......... .......... ..........
P.reichenowi        .......... .......A.. .......... ......A... .......... .......A.. ...C....A. ..........

                            1210      1220      1230      1240      1250      1260      1270      1280
                    ....|....| ....|....| ....|....| ....|....| ....|....| ....|....| ....|....| ....|....|
PF13_0192           AATGAAAATT GTTTTCCTTA CAAGCAAAAT TCAGATATTC ATCCAAATAT AAATAATGAT ---------- ----------
3D7                 .......... .......... .......... .......... .......... .......... ---------- ----------
RO33                .......... .......... .......... .......... .......... .......... AATAATGAAA ATAATGATAA
Palo Alto           .......... .......... .......... .......... .......... .......... ---------- ----------
FCR3                .......... .......... .......... .......... .......... .......... ---------- ----------
Wellcome            .......... .......... .......... .......... .......... .......... ---------- ----------
D6                  .......... .......... .......... .......... .......... .......... AATAATGAA- ----------
T996                .......... .......... .......... .......... .......... .......... AATAATGATA ATAATGAAAA
T9102               .......... .......... .......... .......... .......... .......... ---------- ----------
K1                  .......... .......... .......... .......... .......... .......... ---------- ----------
Dd2                 .......... .......... .......... .......... .......... .......... ---------- ----------
D10                 .......... .......... .......... .......... .......... .......... ---------- ----------
FCC2                .......... .......... .......... .......... .......... .......... ---------- ----------
HB3                 .......... .......... .......... .......... .......... .......... ---------- ----------
7G8                 .......... .......... .......... .......... .......... .......... AATAATGATA ATAATGAAAA
P.reichenowi        .......... ..A..T.... ........G. .......... .......... .........A ---------- ----------
```

```
                          1290       1300       1310       1320       1330       1340       1350       1360
                    ....|....| ....|....| ....|....| ....|....| ....|....| ....|....| ....|....| ....|....|
PF13_0192           ---------- ---------- ---------- ---------- ---------- ---------- ---------- AATAATGATA
3D7                 ---------- ---------- ---------- ---------- ---------- ---------- ---------- ..........
RO33                TAATGAAAAT AATGATAATA ATGAAAATAA T--------- ---------- --------GA TAATAATGAA ..........
Palo Alto           ---------- ---------- ---------- ---------- ---------- ---------- ---------- ..........
FCR3                ---------- ---------- ---------- ---------- ---------- ---------- ---------- ..........
Wellcome            ---------- ---------- ---------- ---------- ---------- ---------- ---------- ..........
D6                  ---------- ---------- ---------- ---------- ---------- ---------- ---------- ..........
T996                TAATGATAAT AATGAAAATA ATGAT----- ---------- ---------- ---------- ---------- ..........
T9102               ---------- ---------- ---------- ---------- ---------- ---------- ---------- ..........
K1                  ---------- ---------- ---------- ---------- ---------- ---------- ---------- ..........
Dd2                 ---------- ---------- ---------- ---------- ---------- ---------- ---------- ..........
D10                 ---------- ---------- ---------- ---------- ---------- ---------- ---------- ..........
FCC2                ---------- ---------- ---------- ---------- ---------- ---------- ---------- ..........
HB3                 ---------- ---------- ---------- ---------- ---------- ---------- ---------- ..........
7G8                 TAATGATAAT AATGAAAATA ATGATAATAA TGAAAATAAT GATAATAATG AAAATAATGA TAATAATGAA ..........
P.reichenowi        ---------- ---------- ---------- ---------- ---------- ---------- ---------- ----------

                          1370       1380       1390       1400       1410       1420       1430       1440
                    ....|....| ....|....| ....|....| ....|....| ....|....| ....|....| ....|....| ....|....|
PF13_0192           ATAATGAAAA TAATGATAAT AATGAAAATA ATGATAATAA T--------- ---------A TACCTGTTAA TAATAAAAAA
3D7                 .......... .......... .......... .......... .--------- ---------. .......... ..........
RO33                .......... .......... .......... .......... .--------- ---------. .......... ..........
Palo Alto           .......... .......... ...------- ---------- ---------- ---------. .......... ..........
FCR3                .......... .......... .......... .......... .--------- ---------. .......... ..........
Wellcome            .......... .......... .......... .......... .--------- ---------. .......... ..........
D6                  .......... .......... .......... .......... .GAAAATAAT GATAATAAT. .......... ..........
T996                .......... .......... .......... .......... .--------- ---------. .......... ..........
T9102               .......... .......... .......... .......... .--------- ---------. .......... ..........
K1                  .......... .......... .......... .......... .GAAAATAAT GATAATAAT. .......... ..........
Dd2                 .......... .......... .......... .......... .--------- ---------. .......... ..........
D10                 .......... .......... .......... .......... .--------- ---------. .......... ..........
FCC2                .......... .......... .......... .......... .--------- ---------. .......... ..........
HB3                 .......... .......... .......... .......... .--------- ---------. .......... ..........
7G8                 .......... .......... .......... .......... .--------- ---------. .......... ..........
P.reichenowi        --------.. .........A C..------- ---------- ---------- ---------. .......... ..........

                          1450       1460       1470       1480       1490       1500       1510       1520
                    ....|....| ....|....| ....|....| ....|....| ....|....| ....|....| ....|....| ....|....|
PF13_0192           AATTCTCATA CTCCTCATGT ACCAAAAGAT ACCAAAAATA ATATAGAAAA AAATCATAGT AACTATAATT ACAAACCAAT
3D7                 .......... .......... .......... .......... .......... .......... .......... ..........
RO33                .......... .......... .......... .......... .......... .......... .......... ..........
Palo Alto           .......... .......... .......... .......... .......... .......... .......... ..........
FCR3                .......... .......... .......... .......... .......... .......... .......... ..........
Wellcome            .......... .......... .......... .......... .......... .......... .......... ..........
D6                  .......... .......... .......... .......... .......... .......... .......... ..........
T996                .......... .......... .......... .......... .......... .......... .......... ..........
T9102               .......... .......... .......... .......... .......... .......... .......... ..........
K1                  .......... .......... .......... .......... .......... .......... .......... ..........
Dd2                 .......... .......... .......... .......... .......... .......... .......... ..........
D10                 .......... .......... .......... .......... .......... .......... .......... ..........
FCC2                .......... .......... .......... .......... .......... .......... .......... ..........
HB3                 .......... .......... .......... .......... .......... .......... .......... ..........
7G8                 .......... .......... .......... .......... .......... .......... .......... ..........
P.reichenowi        .......... ..T....... .......... ...C....A. .......... .......... .......... .......T..

                          1530       1540       1550       1560       1570       1580       1590       1600
                    ....|....| ....|....| ....|....| ....|....| ....|....| ....|....| ....|....| ....|....|
PF13_0192           TAATCAAGAA AATTTATCAA TGTATTTTAA TAATAATAAT AAGAAGAAGA AT-------- -------AAA AAGAATAGTC
3D7                 .......... .......... .......... .......... .......... ..-------- -------... ..........
RO33                .......... .......... .......... .......... .......... ..-------- -------... ..........
Palo Alto           .......... .......... .......... .......... .......... ..-------- -------... ..........
FCR3                .......... .......... .......... .......... .......... ..-------- -------... ..........
Wellcome            .......... .......... .......... .......... .......... ..-------- -------... ..........
D6                  .......... .......... .......... .......... .......... ..-------- -------... ..........
T996                .......... .......... .......... .........G .......... ..-------- -------... ..........
T9102               .......... .......... .......... .......... .......... ..-------- -------... ..........
K1                  .......... .......... .......... .......... .......... ..-------- -------... ..........
Dd2                 .......... .......... .......... .......... .......... ..-------- -------... ..........
D10                 .......... .......... .......... .......... .......... ..-------- -------... ..........
FCC2                .......... .......... .......... .......... .......... ..-------- -------... ..........
HB3                 .......... .......... .......... .......... .......... ..-------- -------... ..........
7G8                 .......... .......... .......... .......... .......... ..-------- -------... ..........
P.reichenowi        .......... .......... .......... .......... .......... ..AATCAAAA GAATAATC.. .......A..
```

```
                          1610       1620       1630       1640       1650       1660       1670       1680
                  ....|....| ....|....| ....|....| ....|....| ....|....| ....|....| ....|....| ....|....|
PF13_0192         AAAAAAATAA TCAAAAGAAT AATCAAAAGA ATAATCAAAA GCATAACCAA GATCTTTATA ATATAAAAAA AAATAATGAT
3D7               .......... .......... .......... .......... .......... .......... .......... ..........
RO33              .......... .......... .......... .......... .......... .......... .......... ..........
Palo Alto         .......... .......... .......... .......... .......... .......... .......... ..........
FCR3              .......... .......... .......... .......... .......... .......... .......... ..........
Wellcome          .......... .......... .......... .......... .......... .......... .......... ..........
D6                .......... .......... .......... .......... .......... .......... .......... ..........
T996              .......... .......... .......... .......... .......... .......... .......... ..........
T9102             .......... .......... .......... .......... .......... .......... ..G....... ..........
K1                .......... .......... .......... .......... .......... .......... .......... ..........
Dd2               .......... .......... .......... .......... .......... .......... .......... ..........
D10               .......... .......... .......... .......... .......... .......... ..G....... ..........
FCC2              .......... .......... .......... .......... .......... .......... .......... ..........
HB3               .......... .......... .......... .......... .......... .......... .......... ..........
7G8               .......... .......... .......... .......... .......... .......... .......... ..........
P.reichenowi      ....G..... .......T.. .......... .......... .A........ .......... .......... ...C......

                          1690       1700       1710       1720       1730       1740       1750       1760
                  ....|....| ....|....| ....|....| ....|....| ....|....| ....|....| ....|....| ....|....|
PF13_0192         CTACCTCAAT ATGAAGAATC TAATAAAACT TATAAAAAAA TTTTTTCAAA TGAACATGAA CCACGTCCGA TGATAACAAC
3D7               .......... .......... .......... .......... .......... .......... .......... ..........
RO33              .......... .......... .......... .......... .......... .......... ......T... ..........
Palo Alto         .......... .......... .......... .......... .......... .......... .......... ..........
FCR3              .......... .......... .......... .......... .......... .......... ......T... ..........
Wellcome          .......... .......... .......... .......... .......... .......... ......T... ..........
D6                .......... .......... .......... .......... .......... .......... ......T... ..........
T996              .......... .......... .......... .......... .......... .......... ......T... ..........
T9102             .......... .......... .......... .......... A......... .......... ......T... ..........
K1                .......... .......... .......... .......... .......... .......... ......T... ..........
Dd2               .......... .......... .......... .......... A......... .......... ......T... ..........
D10               .......... .......... .......... .......... A......... .......... ......T... ..........
FCC2              .......... .......... .......... .......... .......... .......... ......T... ..........
HB3               .......... .......... .......... .......... .......... .......... ......T... ..........
7G8               .......... .......... .......... .......... .......... .......... ......T... ..........
P.reichenowi      .......... .......... ........T. ........C. AA........ .......... ......T... ..........

                          1770       1780       1790       1800       1810       1820       1830       1840
                  ....|....| ....|....| ....|....| ....|....| ....|....| ....|....| ....|....| ....|....|
PF13_0192         ATCCAGATAT GAAAATAAAA TATCAAATGA TAATAACAAC AATAACTATA ATTTGAATGA ACAAGAAAGA AAACTTATTA
3D7               .......... .......... .......... .......... .......... .......... .......... ..........
RO33              .......... .......... .......... .......... .......... .......... .......... ..........
Palo Alto         .......... .......... .......... .......... .......... .......... .......... ..........
FCR3              .......... .......... .......... .......... .......... .......... .......... ..........
Wellcome          .......... .......... .......... .......... .......... .......... .......... ..........
D6                .......... .......... .......... .......... .......... .......... .......... ..........
T996              .......... .......... .......... .......... .......... .......... .......... ..........
T9102             .......... .......... .......... .......... .......... .......... .......... ..........
K1                .......... .......... .......... .......... .......... .......... .......... ..........
Dd2               .......... .......... .......... .......... .......... .......... .......... ..........
D10               .......... .......... .......... .......... .......... .......... .......... ..........
FCC2              .......... .......... .......... .......... .......... .......... .......... ..........
HB3               .......... .......... .......... .......... .......... .......... .......... ..........
7G8               .......... .......... .......... .......... .......... .......... .......... ..........
P.reichenowi      .......... .......... A......C.. ....T..... .......... ....T..... .......... ......C...

                          1850       1860       1870       1880       1890       1900       1910       1920
                  ....|....| ....|....| ....|....| ....|....| ....|....| ....|....| ....|....| ....|....|
PF13_0192         AAAATATGAT AGATATATTT ATTTATACAT ACAAGTTAAA TTATACAAAC AGTAAATCTA TATCTAAATT ATTTAAAAAT
3D7               .......... .......... .......... .......... .......... .......... .......... ..........
RO33              .......... .......... .......... .......... .......... .......... .......... ..........
Palo Alto         .......... .......... .......... .......... .......... .......... .......... ..........
FCR3              .......... .......... .......... .......... .......... .......... .......... ..........
Wellcome          .......... .......... .......... .......... .......... .......... .......... ..........
D6                .......... .......... .......... .......... .......... .......... .......... ..........
T996              .......... .......... .......... .......... .......... .......... .......... ..........
T9102             .......... .......... .......... .......... .......... .......... .......... ..........
K1                .......... .......... .......... .......... .......... .......... .......... ..........
Dd2               .......... .......... .......... .......... .......... .......... .......... ..........
D10               .......... .......... .......... .......... .......... .......... .......... ..........
FCC2              .......... .......... .......... .......... .......... .......... .......... ..........
HB3               .......... .......... .......... .......... .......... .......... .......... ..........
7G8               .......... .......... .......... .......... .......... .......... .......... ..........
P.reichenowi      .......... .......... .......... .......... .......... .......... .......... ..........
```

```
                              1930       1940       1950       1960       1970       1980       1990       2000
                      ....|....| ....|....| ....|....| ....|....| ....|....| ....|....| ....|....| ....|....|
PF13_0192             AATTTACTTA AAAAAAACTT CCGTACTTAT TTCACAAATT ATATATATAC ACTTTTTAAT TATGGAAAGA CATATAATTT
3D7                   .......... .......... .......... .......... .......... .......... .......... ..........
RO33                  .......... .......... .......... .......... .......... .......... .......... ..........
Palo Alto             .......... .......... .......... .......... .......... .......... .......... ..........
FCR3                  .......... .......... .......... .......... .......... .......... .......... ..........
Wellcome              .......... .......... .......... .......... .......... .......... .......... ..........
D6                    .......... .......... .......... .......... .......... .......... .......... ..........
T996                  .......... .......... .......... .......... .......... .......... .......... ..........
T9102                 .......... .......... .......... .......... .......... .......... .......... ..........
K1                    .......... .......... .......... .......... .......... .......... .......... ..........
Dd2                   .......... .......... .......... .......... .......... .......... .......... ..........
D10                   .......... .......... .......... .......... .......... .......... .......... ..........
FCC2                  .......... .......... .......... .......... .......... .......... .......... ..........
HB3                   .......... .......... .......... .......... .......... .......... .......... ..........
7G8                   .......... .......... .......... .......... .......... .......... .......... ..........
P.reichenowi          .......... .......... .......... .......... .......... .......... .......... ..........

                              2010       2020       2030       2040       2050       2060       2070       2080
                      ....|....| ....|....| ....|....| ....|....| ....|....| ....|....| ....|....| ....|....|
PF13_0192             TCTAACACCT TATAATAAAG ATAATGATCA TATGTATAGA CAATTATTTG ATGAAGCTGT ACAAATGATG GATCTACTTA
3D7                   .......... .......... .......... .......... .......... .......... .......
RO33                  .......... .......... .......... .......... .......... .......... .......
Palo Alto             .......... .......... .......... .......... .......... .......... .......
FCR3                  .......... .......... .......... .......... .......... .......... .......
Wellcome              .......... .......... .......... .......... .......... .......... .......
D6                    .......... .......... .......... .......... .......... .......... .......
T996                  .......... .......... .......... .......... .......... .......... .......
T9102                 .......... .......... .......... .......... .......... .......... .......
K1                    .......... .......... .......... .......... .......... .......... .......
Dd2                   .......... .......... .......... .......... .......... .......... .......
D10                   .......... .......... .......... .......... .......... .......... .......
FCC2                  .......... .......... .......... .......... .......... .......... .......
HB3                   .......... .......... .......... .......... .......... .......... .......
7G8                   .......... .......... .......... .......... .......... .......... .......
P.reichenowi          .......... .......... .......... .......... .......... .......... .......

                              2090       2100       2110       2120
                      ....|....| ....|....| ....|....| ....|....| .
PF13_0192             TTAATAAAAT GGACTTAGCT TTAAATCCCA CGAAATTATG A
3D7
RO33
Palo Alto
FCR3
Wellcome
D6
T996
T9102
K1
Dd2
D10
FCC2
HB3
7G8
P.reichenowi
```

PF13_0194

```
                               10         20         30         40         50         60         70         80
                      ....|....| ....|....| ....|....| ....|....| ....|....| ....|....| ....|....| ....|....|
PF13_0194             ATGAAAAGAA CAAGACATAT TACAGTATTT ATTTTTTCTG CATTTTTATT CTTACTAAAT TATGCGACAA CTAGTCAATC
3D7                   .......... .......... .......... .......... .......... .......... .......... ..........
RO33                  .......... .......... .......... .......... .......... .......... .......... ..........
Palo Alto             .......... .......... .......... .......... .......... .......... .......... ..........
FCR3                  .......... .......... .......... .......... .......... .......... .......... ..........
Wellcome                                 .. .......... .......... .......... .......... .......... ..........
D6                    .......... .......... .......... .......... .......... .......... .......... ..........
T996                  .......... .......... .......... .......... .......... .......... .......... ..........
T9102                 .......... .......... .......... .......... .......... .......... .......... ..........
K1                    .......... .......... .......... .......... .......... .......... .......... ..........
D10                   .......... .......... .......... .......... .......... .......... .......... ..........
FCC2                  .......... .......... .......... .......... .......... .......... .......... ..........
HB3                   .......... .......... .......... .......... .......... .......... .......... ..........
7G8                   .......... .......... .......... .......... .......... .......... .......... ..........
P.reichenowi          ......G... .......... .......... .......... .......... .......... .......... ..........
```

```
                              90        100        110        120        130        140        150        160
                      ....|....| ....|....| ....|....| ....|....| ....|....| ....|....| ....|....| ....|....|
PF13_0194             GGTTAATTAT GATAATGAAA AAAAACAAAT AAATAATTTA AAAGAAAGAC TAGAAAATAT AAGTAGTGAT ATTCCAGAAG
3D7                   .......... .......... .......... .......... .......... .......... .......... ..........
RO33                  .......... .......... .......... .......... .......... .......... .......... ..........
Palo Alto             .......... .......... .......... .......... .......... .......... .......... ..........
FCR3                  .......... .......... .......... .......... .......... .......... .......... ..........
Wellcome              .......... .......... .......... .......... .......... .......... .......... ..........
D6                    .......... .......... .......... .......... .......... .......... .......... ..........
T996                  .......... .......... .......... .......... .......... .......... .......... ..........
T9102                 .......... .......... .......... .......... .......... .......... .......... ..........
K1                    .......... .......... .......... .......... .......... .......... .......... ..........
D10                   .......... .......... .......... .......... .......... .......... .......... ..........
FCC2                  .......... .......... .......... .......... .......... .......... .......... ..........
HB3                   .......... .......... .......... .......... .......... .......... .......... ..........
7G8                   .......... .......... .......... .......... .......... .......... .......... ..........
P.reichenowi          .......... .......... .......... .......... .......... .......... .......... ..........

                             170        180        190        200        210        220        230        240
                      ....|....| ....|....| ....|....| ....|....| ....|....| ....|....| ....|....| ....|....|
PF13_0194             ATCTTAAAAA TCACTTAAAA GATAATTTAA ATTTTTTTAA TAATTCCCTT GAAGAAGCAC AAAAGAGTTT ATTAGACCAA
3D7                   .......... .......... .......... .......... .......... .......... .......... ..........
RO33                  .......... .......... .......... .......... .......... .......... .......... ..........
Palo Alto             .......... .......... .......... .......... .......... .......... .......... ..........
FCR3                  .......... .......... .......... .......... .......... .......... .......... ..........
Wellcome              .......... .......... .......... .......... .......... .......... .......... ..........
D6                    .......... .......... .......... .......... .......... .......... .......... ..........
T996                  .......... .......... .......... .......... .......... .......... .......... ..........
T9102                 .......... .......... .......... .......... .......... .......... .......... ..........
K1                    .......... .......... .......... .......... .......... .......... .......... ..........
D10                   .......... .......... .......... .......... .......... .......... .......... ..........
FCC2                  .......... .......... .......... .......... .......... .......... .......... ..........
HB3                   .......... .......... .......... .......... .......... .......... .......... ..........
7G8                   .......... .......... .......... .......... .......... .......... .......... ..........
P.reichenowi          .......... .......... .......... .......... .......... .......... .......... ..........

                             250        260        270        280        290        300        310        320
                      ....|....| ....|....| ....|....| ....|....| ....|....| ....|....| ....|....| ....|....|
PF13_0194             CCAAATATTT CTACATCAAC ACAAGGTAGT GAAAATAATA CTTCAGGTGT TAGTACTAAT AATAATTTAA CAAATCCAAA
3D7                   .......... .......... .......... .......... .......... .......... .......... ..........
RO33                  .......... .......... .......... .......... .......... .......... .......... ..........
Palo Alto             .......... .......... .......... .......... .......... .......... .......... ..........
FCR3                  .......... .......... .......... .......... .......... .......... .......... ..........
Wellcome              .......... .......... .......... .......... .......... .......... .......... ..........
D6                    .......... .......... .......... .......... .......... .......... .......... ..........
T996                  .......... .......... .......... .......... .......... .......... .......... ..........
T9102                 .......... .......... .......... .......... .......... .......... .......... ..........
K1                    .......... .......... .......... .......... .......... .......... .......... ..........
D10                   .......... .......... .......... .......... .......... .......... .......... ..........
FCC2                  .......... .......... .......... .......... .......... .......... .......... ..........
HB3                   .......... .......... .......... .......... .......... .......... .......... ..........
7G8                   .......... .......... .......... .......... .......... .......... .......... ..........
P.reichenowi          .......... .......... .......... ...G...... .......... .......... .......... ..........

                             330        340        350        360        370        380        390        400
                      ....|....| ....|....| ....|....| ....|....| ....|....| ....|....| ....|....| ....|....|
PF13_0194             TGATCCAACA CAATTAAATG AACAAGAATG TCAATCAATC AAAAAATTTA TAAAAGAACT TTTGAGTGCT GCTACAAATG
3D7                   .......... .......... .......... .......... .......... .......... .......... ..........
RO33                  .......... .......... .......... .......... .......... .......... .......... ..........
Palo Alto             .......... .......... .......... .......... .......... .......... .......... ..........
FCR3                  .......... .......... .......... .......... .......... .......... .......... ..........
Wellcome              .......... .......... .......... .......... .......... .......... .......... ..........
D6                    .......... .......... .......... .......... .......... .......... .......... ..........
T996                  .......... .......... .......... .......... .......... .......... .......... ..........
T9102                 .......... .......... .......... .......... .......... .......... .......... ..........
K1                    .......... .......... .......... .......... .......... .......... .......... ..........
D10                   .......... .......... .......... .......... .......... .......... .......... ..........
FCC2                  .......... .......... .......... .......... .......... .......... .......... ..........
HB3                   .......... .......... .......... .......... .......... .......... .......... ..........
7G8                   .......... .......... .......... .......... .......... .......... .......... ..........
P.reichenowi          .......... .......... .......... .......... .......... .......... .......... ..........
```

```
                                 410        420        430        440        450        460        470        480
                        ....|....| ....|....| ....|....| ....|....| ....|....| ....|....| ....|....| ....|....|
PF13_0194               ATGAACATGT TAATACTCCT TTCTCTACAA CTTTTAAAAA TGCATTAGAA AAAGATGAAT TCCGTGATAG TATCACGAAA
3D7                     .......... .......... .......... .......... .......... .......... .......... ..........
RO33                    .......... .......... .......... .......... .......... .......... .......... ..........
Palo Alto               .......... .......... .......... .......... .......... .......... .......... ..........
FCR3                    .......... .......... .......... .......... .......... .......... .......... ..........
Wellcome                .......... .......... .......... .......... .......... .......... .......... ..........
D6                      .......... .......... .......... .......... .......... .......... .......... ..........
T996                    .......... .......... .......... .......... .......... .......... .......... ..........
T9102                   .......... .......... .......... .......... .......... .......... .......... ..........
K1                      .......... .......... .......... .......... .......... .......... .......... ..........
D10                     .......... .......... .......... .......... .......... .......... .......... ..........
FCC2                    .......... .......... .......... .......... .......... .......... .......... ..........
HB3                     .......... .......... .......... .......... .......... .......... .......... ..........
7G8                     .......... .......... .......... .......... .......... .......... .......... ..........
P.reichenowi            .......... .......... .......... .......... .......... .......... .......... CC........

                                 490        500        510        520        530        540        550        560
                        ....|....| ....|....| ....|....| ....|....| ....|....| ....|....| ....|....| ....|....|
PF13_0194               TTAACAGAAG CACTATGTGG ACATACAAAA GATAATCATA ATAATTTAAG AGGAACTAGC CAAAATGATT CAGACTATTT
3D7                     .......... .......... .......... .......... .......... .......... .......... ..........
RO33                    .......... .......... .......... .......... .......... .......... .......... ..........
Palo Alto               .......... .......... .......... .......... .......... .......... .......... ..........
FCR3                    .......... .......... .......... .......... .......... .......... .......... ..........
Wellcome                .......... .......... .......... .......... .......... .......... .......... ..........
D6                      .......... .......... .......... .......... .......... .......... .......... ..........
T996                    .......... .......... .......... .......... .......... .......... .......... ..........
T9102                   .......... .......... .......... .......... .......... .......... .......... ..........
K1                      .......... .......... .......... .......... .......... .......... .......... ..........
D10                     .......... .......... .......... .......... .......... .......... .......... ..........
FCC2                    .......... .......... .......... .......... .......... .......... .......... ..........
HB3                     .......... .......... .......... .......... .......... .......... .......... ..........
7G8                     .......... .......... .......... .......... .......... .......... .......... ..........
P.reichenowi            .......... .......... .......... .......... .......... .......... .......... ..........

                                 570        580        590        600
                        ....|....| ....|....| ....|....| ....|....| ....|....
PF13_0194               ACATTCAGTA TATGATAAGG CCCTTTCTCA TTTAGATACC TTACAATAA
3D7                     .......... .......... .....
RO33                    .......... .......... .....
Palo Alto               .......... .......... .....
FCR3                    .......... .......... .....
Wellcome                .......... .......... .....
D6                      .......... .......... .....
T996                    .......... .......... .....
T9102                   .......... .......... .....
K1                      .......... .......... .....
D10                     .......... .......... .....
FCC2                    .......... .......... .....
HB3                     .......... .......... .....
7G8                     .......... .......... .....
P.reichenowi            .......... .......... .......... .......... .........
```

MSP9 – ABRA_FCC1_HN

```
                                 10         20         30         40         50         60         70         80
                         ....|....| ....|....| ....|....| ....|....| ....|....| ....|....| ....|....| ....|....|
PFL1385c                 ATGATGAACA TGAAAATTGT TTTATTCAGT TTATTGCTCT TTGTCATAAG ATGGAATATT ATTAGTTGTA ATAAAAACGA
3D7
RO33
Palo Alto
FCR3
Wellcome
D6
T996
T9102
K1
Dd2
D10
FCC2
HB3
7G8
P.reichenowi

                                 90        100        110        120        130        140        150        160
                         ....|....| ....|....| ....|....| ....|....| ....|....| ....|....| ....|....| ....|....|
PFL1385c                 CAAGAACCAA GGTGTTGATA TGAATGTTTT GAATAATTAT GAAAATTTAT TTAAATTTGT TAAATGTGAA TATTGTAATG
3D7                                 .......... .......... .......... .......... .......... .......... ..........
RO33                                .......... .......... .......... .......... .......... .......... ..........
Palo Alto                           .......... .......... .......... .......... .......... .......... ..........
FCR3                                .......... .......... .......... .......... .....G.... .......... ..........
Wellcome                            .......... .......... .......... .......... .....G.... .......... ..........
D6                                  .......... .......... .......... .......... .......... .......... ..........
T996                                .......... .......... .......... .......... .......... .......... ..........
T9102                               .......... .......... .......... .......... .....G.... .......... ..........
K1                                  .......... .......... .......... .......... .....G.... .......... ..........
Dd2                                 .......... .......... .......... .......... .....G.... .......... ..........
D10                                 .......... .......... .......... .......... .......... .......... ..........
FCC2                                .......... .......... .......... .......... .....G.... .......... ..........
HB3                                 .......... .......... .......... .......... .....G.... .......... ..........
7G8                                 .......... .......... .......... .......... .......... .......... ..........
P.reichenowi                        .......... .......... .......... .......... .....A.A.. .......... ......C...

                                170        180        190        200        210        220        230        240
                         ....|....| ....|....| ....|....| ....|....| ....|....| ....|....| ....|....| ....|....|
PFL1385c                 AACATACTTA TGTTAAAGGT AAGAAAGCTC CTTCAGATCC TCAATGTGCT GATATAAAAG AAGAATGCAA AGAATTACTT
3D7                      .......... .......... .......... .......... .......... .......... .......... ..........
RO33                     .......... .......... .......... .......... .......... .......... .......... ..........
Palo Alto                .......... .......... .......... .......... .......... .......... .......... ..........
FCR3                     .......... .......... .......... .......... .......... .......... .......... ..........
Wellcome                 .......... .......... .......... .......... .......... .......... .......... ..........
D6                       .......... .......... .......... .......... .......... .......... .......... ..........
T996                     .......... .......... .......... .......... .......... .......... .......... ..........
T9102                    .......... .......... .......... .......... .......... .......... .......... ..........
K1                       .......... .......... .......... .......... .......... .......... .......... ..........
Dd2                      .......... .......... .......... .......... .......... .......... .......... ..........
D10                      .......... .......... .......... .......... .......... .......... .......... ..........
FCC2                     .......... .......... .......... .......... .......... .......... .......... ..........
HB3                      .......... .......... .......... .......... .......... .......... .......... ..........
7G8                      .......... .......... .......... .......... .......... .......... .......... ..........
P.reichenowi             .......... .......... .....GC..T AC........ .......... .C........ .......... ..........

                                250        260        270        280        290        300        310        320
                         ....|....| ....|....| ....|....| ....|....| ....|....| ....|....| ....|....| ....|....|
PFL1385c                 AAGGAAAAAC AATACACAGA TTCAGTTACA TATTTAATGG ATGGTTTTAA ATCAGCAAAT AATTCAGCAA ATAAT-----
3D7                      .......... .......... .......... .......... .......... .......... .......... .....-----
RO33                     .......... .......... .......... .......... .......... .......... .......... .....-----
Palo Alto                .......... .......... .......... .......... .......... .......... .......... .....-----
FCR3                     .......... .......... .......... .......... .......... .......... .......... .....-----
Wellcome                 .......... .......... .......... .......... .......... .......... .......... .....-----
D6                       .......... .......... .......... .......... .......... .......... .......... .....TCAGC
T996                     .......... .......... .......... .......... .......... .......... .......... .....-----
T9102                    .......... .......... .......... .......... .......... .......... .......... .....-----
K1                       .......... .......... .......... .......... .......... .......... .......... .....-----
Dd2                      .......... .......... .......... .......... .......... .......... .......... .....-----
D10                      .......... .......... .......... .......... .......... .......... .......... .....-----
FCC2                     .......... .......... .......... .......... .......... .......... .......... .....-----
HB3                      .......... .......... .......... .......... .......... .......... .......... .....-----
7G8                      .......... .......... .......... .......... .......... .......... .......... .....-----
P.reichenowi             ...A.....G .......... .......... ...A...... .......... .......... .G..T..A.. .....-----
```

```
                        330        340        350        360        370        380        390        400
               ....|....| ....|....| ....|....| ....|....| ....|....| ....|....| ....|....| ....|....|
PFL1385c       -------GGT AAAAAAAATA ACGCTGAAGA AATGAAAAAT TTAGTAAATT TCTTACAATC TCATAAGAAA TTAATTAAAG
3D7            -------... .......... .......... .......... .......... .......... .......... ..........
RO33           -------... .......... .......... .......... .......... .......... .......... ..........
Palo Alto      -------... .......... .......... .......... .......... .......... .......... ..........
FCR3           -------... .......... .......... .......... .......... .......... .......... ..........
Wellcome       -------... .......... .......... .......... .......... .......... .......... ..........
D6             AAATAAT... .......... .......... .......... .......... .......... .......... ..........
T996           -------... .......... .......... .......... .......... .......... .......... ..........
T9102          -------... .......... .......... .......... .......... .......... .......... ..........
K1             -------... .......... .......... .......... .......... .......... .......... ..........
Dd2            -------... .......... .......... .......... .......... .......... .......... ..........
D10            -------... .......... .......... .......... .......... .......... .......... ..........
FCC2           -------... .......... .......... .......... .......... .......... .......... ..........
HB3            -------... .......... .......... .......... .......... .......... .......... ..........
7G8            -------... .......... .......... .......... .......... .......... .......... ..........
P.reichenowi   -------.A. .......... .......... .......... .......... .......... .......... ..........

                        410        420        430        440        450        460        470        480
               ....|....| ....|....| ....|....| ....|....| ....|....| ....|....| ....|....| ....|....|
PFL1385c       CATTAAAAAA GAATATTGAA AGTATACAAA ATAAGAAACA CTTAATTTAT AAAAACAAAT CATATAATCC ATTATTACTT
3D7            .......... .......... .......... .......... .......... .......... .......... ..........
RO33           .......... .......... .......... .......... .......... .......... .......... ..........
Palo Alto      .......... .......... .......... .......... .......... .......... .......... ..........
FCR3           .......... .......... .......... .......... .......... .......... .......... ..........
Wellcome       .......... .......... .......... .......... .......... .......... .......... ..........
D6             .......... .......... .......... .......... .......... .......... .......... ..........
T996           .......... .......... .......... .......... .......... .......... .......... ..........
T9102          .......... .......... .......... .......... .......... .......... .......... ..........
K1             .......... .......... .......... .......... .......... .......... .......... ..........
Dd2            .......... .......... .......... .......... .......... .......... .......... ..........
D10            .......... .......... .......... .......... .......... .......... .......... ..........
FCC2           .......... .......... .......... .......... .......... .......... .......... ..........
HB3            .......... .......... .......... .......... .......... .......... .......... ..........
7G8            .......... .......... .......... .......... .......... .......... .......... ..........
P.reichenowi   G......... .......... .......... ........T. ....G..... .....T.... .......... ..........

                        490        500        510        520        530        540        550        560
               ....|....| ....|....| ....|....| ....|....| ....|....| ....|....| ....|....| ....|....|
PFL1385c       TCTTGTGTTA AAAAAATGAA TATGTTAAAA GAAAATGTTG ACTATATTCA AAAAAATCAA AACTTATTTA AAGAATTAAT
3D7            .......... .......... .......... .......... .......... .......... .......... ..........
RO33           .......... .......... .......... .......... .......... .......... .......... ..........
Palo Alto      .......... .......... .......... .......... .......... .......... .......... ..........
FCR3           .......... .......... .......... .......... .......... .......... .......... ..........
Wellcome       .......... .......... .......... .......... .......... .......... .......... ..........
D6             .......... .......... .......... .......... .......... .......... .......... ..........
T996           .......... .......... .......... .......... .......... .......... .......... ..........
T9102          .......... .......... .......... .......... .......... .......... .......... ..........
K1             .......... .......... .......... .......... .......... .......... .......... ..........
Dd2            .......... .......... .......... .......... .......... .......... .......... ..........
D10            .......... .......... .......... .......... .......... .......... .......... ..........
FCC2           .......... .......... .......... .......... .......... .......... .......... ..........
HB3            .......... .......... .......... .......... .......... .......... .......... ..........
7G8            .......... .......... .......... .......... .......... .......... .......... ..........
P.reichenowi   A...T..... .......... .......... .......... .......... ...G...... .......... ..........

                        570        580        590        600        610        620        630        640
               ....|....| ....|....| ....|....| ....|....| ....|....| ....|....| ....|....| ....|....|
PFL1385c       GAATCAAAAA GCTACCTACT CTTTTGTTAA TACCAAAAAA AAAATTATTT CTTTAAAATC ACAAGGTCAT AAAAAAGAAA
3D7            .......... .......... .......... .......... .......... .......... .......... ..........
RO33           .......... .......... .......... .......... .......... .......... .......... ..........
Palo Alto      .......... .......... .......... .......... .......... .......... .......... ..........
FCR3           .......... .......... .......... .......... .......... .......... .......... ..........
Wellcome       .......... .......... .......... .......... .......... .......... .......... ..........
D6             .......... .......... .......... .......... .......... .......... .......... ..........
T996           .......... .......... .......... .......... .......... .......... .......... ..........
T9102          .......... .......... .......... .......... .......... .......... .......... ..........
K1             .......... .......... .......... .......... .......... .......... .......... ..........
Dd2            .......... .......... .......... .......... .......... .......... .......... ..........
D10            .......... .......... .......... .......... .......... .......... .......... ..........
FCC2           .......... .......... .......... .......... .......... .......... .......... ..........
HB3            .......... .......... .......... .......... .......... .......... .......... ..........
7G8            .......... .......... .......... .......... .......... .......... .......... ..........
P.reichenowi   .G........ T...A..CT. ........G. .......... .....C.... .......... .......... ..........
```

```
                         650        660        670        680        690        700        710        720
                 ....|....| ....|....| ....|....| ....|....| ....|....| ....|....| ....|....| ....|....|
PFL1385c         CCTCACAAAA TCAAAATGAA AATAACGACA ATCAAAAATA TCAAGAAGTT AATGATGAAG ATGATGTAAA TGATGAAGAA
3D7              .......... .......... .......... .......... .......... .......... .......... ..........
RO33             .......... .......... .......... .......... .......... .......... .......... ..........
Palo Alto        .......... .......... .......... .......... .......... .......... .......... ..........
FCR3             .......... .......... .......... .......... .......... .......... .......... ..........
Wellcome         .......... .......... .......... .......... .......... .......... .......... ..........
D6               .......... .......... .......... .......... .......... .......... .......... ..........
T996             .......... .......... .......... .......... .......... .......... .......... ..........
T9102            .......... .......... .......... .......... .......... .......... .......... ..........
K1               .......... .......... .......... .......... .......... .......... .......... ..........
Dd2              .......... .......... .......... .......... .......... .......... .......... ..........
D10              .......... .......... .......... .......... .......... .......... .......... ..........
FCC2             .......... .......... .......... .......... .......... .......... .......... ..........
HB3              .......... .......... .......... .......... .......... .......... .......... ..........
7G8              .......... .......... .......... .......... .......... .......... .......... ..........
P.reichenowi     .......... .......... .....G.... .......... .......... .......... .A..A..T.. ..........

                         730        740        750        760        770        780        790        800
                 ....|....| ....|....| ....|....| ....|....| ....|....| ....|....| ....|....| ....|....|
PFL1385c         GATACAAACG ATGACGAAGA TACTAACGAT GAAGAAGATA CAAACGATGA CGAAGATACA AATGATGACG AAGATACTAA
3D7              .......... .......... .......... .......... .......... .......... .......... ..........
RO33             .......... .......... .......... .......... .......... .......... .......... ..........
Palo Alto        .......... .......... .......... .......... .......... .......... .......... ..........
FCR3             .......... .......... .......... .......... .......... .......... .......... ..........
Wellcome         .......... .......... .......... .......... .......... .......... .......... ..........
D6               .......... .......... .......... .......... .......... .......... .......... ..........
T996             .......... .......... .......... .......... .......... .......... .......... ..........
T9102            .......... .......... .......... .......... .......... .......... .......... ..........
K1               .......... .......... .......... .......... .......... .......... .......... ..........
Dd2              .......... .......... .......... .......... .......... .......... .......... ..........
D10              .......... .......... .......... .......... .......... .......... .......... ..........
FCC2             .......... .......... .......... .......... .......... .......... .......... ..........
HB3              .......... .......... .......... .......... .......... .......... .......... ..........
7G8              .......... .......... .......... .......... .......... .......... .......... ..........
P.reichenowi     ..AGTT..T. ....A..... AGT...T... .........G TT..T..... A......GTT ........A. .....GT...

                         810        820        830        840        850        860        870        880
                 ....|....| ....|....| ....|....| ....|....| ....|....| ....|....| ....|....| ....|....|
PFL1385c         CGATGAAGAA GATACTAACG ACGAAGAAGA TCATGAAAAT AATAATGCTA CAGCATACGA ATTAGGTATC GTCCCAGTTA
3D7              .......... .......... .......... .......... .......... .......... .......... ..........
RO33             .......... .......... .......... .......... .......... .......... .......... ..........
Palo Alto        .......... .......... .......... .......... .......... .......... .......... ..........
FCR3             .......... .......... .......... .......... .......... .......... .......... ..........
Wellcome         .......... .......... .......... .......... .......... .......... .......... ..........
D6               .......... .......... .......... .......... .......... .......... .......... ..........
T996             .......... .......... .......... .......... .......... .......... .......... ..........
T9102            .......... .......... .......... .......... .......... .......... .......... ..........
K1               .......... .......... .......... .......... .......... .......... .......... ..........
Dd2              .......... .......... .......... .......... .......... .......... .......... ..........
D10              .......... .......... .......... .......... .......... .......... .......... ..........
FCC2             .......... .......... .......... .......... .......... .......... .......... ..........
HB3              .......... .......... .......... .......... .......... .......... .......... ..........
7G8              .......... .......... .......... .......... .......... .......... .......... ..........
P.reichenowi     T......... ...TTA..T. .T........ .......... .......G.. ..A....... ........GT ..........

                         890        900        910        920        930        940        950        960
                 ....|....| ....|....| ....|....| ....|....| ....|....| ....|....| ....|....| ....|....|
PFL1385c         ACGATGTGTT AAATGTTAAT ATGAAAAATA TGATAACAGG AAATAATTTT ATGGATGTTG TTAAAAATAC ATTAGCTCAA
3D7              .......... .......... .......... .......... .......... .......... .......... ..........
RO33             .......... .......... .......... .......... .......... .......... .......... ..........
Palo Alto        .......... .......... .......... .......... .......... .......... .......... ..........
FCR3             .......... .......... .......... .......... .......... .......... ......G... ..........
Wellcome         .......... .......... .......... .......... .......... .......... ......G... ..........
D6               .......... .......... .......... .......... .......... .......... .......... ..........
T996             .......... .......... .......... .......... .......... .......... .......... ..........
T9102            .......... .......... .......... .......... .......... .......... ......G... ..........
K1               .......... .......... .......... .......... .......... .......... ......G... ..........
Dd2              .......... .......... .......... .......... .......... .......... ......G... ..........
D10              .......... .......... .......... .......... .......... .......... ......G... ..........
FCC2             .......... .......... .......... .......... .......... .......... ......G... ..........
HB3              .......... .......... .......... .......... .......... .......... .......... ..........
7G8              .......... .......... .......... .......... .......... .......... .......... ..........
P.reichenowi     .T.....A.. .....G.... .......... .......... .......... .......... ......G... T.........
```

```
                        970        980        990       1000       1010       1020       1030       1040
               ....|....| ....|....| ....|....| ....|....| ....|....| ....|....| ....|....| ....|....|
PFL1385c       TCAGGTGGAT TAGGAAGTAA TGATTTAATA AATTTCTTAA ATCAAGGTAA AGAAATAGGA GAAAATTTAT TAAACATAAC
3D7            .......... .......... .......... .......... .......... .......... .......... ..........
RO33           .......... .......... .......... .......... .......... .......... .......... ..........
Palo Alto      .......... .......... .......... .......... .......... .......... .......... ..........
FCR3           .......... .......... .......... .......... .......... .......... .......... ..........
Wellcome       .......... .......... .......... .......... .......... .......... .......... ..........
D6             .......... .......... .......... .......... .......... .......... .......... ..........
T996           .......... .......... .......... .......... .......... .......... .......... ..........
T9102          .......... .......... .......... .......... .......... .......... .......... ..........
K1             .......... .......... .......... .......... .......... .......... .......... ..........
Dd2            .......... .......... .......... .......... .......... .......... .......... ..........
D10            .......... .......... .......... .......... .......... .......... .......... ..........
FCC2           .......... .......... .......... .......... .......... .......... .......... ..........
HB3            .......... .......... .......... .......... .......... .......... .......... ..........
7G8            .......... .......... .......... .......... .......... .......... .......... ..........
P.reichenowi   A......... .......... ........GT .......... .......... .......... .......... ..........

                       1050       1060       1070       1080       1090       1100       1110       1120
               ....|....| ....|....| ....|....| ....|....| ....|....| ....|....| ....|....| ....|....|
PFL1385c       AAAGATGAAC TTGGGAGATA AGAATAATCT TGAAAGTTTT CCTTTAGATG AATTAAATAT GTTAAAAGAT AATTTAATAA
3D7            .......... .......... .......... .......... .......... .......... .......... ..........
RO33           .......... .......... .......... .......... .......... .......... .......... ..........
Palo Alto      .......... .......... .......... .......... .......... .......... .......... ..........
FCR3           .......... .......... .......... .......... .........C .......... .......... ..........
Wellcome       .......... .......... .......... .......... .........C .......... .......... ..........
D6             .......... .......... .......... .......... .......... .......... .......... ..........
T996           .......... .......... .......... .......... .......... .......... .......... ..........
T9102          .......... .......... .......... .......... .........C .......... .......... ..........
K1             .......... .......... .......... .......... .........C .......... .......... ..........
Dd2            .......... .......... .......... .......... .........C .......... .......... ..........
D10            .......... .......... .......... .......... .........C .......... .......... ..........
FCC2           .......... .......... .......... .......... .......... .......... .......... ..........
HB3            .......... .......... .......... .......... .......... .......... .......... ..........
7G8            .......... .......... .......... .......... .......... .......... .......... ..........
P.reichenowi   .......... .......... T......... .........C .........C .......... C......... .........T

                       1130       1140       1150       1160       1170       1180       1190       1200
               ....|....| ....|....| ....|....| ....|....| ....|....| ....|....| ....|....| ....|....|
PFL1385c       ACTATGAATT CATATTAGAT AATTTGAAAA CAAGTGTTTT AAATAAATTA AAAGATTTAT TATTAAGATT ATTATACAAA
3D7            .......... .......... .......... .......... .......... .......... .......... ..........
RO33           .......... .......... .......... .......... .......... .......... .......... ..........
Palo Alto      .......... .......... .......... .......... .......... .......... .......... ..........
FCR3           .......... .......A.. .......... .......... .......... .......... .......... ..........
Wellcome       .......... .......A.. .......... .......... .......... .......... .......... ..........
D6             .......... .......... .......... .......... .......... .......... .......... ..........
T996           .......... .......... .......... .......... .......... .......... .......... ..........
T9102          .......... .......A.. .......... .......... .......... .......... .......... ..........
K1             .......... .......A.. .......... .......... .......... .......... .......... ..........
Dd2            .......... .......A.. .......... .......... .......... .......... .......... ..........
D10            .......... .......A.. .......... .......... .......... .......... .......... ..........
FCC2           .......... .......A.. .......... .......... .......... .......... .......... ..........
HB3            .......... .......... .......... .......... .......... .......... .......... ..........
7G8            .......... .......... .......... .......... .......... .......... .......... ..........
P.reichenowi   C......... .........A .......... .......... .......... .......... .......... ..........

                       1210       1220       1230       1240       1250       1260       1270       1280
               ....|....| ....|....| ....|....| ....|....| ....|....| ....|....| ....|....| ....|....|
PFL1385c       GCATATGTAT CATATAAGAA AAGAAAAGCT CAAGAAAAAG GATTACCAGA ACCTACTGTT ACTAATGAAG AATATGTTGA
3D7            .......... .......... .......... .......... .......... .......... .......... ..........
RO33           .......... .......... .......... .......... .......... .......... .......... ..........
Palo Alto      .......... .......... .......... .......... .......... .......... .......... ..........
FCR3           .......... .......... .......... .......... .......... .......... .......... ..........
Wellcome       .......... .......... .......... .......... .......... .......... .......... ..........
D6             .......... .......... .......... .......... .......... .......... .......... ..........
T996           .......... .......... .......... .......... .......... .......... .......... ..........
T9102          .......... .......... .......... .......... .......... .......... .......... ..........
K1             .......... .......... .......... .......... .......... .......... .......... ..........
Dd2            .......... .......... .......... .......... .......... .......... .......... ..........
D10            .......... .......... .......... .......... .......... .......... .......... ..........
FCC2           .......... .......... .......... .......... .......... .......... .......... ..........
HB3            .......... .......... .......... .......... .......... .......... .......... ..........
7G8            .......... .......... .......... .......... .......... .......... .......... ..........
P.reichenowi   .......... .......... ......G... .......... .......... .......... .......... ..........
```

```
                            1290       1300       1310       1320       1330       1340       1350       1360
                    ....|....| ....|....| ....|....| ....|....| ....|....| ....|....| ....|....| ....|....|
PFL1385c            AGAATTAAAG AAAGGTATTC TAGATATGGG TATCAAATTA TTATTTAGTA AAGTTAAAAG CCTATTAAAA AAATTAAAAA
3D7                 .......... .......... .......... .......... .......... .......... .......... ..........
RO33                .......... .......... .......... .......... .......... .......... .......... ..........
Palo Alto           .......... .......... .......... .......... .......... .......... .......... ..........
FCR3                .......... .......... .......... .......... .......... .......... .......... ..........
Wellcome            .......... .......... .......... .......... .......... .......... .......... ..........
D6                  .......... .......... .......... .......... .......... .......... .......... ..........
T996                .......... .......... .......... .......... .......... .......... .......... ..........
T9102               .......... .......... .......... .......... .......... .......... .......... ..........
K1                  .......... .......... .......... .......... .......... .......... .......... ..........
Dd2                 .......... .......... .......... .......... .......... .......... .......... ..........
D10                 .......... .......... .......... .......... .......... .......... .......... ..........
FCC2                .......... .......... .......... .......... .......... .......... .......... ..........
HB3                 .......... .......... .......... .......... .......... .......... .......... ..........
7G8                 .......... .......... .......... .......... .......... .......... .......... ..........
P.reichenowi        .......... .......... .......... ...T...... .......... .......... .T.....TC. ..........

                            1370       1380       1390       1400       1410       1420       1430       1440
                    ....|....| ....|....| ....|....| ....|....| ....|....| ....|....| ....|....| ....|....|
PFL1385c            ATAAAATATT CCCTAAGAAA AAAGAAGATA ATCAAGCAGT AGATACCAAA AGTATGGAAG AACCCAAAGT TAAAGCACAA
3D7                 .......... .......... .......... .......... .......... .......... .......... ..........
RO33                .......... .......... .......... .......... .......... .......... .......... ..........
Palo Alto           .......... .......... .......... .......... .......... .......... .......... ..........
FCR3                .......... .......... .......... .......... .......... .......... .......... ..........
Wellcome            .......... .......... .......... .......... .......... .......... .......... ..........
D6                  .......... .......... .......... .......... .......... .......... .......... ..........
T996                .......... .......... .......... .......... .......... .......... .......... ..........
T9102               .......... .......... .......... .......... .......... .......... .......... ..........
K1                  .......... .......... .......... .......... .......... .......... .......... ..........
Dd2                 .......... .......... .......... .......... .......... .......... .......... ..........
D10                 .......... .......... .......... .......... .......... .......... .......... ..........
FCC2                .......... .......... .......... .......... .......... .......... .......... ..........
HB3                 .......... .......... .......... .........C .......... .......... .......... ..........
7G8                 .......... .......... .......... .......... .......... .......... .......... ..........
P.reichenowi        .......... .......... ......C.A. .......... ......A... .A.CCA.G.. ..A.T..... ..........

                            1450       1460       1470       1480       1490       1500       1510       1520
                    ....|....| ....|....| ....|....| ....|....| ....|....| ....|....| ....|....| ....|....|
PFL1385c            CCAGCTCTTA GAGGTGTTGA ACCAACGGAA GATTCTAATA TTATGAACAG TATTAATAAT GTTATGGATG AAATTGATTT
3D7                 .......... .......... .......... .......... .......... .......... .......... ..........
RO33                .......... .......... .......... .......... .......... .......... .......... ..........
Palo Alto           .......... .......... .......... .......... .......... .......... .......... ..........
FCR3                .......... .......... .......... .......... .......... .......... .......... ..........
Wellcome            .......... .......... .......... .......... .......... .......... .......... ..........
D6                  .......... .......... .......... .......... .......... .......... .......... ..........
T996                .......... .......... .......... .......... .......... .......... .......... ..........
T9102               .......... .......... .......... .......... .......... .......... .......... ..........
K1                  .......... .......... .......... .......... .......... .......... .......... ..........
Dd2                 .......... .......... .......... .......... .......... .......... .......... ..........
D10                 .......... .......... .......... .......... .......... .......... .......... ..........
FCC2                .......... .......... .......... .......... .......... .......... .......... ..........
HB3                 .......... .......... .......... .......... .......... .......... .......... ..........
7G8                 .......... .......... .......... .......... .......... .......... .......... ..........
P.reichenowi        .......... ......G... ......T... .......... .......... .......... .......... ..........

                            1530       1540       1550       1560       1570       1580       1590       1600
                    ....|....| ....|....| ....|....| ....|....| ....|....| ....|....| ....|....| ....|....|
PFL1385c            CTTTGAAAAA GAATTAATCG AAAATAATAA TACACCTAAT GTTGTACCAC CAACTCAATC AAAAAAAAAA AACAAAAATG
3D7                 .......... .......... .......... .......... .......... .......... .......... ..........
RO33                .......... .......... .......... .......... .......... .......... .......... ..........
Palo Alto           .......... .......... .......... .......... .......... .......... .......... ..........
FCR3                .......... .......... .......... .......... .......... .......... .......... ..........
Wellcome            .......... .......... .......... .......... .......... .......... .......... ..........
D6                  .......... .......... .......... .......... .......... .......... .......... ..........
T996                .......... .......... .......... .......... .......... .......... .......... ..........
T9102               .......... .......... .......... .......... .......... .......... .......... ..........
K1                  .......... .......... .......... .......... .......... .......... .......... ..........
Dd2                 .......... .......... .......... .......... .......... .......... .......... ..........
D10                 .......... .......... .......... .......... .......... .......... .......... ..........
FCC2                .......... .......... .......... .......... .......... .......... .......... ..........
HB3                 .......... .......... .......... .......... .......... .......... .......... ..........
7G8                 .......... .......... .......... .......... .......... .......... .......... ..........
P.reichenowi        .......... .......... .......... .......... .......... .......... .........C ---.......
```

```
                            1610       1620       1630       1640       1650       1660       1670       1680
                      ....|....| ....|....| ....|....| ....|....| ....|....| ....|....| ....|....| ....|....|
PFL1385c              AAACTGTATC TGGTATGGAT GAAAATTTTG ATAATCATCC TGAAAATTAT TTTAAAGAAG AATATTATTA TGATGAAAAT
3D7                   .......... .......... .......... .......... .......... .......... .......... ..........
RO33                  .......... .......... .......... .......... .......... .......... .......... ..........
Palo Alto             .......... .......... .......... .......... .......... .......... .......... ..........
FCR3                  .......... .......... .......... .......... .......... .......... .......... ..........
Wellcome              .......... .......... .......... .......... .......... .......... .......... ..........
D6                    .......... .......... .......... .......... .......... .......... .......... ..........
T996                  .......... .......... .......... .......... .......... .......... .......... ..........
T9102                 .......... .......... .......... .......... .......... .......... .......... ..........
K1                    .......... .......... .......... .......... .......... .......... ......T... ..........
Dd2                   .......... .......... .......... .......... .......... .......... .......... ..........
D10                   .......... .......... .......... .......... .......... .......... .......... ..........
FCC2                  .......... .......... .......... .......... .......... .......... .......... ..........
HB3                   .......... .......... .......... .......... .......... .......... .......... ..........
7G8                   .......... .......... .......... .......... .......... .......... .......... ..........
P.reichenowi          T......... A......AG. .......... .......... .......... .......... .......... ..........

                            1690       1700       1710       1720       1730       1740       1750       1760
                      ....|....| ....|....| ....|....| ....|....| ....|....| ....|....| ....|....| ....|....|
PFL1385c              GATGATATGG AAGTAAAAGT TAAAAAAATA GGTGTCACAT TAAAAAAATT TGAACCACTT AAAAATGGAA ATGTTAGTGA
3D7                   .......... .......... .......... .......... .......... .......... .......... ..........
RO33                  .......... .......... .......... .......... .......... .......... .......... ..........
Palo Alto             .......... .......... .......... .......... .......... .......... .......... ..........
FCR3                  .......... .......... .......... .......... .......... .......... .......... ..........
Wellcome              .......... .......... .......... .......... .......... .......... .......... ..........
D6                    .......... .......... .......... .......... .......... .......... .......... ..........
T996                  .......... .......... .......... .......... .......... .......... .......... ..........
T9102                 .......... .......... .......... .......... .......... .......... .......... ..........
K1                    .......... .......... .......... .......... .......... .......... .......... ..........
Dd2                   .......... .......... .......... .......... .......... .......... .......... ..........
D10                   .......... .......... .......... .......... .......... .......... .......... ..........
FCC2                  .......... .......... .......... .......... .......... .......... .......... ..........
HB3                   .......... .......... .......... .......... .......... .......... .......... ..........
7G8                   .......... .......... .......... .......... .......... .......... .......... ..........
P.reichenowi          .......... ....T..... .........C .......... .......... .......... .......... ..C.......

                            1770       1780       1790       1800       1810       1820       1830       1840
                      ....|....| ....|....| ....|....| ....|....| ....|....| ....|....| ....|....| ....|....|
PFL1385c              AACCATTAAA TTGATTCATT TAGGAAATAA AGATAAAAAA CACATTGAAG CTATAAACAA CGATATTCAA ATTATTAAAC
3D7                   .......... .......... .......... .......... .......... .......... .......... ..........
RO33                  .......... .......... .......... .......... .......... .......... .......... ..........
Palo Alto             .......... .......... .......... .......... .......... .......... .......... ..........
FCR3                  .......... .......... .......... .......... .......... .......... .......... ..........
Wellcome              .......... .......... .......... .......... .......... .......... .......... ..........
D6                    .......... .......... .......... .......... .......... .......... .......... ..........
T996                  .......... .......... .......... .......... .......... .......... .......... ..........
T9102                 .......... .......... .......... .......... .......... .......... .......... ..........
K1                    .......... .......... .......... .......... .......... .......... .......... ..........
Dd2                   .......... .......... .......... .......... .......... .......... .......... ..........
D10                   .......... .......... .......... .......... .......... .......... .......... ..........
FCC2                  .......... .......... .......... .......... .......... .......... .......... ..........
HB3                   .......... .......... .......... .......... .......... .......... .......... ..........
7G8                   .......... .......... .......... .......... .......... .......... .......... ..........
P.reichenowi          .......... ..TG...... .......... .......... .....A.... .......... .......... G.........

                            1850       1860       1870       1880       1890       1900       1910       1920
                      ....|....| ....|....| ....|....| ....|....| ....|....| ....|....| ....|....| ....|....|
PFL1385c              AAGAATTACA AGCTATTTAT AATGAACTTA TGAATTATAC AAATGGAAAC AAAAATATTC AACAAATATT TCAACAAAAT
3D7                   .......... .......... .......... .......... .......... .......... .......... ..........
RO33                  .......... .......... .......... .......... .......... .......... .......... ..........
Palo Alto             .......... .......... .......... .......... .......... .......... .......... ..........
FCR3                  .......... .......... .......... .......... .......... .......... .......... ..........
Wellcome              .......... .......... .......... .......... .......... .......... .......... ..........
D6                    .......... .......... .......... .......... .......... .......... .......... ..........
T996                  .......... .......... .......... .......... .......... .......... .......... ..........
T9102                 .......... .......... .......... .......... .......... .......... .......... ..........
K1                    .......... .......... .......... .......... .......... .......... .......... ..........
Dd2                   .......... .......... .......... .......... .......... .......... .......... ..........
D10                   .......... .......... .......... .......... .......... .......... .......... ..........
FCC2                  .......... .......... .......... .......... .......... .......... .......... ..........
HB3                   .......... .......... .......... .......... .......... .......... .......... ..........
7G8                   .......... .......... .......... .......... .......... .......... .......... ..........
P.reichenowi          .......... .......... .......... ....C..... .......... .....CG... .....C.... ..........
```

```
                         1930      1940      1950      1960      1970      1980      1990      2000
                ....|....| ....|....| ....|....| ....|....| ....|....| ....|....| ....|....| ....|....|
PFL1385c        ATTCTAGAAA ATGATGTTCT TAATCAAGAA ACGGAGGAAG AAATGGAAAA ACAAGTTGAA GCAATCACCA AGCAAATAGA
3D7             .......... .......... .......... .......... .......... .......... .......... ..........
RO33            .......... .......... .......... .......... .......... .......... .......... ..........
Palo Alto       .......... .......... .......... .......... .......... .......... .......... ..........
FCR3            .......... .......... .......... .......... .......... .......... .......... ..........
Wellcome        .......... .......... .......... .......... .......... .......... .......... ..........
D6              .......... .......... .......... .......... .......... .......... .......... ..........
T996            .......... .......... .......... .......... .......... .......... .......... ..........
T9102           .......... .......... .......... .......... .......... .......... .......... ..........
K1              .......... .......... .......... .......... .......... .......... .......... ..........
Dd2             .......... .......... .......... .......... .......... .......... .......... ..........
D10             .......... .......... .......... .......... .......... .......... .......... ..........
FCC2            .......... .......... .......... .......... .......... .......... .......... ..........
HB3             .......... .......... .......... .......... .......... .......... .......... ..........
7G8             .......... .......... .......... .......... .......... .......... .......... ..........
P.reichenowi    .C........ ....A..... .......... ..A....... .......... .......... ..C....A.. ..........

                         2010      2020      2030      2040      2050      2060      2070      2080
                ....|....| ....|....| ....|....| ....|....| ....|....| ....|....| ....|....| ....|....|
PFL1385c        AGCTGAAGTG GATGCCCTCG CACCAAAAAA TAAGGAAGAA GAAGAAAAAG AAAAGGAAAA AGAAGAAAAA GAAAAAGAAG
3D7             .......... .......... .......... .......... .......... .......... .......... ..........
RO33            .......... .......... .......... .......... .......... ....A..... ....A.GG.. A..G.....A
Palo Alto       .......... .......... .......... .......... .......... ....A..... ....A.GG.. A..G.....A
FCR3            .......... .......... .......... .......... .......... ....A..... ....A.GG.. A..G.....A
Wellcome        .......... .......... .......... .......... .......... ....A..... ....A.GG.. A..G.....A
D6              .......... .......... .......... .......... .......... ....A..... ....A.GG.. A..G.....A
T996            .......... .......... .......... .......... .......... ....A..... ....A.GG.. A..G.....A
T9102           .......... .......... .......... .......... .......... ....A..... ....A.GG.. A..G.....A
K1              .......... .......... .......... .......... .......... ....A..... ....A.GG.. A..G.....A
Dd2             .......... .......... .......... .......... .......... ....A..... ....A.GG.. A..G.....A
D10             .......... .......... .......... .......... .......... ....A..... ....A.GG.. A..G.....A
FCC2            .......... .......... .......... .......... .......... ....A..... ....A.GG.. A..G.....A
HB3              ......... .......... .......... .......... .......... ....A..... ....A.GG.. A..G.....A
7G8             .......... .......... .......... .......... .......... ....A..... ....A.GG.. A..G.....A
P.reichenowi    .......... .....A.... .......... .......... A........

                         2090      2100      2110      2120      2130      2140      2150      2160
                ....|....| ....|....| ....|....| ....|....| ....|....| ....|....| ....|....| ....|....|
PFL1385c        AAAAAGAAAA AGAAAAAGAA GAAAAAGAAA AAGAAGAAAA AGAAAAAGAA GAAAAAGAAA AAGAAGAAAA AGAAGAAGAA
3D7             .......... .......... .......... .......... .......... .......... .......... ..........
RO33            ..G..A..G. .......... A..G...... .....A..G. .......... A..G..A..G .......... .A.....A..
Palo Alto       ..G..A..G. .......... A..G...... .....A..G. .A..G..... A..G..A..G ..A.....G. .A........
FCR3            ..G..A..G. .......... A..G..A..G .....A..G. .A..G..A.. .........G ..A.....G. ....A..A..
Wellcome        ..G..A..G. .......... A..G..A..G .....A..G. .A..G..A.. .........G ..A.....G. ....A..A..
D6              ..G..A..G. .......... A..G...... .....A..G. .......... .........G .....A.... ....A.....
T996            ..G..A..G. .......... A..G..A..G .....A..G. .A..G..... A..G..A..G ..A.....G. .A........
T9102           ..G..A..G. .......... A..G...... .....A..G. .......... A..G...... .....A..G. .A........
K1              ..G..A..G. .A..G..... A..G..A..G .....A..G. .A..G..A.. ...G..A..G .......... .A.....A..
Dd2             ..G..A..G. .......... A..G...... .....A..G. .A..G..... A..G.....G ..A..A..G. .A........
D10             ..G..A..G. .......... A..G..A..G .....A..G. .A..G..... A..G..A..G .....A..G. ....A.....
FCC2            ..G..A..G. .......... A..G...... .....A..G. .A..G..... A..G...... ........G. .A..A.....
HB3             .GG..A..G. .......... A..G...... .....A..G. .......... A..G...... .....A..G. ....A.....
7G8             ..G..A..G. .......... A..G...... .....A..G. .A..G..... ...G.....G ........G. .A........
P.reichenowi

                         2170      2180      2190      2200      2210      2220      2230      2240
                ....|....| ....|....| ....|....| ....|....| ....|....| ....|....| ....|....| ....|....| ....
PFL1385c        AAAAAAGAAA AAGAAGAAGA ACAAGAAGAA GAAGAAGAAG AAGAAATAGT ACCAGAAAAT TTGACAACTG AAGAATCAAA ATAA
3D7             .......... .......... .......... .......... .......... .......... .......... .......... ....
RO33            G..G.....C .......... .G.....
Palo Alto       G.....A..G ..A....... .G..C..... .......... .......... ....
FCR3            G........G .....C.... .
Wellcome        G........G .....C.... .G........ ......
D6              G..G..C..G .......... .G.....AT. .T.CC.
T996            G.....A..G ..A....... .G..C..... .......... .....GA.A. .GT.CC.
T9102           ...G...... .......... .A..A..... A......... .....GA..A .GA.
K1              G..G.....C .......... .G........
Dd2             G..C.....G .......... .G..AT..T. CC...
D10             G..G..A... .....A.... .G.....C.. .......... .....GA.A. .GT.
FCC2            ...G.....G ..C....... .G........ ...
HB3             G........G .....A..A. .G..A..... ......
7G8             G..G.....G .......... .G........ ..
P.reichenowi
```

PFB0340c –SERA5

```
                      10         20         30         40         50         60         70         80
              ....|....| ....|....| ....|....| ....|....| ....|....| ....|....| ....|....| ....|....|
PFB0340c      ATGAAGTCAT ATATTTCCTT GTTTTTCATA TTGTGTGTTA TATTTAACAA AAATGTTATA AAATGTACAG GA--------
3D7
RO33                                              G.G.... .......... .......... .......... ..--------
Palo Alto
FCR3
Wellcome
D6
T996
T9102                                             G...... .......... .......... .......... ..--------
K1
Dd2                                                                       ..A... C.G------- ----------
D10
FCC2                                              G.G.... .......... .......... .......... ..--------
HB3
7G8
P.reichenowi  .......... .......... .......... .......... .......... .......... .......... ..GGAAGTAC

                      90        100        110        120        130        140        150        160
              ....|....| ....|....| ....|....| ....|....| ....|....| ....|....| ....|....| ....|....|
PFB0340c      ---------- ---------- ---------- ---------- ---------- ---------- ---------- ----------
3D7           ---------- ---------- ---------- ---------- ---------- ---------- ---------- ----------
RO33
Palo Alto
FCR3
Wellcome
D6
T996
T9102         ---------- ---------- ---------- ---------- ---------- ---------- ---------- ----------
K1
Dd2           ---------- ---------- ---------- ---------- ---------- ---------- ---------- ----------
D10
FCC2          ---------- ---------- ---------- ---------- ---------- ---------- ---------- ----------
HB3
7G8
P.reichenowi  ATCAACAAGT CCCGAACAAA GTAATCCTGG AAGTACAAGA GGAAGCACAC CATCAAGTTC AGTAGAAAGT AATTCTGTAA

                     170        180        190        200        210        220        230        240
              ....|....| ....|....| ....|....| ....|....| ....|....| ....|....| ....|....| ....|....|
PFB0340c      ---------- ---------- ---------- ---------- ---------- ---------- ---------- ----------
3D7
RO33          ---------- ---------- ---------- ---------- ---------- ---------- ---------- ----------
Palo Alto
FCR3
Wellcome
D6
T996
T9102         ---------- ---------- ---------- ---------- ---------- ---------- ---------- ----------
K1
Dd2           ---------- ---------- ---------- ---------- ---------- ---------- ---------- ----------
D10
FCC2          ---------- ---------- ---------- ---------- ---------- ---------- ---------- ----------
HB3
7G8
P.reichenowi  GTACAGGAGG AAGTACATCA TCAAGTTCCG TACAAAGTAA TTCTGTAAGT ACAGGAGAAA ATTCACCATC AAGTTCCGTA

                     250        260        270        280        290        300        310        320
              ....|....| ....|....| ....|....| ....|....| ....|....| ....|....| ....|....| ....|....|
PFB0340c      ---------- ---------- ---------- ---------- ---------- ---------- ---------- --------GA
3D7
RO33          ---------- ---------- ---------- ---------- ---------- ---------- ---------- --------A.
Palo Alto
FCR3
Wellcome
D6
T996
T9102         ---------- ---------- ---------- ---------- ---------- ---------- ---------- --------..
K1
Dd2           ---------- ---------- ---------- ---------- ---------- ---------- ---------- ----------
D10
FCC2          ---------- ---------- ---------- ---------- ---------- ---------- ---------- --------A.
HB3
7G8
P.reichenowi  GAAAGTAATT CTGGAAGTAC AGGAGAAAGT TCACCACCAA GTTCCGTAGA AAGTAATTCT GGAAGTACAG GACAAACT--
```

```
                        330        340        350        360        370        380        390        400
               ....|....| ....|....| ....|....| ....|....| ....|....| ....|....| ....|....| ....|....|
PFB0340c       AAGTCAAACA GGTAATACAG GAGGAGGTCA AGCAGGTAAT ACAGGAGGAG AT-------- ---------- ------CAAG
3D7                             .... .......... .......... .......... ..-------- ---------- ------....
RO33           .........C C......... .......... .......... ....T..... ..CAAGCAGG TAATACAGTA GGAGAT....
Palo Alto                          . .......... .......... .......... G.CAAGCAGG TAATACAGTA GGAGAT....
FCR3                                                       .......... G.CAAGCAGG TAATACAGTA GGAGAT....
Wellcome                                   ..... .......... .......... G.CAAGCAGG TAATACAGTA GGAGAT....
D6                                ........ .......... ....T..... ..CAAGCAGG TAATACAGTA GGAAGT----
T996                                        .. .......... .......... G.CAAGCAGG TAATACAGTA GGAGAT....
T9102          .......... .......... .......... .......... ....T..... G.CAAGCAGG TAATACAGTA GGAGAT....
K1                                 . .......... .......... .......... ..-------- ---------- ------....
Dd2            ---------- ---------. AG.AG.TCA. .......... .......... G.CAAGCAGG TAATACAGTA GGAGAT....
D10                                                                 . G.CAAGCAGG TAATACAGGA GGAGAT....
FCC2           .......... .......... .......... .......... ....T..... G.CAAGCAGG TAATACAGTA GGAGAT....
HB3                                                        .......... G.CAAGCAGG TAATACAGTA GGAGAT....
7G8                                                        .......... G.CAAGCAGG TAATACAGTA GGAGAT....
P.reichenowi   ---------- ---------- ---------- ---------- ---------- ---------- ---------- ----------

                        410        420        430        440        450        460        470        480
               ....|....| ....|....| ....|....| ....|....| ....|....| ....|....| ....|....| ....|....|
PFB0340c       CAGGTAGTAC AGGAGGAAGT CCACAAGGTA GTACGGGAGC AAGTCCACAA GGTAGTACGG GAGCAAGTCC ACAAGGTAGT
3D7            .......... .......... .......... .......... .......... .......... .......... ..........
RO33           .......... .......... .......... .......... ....------ ---------- ---------- ----------
Palo Alto      .......... .......... .......... .......... ....------ ---------- ---------- ----------
FCR3           .......... .......... .......... .......... ....------ ---------- ---------- ----------
Wellcome       .......... .......... .......... .......... ....------ ---------- ---------- ----------
D6             ---------- ---------- .......... .......... .......... .......... ........-- ----------
T996           .......... .......... .......... .......... ....------ ---------- ---------- ----------
T9102          .......... .......... .......... .......... ....------ ---------- ---------- ----------
K1             .......... ........A. ....C..... .......... .......... .......... .......... ..CC......
Dd2            .......... .......... .......... .......... ....------ ---------- ---------- ----------
D10            .......... .......... .......... .......... ....------ ---------- ---------- ----------
FCC2           .......... .......... .......... .......... ....------ ---------- ---------- ----------
HB3            .......... .......... .......... .......... ....------ ---------- ---------- ----------
7G8            .......... .......... .......... .......... ....------ ---------- ---------- ----------
P.reichenowi   ---------- ---------- ---------- ---------- ---------- ---------- ---------- ----------

                        490        500        510        520        530        540        550        560
               ....|....| ....|....| ....|....| ....|....| ....|....| ....|....| ....|....| ....|....|
PFB0340c       ACGGGAGCAA GTCAACCCGG AAGTTCCGAA CCAAGCAATC CTGTAAGTTC CGGACATTCT GTAAGTACTG TATCAGTATC
3D7            .......... .......... .......... .......... .......... .......... .......... ..........
RO33           ---------- --........ .......... .......... .......... .......... .......... ..........
Palo Alto      ---------- --........ .......... .......... .......... .......... .......... ..........
FCR3           ---------- --........ .......... .......... .......... .......... .......... ..........
Wellcome       ---------- --........ .......... .......... .......... .......... .......... ..........
D6             ---------- --........ .......... .......... .......... .......... .......... ..........
T996           ---------- --........ .......... .......... .......... .......... .......... ..........
T9102          ---------- --........ .......... .......... .......... .......... .......... ..........
K1             ....A..... ....T..... .......... ..TT...... .....G.... .......... ....C..... ......A...
Dd2            ---------- --........ .......... .......... .......... .......... .......... ..........
D10            ---------- --........ .......... .......... .......... .......... .......... ..........
FCC2           ---------- --........ .......... .......... .......... .......... .......... ..........
HB3            ---------- --........ .......... .......... .......... .......... .......... ..........
7G8            ---------- --........ .......... .......... .......... .......... .......... ..........
P.reichenowi   ---------- ---------- ---------- ---------- ---------- ---------- .C...C...C C..T....A.

                        570        580        590        600        610        620        630        640
               ....|....| ....|....| ....|....| ....|....| ....|....| ....|....| ....|....| ....|....|
PFB0340c       ACAAACTTCA ACTTCTTCAG AAAAACAGGA TACAATTCAA GTAAAATCAG CTTTATTAAA AGATTATATG GGTTTAAAAG
3D7            .......... .......... .......... .......... .......... .......... .......... ..........
RO33           .......... .......... .......... .......... .......... .......... .......... ..........
Palo Alto      .......... .......... .......... .......... .......... .......... .......... ..........
FCR3           .......... .......... .......... .......... .......... .......... .......... ..........
Wellcome       .......... .......... .......... .......... .......... .......... .......... ..........
D6             .......... .......... .......... .......... .......... .......... .......... ..........
T996           .......... .......... .......... .......... .......... .......... .......... ..........
T9102          .......... .......... .......... .......... .......... .......... .......... ..........
K1             .......... .......... .......... .......... .......... .......... .......... ..........
Dd2            .......... .......... .......... .......... .......... .......... .......... ..........
D10            .......... .......... .......... .......... .......... .......... .......... ..........
FCC2           .......... .......... .......... .......... .......... .......... .......... ..........
HB3            .......... .......... .......... .......... .......... .......... .......... ..........
7G8            .......... .......... .......... .......... .......... .......... .......... ..........
P.reichenowi   ....T..... .......... C...T..AAG .......... .....G.... .......... .......... ..........
```

```
                               650        660        670        680        690        700        710        720
                        ....|....| ....|....| ....|....| ....|....| ....|....| ....|....| ....|....| ....|....|
PFB0340c                TTACTGGTCC ATGTAACGAA AATTTCATAA TGTTCTTAGT TCCTCATATA TATATTGATG TTGATACAGA AGATACTAAT
3D7                     .......... .......... .......... .......... .......... .......... .......... ..........
RO33                    .......... .......... .......... .......... .......... .......... .......... ..........
Palo Alto               .......... .......... .......... .......... .......... .......... .......... ..........
FCR3                    .......... .......... .......... .......... .......... .......... .......... ..........
Wellcome                .......... .......... .......... .......... .......... .......... .......... ..........
D6                      .......... .......... .......... .......... .......... .......... .......... ..........
T996                    .......... .......... .......... .......... .......... .......... .......... ..........
T9102                   .......... .......... .......... .......... .......... .......... .......... ..........
K1                      .......... .......... .......... .......... .......... .......... .......... ..........
Dd2                     .......... .......... .......... .......... .......... .......... .......... ..........
D10                     .......... .......... .......... .......... .......... .......... .......... ..........
FCC2                    .......... .......... .......... .......... .......... .......... .......... ..........
HB3                     .......... .......... .......... .......... .......... .......... .......... ..........
7G8                     .......... .......... .......... .......... .......... .......... .......... ..........
P.reichenowi            .......... .......... .......... .......... .......... .......... .......... .......T..

                               730        740        750        760        770        780        790        800
                        ....|....| ....|....| ....|....| ....|....| ....|....| ....|....| ....|....| ....|....|
PFB0340c                ATCGAATTAA GAACAACATT GAAAAAAACA AATAATGCAA TATCATTTGA ATCAAACAGT GGTTCATTAG AAAAAAAAAA
3D7                     .......... .......... .......... .......... .......... .......... .......... ..........
RO33                    .......... .......... .......... .......... .......... .......... .......... ..........
Palo Alto               .......... .......... .......... .......... .......... .......... .......... ..........
FCR3                    .......... .......... ....G..... .......... .......... .......... .......... ..........
Wellcome                .......... .......... ....G..... .......... .......... .......... .......... ..........
D6                      .......... .......... .......... .......... .......... .......... .......... ..........
T996                    .......... .......... .......... .......... .......... .......... .......... ..........
T9102                   .......... .......... ....G..... .......... .......... .......... .......... ..........
K1                      .......... .......... .......... .......... .......... .......... .......... ..........
Dd2                     .......... .......... ....G..... .......... .......... .......... .......... ..........
D10                     .......... .......... ....G..... .......... .......... .......... .......... ..........
FCC2                    .......... .......... ....G..... .......... .......... .......... .......... ..........
HB3                     .......... .......... ....G..... .......... .......... .......... .......... ..........
7G8                     .......... .......... ....G..... .......... .......... .......... .......... ..........
P.reichenowi            .......... .......... ....G..... .G.......T .......... .G........ .......... ..........

                               810        820        830        840        850        860        870        880
                        ....|....| ....|....| ....|....| ....|....| ....|....| ....|....| ....|....| ....|....|
PFB0340c                ATATGTAAAA CTACCATCAA ATGGTACAAC TGGTGAACAA GGTTCAAGTA CGGGAACAGT TAGAGGAGAT ACAGAACCAA
3D7                     .......... .......... .......... .......... .......... .......... .......... ..........
RO33                    .......... .......... .......... .A...C.... .......... .......... .......... ......T...
Palo Alto               .......... .......... .......... .......... .......... .......... .......... ......T...
FCR3                    .......... .......... .......... .......... A....T...- ---------- ---------- ----------
Wellcome                .......... .......... .......... .......... A....T...- ---------- ---------- ----------
D6                      .......... .......... .......... .......... .......... .......... .......... ..........
T996                    .......... .......... .......... .......... .......... .......... .......... ......T...
T9102                   .......... .......... .......... .......... .......... .......... .......... ......T...
K1                      .......... .......... .......... .......... .......... .......... .......... ..........
Dd2                     .......... .......... .......... .......... .......... .......... .......... ......T...
D10                     .......... .......... .......... .......... A....T...- ---------- ---------- ----------
FCC2                    .......... .......... .......... .......... .......... .......... .......... ..........
HB3                     .......... .......... .......... .......... .......... .......... .......... ..........
7G8                     .......... .......... .......... .......... .......... .......... .......... ..........
P.reichenowi            .......... ....A..... .......... .A........ AA....G.A. .A.....G.. .CA....A.. GA....T...

                               890        900        910        920        930        940        950        960
                        ....|....| ....|....| ....|....| ....|....| ....|....| ....|....| ....|....| ....|....|
PFB0340c                TTTCAGATTC AAGCTCAAGT TCAAGTTCAA GCTCTAGTTC AAGTTCAAGT TCAAGTTCAA GTTCTAGTTC AAGTTCTAGT
3D7                     .......... .......... .......... .......... .......... .......... .......... ..........
RO33                    .......... .......... ........T. TT..A..... .......... ..T....... ....A..... ..........
Palo Alto               .......... .......... .......... .......... ...C...... ..T....... .C..A..C.. .---------
FCR3                    --------.. ...T...... ..T....... AT........ .......... .......... .......... ......A...
Wellcome                --------.. ...T...... ..T....... AT........ .......... .......... .......... ......A...
D6                      .......... .......... ........T. .T..A..... .......... ..T....... ....A..... ..........
T996                    .......... .......... .......... .......... ...C...... .......... .C........ ...C..A...
T9102                   .......... .......... .......... .......... ...C...... ..T....... .C..A..C.. .---------
K1                      .......... .......... .......... .......... .......... .......... .......... ..........
Dd2                     .......... .......... .......... .......... ...C...... ..T....... .C..A..C.. .---------
D10                     --------.. ...T...... ..T....... .T..A..... T......... .......... ....A..... ..........
FCC2                    ......G... .......... ........T. .T..A..... .......... ..T....... ....A..... ......A...
HB3                     .......... .......... .......... .T........ .......... ........T. ....A..... ......A...
7G8                     .......... .......... ........T. .T..A..... .......... ..T....... ....A..... ......A...
P.reichenowi            G......... ......C... .......... .T..AGAAC. .......GA. .....C..G. ....A..... ......A..C
```

```
                  970        980        990        1000       1010       1020       1030       1040
             ....|....| ....|....| ....|....| ....|....| ....|....| ....|....| ....|....| ....|....|
PFB0340c     TCAAGTTCA- ---------- ---------- ---------- ---------- -------GAA AGTCTTCCTG CTAATGGACC
3D7          .........- ---------- ---------- ---------- ---------- -------... .......... ..........
RO33         .........A GTTCAAGTTC AAGTTCTAGT TCAAGTTCA- ---------- -------... .......... ..........
Palo Alto    ---------- ---------- ---------- ---------- ---------- -------.T. .A..CC.... ........G.
FCR3         ........TA GTTCAAGTTC TAGTTCAAGT TCA------- ---------- -------... .......... ..........
Wellcome     ........TA GTTCAAGTTC TAGTTCAAGT TCA------- ---------- -------... .......... ..........
D6           ........TA GTTCAAGTTC A--------- ---------- ---------- -------... .......... ..........
T996         ..T......A GTTCTAGTTC AAGTTCA--- ---------- ---------- -------... .......... ..........
T9102        ---------- ---------- ---------- ---------- ---------- -------.T. .A..CC.... ........G.
K1           .........- ---------- ---------- ---------- ---------- -------... .......... ..........
Dd2          ---------- ---------- ---------- ---------- ---------- -------.T. .A..CC.... ........G.
D10          .........A GTTCTAGTTC AAGTTCTAGT TCAAGTTCAA GTTCTAGTTC AAGTTCA... .......... ..........
FCC2         ..T......A GTTCTAGTTC AAGTTCA--- ---------- ---------- -------... .......... ..........
HB3          ........TA GTTCAAGTTC TAGTTCAAGT TCA------- ---------- -------... .......... ..........
7G8          ..T......A GTTCTAGTTC AAGTTCA--- ---------- ---------- -------... .......... ..........
P.reichenowi ..C......A GTTCTAGTTC TAGTTCAAGC TCGAGTTCAG GTTCA----- -------.T. .A.ACC.... ........G.

                  1050       1060       1070       1080       1090       1100       1110       1120
             ....|....| ....|....| ....|....| ....|....| ....|....| ....|....| ....|....| ....|....|
PFB0340c     TGATTCCCCT ACTGTTAAAC CGCCAAGAAA TTTACAAAAT ATATGTGAAA CTGGAAAAAA CTTCAAGTTG GTAGTATATA
3D7          .......... .......... .......... .......... .......... .......... .......... ..........
RO33         .......... .......... .......... .......... .......... .......... .......... ..........
Palo Alto    ..G...TA.. C...A.GC.A A.AA..A... .......... .......... .......... .......... ..........
FCR3         .......... .......... .......... .......... .......... .......... .......... ..........
Wellcome     .......... .......... .......... .......... .......... .......... .......... ..........
D6           .......... .......... .......... .......... .......... .......... .......... ..........
T996         .......... .......... .......... .......... .......... .......... .......... ..........
T9102        ..G...TA.. C...A.GC.A A.AA..A... .......... .......... .......... .......... ..........
K1           .......... C......... .......... .......... .......... .......... .......... ..........
Dd2          ..G...TA.. C...A.GC.A A.AA..A... .......... .......... .......... .......... ..........
D10          .......... .......... .......... .......... .......... .......... .......... ..........
FCC2         .......... .......... .......... .......... .......... .......... .......... ..........
HB3          .......... .......... .......... .......... .......... .......... .......... ..........
7G8          .......... .......... .......... .......... .......... .......... .......... ..........
P.reichenowi .AG....AA. C...A.GC.A A.AA...... ....A.T... .......C.C .........C G.....A..T ..........

                  1130       1140       1150       1160       1170       1180       1190       1200
             ....|....| ....|....| ....|....| ....|....| ....|....| ....|....| ....|....| ....|....|
PFB0340c     TTAAGGAGAA TACATTAATA CTTAAATGGA AAGTATACGG AGAAACAAAA GATACTACTG AAAATAACAA AGTTGATGTA
3D7          .......... .......... .......... .......... .......... .......... .......... ..........
RO33         .......... .......... .......... .......... .......... .......... .......... ..........
Palo Alto    .......... .......... .......... .......... .......... .......... .......... ..........
FCR3         .......... .......... A......... .......... .......... .......... .......... ..........
Wellcome     .......... .......... A......... .......... .......... .......... .......... ..........
D6           .......... .......... .......... .......... .......... .......... .......... ..........
T996         .......... .......... .......... .......... .......... .......... .......... ..........
T9102        .......... .......... A......... .......... .......... .......... .......... ..........
K1           .......... .......... .......... .......... .......... .......... .......... ..........
Dd2          .......... .......... A......... .......... .......... .......... .......... ..........
D10          .......... .......... A......... .......... .......... .......... .......... ..........
FCC2         .......... .......... A......... .......... .......... .......... .......... ..........
HB3          .......... .......... A......... .......... .......... .......... .......... ..........
7G8          .......... .......... A......... .......... .......... .......... .......... ..........
P.reichenowi .......... .......... .......... .......T.A ..G.C....C .......G.. .......... ..........

                  1210       1220       1230       1240       1250       1260
             ....|....| ....|....| ....|....| ....|....| ....|....| ....|....| ....|....
PFB0340c     AGAAAGTATT TGATAAATGA AAAGGAAACC CCATTTACTA ATATACTAAT ACATGCGTAT AAAGAACAT
3D7          .......... .......... .......... .......... .......... .......... .........
RO33         .......... .......... .......... .......... .......... .......... .........
Palo Alto    .......... .......... .......... .......... .......... .......... .........
FCR3         .......... .......... .......... .......... G......... .......... .........
Wellcome     .......... .......... .......... .......... G......... .......... .........
D6           .......... .......... .......... .......... .......... .......... .........
T996         .......... .......... .......... .......... .......... .......... .........
T9102        .......... .......... .......... .......... .......... ...C...... .........
K1           .......... .......... .......... .......... .......... .......... .........
Dd2          .......... .......... .......... .......... .......... .......... .........
D10          .......... .......... .......... .......... .......... .......... .........
FCC2         .......... .......... .......... .......... .......... .......... .........
HB3          .......... .......... .......... .......... G......... .......... .........
7G8          .......... .......... .......... .......... G......... .......... .........
P.reichenowi .......... .......... .......... .......... G.......G. .......... .........
```
