# Supplementary material for: Prospective Identification of Malaria Parasite Genes under Balancing Selection
Source: PLoS One. 2009 May 15;4(5):e5568. doi: 10.1371/journal.pone.0005568 (PMC2679211; doi:10.1371/journal.pone.0005568)
Supplement: Figure S3 — (0.27 MB PDF) [file pone.0005568.s003.pdf]

Supplementary Figure 3
RAMA – MAL7P1.208

```
                                  10         20         30         40         50         60         70         80
                          ....|....| ....|....| ....|....| ....|....| ....|....| ....|....| ....|....| ....|....|
MAL7P1.208                ATGAATGTTC TACTTCTGTC TTTGCTTGTT GTACAAAATA TAGTAACATA TTTAGAACAA ATAAAAAATG GTATAAGCGG
3D7
RO33
Palo Alto
FCR3
Wellcome
D6
T996
T9102
K1
Dd2
D10
FCC2
HB3
7G8
P.reichenowi

                                  90        100        110        120        130        140        150        160
                          ....|....| ....|....| ....|....| ....|....| ....|....| ....|....| ....|....| ....|....|
MAL7P1.208                TCATTATACA GAAGATCATA ATATTAAGAA TAATAATTGT ATTAGCTTTT CTGATTATGA GAGATCAATA AAAAACTTTT
3D7                                                              ... .......... .......... .......... ..........
RO33                                                             ... .......... .......... .......... ..........
Palo Alto                                                        ... .......... .......... .......... ..........
FCR3                                                             ... .......... .......... .......... ..........
Wellcome                                                         ... .......... .......... .......... ..........
D6                                                               ... .......... .......... .......... ..........
T996                                                             ... .......... .......... .......... ..........
T9102                                                            ... .......... .......... .......... ..........
K1                                                               ... .......... .......... .......... ..........
Dd2                                                              ... .......... .......... .......... ..........
D10                                                              ... .......... .......... .......... ..........
FCC2                                                             ... .......... .......... .......... ..........
HB3                                                              ... .......... .......... .......... ..........
7G8                                                              ... .......... .......... .......... ..........
P.reichenowi

                                 170        180        190        200        210        220        230        240
                          ....|....| ....|....| ....|....| ....|....| ....|....| ....|....| ....|....| ....|....|
MAL7P1.208                CTATTTCTTC TCATGCAGAA AATAATTATG ATAATATAAT AAATGAATAT AAAAAAATAA AAGATATTAA CAACAATATA
3D7                       .......... .......... .......... .......... .......... .......... .......... ..........
RO33                      .......... .......... .......... .......... .......... .......... .......... ..........
Palo Alto                 .......... .......... .......... .......... .......... .......... .......... ..........
FCR3                      .......... .......... .......... .......... .......... .......... .......... ..........
Wellcome                  .......... .......... .......... .......... .......... .......... .......... ..........
D6                        .......... .......... .......... .......... .......... .......... .......... ..........
T996                      .......... .......... .......... .......... .......... .......... .......... ..........
T9102                     .......... .......... .......... .......... .......... .......... .......... ..........
K1                        .......... .......... .......... .......... .......... .......... .......... ..........
Dd2                       .......... .......... .......... .......... .......... .......... .......... ..........
D10                       .......... .......... .......... .......... .......... .......... .......... ..........
FCC2                      .......... .......... .......... .......... .......... .......... .......... ..........
HB3                       .......... .......... .......... .......... .......... .......... .......... ..........
7G8                       .......... .......... .......... .......... .......... .......... .......... ..........
P.reichenowi

                                 250        260        270        280        290        300        310        320
                          ....|....| ....|....| ....|....| ....|....| ....|....| ....|....| ....|....| ....|....|
MAL7P1.208                AACATATTAT CATCAGTACA TAGAAAAGGA AGAATATTGT ACGACAGCTT TTTAGAAATA AATAAGTTGG AAAATGACAA
3D7                       .......... .......... .......... .......... .......... .......... .......... ..........
RO33                      .......... .......... .......... .......... .......... .......... .......... ..........
Palo Alto                 .......... .......... .......... .......... .......... .......... .......... ..........
FCR3                      .......... .......... .......... .......... .......... .......... .......... ..........
Wellcome                  .......... .......... .......... .......... .......... .......... .......... ..........
D6                        .......... .......... .......... .......... .......... .......... .......... ..........
T996                      .......... .......... .......... .......... .......... .......... .......... ..........
T9102                     .......... .......... .......... .......... .......... .......... .......... ..........
K1                        .......... .......... .......... .......... .......... .......... .......... ..........
Dd2                       .......... .......... .......... .......... .......... .......... .......... ..........
D10                       .......... .......... .......... .......... .......... .......... .......... ..........
FCC2                      .......... .......... .......... .......... .......... .......... .......... ..........
HB3                       .......... .......... .......... .......... .......... .......... .......... ..........
7G8                       .......... .......... .......... .......... .......... .......... .......... ..........
P.reichenowi                                             ... .......... ......A... .......... .....C.... .......A..
```

```
                              330        340        350        360        370        380        390        400
                     ....|....| ....|....| ....|....| ....|....| ....|....| ....|....| ....|....| ....|....|
MAL7P1.208           AAAAGAGAAA CATGAAAAAG AAGATGAATA TGAA------ GATAATGATG AAAGCTTTTT AGAAACTGAA GAATATGAAG
3D7                  .......... .......... .......... ....------ .......... .......... .......... ..........
RO33                 .......... .......... .......... ....------ .......... .......... .......... ..........
Palo Alto            .......... .......... .......... ....------ .......... .......... .......... ..........
FCR3                 .......... .......... .......... ....------ .......... .......... .......... ..........
Wellcome             .......... .......... .......... ....------ .......... .......... .......... ..........
D6                   .......... .......... .......... ....------ .......... .......... .......... ..........
T996                 .......... .......... .......... ....------ .......... .......... .......... ..........
T9102                .......... .......... .......... ....------ .......... .......... .......... ..........
K1                   .......... .......... .......... ....------ .......... .......... .......... ..........
Dd2                  .......... .......... .......... ....------ .......... .......... .......... ..........
D10                  .......... .......... .......... ....------ .......... .......... .......... ..........
FCC2                 .......... .......... .......... ....------ .......... .......... .......... ..........
HB3                  .......... .......... .......... ....------ .......... .......... .......... ..........
7G8                  .......... .......... .......... ....------ .......... .......... .......... ..........
P.reichenowi         .........T G....G.... .......... ....TATGAA .......... .......... .......... ..........

                              410        420        430        440        450        460        470        480
                     ....|....| ....|....| ....|....| ....|....| ....|....| ....|....| ....|....| ....|....|
MAL7P1.208           ATAATGAAGA TGAAAAATAT AACAAAGATG AAGATGATTA TGCAGAAAGT TTTATTGAGA CTGATGAATA TGAAGATAAT
3D7                  .......... .......... .......... .......... .......... .......... .......... ..........
RO33                 .......... .......... .......... .......... .......... .......... .......... ..........
Palo Alto            .......... .......... .......... .......... .......... .......... .......... ..........
FCR3                 .......... .......... .......... .......... .......... .......... .......... ..........
Wellcome             .......... .......... .......... .......... .......... .......... .......... ..........
D6                   .......... .......... .......... .......... .......... .......... .......... ..........
T996                 .......... .......... .......... .......... .......... .......... .......... ..........
T9102                .......... .......... .......... .......... .......... .......... .......... ..........
K1                   .......... .......... .......... .......... .......... .......... .......... ..........
Dd2                  .......... .......... .......... .......... .......... .......... .......... ..........
D10                  .......... .......... .......... .......... .......... .......... .......... ..........
FCC2                 .......... .......... .......... .......... .......... .......... .......... ..........
HB3                  .......... .......... .......... .......... .......... .......... .......... ..........
7G8                  .......... .......... .......... .......... .......... .......... .......... ..........
P.reichenowi         .......... .......... ..T..T.... .......... .......... .......... .....A.... ..........

                              490        500        510        520        530        540        550        560
                     ....|....| ....|....| ....|....| ....|....| ....|....| ....|....| ....|....| ....|....|
MAL7P1.208           GAAGATGAT- ---------- ---------- ---------- -----AAATA TAATAAAGAT GAAGATGATT ATTCAGAAAG
3D7                  .........- ---------- ---------- ---------- -----..... .......... .......... ..........
RO33                 .........- ---------- ---------- ---------- -----..... .......... .......... ..........
Palo Alto            .........- ---------- ---------- ---------- -----..... .......... .......... ..........
FCR3                 .........- ---------- ---------- ---------- -----..... .......... .......... ..........
Wellcome             .........- ---------- ---------- ---------- -----..... .......... .......... ..........
D6                   .........- ---------- ---------- ---------- -----..... .......... .......... ..........
T996                 .........- ---------- ---------- ---------- -----..... .......... .......... ..........
T9102                .........- ---------- ---------- ---------- -----..... .......... .......... ..........
K1                   .........- ---------- ---------- ---------- -----..... .......... .......... ..........
Dd2                  .........- ---------- ---------- ---------- -----..... .......... .......... ..........
D10                  .........- ---------- ---------- ---------- -----..... .......... .......... ..........
FCC2                 .........- ---------- ---------- ---------- -----..... .......... .......... ..........
HB3                  .........- ---------- ---------- ---------- -----..... .......... .......... ..........
7G8                  .........- ---------- ---------- ---------- -----..... .......... .......... ..........
P.reichenowi         ........AA AATATAATAA AGATGAAAAA TATAATAAAG ATGAA..... .......... .......... ..G.......

                              570        580        590        600        610        620        630        640
                     ....|....| ....|....| ....|....| ....|....| ....|....| ....|....| ....|....| ....|....|
MAL7P1.208           CTTTATTGAG ACTGATGAAT ATGATGAT-- ---------- ---------- ---------- ---------- ----------
3D7                  .......... .......... ........-- ---------- ---------- ---------- ---------- ----------
RO33                 .......... .......... ........-- ---------- ---------- ---------- ---------- ----------
Palo Alto            .......... .......... ........-- ---------- ---------- ---------- ---------- ----------
FCR3                 .......... .......... ........-- ---------- ---------- ---------- ---------- ----------
Wellcome             .......... .......... ........-- ---------- ---------- ---------- ---------- ----------
D6                   .......... .......... ........-- ---------- ---------- ---------- ---------- ----------
T996                 .......... .......... ........-- ---------- ---------- ---------- ---------- ----------
T9102                .......... .......... ........-- ---------- ---------- ---------- ---------- ----------
K1                   .......... .......... ........-- ---------- ---------- ---------- ---------- ----------
Dd2                  .......... .......... ........-- ---------- ---------- ---------- ---------- ----------
D10                  .......... .......... ........-- ---------- ---------- ---------- ---------- ----------
FCC2                 .......... .......... ........-- ---------- ---------- ---------- ---------- ----------
HB3                  .......... .......... ........-- ---------- ---------- ---------- ---------- ----------
7G8                  .......... .......... ........-- ---------- ---------- ---------- ---------- ----------
P.reichenowi         T......... .......... ....A...AA TAAAGATGAT AAATATAATA AAGATGAAAA ATATAATAAA GATGAAGATG
```

```
                                        650        660        670        680        690        700        710        720
                               ....|....| ....|....| ....|....| ....|....| ....|....| ....|....| ....|....| ....|....|
MAL7P1.208                     ---------- ---------- ---------- ---------- -AATGAAGAA GAACAATATA ATAAAGATGA AGATGATTAT
3D7                            ---------- ---------- ---------- ---------- -......... .......... .......... ..........
RO33                           ---------- ---------- ---------- ---------- -......... .......... .......... ..........
Palo Alto                      ---------- ---------- ---------- ---------- -......... .......... .......... ..........
FCR3                           ---------- ---------- ---------- ---------- -......... .......... .......... ..........
Wellcome                       ---------- ---------- ---------- ---------- -......... .......... .......... ..........
D6                             ---------- ---------- ---------- ---------- -......... .......... .......... ..........
T996                           ---------- ---------- ---------- ---------- -......... .......... .......... ..........
T9102                          ---------- ---------- ---------- ---------- -......... .......... .......... ..........
K1                             ---------- ---------- ---------- ---------- -......... .......... .......... ..........
Dd2                            ---------- ---------- ---------- ---------- -......... .......... .......... ..........
D10                            ---------- ---------- ---------- ---------- -......... .......... .......... ..........
FCC2                           ---------- ---------- ---------- ---------- -......... .......... .......... ..........
HB3                            ---------- ---------- ---------- ---------- -......... .......... .......... ..........
7G8                            ---------- ---------- ---------- ---------- -......... .......... .......... ..........
P.reichenowi                   ATTATGCAGA AAGTTTTATT GAGACTGATG AATATGATGA T......... .......... .......... .....G....

                                        730        740        750        760        770        780        790        800
                               ....|....| ....|....| ....|....| ....|....| ....|....| ....|....| ....|....| ....|....|
MAL7P1.208                     ACAGATAGTT TTATTGAGAC AGACCATTAT GAAAATAACG ATGATAAAAA TGAAGAAGAA GAAGAA---T ATAATGATCA
3D7                            .......... .......... .......... .......... .......... .......... ......---. ..........
RO33                           .......... .......... .......... .......... .......... .......... ......---. ..........
Palo Alto                      G......... .......... .......... .......... .......... .......... ......---. ..........
FCR3                           G......... .......... .......... .......... .......... .......... ......---. ..........
Wellcome                       G......... .......... .......... .......... .......... .......... ......---. ..........
D6                             G......... .......... .......... .......... .......... .......... ......---. ..........
T996                           G......... .......... .......... .......... .......... .......... ......---. ..........
T9102                          G......... .......... .......... .......... .......... .......... ......---. ..........
K1                             G......... .......... .......... .......... .......... .......... ......---. ..........
Dd2                            G......... .......... .......... .......... .......... .......... ......---. ..........
D10                            G......... .......... .......... .......... .......... .......... ......---. ..........
FCC2                           G......... .......... .......... .......... .......... .......... ......---. ..........
HB3                            G......... .......... .......... .......... .......... .......... ......---. ..........
7G8                            G......... .......... .......... .......... .......... .......... ......---. ..........
P.reichenowi                   G......... .......... .......... .G...C.... .......... .......... ......GAA. ..........

                                        810        820        830        840        850        860        870        880
                               ....|....| ....|....| ....|....| ....|....| ....|....| ....|....| ....|....| ....|....|
MAL7P1.208                     AGATAATGAT TATGGATATA ACTTTTTAGA AACTGACGAA TACGATGATA GCGAAGAATA TGATTACGAC GATAAGGAAT
3D7                            .......... .......... .......... .......... .......... .......... .......... ..........
RO33                           .......... .......... .......... .......... .......... .......... .......... ..........
Palo Alto                      .......... .......... .......... .......... .......... .......... .......... ..........
FCR3                           .......... .......... .......... .......... .......... .......... .......... ..........
Wellcome                       .......... .......... .......... .......... .......... .......... .......... ..........
D6                             .......... .......... .......... .......... .......... .......... .......... ..........
T996                           .......... .......... .......... .......... .......... .......... .......... ..........
T9102                          .......... .......... .......... .......... .......... .......... .......... ..........
K1                             .......... .......... .......... .......... .......... .......... .......... ..........
Dd2                            .......... .......... .......... .......... .......... .......... .......... ..........
D10                            .......... .......... .......... .......... .......... .......... .......... ..........
FCC2                           .......... .......... .......... .......... .......... .......... .......... ..........
HB3                            .......... .......... .......... .......... .......... .......... .......... ..........
7G8                            .......... .......... .......... .......... .......... .......... .......... ..........
P.reichenowi                   .......... .......... .......... .......... ..T....... .......... .......... .....C....

                                        890        900        910        920        930        940        950        960
                               ....|....| ....|....| ....|....| ....|....| ....|....| ....|....| ....|....| ....|....|
MAL7P1.208                     ACGGAGAGAG TTTCCTCGAA AAAGAAGAAG GTGAAGAAAT GAAAGATGAA GAGATGAAAG ATGAAGAAAT GAAAGATGTA
3D7                            .......... .......... .......... .......... .......... .......... .......... ..........
RO33                           .......... .......... .......... .......... .......... .......... .......... ..........
Palo Alto                      .......... .......... .......... .......... .......... .......... .......... ..........
FCR3                           .......... .......... .......... .......... .......... .......... .......... .G........
Wellcome                       .......... .......... .......... .......... .......... .......... .......... .G........
D6                             .......... .......... .......... .......... .......... .......... .......... ..........
T996                           .......... .......... .......... .......... .......... .......... .......... ..........
T9102                          .......... .......... .......... .......... .......... .......... .......... .G........
K1                             .......... .......... .......... .......... .......... .......... .......... ..........
Dd2                            .......... .......... .......... .......... .......... .......... .......... .G........
D10                            .......... .......... .......... .......... .......... .......... .......... .G........
FCC2                           .......... .......... .......... .......... .......... .......... .......... .G........
HB3                            .......... .......... .......... .......... .......... .......... .......... ..........
7G8                            .......... .......... .......... .......... .......... .......... .......... ..........
P.reichenowi                   .......... .......... .......... .......... .......... .........T ....C..G.. ........AT
```

```
                          970       980       990      1000      1010      1020      1030      1040
               ....|....| ....|....| ....|....| ....|....| ....|....| ....|....| ....|....| ....|....|
MAL7P1.208     GAAATGAAAG ATGAAGAGAT GAAAGATGAA GAGATGAAAT ATGACGAGAT GAAAAATGAA GAGATGAAAT ATGACGAGAT
3D7            .......... .......... .......... .......... .......... .......... .......... ..........
RO33           .......... .......... .......... .......... .......... .......... .......... ..........
Palo Alto      .......... .......... .......... .......... .......... .......... .......... ..........
FCR3           .......... .......... .......... .......... .......... .......... .......... ..........
Wellcome       .......... .......... .......... .......... .......... .......... .......... ..........
D6             .......... .......... .......... .......... .......... .......... .......... ..........
T996           .......... .......... .......... .......... .......... .......... .......... ..........
T9102          .......... .......... .......... .......... .......... .......... .......... ..........
K1             .......... .......... .......... .......... .......... .......... .......... ..........
Dd2            .......... .......... .......... .......... .......... .......... .......... ..........
D10            .......... .......... .......... .......... .......... .......... .......... ..........
FCC2           .......... .......... .......... .......... .......... .......... .......... ..........
HB3            .......... .......... .......... .....A.... .......... .......... .......... ..........
7G8            .......... .......... .......... .......... .......... .......... .......... ..........
P.reichenowi   C.......TT ....C..... .......... ..A..C...G ....T..A.. ....T....C .........G ....A.....

                         1050      1060      1070      1080      1090      1100      1110      1120
               ....|....| ....|....| ....|....| ....|....| ....|....| ....|....| ....|....| ....|....|
MAL7P1.208     GAAAGATGAA GTGATGAAAG ATGAAGAGAT GAAAGATGAA GTGATGAAAG ATGAAGAGAT GAAAGACGAA ----------
3D7            .......... .......... .......... .......... .......... .......... .......... ----------
RO33           .......... .......... .......... .......... .A........ ......T... ......T... GAGATGAAAG
Palo Alto      .......... .A.......T ....C..... .......... .......... .......... .......... ----------
FCR3           .......... .......... .......... ......C... ---------- ---------- ---------- ----------
Wellcome       .......... .......... .......... ......C... ---------- ---------- ---------- ----------
D6             .......... .A.......T ....C..... .......... .......... .......... .......... ----------
T996           .......... .......... ......T... .......... .A........ .C...----- ---------- ----------
T9102          .......... .......... .......... ......C... ---------- ---------- ---------- ----------
K1             .......... .......... .......... ......C... ---------- ---------- ---------- ----------
Dd2            .......... .......... .......... ......C... ---------- ---------- ---------- ----------
D10            .......... .......... .......... ......C... ---------- ---------- ---------- ----------
FCC2           .......... .......... .......... ......C... ---------- ---------- ---------- ----------
HB3            .......... .......... .......... .......... .......... .......... .......... ----------
7G8            .......... .......... .......... .......... .A........ .C...----- ---------- ----------
P.reichenowi   ....T....C .A........ .......... ....T....C .AT....... ....C..... ....A.T..C GAGATGAAAT

                         1130      1140      1150      1160      1170      1180      1190      1200
               ....|....| ....|....| ....|....| ....|....| ....|....| ....|....| ....|....| ....|....|
MAL7P1.208     ---------- ---------- CAAATGAAAT ATGAAGAATT CAAAAATGAA GAATCCAAAA ATGAAGAATC CAAAAATGAA
3D7            ---------- ---------- .......... .......... .......... .......... .......... ..........
RO33           ACGAA----- ---------- .......... .......... .......... .......... .......... ..........
Palo Alto      ---------- ---------- .......... .......... .......... .......... .......... ..........
FCR3           ---------- ---------- .......... .......... .......... ....T..... .........T ..........
Wellcome       ---------- ---------- .......... .......... .......... ....T..... .........T ..........
D6             ---------- ---------- .......... .......... .......... .......... .......... ..........
T996           ---------- ---------- .......... .......... .......... ....T..... .......... ..........
T9102          ---------- ---------- .......... .......... .......... ....T..... .........T ..........
K1             ---------- ---------- .......... .......... .......... ....T..... .........T ..........
Dd2            ---------- ---------- .......... .......... .......... ....T..... .........T ..........
D10            ---------- ---------- .......... .......... .......... ....T..... .........T ..........
FCC2           ---------- ---------- .......... .......... .......... ....T..... .........T ..........
HB3            ---------- ---------- .......... .......... .......... .......... .......... ..........
7G8            ---------- ---------- .......... .......... .......... ....T..... .........T ..........
P.reichenowi   ATGACGAGAT GAAAGACGAA ..G...G... .......... .--------- ---------- ---------- ----------

                         1210      1220      1230      1240      1250      1260      1270      1280
               ....|....| ....|....| ....|....| ....|....| ....|....| ....|....| ....|....| ....|....|
MAL7P1.208     GAATCCAAAA ATGAAGAATC CAAAAATGAA GAATTCAAAA ATGAAGAATC CAAAAATGAA GAATTTAAAA ATGAAGAATT
3D7            .......... .......... .......... .......... .......... .......... .......... ..........
RO33           .......... .........T .......... ....C..... .........T T......... .....C---- ----------
Palo Alto      .......... .......... .......... .......... .......... .......... .......... ..........
FCR3           .......... .......... .......... ....C..... .......... .......... .....C.... .........C
Wellcome       .......... .......... .......... ....C..... .......... .......... .....C.... .........C
D6             .......... .......... .......... .......... .......... .......... .......... ..........
T996           .......... .......... .......... ....C..... .........T .......... ....CC.... ..........
T9102          .......... .......... .......... ....C..... .......... .......... .....C.... .........C
K1             .......... .......... .......... ....C..... .......... .......... .....C.... .........C
Dd2            .......... .......... .......... ....C..... .......... .......... .....C.... .........C
D10            .......... .......... .......... ....C..... .......... .......... .....C.... .........C
FCC2           .......... .......... .......... ....C..... .......... .......... .....C.... .........C
HB3            .......... .......... .......... .......... .......... .......... .......... ..........
7G8            .......... .......... .......... ....C..... .......... .......... .....C.... .........C
P.reichenowi   ---------- ---------- ---------- ---------- ---------- ---------- ---------- ----------
```

```
                        1290       1300       1310       1320       1330       1340       1350       1360
                ....|....| ....|....| ....|....| ....|....| ....|....| ....|....| ....|....| ....|....|
MAL7P1.208      C--------- ---------- ---------- -AAAAATGAA GAT------- --------AT GTCTTATGAT GAATACATGG
3D7             .--------- ---------- ---------- -......... ...------- --------.. .......... ..........
RO33            ---------- ---------- ---------- -......... ...------- --------.. .......... ..........
Palo Alto       .--------- ---------- ---------- -......... ...------- --------.. .......... ..........
FCR3            .AAAAATGAA GAATTTAAAA ATGAAGAATT C......... ...------- --------.. .......... ..........
Wellcome        .AAAAATGAA GAATTTAAAA ATGAAGAATT C......... ...------- --------.. .......... ..........
D6              .--------- ---------- ---------- -......... ...------- --------.. .......... ..........
T996            TAAAAATGAA GAATTC---- ---------- ---------- ---------- ---------- ---------- ----------
T9102           .AAAAATGAA GAATTTAAAA ATGAAGAATT C......... ...------- --------.. .......... ..........
K1              .AAAAATGAA GAATTTAAAA ATGAAGAATT C......... ...------- --------.. .......... ..........
Dd2             .AAAAATGAA GAATTTAAAA ATGAAGAATT C......... ...------- --------.. .......... ..........
D10             .AAAAATGAA GAATTTAAAA ATGAAGAATT C......... ...------- --------.. .......... ..........
FCC2            .AAAAATGAA GAATTTAAAA ATGAAGAATT C......... ...------- --------.. .......... ..........
HB3             .--------- ---------- ---------- ---------- ---------- ---------- ---------- ----------
7G8             .AAAAATGAA GAATTTAAAA ATGAAGAATT C......... ...------- --------.. .......... ..........
P.reichenowi    ---------- ---------- ---------- -......... ...AAAAAGA AGGAAGAT.. .......... ..........

                        1370       1380       1390       1400       1410       1420       1430       1440
                ....|....| ....|....| ....|....| ....|....| ....|....| ....|....| ....|....| ....|....|
MAL7P1.208      GATATAAAAA GAAAGAAGAA GATGAATCAT ATAATACATT CAATGGTACT AAGAAAAATA ACACATCCAA CAGCTTCCTC
3D7             .......... .......... .......... .......... .......... .......... .......... ..........
RO33            .......... .......C.. .......... .......... .......... .......... .......... ..........
Palo Alto       .......... .......... .......... .......... .......... .......... .......... ..........
FCR3            .......... .......... .......... .......... .......... .......... .......... ..........
Wellcome        .......... .......... .......... .......... .......... .......... .......... ..........
D6              .......... .......... .......... .......... .......... .......... .......... ..........
T996            ---------- ---------- ---------- ---------- ---------- ---------- ---------- ----------
T9102           .......... .......... .......... .......... .......... .......... .......... ..........
K1              .......... .......C.. .......... .......... .......... .......... .......... ..........
Dd2             .......... .......... .......... .......... .......... .......... .......... ..........
D10             .......... .......C.. .......... .......... .......... .......... .......... ..........
FCC2            .......... .......C.. .......... .......... .......... .......... .......... ..........
HB3             ---------- ---------- ---------- ---------- ---------- ---------- ---------- ----------
7G8             .......... .......C.. .......... .......... .......... .......... .......... ..........
P.reichenowi    .......... .......C.. ..A....... .........A ........A. .......... .A......G. ..........

                        1450       1460       1470       1480       1490       1500       1510       1520
                ....|....| ....|....| ....|....| ....|....| ....|....| ....|....| ....|....| ....|....|
MAL7P1.208      GAAAAAGATT TACAAGGAGA TTCTGACGAT GAATTACATA GTACCTTTTA TTCCAAAAAT GTAGATAAAG AAAATTATGA
3D7             .......... .......... .......... .......... .......... .......... .......... ..........
RO33            .......... .......... .......... .......... .......... .......... .......... ..........
Palo Alto       .......... .......... .......... .......... .......... .......... .......... ..........
FCR3            .......... .......... .......... .......... .......... .......... .......... ..........
Wellcome        .......... .......... .......... .......... .......... .......... .......... ..........
D6              .......... .......... .......... .......... .......... .......... .......... ..........
T996            ---------- ---------- ---------- ---------- ---------- ----...... .......... ..........
T9102           .......... .......... .......... .......... .......... .......... .......... ..........
K1              .......... .......... .......... .......... .......... .......... .......... ..........
Dd2             .......... .......... .......... .......... .......... .......... .......... ..........
D10             .......... .......... .......... .......... .......... .......... .......... ..........
FCC2            .......... .......... .......... .......... .......... .......... .......... ..........
HB3             ---------- ---------- ---------- ---------- ---------- ----...... .......... ..........
7G8             .......... .......... .......... .......... .......... .......... .......... ..........
P.reichenowi    .......... .......... .......... .......... .......... .......... .......... ..........

                        1530       1540       1550       1560       1570       1580       1590       1600
                ....|....| ....|....| ....|....| ....|....| ....|....| ....|....| ....|....| ....|....|
MAL7P1.208      TGATAAAAAT ATTTTCTATG GTTATAGTGA TAATGATGAT GAAAGCTTTT TAGAAACTGA TTCTTATGAA GAATATGAAG
3D7             .......... .......... .......... .......... .......... .......... .......... ..........
RO33            .......... .......... .......... .......... .......... .......... .......... ..........
Palo Alto       .......... .......... .......... .......... .......... .......... .......... ..........
FCR3            .......... .......... .......... .......... .......... .......... .......... ..........
Wellcome        .......... .......... .......... .......... .......... .......... .......... ..........
D6              .......... .......... .......... .......... .......... .......... .......... ..........
T996            .......... .......... .......... .......... .......... .......... .......... ..........
T9102           .......... .......... .......... .......... .......... .......... .......... ..........
K1              .......... .......... .......... .......... .......... .......... .......... ..........
Dd2             .......... .......... .......... .......... .......... .......... .......... ..........
D10             .......... .......... .......... .......... .......... .......... .......... ..........
FCC2            .......... .......... .......... .......... .......... .......... .......... ..........
HB3             .......... .......... .......... .......... .......... .......... .......... ..........
7G8             .......... .......... .......... .......... .......... .......... .......... ..........
P.reichenowi    .A..G..... .......... ......A... .......... .......... .......... .......... ..........
```

```
                              1610       1620       1630       1640       1650       1660       1670       1680
                        ....|....| ....|....| ....|....| ....|....| ....|....| ....|....| ....|....| ....|....|
MAL7P1.208              ACGAAGATAA AGATGTTGAA GATGAGTATG AAGAAAGTTT CTTACAAAAT GATGAGAAAA AAATGGTCTT TTATGATTTA
3D7                     .......... .......... .......... .......... .......... .......... .......... ..........
RO33                    .......... .......... .......... .......... .......... .......... .......... ..........
Palo Alto               .......... .......... .......... .......... .......... .......... .......... ..........
FCR3                    .......... .......... .......... .......... .......... .......... .......... ..........
Wellcome                .......... .......... .......... .......... .......... .......... .......... ..........
D6                      .......... .......... .......... .......... .......... .......... .......... ..........
T996                    .......... .......... .......... .......... .......... .......... .......... ..........
T9102                   .......... .......... .......... .......... .......... .......... .......... ..........
K1                      .......... .......... .......... .......... .......... .......... .......... ..........
Dd2                     .......... .......... .......... .......... .......... .......... .......... ..........
D10                     .......... .......... .......... .......... .......... .......... .......... ..........
FCC2                    .......... .......... .......... .......... .......... .......... .......... ..........
HB3                     .......... .......... .......... .......... .......... .......... .......... ..........
7G8                     .......... .......... .......... .......... .......... .......... .......... ..........
P.reichenowi            .......... .....C.... .......... .......... .......... .......... .......... ..........

                              1690       1700       1710       1720       1730       1740       1750       1760
                        ....|....| ....|....| ....|....| ....|....| ....|....| ....|....| ....|....| ....|....|
MAL7P1.208              TACAAGCCAG AAGAAAATGA ATCTTATTAT GAAAAGAAAC AAAAGAAAGA AGAAAAAGAA GAGAAAGAAG AGAAAGAACA
3D7                     .......... .......... .......... .......... .......... .......... .......... ..........
RO33                    .......... .......... .......... .......... .......... .......... .......... ..........
Palo Alto               .......... .......... .......... .......... .......... .......... .......... ..........
FCR3                    .......... .......... .......... .......... .......... .......... .......... ..........
Wellcome                .......... .......... .......... .......... .......... .......... .......... ..........
D6                      .......... .......... .......... .......... .......... .......... .......... ..........
T996                    .......... .......... .......... .......... .......... .......... .......... ..........
T9102                   .......... .......... .......... .......... .......... .......... .......... ..........
K1                      .......... .......... .......... .......... .......... .......... .......... ..........
Dd2                     .......... .......... .......... .......... .......... .......... .......... ..........
D10                     .......... .......... .......... .......... .......... .......... .......... ..........
FCC2                    .......... .......... .......... .......... .......... .......... .......... ..........
HB3                     .......... .......... .......... .......... .......... .......... .......... ..........
7G8                     .......... .......... .......... .......... .......... .......... .......... ..........
P.reichenowi            .....T.... .......... .......... .......... ....A..... .......... ..A......- --------..

                              1770       1780       1790       1800       1810       1820       1830       1840
                        ....|....| ....|....| ....|....| ....|....| ....|....| ....|....| ....|....| ....|....|
MAL7P1.208              AAGTTTGAAC AAACAAAACG ATATGGAAGA CCAAGAAGAT AATGAAGAAT ATAAATTTGA AGAAGAAAAT AAAGAAGACC
3D7                     .......... .......... .......... .......... .......... .......... .......... ..........
RO33                    .......... .......... .......... .......... .......... .......... .......... ..........
Palo Alto               .......... .......... .......... .......... .......... .......... .......... ..........
FCR3                    .......... .......... .......... .......... .......... .......... .......... ..........
Wellcome                .......... .......... .......... .......... .......... .......... .......... ..........
D6                      .......... .......... .......... .......... .......... .......... .......... ..........
T996                    .......... .......... .......... .......... .......... .......... .......... ..........
T9102                   .......... .......... .......... .......... .......... .......... .......... ..........
K1                      .......... .......... .......... .......... .......... .......... .......... ..........
Dd2                     .......... .......... .......... .......... .......... .......... .......... ..........
D10                     .......... .......... .......... .......... .......... .......... .......... ..........
FCC2                    .......... .......... .......... .......... .......... .......... .......... ..........
HB3                     .......... .......... .......... .......... .......... .......... .......... ..........
7G8                     .......... .......... .......... .......... .......... .......... .......... ..........
P.reichenowi            .TA....... .......... .......... .......... .......... .......... .......... ..........

                              1850       1860       1870       1880       1890       1900       1910       1920
                        ....|....| ....|....| ....|....| ....|....| ....|....| ....|....| ....|....| ....|....|
MAL7P1.208              TTCTAGATGT CCAACAAGAT GAAGAATTAC CAAGTGAAGG AAAACAAAAA GTAAAAGGAA AATCATTCGA TAATGAACAT
3D7                     .......... .......... .......... .......... .......... .......... .......... ..........
RO33                    .......... .......... .......... .......... .......... .......... .......... ..........
Palo Alto               .......... .......... .......... .......... .......... .......... .......... ..........
FCR3                    .......... .......... .......... .......... .......... .......... .......... ..........
Wellcome                .......... .......... .......... .......... .......... .......... .......... ..........
D6                      .......... .......... .......... .......... .......... .......... .......... ..........
T996                    .......... .......... .......... .......... .......... .......... .......... ..........
T9102                   .......... .......... .......... .......... .......... .......... .......... ..........
K1                      .......... .......... .......... .......... .......... .......... .......... ..........
Dd2                     .......... .......... .......... .......... .......... .......... .......... ..........
D10                     .......... .......... .......... .......... .......... .......... .......... ..........
FCC2                    .......... .......... .......... .......... .......... .......... .......... ..........
HB3                     .......... .......... .......... .......... .......... .......... .......... ..........
7G8                     .......... .......... .......... .......... .......... .......... .......... ..........
P.reichenowi            .......... .......... .......... .......... .......... .G........ .......... ..........
```

```
                              1930       1940       1950       1960       1970       1980       1990       2000
                      ....|....| ....|....| ....|....| ....|....| ....|....| ....|....| ....|....| ....|....|
MAL7P1.208            TTGAATGAAA TACAAAATGT TAGCGACGTA CATGCATTTA TACAAAAAGA TATGAAATAT TTAGATGATC TCATAGATGA
3D7                   .......... .......... .......... .......... .......... .......... .......... ..........
RO33                  .......... .......... .......... .......... .......... .......... .......... ..........
Palo Alto             .......... .......... .......... .......... .......... .......... .......... ..........
FCR3                  .......... .......... .......... .......... .......... .......... .......... ..........
Wellcome              .......... .......... .......... .......... .......... .......... .......... ..........
D6                    .......... .......... .......... .......... .......... .......... .......... ..........
T996                  .......... .......... .......... .......... .......... .......... .......... ..........
T9102                 .......... .......... .......... .......... .......... .......... .......... ..........
K1                    .......... .......... .......... .......... .......... .......... .......... ..........
Dd2                   .......... .......... .......... .......... .......... .......... .......... ..........
D10                   .......... .......... .......... .......... .......... .......... .......... ..........
FCC2                  .......... .......... .......... .......... .......... .......... .......... ..........
HB3                   .......... .......... .......... .......... .......... .......... .......... ..........
7G8                   .......... .......... .......... .......... .......... .......... .......... ..........
P.reichenowi          .......... .......... ....A.T... .......... .......... .......... .......... ..........

                              2010       2020       2030       2040       2050       2060       2070       2080
                      ....|....| ....|....| ....|....| ....|....| ....|....| ....|....| ....|....| ....|....|
MAL7P1.208            AGAGCAAACT ATTAAAGATG CCGTCAAAAA AAGTGCTTAT AAAGGAAATA AGAAATTAGG AAATAATAAA AAATCACAAA
3D7                   .......... .......... .......... .......... .......... .......... .......... ..........
RO33                  .......... .......... .......... .......... .......... .......... .......... ..........
Palo Alto             .......... .......... .......... .......... .......... .......... .......... ..........
FCR3                  .......... .......... .......... .......... .......... .......... .......... ..........
Wellcome              .......... .......... .......... .......... .......... .......... .......... ..........
D6                    .......... .......... .......... .......... .......... .......... .......... ..........
T996                  .......... .......... .......... .......... .......... .......... .......... ..........
T9102                 .......... .......... .......... .......... .......... .......... .......... ..........
K1                    .......... .......... .......... .......... .......... .......... .......... ..........
Dd2                   .......... .......... .......... .......... .......... .......... .......... ..........
D10                   .......... .......... .......... .......... .......... .......... .......... ..........
FCC2                  .......... .......... .......... .......... .......... .......... .......... ..........
HB3                   .......... .......... .......... .......... .......... .......... .......... ..........
7G8                   .......... .......... .......... .......... .......... .......... .......... ..........
P.reichenowi          ...A...... ........A. .......... .......... ....T..... .......... .......... .........G

                              2090       2100       2110       2120       2130       2140       2150       2160
                      ....|....| ....|....| ....|....| ....|....| ....|....| ....|....| ....|....| ....|....|
MAL7P1.208            TGATACTGGA AGAAGAACCA GAAGAAAATT TTGAAGAAGA TGCTGATGAA GAATTAAATA AACTAATGGA ACAAGAAAAA
3D7                   .......... .......... .......... .......... .......... .......... .......... ..........
RO33                  .......... .......... .......... .......... .......... .......... .......... ..........
Palo Alto             .......... .......... .......... .......... .......... .......... .......... ..........
FCR3                  .A........ .......... .......... .......... .......... .......... .......... ..........
Wellcome              .A........ .......... .......... .......... .......... .......... .......... ..........
D6                    .......... .......... .......... .......... .......... .......... .......... ..........
T996                  .......... .......... .......... .......... .......... .......... .......... ..........
T9102                 .A........ .......... .......... .......... .......... .......... .......... ..........
K1                    .A........ .......... .......... .......... .......... .......... .......... ..........
Dd2                   .A........ .......... .......... .......... .......... .......... .......... ..........
D10                   .......... .......... .......... .......... .......... .......... .......... ..........
FCC2                  .......A.. .......... .......... .......... .......... .......... .......... ..........
HB3                   .......... .......... .......... .......... .......... .......... .......... ..........
7G8                   .......... .......... .......... .......... .......... .......... .......... ..........
P.reichenowi          .......A.. .C........ .......... .......... .......... .......... .......... ..........

                              2170       2180       2190       2200       2210       2220       2230       2240
                      ....|....| ....|....| ....|....| ....|....| ....|....| ....|....| ....|....| ....|....|
MAL7P1.208            AATATTGTAG ATAAAGAAAT CAAAAATAGT AAAGCAAATA AAAGCAACAA AAAATTACAA TTCAATAACA CTAATAAACA
3D7                   .......... .......... .......... .......... .......... .......... .......... ..........
RO33                  .......... .......... .......... .......... .......... .......... .......... ..........
Palo Alto             .......... .......... .......... .......... .......... .......... .......... ..........
FCR3                  .......... .......... .......... .......... .......... .......... .......... ..........
Wellcome              .......... .......... .......... .......... .......... .......... .......... ..........
D6                    .......... .......... .......... .......... .......... .......... .......... ..........
T996                  .......... .......... .......... .......... .......... .......... .......... ..........
T9102                 .......... .......... .......... .......... .......... .......... .......... ..........
K1                    .......... .......... .......... .......... .......... .......... .......... ..........
Dd2                   .......... .......... .......... .......... .......... .......... .......... ..........
D10                   .......... .......... .......... .......... .......... .......... .......... ..........
FCC2                  .......... .......... .......... .......... .......... .......... .......... ..........
HB3                   .......... .......... .......... .......... .......... .......... .......... ..........
7G8                   .......... .......... .......... .......... .......... .......... .......... ..........
P.reichenowi          .......... .......... .......... .......... .......... .......... .......... ..........
```

```
                                 2250       2260       2270       2280       2290       2300       2310       2320
                         ....|....| ....|....| ....|....| ....|....| ....|....| ....|....| ....|....| ....|....|
MAL7P1.208               AAACAAAATG TATATGAAAA ACGAATATAA TAATAAGACA AAAAATAATA AAAACAATAA ATTTGAACAA CAAAATTATG
3D7                      .......... .......... .......... .......... .......... .......... .......... ..........
RO33                     .......... .......... .......... .......... .......... .......... .......... ..........
Palo Alto                .......... .......... .......... .......... .......... .......... .......... ..........
FCR3                     .......... .......... .......... .......... .......... .......... .......... ..........
Wellcome                 .......... .......... .......... .......... .......... .......... .......... ..........
D6                       .......... .......... .......... .......... .G........ .......... .......... ..........
T996                     .......... .......... .......... .......... .......... .......... .......... ..........
T9102                    .......... .......... .......... .......... .......... .......... .......... ..........
K1                       .......... .......... .......... .......... .......... .......... .......... ..........
Dd2                      .......... .......... .......... .......... .......... .......... .......... ..........
D10                      .......... .......... .......... .......... .......... .......... .......... ..........
FCC2                     .......... .......... .......... .......... .......... .......... .......... ..........
HB3                      .......... .......... .......... .......... .......... .......... .......... ..........
7G8                      .......... .......... .......... .......... .......... .......... .......... ..........
P.reichenowi             .......... .......... .......... ......C... .......... ....A..... .......... ..C.......

                                 2330       2340       2350       2360       2370       2380       2390       2400
                         ....|....| ....|....| ....|....| ....|....| ....|....| ....|....| ....|....| ....|....|
MAL7P1.208               ATGAATCATA TATGGATGAT GATTATGAAC AAAATGAAGA ATTTAATGAT AATAATCAAA GCGAAGATAT GAAAGAAACA
3D7                      .......... .......... .......... .......... .......... .......... .......... ..........
RO33                     .......... .......... .......... .......... .......... .......... .......... ..........
Palo Alto                .......... .......... .......... .......... .......... .......... .......... ..........
FCR3                     .......... .......... .......... .......... .......... .......... .......... ..........
Wellcome                 .......... .......... .......... .......... .......... .......... .......... ..........
D6                       .......... .......... .......... .......... .......... .......... .......... ..........
T996                     .......... .......... .......... .......... .......... .......... .......... ..........
T9102                    .......... .......... .......... .......... .......... .......... .......... ..........
K1                       .......... .......... .......... .......... .......... .......... .......... ..........
Dd2                      .......... .......... .......... .......... .......... .......... .......... ..........
D10                      .......... .......... .......... .......... .......... .......... .......... ..........
FCC2                     .......... .......... .......... .......... .......... .......... .......... ..........
HB3                      .......... .......... .......... .......... .......... .......... .......... ..........
7G8                      .......... .......... .......... .......... .......... .......... .......... ..........
P.reichenowi             .......... C..T...... .......... .T........ .......... .......... .......... ..........

                                 2410       2420       2430       2440       2450       2460       2470       2480
                         ....|....| ....|....| ....|....| ....|....| ....|....| ....|....| ....|....| ....|....|
MAL7P1.208               AATGAACTCG ATAAAATTAA TGATGAACTA TTAACTGATC AAGGACCAAA CGAAGATACA TTATTAGAAA ATAATAATAA
3D7                      .......... .......... .......... .......... .......... .......... .......... ..........
RO33                     .......... .......... .......... .......... .......... .......... .......... ..........
Palo Alto                .......... .......... .......... .......... .......... .......... .......... ..........
FCR3                     .......... .......... .......... .......... .......... .......... .......... ..........
Wellcome                 .......... .......... .......... .......... .......... .......... .......... ..........
D6                       .......... .......... .......... .......... .......... .......... .......... ..........
T996                     .......... .......... .......... .......... .......... .......... .......... ..........
T9102                    .......... .......... .......... .......... .......... .......... .......... ..........
K1                       .......... .......... .......... .......... .......... .......... .......... ..........
Dd2                      .......... .......... .......... .......... .......... .......... .......... ..........
D10                      .......... .......... .......... .......... .......... .......... .......... ..........
FCC2                     .......... .......... .......... .......... .......... .......... .......... ..........
HB3                      .......... .......... .......... .......... .......... .......... .......... ..........
7G8                      .......... ..G....... .A........ .......... .......... .......... .......... ..........
P.reichenowi             .......... ..G....... .........C .......... .......... .......... .......... ..........

                                 2490       2500       2510       2520       2530       2540       2550       2560
                         ....|....| ....|....| ....|....| ....|....| ....|....| ....|....| ....|....| ....|....|
MAL7P1.208               AATTTTCGAT AATAAATTTG TAGCACATAA AAAAAGAGAA AAAAGTATAT CCCCACACAG TTACCAAAAG GTATCTACCA
3D7                      .......... .......... .......... .......... .......... .......... .......... ..........
RO33                     .......... .......... .......... .......... .......... .......... .......... ..........
Palo Alto                .......... .......... .......... .......... .......... .......... .......... ..........
FCR3                     .......... .......... .......... .......... .......... .......... .......... ..........
Wellcome                 .......... .......... .......... .......... .......... .......... .......... ..........
D6                       .......... .......... .......... .......... .......... .......... .......... ..........
T996                     .......... .......... .......... .......... .......... .......... .......... ..........
T9102                    .......... .......... .......... .......... .......... .......... .......... ..........
K1                       .......... .......... .......... .......... .......... .......... .......... ..........
Dd2                      .......... .......... .......... .......... .......... .......... .......... ..........
D10                      .......... .......... .......... .......... .......... .......... .......... ..........
FCC2                     .......... .......... .......... .......... .......... .......... .......... ..........
HB3                      .......... .......... .......... .......... .......... .......... .......... ..........
7G8                      .......... .......... .......... .......... .......... .......... .......... ..........
P.reichenowi             .......... .......... .......C.. .......... ....C..... .......... ....A..... ........T.
```

```
                                       2570       2580       2590       2600       2610       2620       2630       2640
                               ....|....| ....|....| ....|....| ....|....| ....|....| ....|....| ....|....| ....|....|
MAL7P1.208                     AAGTACAAAA TAAGGAAGAC ATGGAAAATA AGGAAGAGAA ACAATTGATA AATGATGAAG CCGCTATGAC TGCTGAAGAA
3D7                            .......... .......... .......... .......... ..........
RO33                           .......... .......... .......... .......... ..........
Palo Alto                      .......... .......... .......... .......... ..........
FCR3                           .......... .......... .......... .......... ..........
Wellcome                       .......... .......... .......... .......... ..........
D6                             .......... .......... .......... .......... ..........
T996                           .......... .......... .......... .......... ..........
T9102                          .......... .......... .......... .......... ..........
K1                             .......... .......... .......... .......... ..........
Dd2                            .......... .......... .......... .......... ..........
D10                            .......... .......... .......... .......... ..........
FCC2                           .......... .......... .......... .......... ..........
HB3                            .......... .......... .......... .......... ..........
7G8                            .......... .......... .......... .......... ..........
P.reichenowi                   .......... C......... .......... .......... ..........

                                       2650       2660       2670       2680       2690       2700       2710       2720
                               ....|....| ....|....| ....|....| ....|....| ....|....| ....|....| ....|....| ....|....|
MAL7P1.208                     TTAGTCGAAT TAGAAAACAC AGAAGATGTG AATACACCTA CAATGGTAGA AACAGAAGAA ATTGATTCTG ATGAAAATGG
3D7
RO33
Palo Alto
FCR3
Wellcome
D6
T996
T9102
K1
Dd2
D10
FCC2
HB3
7G8
P.reichenowi

                                       2730       2740       2750       2760       2770       2780       2790
                               ....|....| ....|....| ....|....| ....|....| ....|....| ....|....| ....|....| ....|....
MAL7P1.208                     AAATAAGTCA AGCAGTTCAA TATCATACAT TAGTTCTATA GTATTCCTTA TGGTCACCCT TTTATATTTT ATGAATTAA
3D7
RO33
Palo Alto
FCR3
Wellcome
D6
T996
T9102
K1
Dd2
D10
FCC2
HB3
7G8
P.reichenowi
```

PF13_0348 _Rhop148

```
                               10         20         30         40         50         60         70         80
                       ....|....| ....|....| ....|....| ....|....| ....|....| ....|....| ....|....| ....|....|
PF13_0348              ATGTATGATA TATCTAGTGA ACAAATTAAT ATACAAAATA AGTTTTTAAA AAATTTAGAT TTATATTGTA TATTAAAATT
3D7                                              ...... .......... .......... .......... .......... ..........
RO33                                             ...... .......... .......... .......... .......... ..........
Palo Alto                                        ...... .......... .......... .......... .......... ..........
FCR3                                             ...... .......... .......... .......... .......... ..........
Wellcome                                         ...... .......... .......... .......... .......... ..........
D6                                               ...... .......... .......... .......... .......... ..........
T996                                             ...... .......... .......... .......... .......... ..........
T9102                                            ...... .......... .......... .......... .......... ..........
K1                                               ...... .......... .......... .......... .......... ..........
Dd2                                              ...... .......... .......... .......... .......... ..........
D10                                              ...... .......... .......... .......... .......... ..........
FCC2                                             ...... .......... .......... .......... .......... ..........
HB3                                              ...... .......... .......... .......... .......... ..........
7G8                                              ...... .......... .......... .......... .......... ..........
P.reichenowi                                     ...... .......... .......... .......... .......... ..........

                               90        100        110        120        130        140        150        160
                       ....|....| ....|....| ....|....| ....|....| ....|....| ....|....| ....|....| ....|....|
PF13_0348              TATTATAACA AACAATATAG AAATATACAA TGAAGAGAGT TACAATGTTT TAATAAATCT AGTAAAATCA AATAAAATAA
3D7                    .......... .......... .......... .......... .......... .......... .......... ..........
RO33                   .......... .......... .......... .......... .......... .......... .......... ..........
Palo Alto              .......... .......... .......... .......... .......... .......... .......... ..........
FCR3                   .......... .......... .......... .......... .......... .......... .......... ..........
Wellcome               .......... .......... .......... .......... .......... .......... .......... ..........
D6                     .......... .......... .......... .......... .......... .......... .......... ..........
T996                   .......... .......... .......... .......... .......... .......... .......... ..........
T9102                  .......... .......... .......... .......... .......... .......... .......... ..........
K1                     .......... .......... .......... .......... .......... .......... .......... ..........
Dd2                    .......... .......... .......... .......... .......... .......... .......... ..........
D10                    .......... .......... .......... .......... .......... .......... .......... ..........
FCC2                   .......... .......... .......... .......... .......... .......... .......... ..........
HB3                    .......... .......... .......... .......... .......... .......... .......... ..........
7G8                    .......... .......... .......... .......... .......... .......... .......... ..........
P.reichenowi           .......... .......... .......... .......... .......... .......... .......... ..........

                              170        180        190        200        210        220        230        240
                       ....|....| ....|....| ....|....| ....|....| ....|....| ....|....| ....|....| ....|....|
PF13_0348              TAACTGTAGA GGAATCTAAA AGGATCTTTT CAAATAACAA GAAATTATTC AAAAGGTATA TGTATAAGAA AAATAAAAGA
3D7                    .......... .......... .......... .......... .......... .......... .......... ..........
RO33                   .......... .......... .......... .......... .......... .......... .......... ..........
Palo Alto              .......... .......... .......... .......... .......... .......... .......... ..........
FCR3                   .......... .......... .......... .......... .......... .......... .......... ..........
Wellcome               .......... .......... .......... .......... .......... .......... .......... ..........
D6                     .......... .......... .......... .......... .......... .......... .......... ..........
T996                   .......... .......... .......... .......... .......... .......... .......... ..........
T9102                  .......... .......... .......... .......... .......... .......... .......... ..........
K1                     .......... .......... .......... .......... .......... .......... .......... ..........
Dd2                    .......... .......... .......... .......... .......... .......... .......... ..........
D10                    .......... .......... .......... .......... .......... .......... .......... ..........
FCC2                   .......... .......... .......... .......... .......... .......... .......... ..........
HB3                    .......... .......... .......... .......... .......... .......... .......... ..........
7G8                    .......... .......... .......... .......... .......... .......... .......... ..........
P.reichenowi           .......... .......... .......... .......... .......... .......... .......... .......C..

                              250        260        270        280        290        300        310        320
                       ....|....| ....|....| ....|....| ....|....| ....|....| ....|....| ....|....| ....|....|
PF13_0348              AGGCCATGTC AAATAAACAA CACTCATAAT ATTGATGAT- ---------- ---------- ---------- ----------
3D7                    .......... .......... .......... .........- ---------- ---------- ---------- ----------
RO33                   .......... .......... .......... .........- ---------- ---------- GATAATAATA ATAATAATAT
Palo Alto              .......... .........G .......... .........- ---------- ---------- GATAATAATA ATAATAATAT
FCR3                   .......... .......... .......... .........- ---------- ---------- GATAATAATA ATAATAATAT
Wellcome               .......... .......... .......... .........- ---------- ---------- GATAATAATA ATAATAATAT
D6                     .......... .......... .......... .........- ---------- ---------- GATAATAATA ATAATAATAT
T996                   .......... .......... .......... .........A ATAATAATAA TAATATTGAT GATAATAATA ATAATAATAT
T9102                  .......... .......... .......... .........- ---------- ---------- GATAATAATA ATAATAATAT
K1                     .......... .......... .......... .........- ---------- ---------- GATAATAATA ATAATAATAT
Dd2                    .......... .......... .......... .........- ---------- ---------- GATAATAATA ATAATAATAT
D10                    .......... .......... .......... .........- ---------- ---------- GATAATAATA ATAATAATAT
FCC2                   .......... .......... .......... .........- ---------- ---------- GATAATAATA ATAATAATAT
HB3                    .......... .......... .......... .........- ---------- ---------- GATAATAATA ATAATAATAT
7G8                    .......... .......... .......... .........- ---------- ---------- GATAATAATA ATAATAATAT
P.reichenowi           .......... .T........ ..A.A..... .........- ---------- -------GAT GATAATAATA AT--------
```

```
                                 330        340        350        360        370        380        390        400
                           ....|....| ....|....| ....|....| ....|....| ....|....| ....|....| ....|....| ....|....|
PF13_0348                  ----GATAAT AATAATAATA ATAAT----- ---------- ---------- --ATTCTTAA GAATTATAGT CCTAGTGAGA
3D7                        ----...... .......... .....----- ---------- ---------- --........ .......... ..........
RO33                       TGAT...... .......... .....AAT-- ---------- ---------- --........ .......... ..........
Palo Alto                  TGAT...... .......... .....ATTGA TGATAATAAT AATAATAATA AT........ .......... ..........
FCR3                       TGAT...... .......... .....----- ---------- ---------- --........ .......... ..........
Wellcome                   TGAT...... .......... .....----- ---------- ---------- --........ .......... ..........
D6                         TGAT...... .......... .....----- ---------- ---------- --........ .......... ..........
T996                       TGAT...... .......... .....----- ---------- ---------- --........ .......... ..........
T9102                      TGAT...... .......... .....----- ---------- ---------- --........ .......... ..........
K1                         TGAT...... .......... .....----- ---------- ---------- --........ .......... ..........
Dd2                        TGAT...... .......... .....----- ---------- ---------- --........ .......... ..........
D10                        TGAT...... .......... .....----- ---------- ---------- --........ .......... ..........
FCC2                       TGAT...... .......... .....----- ---------- ---------- --........ .......... ..........
HB3                        TGAT...... .......... .....----- ---------- ---------- --........ .......... ..........
7G8                        TGAT...... .......... .....----- ---------- ---------- --........ .......... ..........
P.reichenowi               ---------- ---------- ---------- ---------- ---------- --........ .......... ..........

                                 410        420        430        440        450        460        470        480
                           ....|....| ....|....| ....|....| ....|....| ....|....| ....|....| ....|....| ....|....|
PF13_0348                  TACACACCGA TTGTGCGAAT ATCTGTAACG ATGATAAAAA AATGGATGTT AATCAAAATT TATATAACAA AGAAATATAC
3D7                        .......... .......... .......... .......... .......... .......... .......... ..........
RO33                       .......... .......... .......... .......... .......... .......... .......... ..........
Palo Alto                  .......... .......... .......... .......... .......... .......... .......... ..........
FCR3                       .......... .......... .......... .......... .......... .......... .......... ..........
Wellcome                   .......... .......... .......... .......... .......... .......... .......... ..........
D6                         .......... .......... .......... .......... .......... .......... .......... ..........
T996                       .......... .......... .......... .......... .......... .......... .......... ..........
T9102                      .......... .......... .......... .......... .......... .......... .......... ..........
K1                         .......... .......... .......... .......... .......... .......... .......... ..........
Dd2                        .......... .......... .......... .......... .......... .......... .......... ..........
D10                        .......... .......... .......... .......... .......... .......... .......... ..........
FCC2                       .......... .......... .......... .......... .......... .......... .......... ..........
HB3                        .......... .......... .......... .......... .......... .......... .......... ..........
7G8                        .......... .......... .......... .......... .......... .......... .......... ..........
P.reichenowi               .......... .......... ..T.C..... .......... .......... .......... .......... ..........

                                 490        500        510        520        530        540        550        560
                           ....|....| ....|....| ....|....| ....|....| ....|....| ....|....| ....|....| ....|....|
PF13_0348                  ACAAATGATT CCCTTTTTTC TAAAGATCTA AACAATAAAA TATTATTTGA GTTTTTTCAA TTCCCCGAAA GTACTTACGT
3D7                        .......... .......... .......... .......... .......... .......... .......... ..........
RO33                       .......... .......... .......... .......... .......... .......... .......... ..........
Palo Alto                  .......... .......... .......... .......... .......... .......... .......... ..........
FCR3                       .......... .......... .......... .......... .......... .......... .......... ..........
Wellcome                   .......... .......... .......... .......... .......... .......... .......... ..........
D6                         .......... .......... .......... .......... .......... .......... .......... ..........
T996                       .......... .......... .......... .......... .......... .......... .......... ..........
T9102                      .......... .......... .......... .......... .......... .......... .......... ..........
K1                         .......... .......... .......... .......... .......... .......... .......... ..........
Dd2                        .......... .......... .......... .......... .......... .......... .......... ..........
D10                        .......... .......... .......... .......... .......... .......... .......... ..........
FCC2                       .......... .......... .......... .......... .......... .......... .......... ..........
HB3                        .......... .......... .......... .......... .......... .......... .......... ..........
7G8                        .......... .......... .......... .......... .......... .......... .......... ..........
P.reichenowi               .......... .......... ...G...... .......... .......... .......... .....T.... ..........

                                 570        580        590        600        610        620        630        640
                           ....|....| ....|....| ....|....| ....|....| ....|....| ....|....| ....|....| ....|....|
PF13_0348                  GTCAGAATAT ATTAATAATT TATGTAAGGA AAAGTTGATA CATGAACCGT TAAATATAAA TTTGAGTCTT GTTAAATTTG
3D7                        .......... .......... .......... .......... .......... .......... .......... ..........
RO33                       .......... .......... .......... .......... .......... .......... .......... ..........
Palo Alto                  .......... .......... .......... .......... .......... .......... .......... ..........
FCR3                       .......... .......... .......... .......... .......... .......... .......... ..........
Wellcome                   .......... .......... .......... .......... .......... .......... .......... ..........
D6                         .......... .......... .......... .......... .......... .......... .......... ..........
T996                       .......... .......... .......... .......... .......... .......... .......... ..........
T9102                      .......... .......... .......... .......... .......... .......... .......... ..........
K1                         .......... .......... .......... .......... .......... .......... .......... ..........
Dd2                        .......... .......... .......... .......... .......... .......... .......... ..........
D10                        .......... .......... .......... .......... .......... .......... .......... ..........
FCC2                       .......... .......... .......... .......... .......... .......... .......... ..........
HB3                        .......... .......... .......... .......... .......... .......... .......... ..........
7G8                        .......... .......... .......... .......... .......... .......... .......... ..........
P.reichenowi               .......... .......... .......... .......... ........A. .......... .......... ..........
```

```
                       650        660        670        680        690        700        710        720
                ....|....| ....|....| ....|....| ....|....| ....|....| ....|....| ....|....| ....|....|
PF13_0348       ATAAGAAGAA AAAGAAAAGC AAAAAAAGAA AAACATGTAA AAGGAATAAT ATTGGAGAGA TGCAAACAAG TACAAATAAA
3D7             .......... .......... .......... .......... .......... .......... .......... ..........
RO33            .......... .......... .......... .......... .......... .......... .......... ..........
Palo Alto       .......... .......... .......... .......... .......... .......... .......... ..........
FCR3            .......... .......... .......... .......... .......... .......... .......... ..........
Wellcome        .......... .......... .......... .......... .......... .......... .......... ..........
D6              .......... .......... .......... .......... .......... .......... .......... ..........
T996            .......... .......... .......... .......... .......... .......... .......... ..........
T9102           .......... .......... .......... .......... .......... .......... .......... ..........
K1              .......... .......... .......... .......... .......... .......... .......... ..........
Dd2             .......... .......... .......... .......... .......... .......... .......... ..........
D10             .......... .......... .......... .......... .......... .......... .......... ..........
FCC2            .......... .......... .......... .......... .......... .......... .......... ..........
HB3             .......... .......... .......... .......... .......... .......... .......... ..........
7G8             .......... .......... .......... .......... .......... .......... .......... ..........
P.reichenowi    .......... .......... .......... .......... .......... .........T ....G..... ...C......

                       730        740        750        760        770        780        790        800
                ....|....| ....|....| ....|....| ....|....| ....|....| ....|....| ....|....| ....|....|
PF13_0348       TATAACAGTG ATTCAGGTAT AGGGGTCACG GCAAAAACAA ATACGGACAT AAATAAAAAC ATTAATAGGA ATGTAAATAG
3D7             .......... .......... .......... .......... .......... .......... .......... ..........
RO33            .......... .......... .......... .......... .......... .......... .......... ..........
Palo Alto       .......... .......... .......... .......... .......... .......... .......... ..........
FCR3            .......... .......... .......... .......... .......... .......... .......... ..........
Wellcome        .......... .......... .......... .......... .......... .......... .......... ..........
D6              .......... .......... .......... .......... .......... .......... .......... ..........
T996            .......... .......... .......... .......... .......... .......... .......... ..........
T9102           .......... .......... .......... .......... .......... .......... .......... ..........
K1              .......... .......... .......... .......... .......... .......... .......... ..........
Dd2             .......... .......... .......... .......... .......... .......... .......... ..........
D10             .......... .......... .......... .......... .......... .......... .......... ..........
FCC2            .......... .......... .......... .......... .......... .......... .......... ..........
HB3             .......... .......... .......... .......... .......... .......... .......... ..........
7G8             .......... .......... .......... .......... .......... .......... .......... ..........
P.reichenowi    .......... ....G..... .....C.... .......... ....A..... .........T .......... ..........

                       810        820        830        840        850        860        870        880
                ....|....| ....|....| ....|....| ....|....| ....|....| ....|....| ....|....| ....|....|
PF13_0348       AAATATAACC ATGATGGGAA ACGCAAACAC AAACACGAAT GCAAACACAA ACACAAATGC AAACGTCAAC ACCAACGTCA
3D7             .......... .......... .......... .......... .......... .......... .......... ..........
RO33            .......... .......... .......... .......... .......... .......... .......... ..........
Palo Alto       .......... .......... .......... .......... .......... .......... .......... ..........
FCR3            .......... .......... .......... .......... .......... .......... .......... ..........
Wellcome        .......... .......... .......... .......... .......... .......... .......... ..........
D6              .......... .......... .......... .......... .......... .....----- ---------- ----------
T996            .......... .......... .......... .......... .......... .......... .......... ..........
T9102           .......... .......... ........-- ---------- ---------- ---------- ---------- ..........
K1              .......... .......... .......... .......... .......... .......... .......... ..........
Dd2             .......... .......... ........-- ---------- ---------- ---------- ---------- ..........
D10             .......... .......... .......... .......... .......... .......... .......... ..........
FCC2            .......... .......... .......... .......... .......... .......... .......... ..........
HB3             .......... .......... .......... .......... .......... .......... .......... ..........
7G8             .......... .......... .......... .......... .......... .......... .......... ..........
P.reichenowi    .......... .....A.A.. .T........ ...A.T...C A....A.TG. .......AAT G...ACA..T G.A..TACA.

                       890        900        910        920        930        940        950        960
                ....|....| ....|....| ....|....| ....|....| ....|....| ....|....| ....|....| ....|....|
PF13_0348       ACACCAACGT CAACACC--- ---------- ---------- ---------- ---------- ---------- -------AAC
3D7             .......... .......--- ---------- ---------- ---------- ---------- ---------- -------...
RO33            ........AC ......A--- ---------A ATGCAAACGT CAACACCAAC GTCAACACCA ACACCAACAC AAATGCA...
Palo Alto       .......... .......AAC ACCAACACAA ATGCAAACGT CAACACCAAC GTCAACACC- ---------- -------...
FCR3            ........AC ......A--- ---------A ATGCAAACGT CAACACCAAC GTCAACACCA ACACCAACAC AAATGCA...
Wellcome        ........AC ......A--- ---------A ATGCAAACGT CAACACCAAC GTCAACACCA ACACCAACAC AAATGCA...
D6              ---------- -.....A--- ---------A ATGCAAACGT CAACACCAAC GTCAACACCA ACACCAACAC AAATGCA...
T996            ........AC ......A--- ---------A ATGCAAACGT CAACACCAAC GTCAACACCA ACACCAACAC AAATGCA...
T9102           ........AC .......AAC ACA------A ATGCAAACGT CAACACCAAC GTCAACACCA ACACCAACAC AAATGCA...
K1              ........AC ......A--- ---------A ATGCAAACGT CAACACCAAC GTCAACACCA ACACCAACAC AAATGCA...
Dd2             ........AC .......AAC ACA------A ATGCAAACGT CAACACCAAC GTCAACACCA ACACCAACAC AAATGCA...
D10             ........AC ......A--- ---------A ATGCAAACGT CAACACCAAC GTCAACACCA ACACCAACAC AAATGCA...
FCC2            ........AC ......A--- ---------A ATGCAAACGT CAACACCAAC GTCAACACCA ACACCAACAC AAATGCA...
HB3             ........AC ......A--- ---------A ATGCAAACGT CAACACCAAC GTCAACACCA ACACCAACAC AAATGCA---
7G8             ........AC ......A--- ---------A ATGCAAACGT CAACACCAAC GTCAACACCA ACACCAACAC AAATGCA---
P.reichenowi    TT..A...AC ...T..AAAT ACAATTACAA ACACCAATAC AAATACAATC ACAAACACCA ATACAAATAC CGAGACA..T
```

```
                              970        980        990        1000       1010       1020       1030       1040
                     ....|....| ....|....| ....|....| ....|....| ....|....| ....|....| ....|....| ....|....|
PF13_0348            GTCAACACCA ACGTCAACAC CAACACCAAC ACC------A ACGTCAACAC CAACACCAAC ACCAACGTCA ACACCAACAC
3D7                  .......... .......... .......... ...------. .......... .......... .......... ..........
RO33                 .......... .......... .......... ..AAATGCA. .......... .--------- ---....... ..........
Palo Alto            .......... .......... .......... ..AAATGCA. ..A....... .--------- ---....... ..........
FCR3                 .......... .......... .......... ..AAATGCA. .......... .--------- ---....... ..........
Wellcome             .......... .......... .......... ..AAATGCA. .......... .--------- ---....... ..........
D6                   .......... .......... .......... ..AAATGCA. .......... .--------- ---....... ..........
T996                 .......... .......... .......... ..AAATGCA. .......... .--------- ---....... ..........
T9102                .......... .......... .......... ..AAATGCA. .......... .--------- ---....... ..........
K1                   .......... .......... .......... ..AAATGCA. .......... .--------- ---....... ..........
Dd2                  .......... .......... .......... ..AAATGCA. .......... .--------- ---....... ..........
D10                  .......... .......... .......... ..AAATGCA. .......... .--------- ---....... ..........
FCC2                 .......... .......... .......... ..AAATGCA. .......... .--------- ---....... ..........
HB3                  ---------- ---------- ---------- ---------. ..A....... .--------- ---....... ..........
7G8                  ---------- ---------- ---------- ---------. ..A....... .--------- ---....... ..........
P.reichenowi         ---------- ---------- ---------- ---------- ---------- ---------- ---------- ----------
```

```
                              1050       1060       1070       1080       1090       1100       1110       1120
                     ....|....| ....|....| ....|....| ....|....| ....|....| ....|....| ....|....| ....|....|
PF13_0348            CAATACAAAT ---------- ---------- ---------- ---------- ---------- ---------- ----------
3D7                  .......... ---------- ---------- ---------- ---------- ---------- ---------- ----------
RO33                 .......... ---------- ---------- ---------- ---------- ---------- ---------- ----------
Palo Alto            .......... ---------- ---------- ---------- ---------- ---------- ---------- ----------
FCR3                 ...C...... GCAAACGTCA ACACCAACGT CAACACCAAC ACCAACACAA ATGCAAACGT CAACACCAAC GTCAACACCA
Wellcome             ...C...... GCAAACGTCA ACACCAACGT CAACACCAAC ACCAACACAA ATGCAAACGT CAACACCAAC GTCAACACCA
D6                   ...CGTC..C ---------- ---------- ----ACCAAC ACCAATACAA AT-------- ---------- ----------
T996                 .......... ---------- ---------- ---------- ---------- ---------- ---------- ----------
T9102                ...C...... GCAAACGTCA ACACCAACGT CAACACCAAC ACCAACACAA ATGCAAACGT CAACACCAAC GTCAACACCA
K1                   .......... ---------- ---------- ---------- ---------- ---------- ---------- ----------
Dd2                  ...C...... GCAAACGTCA ACACCAACGT CAACACCAAC ACCAACACAA ATGCAAACGT CAACACCAAC GTCAACACCA
D10                  ...C...... GCAAACGTCA ACACCAACGT CAACACCAAC ACCAACACCA ACACAAATGC AAACGTCAAC ACCAACGTCA
FCC2                 .......... ---------- ---------- ---------- ---------- ---------- ---------- ----------
HB3                  .......... ---------- ---------- ---------- ---------- ---------- ---------- ----------
7G8                  .......... ---------- ---------- ---------- ---------- ---------- ---------- ----------
P.reichenowi         ---------- ---------- ---------- ---------- ---------- ---------- ---------- ----------
```

```
                              1130       1140       1150       1160       1170       1180       1190       1200
                     ....|....| ....|....| ....|....| ....|....| ....|....| ....|....| ....|....| ....|....|
PF13_0348            ---------- ---------- GCCGAGACAA ATGCCGAGAT AAATACCGAA AGTTTTGATC GGGAAGAGCA CAATAAGTTT
3D7                  ---------- ---------- .......... .......... .......... .......... .......... ..........
RO33                 ---------- ---------- .......... ...A...... .......... .......... .......... ..........
Palo Alto            ---------- ---------- .......... .......... .......... .......... .......... ..........
FCR3                 ACACCAATAC AAAT------ ---------- --........ .......... .......... .......... ..........
Wellcome             ACACCAATAC AAAT------ ---------- --........ .......... .......... .......... ..........
D6                   ---------- ---------- .......... .......... .......... .......... .......... ..........
T996                 ---------- ---------- .......... .......... .......... .......... .......... ..........
T9102                ACACCAATAC AAAT------ ---------- --........ .......... .......... .......... ..........
K1                   ---------- ---------- ---------- --........ .......... .......... .......... ..........
Dd2                  ACACCAATAC AAAT------ ---------- --........ .......... .......... .......... ..........
D10                  ACACCAACAC CAATACAAAT ---------- --........ .......... .......... .......... ..........
FCC2                 ---------- ---------- ---------- --........ .......... .......... .......... ..........
HB3                  ---------- ---------- .......... .......... .......... .......... .......... ..........
7G8                  ---------- ---------- .......... .......... .......... .......... .......... ..........
P.reichenowi         ---------- ---------- .......... .........C ......A... .......... .......... ..........
```

```
                              1210       1220       1230       1240       1250       1260       1270       1280
                     ....|....| ....|....| ....|....| ....|....| ....|....| ....|....| ....|....| ....|....|
PF13_0348            TATAAAATAT CATCAAAATG TTTTTCAAGA GTATTAAACG AGGATGATGT ATCACTTCCT AATATATATT ACATTAAAGG
3D7                  .......... .......... .......... .......... .......... .......... .......... ..........
RO33                 .......... .......... .......... .......... .......... .......... .......... ..........
Palo Alto            .......... .......... .......... .......... .......... .......... .......... ..........
FCR3                 .......... .......... .......... .......... .......... .......... .......... ..........
Wellcome             .......... .......... .......... .......... .......... .......... .......... ..........
D6                   .......... .......... .......... .......... .......... .......... .......... ..........
T996                 .......... .......... .......... .......... .......... .......... .......... ..........
T9102                .......... .......... .......... .......... .......... .......... .......... ..........
K1                   .......... .......... .......... .......... .......... .......... .......... ..........
Dd2                  .......... .......... .......... .......... .......... .......... .......... ..........
D10                  .......... .......... .......... .......... .......... .......... .......... ..........
FCC2                 .......... .......... .......... .......... .......... .......... .......... ..........
HB3                  .......... .......... .......... .......... .......... .......... .......... ..........
7G8                  .......... .......... .......... .......... .......... .......... .......... ..........
P.reichenowi         .......... .......... .......... .......... .......... .......... .......... ..........
```

```
                         1290       1300       1310       1320       1330       1340       1350       1360
                ....|....| ....|....| ....|....| ....|....| ....|....| ....|....| ....|....| ....|....|
PF13_0348       CGTTTCAAAC GATAATATAA ATGTTCATAT AAATTACAAT ATAAATAATA AAATGAATAA CAATATATTT AAAAATGAAA
3D7             .......... .......... .......... .......... .......... .......... .......... ..........
RO33            .......... .......... .......... .......... .......... .......... .......... ..........
Palo Alto       .......... .......... .......... .......... .......... .......... .......... ..........
FCR3            .......... .......... .......... .......... .......... .......... .......... ..........
Wellcome        .......... .......... .......... .......... .......... .......... .......... ..........
D6              .......... .......... .......... .......... .......... .......... .......... ..........
T996            .......... .......... .......... .......... .......... .......... .......... ..........
T9102           .......... .......... .......... .......... .......... .......... .......... ..........
K1              .......... .......... .......... .......... .......... .......... .......... ..........
Dd2             .......... .......... .......... .......... .......... .......... .......... ..........
D10             .......... .......... .......... .......... .......... .......... .......... ..........
FCC2            .......... .......... .......... .......... .......... .......... .......... ..........
HB3             .......... .......... .......... .......... .......... .......... .......... ..........
7G8             .......... .......... .......... .......... .......... .......... .......... ..........
P.reichenowi    ......G... .......... .......... ......T... .......... ....A..... ......T... ..........

                         1370       1380       1390       1400       1410       1420       1430       1440
                ....|....| ....|....| ....|....| ....|....| ....|....| ....|....| ....|....| ....|....|
PF13_0348       ATAATACTCT TGTCGAAAGT TTTAAAACAG ATTTATTGTA TTCAATAGAA AAAAATAAAA ATGACATATA TGCGCATAAT
3D7             .......... .......... .......... .......... .......... .......... .......... ..........
RO33            .......... .......... .......... .......... .......... .......... .......... ..........
Palo Alto       .......... .......... .......... .......... .......... .......... .......... ..........
FCR3            .......... .......... .......... .......... .......... .......... .......... ..........
Wellcome        .......... .......... .......... .......... .......... .......... .......... ..........
D6              .......... .......... .......... .......... .......... .......... .......... ..........
T996            .......... .......... .......... .......... .......... .......... .......... ..........
T9102           .......... .......... .......... .......... .......... .......... .......... ..........
K1              .......... .......... .......... .......... .......... .......... .......... ..........
Dd2             .......... .......... .......... .......... .......... .......... .......... ..........
D10             .......... .......... .......... .......... .......... .......... .......... ..........
FCC2            .......... .......... .......... .......... .......... .......... .......... ..........
HB3             .......... .......... .......... .......... .......... .......... .......... ..........
7G8             .......... .......... .......... .......... .......... .......... .......... ..........
P.reichenowi    ...G...... .......... .....GC... .......... .G........ .......... G...T..... .A.A......

                         1450       1460       1470       1480       1490       1500       1510       1520
                ....|....| ....|....| ....|....| ....|....| ....|....| ....|....| ....|....| ....|....|
PF13_0348       TGTAGTAATT CAGATGAGGA TTCGATATAT AATTATTTTA GTGATAATAC AAATATTGAT GATCATAATA AAATGAATAA
3D7             .......... .......... .......... .......... .......... .......... .......... ..........
RO33            .......... .......... .......... .......... .......... .......... .......... ..........
Palo Alto       .......... .......... .......... .......... .......... .......... .......... ..........
FCR3            .......... .......... .......... .......... .......... .......... .......... ..........
Wellcome        .......... .......... .......... .......... .......... .......... .......... ..........
D6              .......... .......... .......... .......... .......... .......... .......... ..........
T996            .......... .......... .......... .......... .......... .......... .......... ..........
T9102           .......... .......... .......... .......... .......... .......... .......... ..........
K1              .......... .......... .......... .......... .......... .......... .......... ..........
Dd2             .......... .......... .......... .......... .......... .......... .......... ..........
D10             .......... .......... .......... .......... .......... .......... .......... ..........
FCC2            .......... .......... .......... .......... .......... .......... .......... ..........
HB3             .......... .......... .......... .......... .......... .......... .......... ..........
7G8             .......... .......... .......... .......... .......... .......... .......... ..........
P.reichenowi    ........C. .......A.. .......... ..C....... ....C..... ..G..C..T. ...A...... ..........

                         1530       1540       1550       1560       1570       1580       1590       1600
                ....|....| ....|....| ....|....| ....|....| ....|....| ....|....| ....|....| ....|....|
PF13_0348       TGTAATTAGG GAACGCGTTA ATAATATAGG TAAGAATTAT TATAGCACAG AAAAGTATAA TAATAACAAT AATGATAATA
3D7             .......... .......... .......... .......... .......... .......... .......... ..........
RO33            .......... .......... .......... .......... .......... .......... .......... ..........
Palo Alto       .......... .......... .......... .......... .......... .......... .......... ..........
FCR3            .......... .......... .......... .......... .......... .......... .......... ..........
Wellcome        .......... .......... .......... .......... .......... .......... .......... ..........
D6              .......... .......... .......... .......... .......... .......... .......... ..........
T996            .......... .......... .......... .......... .......... .......... .......... ..........
T9102           .......... .......... .......... .......... .......... .......... .......... ..........
K1              .......... .......... .......... .......... .......... .......... .......... ..........
Dd2             .......... .......... .......... .......... .......... .......... .......... ..........
D10             .......... .......... .......... .......... .......... .......... .......... ..........
FCC2            .......... .......... .......... .......... .......... .......... .......... ..........
HB3             .......... .......... .......... .......... .......... .......... .......... ..........
7G8             .......... .......... .......... .......... .......... .......... .......... ..........
P.reichenowi    .......... .......... .C......A. .......... ....AT.... ...T...... ......A... ..........
```

```
                       1610       1620       1630       1640       1650       1660       1670       1680
                ....|....| ....|....| ....|....| ....|....| ....|....| ....|....| ....|....| ....|....|
PF13_0348       ATAACAATAA TGATAATAAT AACAATAATG ATAATAATAA CAATAATGAT AATAATAACA AC-------- ----------
3D7             .......... .......... .......... .......... .......... .......... ..-------- ----------
RO33            .......... .......... .......... .......... .......... .......... ..-------- ----------
Palo Alto       .......... .......... .......... .......... .......... .......... ..-------- ----------
FCR3            .......... .......... .......... .......... .......... .......... ..-------- ----------
Wellcome        .......... .......... .......... .......... .......... .......... ..-------- ----------
D6              .......... .......... .......... .......... .......... .......... ..-------- ----------
T996            .......... .......... .......... .......... .......... .......... ..-------- ----------
T9102           .......... .......... .......... .......... ...C------ ---------- ---------- ----------
K1              .......... .......... .......... .......... ...C------ ---------- ---------- ----------
Dd2             .......... .......... .......... .......... ...C------ ---------- ---------- ----------
D10             .......... .......... .......... .......... .......... .......... ..-------- ----------
FCC2            .......... .......... .......... .......... ...C------ ---------- ---------- ----------
HB3             .......... .......... .......... .......... .......... .......... .TAATGATAA TAATAACAAC
7G8             .......... .......... .......... .......... .......... .......... ..-------- ----------
P.reichenowi    ....T..C.. CA.C.....C .....C...A .C......-- ---------- ---------- ---------- ----------

                       1690       1700       1710       1720       1730       1740       1750       1760
                ....|....| ....|....| ....|....| ....|....| ....|....| ....|....| ....|....| ....|....|
PF13_0348       ATCAATAATA ATAATAATAA GAAAAAG--- AACAAGAACA ACAACAATAA TAATAATAAT TATAATAAT- --------AT
3D7             .......... .......... .......--- .......... .......... .......... .........- --------..
RO33            .......... .......... .......--- .....C.... .......... .......... .........- --------..
Palo Alto       .......... .......... .......--- .....C.... .......... .......... .........- --------..
FCR3            .......... .......... .......--- .....C.... .......... .......... .........- --------..
Wellcome        .......... .......... .......--- .....C.... .......... .......... .........- --------..
D6              .......... .......... .......--- .....C.... .......... .......... .........- --------..
T996            .......... .......... .......AAG .....C.... .......... .......... .........- --------..
T9102           .......... .......... .......--- .....C.... .......... .......... .........- --------..
K1              .......... .......... .......--- .....C.... .......... .......... .........- --------..
Dd2             .......... .......... .......--- .....C.... .......... .......... .........- --------..
D10             .......... .......... .......--- .....C.... .......... .......... .........- --------..
FCC2            .......... .......... .......--- .....C.... .......... .......... .........- --------..
HB3             .......... .......... .......--- .....C.... .......... .......... .........- --------..
7G8             .......... .......... .......--- .......... .......... .......... .........- --------..
P.reichenowi    ---------- ---------- ---------- ---------- ---------- ---------- .........T ACAATAAT..

                       1770       1780       1790       1800       1810       1820       1830       1840
                ....|....| ....|....| ....|....| ....|....| ....|....| ....|....| ....|....| ....|....|
PF13_0348       TTGTAATAGT AGTAGTAATA ATAAAATGAA CGATTATAAA CATATGATT- ---------- ---------- ----------
3D7             .......... .......... .......... .......... .........- ---------- ---------- ----------
RO33            .......... .......... .......... .......... .........- ---------- ---------- ----------
Palo Alto       .......... .......... .......... .......... .........- ---------- ---------- ----------
FCR3            .......... .......... .......... .......... .........- ---------- ---------- ----------
Wellcome        .......... .......... .......... .......... .........- ---------- ---------- ----------
D6              .......... .......... .......... .......... .........- ---------- ---------- ----------
T996            .......... .......... .......... .......... .........- ---------- ---------- ----------
T9102           .......... .......... .......... .......... .........- ---------- ---------- ----------
K1              .......... .......... .......... .......... .........- ---------- ---------- ----------
Dd2             .......... .......... .......... .......... .........- ---------- ---------- ----------
D10             .......... .......... .......... .......... .........- ---------- ---------- ----------
FCC2            .......... .......... .......... .......... .........- ---------- ---------- ----------
HB3             .......... .......... .......... .......... .........- ---------- ---------- ----------
7G8             .......... .......... .......... .......... .........- ---------- ---------- ----------
P.reichenowi    ..A.---... .......... .......... .......... ...----..A ATCATAATGA TAATTATAAT ACAGGTAATA

                       1850       1860       1870       1880       1890       1900       1910       1920
                ....|....| ....|....| ....|....| ....|....| ....|....| ....|....| ....|....| ....|....|
PF13_0348       ---------- ---------- ------TTTA ATAATAATGA TAATACCATA TTCAAGCATA AAAACTCATT ATCTTCTTTA
3D7             ---------- ---------- ------.... .......... .......... .......... .......... ..........
RO33            ---------- ---------- ------.... .......... .......... .......... .......... ..........
Palo Alto       ---------- ---------- ------.... .......... .......... .......... .......... ..........
FCR3            ---------- ---------- ------.... .......... .......... .......... .......... ..........
Wellcome        ---------- ---------- ------.... .......... .......... .......... .......... ..........
D6              ---------- ---------- ------.... .......... .......... .......... .......... ..........
T996            ---------- ---------- ------.... .......... .......... .......... .......... ..........
T9102           ---------- ---------- ------.... .......... .......... .......... .......... ..........
K1              ---------- ---------- ------.... .......... .......... .......... .......... ..........
Dd2             ---------- ---------- ------.... .......... .......... .......... .......... ..........
D10             ---------- ---------- ------.... .......... .......... .......... .......... ..........
FCC2            ---------- ---------- ------.... .......... .......... .......... .......... ..........
HB3             ---------- ---------- ------.... .......... .......... .......... .......... ..........
7G8             ---------- ---------- ------.... .......... .......... .......... .......... ..........
P.reichenowi    CTTATGATAA TACAAAACAT ATCGTT.... .......... ..G....... .....A.... .......... ..........
```

```
                                   1930       1940       1950       1960       1970       1980       1990       2000
                               ....|....| ....|....| ....|....| ....|....| ....|....| ....|....| ....|....| ....|....|
PF13_0348                      AAATTATCAA TGTATAATTA CAAAATATTT AAGAAGGATA CAACATTTAA TAGTACTCAT AATCTTTTTA GTTATGCTTT
3D7                            .......... .......... .......... .......... .......... .......... .......... ..........
RO33                           .......... .......... .......... .......... .......... .......... .......... ..........
Palo Alto                      .......... .......... .......... .......... .......... .......... .......... ..........
FCR3                           .......... .......... .......... .......... .......... .......... .......... ..........
Wellcome                       .......... .......... .......... .......... .......... .......... .......... ..........
D6                             .......... .......... .......... .......... .......... .......... .......... ..........
T996                           .......... .......... .......... .......... .......... .......... .......... ..........
T9102                          .......... .......... .......... .......... .......... .......... .......... ..........
K1                             .......... .......... .......... .......... .......... .......... .......... ..........
Dd2                            .......... .......... .......... .......... .......... .......... .......... ..........
D10                            .......... .......... .......... .......... .......... .......... .......... ..........
FCC2                           .......... .......... .......... .......... .......... .......... .......... ..........
HB3                            .......... .......... .......... .......... .......... .......... .......... ..........
7G8                            .......... .......... .......... .......... .......... .......... .......... ..........
P.reichenowi                   .......... .......... .......... .....C.... .......... .......... .......... .C........

                                   2010       2020       2030       2040       2050       2060       2070       2080
                               ....|....| ....|....| ....|....| ....|....| ....|....| ....|....| ....|....| ....|....|
PF13_0348                      AACGTTTAAA CATTTTTATG ATATACATAA TGTTTTGAAC AGAGATCCTA ATGGATTAAG AAAAATAAAA GTCATACTAA
3D7                            .......... .......... .......... .......... .......... .......... .......... ..........
RO33                           .......... .......... .......... .......... .......... .......... .......... ..........
Palo Alto                      .......... .......... .......... .......... .......... .......... .......... ..........
FCR3                           .......... .......... .......... .......... .......... .......... .......... ..........
Wellcome                       .......... .......... .......... .......... .......... .......... .......... ..........
D6                             .......... .......... .......... .......... .......... .......... .......... ..........
T996                           .......... .......... .......... .......... .......... .......... .......... ..........
T9102                          .......... .......... .......... .......... .......... .......... .......... ..........
K1                             .......... .......... .......... .......... .......... .......... .......... ..........
Dd2                            .......... .......... .......... .......... .......... .......... .......... ..........
D10                            .......... .......... .......... .......... .......... .......... .......... ..........
FCC2                           .......... .......... .......... .......... .......... .......... .......... ..........
HB3                            .......... .......... .......... .......... .......... .......... .......... ..........
7G8                            .......... .......... .......... .......... .......... .......... .......... ..........
P.reichenowi                   ...T...... .......... .......... ......A..T .......... .C........ .......... ..........

                                   2090       2100       2110       2120       2130       2140       2150       2160
                               ....|....| ....|....| ....|....| ....|....| ....|....| ....|....| ....|....| ....|....|
PF13_0348                      AAAAAGATAT TGGAACAATC AGCATATCAC CTTTTTATAT TAATTTTGAT TCTATACGTA TTTTTGATAA TGTATGTAAA
3D7                            .......... .......... .......... .......... .......... .......... .......... ..........
RO33                           .......... .......... .......... .......... .......... .......... .......... ..........
Palo Alto                      .......... .......... .......... .......... .......... .......... .......... ..........
FCR3                           .......... .......... .......... .......... .......... .......... .......... ..........
Wellcome                       .......... .......... .......... .......... .......... .......... .......... ..........
D6                             .......... .......... .......... .......... .......... .......... .......... ..........
T996                           .......... .......... .......... .......... .......... .......... .......... ..........
T9102                          .......... .......... .......... .......... .......... .......... .......... ..........
K1                             .......... .......... .......... .......... .......... .......... .......... ..........
Dd2                            .......... .......... .......... .......... .......... .......... .......... ..........
D10                            .......... .......... .......... .......... .......... .......... .......... ..........
FCC2                           .......... .......... .......... .......... .......... .......... .......... ..........
HB3                            .......... .......... .......... .......... .......... .......... .......... ..........
7G8                            .......... .......... .......... .......... .......... .......... .......... ..........
P.reichenowi                   .......... ...C...... .......... .......C.. .......... .......... .......... ..........

                                   2170       2180       2190       2200       2210       2220       2230       2240
                               ....|....| ....|....| ....|....| ....|....| ....|....| ....|....| ....|....| ....|....|
PF13_0348                      ACATTTAATA TTACAGACTA TAATTATAAA AAACAAAATA TATATAATTT TATGAATCTC TGCTCCACAA ATAATCAATC
3D7                            .......... .......... .......... .......... .......... .......... .......... ..........
RO33                           .......... .......... .......... .......... .......... .......... .......... ..........
Palo Alto                      .......... .......... .......... .......... .......... .......... .......... ..........
FCR3                           .......... .......... .......... .......... .......... .......... .......... ..........
Wellcome                       .......... .......... .......... .......... .......... .......... .......... ..........
D6                             .......... .......... .......... .......... .......... .......... .......... ..........
T996                           .......... .......... .......... .......... .......... .......... .......... ..........
T9102                          .......... .......... .......... .......... .......... .......... .......... ..........
K1                             .......... .......... .......... .......... .......... .......... .......... ..........
Dd2                            .......... .......... .......... .......... .......... .......... .......... ..........
D10                            .......... .......... .......... .......... .......... .......... .......... ..........
FCC2                           .......... .......... .......... .......... .......... .......... .......... ..........
HB3                            .......... .......... .......... .......... .......... .......... .......... ..........
7G8                            .......... .......... .......... .......... .......... .......... .......... ..........
P.reichenowi                   .......... .......... .......... .......... .......... .......... .....G.... ....A.....
```

```
                   2250       2260       2270       2280       2290       2300       2310       2320
             ....|....| ....|....| ....|....| ....|....| ....|....| ....|....| ....|....| ....|....|
PF13_0348    TTTATATATA CTCAAAAATA TTTTCTTAAA AGATTTATAT AAACAACAAA AGATTTATCA AAATGATCAT CATAATAACA
3D7          .......... .......... .......... .......... .......... .......... .......... ..........
RO33         .......... .......... .......... .......... .......... .C........ .......... ..........
Palo Alto    .......... .......... .......... .......... .......... .C........ .......... ..........
FCR3         .......... .......... .......... .......... .......... .......... .......... ..........
Wellcome     .......... .......... .......... .......... .......... .......... .......... ..........
D6           .......... .......... .......... .......... .......... .C........ .......... ..........
T996         .......... .......... .......... .......... .......... .C........ .......... ..........
T9102        .......... .......... .......... .......... .......... .......... .......... ..........
K1           .......... .......... .......... .......... .......... .......... .......... ..........
Dd2          .......... .......... .......... .......... .......... .......... .......... ..........
D10          .......... .......... .......... .......... .......... .......... .......... ..........
FCC2         .......... .......... .......... .......... .......... .......... .......... ..........
HB3          .......... .......... .......... .......... .......... .C........ .......... ..........
7G8          .......... .......... .......... .......... .......... .......... .......... ..........
P.reichenowi A......... ..G....... .......... ...C...... .......... .......... .......... ..........

                   2330       2340       2350       2360       2370       2380       2390       2400
             ....|....| ....|....| ....|....| ....|....| ....|....| ....|....| ....|....| ....|....|
PF13_0348    AAAAAAAAAT TTATAGAAAA AGAAATGCTT TAAGGAAAAA AAAAAAAAAA GAAAAAGAAA ATATTTTAGA AATATTAGAT
3D7          .......... .......... .......... .......... .......... .......... .......... ..........
RO33         .......... .......... .......... .......... .......... .......... .......... ..........
Palo Alto    .......... .......... .......... .......... .......... .......... .......... ..........
FCR3         .......... .......... .......... .......... .......... .......... .......... ..........
Wellcome     .......... .......... .......... .......... .......... .......... .......... ..........
D6           .......... .......... .......... .......... .......... .......... .......... ..........
T996         .......... .......... .......... .......... .......... .......... .......... ..........
T9102        .......... .......... .......... .......... .......... .......... .......... ..........
K1           .......... .......... .......... .......... .......... .......... .......... ..........
Dd2          .......... .......... .......... .......... .......... .......... .......... ..........
D10          .......... .......... .......... .......... .......... .......... .......... ..........
FCC2         .......... .......... .......... .......... .......... .......... .......... ..........
HB3          .......... .......... .....G.... .......... .......... .......... .......... ..........
7G8          .......... .......... .......... .......... .......... .......... .......... ..........
P.reichenowi .........G .......... .......... .......... ...G...... .......... .....C.... ......G...

                   2410       2420       2430       2440       2450       2460       2470       2480
             ....|....| ....|....| ....|....| ....|....| ....|....| ....|....| ....|....| ....|....|
PF13_0348    GAAGAGAAAA GAATTAATGT TCTATATTTT ATTAATCGTT TTCTATATTC AAATAAAACC CCTTTATCAT ATTTATTTAG
3D7          .......... .......... .......... .......... .......... .......... .......... ..........
RO33         .......... .......... .......... .......... .......... .......... .......... ..........
Palo Alto    .......... .......... .......... .......... .......... .......... .......... ..........
FCR3         .......... .......... .......... .......... .......... .......... .......... ..........
Wellcome     .......... .......... .......... .......... .......... .......... .......... ..........
D6           .......... .......... .......... .......... .......... .......... .......... ..........
T996         .......... .......... .......... .......... .......... .......... .......... ..........
T9102        .......... .......... .......... .......... .......... .......... .......... ..........
K1           .......... .......... .......... .......... .......... .......... .......... ..........
Dd2          .......... .......... .......... .......... .......... .......... .......... ..........
D10          .......... .......... .......... .......... .......... .......... .......... ..........
FCC2         .......... .......... .......... .......... .......... .......... .......... ..........
HB3          .......... .......... .......... .......... .......... .......... .......... ..........
7G8          .......... .......... .......... .......... .......... .......... .......... ..........
P.reichenowi .....A.... .......... .......... .......... .......... .........A .......... ..........

                   2490       2500       2510       2520       2530       2540       2550       2560
             ....|....| ....|....| ....|....| ....|....| ....|....| ....|....| ....|....| ....|....|
PF13_0348    AAATTATAAA ACAACTTTGC TTAACCAACA AATAAATGTG AACCAAAAAA ATGAACACAC ACATAAATGT ATACATTCAA
3D7          .......... .......... .......... .......... .......... .......... .......... ..........
RO33         .......... .......... .......... .......... .......... .......... .......... ..........
Palo Alto    .......... .......... .......... .......... .......... .......... .......... ..........
FCR3         .......... .......... .......... .......... .......... .......... .......... ..........
Wellcome     .......... .......... .......... .......... .......... .......... .......... ..........
D6           .......... .......... .......... .......... .......... .......... .......... ..........
T996         .......... .......... .......... .......... .......... .......... .......... ..........
T9102        .......... .......... .......... .......... .......... .......... .......... ..........
K1           .......... .......... .......... .......... .......... .......... .......... ..........
Dd2          .......... .......... .......... .......... .......... .......... .......... ..........
D10          .......... .......... .......... .......... .......... .......... .......... ..........
FCC2         .......... .......... .......C.. .......... .......... .......... .......... ..........
HB3          .......... .......... .......... .......... .......... .......... .......... ..........
7G8          .......... .......... .......... .......... .......... .......... .......... ..........
P.reichenowi ......C.C. ....A..... .....G.... .......... .......... .......... C......... ..........
```

```
                                2570       2580       2590       2600       2610       2620       2630       2640
                        ....|....| ....|....| ....|....| ....|....| ....|....| ....|....| ....|....| ....|....|
PF13_0348               ATCAAGTGAA ACAAGAAAAT GAATTCGTTT TATATTCGGA CGAATTAACT ATTGATTATT TTCTTTTCCT TATGATTTTT
3D7                     .......... .......... .......... .......... .......... .......... .......... ..........
RO33                    .......... .......... .......... .......... .......... .......... .......... ..........
Palo Alto               .......... .......... .......... .......... .......... .......... .......... ..........
FCR3                    .......... .......... .......... .......... .......... .......... .......... ..........
Wellcome                .......... .......... .......... .......... .......... .......... .......... ..........
D6                      .......... .......... .......... .......... .......... .......... .......... ..........
T996                    .......... .......... .......... .......... .......... .......... .......... ..........
T9102                   .........T .......... .......... .......... .......... .......... .......... ..........
K1                      .......... .......... .......... .......... .......... .......... .......... ..........
Dd2                     .........T .......... .......... .......... .......... .......... .......... ..........
D10                     .......... .......... .......... .......... .......... .......... .......... ..........
FCC2                    .......... .......... .......... .......... .......... .......... .......... ..........
HB3                     .......... .......... .......... .......... .......... .......... .......... ..........
7G8                     .......... .......... .......... .......... .......... .......... .......... ..........
P.reichenowi            ....G..... .......... .......... .......... .......... .......... .......... ..........

                                2650       2660       2670       2680       2690       2700       2710       2720
                        ....|....| ....|....| ....|....| ....|....| ....|....| ....|....| ....|....| ....|....|
PF13_0348               GAAAAGAAGG TGGAGCAAAA CTTTTCAAAC TATTTAATAA ACAAGATAAA TGAATGTAAA AAATTTAAAA CAACTGATAA
3D7                     .......... .......... .......... .......... .......... .......... .......... ..........
RO33                    .......... .......... .......... .......... .......... .......... .......... ..........
Palo Alto               .......... .......... .......... .......... .......... .......... .......... ..........
FCR3                    .......... .......... .......... .......... .......... .......... .......... ..........
Wellcome                .......... .......... .......... .......... .......... .......... .......... ..........
D6                      .......... .......... .......... .......... .......... .......... .......... ..........
T996                    .......... .......... .......... .......... .......... .......... .......... ..........
T9102                   .......... .......... .......... .......... .......... .......... .......... ..........
K1                      .......... .......... .......... .......... .......... .......... .......... ..........
Dd2                     .......... .......... .......... .......... .......... .......... .......... ..........
D10                     .......... .......... .......... .......... .......... .......... .......... ..........
FCC2                    .......... .......... .......... .......... .......... .......... .......... ..........
HB3                     .......... .......... .......... .......... .......... .......... .......... ..........
7G8                     .......... .......... .......... .......... .......... .......... .......... ..........
P.reichenowi            .......... .T...A.... .......... .......... .......... .......... .......... TT........

                                2730       2740       2750       2760       2770       2780       2790       2800
                        ....|....| ....|....| ....|....| ....|....| ....|....| ....|....| ....|....| ....|....|
PF13_0348               TACATATACA AACACGGACA ACAAGACGCA CGTATCTAAA GATGTATTGG TTCATTTTGA TGATGATATA TTTCTTAAAA
3D7                     .......... .......... .......... .......... .......... .......... .......... ..........
RO33                    .......... .......... .......... .......... .......... .......... .......... ..........
Palo Alto               .......... .......... .......... .......... .......... .......... .......... ..........
FCR3                    .......... .......... .......... .......... .......... .......... .......... ..........
Wellcome                .......... .......... .......... .......... .......... .......... .......... ..........
D6                      .......... .......... .......... .......... .......... .......... .......... ..........
T996                    .......... .......... .......... .......... .......... .......... .......... ..........
T9102                   .......... .......... .......... .......... .......... .......... .......... ..........
K1                      .......... .......... .......... .......... .......... .......... .......... ..........
Dd2                     .......... .......... .......... .......... .......... .......... .......... ..........
D10                     .......... .......... .......... .......... .......... .......... .......... ..........
FCC2                    .......... .......... .......... .......... .......... .......... .......... ..........
HB3                     .......... .......... .......... .......... .......... .......... .......... ..........
7G8                     .......... .......... .......... .......... .......... .......... .......... ..........
P.reichenowi            ....A..... G.....C... ......T... .......... ......A... ..G....A.. .......... ...G......

                                2810       2820       2830       2840       2850       2860       2870       2880
                        ....|....| ....|....| ....|....| ....|....| ....|....| ....|....| ....|....| ....|....|
PF13_0348               AATCATTTGA TGTGTCAACC AAAATTTCTC GTGATGTATC AAATAAAATG TCT------- ---------- ----------
3D7                     .......... .......... .......... .......... .......... ...------- ---------- ----------
RO33                    .......... .......... .......... .......... .......... ...------- ---------- ----------
Palo Alto               .......... .......... .......... .......... .......... ...------- ---------- ----------
FCR3                    .......... .......... ..T....... .......... .......... ...------- ---------- ----------
Wellcome                .......... .......... ..T....... .......... .......... ...------- ---------- ----------
D6                      .......... .......... .......... .......... .......... ...------- ---------- ----------
T996                    .......... .......... .......... .......... .......... ...------- ---------- ----------
T9102                   .......... .......... .......... .......... .......... ...------- ---------- ----------
K1                      .......... .......... .......... .......... .......... ...------- ---------- ----------
Dd2                     .......... .......... .......... .......... .......... ...------- ---------- ----------
D10                     .......... .......... .......... .......... .......... ...------- ---------- ----------
FCC2                    .......... .......... .......... .......... .......... ...------- ---------- ----------
HB3                     .......... .......... .......... .......... .......... ...------- ---------- ----------
7G8                     .......... .......... .......... .......... .......... ...------- ---------- ----------
P.reichenowi            .......... .......... .........G ......CG.. .......... ..AGGTGATG TGTCAAATAA AATGTCTGTT
```

```
                               2890       2900       2910       2920       2930       2940       2950       2960
                      ....|....| ....|....| ....|....| ....|....| ....|....| ....|....| ....|....| ....|....|
PF13_0348             ---------- ---------- ---------- ---------- ---------- ---------- ---------- ----------
3D7                   ---------- ---------- ---------- ---------- ---------- ---------- ---------- ----------
RO33                  ---------- ---------- ---------- ---------- ---------- ---------- ---------- ----------
Palo Alto             ---------- ---------- ---------- ---------- ---------- ---------- ---------- ----------
FCR3                  ---------- ---------- ---------- ---------- ---------- ---------- ---------- ----------
Wellcome              ---------- ---------- ---------- ---------- ---------- ---------- ---------- ----------
D6                    ---------- ---------- ---------- ---------- ---------- ---------- ---------- ----------
T996                  ---------- ---------- ---------- ---------- ---------- ---------- ---------- ----------
T9102                 ---------- ---------- ---------- ---------- ---------- ---------- ---------- ----------
K1                    ---------- ---------- ---------- ---------- ---------- ---------- ---------- ----------
Dd2                   ---------- ---------- ---------- ---------- ---------- ---------- ---------- ----------
D10                   ---------- ---------- ---------- ---------- ---------- ---------- ---------- ----------
FCC2                  ---------- ---------- ---------- ---------- ---------- ---------- ---------- ----------
HB3                   ---------- ---------- ---------- ---------- ---------- ---------- ---------- ----------
7G8                   ---------- ---------- ---------- ---------- ---------- ---------- ---------- ----------
P.reichenowi          GATGTGTCAA ATAAAATGTC TGTTGATGTG TCAAATAAAA TGTCTGGTGA TGTGTCAAAT AAAATGTCTG TTGATGTGTC

                               2970       2980       2990       3000       3010       3020       3030       3040
                      ....|....| ....|....| ....|....| ....|....| ....|....| ....|....| ....|....| ....|....|
PF13_0348             ---------- ---------- ---------- ---------- ---------- ---------- ---------- ----------
3D7                   ---------- ---------- ---------- ---------- ---------- ---------- ---------- ----------
RO33                  ---------- ---------- ---------- ---------- ---------- ---------- ---------- ----------
Palo Alto             ---------- ---------- ---------- ---------- ---------- ---------- ---------- ----------
FCR3                  ---------- ---------- ---------- ---------- ---------- ---------- ---------- ----------
Wellcome              ---------- ---------- ---------- ---------- ---------- ---------- ---------- ----------
D6                    ---------- ---------- ---------- ---------- ---------- ---------- ---------- ----------
T996                  ---------- ---------- ---------- ---------- ---------- ---------- ---------- ----------
T9102                 ---------- ---------- ---------- ---------- ---------- ---------- ---------- ----------
K1                    ---------- ---------- ---------- ---------- ---------- ---------- ---------- ----------
Dd2                   ---------- ---------- ---------- ---------- ---------- ---------- ---------- ----------
D10                   ---------- ---------- ---------- ---------- ---------- ---------- ---------- ----------
FCC2                  ---------- ---------- ---------- ---------- ---------- ---------- ---------- ----------
HB3                   ---------- ---------- ---------- ---------- ---------- ---------- ---------- ----------
7G8                   ---------- ---------- ---------- ---------- ---------- ---------- ---------- ----------
P.reichenowi          AAATAAAATG TCTGTTGATG TGTCAAATAA AATGTCTGTT GATGTGTCAA ATAAAATGTC TGGTGATGTG TCAAATAAAA

                               3050       3060       3070       3080       3090       3100       3110       3120
                      ....|....| ....|....| ....|....| ....|....| ....|....| ....|....| ....|....| ....|....|
PF13_0348             ---------- ---------- ---------- ---------- ---------- ---------- ---------- -------GTT
3D7                   ---------- ---------- ---------- ---------- ---------- ---------- ---------- -------...
RO33                  ---------- ---------- ---------- ---------- ---------- ---------- ---------- -------...
Palo Alto             ---------- ---------- ---------- ---------- ---------- ---------- ---------- -------...
FCR3                  ---------- ---------- ---------- ---------- ---------- ---------- ---------- -------...
Wellcome              ---------- ---------- ---------- ---------- ---------- ---------- ---------- -------...
D6                    ---------- ---------- ---------- ---------- ---------- ---------- ---------- -------...
T996                  ---------- ---------- ---------- ---------- ---------- ---------- ---------- -------...
T9102                 ---------- ---------- ---------- ---------- ---------- ---------- ---------- -------...
K1                    ---------- ---------- ---------- ---------- ---------- ---------- ---------- -------...
Dd2                   ---------- ---------- ---------- ---------- ---------- ---------- ---------- -------...
D10                   ---------- ---------- ---------- ---------- ---------- ---------- ---------- -------...
FCC2                  ---------- ---------- ---------- ---------- ---------- ---------- ---------- -------...
HB3                   ---------- ---------- ---------- ---------- ---------- ---------- ---------- -------...
7G8                   ---------- ---------- ---------- ---------- ---------- ---------- ---------- -------...
P.reichenowi          TGTCTGTTGA TATGTCTGTT GATATGTCTG TTGATATGTT TGGTGATATG TCTGTTGATA TGTCTGGTGA TATGTCTA..

                               3130       3140       3150       3160       3170       3180       3190       3200
                      ....|....| ....|....| ....|....| ....|....| ....|....| ....|....| ....|....| ....|....|
PF13_0348             GATATGTCTG TTGATGTGTC TGACGATTTG GAAAAAGAAT CTACTAAGGA TGATATGATA TTAGACAATA AACAAATAGA
3D7                   .......... .......... .......... .......... .......... .......... .......... ..........
RO33                  .......... .......... .......... .......... .......... .......... .......... ..........
Palo Alto             .......... .......... .......... .......... .......... .......... .......... ..........
FCR3                  .......... .......... .......... .......... .......... .......... .......... ..........
Wellcome              .......... .......... .......... .......... .......... .......... .......... ..........
D6                    .......... .......... .......... .......... .......... .......... .......... ..........
T996                  .......... .......... .......... .......... .......... .......... .......... ..........
T9102                 .......... .......... .......... .......... .......... .......... .......... ..........
K1                    .......... .......... .......... .......... .......... .......... .......... ..........
Dd2                   .......... .......... .......... .......... .......... .......... .......... ..........
D10                   .......... .......... .......... .......... .......... .......... .......... ..........
FCC2                  .......... .......... .......... .......... .......... .......... .......... ..........
HB3                   .......... .......... .......... .......... .......... .......... .......... ..........
7G8                   .......... .......... .......... .......... .......... .......... .......... ..........
P.reichenowi          .....A.... .........G .......... .......... .......... ..T...A... .....T.... ..........
```

```
                         3210       3220       3230       3240       3250       3260       3270       3280
                    ....|....| ....|....| ....|....| ....|....| ....|....| ....|....| ....|....| ....|....|
PF13_0348           AGACAGCAGC GATAATAATA ATAATAATAA TAATAATAAT AATAAT---- ---------- ----GATAGT AGTAATAATA
3D7                 .......... .......... .......... .......... ......---- ---------- ----...... ..........
RO33                .......... .......... .......... .......... ...------- ---------- ----...... ..........
Palo Alto           .......... .......... .......... .......... ......AAT- ---------- ----...... ..........
FCR3                .......... .......... .......... .......... ......AAT- ---------- ----...... ..........
Wellcome            .......... .......... .......... .......... ......AAT- ---------- ----...... ..........
D6                  .......... .......... .......... .......... ---------- ---------- ----...... ..........
T996                .......... .......... .......... .......... ......AAT- ---------- ----...... ..........
T9102               .......... .......... .......... .......... ...------- ---------- ----...... ..........
K1                  .......... .......... .......... .......... ......AATA ATAATAATAA TAAT...... ..........
Dd2                 .......... .......... .......... .......... ...------- ---------- ----...... ..........
D10                 .......... .......... .......... .......... ......---- ---------- ----...... ..........
FCC2                .......... .......... .......... .......--- ---------- ---------- ----...... ..........
HB3                 .......... .......... .......... .......... ...------- ---------- ----...... ..........
7G8                 .......... .......... .......... .......... ...------- ---------- ----...... ..........
P.reichenowi        .......... .......... .......... .......... ......---- ---------- ---------- ----------

                         3290       3300       3310       3320       3330       3340       3350       3360
                    ....|....| ....|....| ....|....| ....|....| ....|....| ....|....| ....|....| ....|....|
PF13_0348           ATAGTAGTAA TAATAAT--- ---------- -----AGTAA T--------- ---------- ---------- ----------
3D7                 .......... .......--- ---------- -----..... .--------- ---------- ---------- ----------
RO33                .......... .......AGT AGTAATAATA AT---..... .--------- ---------- ---------- ----------
Palo Alto           .......... .......--- ---------- -----..... .--------- ---------- ---------- ----------
FCR3                .......... .......--- ---------- -----..... .--------- ---------- ---------- ----------
Wellcome            .......... .......--- ---------- -----..... .--------- ---------- ---------- ----------
D6                  .......... .......AGT AGTAATAATA AT---..... .--------- ---------- ---------- ----------
T996                .......... .......AGT AGTAATAATA AT---..... .--------- ---------- ---------- ----------
T9102               .......... .......AGT AGTAATAATA ATAGT..... .AATAATAGT AAT------- ---------- ----------
K1                  .......... .......AGT AGTAATAATA AT---..... .--------- ---------- ---------- ----------
Dd2                 .......... .......AGT AGTAATAATA ATAGT..... .AATAATAGT AAT------- ---------- ----------
D10                 .......... .......AGT AGTAATAATA AT---..... .--------- ---------- ---------- ----------
FCC2                .......... .......AGT AAT------- ---------- ---------- ---------- ---------- ----------
HB3                 .......... .......AGT AGTAATAATA AT---..... .--------- ---------- ---------- ----------
7G8                 .......... .......AGT AAT------- ---------- ---------- ---------- ---------- ----------
P.reichenowi        ---------- ---------- ---------- ---------- ---------- ---AGGGGAA GCCAAGAGAT CCATTTAAAT

                         3370       3380       3390       3400       3410       3420       3430       3440
                    ....|....| ....|....| ....|....| ....|....| ....|....| ....|....| ....|....| ....|....|
PF13_0348           ---------- ---------- ---------- ---------- ---------- ---------- ---------- ----------
3D7                 ---------- ---------- ---------- ---------- ---------- ---------- ---------- ----------
RO33                ---------- ---------- ---------- ---------- ---------- ---------- ---------- ----------
Palo Alto           ---------- ---------- ---------- ---------- ---------- ---------- ---------- ----------
FCR3                ---------- ---------- ---------- ---------- ---------- ---------- ---------- ----------
Wellcome            ---------- ---------- ---------- ---------- ---------- ---------- ---------- ----------
D6                  ---------- ---------- ---------- ---------- ---------- ---------- ---------- ----------
T996                ---------- ---------- ---------- ---------- ---------- ---------- ---------- ----------
T9102               ---------- ---------- ---------- ---------- ---------- ---------- ---------- ----------
K1                  ---------- ---------- ---------- ---------- ---------- ---------- ---------- ----------
Dd2                 ---------- ---------- ---------- ---------- ---------- ---------- ---------- ----------
D10                 ---------- ---------- ---------- ---------- ---------- ---------- ---------- ----------
FCC2                ---------- ---------- ---------- ---------- ---------- ---------- ---------- ----------
HB3                 ---------- ---------- ---------- ---------- ---------- ---------- ---------- ----------
7G8                 ---------- ---------- ---------- ---------- ---------- ---------- ---------- ----------
P.reichenowi        AAATGTGACA TTCTAAAGAA TGAAGAGTAC AATGTGTCGA ATGAATATAA GAATAAAAAG TTTATAGATA TGTCAAATAA

                         3450       3460       3470       3480       3490       3500       3510       3520
                    ....|....| ....|....| ....|....| ....|....| ....|....| ....|....| ....|....| ....|....|
PF13_0348           ---------- ---------- ---------- ---------- ---------- ---------- -TGTGTTGAT AGGAGTGATA
3D7                 ---------- ---------- ---------- ---------- ---------- ---------- -......... ..........
RO33                ---------- ---------- ---------- ---------- ---------- ---------- -......... ..........
Palo Alto           ---------- ---------- ---------- ---------- ---------- ---------- -......... ..........
FCR3                ---------- ---------- ---------- ---------- ---------- ---------- -......... ..........
Wellcome            ---------- ---------- ---------- ---------- ---------- ---------- -......... ..........
D6                  ---------- ---------- ---------- ---------- ---------- ---------- -......... ..........
T996                ---------- ---------- ---------- ---------- ---------- ---------- -......... ..........
T9102               ---------- ---------- ---------- ---------- ---------- ---------- -......... ..........
K1                  ---------- ---------- ---------- ---------- ---------- ---------- -......... ..........
Dd2                 ---------- ---------- ---------- ---------- ---------- ---------- -......... ..........
D10                 ---------- ---------- ---------- ---------- ---------- ---------- -......... ..........
FCC2                ---------- ---------- ---------- ---------- ---------- ---------- -......... ..........
HB3                 ---------- ---------- ---------- ---------- ---------- ---------- -......... ..........
7G8                 ---------- ---------- ---------- ---------- ---------- ---------- -......... ..........
P.reichenowi        AAATGGTGCA AATGTTTGTG TGAATATAAA TAATATACAA GATAATAGAA ATAATAATAA TAA....... ..........
```

```
                                     3530       3540       3550       3560       3570       3580       3590       3600
                           ....|....| ....|....| ....|....| ....|....| ....|....| ....|....| ....|....| ....|....|
PF13_0348                  ACAAAAGTTA CAATGATAAA GACGATTCGA ATTCCTTAAA AAATAAAAAA TCAGGAGACC AATTAAGTGA ATGCACAGAA
3D7                        .......... .......... .......... .......... .......... .......... .......... ..........
RO33                       .......... .......... .......... .......... .......... .......... .......... ..........
Palo Alto                  .......... .......... .......... .......... .......... .......... .......... ..........
FCR3                       .......... .......... .......... .......... .......... .......... .........C ..........
Wellcome                   .......... .......... .......... .......... .......... .......... .........C ..........
D6                         .......... .......... .......... .......... .......... .......... .......... ..........
T996                       .......... .......... .......... .......... .......... .......... .......... ..........
T9102                      .......... .......... .......... .......... .......... .......... .......... ..........
K1                         .......... .......... .......... .......... .......... .......... .......... ..........
Dd2                        .......... .......... .......... .......... .......... .......... .......... ..........
D10                        .......... .......... .......... .......... .......... .......... .......... ..........
FCC2                       .......... .......... .......... .......... .......... .......... .......... ..........
HB3                        .......... .......... .......... .......... .......... .......... .......... ..........
7G8                        .......... .......... .......... .......... .......... .......... .......... ..........
P.reichenowi               .......... .......... .......... .......... .......... .......... ......A... .....T....

                                     3610       3620       3630       3640       3650       3660       3670       3680
                           ....|....| ....|....| ....|....| ....|....| ....|....| ....|....| ....|....| ....|....|
PF13_0348                  ATATACGAAA AGAAAAACAA ATTGACTAAT GAACAAAATT GTTGTGATAA CAGTTCATCA GAAAAAAATA TATCTAATAT
3D7                        .......... .......... .......... .......... .......... .......... .......... ..........
RO33                       .......... .......... .......... .......... .......... .......... .......... ..........
Palo Alto                  .......... .......... .......... .......... .......... .......... .......... ..........
FCR3                       .......... .......... .......... .......... .......... .......... .......... ..........
Wellcome                   .......... .......... .......... .......... .......... .......... .......... ..........
D6                         .......... .......... .......... .......... .......... .......... .......... ..........
T996                       .......... .......... .......... .......... .......... .......... .......... ..........
T9102                      .......... .......... .......... .......... .......... .......... .......... ..........
K1                         .......... .......... .......... .......... .......... .......... .......... ..........
Dd2                        .......... .......... .......... .......... .......... .......... .......... ..........
D10                        .......... .......... .......... .......... .......... .......... .......... ..........
FCC2                       .......... .......... .......... .......... .......... .......... .......... ..........
HB3                        .......... .......... .......... .......... .......... .......... .......... ..........
7G8                        .......... .......... .......... .......... .......... .......... .......... ..........
P.reichenowi               .......... .......... .......... .......... C........T A........C ........A. ..........

                                     3690       3700       3710       3720       3730       3740       3750       3760
                           ....|....| ....|....| ....|....| ....|....| ....|....| ....|....| ....|....| ....|....|
PF13_0348                  GAAACCGAAT TATATAATAG ATAAAGACTA TAATAATAAT GAAGATAATA CGATTCTGAG TAAACAAACA TATTTAATAG
3D7                        .......... .......... .......... .......... .......... .......... .......... ..........
RO33                       .......... .......... .......... .......... .......... .......... .......... ..........
Palo Alto                  .......... .......... .......... .......... .......... .......... .......... ..........
FCR3                       .......... .......... .......... .......... .......... .......... .......... ..........
Wellcome                   .......... .......... .......... .......... .......... .......... .......... ..........
D6                         .......... .......... .......... .......... .......... .......... .......... ..........
T996                       .......... .......... .......... .......... .......... .......... .......... ..........
T9102                      .......... .......... .......... .......... .......... .......... .......... ..........
K1                         .......... .......... .......... .......... .......... .......... .......... ..........
Dd2                        .......... .......... .......... .......... .......... .......... .......... ..........
D10                        .......... .......... .......... .......... .......... .......... .......... ..........
FCC2                       .......... .......... .......... .......... .......... .......... .......... ..........
HB3                        .......... .......... .......... .......... .......... .......... .......... ..........
7G8                        .......... .......... .......... .......... .......... .......... .......... ..........
P.reichenowi               C....A.... .......... ......C... .G........ .......... ..G....... .......... ..........

                                     3770       3780       3790       3800       3810       3820       3830       3840
                           ....|....| ....|....| ....|....| ....|....| ....|....| ....|....| ....|....| ....|....|
PF13_0348                  AAAGCAAGAA TATAGATAAA GATAAAAATA ATGATATAAA TAATGATATA AATAATAATT GTAGTAATAA TAATAATAAT
3D7                        .......... .......... .......... .......... .......... .......... .......... ..........
RO33                       .......... .......... .......... .......... .......... .......... .......... ..........
Palo Alto                  .......... .......... .......... .......... .......... .......... .......... ..........
FCR3                       .......... .......... .......... .......... .......... .......... .......... ..........
Wellcome                   .......... .......... .......... .......... .......... .......... .......... ..........
D6                         .......... .......... .......... .......... .......... .......... .......... ..........
T996                       .......... .......... .......... .......... .......... .......... .......... ..........
T9102                      .......... .......... .......... .......... .......... .......... .......... ..........
K1                         .......... .......... .......... .......... .......... .......... .......... ..........
Dd2                        .......... .......... .......... .......... .......... .......... .......... ..........
D10                        .......... .......... .......... .......... .......... .......... .......... ..........
FCC2                       .......... .......... .......... .......... .......... .......... .......... ..........
HB3                        .......... .......... .......... .......... .......... .......... .......... ..........
7G8                        .......... .......... .......... .......... .......... .......... .......... ..........
P.reichenowi               .......... .........T .....G.... TA....ATG. ...GA...AT G...G....A A..A.....G ..GC......
```

```
                         3850       3860       3870       3880       3890       3900       3910       3920
                 ....|....| ....|....| ....|....| ....|....| ....|....| ....|....| ....|....| ....|....|
PF13_0348        AATAATAATT GTGATGATAG TAATTGCTTG AAGAGCATTG AGAATAGAAA TAATAATAGA GATATTAAAA ATAATAAGAT
3D7              .......... .......... .......... .......... .......... .......... .......... ..........
RO33             .......... .......... .......... .......... .......... .......... .......... ..........
Palo Alto        .......... .......... .......... .......... .......... .......... .......... ..........
FCR3             ......---. .......... .......... .......... .......... .......... .......... ..........
Wellcome         ......---. .......... .......... .......... .......... .......... .......... ..........
D6               .......... .......... .......... .......... .......... .......... .......T.. ..........
T996             ......---. .......... .......... .......... .......... .......... .......... ..........
T9102            .......... .......... .......... .......... .......... .......... .......... ..........
K1               .......... .......... .......... .......... .......... .......... .......... ..........
Dd2              .......... .......... .......... .......... .......... .......... .......... ..........
D10              ......---. .......... .......... .......... .......... .......... .......... ..........
FCC2             ......---. .......... .......... .......... .......... .......... .......... ..........
HB3              .......... .......... .......... .......... .......... .......... .......... ..........
7G8              .......... .......... .......... .......... .......... .......... .......... ..........
P.reichenowi     .......... ...G...... ....A..... ......T... .A...A.... ........T. .......... .G........

                         3930       3940       3950       3960       3970       3980       3990       4000
                 ....|....| ....|....| ....|....| ....|....| ....|....| ....|....| ....|....| ....|....|
PF13_0348        TGAAGGAGAA AGCACTGATT CTTTATACAC CTCTAGTTCA GAAGATGAAT ATAATGATAG ACATGATAAT ATAAAGAAAA
3D7              .......... .......... .......... .......... .......... .......... .......... ..........
RO33             .......... .......... .......... .......... .......... .......... .......... ..........
Palo Alto        .......... .......... .......... .......... .......... .......... .......... ..........
FCR3             .......... .......... .......... .......... .......... .......... .......... ..........
Wellcome         .......... .......... .......... .......... .......... .......... .......... ..........
D6               .......... .......... .......... .......... .......... .......... .......... ..........
T996             .......... .......... .......... .......... .......... .......... .......... ..........
T9102            .......... .......... .......... .......... .......... .......... .......... ..........
K1               .......... .......... .......... .......... .......... .......... .......... ..........
Dd2              .......... .......... .......... .......... .......... .......... .......... ..........
D10              .......... .......... .......... .......... .......... .......... .......... ..........
FCC2             .......... .......... .......... .......... .......... .......... .......... ..........
HB3              .......... .......... .......... .......... .......... .......... .......... ..........
7G8              .......... .......... .......... .......... .......... .......... .......... ..........
P.reichenowi     .......... .......... .......... ...C...... ..G....... .C........ .......... .....A....

                         4010       4020       4030       4040       4050       4060       4070       4080
                 ....|....| ....|....| ....|....| ....|....| ....|....| ....|....| ....|....| ....|....|
PF13_0348        TAAAAAAGAG AACAGATATT GAAAATGAAA TTATTTTATT AAAAAATATA AGACCTTTTC CAACTATTTA TTGGTTAATA
3D7              .......... .......... .......... .......... .......... .......... .......... ..........
RO33             .......... .......... .......... .......... .......... .......... .......... ..........
Palo Alto        .......... .......... .......... .......... .......... .......... .......... ..........
FCR3             .......... .......... .......... .......... .......... .......... .......... ..........
Wellcome         .......... .......... .......... .......... .......... .......... .......... ..........
D6               .......... .......... .......... .......... .......... .......... .......... ..........
T996             .......... .......... .......... .......... .......... .......... .......... ..........
T9102            .......... .......... .......... .......... .......... .......... .......... ..........
K1               .......... .......... .......... .......... .......... .......... .......... ..........
Dd2              .......... .......... .......... .......... .......... .......... .......... ..........
D10              .......... .......... .......... .......... .......... .......... .......... ..........
FCC2             .......... .......... .......... .......... .......... .......... .......... ..........
HB3              .......... .......... .......... .......... .......... .......... .......... ..........
7G8              .......... .......... .......... .......... .......... .......... .......... ..........
P.reichenowi     .......A.. .........A .......... .......... .........T .......... .......... ..........

                         4090       4100       4110       4120       4130       4140       4150       4160
                 ....|....| ....|....| ....|....| ....|....| ....|....| ....|....| ....|....| ....|....|
PF13_0348        AATAAAAATA TATGTGGATA TATATCTCAT TTGGAAAAAA TAAATATTAT AAAAAATATT GAAAATTTTA TTAATCATCC
3D7              .......... .......... .......... .......... .......... .......... .......... ..........
RO33             .......... .......... .......... .......... .......... .......... .......... ..........
Palo Alto        .......... .......... .......... .......... .......... .......... .......... ..........
FCR3             .......... .......... .......... .......... .......... .......... .......... ..........
Wellcome         .......... .......... .......... .......... .......... .......... .......... ..........
D6               .......... .......... .......... .......... .......... .......... .......... ..........
T996             .......... .......... .......... .......... .......... .......... .......... ..........
T9102            .......... .......... .......... .......... .......... .......... .......... ..........
K1               .......... .......... .......... .......... .......... .......... .......... ..........
Dd2              .......... .......... .......... .......... .......... .......... .......... ..........
D10              .......... .......... .......... .......... .......... .......... .......... ..........
FCC2             .......... .......... .......... .......... .......... .......... .......... ..........
HB3              .......... .......... .......... .......... .......... .......... .......... ..........
7G8              .......... .......... .......... .......... .......... .......... .......... ..........
P.reichenowi     .......... .......... .......... .......... .......... ......C... .......... ..........
```

```
                               4170       4180       4190       4200       4210       4220       4230       4240
                         ....|....| ....|....| ....|....| ....|....| ....|....| ....|....| ....|....| ....|....|
PF13_0348                AGGAGAAGAA TTTAAATTAT TAAGATATTA TTTAATATAT GATCATTTAA AATATATAGT TATACGTTTA AGACATATAC
3D7                      .......... .......... .......... .......... .......... .......... .......... ..........
RO33                     .......... .......... .......... .......... .......... .......... .......... ..........
Palo Alto                .......... .......... .......... .......... .......... .......... .......... ..........
FCR3                     .......... .......... .......... .......... .......... .......... .......... ..........
Wellcome                 .......... .......... .......... .......... .......... .......... .......... ..........
D6                       .......... .......... .......... .......... .......... .......... .......... ..........
T996                     .......... .......... .......... .......... .......... .......... .......... ..........
T9102                    .......... .......... .......... .......... .......... .......... .......... ..........
K1                       .......... .......... .......... .......... .......... .......... .......... ..........
Dd2                      .......... .......... .......... .......... .......... .......... .......... ..........
D10                      .......... .......... .......... .......... .......... .......... .......... ..........
FCC2                     .......... .......... .......... .......... .......... .......... .......... ..........
HB3                      .......... .......... .......... .......... .......... .......... .......... ..........
7G8                      .......... .......... .......... .......... .......... .......... .......... ..........
P.reichenowi             ...T...... .......... .......... .......... .......... .......... .......... ..........

                               4250       4260       4270       4280       4290       4300       4310       4320
                         ....|....| ....|....| ....|....| ....|....| ....|....| ....|....| ....|....| ....|....|
PF13_0348                ATAAAAGAAT ACTTATCTTT TTTTATAACT ATTTTTTAAA CAGTGAATAT TTCATATCTC AACATAAACA AAATAATCAT
3D7                      .......... .......... .......... .......... .......... .......... .......... ..........
RO33                     .......... .......... .......... .......... .......... .......... .......... ..........
Palo Alto                .......... .......... .......... .......... .......... .......... .......... ..........
FCR3                     .......... .......... .......... .......... .......... .......... .......... ..........
Wellcome                 .......... .......... .......... .......... .......... .......... .......... ..........
D6                       .......... .......... .......... .......... .......... .......... .......... ..........
T996                     .......... .......... .......... .......... .......... .......... .......... ..........
T9102                    .......... .......... .......... .......... .......... .......... .......... ..........
K1                       .......... .......... .......... .......... .......... .......... .......... ..........
Dd2                      .......... .......... .......... .......... .......... .......... .......... ..........
D10                      .......... .......... .......... .......... .......... .......... .......... ..........
FCC2                     .......... .......... .......... .......... .......... .......... .......... ..........
HB3                      .......... .......... .......... .......... .......... .......... .......... ..........
7G8                      .......... .......... .......... .......... .......... .......... .......... ..........
P.reichenowi             .......... .......... .......... .......... .......... .......... .......... ..........

                               4330       4340       4350       4360       4370       4380       4390       4400
                         ....|....| ....|....| ....|....| ....|....| ....|....| ....|....| ....|....| ....|....|
PF13_0348                CTATCAAATA TCGATCTATA TAAAAATAAT CCATTCTCAC AAATATTCGA AATCACACCT ATCATACAAT CATATTATGA
3D7                      .......... .......... .......... .......... .......... .......... .......... ..........
RO33                     .......... .......... .......... .......... .......... .......... .......... ..........
Palo Alto                .......... .......... .......... .......... .......... .......... .......... ..........
FCR3                     .......... .......... .......... .......... .......... .......... .......... ..........
Wellcome                 .......... .......... .......... .......... .......... .......... .......... ..........
D6                       .......... .......... .......... .......... .......... .......... .......... ..........
T996                     .......... .......... .......... .......... .......... .......... .......... ..........
T9102                    .......... .......... .......... .......... .......... .......... .......... ..........
K1                       .......... .......... .......... .......... .......... .......... .......... ..........
Dd2                      .......... .......... .......... .......... .......... .......... .......... ..........
D10                      .......... .......... .......... .......... .......... .......... .......... ..........
FCC2                     .......... .......... .......... .......... .......... .......... .......... ..........
HB3                      .......... .......... .......... .......... .......... .......... .......... ..........
7G8                      .......... .......... .......... .......... .......... .......... .......... ..........
P.reichenowi             .....TT... .GC..GA... .......... ..C....... ..C....... ...G.....C ........T. ..........

                               4410       4420       4430       4440       4450       4460       4470       4480
                         ....|....| ....|....| ....|....| ....|....| ....|....| ....|....| ....|....| ....|....|
PF13_0348                AAAATATCCT ATAGATTTTA ATAGTATATG TGAAATATTA AGAAAAATTA ATACACTCAG AATTAAAGGT ATAGGAGGTA
3D7                      .......... .......... .......... .......... .......... .......... .......... .......
RO33                     .......... .......... .......... .......... .......... .......... .......... .......
Palo Alto                .......... .......... .......... .......... .......... .......... .......... .......
FCR3                     .......... .......... .......... .......... .......... .......... .......... .......
Wellcome                 .......... .......... .......... .......... .......... .......... .......... .......
D6                       .......... .......... .......... .......... .......... .......... .......... .......
T996                     .......... .......... .......... .......... .......... .......... .......... .......
T9102                    .......... .......... .......... .......... .......... .......... .......... .......
K1                       .......... .......... .......... .......... .......... .......... .......... .......
Dd2                      .......... .......... .......... .......... .......... .......... .......... .......
D10                      .......... .......... .......... .......... .......... .......... .......... .......
FCC2                     .......... .......... .......... .......... .......... .......... .......... .......
HB3                      .......... .......... .......... .......... .......... .......... .......... .......
7G8                      .......... .......... .......... .......... .......... .......... .......... .......
P.reichenowi             .......... .......C.. .......... .......... .......... .......... .......... ..........
```

```
                                    4490      4500      4510      4520      4530      4540      4550      4560
                       ....|....| ....|....| ....|....| ....|....| ....|....| ....|....| ....|....| ....|....|
PF13_0348              TATCAAACTT TCTAACTTTA AAATGTATAC ACTTATATTT TGCATCCCAT TTATCTTATC CAAATACTGT TGGTTATATA
3D7
RO33
Palo Alto
FCR3
Wellcome
D6
T996
T9102
K1
Dd2
D10
FCC2
HB3
7G8
P.reichenowi           .C........ ......C..G .......... .......... .......... .......... .......A.. ..........

                                    4570      4580
                       ....|....| ....|....| ....
PF13_0348              ATAGAGGAAT ATTTTAAGTC ATAA
3D7
RO33
Palo Alto
FCR3
Wellcome
D6
T996
T9102
K1
Dd2
D10
FCC2
HB3
7G8
P.reichenowi           ..T....... .
```

PF10_0144

```
                                      10        20        30        40        50        60        70        80
                       ....|....| ....|....| ....|....| ....|....| ....|....| ....|....| ....|....| ....|....|
PF10-0144              ATGTATAAAT TTAAATTTAA ATTAAGGAAC ATTTACAAAA TTCATTTCAA GCCTTATATT AACATGAATG CTCCTCCTAA
3D7                             . .......... .......... .......... .......... .......... .......... ..........
RO33                            . .......... .......... .......... .......... .......... .......... ..........
PALO ALTO                       . .......... .......... .......... .......... .......... .......... ..........
FCR3                            . .......... .......... .......... .......... .......... .......... ..........
WELLCOME                        . .......... .......... .......... .......... .......... .......... ..........
D6                              . .......... .......... .......... .......... .......... .......... ..........
T996                            . .......... .......... .......... .......... .......... .......... ..........
T9102                           . .......... .......... .......... .......... .......... .......... ..........
K1                              . .......... .......... .......... .......... .......... .......... ..........
Dd2                             . .......... .......... .......... .......... .......... .......... ..........
D10                             . .......... .......... .......... .......... .......... .......... ..........
FCC2                            . .......... .......... .......... .......... .......... .......... ..........
HB3                             . .......... .......... .......... .......... .......... .......... ..........
7G8                             . .......... .......... .......... .......... .......... .......... ..........
P.reichenowi                    . .......... .......... .......... ...T...... .......... ..T......A ..........

                                      90       100       110       120       130       140       150       160
                       ....|....| ....|....| ....|....| ....|....| ....|....| ....|....| ....|....| ....|....|
PF10_144               TTTTGACATA CATCAGGTTA AGAAATTAGG GAAATTAGGT GCAACTATAG GAGCAATAAT AGGTGTAACT TCATTTGGTA
3D7                    .......... .......... .......... .......... .......... .......... .......... ..........
RO33                   .......... .......... .......... .......... .......... .......... .......... ..........
PALO ALTO              .......... .......... .......... ....A..... .......... .......... .......... ..........
FCR3                   .......... .......... .......... .......... .......... .......... .......... ..........
WELLCOME               .......... .......... .......... .......... .......... .......... .......... ..........
D6                     .......... .......... .......... .......... .......... .......... .......... ..........
T996                   .......... .......... .......... .......... .......... .......... .......... ..........
T9102                  .......... .......... .......... .......... .......... .......... .......... ..........
K1                     .......... .......... .......... .......... .......... .......... .......... ..........
Dd2                    .......... .......... .......... .......... .......... .......... .......... ..........
D10                    .......... .......... .......... .......... .......... .......... .......... ..........
FCC2                   .......... .......... .......... .......... .......... .......... .......... ..........
HB3                    .......... .......... .......... .......... .......... .......... .......... ..........
7G8                    .......... .......... .......... .......... .......... .......... .......... ..........
P.reichenowi           .......... .......... .......... .......... .......... .......... .......... ..........
```

```
                           170        180        190        200        210        220        230        240
                    ....|....| ....|....| ....|....| ....|....| ....|....| ....|....| ....|....| ....|....|
PF10_144            GTTGGTTTTT TAAAAATAGC TTATATAATG TAGAGGCGGG GAAGCGAGCT ATAAAATATA ATAGAATATT TGGCTTGTCA
3D7                 .......... .......... .......... .......... .......... .......... .......... ..........
RO33                .......... .......... .......... .......... .......... .......... .......... ..........
PALO ALTO           .......... .......... .......... .......T.. .......... .......... .......... ..........
FCR3                .......... .......... .......... .......... .......... .......... .......... ..........
WELLCOME            .......... .......... .......... .......... .......... .......... .......... ..........
D6                  .......... .......... .......... .......... .......... .......... .......... ..........
T996                .......... .......... .......... .......... .......... .......... .......... ..........
T9102               .......... .......... .......... .......... .......... .......... .......... ..........
K1                  .......... .......... .......... .......... ...A...... .......... .......... ..........
Dd2                 .......... .......... .......... .......... .......... .......... .......... ..........
D10                 .......... .......... .......... .......... ...A...... .......... .......... ..........
FCC2                .......... .......... .......... .......... .......... .......... .......... ..........
HB3                 .......... .......... .......... .......... .......... .......... .......... ..........
7G8                 .......... .......... .......... .......... .......... .......... .......... ..........
P.reichenowi        .......... .......... .......... .......... ...A...... .......... .......... ..........

                           250        260        270        280        290        300        310        320
                    ....|....| ....|....| ....|....| ....|....| ....|....| ....|....| ....|....| ....|....|
PF10_144            AATAAAATAT ACGGTGAAGG TACGCACTTT TTAATACCAT TTTTTGAGAG ATCTATAATA TATGATGTAA GAACAAAACC
3D7                 .......... .......... .......... .......... .......... .......... .......... ..........
RO33                .......... .......... .......... .......... .......... .......... .......... ..........
PALO_ALTO           .......... .......... .......... .......... .......... .......... .......... ..........
FCR3                .......... .......... .......... .......... .......... .......... .......... ..........
WELLCOME            .......... .......... .......... .......... .......... .......... .......... ..........
D6                  .......... .......... .......... .......... .......... .......... .......... ..........
T996                .......... .......... .......... .......... .......... .......... .......... ..........
T9102               .......... .......... .......... .......... .......... .......... .......... ..........
K1                  .......... .......... .......... .......... .......... .......... .......... ..........
Dd2                 .......... .......... .......... .......... .......... .......... .......... ..........
D10                 .......... .......... .......... .......... .......... .......... .......... ..........
FCC2                .......... .......... .......... .......... .......... .......... .......... ..........
HB3                 .......... .......... .......... .......... .......... .......... .......... ..........
7G8                 .......... .......... .......... .......... .......... .......... .......... ..........
P.reichenowi        .......... .......... ...A...... .......... .......... .......... .......... ....C.....

                           330        340        350        360        370        380        390        400
                    ....|....| ....|....| ....|....| ....|....| ....|....| ....|....| ....|....| ....|....|
PF10_144            TAGAGTATTA ATGTCATTAA CAGGTTCTCG AGATCTACAA ATGGTAAACA TAACATGTCG TGTATTATCA AGACCAAATG
3D7                 .......... .......... .......... .......... .......... .......... .......... ..........
RO33                .......... .......... .......... .......... .......... .......... .......... ..........
Palo Alto           .......... .......... .......... .......... .......... .......... .......... ..........
FCR3                .......... .......... .......... .......... .......... .......... .......... ..........
Wellcome            .......... .......... .......... .......... .......... .......... .......... ..........
D6                  .......... .......... .......... .......... .......... .......... .......... ..........
T996                .......... .......... .......... .......... .......... .......... .......... ..........
T9102               .......... .......... .......... .......... .......... .......... .......... ..........
K1                  .......... .......... .......... .......... .......... .......... .......... ..........
Dd2                 .......... .......... .......... .......... .......... .......... .......... ..........
D10                 .......... .......... .......... .......... .......... .......... .......... ..........
FCC                 .......... .......... .......... .......... .......... .......... .......... ..........
HB3                 .......... .......... .......... .......... .......... .......... .......... ..........
7G8                 .......... .......... .......... .......... .......... .......... .......... ..........
P.reichenowi        .......... .......... .......... .......... .......... .......... .......... ..........

                           410        420        430        440        450        460        470        480
                    ....|....| ....|....| ....|....| ....|....| ....|....| ....|....| ....|....| ....|....|
PF10_144            AAAAGAAATT AGTAGAAATA TATAGAACCT TAGGAAAGGA ATATGATGAA AAAGTATTAC CATCAATTAT TAATGAAGTT
3D7                 .......... .......... .......... .......... .......... .......... .......... ..........
RO33                .......... .......... .......... .......... .......... .......... .......... ..........
Palo Alto           .......... .......... .......... .......... .......... .......... .......... ..........
FCR3                .......... .......... .......... .......... .......... .......... .......... ..........
Wellcome            .......... .......... .......... .......... .......... .......... .......... ..........
D6                  .......... .......... .......... .......... .......... .......... .......... ..........
T996                .......... .......... .......... .......... .......... .......... .......... ..........
T9102               ..C....... .......... .......... .......... .......... .......... .......... ..........
K1                  .......... .......... .......... .......... .......... .......... .......... ..........
Dd2                 .......... .......... .......... .......... .......... .......... .......... ..........
D10                 .......... .......... .......... .......... .......... .......... .......... ..........
FCC2                .......... .......... .......... .......... .......... .......... .......... ..........
HB                  .......... .......... .......... .......... .......... .......... .......... ..........
7G8                 .......... .......... .......... .......... .......... .......... .......... ..........
P.reichenowi        .......... .......... .......... .......... .......... .......... .......... ..........
```

```
                         490        500        510        520        530        540        550        560
                ....|....| ....|....| ....|....| ....|....| ....|....| ....|....| ....|....| ....|....|
PF10_144        TTAAAAAGTG TTGTAGCACA ATATAATGCT TCTCAATTAA TTACTCAAAG AGAAGTTGTA AGTAAATCAG TACGTGAACA
3D7             .......... .......... .......... .......... .......... .......... .......... ..........
RO33            .......... .......... .......... .......... .......... .......... .......... ..........
Palo Alto       .......... .......... .......... .......... .......... .......... .......... ..........
FCR3            .......... .......... .......... .......... .......... .......... .......... ..........
Wellcome        .......... .......... .......... .......... .......... .......... .......... ..........
D6              .......... .......... .......... .......... .......... .......... .......... ..........
T996            .......... .......... .......... .......... .......... .......... .......... ..........
T9102           .......... .......... .......... .......... .......... .......... .......... ..........
K1              .......... .......... .......... .......... .......... .......... .......... ..........
Dd2             .......... .......... .......... .......... .......... .......... .......... ..........
D10             .......... .......... .......... .......... .......... .......... .......... ..........
FCC2            .......... .......... .......... .......... .......... .......... .......... ..........
HB3             .......... .......... .......... .......... .......... .......... .......... ..........
7G8             .......... .......... .......... .......... .......... .......... .......... ..........
P.reichenowi    .......... .......... .......... .....G.... .......... .......... .......... ..........

                         570        580        590        600        610        620        630        640
                ....|....| ....|....| ....|....| ....|....| ....|....| ....|....| ....|....| ....|....|
PF10_144        ATTAGTACAA AGAGCAAAAG ATTTTAATAT ACTTCTTGAT GATGCATCTA TTACTCATTT GAGCTTTAGT AATGAATATG
3D7             .......... .......... .......... .......... .......... .......... .......... ..........
RO33            .......... .......... .......... .......... .......... .......... .......... ..........
Palo Alto       .......... .......... .......... .......... .......... .......... .......... ..........
FCR3            .......... .......... .......... .......... .......... .......... .......... ..........
Wellcome        .......... .......... .......... .......... .......... .......... .......... ..........
D6              .......... .......... .......... .......... .......... .......... .......... ..........
T996            .......... .......... .......... .......... .......... .......... .......... ..........
T9102           .......... .......... .......... .......... .......... .......... .......... ..........
K1              .......... .......... .......... .......... .......... .......... .......... ..........
Dd2             .......... .......... .......... .......... .......... .......... .......... ..........
D10             .......... .......... .......... .......... .......... .......... .......... ..........
FCC2            .......... .......... .......... .......... .......... .......... .......... ..........
HB3             .......... .......... .......... .......... .......... .......... .......... ..........
7G8             .......... .......... .......... .......... .......... .......... .......... ..........
P.reichenowi    .......... .......... .......... .......... .......... .......... .......... ..........

                         650        660        670        680        690        700        710        720
                ....|....| ....|....| ....|....| ....|....| ....|....| ....|....| ....|....| ....|....|
PF10_144        AAAAAGCTGT TGAAGCTAAG CAAGTTGCAC AACAAGAAGC AGAAAGAAGT AAATATGTTG TTCTTAAAGC AGAGCAAGAA
3D7             .......... .......... .......... .......... .......... .......... .......... ..........
RO33            .......... .......... .......... .......... .......... .......... .......... ..........
Palo Alto       .......... .......... .......... .......... .......... .......... .......... ..........
FCR3            .......... .......... .......... .......... .......... .......... .......... ..........
Wellcome        .......... .......... .......... .......... .......... .......... .......... ..........
D6              .......... .......... .......... .......... .......... .......... .......... ..........
T996            .......... .......... .......... .......... .......... .......... .......... ..........
T9102           .......... .......... .......... .......... .......... .......... .......... ..........
K1              .......... .......... .......... .......... .......... .......... .......... ..........
Dd2             .......... .......... .......... .......... .......... .......... .......... ..........
D10             .......... .......... .......... .......... .......... .......... .......... ..........
FCC2            .......... .......... .......... .......... .......... .......... .......... ..........
HB3             .......... .......... .......... .......... .......... .......... .......... ..........
7G8             .......... .......... .......... .......... .......... .......... .......... ..........
P.reichenowi    .......... .......... .......... .......... .......... .......... .......... ..........

                         730        740        750        760        770        780        790        800
                ....|....| ....|....| ....|....| ....|....| ....|....| ....|....| ....|....| ....|....|
PF10_144        AAAAAATCTA CAATTATTAA AGCACAAGGA GAAGCTGAAG TAGCCAAATT AATAGGTCTT GCTGTTAAGG ACAATCCTGC
3D7             .......... .......... .......... .......... .......... .......... .......... ..........
RO33            .......... .......... .......... .......... .......... .......... .......... ..........
Palo Alto       .......... .......... .......... .......... .......... .......... .......... ..........
FCR3            .......... .......... .......... .......... .......... .......... .......... ..........
Wellcome        .......... .......... .......... .......... .......... .......... .......... ..........
D6              .......... .......... .......... .......... .......... .......... .......... ..........
T996            .......... .......... .......... .......... .......... .......... .......... ..........
T9102           .......... .......... .......... .......... .......... .......... .......... ..........
K1              .......... .......... .......... .......... .......... .......... .......... ..........
Dd2             .......... .......... .......... .......... .......... .......... .......... ..........
D10             .......... .......... .......... .......... .......... .......... .......... ..........
FCC2            .......... .......... .......... .......... .......... .......... .......... ..........
HB3             .......... .......... .......... .......... .......... .......... .......... ..........
7G8             .......... .......... .......... .......... .......... .......... .......... ..........
P.reichenowi    .......... .......... .......... .......... .......... .......... .......... ..........
```

```
                        810        820        830        840        850        860        870        880
                ....|....| ....|....| ....|....| ....|....| ....|....| ....|....| ....|....| ....|....|
PF10_144        ATTTATGGAA TTAAAAAAAA TTGAATTGTC AAGAGAAGTT TCAAATATCA TATCAAAATG TCAAAATAAG GTTATGTTAC
3D7             .......... .......... .......... .......... .......... .......... .......... ..........
RO33            .......... .......... .......... .......... .......... .......... .......... ..........
Palo Alto       .......... .......... .......... .......... .......... .......... .......... ..........
FCR3            .......... .......... .......... .......... .......... .......... .......... ..........
Wellcome        .......... .......... .......... .......... .......... .......... .......... ..........
D6              .......... .......... .......... .......... .......... .......... .......... ..........
T996            .......... .......... .......... .......... .......... .......... .......... ..........
T9102           .......... .......... .......... .......... .......... .......... .......... ..........
K1              .......... .......... .......... .......... .......... .......... .......... ..........
Dd2             .......... .......... .......... .......... .......... .......... .......... ..........
D10             .......... .......... .......... .......... .......... .......... .......... ..........
FCC2            .......... .......... .......... .......... .......... .......... .......... ..........
HB3             .......... .......... .......... .......... .......... .......... .......... ..........
7G8             .......... .......... .......... .......... .......... .......... .......... ..........
P.reichenowi    .......... .......... .....C.... .......... .......... .......... .......... ..........

                        890        900        910
                ....|....| ....|....| ....|....| ....|
PF10_144        CTACGGATTC GTTGTTGATA AATTTTACAA AATAA
3D7             .......... .......... ...
RO33            .......... .......... ...
Palo Alto       .......... .......... ...
FCR3            .......... .......... ...
Wellcome        .......... .......... ...
D6              .......... .......... ...
T996            .......... .......... ...
T9102           .......... .......... ...
K1              .......... .......... ...
Dd2             .......... .......... ...
D10             .......... .......... ...
FCC2            .......... .......... ...
HB3             .......... .......... ...
7G8             .......... .......... ...
P.reichenowi    .......... .......... ...
```

PF14_0102 – RAP1

```
                         10         20         30         40         50         60         70         80
                ....|....| ....|....| ....|....| ....|....| ....|....| ....|....| ....|....| ....|....|
PF14_0102       ATGAGTTTCT ATTTGGGTAG CTTAGTAATA ATATTCCATG TACTCTTCCG TAATGTCGCT GATGGTATAA ATGTAAACGG
3D7                               .. .......... .......... .......... .......... .......... ..........
RO33                              .. .......... .......... .......... .......... .......... ..........
Palo Alto                         .. .......... .......... .......... .......... .......... ..........
FCR3                              .. .......... .......... .......... .......... .......... ..........
Wellcome                          .. .......... .......... .......... .......... .......... ..........
D6                                .. .......... .......... .......... .......... .......... ..........
T996                              .. .......... .......... .......... .......... .......... ..........
T9102                             .. .......... .......... .......... .......... .......... ..........
K1                                .. .......... .......... .......... .......... .......... ..........
Dd2                               .. .......... .......... .......... .......... .......... ..........
D10                               .. .......... .......... .......... .......... .......... ..........
FCC2                              .. .......... .......... .......... .......... .......... ..........
HB3                               .. .......... .......... .......... .......... .......... ..........
7G8                               .. .......... .......... .......... .......... .......... ..........
P.reichenowi    .......... .......... .......... .......... .......... .........A .......... ..........

                         90        100        110        120        130        140        150        160
                ....|....| ....|....| ....|....| ....|....| ....|....| ....|....| ....|....| ....|....|
PF14_0102       AGATAATAAT TATGGGAAAA CAATAATCAA TAATGATTTC AATTTTGATG ATTACAATTA TTGGACACCA ATAAATAAAA
3D7             .......... .......... .......... .......... .......... .......... .......... ..........
RO33            .......... .......... .......... .......... .......... .......... .......... ..........
Palo Alto       .......... .......... .......... .......... .......... .......... .......... ..........
FCR3            .......... .......... .......... .......... .......... .......... .......... ..........
Wellcome        .......... .......... .......... .......... .......... .......... .......... ..........
D6              .......... .......... .......... .......... .......... .......... .......... ..........
T996            .......... .......... .......... .......... .......... .......... .......... ..........
T9102           .......... .......... .......... .......... .......... .......... .......... ..........
K1              .......... .......... .......... .......... .......... .......... .......... ..........
Dd2             .......... .......... .......... .......... .......... .......... .......... ..........
D10             .......... .......... .......... .......... .......... .......... .......... ..........
FCC2            .......... .......... .......... .......... .......... .......... .......... ..........
HB3             .......... .......... .......... .......... .......... .......... .......... ..........
7G8             .......... .......... .......... .......... .......... .......... .......... ..........
P.reichenowi    .......... .......... .......TC. .......... .......... .......... .......... ..........
```

```
                      170       180       190       200       210       220       230       240
               ....|....| ....|....| ....|....| ....|....| ....|....| ....|....| ....|....| ....|....|
PF14_0102      AGGAATTTTT AAATTCCTAT GAAGATAAAT TTTCAAGTGA ATCCTTTTTA GAAAATAAAT CTAGTGTTGA TGATGGAAAT
3D7            .......... .......... .......... .......... .......... .......... .......... ..........
RO33           .......... .......... ......G... ......A... .......... .......... .......... ..........
Palo Alto      .......... .......... ......G... .......... .......... .......... .......... ..........
FCR3           .......... .......... ......G... .......... .......... .......... .......... ..........
Wellcome       .......... .......... ......G... .......... .......... .......... .......... ..........
D6             .......... .......... ...A..G... .......... .......... .......... .......... ..........
T996           .......... .......... ......G... .......... .......... .......... .......... ..........
T9102          .......... .......... ...A..G... .......... .......... .......... .......... ..........
K1             .......... .......... ......G... .......... .......... .......... .......... ..........
Dd2            .......... .......... ...A..G... .......... .......... .......... .......... ..........
D10            .......... .......... ......G... .......... .......... .......... .......... ..........
FCC2           .......... .......... ......G... .......... .......... .......... .......... ..........
HB3            .......... .......... .......... .......... .......... .......... .......... ..........
7G8            .......... .......... .......... .......... .......... .......... .......... ..........
P.reichenowi   .......... .......... ......G... .......... .......... .......... .......... ..........

                      250       260       270       280       290       300       310       320
               ....|....| ....|....| ....|....| ....|....| ....|....| ....|....| ....|....| ....|....|
PF14_0102      ATAAATTTAA CAGATACAAG TACATCAAAT AAAAGTTCTA AAAAAGGACA TGGTAGAAGT AGAGTAAGAT CAGCATCAGC
3D7            .......... .......... .......... .......... .......... .......... .......... ..........
RO33           .......... .......... .......... .......... .......... .......... .......... ..........
Palo Alto      .......... .......... .......... .......... .......... .......... .......... ..........
FCR3           .......... .......... .......... .......... .......... .......... .......... ..........
Wellcome       .......... .......... .......... .......... .......... .......... .......... ..........
D6             .......... .......... .......... .......... .......... .......... .......... ..........
T996           .......... .......... .......... .......... .......... .......... .......... ..........
T9102          .......... .......... .......... .......... .......... .......... .......... ..........
K1             .......... .......... .......... .......... .......... .......... .......... ..........
Dd2            .......... .......... .......... .......... .......... .......... .......... ..........
D10            .......... .......... .......... .......... .......... .......... .......... ..........
FCC2           .......... .......... .......... .......... .......... .......... .......... ..........
HB3            .......... .......... .......... .......... .......... .......... .......... ..........
7G8            .......... .......... .......... .......... .......... .......... .......... ..........
P.reichenowi   .......... .......... .G........ .......... G......... .......... .......... ..........

                      330       340       350       360       370       380       390       400
               ....|....| ....|....| ....|....| ....|....| ....|....| ....|....| ....|....| ....|....|
PF14_0102      TGCTGCAATT CTTGAAGAAG ATGATTCAAA AGATGATATG GAATTTAAAG CTTCTCCTTC AGTTGTTAAA ACATCTACTC
3D7            .......... .......... .......... .......... .......... .......... .......... ..........
RO33           .......... .......... .......... .......... .......... .......... .......... ..........
Palo Alto      .......... .......... .......... .......... .......... .......... .......... ..........
FCR3           .......... .......... .......... .......... .......... .......... .......... ..........
Wellcome       .......... .......... .......... .......... .......... .......... .......... ..........
D6             .......... .......... .......... .......... .......... .......... .......... ..........
T996           .......... .......... .......... .......... .......... .......... .......... ..........
T9102          .......... .......... .......... .......... .......... .......... .......... ..........
K1             .......... .......... .......... .......... .......... .......... .......... ..........
Dd2            .......... .......... .......... .......... .......... .......... .......... ..........
D10            .......... .......... .......... .......... .......... .......... .......... ..........
FCC2           .......... .......... .......... .......... .......... .......... .......... ..........
HB3            .......... .......... .......... .......... .......... .......... .......... ..........
7G8            .......... .......... .......... .......... .......... .......... .......... ..........
P.reichenowi   .......... .....G.... .......... .........T .......... .C........ .......... ..........

                      410       420       430       440       450       460       470       480
               ....|....| ....|....| ....|....| ....|....| ....|....| ....|....| ....|....| ....|....|
PF14_0102      CATCAGGTAC ACAGACATCT GGTTTAAAAT CATCTAGTCC ATCTAGTACA AAGTCATCAA GTCCATCAAA TGTAAAATCA
3D7            .......... .......... .......... .......... .......... .......... .......... ..........
RO33           .......... .......... .......... .......... .......... .......... .......... ..........
Palo Alto      .......... .......... ....C..... .......... .......... .......... .......... ..........
FCR3           .......... .......... .......... .......... .......... .......... .......... ..........
Wellcome       .......... .......... .......... .......... .......... .......... .......... ..........
D6             .......... .......... .......... .......... .....T.... .......... .......... ..........
T996           .......... .......... ....C..... .......... .......... .......... .......... ..........
T9102          .......... .......... .......... .......... .....T.... .......... .......... ..........
K1             .......... .......... .......... .......... .......... .......... .......... ..........
Dd2            .......... .......... .......... .......... .....T.... .......... .......... ..........
D10            .......... .......... .......... .......... .......... .......... .......... ..........
FCC2           .......... .......... .......... .......... .......... .......... .......... ..........
HB3            .......... .......... ....C..... .......... .......... .......... .......... ..........
7G8            .......... .......... ....C..... .......... .......... .......... .......... ..........
P.reichenowi   ..A....... ...T...... ....C..... .......... .......... ..A....... .......... .......A..
```

```
                            490        500        510        520        530        540        550        560
                     ....|....| ....|....| ....|....| ....|....| ....|....| ....|....| ....|....| ....|....|
PF14_0102            GCTAGTCCAC ATGGTGAATC TAATTCTTCT GAAGAAAGTA CTACTAAATC CTCAAAGAGA AGTGCTTCGG TTGCAGGTAT
3D7                  .......... .......... .......... .......... .......... .......... .......... ..........
RO33                 .......... .......... .......... .......... .......... .......... .......... ..........
Palo Alto            .......... .......... .......... .......... .......... .......... .......... ..........
FCR3                 .......... .......... .......... .......... .......... .......... .......... ..........
Wellcome             .......... .......... .......... .......... .......... .......... .......... ..........
D6                   .......... .......... .......... .......... .......... .......... .......... ..........
T996                 .......... .......... .......... .......... .......... .......... .......... ..........
T9102                .......... .......... .......... .......... .......... .......... .......... ..........
K1                   .......... .......... .......... .......... .......... .......... .......... ..........
Dd2                  .......... .......... .......... .......... .......... .......... .......... ..........
D10                  .......... .......... .......... .......... .......... .......... .......... ..........
FCC2                 .......... .......... .......... .......... .......... .......... .......... ..........
HB3                  .......... .......... .......... .......... .......... .......... .......... ..........
7G8                  .......... .......... .......... .......... .......... .......... .......... ..........
P.reichenowi         .......... .......... .......... .......... ..T....... .......... ........A. ..T.......

                            570        580        590        600        610        620        630        640
                     ....|....| ....|....| ....|....| ....|....| ....|....| ....|....| ....|....| ....|....|
PF14_0102            TGTAGGTGCC GACGAAGAAG CACCTCCTGC ACCAAAAAAC ACCCTCACTC CATTAGAAGA ATTATATCCT ACTAATGTTA
3D7                  .......... .......... .......... .......... .......... .......... .......... ..........
RO33                 .......... .......... .......... .......... .......... .......... .......... ..........
Palo Alto            .......... .......... .......... .......... .......... .......... .......... ..........
FCR3                 .......... .......... .......... .......... .......... .......... .......... ..........
Wellcome             .......... .......... .......... .......... .......... .......... .......... ..........
D6                   .......... .......... .......... .......... .......... .......... .......... ..........
T996                 .......... .......... .......... .......... .......... .......... .......... ..........
T9102                .......... .......... .......... .......... .......... .......... .......... ..........
K1                   .......... .......... .......... .......... .......... .......... .......... ..........
Dd2                  .......... .......... .......... .......... .......... .......... .......... ..........
D10                  .......... .......... .......... .......... .......... .......... .......... ..........
FCC2                 .......... .......... .......... .......... .......... .......... .......... ..........
HB3                  .......... .......... .......... .......... .......... .......... .......... ..........
7G8                  .......... .......... .......... .......... .......... .......... .......... ..........
P.reichenowi         .......... .......... T......... ......G... ........C. .......... .......... ..........

                            650        660        670        680        690        700        710        720
                     ....|....| ....|....| ....|....| ....|....| ....|....| ....|....| ....|....| ....|....|
PF14_0102            ATTTATTTAA CTATAAATAT TCATTAAACA ATATGGAAGA AAATATCAAT ATACTTAAAA ACGAAGGAGA TTTAGTTGCA
3D7                  .......... .......... .......... .......... .......... .......... .......... ..........
RO33                 .......... .......... .......... .......... .......... .......... .......... ..........
Palo Alto            .......... .......... .......... .......... .......... .......... .......... ..........
FCR3                 .......... .......... .......... .......... .......... .......... .......... ..........
Wellcome             .......... .......... .......... .......... .......... .......... .......... ..........
D6                   .......... .......... .......... .......... .......... .......... .......... ..........
T996                 .......... .......... .......... .......... .......... .......... .......... ..........
T9102                .......... .......... .......... .......... .......... .......... .......... ..........
K1                   .......... .......... .......... .......... .......... .......... .......... ..........
Dd2                  .......... .......... .......... .......... .......... .......... .......... ..........
D10                  .......... .......... .......... .......... .......... .......... .......... ..........
FCC2                 .......... .......... .......... .......... .......... .......... .......... ..........
HB3                  .......... .......... .......... .......... .......... .......... .......... ..........
7G8                  .......... .......... .......... .......... .......... .......... .......... ..........
P.reichenowi         .......... .......... .......... .......... .......... .......... ........C. A.........

                            730        740        750        760        770        780        790        800
                     ....|....| ....|....| ....|....| ....|....| ....|....| ....|....| ....|....| ....|....|
PF14_0102            CAAAAAGAAG AATTTGAATA TGATGAAAAT ATGGAAAAAG CTAAACAAGA CAAAAAAAAA GCACTTGAGA AAATAGGAAA
3D7                  .......... .......... .......... .......... .......... .......... .......... ..........
RO33                 .......... .......... .......... .......... .......... .......... .......... ..........
Palo Alto            .......... .......... .......... .......... .......... .......... .......... ..........
FCR3                 .......... .......... .......... .......... .......... .......... .......... ..........
Wellcome             .......... .......... .......... .......... .......... .......... .......... ..........
D6                   .......... .......... .......... .......... .......... .......... .......... ..........
T996                 .......... .......... .......... .......... .......... .......... .......... ..........
T9102                .......... .......... .......... .......... .......... .......... .......... ..........
K1                   .......... .......... .......... .......... .......... .......... .......... ..........
Dd2                  .......... .......... .......... .......... .......... .......... .......... ..........
D10                  .......... .......... .......... .......... .......... .......... .......... ..........
FCC2                 .......... .......... .......... .......... .......... .......... .......... ..........
HB3                  .......... .......... .......... .......... .......... .......... .......... ..........
7G8                  .......... .......... .......... .......... .......... .......... .......... ..........
P.reichenowi         .......... .......T.. .......... .......... .....G.... G..C...... .......... ..........
```

```
                                  810        820        830        840        850        860        870        880
                         ....|....| ....|....| ....|....| ....|....| ....|....| ....|....| ....|....| ....|....|
PF14_0102                AGAATCAGAC GAAGAACCTT TTATGTTTTC AGAAAATAAA TTTCTTGAAA ATCAAGTAAA AGAAAGAAAT GTTGCTGGAT
3D7                      .......... .......... .......... .......... .......... .......... .......... ..........
RO33                     .......... .......... .......... .......... .......... .......... .......... ..........
Palo Alto                .......... .......... .......... .......... .......... .......... .......... ..........
FCR3                     .......... ....C..... .......... .......... .......... .......... .......... ..........
Wellcome                 .......... ....C..... .......... .......... .......... .......... .......... ..........
D6                       .C........ .......... .......... .......... .......... .......... .......... ..........
T996                     .......... .......... .......... .......... .......... .......... .......... ..........
T9102                    .C........ .......... .......... .......... .......... .......... .......... ..........
K1                       .C........ .......... .......... .......... .......... .......... .......... ..........
Dd2                      .C........ .......... .......... .......... .......... .......... .......... ..........
D10                      .......... ....C..... .......... .......... .......... .......... .......... ..........
FCC2                     .C........ .......... .......... .......... .......... .......... .......... ..........
HB3                      .......... .......... .......... .......... .......... .......... .......... ..........
7G8                      .......... .......... .......... .......... .......... .......... .......... ..........
P.reichenowi             ....A..... .......... ........A. ...TG..... .......... .......... .......... ..........

                                  890        900        910        920        930        940        950        960
                         ....|....| ....|....| ....|....| ....|....| ....|....| ....|....| ....|....| ....|....|
PF14_0102                CCTTTTCTCG ATTTTTCAGT AAATTAAATC CTTTTAAGAA AGATGAAGTA ATAGAAAAAA CTGAAGTATC AAAGAAAACA
3D7                      .......... .......... .......... .......... .......... .......... .......... ..........
RO33                     .......... .......... .......... .......... .......... .......... .......... ..........
Palo Alto                .......... .......... .......... .......... .......... .......... .......... ..........
FCR3                     .......... .......... .......... .......... .......... .......... .......... ..........
Wellcome                 .......... .......... .......... .......... .......... .......... .......... ..........
D6                       .......... .......... .......... .......... .......... .......... .......... ..........
T996                     .......... .......... .......... .......... .......... .......... .......... ..........
T9102                    .......... .......... .......... .......... .......... .......... .......... ..........
K1                       .......... .......... .......... .......... .......... .......... .......... ..........
Dd2                      .......... .......... .......... .......... .......... .......... .......... ..........
D10                      .......... .......... .......... .......... .......... .......... .......... ..........
FCC2                     .......... .......... .......... .......... .......... .......... .......... ..........
HB3                      .......... .......... .......... .......... .......... .......... .......... ..........
7G8                      .......... .......... .......... .......... .......... .......... .......... ..........
P.reichenowi             .......... .......... .......... .C........ .......... .......... .......... ..........

                                  970        980        990       1000       1010       1020       1030       1040
                         ....|....| ....|....| ....|....| ....|....| ....|....| ....|....| ....|....| ....|....|
PF14_0102                TTTTCAGGTA TAGGTTTTAA TCTTACTGAG AAAGAAGCTA AAGTATTAGG TGTAGGTGTA ACCTATCAAG AATATCCAGA
3D7                      .......... .......... .......... .......... .......... .......... .......... ..........
RO33                     .......... .......... .......... .......... .......... .......... .......... ..........
Palo Alto                .......... .......... .......T.C .......... .......... .......... .......... ..........
FCR3                     .......... .......... .........C .......... .......... ........C. .......... ..........
Wellcome                 .......... .......... .........C .......... .......... ........C. .......... ..........
D6                       .......... .......... .........C .......... .......... .......... .......... ..........
T996                     .......... .......... .........A .......... .......... .......... .......... ..........
T9102                    .......... .......... .........C .......... .......... .......... .......... ..........
K1                       .......... .......... .........C .......... .......... ........C. .......... ..........
Dd2                      .......... .......... .........C .......... .......... .......... .......... ..........
D10                      .......... .......... .........C .......... .......... ........C. .......... ..........
FCC2                     .......... .......... .........C .......... .......... .......... .......... ..........
HB3                      .......... .......... .........A .......... .......... .......... .......... ..........
7G8                      .......... .......... .........C .......... .......... .......... .......... ..........
P.reichenowi             .A........ .......... .G...A...C .......... ..A....... ........C. .......... ..........

                                 1050       1060       1070       1080       1090       1100       1110       1120
                         ....|....| ....|....| ....|....| ....|....| ....|....| ....|....| ....|....| ....|....|
PF14_0102                AACCATGTTA TATAACTGTC CAAACAATTC TAATTTGTTT GATACTATAG AATCATTACA AGGAAGAGTA ATTGATATTA
3D7                      .......... .......... .......... .......... .......... .......... .......... ..........
RO33                     .......... .......... .......... .......... .......... .......... .......... ..........
Palo Alto                .......... .......... .......... .......... .......... .......... .......A.. ..........
FCR3                     .......... .......... .......... .......... .......... .......... .......A.. ..........
Wellcome                 .......... .......... .......... .......... .......... .......... .......A.. ..........
D6                       .......... .......... .......... .......... .......... .......... .......A.. ..........
T996                     .......... .......... .......... .......... .......... .......... .......A.. ..........
T9102                    .......... .......... .......... .......... .......... .......... .......A.. ..........
K1                       .......... .......... .......... .......... .......... .......... .......A.. ..........
Dd2                      .......... .......... .......... .......... .......... .......... .......A.. ..........
D10                      .......... .......... .......... .......... .......... .......... .......A.. ..........
FCC2                     .......... .......... .......... .......... .......... .......... .......A.. ..........
HB3                      .......... .......... .......... .......... .......... .......... .......A.. ..........
7G8                      .......... .......... .......... .......... .......... .......... .......... ..........
P.reichenowi             .......... .......... .......... .C........ .........A GC........ .......T.. ..........
```

```
                         1130       1140       1150       1160       1170       1180       1190       1200
                 ....|....| ....|....| ....|....| ....|....| ....|....| ....|....| ....|....| ....|....|
PF14_0102        AAAAAAGAGA AAGCATGATA TCAACAACTT TCGAACAACA AAAAGAATGT TTAAAAAATA TGGGTGTACT TGATCTTGAA
3D7              .......... .......... .......... .......... .......... .......... .......... ..........
RO33             .......... .......... .......... .......... .......... .......... .......... ..........
Palo Alto        .......... .......... .......... .......... .......... .......... .......... ..........
FCR3             .......... .......... .......... .......... .......... .......... .......... ..........
Wellcome         .......... .......... .......... .......... .......... .......... .......... ..........
D6               .......... .......... .......... .......... .......... .......... .......... ..........
T996             .......... .......... .......... .......... .......... .......... .......... ..........
T9102            .......... .......... .......... .......... .......... .......... .......... ..........
K1               .......... .......... .......... .......... .......... .......... .......... ..........
Dd2              .......... .......... .......... .......... .......... .......... .......... ..........
D10              .......... .......... .......... .......... .......... .......... .......... ..........
FCC2             .......... .......... .......... .......... .......... .......... .......... ..........
HB3              .......... .......... .......... .......... .......... .......... .......... ..........
7G8              .......... .......... .......... .......... .......... .......... .......... ..........
P.reichenowi     .......... ..AT...... .......... .......... .......... .......... .......... ..........

                         1210       1220       1230       1240       1250       1260       1270       1280
                 ....|....| ....|....| ....|....| ....|....| ....|....| ....|....| ....|....| ....|....|
PF14_0102        TTAAACGATA CACAATGTAA ATTTGGTACA TGTATAGGTA GCTTTGGAGA ACATCATCTT AGATTATACG AATTTGAGAA
3D7              .......... .......... .......... .......... .......... .......... .......... ..........
RO33             .......... .......... .......... .......... .......... .......... .......... ..........
Palo Alto        .......... .......... .......... .......... .......... .......... .......... ..........
FCR3             .......... .......... .......... .......... .......... .......... .......... ..........
Wellcome         .......... .......... .......... .......... .......... .......... .......... ..........
D6               .......... .......... .......... .......... .......... .......... .......... ..........
T996             .......... .......... .......... .......... .......... .......... .......... ..........
T9102            .......... .......... .......... .......... .......... .......... .......... ..........
K1               .......... .......... .......... .......... .......... .......... .......... ..........
Dd2              .......... .......... .......... .......... .......... .......... .......... ..........
D10              .......... .......... .......... .......... .......... .......... .......... ..........
FCC2             .......... .......... .......... .......... .......... .......... .......... ..........
HB3              .......... .......... .......... .......... .......... .......... .......... ..........
7G8              .......... .......... .......... .......... .......... .......... .......... ..........
P.reichenowi     .....A.... ..G....... .......... .......... .......... .......... .......... ..........

                         1290       1300       1310       1320       1330       1340       1350       1360
                 ....|....| ....|....| ....|....| ....|....| ....|....| ....|....| ....|....| ....|....|
PF14_0102        TGACTTATTA AAATTTCATC CAAATATTGA TTATTTAACT TTAGCTGATG GATATAAATT ACAAAAAAAT GATATATATG
3D7              .......... .......... .......... .......... .......... .......... .......... ..........
RO33             .........T .......... .......... .......... .......... .......... .......... ..........
Palo Alto        .........T .......... .......... .......... .......... .......... .......... C.........
FCR3             .........T .......... .......... .......... .......... .......... .......... C.........
Wellcome         .........T .......... .......... .......... .......... .......... .......... C.........
D6               .........T .......... .......... .......... .......... .......... .......... ..........
T996             .........T .......... .......... .......... .......... .......... .......... C.........
T9102            .........T .......... .......... .......... .......... .......... .......... C.........
K1               .........T .......... .......... .......... .......... .......... .......... C.........
Dd2              .........T .......... .......... .......... .......... .......... .......... C.........
D10              .........T .......... .......... .......... .......... .......... .......... C.........
FCC2             .........T .......... .......... .......... .......... .......... .......... C.........
HB3              .........T .......... .......... .......... .......... .......... .......... C.........
7G8              .........T .......... .......... .......... .......... .......... .......... ..........
P.reichenowi     .........T .......... .......... .......... .......... .......... .......... C.........

                         1370       1380       1390       1400       1410       1420       1430       1440
                 ....|....| ....|....| ....|....| ....|....| ....|....| ....|....| ....|....| ....|....|
PF14_0102        AATTATCCCA TGTAAACTTT TGCTTATTAA ATCCTAAAAC ATTAGAAGAA TTTTTAAAAA AAAAAGAAAT CAAGGATCTT
3D7              .......... .......... .......... .......... .......... .......... .......... ..........
RO33             .......... .......... .......... .......... .......... .......... .......... ..........
Palo Alto        .......... .......... .......... .......... ......G... .......... .......... ..........
FCR3             .......... .......... .......... .......... .......... .......... .......... ..........
Wellcome         .......... .......... .......... .......... .......... .......... .......... ..........
D6               .......... .......... .......... .......... .......... .......... .......... ..........
T996             .......... .......... .......... .......... .......... .......... .......... ..........
T9102            .......... .......... .......... .......... .......... .......... .......... ..........
K1               .......... .......... .......... .......... .......... .......... .......... ..........
Dd2              .......... .......... .......... .......... .......... .......... .......... ..........
D10              .......... .......... .......... .......... .......... .......... .......... ..........
FCC2             .......... .......... .......... .......... .......... .......... .......... ..........
HB3              .......... .......... .......... .......... .......... .......... .......... ..........
7G8              .......... .......... .......... .......... .......... .......... .......... ..........
P.reichenowi     .......... .......... .......... .......... .......... .......... .......... ..........
```

```
                    1450       1460       1470       1480       1490       1500       1510       1520
                ....|....| ....|....| ....|....| ....|....| ....|....| ....|....| ....|....| ....|....|
PF14_0102       ATGGGTGGTG ATGATCTTAT AAAATATAAA GAAAATTTTG ATAACTTTAT GAGTATATCT ATAACATGCC ATATTGAATC
3D7             .......... .......... .......... .......... .......... .......... .......... ..........
RO33            .......... .......... .......... .......... .......... .......... .......... ..........
Palo Alto       .......... .......... .......... .......... .......... .......... .......... ..........
FCR3            .......... .......... .......... .......... .......... .......... .......... ..........
Wellcome        .......... .......... .......... .......... .......... .......... .......... ..........
D6              .......... .......... .......... .......... .......... .......... .......... ..........
T996            .......... .......... .......... .......... .......... .......... .......... ..........
T9102           .......... .......... .......... .......... .......... .......... .......... ..........
K1              .......... .......... .......... .......... .......... .......... .......... ..........
Dd2             .......... .......... .......... .......... .......... .......... .......... ..........
D10             .......... .......... .......... .......... .......... .......... .......... ..........
FCC2            .......... .......... .......... .......... .......... .......... .......... ..........
HB3             .......... .......... .......... .......... .......... .......... .......... ..........
7G8             .......... .......... .......... .......... .......... .......... .......... ..........
P.reichenowi    .......... .......... .......... .......... .......... .......... .......... ..........

                    1530       1540       1550       1560       1570       1580       1590       1600
                ....|....| ....|....| ....|....| ....|....| ....|....| ....|....| ....|....| ....|....|
PF14_0102       TTTAATATAT GATGATATTG AAGCATCTCA AGATATTGCT GCTGTATTAA AAATTGCTAA AAGTAAATTA CATGTAATAA
3D7             .......... .......... .......... .......... .......... .......... .......... ..........
RO33            .......... .......... .......... .......... .......... .......... .......... ..........
Palo Alto       .......... .......... .......... .......... .......... .......... .......... ..........
FCR3            .......... .......... .......... .......... .......... .......... .......... ..........
Wellcome        .......... .......... .......... .......... .......... .......... .......... ..........
D6              .......... .......... .......... .......... .......... .......... .......... ..........
T996            .......... .......... .......... .......... .......... .......... .......... ..........
T9102           .......... .......... .......... .......... .......... .......... .......... ..........
K1              .......... .......... .......... .......... .......... .......... .......... ..........
Dd2             .......... .......... .......... .......... .......... .......... .......... ..........
D10             .......... .......... .......... .......... .......... .......... .......... ..........
FCC2            .......... .......... .......... .......... .......... .......... .......... ..........
HB3             .......... .......... .......... .......... .......... .......... .......... ..........
7G8             .......... .......... .......... .......... .......... .......... .......... ..........
P.reichenowi    .......... .......... .......... ....G..... .......... .......... .......... ..........

                    1610       1620       1630       1640       1650       1660       1670       1680
                ....|....| ....|....| ....|....| ....|....| ....|....| ....|....| ....|....| ....|....|
PF14_0102       CATCAGGTTT ATCATATAAA GCAAGAAAAT TAGTATATAA AATTTATAGT GAAATTCAAA AAAATCCAGA TGAACTCTAT
3D7             .......... .......... .......... .......... .......... .......... .......... ..........
RO33            .......... .......... .......... .......... .......... .......... .......... ..........
Palo Alto       .......... .......... .......... .......... .......... .......... .......... ..........
FCR3            .......... .......... .......... .......... .......... .......... .......... ..........
Wellcome        .......... .......... .......... .......... .......... .......... .......... ..........
D6              .......... .......... .......... .......... .......... .......... .......... ..........
T996            .......... .......... .......... .......... .......... .......... .......... ..........
T9102           .......... .......... .......... .......... .......... .......... .......... ..........
K1              .......... .......... .......... .......... .......... .......... .......... ..........
Dd2             .......... .......... .......... .......... .......... .......... .......... ..........
D10             .......... .......... .......... .......... .......... .......... .......... ..........
FCC2            .......... .......... .......... .......... .......... .......... .......... ..........
HB3             .......... .......... .......... .......... .......... .......... .......... ..........
7G8             .......... .......... .......... .......... .......... .......... .......... ..........
P.reichenowi    .......... .......... .......... .......... .......... .....A.... .......... .........C

                    1690       1700       1710       1720       1730       1740       1750       1760
                ....|....| ....|....| ....|....| ....|....| ....|....| ....|....| ....|....| ....|....|
PF14_0102       GAAAAATTAA CATGGATTTA TGATAATATC TATATGATTA AAAGATATTA TACTGCATAT GCTTTAGAAG GTGTCTGTTC
3D7             .......... .......... .......... .......... .......... .......... .......... ..........
RO33            .......... .......... .......... .......... .......... .......... .......... ..........
Palo Alto       .......... .......... .......... .......... .......... .......... .......... ..........
FCR3            .......... .......... .......... .......... .......... .......... .......... ..........
Wellcome        .......... .......... .......... .......... .......... .......... .......... ..........
D6              .......... .......... .......... .......... .......... .......... .......... ..........
T996            .......... .......... .......... .......... .......... .......... .......... ..........
T9102           .......... .......... .......... .......... .......... .......... .......... ..........
K1              .......... .......... .......... .......... .......... .......... .......... ..........
Dd2             .......... .......... .......... .......... .......... .......... .......... ..........
D10             .......... .......... .......... .......... .......... .......... .......... ..........
FCC2            .......... .......... .......... .......... .......... .......... .......... ..........
HB3             .......... .......... .......... .......... .......... .......... .......... ..........
7G8             .......... .......... .......... .......... .......... .......... .......... ..........
P.reichenowi    .......... .......... .......... ......C... .......... .......... .......... ..........
```

```
                                  1770       1780       1790       1800       1810       1820       1830       1840
                              ....|....| ....|....| ....|....| ....|....| ....|....| ....|....| ....|....| ....|....|
PF14_0102                     ATATCTTGAA CATGATAAAA GTCAAATGTA TACAGAATTA CATATTTATA ACAAAATAGT CGACTCTGTT CGTTATTATA
3D7                           .......... .......... .......... .......... .......... .......... .......... ..........
RO33                          .......... .......... .......... .......... .......... .......... .......... ..........
Palo Alto                     .......... .......... .......... .......... .......... .......... .......... ..........
FCR3                          .......... .......... .......... .......... .......... .......... .......... ..........
Wellcome                      .......... .......... .......... .......... .......... .......... .......... ..........
D6                            .......... .......... .......... .......... .......... .......... .......... ..........
T996                          .......... .......... .......... .......... .......... .......... .......... ..........
T9102                         .......... .......... .......... .......... .......... .......... .......... ..........
K1                            .......... .......... .......... .......... .......... .......... .......... ..........
Dd2                           .......... .......... .......... .......... .......... .......... .......... ..........
D10                           .......... .......... .......... .......... .......... .......... .......... ..........
FCC2                          .......... .......... .......... .......... .......... .......... .......... ..........
HB3                           .......... .......... .......... .......... .......... .......... .......... ..........
7G8                           .......... .......... .......... .......... .......... .......... .......... ..........
P.reichenowi                  ....A..... A......... C.......G. .......... .......... .......... .......... ..........

                                  1850       1860       1870       1880       1890       1900       1910       1920
                              ....|....| ....|....| ....|....| ....|....| ....|....| ....|....| ....|....| ....|....|
PF14_0102                     GTTCATGCTT TAAAAACGTT ATTGTTTATA ATGCTATCAT TTCTGGTATA CATGAAAAAA TAAAACATTT CTTAAAATTA
3D7                           .......... .......... .......... .......... .......... .......... .......... ..........
RO33                          .......... .......... .......... .......... .......... .......... .......... ..........
Palo Alto                     .......... .......... .......... .......... .......... .......... .......... ..........
FCR3                          .......... .......... .......... .......... .......... .......... .......... ..........
Wellcome                      .......... .......... .......... .......... .......... .......... .......... ..........
D6                            .......... .......... .......... .......... .......... .......... .......... ..........
T996                          .......... .......... .......... .......... .......... .......... .......... ..........
T9102                         .......... .......... .......... .......... .......... .......... .......... ..........
K1                            .......... .......... .......... .......... .......... .......... .......... ..........
Dd2                           .......... .......... .......... .......... .......... .......... .......... ..........
D10                           .......... .......... .......... .......... .......... .......... .......... ..........
FCC2                          .......... .......... .......... .......... .......... .......... .......... ..........
HB3                           .......... .......... .......... .......... .......... .......... .......... ..........
7G8                           .......... .......... .......... .......... .......... .......... .......... ..........
P.reichenowi                  .......... .......... .......... .......... G......... .......... .......... ..........

                                  1930       1940       1950       1960       1970       1980       1990       2000
                              ....|....| ....|....| ....|....| ....|....| ....|....| ....|....| ....|....| ....|....|
PF14_0102                     GTACCAAGAC ACAACTTTCT TTTGGATTAT CACTTTAATT CAATTTTTGA AAAAGAAATT AAACCAGCCA AAAAATATAG
3D7                           .......... .......... .......... .......... .......... .......... .......... ..........
RO33                          .......... .......... .......... .......... .......... .......... .......... ..........
Palo Alto                     .......... .......... .......... .......... .......... .......... .......... ..........
FCR3                          .......... .......... .......... .......... .......... .......... .......... ..........
Wellcome                      .......... .......... .......... .......... .......... .......... .......... ..........
D6                            .......... .......... .......... .......... .......... .......... .......... ..........
T996                          .......... .......... .......... .......... .......... .......... .......... ..........
T9102                         .......... .......... .......... .......... .......... .......... .......... ..........
K1                            .......... .......... .......... .......... .......... .......... .......... ..........
Dd2                           .......... .......... .......... .......... .......... .......... .......... ..........
D10                           .......... .......... .......... .......... .......... .......... .......... ..........
FCC2                          .......... .......... .......... .......... .......... .......... .......... ..........
HB3                           .......... .......... .......... .......... .......... .......... .......... ..........
7G8                           .......... .......... .......... .......... .......... .......... .......... ..........
P.reichenowi                  .......... .A........ .......... .......... .......... .......... .......T.. ..........

                                  2010       2020       2030       2040       2050       2060       2070       2080
                              ....|....| ....|....| ....|....| ....|....| ....|....| ....|....| ....|....| ....|....|
PF14_0102                     TACTTCACAT ATTTATTTTG ATCCAACTGT TGCATCATAT GCTTATTATA ATTTAGATAG AAGAACCATG GTTACTATTA
3D7                           .......... .......... .......... .......... .......... .......... .......... ..........
RO33                          .......... .......... .......... .......... .......... .......... .......... ..........
Palo Alto                     .......... .......... .......... .......... .......... .......... .......... ..........
FCR3                          .......... .......... .......... .......... .......... .......... .......... ..........
Wellcome                      .......... .......... .......... .......... .......... .......... .......... ..........
D6                            .......... .......... .......... .......... .......... .......... .......... ..........
T996                          .......... .......... .......... .......... .......... .......... .......... ..........
T9102                         .......... .......... .......... .......... .......... .......... .......... ..........
K1                            .......... .......... .......... .......... .......... .......... .......... ..........
Dd2                           .......... .......... .......... .......... .......... .......... .......... ..........
D10                           .......... .......... .......... .......... .......... .......... .......... ..........
FCC2                          .......... .......... .......... .......... .......... .......... .......... ..........
HB3                           .......... .......... .......... .......... .......... .......... .......... ..........
7G8                           .......... .......... .......... .......... .......... .......... .......... ..........
P.reichenowi                  .......... G......A.. .......... .......... .......... .C........ ......T... ..........
```

```
                               2090       2100       2110       2120       2130       2140       2150       2160
                      ....|....| ....|....| ....|....| ....|....| ....|....| ....|....| ....|....| ....|....|
PF14_0102             TTAATGATTA TTTCGAAGCA AAAAAAAAAG AATTAACCGT TATAGTATCT CGTATGAAAA CAGATATGCT CAGTCTTCAA
3D7                   .......... .......... .......... .......... .......... .......... .......... ..........
RO33                  .......... .......... .......... .......... .......... .......... .......... ..........
Palo Alto             .......... .......... .......... .......... .......... .......... .......... ..........
FCR3                  .......... .......... .......... .......... .......... .......... .......... ....A.....
Wellcome              .......... .......... .......... .......... .......... .......... .......... ....A.....
D6                    .......... .......... .......... .......... .......... .......... .......... ..........
T996                  .......... .......... .......... .......... .......... .......... .......... ..........
T9102                 .......... .......... .......... .......... .......... .......... .......... ..........
K1                    .......... .......... .......... .......... .......... .......... .......... ....A.....
Dd2                   .......... .......... .......... .......... .......... .......... .......... ..........
D10                   .......... .......... .......... .......... .......... .......... .......... ....A.....
FCC2                  .......... .......... .......... .......... .......... .......... .......... ....A.....
HB3                   .......... .......... .......... .......... .......... .......... .......... ..........
7G8                   .......... .......... .......... .......... .......... .......... .......... ..........
P.reichenowi          .......... .......... .......... .......... .......... .......... .......... ..........

                               2170       2180       2190       2200       2210       2220       2230       2240
                      ....|....| ....|....| ....|....| ....|....| ....|....| ....|....| ....|....| ....|....|
PF14_0102             AATGAAGAAT CAAAAATACC AAATGACAAA AGTGCAAATT CAAAACTAGC TACAAGATTA ATGAAAAAAT TTAAAGCTGA
3D7                   .......... .......... .......... .......... .......... .......... .......... ..........
RO33                  .......... .......... .......... .......... .......... .......... .......... ..........
Palo Alto             .......... .......... .......... .......... .......... .......... .......... ..........
FCR3                  .......... .......... .......... .......... .......... .......... .......... ..........
Wellcome              .......... .......... .......... .......... .......... .......... .......... ..........
D6                    .......... .......... .......... .......... .......... .......... .......... ..........
T996                  .......... .......... .......... .......... .......... .......... .......... ..........
T9102                 .......... .......... .......... .......... .......... .......... .......... ..........
K1                    .......... .......... .......... .......... .......... .......... .......... ..........
Dd2                   .......... .......... .......... .......... .......... .......... .......... ..........
D10                   .......... .......... .......... .......... .......... .......... .......... ..........
FCC2                  .......... .......... .......... .......... .......... .......... .......... ..........
HB3                   .......... .......... .......... .......... .......... .......... .......... ..........
7G8                   .......... .......... .......... .......... .......... .......... .......... ..........
P.reichenowi          .....C.... .......... .........G .......... .......... .......... ....G..... ..........

                               2250       2260       2270       2280       2290       2300       2310       2320
                      ....|....| ....|....| ....|....| ....|....| ....|....| ....|....| ....|....| ....|....|
PF14_0102             AATCAGAGAT TTCTTCAAAG AAATGCGTAT ACAATATGCT AAATTAATAA ACATACGTTA CAGATCTCAC TTAAAGAAAA
3D7                   .......... .......... .......... .......... .......... .......... .......... ..........
RO33                  .......... .......... .......... .......... .......... .......... .......... ..........
Palo Alto             .......... .......... .......... .......... .......... .......... .......... ..........
FCR3                  .......... .......... .......... .......... .......... .......... .......... ..........
Wellcome              .......... .......... .......... .......... .......... .......... .......... ..........
D6                    .......T.. .......... .......... .......... .......... .......... .......... ..........
T996                  .......... .......... .......... .......... .......... .......... .......... ..........
T9102                 .......... .......... .......... .......... .......... .......... .......... ..........
K1                    .......... .......... .......... .......... .......... .......... .......... ..........
Dd2                   .......... .......... .......... .......... .......... .......... .......... ..........
D10                   .......... .......... .......... .......... .......... .......... .......... ..........
FCC2                  .......... .......... .......... .......... .......... .......... .......... ..........
HB3                   .......... .......... .......... .......... .......... .......... .......... ..........
7G8                   .......... .......... .......... .......... .......... .......... .......... ..........
P.reichenowi          .........C .......... .......... .......... ..G....... ....T..... ..A....... ..........

                               2330       2340
                      ....|....| ....|....| ....|....
PF14_0102             ACTACTTTGC CTTCAAGAGA TTAGATTAA
3D7                   .......... ....
RO33                  .......... ....
Palo Alto             .......... ....
FCR3                  .......... ....
Wellcome              .......... ....
D6                    .......... ....
T996                  .......... ....
T9102                 .......... ....
K1                    .......... ....
Dd2                   .......... ....
D10                   .......... ....
FCC2                  .......... ....
HB3                   .......... ....
7G8                   .......... ....
P.reichenowi          .......... .......... .........
```

PFE0080c – RAP2

```
                               10         20         30         40         50         60         70         80
                       ....|....| ....|....| ....|....| ....|....| ....|....| ....|....| ....|....| ....|....|
PFE0080c               ATGGGTTTAA AATTTTATGT ATTAGTTTTT CTTATTTTAT GTTTGAAGAA TGTTGTAAAA GGGGATAAGT GTGAAACTGA
3D7
RO33
Palo Alto
FCR3
Wellcome
D6
T996
T9102
K1
Dd2
D10
FCC2
HB3
7G8
P.reichenowi

                               90        100        110        120        130        140        150        160
                       ....|....| ....|....| ....|....| ....|....| ....|....| ....|....| ....|....| ....|....|
PFE0080c               ATTTTCAAAA TTATATCCGG AATCAAATTC TTTGACTGGT TTAATTTATG CACACACTGC AAATGTTCAT AAATTATCTA
3D7                                                            ... .......... .......... .......... ..........
RO33                                                           ... .......... .......... .C........ ..........
Palo Alto                                                      ... .......... .......... .C........ ..........
FCR3                                                           ... .......... .......... .C........ ..........
Wellcome                                                       ... .......... .......... .C........ ..........
D6                                                             ... .......... .......... .C........ ..........
T996                                                           ... .......... .......... .C........ ..........
T9102                                                          ... .......... .......... .C........ ..........
K1                                                             ... .......... .......... .C........ ..........
Dd2                                                            ... .......... .......... .......... ..........
D10                                                            ... .......... .......... .C........ ..........
FCC2                                                           ... .......... .......... .C........ ..........
HB3                                                            ... .......... .......... .......... ..........
7G8                                                            ... .......... .......... .C........ ..........
P.reichenowi                                                   ... .......... .........A .CG....... ..T.......

                              170        180        190        200        210        220        230        240
                       ....|....| ....|....| ....|....| ....|....| ....|....| ....|....| ....|....| ....|....|
PFE0080c               TGTGGGTTTA TTTTATTTAT AATCACTTTA GTAGTGCAGA TGAATTAATA AAATATTTAG AAAAAACCAA CATAAATACT
3D7                    .......... .......... .......... .......... .......... .......... .......... ..........
RO33                   .......... .......... .......... .......... .......... .......... .......... ..........
Palo Alto              .......... .......... .......... .......... .......... .......... .......... ..........
FCR3                   .......... .......... .......... .......... .......... .......... .......... ..........
Wellcome               .......... .......... .......... .......... .......... .......... .......... ..........
D6                     .......... .......... .......... .......... .......... .......... .......... ..........
T996                   .......... .......... .......... .......... .......... .......... .......... ..........
T9102                  .......... .......... .......... .......... .......... .......... .......... ..........
K1                     .......... .......... .......... .......... .......... .......... .......... ..........
Dd2                    .......... .......... .......... .......... .......... .......... .......... ..........
D10                    .......... .......... .......... .......... .......... .......... .......... ..........
FCC2                   .......... .......... .......... .......... .......... .......... .......... ..........
HB3                    .......... .......... .......... .......... .......... .......... .......... ..........
7G8                    .......... .......... .......... .......... .......... .......... .......... ..........
P.reichenowi           .......... ....C....C .......... .......... .......... .......... ......TG.. ..........

                              250        260        270        280        290        300        310        320
                       ....|....| ....|....| ....|....| ....|....| ....|....| ....|....| ....|....| ....|....|
PFE0080c               TTAGAAAATA GTGATCATAC ATGTTTTGCT AGAGCAGTTA CTTTATATTT GTTTTATTAC TATCTTAAGG ATATTAAGTC
3D7                    .......... .......... .......... .......... .......... .......... .......... ..........
RO33                   .......... .......... .......... .......... .......... .......... .......... ..........
Palo Alto              .......... .......... .......... .......... .......... .......... .......... ..........
FCR3                   .......... .......... .......... .......... .......... .......... .......... ..........
Wellcome               .......... .......... .......... .......... .......... .......... .......... ..........
D6                     .......... .......... .......... .......... .......... .......... .......... ..........
T996                   .......... .......... .......... .......... .......... .......... .......... ..........
T9102                  .......... .......... .......... .......... .......... .......... .......... ..........
K1                     .........G .......... .......... .......... .......... .......... .......... ..........
Dd2                    .......... .......... .......... .......... .......... .......... .......... ..........
D10                    .......... .......... .......... .......... .......... .......... .......... ..........
FCC2                   .......... .......... .......... .......... .......... .......... .......... ..........
HB3                    .......... .......... .......... .......... .......... .......... .......... ..........
7G8                    .......... .......... .......... .......... .......... .......... .......... ..........
P.reichenowi           .......G.. .G.......A .......... .......... .......... C......... .......... ..........
```

```
                                 330        340        350        360        370        380        390        400
                          ....|....| ....|....| ....|....| ....|....| ....|....| ....|....| ....|....| ....|....|
PFE0080c                  TATGTTAAGT ACAGATGATT ATCAATCATT TTTTAAGAAT AAATTCAAAG ATATTAATCC ATTGTTTATT AATGATTTTA
3D7                       .......... .......... .......... .......... .......... .......... .......... ..........
RO33                      .......... .......... .......... .......... .......... .......... .......... ..........
Palo Alto                 .......... .......... .......... .......... .......... .......... .......... ..........
FCR3                      .......... .......... .......... .......... .......... .......... .......... ..........
Wellcome                  .......... .......... .......... .......... .......... .......... .......... ..........
D6                        .......... .......... .......... .......... .......... .......... .......... ..........
T996                      .......... .......... .......... .......... .......... .......... .......... ..........
T9102                     .......... .......... .......... .......... .......... .......... .......... ..........
K1                        .......... .......... .......... .......... .......... .......... .......... ..........
Dd2                       .......... .......... .......... .......... .......... .......... .......... ..........
D10                       .......... .......... .......... .......... .......... .......... .......... ..........
FCC2                      .......... .......... .......... .......... .......... .......... .......... ..........
HB3                       .......... .......... .......... .......... .......... .......... .......... ..........
7G8                       .......... .......... .......... .......... .......... .......... .......... ..........
P.reichenowi              .......... .......... .......... .......... .......... .......... .......... ..........

                                 410        420        430        440        450        460        470        480
                          ....|....| ....|....| ....|....| ....|....| ....|....| ....|....| ....|....| ....|....|
PFE0080c                  TTTTAATTCT TAATGATAAG AAATTTATGG AAAATCTGGA TTTATATATA ATGAAAGAAT CTGAGAGAGA ACATTTGGTT
3D7                       .......... .......... .......... .......... .......... .......... .......... ..........
RO33                      .......... .......... .......... .......... .......... .......... .......... ..........
Palo Alto                 .......... .......... .......... .......... .......... .......... .......... ..........
FCR3                      .......... .......... .......... .......... .......... .......... .......... ..........
Wellcome                  .......... .......... .......... .......... .......... .......... .......... ..........
D6                        .......... .......... .......... .......... .......... .......... .......... ..........
T996                      .......... .......... .......... .......... .......... .......... .......... ..........
T9102                     .......... .......... .......... .......... .......... .......... .......... ..........
K1                        .......... .......... .......... .......... .......... .......... .......... ..........
Dd2                       .......... .......... .......... .......... .......... .......... .......... ..........
D10                       .......... .......... .......... .......... .......... .......... .......... ..........
FCC2                      .......... .......... .......... .......... .......... .......... .......... ..........
HB3                       .......... .......... .......... .......... .......... .......... .......... ..........
7G8                       .......... .......... .......... .......... .......... .......... .......... ..........
P.reichenowi              .......... C......... .......... .....T.... .......... ..T....... .......... ..........

                                 490        500        510        520        530        540        550        560
                          ....|....| ....|....| ....|....| ....|....| ....|....| ....|....| ....|....| ....|....|
PFE0080c                  ATAAAGAAGA ATCCATTTTT ACGTGTATTG AATAAAGCAT CAACTACTAC ACATGCAACA TATAAGTCTA ATCCATACTT
3D7                       .......... .......... .......... .......... .......... .......... .......... ..........
RO33                      .......... .......... .......... .......... .......... .......... .......... ..........
Palo Alto                 .......... .......... .......... .......... .......... .......... .......... ..........
FCR3                      .......... .......... .......... .......... .......... .......... .......... ..........
Wellcome                  .......... .......... .......... .......... .......... .......... .......... ..........
D6                        .......... .......... .......... .......... .......... .......... .......... ..........
T996                      .......... .......... .......... .......... .......... .......... .......... ..........
T9102                     .......... .......... .......... .......... .......... .......... .......... ..........
K1                        .......... .......... .......... .......... .......... .......... .......... ..........
Dd2                       .......... .......... .......... .......... .......... .......... .......... ..........
D10                       .......... .......... .......... .......... .......... .......... .......A.. ..........
FCC2                      .......... .......... .......... .......... .......... .......... .......... ..........
HB3                       .......... .......... .......... .......... .......... .......... .......... ..........
7G8                       .......... .......... .......... .......... .......... .......... .......... ..........
P.reichenowi              .......... .....C.... ....A..... .......... ......G... .......... .......... ....C.T...

                                 570        580        590        600        610        620        630        640
                          ....|....| ....|....| ....|....| ....|....| ....|....| ....|....| ....|....| ....|....|
PFE0080c                  TATAGTAGGA TCAAGAGTTC ATACACCTTA TAAAGATTAC TTAGGAGATT TTAATAAATA TACTGAGATA AGTGTACTTA
3D7                       .......... .......... .......... .......... .......... .......... .......... ..........
RO33                      .......... .......... .......... .......... ..G....... .......... .......... ..........
Palo Alto                 .......... .......... .......... .......... C.T....... .......... .......... ..........
FCR3                      .......... .......... .......... .......... ..T....... .......... .......... ..........
Wellcome                  .......... .......... .......... .......... ..T....... .......... .......... ..........
D6                        .......... .......... .......... .......... .......... .......... .......... ..........
T996                      .......... .......... .......... .......... .......... .......... .......... ..........
T9102                     .......... .......... .......... .......... ..T....... .......... .......... ..........
K1                        .......... .......... .......... .......... ..T....... .......... .......... ..........
Dd2                       .......... .......... .......... .......... ..T....... .......... .......... ..........
D10                       .......... .......... .......... .......... ..T....... .......... .......... ..........
FCC2                      .......... .......... .......... .......... C.T....... .......... .......... ..........
HB3                       .......... .......... .......... .......... C.T....... .......... .......... ..........
7G8                       .......... .......... .......... .......... .......... .......... .......... ..........
P.reichenowi              .......... .......... .A........ ...T...... ..T....... .......... ......T... .........G
```

```
                        650        660        670        680        690        700        710        720
                ....|....| ....|....| ....|....| ....|....| ....|....| ....|....| ....|....| ....|....|
PFE0080c        ATTATGTTCG TGATTACAAT TTTTTAATTT ATGCTGGTTC AAGGGAAAAT TACTACAATT CAGATATAGC TGGACCAGCA
3D7             .......... .......... .......... .......... .......... .......... .......... ..........
RO33            .......... .......... .......... .......... .......... .......... .......... ..........
Palo Alto       .......... .......... .......... .......... .......... .......... .......... ..........
FCR3            .......... .......... .......... .......... .......... .......... .......... ..........
Wellcome        .......... .......... .......... .......... .......... .......... .......... ..........
D6              .......... .......... .......... .......... .......... .......... .......... ..........
T996            .......... .......... .......... .......... .......... .......... .......... ..........
T9102           .......... .......... .......... .......... .......... .......... .......... ..........
K1              .......... .......... .......... .......... .......... .......... .......... ..........
Dd2             .......... .......... .......... .......... .......... .......... .......... ..........
D10             .......... .......... .......... .......... .......... .......... .......... ..........
FCC2            .......... .......... .......... .......... .......... .......... .......... ..........
HB3             .......... .......... .......... .......... .......... .......... .......... ..........
7G8             .......... .......... .......... .......... .......... .......... .......... ..........
P.reichenowi    ...T...... .......... .......... .......... ..A....... .......... .......... A.........

                        730        740        750        760        770        780        790        800
                ....|....| ....|....| ....|....| ....|....| ....|....| ....|....| ....|....| ....|....|
PFE0080c        AGAAGTGTTA ATAATGTAAT TAGTAAGAAT AAAACATTAG GATTGAGAAA ACGTAGTAGT TCTCTCGCTT TAGTAGGAAC
3D7             .......... .......... .......... .......... .......... .......... .......... ..........
RO33            .......... .......... .......... .......... .......... .......... .......... ..........
Palo Alto       .......... .......... .......... .......... .......... .......... .......... ..........
FCR3            .......... .......... .......... .......... .......... .......... .......... ..........
Wellcome        .......... .......... .......... .......... .......... .......... .......... ..........
D6              .......... .......... .......... .......... .......... .......... .......... ..........
T996            .......... .......... .......... .......... .......... .......... .......... ..........
T9102           .......... .......... .......... .......... .......... .......... .......... ..........
K1              .......... .......... .......... .......... .......... .......... .......... ..........
Dd2             .......... .......... .......... .......... .......... .......... .......... ..........
D10             .......... .......... .......... .......... .......... .......... .......... ..........
FCC2            .......... .......... .......... .......... .......... .......... .......... ..........
HB3             .......... .......... .......... .......... .......... .......... .......... ..........
7G8             .......... .......... .......... .......... .......... .......... .......... ..........
P.reichenowi    .......... .......... .......... .......... .......... .......... .....T.... ..........

                        810        820        830        840        850        860        870        880
                ....|....| ....|....| ....|....| ....|....| ....|....| ....|....| ....|....| ....|....|
PFE0080c        AAATAACAAT GACCCTATAT TTGCTTATTG TGAAAAAGAT AATAAATCAG AATATTACGG TACACCAGAT GATTTAATTA
3D7             .......... .......... .......... .......... .......... .......... .......... ..........
RO33            .......... .......... .......... .......... .......... .......... .......... ..........
Palo Alto       .......... .......... .......... .......... .......... .......... .......... ..........
FCR3            .......... .......... .......... .......... .......... .......... .......... ..........
Wellcome        .......... .......... .......... .......... .......... .......... .......... ..........
D6              .......... .......... .......... .......... .......... .......... .......... ..........
T996            .......... .......... .......... .......... .......... .......... .......... ..........
T9102           .......... .......... .......... .......... .......... .......... .......... ..........
K1              .......... .......... .......... .......... .......... .......... .......... ..........
Dd2             .......... .......... .......... .......... .......... .......... .......... ..........
D10             .......... .......... .......... .......... .......... .......... .......... ..........
FCC2            .......... .......... .......... .......... .......... .......... .......... ..........
HB3             .......... .......... .......... .......... .......... .......... .......... ..........
7G8             .......... .......... .......... .......... .......... .......... .......... ..........
P.reichenowi    .......... .......... .......... .......... .......... .......... .......... ..........

                        890        900        910        920        930        940        950        960
                ....|....| ....|....| ....|....| ....|....| ....|....| ....|....| ....|....| ....|....|
PFE0080c        CATCTTTCTT TTCAATTATA AAAACTAAAA TGTTAAATTC TCATAAAACG TTTTTAAGAC AATTTGATTA TGCTTTATTT
3D7             .......... .......... .......... .......... .......... .......... .......... ..........
RO33            .......... .......... .......... .......... .......... .......... .......... ..........
Palo Alto       .......... .......... .......... .......... .......... .......... .......... ..........
FCR3            .......... .......... .......... .......... .......... .......... .......... ..........
Wellcome        .......... .......... .......... .......... .......... .......... .......... ..........
D6              .......... .......... .......... .......... .......... .......... .......... ..........
T996            .......... .......... .......... .......... .......... .......... .......... ..........
T9102           .......... .......... .......... .......... .......... .......... .......... ..........
K1              .......... .......... .......... .......... .......... .......... .......... ..........
Dd2             .......... .......... .......... .......... .......... .......... .......... ..........
D10             .......... .......... .......... .......... .......... .......... .......... ..........
FCC2            .......... .......... .......... .......... .......... .......... .......... ..........
HB3             .......... .......... .......... .......... .......... .......... .......... ..........
7G8             .......... .......... .......... .......... .......... .......... .......... ..........
P.reichenowi    .......... .......... .......... .......... ........A. .......... .......... ..........
```

```
                         970       980       990      1000      1010      1020      1030      1040
                  ....|....| ....|....| ....|....| ....|....| ....|....| ....|....| ....|....| ....|....|
PFE0080c          CACAAAACAT ATTCAATACC TAACTTAAAA GGTTTCAGAT TTTTGAAACA CCTTTTCCAA AAAAAGAATT TAGTGAATTT
3D7               .......... .......... .......... .......... .......... .......... .......... ..........
RO33              .......... .......... .......... .......... .......... .......... .......... ..........
Palo Alto         .......... .......... .......... .......... .......... .......... .......... ..........
FCR3              .......... .......... .......... .......... .......... .......... .......... ..........
Wellcome          .......... .......... .......... .......... .......... .......... .......... ..........
D6                .......... .......... .......... .......... .......... .......... .......... ..........
T996              .......... .......... .......... .......... .......... .......... .......... ..........
T9102             .......... .......... .......... .......... .......... .......... .......... ..........
K1                .......... .......... .......... .......... .......... .......... .......... ..........
Dd2               .......... .......... .......... .......... .......... .......... .......... ..........
D10               .......... .......... .......... .......... .......... .......... .......... ..........
FCC2              .......... .......... .......... .......... .......... .......... .......... ..........
HB3               .......... .......... .......... .......... .......... .......... .......... ..........
7G8               .......... .......... .......... .......... .......... .......... .......... ..........
P.reichenowi      ..T....... .......... .......... .......... .......... .......... .G........ ..........

                        1050      1060      1070      1080      1090      1100      1110      1120
                  ....|....| ....|....| ....|....| ....|....| ....|....| ....|....| ....|....| ....|....|
PFE0080c          TGTAGGTATG TATGAAAATC ACGTATCAAC AGAAATAAAT TTCTTAGCTG AAGATTTCGT TGAATTATTT GATGTAACTA
3D7               .......... .......... .......... .......... .......... .......... .......... ..........
RO33              .......... .......... .......... .......... .......... .......... .......... ..........
Palo Alto         .......... .......... .......... .......... .......... .......... .......... ..........
FCR3              .......... .......... .......... .......... .......... .......... .......... ..........
Wellcome          .......... .......... .......... .......... .......... .......... .......... ..........
D6                .......... .......... .......... .......... .......... .......... .......... ..........
T996              .......... .......... .......... .......... .......... .......... .......... ..........
T9102             .......... .......... .......... .......... .......... .......... .......... ..........
K1                .......... .......... .......... .......... .......... .......... .......... ..........
Dd2               .......... .......... .......... .......... .......... .......... .......... ..........
D10               .......... .......... .......... .......... .......... .......... .......... ..........
FCC2              .......... .......... .......... .......... .......... .......... .......... ..........
HB3               .......... .......... .......... .......... .......... .......... .......... ..........
7G8               .......... .......... .......... .......... .......... .......... .......... ..........
P.reichenowi      .......... .......... .......... .......... .......... .......... .......... ..........

                        1130      1140      1150      1160      1170      1180      1190
                  ....|....| ....|....| ....|....| ....|....| ....|....| ....|....| ....|....| ....|..
PFE0080c          TGGATTGTTA TTCTCGCCAA TATTCAAACC GTGCTGCAGA AAACTTCAAA GCTATTAGAG AATTAAATGT TCTTTAA
3D7               .......... .......... .......... .......... .......
RO33              .......... .......... .......... .......... .......
Palo Alto         .......... .......... .......... .......... .......
FCR3              .......... .......... .......... .......... .......
Wellcome          .......... .......... .......... .......... .......
D6                .......... .......... .......... .......... .......
T996              .......... .......... .......... .......... .......
T9102             .......... .......... .......... .......... .......
K1                .......... .......... .......... .......... .......
Dd2               .......... .......... .......... .......... .......
D10               .......... .......... .......... .......... .......
FCC2              .......... .......... .......... .......... .......
HB3               .......... .......... .......... .......... .......
7G8               .......... .......... .......... .......... .......
P.reichenowi      .......... .......... .......... .......... .......
```

PFE0075c – RAP3

```
                               10         20         30         40         50         60         70         80
                       ....|....| ....|....| ....|....| ....|....| ....|....| ....|....| ....|....| ....|....|
PFE0075c               ATGATTAGAA AATTTTTGAT TTCGTTATTT TTAATATTTT TATGTTTGAA CAATGTTGTA ATTGGAAATA AGTGTAAGAA
3D7                                                                                                 ........
RO33                                                                                                ........
Palo Alto                                                                                           ........
FCR3                                                                                                ........
Wellcome                                                                                            ........
D6                                                                                                  ........
T996                                                                                                ........
T9102                                                                                               ........
K1                                                                                                  ........
Dd2                                                                                                 ........
D10                                                                                                 ........
FCC2                                                                                                ........
HB3                                                                                                 ........
7G8                                                                                                 ........
P.reichenowi                                                                                        ..C.....

                               90        100        110        120        130        140        150        160
                       ....|....| ....|....| ....|....| ....|....| ....|....| ....|....| ....|....| ....|....|
PFE0075c               AGCATTGATA GATATTGATA CAAAGGATTT GTCTTTGTCT AGTATATTAC GTGCACATAA ACCCGATAAT ACAACATTAG
3D7                    .......... .......... .......... .......... .......... .......... .......... ..........
RO33                   .......... .......... .......... .......... .......... .......... .......... ..........
Palo Alto              .......... .......... .......... .......... .......... .......... .......... ..........
FCR3                   .......... .......... .......... .......... .......... .......... .......... ..........
Wellcome               .......... .......... .......... .......... .......... .......... .......... ..........
D6                     .......... .......... .......... .......... .......... .......... .......... ..........
T996                   .......... .......... .......... .......... .......... .......... .......... ..........
T9102                  .......... .......... .......... .......... .......... .......... .......... ..........
K1                     .......... .......... .......... .......... .......... .......... .......... ..........
Dd2                    .......... .......... .......... .......... .......... .......... .......... ..........
D10                    .......... .......... .......... .......... .......... .......... .......... ..........
FCC2                   .......... .......... .......... .......... .......... .......... .......... ..........
HB3                    .......... .......... .......... .......... .......... .......... .......... ..........
7G8                    .......... .......... .......... .......... .......... .......... .......... ..........
P.reichenowi           .......... .......... .......... .......... .......... .......... ......A... GG........

                              170        180        190        200        210        220        230        240
                       ....|....| ....|....| ....|....| ....|....| ....|....| ....|....| ....|....| ....|....|
PFE0075c               GTTCATGGGT TTATTTTTTC TTTAATCATT TTTCAAATGT GGATGAAGCA ATAGAATATT TAAAGGGATT AAATATAAAT
3D7                    .......... .......... .......... .......... .......... .......... .......... ..........
RO33                   .......... .......... .......... .......... .......... .......... .......... ..........
Palo Alto              .......... .......... .......... .......... .......... .......... .......... ..........
FCR3                   .......... .......... .......... .......... .......... .......... .......... ..........
Wellcome               .......... .......... .......... .......... .......... .......... .......... ..........
D6                     .......... .......... .......... .......... .......... .......... .......... ..........
T996                   .......... .......... .......... .......... .......... .......... .......... ..........
T9102                  .......... .......... .......... .......... .......... .......... .......... ..........
K1                     .......... .......... .......... .......... .......... .......... .......... ..........
Dd2                    .......... .......... .......... .......... .......... .......... .......... ..........
D10                    .......... .......... .......... .......... .......... .......... .......... ..........
FCC2                   .......... .......... .......... .......... .......... .......... .......... ..........
HB3                    .......... .......... .......... .......... .......... .......... .......... ..........
7G8                    .......... .......... .......... .......... .......... .......... .......... ..........
P.reichenowi           .......... .......... .......... ....T..... A......... .......... .......... ..........

                              250        260        270        280        290        300        310        320
                       ....|....| ....|....| ....|....| ....|....| ....|....| ....|....| ....|....| ....|....|
PFE0075c               GTATTAGATA TTGAAGATCA TGCTTGTTTT GCAAGAGCTT TTAGTGTATA TTTGCTTCAT TTTTATGCAA AAGATTTAAA
3D7                    .......... .......... .......... .......... .......... .......... .......... ..........
RO33                   .......... .......... .......... .......... .......... .......... .......... ..........
Palo Alto              .......... .......... .......... .......... .......... .......... .......... ..........
FCR3                   .......... .......... .......... .......... .......... .......... .......... ..........
Wellcome               .......... .......... .......... .......... .......... .......... .......... ..........
D6                     .......... .......... .......... .......... .......... .......... .......... ..........
T996                   .......... .......... .......... .......... .......... .......... .......... ..........
T9102                  .......... .......... .......... .......... .......... .......... .......... ..........
K1                     .......... .......... .......... .......... .......... .......... .......... ..........
Dd2                    .......... .......... .......... .......... .......... .......... .......... ..........
D10                    .......... .......... .......... .......... .......... .......... .......... ..........
FCC2                   .......... .......... .......... .......... .......... .......... .......... ..........
HB3                    .......... .......... .......... .......... .......... .......... .......... ..........
7G8                    .......... .......... .......... .......... .......... .......... .......... ..........
P.reichenowi           .......... .........G .......... .......... .......... .......... .......... G.........
```

```
                                330        340        350        360        370        380        390        400
                        ....|....| ....|....| ....|....| ....|....| ....|....| ....|....| ....|....| ....|....|
PFE0075c                AATGATGATT AGAAATGAAG AACATGAATC ATTTTTCAAA AATAAATTAA GTGAAATTAA TAATATAATA TCTGGTGATT
3D7                     .......... .......... .......... .......... .......... .......... .......... ..........
RO33                    .......... .......... .......... .......... .......... .......... .......... ..........
Palo Alto               .......... .......... .......... .......... .......... .......... .......... ..........
FCR3                    .......... .......... .......... .......... .......... .......... .......... ..........
Wellcome                .......... .......... .......... .......... .......... .......... .......... ..........
D6                      .......... .......... .......... .......... .......... .......... .......... ..........
T996                    .......... .......... .......... .......... .......... .......... .......... ..........
T9102                   .......... .......... .......... .......... .......... .......... .......... ..........
K1                      .......... .......... .......... .......... .......... .......... .......... ..........
Dd2                     .......... .......... .......... .......... .......... .......... .......... ..........
D10                     .......... .......... .......... .......... .......... .......... .......... ..........
FCC2                    .......... .......... .......... .......... .......... .......... .......... ..........
HB3                     .......... .......... .......... .......... .......... .......... .......... ..........
7G8                     .......... .......... .......... .......... .......... .......... .......... ..........
P.reichenowi            .......... .......... .......... .......... .C.......G .......... .......... ..........

                                410        420        430        440        450        460        470        480
                        ....|....| ....|....| ....|....| ....|....| ....|....| ....|....| ....|....| ....|....|
PFE0075c                TCCTTTCAAC ATTAAAACAT GAATATTTTT TTGATAAGTT ACCATCTATT ATAGTTAAAG AAAAAGATGC ATCCCATATA
3D7                     .......... .......... .......... .......... .......... .......... .......... ..........
RO33                    .......... .......... .......... .......... .......... .......... .......... ..........
Palo Alto               .......... .......... .......... .......... .......... .......... .......... ..........
FCR3                    .......... .......... .......... .......... .......... .......... .......... ..........
Wellcome                .......... .......... .......... .......... .......... .......... .......... ..........
D6                      .......... .......... .......... .......... .......... .......... .......... ..........
T996                    .......... .......... .......... .......... .......... .......... .......... ..........
T9102                   .......... .......... .......... .......... .......... .......... .......... ..........
K1                      .......... .......... .......... .......... .......... .......... .......... ..........
Dd2                     .......... .......... .......... .......... .......... .......... .......... ..........
D10                     .......... .......... .......... .......... .......... .......... .......... ..........
FCC2                    .......... .......... .......... .......... .......... .......... .......... ..........
HB3                     .......... .......... .......... .......... .......... .......... .......... ..........
7G8                     .......... .......... .......... .......... .......... .......... .......... ..........
P.reichenowi            .......... ...G...... .......... .......... ...T...... .......... .......... ...T......

                                490        500        510        520        530        540        550        560
                        ....|....| ....|....| ....|....| ....|....| ....|....| ....|....| ....|....| ....|....|
PFE0075c                GTAAAAAGGA CTGATTTTTT ACAAGATATA TTAGAAAAAG CAGATTTAAA TAACCATGCA ATTTATAAAA ACGATCCTAC
3D7                     .......... .......... .......... .......... .......... .......... .......... ..........
RO33                    .......... .......... .......... .......... .......... .......... .......... ..........
Palo Alto               .......... .......... .......... .......... .......... .......... .......... ..........
FCR3                    .......... .......... .......... .......... .......... .......... .......... ..........
Wellcome                .......... .......... .......... .......... .......... .......... .......... ..........
D6                      .......... .......... .......... .......... .......... .......... .......... ..........
T996                    .......... .......... .......... .......... .......... .......... .......... ..........
T9102                   .......... .......... .......... .......... .......... .......... .......... ..........
K1                      .......... .......... .......... .......... .......... .......... .......... ..........
Dd2                     .......... .......... .......... .......... .......... .......... .......... ..........
D10                     .......... .......... .......... .......... .......... .......... .......... ..........
FCC2                    .......... .......... .......... .......... .......... .......... .......... ..........
HB3                     .......... .......... .......... .......... .......... .......... .......... ..........
7G8                     .......... .......... .......... .......... .......... .......... .......... ..........
P.reichenowi            .......... .......... G......... .......... .C........ ...A...... .......... ..........

                                570        580        590        600        610        620        630        640
                        ....|....| ....|....| ....|....| ....|....| ....|....| ....|....| ....|....| ....|....|
PFE0075c                AAAAGTATTT ATTTTTAATG AAATAAATTT TTTTAGAACA TTCCAATTAG AAGGTAAACC ACATATACCT GATGATCAAC
3D7                     .......... .......... .......... .......... .......... .......... .......... ..........
RO33                    .......... .......... .......... .......... .......... .......... .......... ..........
Palo Alto               .......... .......... .......... .......... .......... .......... .......... ..........
FCR3                    .......... .......... .......... .......... .......... .......... .......... ..........
Wellcome                .......... .......... .......... .......... .......... .......... .......... ..........
D6                      .......... .......... .......... .......... .......... .......... .......... ..........
T996                    .......... .......... .......... .......... .......... .......... .......... ..........
T9102                   .......... .......... .......... .......... .......... .......... .......... ..........
K1                      .......... .......... .......... .......... .......... .......... .......... ..........
Dd2                     .......... .......... .......... .......... .......... .......... .......... ..........
D10                     .......... .......... .......... .......... .......... .......... .......... ..........
FCC2                    .......... .......... .......... .......... .......... .......... .......... ..........
HB3                     .......... .......... .......... .......... .......... .......... .......... ..........
7G8                     .......... .......... .......... .......... .......... .......... .......... ..........
P.reichenowi            .......... .......... .......... ......GT.. ..T....... .......... .......... A.........
```

```
                          650        660        670        680        690        700        710        720
                 ....|....| ....|....| ....|....| ....|....| ....|....| ....|....| ....|....| ....|....|
PFE0075c         TTTCTTTTAT GCGTGATTAT GCTTTATTAA TTTATCTTGG TACTAAGGAA AATTATTATA ATTCTGATAT AACAGAATAT
3D7              .......... .......... .......... .......... .......... .......... .......... ..........
RO33             .......... .......... .......... .......... .......... .......... .......... ..........
Palo Alto        .......... .......... .......... .......... .......... .......... .......... ..........
FCR3             .......... .......... .......... .......... .......... .......... .......... ..........
Wellcome         .......... .......... .......... .......... .......... .......... .......... ..........
D6               .......... .......... .......... .......... .......... .......... .......... ..........
T996             .......... .......... .......... .......... .......... .......... .......... ..........
T9102            .......... .......... .......... .......... .......... .......... .......... ..........
K1               .......... .......... .......... .......... .......... .......... .......... ..........
Dd2              .......... .......... .......... .......... .......... .......... .......... ..........
D10              .......... .......... .......... .......... .......... .......... .......... ..........
FCC2             .......... .......... .......... .......... .......... .......... .......... ..........
HB3              .......... .......... .......... .......... .......... .......... .......... ..........
7G8              .......... .......... .......... .......... .......... .......... .......... ..........
P.reichenowi     .......... .......... ..A....... .......... .......... .......... .......... ..........

                          730        740        750        760        770        780        790        800
                 ....|....| ....|....| ....|....| ....|....| ....|....| ....|....| ....|....| ....|....|
PFE0075c         GCACAAGGAA ATTATAATAT TTCGAAAAAT AGAACAAGAT TAGGATTAAA AAAACGTAGT AAAACATTTT CTTTAGATGA
3D7              .......... .......... .......... .......... .......... .......... .......... ..........
RO33             .......... .......... .......... .......... .......... .......... .......... ..........
Palo Alto        .......... .......... .......... .......... .......... .......... .......... ..........
FCR3             .......... .......... .......... .......... .......... .......... .......... ..........
Wellcome         .......... .......... .......... .......... .......... .......... .......... ..........
D6               .......... .......... .......... .......... .......... .......... .......... ..........
T996             .......... .......... .......... .......... .......... .......... .......... ..........
T9102            .......... .......... .......... .......... .......... .......... .......... ..........
K1               .......... .......... .......... .......... .......... .......... .......... ..........
Dd2              .......... .......... .......... .......... .......... .......... .......... ..........
D10              .......... .......... .......... .......... .......... .......... .......... ..........
FCC2             .......... .......... .......... .......... .......... .......... .......... ..........
HB3              .......... .......... .......... .......... .......... .......... .......... ..........
7G8              .......... .......... .......... .......... .......... .......... .......... ..........
P.reichenowi     .......... .......... .......... .......... .......... .......... .......... ..........

                          810        820        830        840        850        860        870        880
                 ....|....| ....|....| ....|....| ....|....| ....|....| ....|....| ....|....| ....|....|
PFE0075c         TCCCCAAAAA AATTCCAATA TATTTGCTTT TTGTGAAAAA AATGGAAAAG AAGAATTTTT TGGTACACCT GATGATTTAA
3D7              .......... .......... .......... .......... .......... .......... .......... ..........
RO33             .......... .......... .......... .......... .......... .......... .......... ..........
Palo Alto        .......... .......... .......... .......... .......... .......... .......... ..........
FCR3             .......... .......... .......... .......... .......... .......... .......... ..........
Wellcome         .......... .......... .......... .......... .......... .......... .......... ..........
D6               .......... .......... .......... .......... .......... .......... .......... ..........
T996             .......... .......... .......... .......... .......... .......... .......... ..........
T9102            .......... .......... .......... .......... .......... .......... .......... ..........
K1               .......... .......... .......... .......... .......... .......... .......... ..........
Dd2              .......... .......... .......... .......... .......... .......... .......... ..........
D10              .......... .......... .......... .......... .......... .......... .......... ..........
FCC2             .......... .......... .......... .......... .......... .......... .......... ..........
HB3              .......... .......... .......... .......... .......... .......... .......... ..........
7G8              .......... .......... .......... .......... .......... .......... .......... ..........
P.reichenowi     .......... .....A.... .......C.. .......... .........C .......... .......... ..........

                          890        900        910        920        930        940        950        960
                 ....|....| ....|....| ....|....| ....|....| ....|....| ....|....| ....|....| ....|....|
PFE0075c         TTTCATCATT TTTTTCTGAT ATGAAAGCTA AAATGGTTAA AGGGCATAAG AGATTTTTGA TGGAATTTGA TTATGCTGTT
3D7              .......... .......... .......... .......... .......... .......... .......... ..........
RO33             .......... .......... .......... .......... .......... .......... .......... ..........
Palo Alto        .......... .......... .......... .......... .......... .......... .......... ..........
FCR3             .......... .......... .......... .......... .......... .......... .......... ..........
Wellcome         .......... .......... .......... .......... .......... .......... .......... ..........
D6               .......... .......... .......... .......... .......... .......... .......... ..........
T996             .......... .......... .......... .......... .......... .......... .......... ..........
T9102            .......... .......... .......... .......... .......... .......... .......... ..........
K1               .......... .......... .......... .......... .......... .......... .......... ..........
Dd2              .......... .......... .......... .......... .......... .......... .......... ..........
D10              .......... .......... .......... .......... .......... .......... .......... ..........
FCC2             .......... .......... .......... .......... .......... .......... .......... ..........
HB3              .......... .......... .......... .......... .......... .......... .......... ..........
7G8              .......... .......... .......... .......... .......... .......... .......... ..........
P.reichenowi     .......... .......... .......... .......... .........A .......... .......... ..........
```

```
                    970        980        990        1000       1010       1020       1030       1040
            ....|....| ....|....| ....|....| ....|....| ....|....| ....|....| ....|....| ....|....|
PFE0075c    AAAAATAGAA CTTATGCTTT ACCTAAAGTT AAGGGATTTC GATTTTTAAA ACAACTTTTT CAAAGAAAAA ATTTAAAAAA
3D7         .......... .......... .......... .......... .......... .......... .......... ..........
RO33        .......... .......... .......... .......... .......... .......... .......... ..........
Palo Alto   .......... .......... .......... .......... .......... .......... .......... ..........
FCR3        .......... .......... .......... .......... .......... .......... .......... ..........
Wellcome    .......... .......... .......... .......... .......... .......... .......... ..........
D6          .......... .......... .......... .......... .......... .......... .......... ..........
T996        .......... .......... .......... .......... .......... .......... .......... ..........
T9102       .......... .......... .......... .......... .......... .......... .......... ..........
K1          .......... .......... .......... .......... .......... .......... .......... ..........
Dd2         .......... .......... .......... .......... .......... .......... .......... ..........
D10         .......... .......... .......... .......... .......... .......... .......... ..........
FCC2        .......... .......... .......... .......... .......... .......... .......... ..........
HB3         .......... .......... .......... .......... .......... .......... .......... ..........
7G8         .......... .......... .......... .......... .......... .......... .......... ..........
P.reichenowi .......... .......... .......... .......... .......... ...C...... .......... ....T.....

                    1050       1060       1070       1080       1090       1100       1110       1120
            ....|....| ....|....| ....|....| ....|....| ....|....| ....|....| ....|....| ....|....|
PFE0075c    TTTCGTAGGA ATGTATATAA ACCTTCTGTC TACTGAAATT GATTTCTTAG CAGAAGATTT TGTAGAAATG TTCGATACTA
3D7         .......... .......... .......... .......... .......... .......... .......... ..........
RO33        .......... .......... .......... .......... .......... .......... .......... ..........
Palo Alto   .......... .......... .......... .......... .......... .......... .......... ..........
FCR3        .......... .......... .......... .......... .......... .......... .......... ..........
Wellcome    .......... .......... .......... .......... .......... .......... .......... ..........
D6          .......... .......... .......... .......... .......... .......... .......... ..........
T996        .......... .......... .......... .......... .......... .......... .......... ..........
T9102       .......... .......... .......... .......... .......... .......... .......... ..........
K1          .......... .......... .......... .......... .......... .......... .......... ..........
Dd2         .......... .......... .......... .......... .......... .......... .......... ..........
D10         .......... .......... .......... .......... .......... .......... .......... ..........
FCC2        .......... .......... .......... .......... .......... .......... .......... ..........
HB3         .......... .......... .......... .......... .......... .......... .......... ..........
7G8         .......... .......... .......... .......... .......... .......... .......... ..........
P.reichenowi .......... .......... .T........ .......... .......... .......... .......... ..T.......

                    1130       1140       1150       1160       1170       1180       1190       1200
            ....|....| ....|....| ....|....| ....|....| ....|....| ....|....| ....|....| ....|....| ...
PFE0075c    CTATGAATTG TTATGGACGC CAACATGCCG CTCGTGCAGC AGATCATTAT ATGGATATGA AACTATCGAA CATATTCAAA TAA
3D7         .......... .......... .......... .......... .......... .......... .......... .
RO33        .......... .......... .......... .......... .......... .......... .......... .
Palo Alto   .......... .......... .......... .......... .......... .......... .......... .
FCR3        .......... .......... .......... .......... .......... .......... .......... .
Wellcome    .......... .......... .......... .......... .......... .......... .......... .
D6          .......... .......... .......... .......... .......... .......... .......... .
T996        .......... .......... .......... .......... .......... .......... .......... .
T9102       .......... .......... .......... .......... .......... .......... .......... .
K1          .......... .......... .......... .......... .......... .......... .......... .
Dd2         .......... .......... .......... .......... .......... .......... .......... .
D10         .......... .......... .......... .......... .......... .......... .......... .
FCC2        .......... .......... .......... .......... .......... .......... .......... .
HB3         .......... .......... .......... .......... .......... .......... .......... .
7G8         .......... .......... .......... .......... .......... .......... .......... .
P.reichenowi .......C.. .......... .......... .......... .......... .......... .......... .
```

PFD0955w – Pf34

```
                      10         20         30         40         50         60         70         80
              ....|....| ....|....| ....|....| ....|....| ....|....| ....|....| ....|....| ....|....|
PFD0955w      ATGTATGGTA CATTTTTGAA GGGGTCCATT TTTTACCTGT GTATATTTTT CCCATTTTTT TCGTGTGAGT GTAATAATAT
3D7                              .. .......... .......... .......... .......... .......... ..........
RO33                             .. .......... .......... .......... .......... .......... ..........
Palo Alto                        .. .......... .......... .......... .......... .......... ..........
FCR3                             .. .......... .......... .......... .......... .......... ..........
Wellcome                         .. .......... .......... .......... .......... .......... ..........
D6                               .. .......... .......... .......... .......... .......... ..........
T996                             .. .......... .......... .......... .......... .......... ..........
T9102                            .. .......... .......... .......... .......... .......... ..........
K1                               .. .......... .......... .......... .......... .......... ..........
Dd2                              .. .......... .......... .......... .......... .......... ..........
D10                              .. .......... .......... .......... .......... .......... ..........
FCC2                             .. .......... .......... .......... .......... .......... ..........
HB3                              .. .......... .......... .......... .......... .......... ..........
7G8                              .. .......... .......... .......... .......... .......... ..........
P.reichenowi  .......... .......... .......... .......... .......... .......... .......... ..........

                      90        100        110        120        130        140        150        160
              ....|....| ....|....| ....|....| ....|....| ....|....| ....|....| ....|....| ....|....|
PFD0955w      AAAATTAAAC GATAAAGAGA ATATAAATTT TGAGAGTTAT TTTAATAAAA GGACAAATGA AGAAAATGTA TTAAATAAAA
3D7           .......... .......... .......... .......... .......... .......... .......... ..........
RO33          .......... .......... .......... .......... .......... .......... .......... ..........
Palo Alto     .......... .......... .......... .......... .......... .......... .......... ..........
FCR3          .......... .......... .......... .......... .......... .......... .......... ..........
Wellcome      .......... .......... .......... .......... .......... .......... .......... ..........
D6            .......... .......... .......... .......... .......... .......... .......... .........G
T996          .......... .......... .......... .......... .......... .......... .......... ..........
T9102         .......... .......... ........C. .......... .......... .......... .......... ..........
K1            .......... .......... .......... .......... .......... .......... .......... ..........
Dd2           .......... .......... .......... .......... .......... .......... .......... ..........
D10           .......... .......... .......... .......... .......... .......... .......... ..........
FCC2          .......... .......... .......... .......... .......... .......... .......... ..........
HB3           .......... .......... .......... .......... .......... .......... .......... ..........
7G8           .......... .......... .......... .......... .......... .......... .......... ..........
P.reichenowi  .......... ........T. .......... .......... .......... A......... .......... ..........

                     170        180        190        200        210        220        230        240
              ....|....| ....|....| ....|....| ....|....| ....|....| ....|....| ....|....| ....|....|
PFD0955w      ACGTATCGAA AGAAATGGGG GATACGTTTG TTGCACATAA GGCTATAGAA TTAAACATTA ATCATCACCA CGTTAATAAT
3D7           .......... .......... .......... .......... .......... .......... .......... ..........
RO33          .......... .......... .......... .......... .......... .......... .......... ..........
Palo Alto     .......... .......... .......... .......... .......... .......... .......... ..........
FCR3          .......... .......... .......... .......... .......... .......... .......... ..........
Wellcome      .......... .......... .......... .......... .......... .......... .......... ..........
D6            .......... .......... .......... .......... .......... .......... .......... ..........
T996          .......... .......... .......... .......... .......... .......... .......... ..........
T9102         .......... .......... .......... .......... .......... .......... .......... ..........
K1            .......... .......... .......... .......... .......... .......... .......... ..........
Dd2           .......... .......... .......... .......... .......... .......... .......... ..........
D10           .......... .......... .......... .......... .......... .......... .......... ..........
FCC2          .......... .......... .......... .......... .......... .......... .......... ..........
HB3           .......... .......... .......... .......... .......... .......... .......... ..........
7G8           .......... .......... .......... .......... .......... .......... .......... ..........
P.reichenowi  GGA....... .......... .......... .......... A......... .......... .......... ..........

                     250        260        270        280        290        300        310        320
              ....|....| ....|....| ....|....| ....|....| ....|....| ....|....| ....|....| ....|....|
PFD0955w      GATAAAGAAT TTAATAATAA TAATAATAAT AAACATCAGC CTTATTATCA TAATGAGCAT GATAAGAAAT TTTCTGAAAG
3D7           .......... .......... .......... .......... .......... .......... .......... ..........
RO33          .......... .......... .......... .......... .......... .......... .......... ..........
Palo Alto     .......... .......... .......... .......... .......... .......... .......... ..........
FCR3          .......... .......... .......... .......... .......... .......... .......... ..........
Wellcome      .......... .......... .......... .......... .......... .......... .......... ..........
D6            .......... .......... .......... .......... .......... .......... .......... ..........
T996          .......... .......... .......... .......... .......... .......... .......... ..........
T9102         .......... .......... .......... .......... .......... .......... .......... ..........
K1            .......... .......... .......... .......... .......... .......... .......... ..........
Dd2           .......... .......... .......... .......... .......... .......... .......... ..........
D10           .......... .......... .......... .......... .......... .......... .......... ..........
FCC2          .......... .......... .......... .......... .......... .......... .......... ..........
HB3           .......... .......... .......... .......... .......... .......... .......... ..........
7G8           .......... .......... .......... .......... .......... .......... .......... ..........
P.reichenowi  .......... .......... ---------. ..G....... .......... .....G.... .......... ..........
```

```
                                  330        340        350        360        370        380        390        400
                          ....|....| ....|....| ....|....| ....|....| ....|....| ....|....| ....|....| ....|....|
PFD0955w                  TTTAAAAGCA CATATGGATC ACCTTAAGAT ATTAAATAAT GATTTAAAAC AACATATAGA TAAAAAAGAG AGAAATGAAA
3D7                       .......... .......... .......... .......... .......... .......... .......... ..........
RO33                      .......... .......... .......... .......... .......... .......... .......... ..........
Palo Alto                 .......... .......... .......... .......... .......... .......... .......... ..........
FCR3                      .......... .......... .......... .......... .......... .......... .......... ..........
Wellcome                  .......... .......... .......... .......... .......... .......... .......... ..........
D6                        .......... .......... .......... .......... .......... .......... .......... ..........
T996                      .......... .......... .......... .......... .......... .......... .......... ..........
T9102                     .......... .......... .......... .......... .......... .......... .......... ..........
K1                        .......... .......... .......... .......... .......... .......... .......... ..........
Dd2                       .......... .......... .......... .......... .......... .......... .......... ..........
D10                       .......... .......... .......... .......... .......... .......... .......... ..........
FCC2                      .......... .......... .......... .......... .......... .......... .......... ..........
HB3                       .......... .......... .......... .......... .......... .......... .......... ..........
7G8                       .......... .......... .......... .......... .......... .......... .......... ..........
P.reichenowi              .......... .......... .......... .......... .........G .......... .......... ..........

                                  410        420        430        440        450        460        470        480
                          ....|....| ....|....| ....|....| ....|....| ....|....| ....|....| ....|....| ....|....|
PFD0955w                  TATATGAAAA TAATGATTTA AAAAAATATA TAATAAAAGA GATACAAAAT AATAAATATT TAAATAAAGA AAAGAAAAGC
3D7                       .......... .......... .......... .......... .......... .......... .......... ..........
RO33                      .......... .......... .......... .......... .......... .......... .......... ..........
Palo Alto                 .......... .......... .......... .......... .......... .......... .......... ..........
FCR3                      .......... .......... .......... .......... .......... .......... .......... ..........
Wellcome                  .......... .......... .......... .......... .......... .......... .......... ..........
D6                        .......... .......... .......... .......... .......... .......... .......... ..........
T996                      .......... .......... .......... .......... .......... .......... .......... ..........
T9102                     .......... .......... .......... .......... .......... .......... .......... ..........
K1                        .......... .......... .......... .......... .......... .......... .......... ..........
Dd2                       .......... .......... .......... .......... .......... .......... .......... ..........
D10                       .......... .......... .......... .......... .......... .......... .......... ..........
FCC2                      .......... .......... .......... .......... .......... .......... .......... ..........
HB3                       .......... .......... .......... .......... .......... .......... .......... ..........
7G8                       .......... .......... .......... .......... .......... .......... .......... ..........
P.reichenowi              .......... .......... .......... .......... .......... .......... .......... G.........

                                  490        500        510        520        530        540        550        560
                          ....|....| ....|....| ....|....| ....|....| ....|....| ....|....| ....|....| ....|....|
PFD0955w                  AGTGAAGATA TTCAAATATT AGAAGAGCAT TCAAAAAAAT TACAAAAAGA AATTCATGAA TGGTTAGAAT CTGTTAATAA
3D7                       .......... .......... .......... .......... .......... .......... .......... ..........
RO33                      .......... .......... .......... .......... .......... .......... .......... ..........
Palo Alto                 .......... .......... .......... .......... .......... .......... .......... ..........
FCR3                      .......... .......... .......... .......... .......... .......... .......... ..........
Wellcome                  .......... .......... .......... .......... .......... .......... .......... ..........
D6                        .......... .......... .......... .......... .......... .......... .......... ..........
T996                      .......... .......... .......... .......... .......... .......... .......... ..........
T9102                     .......... .......... .......... .......... .......... .......... .......... ..........
K1                        .......... .......... .......... .......... .......... .......... .......... ..........
Dd2                       .......... .......... .......... .......... .......... .......... .......... ..........
D10                       .......... .......... .......... .......... .......... .......... .......... ..........
FCC2                      .......... .......... .......... .......... .......... .......... .......... ..........
HB3                       .......... .......... .......... .......... .......... .......... .......... ..........
7G8                       .......... .......... .......... .......... .......... .......... .......... ..........
P.reichenowi              .......... .......... .......... .......... .......... .......... ........G. ..........

                                  570        580        590        600        610        620        630        640
                          ....|....| ....|....| ....|....| ....|....| ....|....| ....|....| ....|....| ....|....|
PFD0955w                  TATTGAAGAG AAATCAAATA TTTTAAAAAA TATCAAAAGT CAATTATTAA ATAATATAGC TTCTTTAAAT CATACGCTCT
3D7                       .......... .......... .......... .......... .......... .......... .......... ..........
RO33                      .......... .......... .......... .......... .......... .......... .......... ..........
Palo Alto                 .......... .......... .......... .......... .......... .......... .......... ..........
FCR3                      .......... .......... .......... .......... .......... .......... .......... ..........
Wellcome                  .......... .......... .......... .......... .......... .......... .......... ..........
D6                        .......... .......... .......... .......... .......... .......... .......... ..........
T996                      .......... .......... .......... .......... .......... .......... .......... ..........
T9102                     .......... .......... .......... .......... .......... .......... .......... ..........
K1                        .......... .......... .......... .......... .......... .......... .......... ..........
Dd2                       .......... .......... .......... .......... .......... .......... .......... ..........
D10                       .......... .......... .......... .......... .......... .......... .......... ..........
FCC2                      .......... .......... .......... .......... .......... .......... .......... ..........
HB3                       .......... .......... .......... .......... .......... .......... .......... ..........
7G8                       .......... .......... .......... .......... .......... .......... .......... ..........
P.reichenowi              .......... .......... .......... .G.T.....C .......... .......... .......... .....A....
```

```
                      650        660        670        680        690        700        710        720
              ....|....| ....|....| ....|....| ....|....| ....|....| ....|....| ....|....| ....|....|
PFD0955w      CAGAAGAAAT AAAAAATATT AACGATATAA AAGAATTACA AAAACAACAA AATGATTTAT TTTCTGAAAA TTGGTTATAT
3D7           .......... .......... .......... .......... .......... .......... .......... ..........
RO33          .......... .......... .......... .......... .......... .......... .......... ..........
Palo Alto     .......... .......... .......... .......... .......... .......... .......... ..........
FCR3          .......... .......... .......... .......... .......... .......... .......... ..........
Wellcome      .......... .......... .......... .......... .......... .......... .......... ..........
D6            .......... .......... .......... .......... .......... .......... .......... ..........
T996          .......... .......... .......... .......... .......... .......... .......... ..........
T9102         .......... .......... .......... .......... .......... .......... .......... ..........
K1            .......... .......... .......... .......... .......... .......... .......... ..........
Dd2           .......... .......... .......... .......... .......... .......... .......... ..........
D10           .......... .......... .......... .......... .......... .......... .......... ..........
FCC2          .......... .......... .......... .......... .......... .......... .......... ..........
HB3           .......... .......... .......... .......... .......... .......... .......... ..........
7G8           .......... .......... .......... .......... .......... .......... .......... ..........
P.reichenowi  .......... .......... .......... .......... .......... .......... .......... ..T.......

                      730        740        750        760        770        780        790        800
              ....|....| ....|....| ....|....| ....|....| ....|....| ....|....| ....|....| ....|....|
PFD0955w      TTTCTTCCAT CCTCATCAGA TTATCTCTTA AACGAAAAAA AAAAAAATTT ATATGATAAT CAAGATAATA GTATGAAGGA
3D7           .......... .......... .......... .......... .......... .......... .......... ..........
RO33          .......... .......... .......... .......... .......... .......... .......... ..........
Palo Alto     .......... .......... .......... .......... .......... .......... .......... ..........
FCR3          .......... .......... .......... .......... .......... .......... .......... ..........
Wellcome      .......... .......... .......... .......... .......... .......... .......... ..........
D6            .......... .......... .......... .......... .......... .......... .......... ..........
T996          .......... .......... .......... .......... .......... .......... .......... ..........
T9102         .......... .......... .......... .......... .......... .......... .......... ..........
K1            .......... .......... .......... .......... .......... .......... .......... ..........
Dd2           .......... .......... .......... .......... .......... .......... .......... ..........
D10           .......... .......... .......... .......... .......... .......... .......... ..........
FCC2          .......... .......... .......... .......... .......... .......... .......... ..........
HB3           .......... .......... .......... .......... .......... .......... .......... ..........
7G8           .......... .......... ....T..... .......... .......... .......... .......... ..........
P.reichenowi  .......... .T........ .......... .......... .......... .........C .....G..C. .C........

                      810        820        830        840        850        860        870        880
              ....|....| ....|....| ....|....| ....|....| ....|....| ....|....| ....|....| ....|....|
PFD0955w      TGATATAAAT AATAATGACA AATATAATAT TTTTAATTAT TTACAAAACG TTCAAGATAA GGATAACCAA TATGAAGTTA
3D7           .......... .......... .......... .......... .......... .......... .......... ..........
RO33          .......... .......... .......... .......... .......... .......... .......... ..........
Palo Alto     .......... .......... .......... .......... .......... .......... .......... ..........
FCR3          .......... .......... .......... .......... .......... .......... .......... ..........
Wellcome      .......... .......... .......... .......... .......... .......... .......... ..........
D6            .......... .......... .......... .......... .......... .......... .......... ..........
T996          .......... .......... .......... .......... .......... .......... .......... ..........
T9102         .......... .......... .......... .......... .......... .......... .......... ..........
K1            .......... .......... .......... .......... .......... .......... .......... ..........
Dd2           .......... .......... .......... .......... .......... .......... .......... ..........
D10           .......... .......... .......... .......... .......... .......... .......... ..........
FCC2          .......... .......... .......... .......... .......... .......... .......... ..........
HB3           .......... .......... .......... .......... .......... .......... .......... ..........
7G8           .......... .......... .......... .......... .......... .......... .......... ..........
P.reichenowi  .......... .......... .......... ...C...... .......... .......... A.....G... ..........

                      890        900        910        920        930        940        950        960
              ....|....| ....|....| ....|....| ....|....| ....|....| ....|....| ....|....| ....|....|
PFD0955w      TGAAACAAGA CAATAATAAT ATACATAGTG GTTCCTCTAC TCATAATCAT CTATTATTAA CTTGTATAAT TTTTTTGTTA
3D7           .......... .......... .......... ..
RO33          .......... .......... .......... ..
Palo Alto     .......... .......... .......... ..
FCR3          .......... .......... .......... ..
Wellcome      .......... .......... .......... ..
D6            .......... .......... .......... ..
T996          .......... .......... .......... ..
T9102         .......... .......... .......... ..
K1            .......... .......... .......... ..
Dd2           .......... .......... .......... ..
D10           .......... .......... .......... ..
FCC2          .......... .......... .......... ..
HB3           .......... .......... .......... ..
7G8           .......... .......... .......... ..
P.reichenowi  .A........ .......... .......... ..CA...... .......... .......... .......... ..........
```

```
                                 970
                       ....|....| ....|...
PFD0955w               ATACTTTTAA TTTTATAA
3D7
RO33
Palo Alto
FCR3
Wellcome
D6
T996
T9102
K1
Dd2
D10
FCC2
HB3
7G8
P.reichenowi           .......... ........
```
