# Supplementary material for: Prospective Identification of Malaria Parasite Genes under Balancing Selection
Source: PLoS One. 2009 May 15;4(5):e5568. doi: 10.1371/journal.pone.0005568 (PMC2679211; doi:10.1371/journal.pone.0005568)
Supplement: Figure S4 — (0.05 MB PDF) [file pone.0005568.s004.pdf]

Supplementary Figure 4

PF13_0338 [Pf92] Repeat 1

```
[P reichenowi      GDIVYGHREFKGG]
Dd2                GDIVYGHREFKGG--------------------
T9/96              GDIVYGHREFKGGIVYGHREFKGG---------
T9/102             GDIVYGHREFKGG--------------------
R033               GDIVYGHREFKGGIVYGHREFKGG---------
3D7                GDIVYGHREFKGG--------------------
K1                 GDIVYGHREFKGGIVYGHREFKGG---------
Palo Alto          GDIVYGHREFKGGIVYGHREFKGG---------
FCR3               GDIVYGHREFKGGIVYGHREFKGGIVYGHREFK
D10                GDIVYGHREFKGGIVYGHREFKGG---------
HB3                GDIVYGHREFKGG--------------------
Wellcome           GDIVYGHREFKGGIVYGHREFKGGIVYGHREFK
Fcc2               GDIVYGHREFKGG--------------------
7G8                GDIVYGHREFKGG--------------------
D6                 GDIVYGHREFKGG--------------------
                   *************
```

PF14_0201 [Pf113] Repeat block

```
[P reichenowi -NNNNN--]
Dd2           NNNNNNNN
T9/96         NNNNNN--
T9/102        NNNNNNNN
R033          NNNNNN--
3D7           NNNNNNNN
K1            NNNNNNNN
Palo Alto     NNNNNNNN
FCR3          NNNNNNNN
D10           NNNNNNNN
HB3           NNNNNNNN
Wellcome      NNNNNNNN
Fcc2          NNNNNNNN
7G8           NNNNNNNN
D6            NNNNNN--
              ******
```

PFF0995c [MSP10] Repeat block 1

```
[P reichenowi KNDNK---DSIYNNNN--DNINK-----]
Dd2           KNDNKDNKDNIYNDNINN----------
T9/96         KNDNKDN----YNDNINNDNINN-----
T9/102        KNDNKDNKDNIYNDNINNDNINN-----
R033          KNDNKDNKDNIYNDNINNDNINN-----
3D7           KNDNKDNKDNIYNDNINNDNINNDNINN
K1            KNDNKDNKDNIYNDNINNDNINN-----
Palo Alto     KNDNKDN----YNDNINNDNINN-----
FCR3          KNDNKDNKDNIYNDNINN----------
D10           KNDNKDN----YNDNINNDNINN-----
HB3           KNDNKDNKDNIYNDNINNDNINN-----
Wellcome      KNDNKDNKDNIYNDNINN----------
Fcc2          KNDNKDNKDNIYNDNINN----------
7G8           KNDNKDNKDNIYNDNINN----------
D6            KNDNKDNKDNIYNDNINNDNINN-----
              *******    *******
```

PFF0995c [MSP10] Repeat Block 2 (continued)

```
[P reichenowi NENNENNENNENNENNENNENNEN------------------------------]
Dd2          NENIENNENVENNENIENNENNENIENIENNENNENNENIENNENNENNE---N
T9/96        NENIENNENVENNENIENNENNENIENIENNENNENNENIENNENNENNENNEN
T9/102       NENIENNENAENNENIENNENNENIENIENNENNENNENNENNENNENNE---N
R033         NENIENNENVENNENIENNENNENIENIENNENNENNENIENNENNENNE---N
3D7          NENIENNENVENNENIENNENNENIENIENNENNENNENIENNENNENNE---N
K1           NENIENNENVENNENIENNENNENIENIENNENNENNENIENNENNENNE---N
Palo Alto    NENIENNENVENNENIENNENNENIENIENNENNENNENIENNENNENNE---N
FCR3         NENIENNENVENNENIENNENNENIENIENNENNENNENIENNENNENNE---N
D10          NENIENNENVENNENIENNENNENIENIENNENNENNENIENNENNENNE---N
HB3          NENIENNENVENNENIENNENNENIENIENNENNENNENIENNENNENNE---N
Wellcome     NENIENNENVENNENIENNENNENIENIENNENNENNENIENNENNENNE---N
Fcc2         NENIENNENVENNENIENNENNENIENIENNENNENNENIENNENNENNE---N
7G8          NENIENNENVENNENIENNENNENIENIENNENNENNENIENNENNENNE---N
D6           NENIENNENVENNENIENNENNENIENIENNENNENNENIENNENNENNENNEN
             ********* ***************************** **********   *
```

PF10_0346 [MSP6]

```
[P reichenowi NDEEETETETENLETEEDNNEEIEENEEDDIDEEIVEENEEDDINDESVEEKEEKTEEKTENK]
Dd2           DEDEDEXXXTETENLETEDDNNEEIEENEEDDIDEESVEEKEEEEEKKEEEEKKEEKKEEKK
T9/96         DEDEDEXXXTETENLETEDDNNEEIEENEEDDIDEESVEEKEEEEEKKEEEEKKEEKKEEKK
T9/102        DEDEDEXXXTETENLETEDDNNEEIEENEEDDIDEESVEEKEEEEEKKEEEEKKEEKKEEKK
R033          DEDEDEXXXTETENLETEDDNNEEIEENEEDDIDEESVEEKEEEEEKKEEEEKKEEKKEEKK
3D7           DEDEDEXXXTETENLETEDDNNEEIEENEEDDIDEESVEEKEEEEEKKEEEEKKEEKKEEKK
K1            DEDEDEXXXTETENLETEDDNNEEIEENEEDDIDEESVEEKEEEEEKKEEEEKKEEKKEEKK
Palo Alto     DEDEDEDEETETENLETEDDNNEEIEENEEDDIDEESVEEKEEEEEKKEEEEKKEEKKEEKK
FCR3          DEDEDEXXXTETENLETEDDNNEEIEENEEDDIDEESVEEKEEEEEKKEEEEKKEEKKEEKK
D10           DEDEDEXXXTETENLETEDDNNEEIEENEEDDIDEESVEEKEEEEEKKEEEEKKEEKKEEKK
HB3           DEDEDEXXXTETENLETEDDNNEEIEENEEDDIDEESVEEKEEEEEKKEEEEKKEEKKEEKK
Wellcome      DEDEDEXXXTETENLETEDDNNEEIEENEEDDIDEESVEEKEEEEEKKEEEEKKEEKKEEKK
Fcc2          DEDEDEXXXTETENLETEDDNNEEIEENEEDDIDEESVEEKEEEEEKKEEEEKKEEKKEEKK
7G8           DEDEDEXXXTETENLETEDDNNEEIEENEEDDIDEESVEEKEEEEEKKEEEEKKEEKKEEKK
D6            DEDEDEXXXTETENLETEDDNNEEIEENEEDDIDEESVEEKEEEEEKKEEEEKKEEKKEEKK
              ******   *****************************************************
```

PF13_0193 [MRSP3]

```
[P reichenowi sequence stops short of repeat region]
Dd2           KQEDKQEDKREDK------------QE
T9/96         KQEDKQEDKREDK------------QE
T9/102        KQEDKQEDKREDK------------QE
R033          KQEDKQEDKREDK------------QE
3D7           KQEDKQEDKREDK------------QE
K1            KQEDKQEDKREDK------------QE
Palo Alto     KQEDKQEDKREDK------------QE
FCR3          KQEDKQEDKREDK------------QE
D10           KQEDKQEDKREDK------------QE
HB3           KQEDKQEDKREDK------------QE
Wellcome      KQEDKQEDKREDK------------QE
Fcc2          KQEDKQEDKREDK------------QE
7G8           KQEDKQEDKREDK------------QE
D6            KQEDKQEDKREDKQEDKQEDKREDKQE
              *************            **
```

MAL13P1.173 [MRSP4]

```
[P reichenowi ELEKEKE--
Dd2           ELEKEKEKE
T9/96         ELEKEKEKE
T9/102        ELEKEKEKE
R033          ELEKEKEKE
3D7           ELEKEKEKE
K1            ELEKEKEKE
Palo Alto     ELEKEKEKE
FCR3          ELEKEKE--
D10           ELEKEKEKE
HB3           ELEKEKEKE
Wellcome      ELEKEKE--
Fcc2          ELEKEKEKE
7G8           ELEKEKEKE
D6            ELEKEKEKE
              *******
```

PF13_0191 [MRSP5] Repeat block 1

```
[P reichenowi DEEETDEDDDEDSDEDDEEQEE[LNVEPKER]EDEQEETDDEQ
Dd2           DEEETDEDDDEDSDEDDEEQEE[LNVEPKER]EDEQEETDDEQ
T9/96         DEEETDEDDDEDSDEDDEEQEE[LNVEPKER]EDEQEETDDEQ
T9/102        DEEETDEDDDEDSDEDDEEQEE[LNVEPKER]EDEQEETDDEQ
R033          DEEETDEDDDEDSDEDDEEQEE[LNVEPKER]EDEQEETDDEQ
3D7           DEEETDEDDDEDSDEDDEEQEE[LNVEPKER]EDEQEETDDEQ
K1            DEEETDEDDDEDSDEDDEEQEE[LNVEPKER]EDEQEETDDEQ
Palo Alto     DEEETDEDDDEDSDEDDEEQEE[LNVEPKER]EDEQEETDDEQ
FCR3          DEEETDEDDDEDSDEDDEEQEE[LNVEPKER]EDEQEETDDEQ
D10           DEEETDEDDDEDSDEDDEEQEE[LNVEPKER]EDEQEETDDEQ
HB3           DEEETDEDDDEDSDEDDEEQEE[LNVEPKER]EDEQEETDDEQ
Wellcome      DEEETDEDDDEDSDEDDEEQEE[LNVEPKER]EDEQEETDDEQ
Fcc2          DEEETDEDDDEDSDEDDEEQEE[LNVEPKER]EDEQEETDDEQ
7G8           DEEETDEDDDEDSDEDDEEQEE[LNVEPKER]EDEQEETDDEQ
D6            DEEETDEDDDEDSDEDDEEQEE[LNVEPKER]EDEQEETDDEQ
              **********************[non-rep ]***********
```

PF13_0191 [MRSP5] Repeat block 2

```
[P reichenowi-------KETDDEQKETDDEQKETDDEQKETDDEQKETDDEQKETDDDQKETDDEQKETDDEQ-------------]
Dd2          -------KETEDEQKETEDEQKETEDEQ--------------KESDDEQ-------KETEDEQKETEDEQ-------
T9/96        -------KETEDEQKETEDEQKETEDEQKETEDEQ-------KESDDEQ-------KETEDEQ--------------
T9/102       -------KETEDEQKETEDEQKETEDEQKETEDEQ-------KESDDEQ-------KETEDEQKETEDEQ-------
R033         EETDDEQKETEDEQKETEDEQKETEDEQKETEDEQKETEDEQKESDDEQ-------KETEDEQ--------------
3D7          -------KETEDEQKETEDEQKETEDEQKETEDEQKETEDEQKESDDEQ-------KETEDEQ--------------
K1           -------KETEDEQKETEDEQKETEDEQKETEDEQ-------KESDDEQ-------KETEDEQKETEDEQ-------
Palo Alto    EETDDEQKETEDEQKETEDEQKETEDEQ--------------KESDDEQ-------KETEDEQKETEDEQ-------
FCR3         -------KETEDEQKETEDEQKETEDEQ--------------KESDDEQ-------KETEDEQKETEDEQKETEDEQ
D10          -------KETEDEQKETEDEQKETEDEQ--------------KESDDEQ-------KETEDEQKETEDEQ-------
HB3          -------KETEDEQKETEDEQ---------------------KESDDEQ-------KETEDEQ--------------
Wellcome     -------KETEDEQKETEDEQKETEDEQ--------------KESDDEQ-------KETEDEQKETEDEQKETEDEQ
Fcc2         -------KETEDEQKETEDEQKETEDEQKETEDEQKETEDEQKESDDEQ-------KETEDEQ--------------
7G8          -------KETEDEQKETEDEQKETEDEQKETEDEQ-------KESDDEQ-------KETEDEQKETEDEQ-------
D6           -------KETEDEQKETEDEQKETEDEQ--------------KESDDEQKESDDEQKETEDEQ--------------
                    **************                     *******       *****
```

PF13_0191 [MRSP5] Repeat block 3

```
[P reichenowiDNEEEDEE----ESEENEENNDNDTNEENEDNDEN------EEE]
Dd2          DNEEDDEEDEEDDEEENEENNDNETNEENEDNDEN------EEE
T9/96        DNEEDDEEDEEDDEEENEENNDNETNEENEDNDENEDNDENEEE
T9/102       DNEEDDEEDEEDDEEENEENNDNETNEENEDNDEN------EEE
R033         DNEEDDEE---DDEEENEENNDNETNEENEDNDEN------EEE
3D7          DNEEDDEEDEEDDEEENEENNDNETNEENEDNDENEDNDENEEE
K1           DNEEDDEEDEEDDEEENEENNDNETNEENEDNDEN------EEE
Palo Alto    DNEEDDEEDEEDDEEENEENNDNETNEENEDNDEN------EEE
FCR3         DNEEDDEE---DDEEENEENNDNETNEENEDNDEN------EEE
D10          DNEEDDEEDEEDDEEENEENNDNETNEENEDNDEN------EEE
HB3          DNEEDDEEDEEDDEEENEENNDNETNEENEDNDEN------EEE
Wellcome     DNEEDDEE---DDEEENEENNDNETNEENEDNDEN------EEE
Fcc2         DNEEDDEEDEEDDEEENEENNDNETNEENEDNDEN------EEE
7G8          DNEEDDEEDEEDDEEENEENNDNETNEENEDNDEN------EEE
D6           DNEEDDEEDEEDDEEENEENNDNETNEENEDNDENEDNDENEEE
             ********   ************************      ***
```

Pf13_0192 (MRSP family member) Repeat Block 1

```
[P.reichenowi      GEDDD------------------------------------------DEDDEDDVDDEDD------VDDED
Dd2                GEDDDDFDEDDDDFDEDDDDFDEDDDDFDEDDDDFDEDDE------------DDVDDEDD------LDVED
T9/96              GEDDDDFDEDDDDF------------------------DEDDEDDEDDEDDEDDVDDEDD------LDVED
T9/102             GEDDDDFDEDDDDFDEDDDDF----------------------DEDDDEDDEDDVDDEDD------LDVED
RO33               GEDDDDFDEDDDDF------------------------DEDDEDDEDDEDDEDDVDDEDD------VDVED
3D7                GEDDDDFDEDDDDF------------------------DEDDEDDEDDEDDEDDVDDEDD------LDVED
K1                 GEDDDDFDEDDDDFDEDDDDF----------------------DEDDDEDDEDDVDDEDD------LDVED
Palo Alto          GEDDDDFDEDDDDF---------------------------------DEDDEDDVDDEDDVDDEDDLDVED
FCR3               GEDDDDFDEDDDDFDEDDDDF--------------DEDDEDDEDDEDDE---DDVDDEDD------LDVED
D10                GEDDDDFDEDDDDFDEDDDDF--------------DEDDEDDEDDEDDE---DDVDDEDD------LDVED
HB3                GEDDDDFDEDDDDF---------------------DEDDEDDEDDEDDEDDEDDVDDEDD------LDVED
Wellcome           GEDDDDFDEDDDDFDEDDDDF-----------------DEDDEDDEDDEDDEDDVDDEDD------LDVED
FCC2               GEDDDDFDEDDDDF---------------------DEDDEDDEDDEDDE---DDVDDEDD------LDVED
7G8                GEDDDDFDEDDDDFDEDDDDF--------------DEDDEDDE---------DDVDDEDD------LDVED
D6                 GEDDDDFDEDDDDFDEDDDDF--------------------------DEDDEDDVDDEDDVDDEDDVDDED
                   **************                                          ********   * **
```

Pf13_0192 – Repeat Block 2

```
[P.reichenowi]     NINNE------------------------------------NNDKH------------
Dd2                NINND------------------------------NNDNNENNDNNENNDNN------
T9/96              NINNDNNDNNENNDNNENND---------------NNDNNENNDNNENNDNN------
T9/102             NINND------------------------------NNDNNENNDNNENNDNN------
RO33               NINNDNNENNDNNENNDNNENN---------DNNENNDNNENNDNNENNDNN------
3D7                NINND------------------------------NNDNNENNDNNENNDNN------
K1                 NINND------------------------------NNDNNENNDNNENNDNNENNDNN
Palo Alto          NINND------------------------------NNDNNENNDNN------------
FCR3               NINND------------------------------NNDNNENNDNNENNDNN------
D10                NINND------------------------------NNDNNENNDNNENNDNN------
HB3                NINNDNNDNNENNDNNENNDNNENNDNNENNDNNENNDNNENNDNNENNDNN------
Wellcome           NINND------------------------------NNDNNENNDNNENNDNN------
FCC2               NINND------------------------------NNDNNENNDNNENNDNN------
7G8                NINND------------------------------NNDNNENNDNNENNDNN------
D6                 NINNDNNE---------------------------NNDNNENNDNNENNDNNENNDNN
                   ****                               ***********
```

Pf13_0192 – Repeat Block 3

```
[P.reichenowi]      NNNNKKKNNQKNNQKNNQKNNQKYNQKNNQKNN
Dd2                 NNNNKKKN-----KKNSQKNNQKNNQKNNQKHN
T9/96               NNNKKKKN-----KKNSQKNNQKNNQKNNQKHN
T9/102              NNNNKKKN-----KKNSQKNNQKNNQKNNQKHN
RO33                NNNNKKKN-----KKNSQKNNQKNNQKNNQKHN
3D7                 NNNNKKKN-----KKNSQKNNQKNNQKNNQKHN
K1                  NNNNKKKN-----KKNSQKNNQKNNQKNNQKHN
Palo Alto           NNNNKKKN-----KKNSQKNNQKNNQKNNQKHN
FCR3                NNNNKKKN-----KKNSQKNNQKNNQKNNQKHN
D10                 NNNNKKKN-----KKNSQKNNQKNNQKNNQKHN
HB3                 NNNNKKKN-----KKNSQKNNQKNNQKNNQKHN
Wellcome            NNNNKKKN-----KKNSQKNNQKNNQKNNQKHN
FCC2                NNNNKKKN-----KKNSQKNNQKNNQKNNQKHN
7G8                 NNNNKKKN-----KKNSQKNNQKNNQKNNQKHN
D6                  NNNNKKKN-----KKNSQKNNQKNNQKNNQKHN
                    *** ****      ***************
```

PF10_0348 [MSP3/6-like]

```
[P reichenowiDTED------------]
Dd2          DTED---TDDTEDTED
T9/96        DTEDTEDTDDTEDTED
T9/102       ---DTEDTDDTEDTED
R033         ---DTEDTDDTEDTED
3D7          ---DTEDTDDTEDTED
K1           ---DTEDTDDTEDTED
Palo Alto    ---DTEDTDDTEDTED
FCR3         ---DTEDTDDTEDTED
D10          ---DTEDTDDTEDTED
HB3          ---DTEDTDDTEDTED
Wellcome     ---DTEDTDDTEDTED
Fcc2         ---DTEDTDDTEDTED
7G8          ---DTEDTDDTEDTED
D6           ---DTEDTDDTEDTED
                    *********
```

PF10_0352 [MSP3/6-like]

```
[P reichenowiTDVTDETDETEETEETED]
Dd2          TEDTDETEDTDETEETED
T9/96        TEDTDETEDTDETEETED
T9/102       TEDTDETEDTDETEETED
R033         TEDTDETEDTDETEETED
3D7          TEDTDETEDTDETEETED
K1           TEDTDETEDTDETEETED
Palo Alto    TEDTDETEDTDETEETED
FCR3         TEDTDETEDTDETEETED
D10          TEDTDETEDTDETEETED
HB3          TEDTDETEDTDETEETED
Wellcome     TEDTDETEDTDETEETED
Fcc2         TEDTDETEDTDETEETED
7G8          TEDTDETEDTDETEETED
D6           TEDTDETEDTDETEETED
             ******************
```

PFL1385c [MSP9] Repeat block 1

```
[P reichenowiSANSLENN----]
Dd2          SANNSANN----
T9/96        SANNSANN----
T9/102       SANNSANN----
R033         SANNSANN----
3D7          SANNSANN----
K1           SANNSANN----
Palo Alto    SANNSANN----
FCR3         SANNSANN----
D10          SANNSANN----
HB3          SANNSANN----
Wellcome     SANNSANN----
Fcc2         SANNSANN----
7G8          SANNSANN----
D6           SANNSANNSANN
             ********
```

PFL1385c [MSP9] Repeat block 2 (continued)

```
[P reichenowi DEEEVNDEEEVNDEEDVNDEEDVNDEEDVNDEEDLNDEED]
Dd2          DEEDTNDDEDTNDEEDTNDDEDTNDDEDTNDEEDTNDEED
T9/96        DEEDTNDDEDTNDEEDTNDDEDTNDDEDTNDEEDTNDEED
T9/102       DEEDTNDDEDTNDEEDTNDDEDTNDDEDTNDEEDTNDEED
R033         DEEDTNDDEDTNDEEDTNDDEDTNDDEDTNDEEDTNDEED
3D7          DEEDTNDDEDTNDEEDTNDDEDTNDDEDTNDEEDTNDEED
K1           DEEDTNDDEDTNDEEDTNDDEDTNDDEDTNDEEDTNDEED
Palo Alto    DEEDTNDDEDTNDEEDTNDDEDTNDDEDTNDEEDTNDEED
FCR3         DEEDTNDDEDTNDEEDTNDDEDTNDDEDTNDEEDTNDEED
D10          DEEDTNDDEDTNDEEDTNDDEDTNDDEDTNDEEDTNDEED
HB3          DEEDTNDDEDTNDEEDTNDDEDTNDDEDTNDEEDTNDEED
Wellcome     DEEDTNDDEDTNDEEDTNDDEDTNDDEDTNDEEDTNDEED
Fcc2         DEEDTNDDEDTNDEEDTNDDEDTNDDEDTNDEEDTNDEED
7G8          DEEDTNDDEDTNDEEDTNDDEDTNDDEDTNDEEDTNDEED
D6           DEEDTNDDEDTNDEEDTNDDEDTNDDEDTNDEEDTNDEED
             ****************************************
```

PFL1385c [MSP9] Repeat block 3

```
[P reichenowi KEEKE no further sequence obtained]
Dd2          KEEEEKEKEKE-KEKEEKE--KEEKEKEEKE---------------KE---KEEKEEEKKEKEEEQEEEEEE-
T9/96        KEEEEKEKEKE-KEKEEKE--KEEKEKEKEE-----KEKEEKEKE------KEEKEEEKKEKEEEQEEEEEEE
T9/102       KEEEEKEKEKE-KEKEEKE--KEEKEKEEKE--KEEKEKEEKEKE---KEEKEEKEEEKKEKEEEEEEEEG
R033         KEEEEKEKEKE-KEKEEKE--KEEKEKEEKE--KEE-----KEKE---------KEEEKKEKEEEQEEEEEE
3D7          KEEEEKEKEKEEKEKEEKEKEKEEKEKEEKE--KEEKEKEE-------------KEEEKKEKEEEQEEEEEEE
K1           KEEEEKEKEKE-KEKEEKEKEKEEKEKEEKEKEKEE-----------------KEEEKKEKEEEQEEEEEE
Palo Alto    KEEEEKEKEKE-KEKEEKE--KEEKEKEEKEKEKEEKE---KE--------KEEKEEEKKEKEEEQEEEEEE-
FCR3         KEEEEKEKEKE-KEKEEKE--KEEKE-----KEKEEKE---KEKE------KEEKEEEKKEKEEEQE
D10          KEEEEKEKEKE-KEKEEKE--KEEKE-----KEKEEKEKEE-----KEKEEKEEKEEEKKEKEEEQEEEEEE-
HB3          KEEEEKEKEKE-KEKEEKE--KEEKE-------KEEKEKEEKEKEEKEKEEKEEKEEEKKEKEEER
Wellcome     KEEEEKEKEKE-KEKEEKE--KEEKE-------KEKEEKE-KEKE------KEEKEEEKKEKEEEQEEEEEE
Fcc2         KEEEEKEKEKE-KEKEEKE--KEEKE-------KEEKE--------KEKEEKEEKEEEKKEKEEEQEEEEEER
7G8          KEEEEKEKEKE-KEKEEKE--KEEKE-------KEEKE--------KE------KEEE-EEEEEEKEEEEEEEEEEEE
D6           KEEEEKEKEKE-KEKEEKE--KEEKE-------KEEKEKEE-------KEE---KEEEKKEKEEEQEEEEEE
```

PFB0340c [SERA5] Repeat block 1

```
[P reichenowi   no equivalent sequence obtained]
Dd2          KAGNTGGGQAGNTVGD------------------------QAGSTGGS
T9/96        QAGNTGGGQAGNTVGD------------------------QAGSTGGS
T9/102       QTGNTGGGQAGNTVGGQAGNTVGD----------------QAGSTGGS
R033         QTRNTGGGQAGNTVGDQAGNTVGD----------------QAGSTGGS
3D7          ----TGGGQAGNTGGD------------------------QAGSTGGS
K1           -----GGGQAGNTGGD--------QAGSTGGN----------------
Palo Alto    -----GGGQAGNTGGGQAGNTVGD----------------QAGSTGGS
FCR3         ----TGGGQAGNTVGD------------------------QAGSTGGS
D10          -------GQAGNTGGD------------------------QAGSTGGS
HB3          ----TGGGQAGNTVGD------------------------QAGSTGGS
Wellcome     QAGNTGGGQAGNTVGD------------------------QAGSTGGS
Fcc2         ---YTGGGQAGNTVGGQAGNTVGD----------------QAGSTGGS
7G8          ----TGGGQAGNTVGD------------------------QAGSTGGS
D6           ------GGQAGNTVGD----------------QAGNTVGS--------
                    ****** *
```

PFB0340c [SERA5] Repeat block 2

```
[P reichenowi no equivalent sequence obtained]
Dd2          PQGSTGAS----------------
T9/96        PQGSTGAS----------------
T9/102       PQGSTGAS----------------
R033         PQGSTGAS----------------
3D7          PQGSTGASPQGSTGASPQGSTGAS
K1           PPGSTGASPQGSTGASPPGSTEAS
Palo Alto    PQGSTGAS----------------
FCR3         PQGSTGAS----------------
D10          PQGSTGAS----------------
HB3          PQGSTGAS----------------
Wellcome     PQGSTGAS----------------
Fcc2         PQGSTGAS----------------
7G8          PQGSTGAS----------------
D6           PQGSTGASPQGSTGAS--------
             * ******
```

PFB0340c [SERA5] Repeat block 3

```
[P reichenowi SSSSSSSSSEPSSDSSSSSSSSSSSSSSSSSSSSSSSGS]
Dd2          SSSSSSSSSSSSSSSSSSSSS-------------------------
T9/96        SSSSSSSSSSSSSSSSSSSSSSSSSSSSSSSSS-------------
T9/102       SSSSSSSSSSSSSSSSSSSSS-------------------------
R033         SSSSSSSISSSSSSSSSSSSSSSSSSSSSSSSSSSSS---------
3D7          SSSSSSSSSSSSSSSSSSSSSSSSSSS-------------------
K1           SSSSSSSSSSSSSSSSSSSSSSSSSSS-------------------
Palo Alto    SSSSSSSSSSSSSSSSSSSSS-------------------------
FCR3         SSSSSSSSSSNSSSSSSSSSSSSSSSSSSSSSSSSSSS--------
D10          SSSSSSSSSSSSSSSSSSSSSSSSSSSSSSSSSSSSSSSSSSSSSS
HB3          SSSSSSSSSSSSSSSSSSSSSSSSSSSSSSSSSSS-----------
Wellcome     SSSSSSSSSSNSSSSSSSSSSSSSSSSSSSSSSSSSSS--------
Fcc2         SSSSSSSSSSSSSSSSSSSSSSSSSSSSSSSSS-------------
7G8          SSSSSSSSSSSSSSSSSSSSSSSSSSSSSSSSS-------------
D6           SSSSSSSSSSSSSSSSSSSSSSSSSSSSSSS---------------
             ******* ** **********
```

MAL7P1.208 [RAMA] Repeat block 1

```
[P reichenowi NFLEINNLENEKKENDEKEDEYEYEDNDESFLETEEYEDNEDEKYNNDEDDYAESFIETDKYEDNEDEKYNKDEKYNKDEKYNKDEDDYAE
Dd2           SFLEINKLENDKKEKHEKEDEYE—-DNDESFLETEEYEDNEDEKYNKDEDDYAESFIETDEYEDNED------------------------
T9/96         SFLEINKLENDKKEKHEKEDEYE—-DNDESFLETEEYEDNEDEKYNKDEDDYAESFIETDEYEDNED------------------------
T9/102        SFLEINKLENDKKEKHEKEDEYE—-DNDESFLETEEYEDNEDEKYNKDEDDYAESFIETDEYEDNED------------------------
R033          SFLEINKLENDKKEKHEKEDEYE—-DNDESFLETEEYEDNEDEKYNKDEDDYAESFIETDEYEDNED------------------------
3D7           SFLEINKLENDKKEKHEKEDEYE—-DNDESFLETEEYEDNEDEKYNKDEDDYAESFIETDEYEDNED------------------------
K1            SFLEINKLENDKKEKHEKEDEYE—-DNDESFLETEEYEDNEDEKYNKDEDDYAESFIETDEYEDNED------------------------
Palo Alto     SFLEINKLENDKKEKHEKEDEYE—-DNDESFLETEEYEDNEDEKYNKDEDDYAESFIETDEYEDNED------------------------
FCR3          SFLEINKLENDKKEKHEKEDEYE—-DNDESFLETEEYEDNEDEKYNKDEDDYAESFIETDEYEDNED------------------------
D10           SFLEINKLENDKKEKHEKEDEYE—-DNDESFLETEEYEDNEDEKYNKDEDDYAESFIETDEYEDNED------------------------
HB3           SFLEINKLENDKKEKHEKEDEYE—-DNDESFLETEEYEDNEDEKYNKDEDDYAESFIETDEYEDNED------------------------
Wellcome      SFLEINKLENDKKEKHEKEDEYE—-DNDESFLETEEYEDNEDEKYNKDEDDYAESFIETDEYEDNED------------------------
Fcc2          SFLEINKLENDKKEKHEKEDEYE—-DNDESFLETEEYEDNEDEKYNKDEDDYAESFIETDEYEDNED------------------------
7G8           SFLEINKLENDKKEKHEKEDEYE—-DNDESFLETEEYEDNEDEKYNKDEDDYAESFIETDEYEDNED------------------------
D6            SFLEINKLENDKKEKHEKEDEYE—-DNDESFLETEEYEDNEDEKYNKDEDDYAESFIETDEYEDNED------------------------
              ***********************  ******************************************
```

MAL7P1.208 [RAMA] Repeat block 1 (continued)

```
[P reichenowi SFIETDEYEDNKDDKYNKDEKYNKDEDDYAESFIETDEYDDNE]
Dd2           -------------------DKYNKDEDDYSESFIETDEYDDNE
T9/96         -------------------DKYNKDEDDYSESFIETDEYDDNE
T9/102        -------------------DKYNKDEDDYSESFIETDEYDDNE
R033          -------------------DKYNKDEDDYSESFIETDEYDDNE
3D7           -------------------DKYNKDEDDYSESFIETDEYDDNE
K1            -------------------DKYNKDEDDYSESFIETDEYDDNE
Palo Alto     -------------------DKYNKDEDDYSESFIETDEYDDNE
FCR3          -------------------DKYNKDEDDYSESFIETDEYDDNE
D10           -------------------DKYNKDEDDYSESFIETDEYDDNE
HB3           -------------------DKYNKDEDDYSESFIETDEYDDNE
Wellcome      -------------------DKYNKDEDDYSESFIETDEYDDNE
Fcc2          -------------------DKYNKDEDDYSESFIETDEYDDNE
7G8           -------------------DKYNKDEDDYSESFIETDEYDDNE
D6            -------------------DKYNKDEDDYSESFIETDEYDDNE
                                 ************************
```

MAL7P1.208 [RAMA] Repeat block 2

```
[P reichenowiEEMKDEEM------------------------------KYDEMKDDQMNYDEMKDEEIKDDEMKYDEMKDEEMKYDEMKDEEMKY
Dd2          EEMKDEEMKDEEMEDVEMKDEEMKDEEMKYDEMKNEEMKYDEM------------------------------------------
T9/96        EEMKDEEMKDEEMKDVEMKDEEMKDEEMKYDEMKNEEMKYDEM------------------------------------------
T9/102       EEMKDEEMKDEEMEDVEMKDEEMKDEEMKYDEMKNEEMKYDEM------------------------------------------
R033         EEMKDEEMKDEEMKDVEMKDEEMKDEEMKYDEMKNEEMKYDEM------------------------------------------
3D7          EEMKDEEMKDEEMKDVEMKDEEMKDEEMKYDEMKNEEMKYDEM------------------------------------------
K1           EEMKDEEMKDEEMKDVEMKDEEMKDEEMKYDEMKNEEMKYDEM------------------------------------------
Palo Alto    EEMKDEEMKDEEMKDVEMKDEEMKDEEMKYDEMKNEEMKYDEMKDEEMKYDEM--------------------------------
FCR3         EEMKDEEMKDEEMEDVEMKDEEMKDEEMKYDEMKNEEMKYDEM------------------------------------------
D10          EEMKDEEMKDEEMEDVEMKDEEMKDEEMKYDEMKNEEMKYDEM------------------------------------------
HB3          EEMKDEEMKDEEMKDVEMKDEEMK------------------------------DEEI-----KYDEMKNEEMKYDEM-------
Wellcome     EEMKDEEMKDEEMEDVEMKDEEMKDEEMKYDEMKNEEMKYDEM------------------------------------------
Fcc2         EEMKDEEMKDEEMEDVEMKDEEMKDEEMKYDEMKNEEMKYDEM------------------------------------------
7G8          EEMKDEEMKDEEMKDVEMKDEEMKDEEMKYDEMKNEEMKYDEM------------------------------------------
D6           EEMKDEEMKDEEMKDVEMKDEEMKDEEMKYDEMKNEEMKYDEMKDEEMKYDEM--------------------------------
             ************* **********
```

MAL7P1.208 [RAMA] Repeat block 2

```
[P reichenowiDDMKDDEMKNDEMKYDEMKDE-------------------------QMEY
Dd2          ------------------KDEVMKDEEMKDE---------------QMKY
T9/96        -------------KDEVMKDEVMKDEEMKDE---------------QMKY
T9/102       ------------------KDEVMKDEEMKDE---------------QMKY
R033         ------------------KDEVMKDEEMKDEEMKDEVMKDEEMKDEQMKY
3D7          ------------------KDEVMKDEEMKDE-----VMKDEEMKDEQMKY
K1           ------------------KDEVMKDEEMKDE---------------QMKY
Palo Alto    ------------------KDEVMKDEEMKDE---------------QMKY
FCR3         ------------------KDEVMKDEEMKDE---------------QMKY
D10          ------------------KDEVMKDEEMKDE---------------QMKY
HB3          ------------------KDEVMKDEEMKDE-----VMKDEEMKDEQMKY
Wellcome     ------------------KDEVMKDEEMKDE---------------QMKY
Fcc2         ------------------KDEVMKDEEMKDE---------------QMKY
7G8          ------------------KDEVMKDEEMKDEEMKDE----------QMKY
D6           ------------------KDEVMKDEEMKDE---------------QMKY
                               *************               ****
```

MAL7P1.208 [RAMA] Repeat block 3

```
[P reichenowiEEF--------------------------------------------------KNEDKKKED]
Dd2          EEFKNEEFKNEEFKNEESKNEESKNEESKNEESKNEEFKNEESKNEEFKNEEFKNE-----D
T9/96        EEFKNEEFK-----NEESKNEESKNEESKNEESKNEEFKNEESKNEEFKNEEF---
T9/102       EEFKNEEFKNEEFKNEESKNEESKNEESKNEESKNEEFKNEESKNEEFKNEEFKNE-----D
R033         EEFK----------NEESKNEESKNEESK-----NEEFKNEESKNEEFKNEEFKNE-----D
3D7          EEFK----------NEESKNEESKNEESKNEESKNEEFKNEESKNEEFKNEEFKNE-----D
K1           EEFKNEEFKNEEFKNEESKNEESKNEESKNEESKNEEFKNEESKNEEFKNEEFKNE-----D
Palo Alto    EEFK----------NEESKNEESKNEESKNEESKNEEFKNEESKNEEFKNEEFKNE-----D
FCR3         EEFKNEEFKNEEFKNEESKNEESKNEESKNEESKNEEFKNEESKNEEFKNEEFKNE-----D
D10          EEFKNEEFKNEEFKNEESKNEESKNEESKNEESKNEEFKNEESKNEEFKNEEFKNE-----D
HB3          EEFK----------NEESKNEESKNEESKNEESKNEEFKNEESKNEEFKNEEF---------
Wellcome     EEFKNEEFKNEEFKNEESKNEESKNEESKNEESKNEEFKNEESKNEEFKNEEFKNE-----D
Fcc2         EEFKNEEFKNEEFKNEESKNEESKNEESKNEESKNEEFKNEESKNEEFKNEEFKNE-----D
7G8          EEFKNEEFKNEEFKNEESKNEESKNEESKNEESKNEEFKNEESKNEEFKNEEFKNE-----D
D6           EEFK----------NEESKNEESKNEESKNEESKNEEFKNEESKNEEFKNEEFKNE-----D
             ****          ***************     *******************
```

MAL7P1.208 [RAMA] Repeat block 4

```
[P reichenowiKEEKEEKE---]
Dd2          KEEKEEKEEKE
T9/96        KEEKEEKEEKE
T9/102       KEEKEEKEEKE
R033         KEEKEEKEEKE
3D7          KEEKEEKEEKE
K1           KEEKEEKEEKE
Palo Alto    KEEKEEKEEKE
FCR3         KEEKEEKEEKE
D10          KEEKEEKEEKE
HB3          KEEKEEKEEKE
Wellcome     KEEKEEKEEKE
Fcc2         KEEKEEKEEKE
7G8          KEEKEEKEEKE
D6           KEEKEEKEEKE
             ********
```

PF13_0348 [Rhop148] Repeat block 1

```
[P reichenowi NIDD-------DDNNN-------------I
Dd2          NIDDDNNNNNIDD----------NNNNNNI
T9/96        NIDD-NNNNNIDDNNNNNIDD--NNNNNNI
T9/102       NIDDDNNNNNIDD---------NNNNNNNI
R033         NIDDDNNNNNIDD---------NNNNNNNI
3D7          NIDDD------------------NNNNNNI
K1           NIDDDNNNNNIDD----------NNNNNNI
Palo Alto    NIDDDNNNNNIDDNNNNNNIDD-NNNNNNI
FCR3         NIDDDNNNNNIDD----------NNNNNNI
D10          NIDDDNNNNNIDD----------NNNNNNI
HB3          NIDDDNNNNNIDD----------NNNNNNI
Wellcome     NIDDDNNNNNIDD----------NNNNNNI
Fcc2         NIDDDNNNNNIDD----------NNNNNNI
7G8          NIDDDNNNNNIDD----------NNNNNNI
D6           NIDDDNNNNNIDD----------NNNNNNI
             ****                   *******
```

PF13_0348 [Rhop148] Repeat block 2

```
[P reichenowi NANTKMNTKMNTKMNTNANTITNTNTNTITNTNTNTITNTNTNTETN----------------------------------------------------]
Dd2           NANTNVNTNTNTNTNANVNTNVNTNTNTNANVNTNVNTNTNTNANVNTNVNTNTNTNANVNTNVNTNTNTNANVNTNVNTNTNT---------------
T9/96         NANTNTNANTNTNANVNTNVNTNTNTNANVNTNVNTNTNTNANVNTNVNTNTNTNANVNTNVNTNTNTN------------------------------
T9/102        NANTNVNTNTNTNTNANVNTNVNTNTNTNANVNTNVNTNTNTNANVNTNVNTNTNTNANVNTNVNTNTNTNANVNTNVNTNTNT---------------
R033          NANTNTNANTNTNANVNTNVNTNTNTNANVNTNVNTNTNTNANVNTNVNTNTNTNANVNTNVNTNTNTN------------------------------
3D7           NANTNTNANTNTNANVNTNVNTNVNTNVNTNVNTNTNTNVNTNTNTNVNTNTNTN--------------------------------------------
K1            NANTNTNANTNTNANVNTNVNTNTNTNANVNTNVNTNTNTNANVNTNVNTNTNTNANVNTNVNTNTNT-------------------------------
Palo Alto     NANTNTNANTNTNANVNTNVNTNVNTNTNTNANVNTNVNTNVNTNVNTNTNTNANINTNVNTNTNTN--------------------------------
FCR3          NANTNTNANTNTNANVNTNVNTNTNTNANVNTNVNTNTNTNANVNTNVNTNTNTNANVNTNVNTNTNTNANVNTNVNTNTNTNANVNTNVNTNTNT---
D10           NANTNTNANTNTNANVNTNVNTNTNTNANVNTNVNTNTNTNANVNTNVNTNTNTNANVNTNVNTNTNTNANVNTNVNTNTNTNTNANVNTNVNTNTNT-
HB3           NANTNTNANTNTNANVNTNVNTNTNTNANVNTNVNTNTNTNANINTNVNTNTNTN--------------------------------------------
Wellcome      NANTNTNANTNTNANVNTNVNTNTNTNANVNTNVNTNTNTNANVNTNVNTNTNTNANVNTNVNTNTNTNANVNTNVNTNTNTNANVNTNVNTNTNT---
Fcc2          NANTNTNANTNTNANVNTNVNTNTNTNANVNTNVNTNTNTNANVNTNVNTNTNTNANVNTNVNTNTNT-------------------------------
7G8           NANTNTNANTNTNANVNTNVNTNTNTNANVNTNVNTNTNTNANINTNVNTNTNTN--------------------------------------------
D6            NANTNTNANTNTNTNANVNTNVNTNTNTNANVNTNVNTNTNTNANVNTNVNTNTNVNTNTNTN------------------------------------
```

PF13_0348 [Rhop148] Repeat block 3

```
[P reichenowiNNKNNDNNNNNXNNNNN--------------------------------NNNN-YNNYNNI
Dd2          NNNNNDNNNNNDNNNNND------------NNNNINNNNNKKNNNNNNNNNNN----YNNI
T9/96        NNNNNDNNNNNDNNNNNDNNNNND------NNNNINNNNNKKKNNNNNNNNNNN---YNNI
T9/102       NNNNNDNNNNNDNNNNND------------NNNNINNNNNKKNNNNNNNNNNN----YNNI
R033         NNNNNDNNNNNDNNNNNDNNNNND------NNNNINNNNNKKNNNNNNNNNNN----YNNI
3D7          NNNNNDNNNNNDNNNNNDNNNNND------NNNNINNNNNKKNKNNNNNNNNN----YNNI
K1           NNNNNDNNNNNDNNNNND------------NNNNINNNNNKKNNNNNNNNNNN----YNNI
Palo Alto    NNNNNDNNNNNDNNNNNDNNNNND------NNNNINNNNNKKNNNNNNNNNNN----YNNI
FCR3         NNNNNDNNNNNDNNNNNDNNNNND------NNNNINNNNNKKNNNNNNNNNNN----YNNI
D10          NNNNNDNNNNNDNNNNNDNNNNND------NNNNINNNNNKKNNNNNNNNNNN----YNNI
HB3          NNNNNDNNNNNDNNNNNDNNNNNDNNNNNDNNNNINNNNNKKNNNNNNNNNNN----YNNI
Wellcome     NNNNNDNNNNNDNNNNNDNNNNND------NNNNINNNNNKKNNNNNNNNNNN----YNNI
Fcc2         NNNNNDNNNNNDNNNNND------------NNNNINNNNNKKNNNNNNNNNNN----YNNI
7G8          NNNNNDNNNNNDNNNNNDNNNNND------NNNNINNNNNKKNKNNNNNNNNN----YNNI
D6           NNNNNDNNNNNDNNNNNDNNNNND------NNNNINNNNNKKNNNNNNNNNNN----YNNI
             ******************            ************* ********     ****
```

PF13_0348 [Rhop148] Repeat block 4

```
[P reichenowiDSSDNNNNNNNNNNNRGSQEIHLNKCDILKNEEYNVSNEYKNKKFIDMSNKNGANVCVNINNIQDNRNNNN
Dd2          DSSDNNNNNNNNNN-------DSSNNNSSNNNSSNNNSS---------------------------NNNSN
T9/96        DSSDNNNNNNNNNNNN-----DSSNNNSSNNNSS--------------------------------NNNSN
T9/102       DSSDNNNNNNNNNN-------DSSNNNSSNNNSSNNNSS---------------------------NNNSN
R033         DSSDNNNNNNNNNN-------DSSNNNSSNNNSS--------------------------------NNNSN
3D7          DSSDNNNNNNNNNNN------DSSNNNSS-------------------------------------NNNSN
K1           DSSDNNNNNNNNNNNNNNNNNDSSNNNSSNNNSS--------------------------------NNNSN
Palo Alto    DSSDNNNNNNNNNNNN-----DSSNNNSS-------------------------------------NNNSN
FCR3         DSSDNNNNNNNNNNNN-----DSSNNNSS-------------------------------------NNNSN
D10          DSSDNNNNNNNNNNN------DSSNNNSSNNNSS--------------------------------NNNSN
HB3          DSSDNNNNNNNNNN-------DSSNNNSSNNNSS--------------------------------NNNSN
Wellcome     DSSDNNNNNNNNNNNN-----DSSNNNSS-------------------------------------NNNSN
Fcc2         DSSDNNNNNNNN---------DSSNNNSS-------------------------------------NNNSN
7G8          DSSDNNNNNNNNNN-------DSSNNNSS-------------------------------------NNNSN
D6           DSSDNNNNNNNNN--------DSSNNNSSNNNSS--------------------------------NNNSN
             ***********          ********                                     *****
```

PF13_0348 [Rhop148] Repeat block 5

```
[P reichenowi NIDNDKNNDSNNNNSSNNNNNC]
Dd2           NNDINNDINNNCSNNNNNNNNC
T9/96         NNDINNDINNNCSNNNNNNN-C
T9/102        NNDINNDINNNCSNNNNNNNNC
R033          NNDINNDINNNCSNNNNNNNNC
3D7           NNDINNDINNNCSNNNNNNNNC
K1            NNDINNDINNNCSNNNNNNNNC
Palo Alto     NNDINNDINNNCSNNNNNNNNC
FCR3          NNDINNDINNNCSNNNNNNN-C
D10           NNDINNDINNNCSNNNNNNN-C
HB3           NNDINNDINNNCSNNNNNNNNC
Wellcome      NNDINNDINNNCSNNNNNNN-C
Fcc2          NNDINNDINNNCSNNNNNNN-C
7G8           NNDINNDINNNCSNNNNNNNNC
D6            NNDINNDINNNCSNNNNNNNNC
              ******************** *
```

Pf14_0102 [RAP1] Repeat block 1

```
[P reichenowi SKSSSPSSTKSSSPS]
Dd2           LKSSSPSITKSSSPS
T9/96         SKSSSPSSTKSSSPS
T9/102        LKSSSPSITKSSSPS
R033          LKSSSPSSTKSSSPS
3D7           LKSSSPSSTKSSSPS
K1            LKSSSPSSTKSSSPS
Palo Alto     SKSSSPSSTKSSSPS
FCR3          LKSSSPSSTKSSSPS
D10           LKSSSPSSTKSSSPS
HB3           SKSSSPSSTKSSSPS
Wellcome      LKSSSPSSTKSSSPS
Fcc2          LKSSSPSSTKSSSPS
7G8           SKSSSPSSTKSSSPS
D6            LKSSSPSITKSSSPS
               ****** *******
```
