# Supplementary material for: Prospective Identification of Malaria Parasite Genes under Balancing Selection
Source: PLoS One. 2009 May 15;4(5):e5568. doi: 10.1371/journal.pone.0005568 (PMC2679211; doi:10.1371/journal.pone.0005568)
Supplement: Figure S5 — (1.42 MB PDF) [file pone.0005568.s005.pdf]

Alignment of all alleles observed in PF10_0348 (reference strain 3D7 from PlasmoDB included for comparison)

```
PlasmoDB 3D7 ref        MKKIYSIFFSLFILNLHIYIKNIKCNDLINYNDSNLRNGLLNNSLDLTNGLNNKDNSFIDSKIEEHENKSYQNKDNNISIVGQDVPITSVYSSKIINAND
Plasmodium reichenowi                                   DSNLRNGLLTNSLDATNGLNNKDNSFIDSKIEEHENNSYQNKDNNISIVGQDVPTTSVASSKIINAND
3D7                                                     DSNLRNGLLNNSLDLTNGLNNKDNSFIDSKIEEHENKSYQNKDNNISIVGQDVPITSVYSSKIINAND
RO33                                                    DSNLRNGLLNNSLDLTNGLNNKDNSFIDSKIEEHENKSYQNKDNNISIVGQDVPITSVDSSKIINAND
FCR3                                                    DSNLRNGLLNNSLDLTNGLNNKDNSFIDSKIEEHENKSYQNKDNNISIVGQDVPITSVDSSKIINAND
Wellcome                                                DSNLRNGLLNNSLDLTNGLNNKDNSFIDSKIEEHENKSYQNKDNNISIVGQDVPITSVDSSKIINAND
T996                                                    DSNLRNGLLNNSLDLTNGLNNKDNSFIDSKIEEHENKSYQNKDNNISIVGQDVPITSVDSSKIINAND
T9102                                                   DSNLRNGLLNNSLDLTNGLNNKDNSFIDSKIEEHENKSYQNKDNNISIVGQDVPITSVDSSKIINAND
K1                                                      DSNLRNGLLNNSLDLTNGLNNKDNSFIDSKIEEHENKSYQNKDNNISIVGQDVPITSVDSSKIINAND
Dd2                                                     DSNLRNGLLNNSLDLTNGLNNKDNSFIDSKIEEHENKSYQNKDNNISIVGQDVPITSVDSSKIINAND
D10                                                     DSNLRNGLLNNSLDLTNGLNNKDNSFIDSKIEEHENKSYQNKDNNISIVGQDVPITSVDSSKIINAND
FCC2 A                                                  DSNLRNGLLNNSLDLTNGLNNKDNSFIDSKIEEHENKSYQNKDNNISIVGQDVPITSVDSSKIINAND
D6 A                                                    DSNLRNGLLNNSLDLTNGLNNKDNSFIDSKIEEHENKSYQNKDNNISIVGQDVPITSVDSSKIINAND
Palo Alto A                                             DSNLRNGLLNNSLDLTNGLNNKDNSFIDSKIEEHENKSYQNKDNNISIVGQDVPITSVDSSKIINAND
HB3                                                     DSNLRNGLLNNSLDLTNGLNNKDNSFIDSKIEEHENKSYQNKDNNISIVGQDVPITSVDSSKIINAND
7G8                                                     DSNLRNGLLNNSLDLTNGLNNKDNSFIDSKIEEHENKSYQNKDNNISIVGQDVPITSVDSSKIINAND
FCC2 B                                                  DSNLRNGLLNNSLDLTNGLNNKDNSFIDSKIEEHENKSYQNKDNNISIVGQDVPITSVDSSKIINAND
D6 B                                                    DSNLRNGLLNNSLDLTNGLNNKDNSFIDSKIEEHENKSYQNKDNNISIVGQDVPITSVDSSKIINAND
Palo Alto B                                                                NNKDNSFIDSKIEEHENKSYQNKDNNISIVGQDVPITSVDSSKIINAND

PlasmoDB 3D7 ref        LEGNSIDDTKGLSVTNSGFDDGSAFGGGLPFSGYSPLQGNHNKCPDENFCKGIKNVLSCPPKNSTGRNGDWISVAVKESSTTNKGVLVPPRRTKLCLR-N
Plasmodium reichenowi   LEGNSIDNTKGLSVTNSGFDDGSAFGGGLPLSGYSPLQGNHNKCPSENFCEGIKNALSCPEKHFTGRNGDWISVAVKESSTTNKGVLVPPRRRKLCLR-N
3D7                     LEGNSIDDTKGLSVTNSGFDDGSAFGGGLPFSGYSPLQGNHNKCPDENFCKGIKNVLSCPPKNSTGRNGDWISVAVKESSTTNKGVLVPPRRTKLCLR-N
RO33                    LEGNSIDDTKGLSVTNSGFDDGSAFGGGLPFSGYSPLQGNHNKCPDENFCKGIKNALSCPPKNSTGRNGDWISVTVKESSTTNKGVLVPPRRTKLCLR-N
FCR3                    LEGNSIDDTKGLSVTNSGFDDGSAFGGGLPFSGYSPLQGNHNKCPDENFCKGIKNVLSCPPKNSTGRNRDWISVAVKESSTTNKGVLVPPRRTKLCLR-N
Wellcome                LEGNSIDDTKGLSVTNSGFDDGSAFGGGLPFSGYSPLQGNHNKCPDENFCKGIKNVLSCPPKNSTGRNRDWISVAVKESSTTNKGVLVPPRRTKLCLR-N
T996                    LEGNSIDDTKGLSVTNSGFDDGSAFGGGLPFSGYSPLQGNHNKCPDENFCKGIKNVLSCPPKNSTGRNGDWISVAVKESSTTNKGVLVPPRRKKLCLR-N
T9102                   LEGNSIDDTKGLSVTNSGFDDGSAFGGGLPFSGYSPLQGNHNKCPDENFCKGIKNALSCPPKNSTGRNGDWISVAVKESSTTNKGVLVPPRRKKLCLR-N
K1                      LEGNSIDDTKGLSVTNSGFDDGSAFGGGLPFSGYSPLQGNHNKCPDENFCKGIKNALSCPPKNSTGRNGDWISVAVKESSTTNKGVLVPPRRTKLCLR-N
Dd2                     LEGNSIDDTKGLSVTNSGFDDGSAFGGGLPFSGYSPLQGNHNKCPDENFCKGIKNALSCPPKNSTGRNGDWISVAVKESSTTNKGVLVPPRRTKLCLR-N
D10                     LEGNSIDDTKGLSVTNSGFDDGSAFGGGLPFSGYSPLQGNHNKCPDENFCKGIKNALSCPPKNSTGRNGDWISVAVKESSTTNKGVLVPPRRTKLCLR-N
FCC2 A                  LEGNSIDDTKGLSVTNSGFDDGSAFGGGLPFSGYSPLQGNHNKCPDENFCKGIKNVLSCPPKNSTGRNGDWASSNVRNFLTVNKGVLVPPRRKQMCFRIN
D6 A                    LEGNSIDDTKGLSVTNSGFDDGSAFGGGLPFSGYSPLQGNHNKCPDKNFCNGIQNVPNCPLKNFTGTKGDWASSNVRNFLTVNKGVLVPPRRKQMCFRIN
Palo Alto A             LEGNSIDDTKGLSVTNSGFDDGSAFGGGLPFSGYSPLQGNHNKCPDKNFCNGIQNVPNCPLKNFTGTKGDWASSNVRNFLTVNKGVLVPPRRKQMCFRIN
HB3                     LEGNSIDDTKGLSVTNSGFDDGSAFGGGLPFSGYSPLQGNHNKCPDKNFCNGIQNVPNCPLKDFTGTKGDWASSNVRNFLTVNKGVLVPPRRKQMCFRIN
7G8                     LEGNSIDDTKGLSVTNSGFDDGSAFGGGLPFSGYSPLQGNHNKCPDKNFCNGIQNVPNCPLKDFTGTKGDWASSNVRNFLTVNKGVLVPPRRKQMCFRIN
FCC2 B                  LEGNSIDDTKGLSVTNSGFDDGSAFGGGLPFSGYSPLQGNHNKCPDENFCKGIKNVLSCPPKNSTGRNGDWISVNVKESSTTNKGVLVPPRRKQMCFRIN
D6 B                    LEGNSIDDTKGLSVTNSGFDDGSAFGGGLPFSGYSPLQGNHNKCPDENFCKGIKNVLSCPPKNSTGRNGDWISVNVKESSTTNKGVLVPPRRKQMCFRIN
Palo Alto B             LEGNSIDDTKGLSVTNSGFDDGSAFGGGLPFSGYSPLQGNHNKCPDENFCKGIKNVLSCPPKNSTGRNGDWISVNVKESSTTNKGVLVPPRRKQMCFRIN
```

PlasmoDB 3D7 ref	INKVWHRIKDEKNFKEEFVKVALGESNALMKHYKEKNLNALTAIKYGFSDMGDIIKGTDLIDYQITKNINRALDKILRNETSN-DKIKKRVDWWEANKSA
Plasmodium reichenowi	ITHIWYRINDEKNFKEKFFKDALGESNALIKYYKEKNLNPLTAIKYGFSDMGDIIKGTDLIDYQITKNINRALDKILGNERSN-AEIKNRVDWWEANKSE
D7	INKVWHRIKDEKNFKEEFVKVALGESNALMKHYKEKNLNALTAIKYGFSDMGDIIKGTDLIDYQITKNINRALDKILRNETSN-DKIKKRVDWWEANKSA
RO33	INKVWHRIKDEKNFKEEFVKVALGESNALMKHYKEKNLNALTAIKYGFSDMGDIIKGTDLIDYQITKNINRALDKILGNETSN-DKIKKRVDWWEANKSA
FCR3	INKVWHRIKDEKNFKEEFVKVALGESNALMKHYKEKNLNALTAIKYGFSDMGDIIKGTDLIDYQITKNINRALDKILGNETSN-DKIKKRVDWWEANKSA
Wellcome	INKVWHRIKDEKNFKEEFVKVALGESNALMKHYKEKNLNALTAIKYGFSDMGDIIKGTDLIDYQITKNINRALDKILGNETSN-DKIKKRVDWWEANKSA
T996	INQVWHRIKDEKNFKEEFVKVALGESNALMKHYKEKNLNALTAIKYGFSDMGDIIKGTDLIDYQITKNINRALDKILGNETSN-DKIKKRVDWWEANKSA
T9102	INQVWHRIKDEKNFKEEFVKVALGESNALMKHYKEKNLNALTAIKYGFSDMGDIIKGTDLIDYQITKNINRALDKILGNETSN-DKIKKRVDWWEANKSA
K1	INKVWHRIKDEKNFKEEFVKVALGESNALMKHYKEKNLNALTAIKYGFSDMGDIIKGTDLIDYQITKNINRALDKILGNETSN-DKIKKRVDWWEANKSA
Dd2	INKVWHRIKDEKNFKEEFVKVALGESNALMKHYKEKNLNALTAIKYGFSDMGDIIKGTDLIDYQITKNINRALDKILGNETSN-DKIKKRVDWWEANKSA
D10	INKVWHRIKDEKNFKEEFVKVALGESNALMKHYKEKNLNALTAIKYGFSDMGDIIKGTDLIDYQITKNINRALDKILGNETSN-DKIKKRVDWWEANKSA
FCC2 A	INNFPELKKTEGKFENFIYSSAGSEAKQLIKLYGNNTEKALQAMKYGFADIGNIVQGNDMIDTPTSNKTKTYLEEVLGKQYKNVNDPKDAKTWWIQNKHR
D6 A	INNFPELKKTEGKFENFIYSSAGSEAKQLIKLYGNNTEKALQAMKYGFADIGNIVQGNDMIDTPTSNKTKTYLEEVLGKQYKNVNDPKDAKTWWIQNKHR
Palo Alto A	INNFPELKKTEGKFENFIYSSAGSEAKQLIKLYGNNTEKALQAMKYGFADIGNIVQGNDMIDTPTSNKTKTYLEEVLGKQYKNVNDPKDAKTWWIQNKHR
HB3	INNFPKLKKTEGKFENFIYSSAGSEAKQLIKLYGNNTEKALQAMKYGFADIGNIVQGNDMIDTPTSNKTKTYLEEVLGKQYKNVNDPKDAKTWWIQNKHR
7G8	INNFPKLKKTEGKFENFIYSSAGSEAKQLIKLYGNNTEKALQAMKYGFADIGNIVQGNDMIDTPTSNKTKTYLEEVLGKQYKNVNDPKDAKTWWIQNKHR
FCC2 B	INNFPKLKKTEGKFENFIYSSAGSEAKQLIKLYGNNTEKAHQAIRYSFADIGNIIRGDDMMDTPTSKETITYLEKVLKIYNENNDKPKDAKKWWTENRHH
D6 B	INNFPKLKKTEGKFENFIYSSAGSEAKQLIKLYGNNTEKAHQAIRYSFADIGNIIRGDDMMDTPTSKETITYLEKVLKIYNENNDKPKDAKKWWTENRHH
Palo Alto B	INNFPKLKKTEGKFENFIYSSAGSEAKQLIKLYGNNTEKAHQAIRYSFADIGNIIRGDDMMDTPTSKETITYLEKVLKIYNENNDKPKDAKKWWTENRHH

PlasmoDB 3D7 ref	FWDAFMCGYKVHIGNKPCPEHDNMDRIPQYLRWFREWGTYVCSEYKNKFEDVIKLCNIQQFTNQDDSQLLEISKKDKCKEALKHYEEWVNRRRPEWKGQC
Plasmodium reichenowi	LWDALMCGYKIHIGNKACPEHDNMDRIPQYLRWFREWGTYVCSEYKNKFENVIKLCNIQQITNQDDSQLLGLSNDDKCKDALKHYEEWINRRRPEWKGQC
3D7	FWDAFMCGYKVHIGNKPCPEHDNMDRIPQYLRWFREWGTYVCSEYKNKFEDVIKLCNIQQFTNQDDSQLLEISKKDKCKEALKHYEEWVNRRRPEWKGQC
RO33	FWDAFMCGYKVHI.NKPCPEHDNMDRIPQYLRWFREWGTYVCSEYKNKFENVIELCNVRQITNQNDSQLLEISKKDKCKGALKHYEEWVNRRRPEWKGQC
FCR3	FWDAFMCGYKVHIGNKPCPEHDNMDRIPQYLRWFREWGTYVCSEYKNKFENVIELCNVRQITNQNDSQLLEISKKDKCKGALKHYEEWVNRRRPEWKGQC
Wellcome	FWDAFMCGYKVHIGNKPCPEHDNMDRIPQYLRWFREWGTYVCSEYKNKFENVIELCNVRQITNQNDSQLLEISKKDKCKGALKHYEEWVNRRRPEWKGQC
T996	FWDAFMCGYKVHIGNKPCPEHDNMDRIPQYLRWFREWGTYVCSEYKNKFEDVIKLCNIQQFTNQDDSQLLEISKKDKCKEALKHYEEWVNRRRPEWKGQC
T9102	FWDAFMCGYKVHIGNKPCPEHDNMDRIPQYLRWFREWGTYVCSEYKNKFEDVIKLCNIQQFTNQNDSQLLEISKKDKCKGALKHYEEWVNRRRPEWKGQC
K1	FWDAFMCGYKVHIGNKPCPEHDNMDRIPQYLRWFREWGTYVCSEYKNKFENVIELCNVRQITNQNDSQLLEISKKDKCKGALKHYEEWVNRRRPEWKGQC
Dd2	FWDAFMCGYKVHIGNKPCPEHDNMDRIPQYLRWFREWGTYVCSEYKNKFENVIELCNVRQITNQNDSQLLEISKKDKCKGALKHYEEWVNRRRPEWKGQC
D10	FWDAFMCGYKVHIGNKPCPEHDNMDRIPQYLRWFREWGTYVCSEYKNKFEDVIKLCNIQQFTNQDDSQLLEISKKDKCKEALKHYEEWVNRRRPEWKGQC
FCC2 A	VWDAMMCGYKVHIGNKPCPEHDNMDRIPQYLRWFREWGTYVCSEYKNKFENVIELCNVRQITNQNDSQLLEISKKDKCKGALKHYEEWVNRRRPEWKGQC
D6 A	VWDAMMCGYKVHIGNKPCPEHDNMDRIPQYLRWFREWGTYVCSEYKNKFENVIELCNVRQITNQNDSQLLEISKKDKCKGALKHYEEWVNRRRPEWKGQC
Palo Alto A	VWDAMMCGYKVHIGNKPCPEHDNMDRIPQYLRWFREWGTYVCSEYKNKFENVIELCNVRQITNQNDSQLLEISKKDKCKGALKHYEEWVNRRRPEWKGQC
HB3	VWDAMMCGYQYEKKDNKCTGYGNIYDIPQYLRWFREWGTYVCSEYKNKFEDVIKLCNIQQFTNQDDSQLLEISKKDKCKEALKHYEEWVNRRRPEWKGQC
7G8	VWDAMMCGYQYEKKDNKCTGYGNIYDIPQYLRWFREWGTYVCSEYKNKFEDVIKLCNIQQFTNQDDSQLLEISKKDKCKEALKHYEEWVNRRRPEWKGQC
FCC2 B	VWEAMMCGYQSAQKDNQCTGYGNIDDIPQYLRWFREWGTYVCSEYKNKFEDVIKLCNIQQFTNQDDSQLLEISKEDKCKGALKHYEEWVNRRRPEWKGQC
D6 B	VWEAMMCGYQSAQKDNQCTGYGNIDDIPQYLRWFREWGTYVCSEYKNKFEDVIKLCNIQQFTNQDDSQLLEISKEDKCKGALKHYEEWVNRRRPEWKGQC
Palo Alto B	VWEAMMCGYQSAQKDNQCTGYGNIDDIPQYLRWFREWGTYVCSEYKNKFEDVIKLCNIQQFTNQDDSQLLEISKEDKCKGALKHYEEWVNRRRPEWKGQC

PlasmoDB 3D7 ref	DKFEKEKSKYEDTKSITAEKYLKEICSECDCKYKDLDNTFKEFKDNVTLLKAVIDNKKNQDSLTTTSLSTSINSVRDSSNLDQRGNITTSQGNSHRATVV
Plasmodium reichenowi	DKFEKEKNKYEDTKSITAEKYLKEICSECDCKYKDLDNTFKEFKDKVTLLKAVIENKKNQDSLTTTS-----------------------QGNSHRETVV
3D7	DKFEKEKSKYEDTKSITAEKYLKEICSECDCKYKDLDNTFKEFKDNVTLLKAVIDNKKNQDSLTTTSLSTSINSVRDSSNLDQRGNITTSQGNSHRATVV
RO33	DKFEKEKSKYEDTKSRTAEIYLKEICSECDCKYKDLDNTFKEFKDNVTLLKAVIDNKKNQDSLTTTSLSTSINSVRDSSNLDQRGNITTSQGNSHRATVV
FCR3	DKFEKEKSKYEDTKSRTAEIYLKEICSECDCKYKDLDNTFKEFKDNVTLLKAVIDNKKNQDSLTTTSLSTSINSVRDSSNLDQRGNITTSQGNSHRATVV
Wellcome	DKFEKEKSKYEDTKSRTAEIYLKEICSECDCKYKDLDNTFKEFKDNVTLLKAVIDNKKNQDSLTTTSLSTSINSVRDSSNLDQRGNITTSQGNSHRATVV
T996	DKFEKEKSKYEDTKSITAEKYLKEICSECDCKYKDLDNTFKEFKDNVTLLKAVIDNKKNQDSLTTTSLSTSINSVRDSSNLDQRGNITTSQGNSHRATVV
T9102	DKFEKEKSKYEDTKSRTAEIYLKEICSECDCKYKDLDNTFKEFNDNVTLLKAVIDNKKNQDSLTTTSLSTSINSVRDSSNLDQRGNITTSQGNSHRATVV
K1	DKFEKEKSKYEDTKSRTAEIYLKEICSECDCKYKDLDNTFKEFKDNVTLLKAVIDNKKNQDSLTTTSLSTSINSVRDSSNLDQRGNITTSQGNSHRATVV
Dd2	DKFEKEKSKYEDTKSRTAEIYLKEICSECDCKYKDLDNTFKEFKDNVTLLKAVIDNKKNQDSLTTTSLSTSINSVRDSSNLDQRGNITTSQGNSHRATVV
D10	DKFEKEKSKYEDTKSITAEKYLKEICSECDCKYKDLDNTFKEFKDNVTLLKAVIDNKKNQDSLTTTSLTTSINSGRDSSNLNQRGNITTSQGNSHRATVV
FCC2 A	DKFEKEKSKYEDTKSRTAEIYLKEICSECDCKYKDLDNTFKEFNDNVTLLKAVIDNKKNQDSLTTTSLSTSINSVRDSSNLDQRGNITTSQGNSHRATVV
D6 A	DKFEKEKSKYEDTKSRTAEIYLKEICSECDCKYKDLDNTFKEFNDNVTLLKAVIDNKKNQDSLTTTSLSTSINSVRDSSNLDQRGNITTSQGNSHRATVV
Palo Alto A	DKFEKEKSKYEDTKSRTAEIYLKEICSECDCKYKDLDNTFKEFNDNVTLLKAVIDNKKNQDSLTTTSLSTSINSVRDSSNLDQRGNITTSQGNSHRATVV
HB3	DKFEKEKSKYEDTKSITAEKYLKEICSECDCKYKDLDNTFKEFKDNVTLLKAVIDNKKNQDSLTTTSLTTSINSGRDSSNLNQRGNITTSQGNSHRATVV
7G8	DKFEKEKSKYEDTKSITAEKYLKEICSECDCKYKDLDNTFKEFKDNVTLLKAVIDNKKNQDSLTTTSLTTSINSGRDSSNLNQRGNITTSQGNSHRATVV
FCC2 B	DKFEKEKSKYEDTKSRTAEIYLKQKCSECDCKYKDLDNTFKEFKDNVTLLKAVIDNKKNQDSLTTTSLSTSINSVRDSSNLDQRGNITTSQGNSHRATVV
D6 B	DKFEKEKSKYEDTKSRTAEIYLKQKCSECDCKYKDLDNTFKEFKDNVTLLKAVIDNKKNQDSLTTTSLSTSINSVRDSSNLDQRGNITTSQGNSHRATVV
Palo Alto B	DKFEKEKSKYEDTKSRTAEIYLKQKCSECDCKYKDLDNTFKEFKDNVTLLKAVIDNKKNQDSLTTTSLSTSINSVRDSSNLDQRGNITTSQGNSHRATVV

PlasmoDB 3D7 ref	QQVDQTNRLDNVNSVTQRGNNNYNNNLERGLGSGALPGTNIITEEKYSLELIKLTSKDEEDIIKHNEDVREEIEEQQEDIEEDEEELENEGEE-------
Plasmodium reichenowi	QQPDRTNILDNVNAVTQRGNNNHNNNLERGLGHGALPGTNIITEEKKLLEFINLTSKDEEDITKHNEDVREEIEEQQEEIEEDEEELENEGEEQQEEIEE
3D7	QQVDQTNRLDNVNSVTQRGNNNYNNNLERGLGSGALPGTNIITEEKYSLELIKLTSKDEEDIIKHNEDVREEIEEQQEDIEEDEEELENEGEE-------
RO33	QQADQTNRLDNVNSVTQRGNNNYNNNLERGLGSGALPGTNIITEEKYSLELIKLTSKDEEDIIKHNEDVREEIEEQQEDIEEDEEELENEGEE-------
FCR3	QQADQTNRLDNVNSVTQRGNNNYNNNLERGLGSGALPGTNIITEEKYSLELIKLTSKDEEDIIKHNEDVREEIEEQQEDIEEDEEELENEGEE-------
Wellcome	QQADQTNRLDNVNSVTQRGNNNYNNNLERGLGSGALPGTNIITEEKYSLELIKLTSKDEEDIIKHNEDVREEIEEQQEDIEEDEEELENEGEE-------
T996	QQADQTNRLDNVNSVTQRGNNNYNNNLERGLGSGALPGTNIITEEKYSLELIKLTSKDEEDIIKHNEDVREEIEEQQEDIEEDEEELENEGEE-------
T9102	QQADQTNRLDNVNSVTQRGNNNYNNNLERGLGSGALPGTNIITEEKYSLELIKLTSKDEEDIIKHNEDVREEIEEQQEDIEEDEEELENEGEE-------
K1	QQADQTNRLDNVNSVTQRGNNNYNNNLERGLGSGALPGTNIITEEKYSLELIKLTSKDEEDIIKHNEDVREEIEEQQEDIEEDEEELENEGEE-------
Dd2	QQADQTNRLDNVNSVTQRGNNNYNNNLERGLGSGALPGTNIITEEKYSLELIKLTSKDEEDIIKHNEDVREEIEEQQEDIEEDEEELENEGEE-------
D10	QQADQTNRLDNVNSVTQRGNNNYNNNLERGLGSGALPGTNIITEEKYSLELIKLTSKDEEDIIKHNEDVREEIEEQQEDIEEDEEELENEGEE-------
FCC2 A	QQADQTNRLDNVNSVTQRGNNNYNNNLERGLGSGALPGTNIITEEKYSLELIKLTSKDEEDIIKHNEDVREEIEEQQEDIEEDEEELENEGEE-------
D6 A	QQADQTNRLDNVNSVTQRGNNNYNNNLERGLGSGALPGTNIITEEKYSLELIKLTSKDEEDIIKHNEDVREEIEEQQEDIEEDEEELENEGEE-------
Palo Alto A	QQADQTNRLDNVNSVTQRGNNNYNNNLERGLGSGALPGTNIITEEKYSLELIKLTSKDEEDIIKHNEDVREEIEEQQEDIEEDEEELENEGEE-------
HB3	QQADQTNRLDNVNSVTQRGNNNYNNNLERGLGSGALPGTNIITEEKYSLELIKLTSKDEEDIIKHNEDVREEIEEQQEDIEEDEEELENEGEE-------
7G8	QQADQTNRLDNVNSVTQRGNNNYNNNLERGLGSGALPGTNIITEEKYSLELIKLTSKDEEDIIKHNEDVREEIEEQQEDIEEDEEELENEGEE-------
FCC2 B	QQADQTNRLDNVNSVTQRGNNNYNNNLERGLGSGALPGTNIITEEKYSLELIKLTSKDEEDIIKHNEDVREEIEEQQEDIEEDEEELENEGEE-------
D6 B	QQADQTNRLDNVNSVTQRGNNNYNNNLERGLGSGALPGTNIITEEKYSLELIKLTSKDEEDIIKHNEDVREEIEEQQEDIEEDEEELENEGEE-------
Palo Alto B	QQADQTNRLDNVNSVTQRGNNNYNNNLERGLGSGALPGTNIITEEKYSLELIKLTSKDEEDIIKHNEDVREEIEEQQEDIEEDEEELENEGEE-------

PlasmoDB 3D7 ref	-----------TKEEDDEEKNETNDTED---TDDTEDTEDIEEENKEKELSNQQQSEKKSISKVDEDSYRILSVSYKDNNEVKNVAESIVKKLFSLFNDN
Plasmodium *reichenowi*	DEEELENEGEETKEEDDEEKNETNDTED------------IEEENKEKGLSNQKQSEEKSISKVDEDSYRILSVSYKDNNEVKNVPESIVKKLFSLFNDN
3D7	-----------TKEEDDEEKNETNDTED---TDDTEDTEDIEEENKEKELSNQQQSEKKSISKVDEDSYRILSVSYKDNNEVKNVAESIVKKLFSLFNDN
RO33	-----------TKEEDDEEKNETNDTED---TDDTEDTEDIEEENKEKELSNQQQSEKKSISKVDEDSYRILSVSYKDNNEVKNVAESIVKKLFSLFNDN
FCR3	-----------TKEEDDEEKNETNDTED---TDDTEDTEDIEEENKEKELSNQQQSEKKSISKVDEDSYRILSVSYKDNNEVKNVAESIVKKLFSLFNDN
Wellcome	-----------TKEEDDEEKNETNDTED---TDDTEDTEDIEEENKEKELSNQQQSEKKSISKVDEDSYRILSVSYKDNNEVKNVAESIVKKLFSLFNDN
T996	-----------TKEEDDEEKNETNDTEDTEDTDDTEDTEDIEEENKEKELSNQQQSEKKSISKVDEDSYRILSVSYKDNNEVKNVAESIVKKLFSLFNDN
T9102	-----------TKEEDDEEKNETNDTED---TDDTEDTEDIEEENKEKELSNQQQSEKKSISKVDEDSYRILSVSYKDNNEVKNVAESIVKKLFSLFNDN
K1	-----------TKEEDDEEKNETNDTED---TDDTEDTEDIEEENEEKELSNQQQSEKKSISKVDEDSYRILSVSYKDNNEVKNVAESIVKKLFSLFNDN
Dd2	-----------TKEEDDEEKNETNDTED---TDDTEDTEDIEEENEEKELSNQQQSEKKSISKVDEDSYRILSVSYKDNNEVKNVAESIVKKLFSLFNDN
D10	-----------TKEEDDEEKNETNDTED---TDDTEDTEDIEEENEEKELSNQQQSEKKSISKVDEDSYRILSVSYKDNNEVKNVAESIVKKLFSLFNDN
FCC2 A	-----------TKEEDDEEKNETNDTED---TDDTEDTEDIEEENKEKELSNQQQSEKKSISKVDEDSYRILSVSYKDNNEVKNVAESIVKKLFSLFNDN
D6 A	-----------TKEEDDEEKNETNDTED---TDDTEDTEDIEEENKEKELSNQQQSEKKSISKVDEDSYRILSVSYKDNNEVKNVAESIVKKLFSLFNDN
Palo Alto A	-----------TKEEDDEEKNETNDTED---TDDTEDTEDIEEENKEKELSNQQQSEKKSISKVDEDSYRILSVSYKDNNEVKNVAESIVKKLFSLFNDN
HB3	-----------TKEEDDEEKNETNDTED---TDDTEDTEDIEEENKEKELSNQQQSEKKSISKVDEDSYRILSVSYKDNNEVKNVAESIVKKLFSLFNDN
7G8	-----------TKEEDDEEKNETNDTED---TDDTEDTEDIEEENKEKELSNQQQSEKKSISKVDEDSYRILSVSYKDNNEVKNVAESIVKKLFSLFNDN
FCC2 B	-----------TKEEDDEEKNETNDTEDTED---------IEEENKEKELSNQQQSEKKSISKVDEDSYRILSVSYKDNNEVKNVAESIVKKLFSLFNDN
D6 B	-----------TKEEDDEEKNETNDTEDTED---------IEEENKEKELSNQQQSEKKSISKVDEDSYRILSVSYKDNNEVKNVAESIVKKLFSLFNDN
Palo Alto B	-----------TKEEDDEEKNETNDTEDTED---------IEEENKEKELSNQQQSEKKSISKVDEDSYRILSVSYKDNNEVKNVAESIVKKLFSLFNDN

PlasmoDB 3D7 ref	NNLETIFKGLTEDMTDLFQK.
Plasmodium *reichenowi*	NNLETIFKGLTEDMTNLFQK.
3D7	NNLETIFKGLTED
RO33	NNLETIFKGLTED
FCR3	NNLETIFKGLTED
Wellcome	NNLETIFKGLTED
T996	NNLETIFKGLTED
T9102	NNLETIFKGLTED
K1	NNLETIFKGLTED
Dd2	NNLETIFKGLTED
D10	NNLETIFKGLTED
FCC2 A	NNLETIFKGLTED
D6 A	NNLETIFKGLTED
Palo Alto A	NNLETIFKGLTED
HB3	NNLETIFKGLTED
7G8	NNLETIFKGLTED
FCC2 B	NNLETIFKGLTED
D6 B	NNLETIFKGLTED
Palo Alto B	NNLETIFKGLTED

This alignment represents all sequences observed, all B sequences were removed for MK analysis. The stop codon in RO33 is represented here in red but removed from analysis.
