# Supplementary material for: Prospective Identification of Malaria Parasite Genes under Balancing Selection
Source: PLoS One. 2009 May 15;4(5):e5568. doi: 10.1371/journal.pone.0005568 (PMC2679211; doi:10.1371/journal.pone.0005568)
Supplement: Figure S6 — (0.03 MB DOC) [file pone.0005568.s006.doc]

Supplementary Figure S6. Affect of removing repeat sequences on calculated crude nucleotide diversity (π) among the *P. falciparum* laboratory isolates for the 15 genes with repeats studied in Table 1. Broken lines join the values for fhe five genes with repeats that were subsequently studied in a population analysis in Table 2. The alignments had repeats removed for all analyses in the manuscript (Tables 1 and 2, and Figures 1-3)
