# Supplementary material for: Prospective Identification of Malaria Parasite Genes under Balancing Selection
Source: PLoS One. 2009 May 15;4(5):e5568. doi: 10.1371/journal.pone.0005568 (PMC2679211; doi:10.1371/journal.pone.0005568)
Supplement: Table S1 — (0.05 MB DOC) [file pone.0005568.s007.doc]

Supplementary Table S1: Accession Numbers for gene sequences derived from a panel of 14 *P. falciparum* cultured isolates and *P. reichenowi*

| **Gene** | **Locus** | **Accession Numbers** |
| --- | --- | --- |
| *Pf12/6-cys* | PFF0615c | Pf - FJ406677 - FJ406694; Pr - FJ406695 |
| *Pf92/6-cys* | Pf13_0338 | Pf - FJ406623 - FJ406639; Pr - FJ406640 |
| *Pf38/6-cys* | PFE0395c | Pf - FJ406659 - FJ406676; Pr ^1^ |
| *Pf113* | PF14_0201 | Pf - FJ406642 - FJ406658; Pr -FJ406641 |
| *MSP10* | PFF0995c | Pf - FJ406606 - FJ406622; Pr - FJ406696 |
| *MSP6* | PF10_0346 | Pf - FJ406772 - FJ406788; Pr - FJ406771 |
| *MSP3/6-like* | PF10_0347 | Pf - FJ406828 - FJ406844; Pr - FJ406827 |
| *MSP3/6-like* | PF10_0348 | Pf - FJ406846 - FJ406862; Pr - FJ406845 |
| *MSP3/6-like* | PF10_0352 | Pf - FJ406864 - FJ406880; Pr - FJ406863 |
| *MSP7* | PF13_0197 | Pf - FJ406790 - FJ406807; Pr - FJ406789 |
| *MRSP1* | PF13_0196 | Pf - FJ406697 - FJ406713; Pr - FJ406714 |
| *MRSP2* | MAL13P1.174 | Pf - FJ406715 - FJ406731; Pr - FJ406732 |
| *MRSP3* | PF13_0193 | Pf - FJ406930 - FJ406947; Pr - FJ406929 |
| *MRSP4* | MAL13P1.173 | Pf - FJ406733 - FJ406750; Pr - FJ406751 |
| *MRSP5* | Pf13_0191 | Pf - FJ406752 - FJ406769; Pr - FJ406770 |
| *MRSP-like* | PF13_0192 | Pf - FJ406882 - FJ406895; Pr - FJ406881 |
| *MRSP-like* | PF13_0194 | Pf - FJ406897 - FJ406910; Pr - FJ406896 |
| *MSP9/ABRA* | PFL1385c | Pf - FJ406809 - FJ406826; Pr - FJ406808 |
| *SERA 5* | PFB0340c | Pf - FJ406911 - FJ406927; Pr - FJ406928 |
| *RAMA* | MAL7P1.208 | Pf - FJ406985 - FJ407002; Pr - FJ407003 |
| *Rhop148* | PF13_0348 | Pf - FJ407032 - FJ407047; Pr - FJ406948 |
| *Prohibitin* | PF10_0144 | Pf - FJ406949 - FJ406965; Pr - FJ406966 |
| *RAP1* | PF14_0102 | Pf - FJ407004 - FJ407014; Pr ^2^ |
| *RAP2* | PFE0080c | Pf - FJ407015 - FJ407022; Pr ^3^ |
| *RAP3* | PFE0075c | Pf - FJ407023 - FJ407031; Pr ^4^ |
| *Pf34* | PFD0955w | Pf - FJ406967 - FJ406983; Pr - FJ406984 |

Pf: *Plasmodium falciparum*

Pr: *Plasmodium reichenowi*

Pr ^1^ - EF123272

Pr ^2^ - U20986

Pr ^3^ - Derived from Sanger shotgun sequence: reich91f07.q1k, reich581b11.q1k, reich301f10.q1k

Pr ^4^ - Derived from Sanger shotgun sequence: reich308g09.p1k, reich451f07.p1k, reich29g09.q1k

reich1212e05.q1k, reich451g08.q1k, reich308g09.q1k

For PF10_0348, the extra ‘B sequences’ in three of the isolates were deposited in FJ556419 - FJ556421
