# Supplementary material for: Prospective Identification of Malaria Parasite Genes under Balancing Selection
Source: PLoS One. 2009 May 15;4(5):e5568. doi: 10.1371/journal.pone.0005568 (PMC2679211; doi:10.1371/journal.pone.0005568)
Supplement: Table S2 — (0.03 MB DOC) [file pone.0005568.s008.doc]

Supplementary Table S2: Accession Numbers for gene sequences derived from a population sample of *P. falciparum* in The Gambia

| **Gene** | **Locus** | **Accession Numbers** |
| --- | --- | --- |
| *Pf92/6-cys* | Pf13_0338 | FJ556095 - FJ556181 |
| *Pf38/6-cys* | PFE0395c | FJ556182 - FJ556269 |
| *MSP3/6-like* | PF10_0348 | FJ556029 - FJ556094 |
| *MSP7* | PF13_0197 | FJ555866 - FJ555950 |
| *SERA 5* | PFB0340c | FJ555696 - FJ555751 |
| *RAMA* | MAL7P1.208 | FJ556270 - FJ556337 |
| *Rhop148* | PF13_0348 | FJ556338 - FJ556418 |
| *ETRAMP10.2* | PF10_0323 | FJ555951 - FJ556028 |
| *AMA1* | PF11_0344 | FJ555752 - FJ555865 |
