# Supplementary material for: Prospective Identification of Malaria Parasite Genes under Balancing Selection
Source: PLoS One. 2009 May 15;4(5):e5568. doi: 10.1371/journal.pone.0005568 (PMC2679211; doi:10.1371/journal.pone.0005568)
Supplement: Table S4 — (0.06 MB DOC) [file pone.0005568.s010.doc]

**Supplementary Table S4: PCR conditions for *Plasmodium reichenowi* genes**

Tabulated details of conditions and primers used to generate *P. reichenowi* PCR products for sequencing

| **Gene Locus** | **Gene Description** | **PCR Conditions and Product generation primers (5’-3’)** |
| --- | --- | --- |
| **PFF0615** | Pf12/6-cys | 94°C^4min^ x 1 cycle-(94°C^1min^, 50°C^1min^, 72°C^1min^) x 45 cycles, 72°C^7min^ x 1 cycle  Pf12_F1 – gtaagaagtattgtttaggg  Pf12_R1- gatgaatgataagaatgatgtg |
| **PF13_0338** | Pf92/6-cys | 94°C^2min^ x 1 cycle-(94°C^15sec^- 55°C^30sec^ – 67.2°C^3min^) x 14 cycles –  (94°C^15sec^ – 55°C^30sec^ – 67.2°C^2min+5sec/cycle^) x 29 cycles -60.3°C^10min^ x 1 cycle  Pf92_F1 – atgtttgctgtaaacttaaa  Pf92_R1 - gaaagaagaaatagacaagg |
| **PFE0395c** | Pf38/6-cys | 94°C^4min^ x 1 cycle-(94°C^1min^, 50°C^1min^, 72°C^1min^) x 45 cycles, 72°C^10min^ x 1 cycle  Pf38_F1 – gctggtcaatcattacag  Pf38_R1 – gaatgcttgttaactccaata |
| **PF14_0201** | Pf113 | 94°C^2min^ x 1 cycle-(94°C^15sec^- 51.7°C^30sec^ - 72°C^2min^) x 14 cycles –  (94°C^15sec^ – 51.7°C^30sec^ - 72°C^2min+5sec/cycle^) x 29 cycles -72°C^7min^ x 1 cycle Pf14_0201_F1v2 - gttcacaaggaaacctgtatg  Pf14_0201_R1 - ctaagagtgcattactcatag |
| **PFF0995c** | MSP10 | 94°C^2min^ x 1 cycle-(94°C^15sec^, 48.4°C^30sec^, 72°C^1.15min^) x 14 cycles –  (94°C^15sec^, 48.4°C^30sec^, 72°C^1.15min+5sec/cycle^) x 29 cycles -72°C^7min^ x 1 cycle  MSP10_F1 - gcaaggtaaataatatgatg  MSP10_R1 - catatgaatcttcatttatcg |
| **PF10-0346** | MSP6 | 94°C^2min^ x 1 cycle-(94°C^15sec^, 48.4°C^30sec^, 72°C^1min^) x 14 cycles –  (94°C^15sec^, 48.4°C^30sec^, 72°C^1min+5sec/cycle^) x 29 cycles -72°C^7min^ x 1 cycle MSP6_KTR1 - ctaaatagatggatcatttcttg  MSP6_OUT_F2 – tcaaaatgaataagatttataatattac |
| **PF13_0197** | MSP7 | 94°C^2min^ x 1 cycle-(94°C^15sec^- 45.5°C^30sec^ - 72°C^1min^) x 14 cycles –  (94°C^15sec^ – 45.5°C^30sec^ - 72°C^1min+5sec/cycle^) x 29 cycles -72°C^7min^ x 1 cycle  MSP7_5’UTR F1 – gtgcaaatcaaatttttaaataatttg  MSP7 3’UTR_R1 – gaaaaaaaaataaagttgtacc |
| **PF13_0196** | MRSP1 | 94°C^4min^ x 1 cycle-(94°C^1min^, 62°C^1min^, 72°C^2min^) x 45 cycles, 72°C^10min^ x 1 cycle  MRSP1_R3 – catatatacatatgtatccactttgttc  MRSP1_F1 – catatatacatatattttatttgataag |
| **MAL13P1.174** | MRSP2 | 94°C^4min^ x 1 cycle-(94°C^1min^, 45.0°C^1min^, 72°C^1min^) x 45 cycles, 72°C^7min^ x 1 cycle  MRSP2 F3 – taaaatgaagggacaagcaatttatttcg  MRSP2 R3 - cctaaatagtatttattaaattaagagcattttc |
| **PF13_0193** | MRSP3 | 94°C^2min^ x 1 cycle-(94°C^15sec^- 59.6°C^30sec^ - 72°C^2min^) x 10 cycles –  (94°C^15sec^ – 59.6°C^30sec^ - 72°C^2min+5sec/cycle^) x 25 cycles -72°C^7min^ x 1 cycle MRSP3 F1v2 – gagaaaaaatgaaaggtagaattatatc  MRSP3 R3 - ctggtgatggtatggtttc |
| **MAL13P1.173** | MRSP4 | 94°C^4min^ x 1 cycle-(94°C^1min^, 45.0°C^1min^, 72°C^1min^) x 45 cycles, 72°C^7min^ x 1 cycle  MRSP4 F1v2 – gagaaaaaatgaaaggtagaattatatc  MRSP4 R2 – gtgtggtggtttaattaaaagg |
| **PF13_0191** | MRSP5 | 94°C^2min^ x 1 cycle-(94°C^15sec^- 50.5°C^30sec^ - 65°C^2min^) x 14 cycles –  (94°C^15sec^ – 50.5°C^30sec^ - 65°C^2min+5sec/cycle^) x 29 cycles -72°C^7min^ x 1 cycle MRSP5_R1 - tcatatggtatctaataagttaag  MRSP5_F1 - atgcaaagtgaattcttcatttg |
| **PF13_0192** | MRSP-like | 94°C^2min^ x 1 cycle-(94°C^15sec^- 45.0°C^30sec^ - 72°C^2min^) x 14 cycles –  (94°C^15sec^ – 45.0°C^30sec^ – 72°C^2min+5sec/cycle^) x 29 cycles -72°C^7min^ x 1 cycle  Pf13_0192 F9 – gcaaaaaaataatatgttcatcttg  Pf13_0192 R8 - cataatttcgtgggatttaaagc |
| **PF13_0194** | MRSP-like | 94°C^2min^ x 1 cycle-(94°C^15sec^- 45.0°C^30sec^ - 72°C^2min^) x 14 cycles –  (94°C^15sec^ – 45.0°C^30sec^ - 72°C^2min+5sec/cycle^) x 29 cycles -72°C^7min^ x 1 cycle  Pf13_0194 F1 – caatgaaaagaacaagacatattac  Pf13_0194 R1 - gtaaggtatctaaatgagaaag |

**Table S2 continued: PCR conditions for *Plasmodium reichenowi* genes**

| **Gene Name** | **Gene Description** | **PCR Conditions and Product generation primers (5’-3’)** |
| --- | --- | --- |
| **PF10_0347** | MSP3/6 like | 94°C^2min^ x 1 cycle-(94°C^15sec^- 48.4°C^30sec^ - 72°C^2min^) x 14 cycles –  (94°C^15sec^ – 40.5°C^30sec^ - 72°C^2min+5sec/cycle^) x 29 cycles -72°C^7min^ x 1 cycle  Pf10_0347_F1 - cgtgaatataatattttatatc  Pf10_0347_R1 - ccataaacctatgttttaatc |
| **PF10_0348** | MSP3/6 like | 94°C^2min^ x 1 cycle-(94°C^15sec^- 45.5°C^30sec^ – 72°C^3min^) x 14 cycles –  (94°C^15sec^ – 45.5°C^30sec^ – 72°C^3min+5sec/cycle^) x 29 cycles – 55.4°C^10min^ x 1 cycle Pf10_0348_F4 – tgattcgaatctaagaaacg  Pf10_0348_R1 - gaaataaatctgtcatatcttc |
| **PF10_0352** | MSP3/6 like | 94°C^2min^ x 1 cycle-(94°C^15sec^- 51.7°C^30sec^ - 72°C^2min^) x 10 cycles –  (94°C^15sec^ – 51.7°C^30sec^ - 72°C^2min+5sec/cycle^) x 25 cycles -72°C^7min^ x 1 cycle Pf10_0352_R1 - ctcattttctaaatcttttagtg  Pf10_0352_F1 - atgaataagtttttgaatattata |
| **PFL1385c** | MSP9/ABRA | 94°C^2min^ x 1 cycle-(94°C^15sec^, 60°C^30sec^, 72°C^2min^) x 14 cycles –  (94°C^15sec^, 60°C^30sec^, 72°C^2min+5sec/cycle^) x 29 cycles -72°C^7min^ x 1 cycle ABRA_FCC1_HN_F1 - gtttattgctctttgtcataagatg  ABRA_FCC1_HN_R2 - cagttgtcaaattttctggtac |
| **PFB0340c** | SERA5 | 94°C^2min^ x 1 cycle-(94°C^15sec^- 48.2°C^30sec^ – 65.0°C^2min^) x 14 cycles –  (94°C^15sec^ – 48.2°C^30sec^ - 65°C^2min+5sec/cycle^) x 29 cycles -72°C^7min^ x 1 cycle PFB0340c_F9 - gaagtcatatatttccttgtttttc  PFB0340c_R6 - caacactgtgaataaagttaaaatg |
| **MAL7P1.208** | RAMA | 94°C^2min^ x 1 cycle-(94°C^15sec^, 59.6°C^30sec^, 72°C^2min^) x 14 cycles –  (94°C^15sec^ – 59.6°C^30sec^ - 72°C^2min+5sec/cycle^) x 29 cycles -72°C^7min^ x 1 cycle RAMA_F1 – aaaatgaatgttctacttctgtctttgc  RAMA_R5 - gcagtcatagcggcttca |
| **PF13_0348** | Rhop148 | 94°C^2min^ x 1 cycle-(94°C^15sec^, 48.4°C^30sec^, 72°C^2min^) x 14 cycles –  (94°C^15sec^, 48.4°C^30sec^, 72°C^2min+5sec/cycle^) x 29 cycles -72°C^7min^ x 1 cycle Rhop148_F1 – gcatatattgaacatattgtac  Rhop148_R8 – gatagcattttatgacttaaaatattcc |
| **PF10_0144** | Prohibitin | 94°C^2min^ x 1 cycle-(94°C^15sec^- 45.5°C^30sec^ - 72°C^2min^) x 14 cycles –  (94°C^15sec^ – 45.5°C^30sec^ - 72°C^2min+5sec/cycle^) x 29 cycles -72°C^7min^ x 1 cycle Pf10_0144_F1 - tttaaatttaaattaaggaacatttac  Pf10_0144_R1 - gtaaaatttatcaacaacgaatc |
| **PF14_0102** | RAP1 | Derived from Genbank database: Accession number U20986  <http://www.ncbi.nlm.nih.gov/Genbank/> |
| **PFE0080c** | RAP2 | Derived from Sanger shotgun sequence: reich91f07.q1k  reich581b11.q1k  reich301f10.q1k  <http://www.sanger.ac.uk/>. |
| **PFE0075c** | RAP3 | Derived from Sanger shotgun sequence: reich308g09.p1k  reich451f07.p1k  reich29g09.q1k  reich1212e05.q1k  reich451g08.q1k  reich308g09.q1k  <http://www.sanger.ac.uk/>. |
| **PFD0955w** | Pf34 | 94°C^2min^ x 1 cycle-(94°C^15sec^, 45.5°C^30sec^ , 72°C^1min^) x 14 cycles –  (94°C^15sec^, 45.5°C^30sec^, 72°C^1min+5sec/cycle^) x 29 cycles -72°C^7min^ x 1 cycle PFD0955w_F1 - gtatggtacatttttgaagg  PFD0955w_R1 - gtattaacaaaaaaattatac |
